# Associations between Diet Quality and Global Cognitive Ability across the Life Course: Longitudinal Analysis of the 1946 British Birth Cohort

**DOI:** 10.1016/j.cdnut.2025.107619

**Published:** 2025-12-20

**Authors:** Kelly C Cara, Tammy M Scott, Mei Chung, Paul F Jacques

**Affiliations:** 1 Division of Nutrition Epidemiology and Data Science, Gerald J. and Dorothy R. Friedman School of Nutrition Science and Policy, Tufts University, Boston, MA, United States; 2 Department of Population Science, American Cancer Society, Atlanta, GA, United States; 3 Jean Mayer USDA Human Nutrition Research Center on Aging, Boston, MA, United States

**Keywords:** cohort studies, cognition, diet, healthy, Healthy Eating Index, group-based trajectory modeling

## Abstract

**Background:**

Diet is a risk factor for later-life cognitive decline and dementia. The long-term relationship between diet quality and cognitive function is unknown.

**Objectives:**

This study investigated trends in diet quality and cognitive ability and their interrelationship across the life course.

**Methods:**

Using data from the 1946 British Birth Cohort (*n* = 3059, 50.2% male), group-based trajectory modeling identified diet and cognitive trajectories from childhood to later adulthood, associations between those trajectories, and associations between diet trajectories and later indications of likely dementia. Healthy Eating Index-2020 scores were calculated from food recalls and diaries at ages 4, 36, 43, 53, and 60 to 64 y. Global cognitive ability percentile ranks were derived from tests of intellectual ability and cognitive function at ages 8, 11, 15, 43, 53, 60 to 64, and 68 to 69 y. Addenbrooke’s Cognitive Examination-III scores indicated likely dementia at age 68 to 69 y. Multinomial logit models determined early-life predictors of trajectory groups.

**Results:**

Three diet quality trajectories and 4 cognitive ability trajectories were identified. Sex, birth region, childhood social class, and leisure activities predicted trajectory group membership. In a joint trajectory model, the lowest cognitive ability group included mostly participants with lower (58%) or moderate (35%) diet quality. Conversely, the highest cognitive ability group included mostly participants with moderate (57%) and higher (36%) diet quality. The percentage of participants showing indications of likely dementia at age 68 to 69 y was 3.8% to 7.4% greater in the lower diet quality group compared with the moderate and higher groups, respectively.

**Conclusions:**

Findings indicate a link between diet quality and cognitive ability across the life course and a higher chance of likely dementia in individuals with lower diet quality from childhood to later adulthood. Consistent dietary alignment with dietary guidelines over time may positively impact cognitive outcomes throughout life, but more longitudinal studies are needed to confirm these findings.

## Introduction

Due to changes that occur in the brain over time, gradual cognitive decline is a natural part of aging [1]. However, more severe decline than expected could be a sign of age-related degenerative brain diseases, like Alzheimer’s disease (AD) and Alzheimer’s disease-related dementias (ADRD). These conditions have become more common as our population has gotten older. Since 1990, AD and other dementias have been on the rise in the United States and globally [2,3]. Known AD/ADRD risk factors include nonmodifiable factors (e.g. age, sex, genetics, early social and material environment) and potentially modifiable factors (e.g. education, occupation, health behaviors and related conditions, and lifestyle) [4,5]. Lifestyle behaviors presenting in midlife, such as smoking, high alcohol intake, lack of physical activity, and poor diet, can especially increase the risk of AD [[5], [6], [7]].

Dietary patterns that have been associated with lower levels of cognitive decline and dementias include those aligned with the Dietary Guidelines for Americans (DGA), Mediterranean-style diets, the Dietary Approaches to Stop Hypertension (DASH) diet, and the Mediterranean-DASH Intervention for Neurodegenerative Delay (MIND) diet [7,8]. This suggests that dietary changes have the potential to improve or maintain cognitive health, yet most studies in this area focus on older populations when efforts to prevent cognitive decline may be less effective. An extensive systematic review of dietary patterns and cognitive decline searched for studies in populations as young as 40 y old [8]. Still, the 50 observational studies and 4 randomized controlled trials identified for the review were mostly in populations aged ≥60 y. Symptoms of late-onset AD, the most common form, can begin after age 65 y. However, 15 to 20 y before AD symptoms onset, preclinical AD symptoms can present, such as altered cerebrospinal fluid (CSF) levels of Alzheimer pathology biomarkers (e.g. CSF tau and CSF Ab42) [9]. Therefore, dietary improvements up to midlife, and well before age 65 y, may be most effective at preventing, slowing, or halting deleterious cognitive outcomes, whereas improvements to diet after this period may be less impactful. This was demonstrated in the Finnish Geriatric (FINGER) Intervention Study to Prevent Cognitive Impairment and Disability, where dietary changes initiated in participants at age 60 to 77 y were related to changes in executive function after 2 y, but long-term dietary habits—those followed before the study—appeared to have the greatest impact on global cognition [10].

Connections between diet quality and cognitive ability over the life course have not been extensively studied. Furthermore, it is not clear if maintaining a healthy dietary pattern up to midlife can impact dementia risk. Understanding this could inform whether research, clinical approaches, policies, and programs aimed at preventing neurodegenerative diseases with long developmental periods, like AD/ADRD, should focus on diet in the first half of life. The primary aims of this study were to identify trends in diet quality and global cognitive ability across the life course and to investigate associations between these trends. We hypothesized that diet quality from early childhood to later adulthood would be positively associated with global cognitive ability trends. We also aimed to explore the association between diet trajectories up to age 60 to 64 y and likely dementia at age 68 to 69 y, which we hypothesized would show an inverse association.

## Methods

### Study population

The National Survey of Health and Development (NSHD) by the Medical Research Council (MRC) is a prospective study of the British Birth Cohort that followed the 1946 Maternity Survey [11]. British Birth Cohort participants were randomly sampled to represent all regions of England, Wales, and Scotland and all parts of society based on socioeconomic status and family circumstances. The original sample included 5362 singleton infants (52% male) born during 1 wk in March of 1946 and drawn from 16,695 total births in the British birth registries [12]. Data from this cohort have been collected via interviews, medical exams, and postal questionnaires 25 times >75 y [12]. During the 24th follow-up (age 68–69 y), 2816 study members still lived in mainland Britain, and 2638 contributed data. Others had died (*n* = 957), had withdrawn permanently from the study (*n* = 620), lived abroad (*n* = 574), or were untraceable for more than 5 y (*n* = 395) [13]. To be included in the present analysis, participants needed complete data at birth, dietary data from ≥2 data collection periods, and sufficient cognitive data to produce at least 1 global cognitive score in childhood (age 8–15 y) and 1 in adulthood (age 43–69 y).

### Ethics approval and consent to participate

The cohort members, or their mothers when participants were children [12], have given written informed consent to participate in each data collection period of the study [13]. Throughout the study, participants have also consented to having their data stored according to guidelines of the Data Protection Act [14]. Ethical approval for NSHD has been provided by Research Ethics Committees in both England and Scotland throughout the study duration [13,14]. The present study received ethical approval from the MRC Unit for Lifelong Health and Ageing at University College London to utilize NSHD data from 1946 to 2005 [15], 2006 to 2012 [16], and 2013 to 2018 [17]. This study was also granted exempt status from review by the Tufts University Social, Behavioral & Educational Research institutional review board (SBER IRB number: STUDY00002289).

### Dietary assessments

Dietary intake was assessed on 5 occasions in the cohort, and this study utilized available dietary data from all 5 data collection periods [18]. At age 4 y, mothers or caregivers of participants completed a 24-h dietary recall on behalf of the cohort member. This recall was recorded by a research staff member who asked for a report of all foods and beverages consumed by the child across meals and between meals in the 24-h period before to the interview. At ages 36 and 43 y, participants completed a 48-h (2-d) dietary recall during a home visit by a research nurse, then they completed self-administered food diaries for the subsequent 5 d. At ages 53 y and 60 to 64 y, participants again completed 5-d food diaries. Furthermore, details on all dietary measurements are published online [18]. The MRC in-house software, Diet In Nutrients Out, was used to code the recalls and diaries and produce nutrient data based on British food composition tables relevant for each year of data collection [19]. For the present study, cohort members with ≥1 d of dietary intake data from ≥2 data collection years were analyzed without exclusions (e.g. very low or high calories).

### Diet quality

Diet quality was identified using the Healthy Eating Index (HEI)-2020 which compares dietary intake with the DGA, 2020 to 2025 [20,21]. Although this index was designed for the United States population, it measures culturally neutral food groups and has been used to assess diet quality in many other countries [22]. The HEI-2020 scoring standards organize these food groups into 13 components including 9 adequacy components, which indicate foods to eat more of (total fruits, whole fruits, total vegetables, greens and beans, whole grains, dairy, total protein foods, seafood and plant proteins, and fatty acids), and 4 moderation components, which are foods to limit (refined grains, sodium, added sugars, and saturated fats). Most component scores are based on dietary intakes measured in cup- or ounce-equivalents per 1000 kcal of total energy. The fatty acids score is based on a ratio of total unsaturated (PUFA and MUFA) to SFAs. Sodium is scored based on grams of intake per 1000 kcal of total energy. Scores for added sugars and saturated fats are based on percentages of total energy. HEI-2020 component scores range from 0 to 5 or 0 to 10, and total scores range from 0 to 100 with higher values indicating greater dietary alignment with the DGA [20].

To assess intake of HEI components in this study, we first manually matched the NSHD food items data (excluding dietary supplements) with the closest matching descriptions of foods from the 2015 to 2016 USDA Food Patterns Equivalents Database (FPED 1516) and Food Patterns Equivalents Ingredients Database (FPID 1516) [23]. Our crosswalk for the NSHD integration with FPED/FPID is provided as Supplemental data. Because foods in the United Kingdom and United States food systems vary in nutrient profiles, we pulled only the HEI food group components data from FPED and FPID (e.g. cup and ounce-equivalents for fruit, dairy, nuts, etc.), and we used the NSHD nutrient data to calculate total dietary energy (kcal), sodium, fatty acids, and added sugars. In some years, the NSHD data showed 0 added sugars for foods identified as nutritive sweeteners (e.g. table sugar, honey, and syrups). For these, we substituted NSHD added sugars data from previous years, when available. Otherwise, we used FPED and FPID to estimate added sugars. We then used the FPED/FPID food group component values and the NSHD nutrients data with corrected added sugars to create HEI-2020 scores. When multiple days of data were reported in a single data collection period (e.g. 5-d food diaries), we summed intakes for each HEI component across all days per person using the method for “describing dietary intake” recommended by the NIH National Cancer Institute (NCI) [24]. We then applied the HEI-2020 scoring algorithm to create mean component and mean total scores for each data collection year [20].

### Cognitive measures

Cognitive ability in the cohort was assessed on 8 occasions, when participants were 8, 11, 15, 26, 43, 53, 60 to 64, and 68 to 69 y old. From childhood to age 26 y, cognitive function measures focused on intellectual ability and primarily included tests created by the National Foundation for Educational Research in England and Wales to assess verbal and nonverbal abilities [25]. At age 43 y, the study’s focus shifted to measures of functional cognitive performance like memory and processing speed [26]. Details for all cognitive measures are published online [27], and brief descriptions are provided below and in Supplemental Table 1.

At age 8, participants were given verbal and nonverbal ability tests which assessed reading comprehension, word reading and pronunciation, vocabulary, and nonverbal reasoning based on evaluating pictures [28]. Age 11 y assessments included tests of general ability, arithmetic, word reading, and vocabulary. When participants were of age 15 y, they took the Alice Heim Group Ability Test [29], the Watts-Vernon Reading Test [30], and a mathematics test. The Watts-Vernon Reading Test was a timed reading comprehension test used in national surveys of schoolchildren in England and Wales [31], and this test was again administered to participants at age 26 y but with 10 additional items of increased difficulty [26].

Starting at age 43 y, cohort members were given a series of cognitive function tests devised by NSHD including a verbal memory word list test and a timed visual search speed test that were repeated at ages 53, 60 to 64, and 68 to 69 y [26]. At age 43 y, participants were also tested on long-term recall, visual memory, and motor speed and praxis. At age 53 y, participants were additionally given an animal naming test to assess verbal fluency, a prospective memory and delayed verbal memory test, and the National Adult Reading Test [32]. The only additional test at age 60–64 was a reaction time test. At age 68–69, participants were additionally given a finger tapping test to assess psychomotor speed, and they took Addenbrooke’s Cognitive Examination version III (ACE-III) to demonstrate verbal and nonverbal ability [33]. The ACE-III total score ranges from 0 to 100 and comprises subscores from 5 domains: attention and orientation (0–18 possible points), verbal fluency (0–14 points), memory (0–26 points), language (0–26 points), and visuospatial function (0–16 points). ACE-III total scores at a threshold of 82 are used to identify dementia and mild cognitive impairment for patients in clinical settings [34]. In the absence of other clinical measures to determine dementia in this cohort, we categorized participants with ACE-III total scores <82 as showing indications of likely dementia.

### Global cognitive ability

We pooled all cognitive measures available in this cohort to derive global cognitive ability at each age. To create global scores, we first calculated percentile ranks for each available cognitive measure. This provided a standardized metric, because the cognitive measures had various units and scales, and it established a ranking of cognitive abilities among participants. Next, for participants with ≥2 cognitive scores in a given year, we averaged their percentile ranks. Therefore, global cognition was represented by the mean of ≥2 percentile rank scores at each data collection period. Those with <2 scores were counted as missing for that year. We did not create a global score for age 26 y, when only 1 cognitive assessment was given. We also excluded the age 43 y long-term recall test which could have been influenced by guessing. Those scores were based largely on remembering common biometric measures taken during the age 36 interview (e.g. pulse, blood pressure, height, weight). In sensitivity analyses, we created a global cognitive ability score for age 68 to 69 y that excluded the ACE-III total score because it was also used as an indicator of likely dementia, a secondary outcome of interest in this study.

### Covariates

In this cohort, several early-life characteristics and experiences were measured. Those thought to influence dietary intake and cognitive ability were examined in this study as potential predictors of trends in diet and cognition over time. In particular, factors that may have influenced early-life resources and experiences of the study participants included birth sex (male, female), region of birth (Wales, Northern, Yorkshire and Humberside, Northwest England, East Midlands, West Midlands, East Anglia, Southeast England, Southwest England, and Scotland), and childhood social class [professional, intermediate, skilled (nonmanual), skilled (manual), partly skilled, unskilled, armed forces, not working or never worked full-time, and unknown or no valid social class]. The largest proportion of participants was from Southeast England (including London), so we created a binary variable to distinguish those born in Southeast England from other regions. Childhood social class was derived from the fathers’ occupations when participants were age 11 y (if missing, age 15 y then age 4 y), and we created a binary indicator to identify participants from households in the “skilled (nonmanual)” class and above.

We also considered early-life BMI and participation in intellectual and social leisure activities as potential contributors to cognitive trends in the cohort. BMI (kg/m^2^) was calculated from height and weight measured by research nurses when participants were aged 4 y. Involvement in various leisure activities (yes/no) was reported when participants were aged 11 y. We checked point-biserial correlations (*r*_*pb*_) to first determine whether these activities were related to global cognitive ability at age 11 y. After confirming correlations, we summed the number of activities reported by each participant and classified them as intellectual (e.g. academic activities, arts) or social (e.g. clubs, sports) activities. Supplemental Table 2 presents the age 11 y leisure activities, their correlations with global cognitive ability, and their assigned classifications.

### Statistical analyses

This study followed participants from birth in March of 1946 through 2015 (the most recently available data) or the last date participants contributed cognitive data after age 43 y. To summarize participant characteristics, we have reported total sample size and percentage providing data at each data collection period along with *n* (%) for categorical measures and mean (SD) for continuous measures. For each cognitive measure, we have also reported medians and IQR from the analytical sample. We compared baseline characteristics for participants included in or excluded from the analytical sample using Pearson’s chi-squared (χ^*2*^) tests for categorical measures, and independent means *t*-tests (*t*) with unequal variances assumed for continuous measures, where *P <* 0.05 indicated statistically significant differences. To distinguish potentially meaningful differences, we calculated effect sizes using Cramér’s V (following χ^*2*^) and Cohen’s *d* (following *t*-tests), where *V* was interpreted based on degrees of freedom (*df*) using standard tables [35,36], and *d* was interpreted as small (0.2), medium (0.5), or large (0.8) based on conventions [37]. Except when generating HEI scores, all data management and analyses were performed using Stata software (version 18.0 for Windows, StataCorp LLC). Scoring for the 2015 and 2020 versions of the HEI is identical, so we created HEI component and total scores with the HEI-2015 Scoring Macro (version 1.0, 06/25/2017) provided by the NCI [38] using SAS software (version 9.4, SAS Institute, Inc.).

Group-based trajectory modeling (GBTM) was used to identify variations in dietary and cognitive trends over the life course in the analytical cohort. GBTM is a semiparametric finite mixture model technique, where maximum likelihood estimation is used to approximate lines of best fit (i.e. trajectories) to distinguish individuals showing similar trends in data over time [39]. The number and shape of trajectories examined in the model should be informed by pre-established content knowledge. In this study, diet trajectories were informed by United States trends showing higher diet quality in early and late life with lower diet quality in teen years and early adulthood [21]. We also drew knowledge from a prior study of the 1946 British Birth Cohort which found a general increase in diet quality over adulthood [40]. The cognitive trajectories were informed by aging research which suggests general improvements in cognitive ability from childhood to adulthood followed by various degrees of decline in later life due to individual differences in the accumulation, maintenance, and deployment of neural resources [41].

Modeling trends over time with GBTM requires 2 or more waves of data collection and a continuous or ordered (nominal or ordinal) outcome [39]. Because our aims were to separately and concurrently examine dietary and cognitive trends, our analytical sample included only participants with both HEI scores and global cognitive ability scores from ≥2 data collection periods (with at least 1 cognitive score in both childhood and adulthood). Participants lacking 2 scores for both outcomes were excluded. We followed standard GBTM methods for a 3-step process which first determines the number and shape of trajectories, then identifies significant time-stable predictors, and lastly combines trajectories and predictors in a final model [42]. For the GBTM models, participants’ HEI-2020 total scores and global cognitive ability percentile rank scores were dependent variables, and their age at data collection was an independent variable. To conduct the analyses, we used the traj plugin in Stata [43,44] and specified a censored normal distribution with lower and upper bounds defined at 0 and 100 to accommodate the full range of possible scores for the HEI-2020 and cognitive percentile ranks. The details of our process are provided below.

In analysis step 1, we first identified the number of trajectories, restricting all to cubic polynomial orders, and then we adjusted the polynomial orders to determine the best-fitting shape for each trajectory. When selecting the number of trajectories, we aimed to maintain a balance between interpretability, model fit, and classification certainty, as is recommended [42]. We modeled 2, 3, 4, 5, and 6 groups, but we did not go beyond 6 groups to avoid compromising interpretability. Bayesian Information Criterion (BIC) determined model fit, and model comparisons were based on Jeffrey’s scale of evidence for Bayes factors approximated as $e^{{BIC}_{i}-{BIC}_{j}}$, where BIC_*i*_ represents the model with more groups, and BIC_*j*_ represents the model with fewer groups [42]. Entropy determined classification certainty based on participants’ posterior probabilities of group membership, where higher entropy values indicated greater accuracy [45]. After determining the number of groups, we systematically adjusted each trajectory’s polynomial order until all groups showed statistical significance at *P <* 0.05, membership ≥5%, and good fit based on visual assessment, and until a model meeting these criteria with the best (lowest) BIC emerged.

To reduce the chance of developing spurious trajectories, we assessed overall model adequacy using multiple statistical and visual measures of model fit, as recommended [45]. In GBTM, participants are assigned to the group where they have the highest posterior probability of membership. We calculated expected group membership as each group’s maximum posterior probability divided by the total sample size. The difference between actual and expected group membership was measured by “mismatch,” where values closer to 0 suggested more adequate fit. An average posterior probability >70% for each trajectory group indicated adequate classification. Relative entropy >0.8 suggested classification certainty across all groups. Supplemental Tables 3–5 show these adequacy measures for each tested model. To visually check the fit of selected trajectories, we used spaghetti plots and scatter plots with prediction lines for trends over time (Supplemental Figures 1–4). We created line graphs depicting the selected dietary and cognitive trajectory groups along with their expected group membership percentages. In sensitivity analyses, we used Cohen’s kappa-statistic (κ) to check agreement between participants’ cognitive trajectory group assignments based on global cognitive ability scores that included or excluded the ACE-III, where κ was interpreted from standard tables [46].

Moving on to analysis step 2, we performed unadjusted multinomial logit models using mlogit in Stata to identify significant time-stable predictors of trajectory group membership for the dietary and cognitive outcomes. Each model’s dependent variable was the trajectory group assignment from step 1, and the independent variable was a hypothesized early-life predictor (see covariates above). For both dietary and cognitive outcomes, we examined sex, birth region, childhood social class, and age 4 BMI as hypothesized predictors. For cognitive trajectories, we additionally examined age 4 HEI scores and the number of social and intellectual activities at age 11 y. Participants missing data for a given predictor were excluded from that analysis only. Results showing *P <* 0.05 were considered significant.

In analysis step 3, we added all significant predictors from step 2 as covariates to the trajectory parameters from step 1 using GBTM to create final models for the diet and cognition outcomes. This step determines which trajectory a participant is most likely to follow based on their time-stable predictors. We converted the resulting log-odds and SEs to odds ratios (ORs) and 95% confidence intervals (CIs), where results indicate the likelihood of being in each trajectory group relative to the reference group, given the predictor. Adding time-stable predictors to the GBTM analysis does not change the number or shape of the trajectories [42], but the percentage of participants expected in each trajectory group may shift.

In a fourth analysis step [42], we conducted a joint trajectory model combining the diet and cognitive trajectories to analyze their connections over time and test our hypothesis that dietary trends would be positively associated with global cognitive ability trends. Here, we used the unadjusted models from step 1 (no missing data) to retain the full analytical cohort. Following this analysis, we calculated χ^*2*^ for the diet and cognitive group membership assignments from the joint model to identify if and where membership probabilities differed from expected. These findings are presented in bar plots, and radar plots depict differences in HEI-2020 component scores across the cognitive trajectory groups. Repeated-measures analysis of variance (ANOVA) tests were conducted to examine trends in HEI-2020 and global cognitive ability scores over time within each trajectory group.

Finally, to examine our aim of exploring the association between diet quality trends and likely dementia, we analyzed a subgroup of participants with ACE-III total scores. Because the diet trajectory terminated at age 60 to 64 y, and likely dementia (ACE-III <82 points) was captured at age 68 to 69 y, we used GBTM to model likely dementia as a subsequent cross-sectional outcome on the unadjusted (step 1) and adjusted (step 3) diet trajectories. The logit model type was selected in Stata to accommodate the binary dementia indicator. The resulting OR and 95% CI indicate the chance of having likely dementia (versus not having likely dementia) in each diet trajectory group. We subsequently calculated χ^*2*^ to compare the observed and expected number of participants with likely dementia in each diet trajectory group.

## Results

### Participant characteristics

**FIGURE 1:**
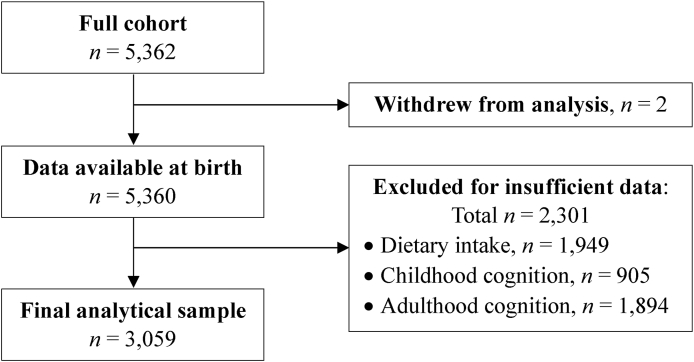
Selection process for the final analytical sample of 1946 British Birth Cohort members.

Of the 5362 participants in the original 1946 British Birth Cohort, 2 withdrew from the present study, and we excluded 2301 participants with incomplete or missing data for diet (*n* = 1949), childhood cognition (*n* = 905), and/or adulthood cognition (*n* = 1894). The remaining 3059 participants (50.2% males) were included in this study. Figure 1 presents the selection process, and Table 1 presents early-life characteristics. Compared with excluded participants, the analytical sample had more females (49.8% compared with 44.4%), higher mean global cognitive scores at age 8 y (51.1 compared with 47.7; 0–100 possible), and more types of social activities reported on average at age 11 y (0.81 compared with 0.74; 0–2 possible). Many fewer included participants had “unknown” childhood social class (1.4% compared with 28.1%), but when all participants with unknown status were removed (*n* = 689), social class distributions were similar for included and excluded participants. Distributions across regions of birth also differed, but patterns across regions were similar for included and excluded participants and resembled those of the full cohort. There were no differences in mean BMI and HEI scores at age 4 or mean number of intellectual activities at age 11 y.

**TABLE 1:** Early-life characteristics for the full cohort with comparisons for those included in or excluded from the analytical sample.

Characteristics	Full cohort (*n* = 5360)		Excluded (*n* = 2301)		Included (*n* = 3059)		Comparisons^1^		
	*n*	*%*	*N*	*%*	*n*	*%*	χ^2^	*P*	V
Categorical measures									
Sex							15.38	<0.001	0.05
Male	2814	52.5	1279	55.58	1535	50.18	—	—	—
Female	2546	47.5	1022	44.42	1524	49.82	—	—	—
Region of birth							27.62	0.001	0.07
Wales	287	5.35	105	4.56	182	5.95	—	—	—
Northern	390	7.28	151	6.56	239	7.81	—	—	—
Yorkshire and Humberside	449	8.38	190	8.26	259	8.47	—	—	—
Northwest England	556	10.37	245	10.65	311	10.17	—	—	—
E Midlands	342	6.38	129	5.61	213	6.96	—	—	—
W Midlands	471	8.79	216	9.39	255	8.34	—	—	—
E Anglia	191	3.56	79	3.43	112	3.66	—	—	—
Southeast England	1712	31.94	763	33.16	949	31.02	—	—	—
Southwest England	306	5.71	110	4.78	196	6.41	—	—	—
Scotland	656	12.24	313	13.6	343	11.21	—	—	—
Childhood social class, 11 y							839.17	<0.001	0.40
Professional etc.	289	5.39	104	4.52	185	6.05	—	—	—
Intermediate	907	16.92	321	13.95	586	19.16	—	—	—
Skilled (nonmanual)	729	13.6	235	10.21	494	16.15	—	—	—
Skilled (manual)	1554	28.99	569	24.73	985	32.2	—	—	—
Partly skilled	881	16.44	306	13.3	575	18.8	—	—	—
Unskilled	311	5.8	120	5.22	191	6.24	—	—	—
Unknown	689	12.85	646	28.07	43	1.41	—	—	—
Continuous measures	*n*	Mean (SD)	*N*	Mean (SD)	*n*	Mean (SD)	*t*	*P*	*d*
BMI (kg/m^2^), 4 y	4182	16.19 (1.67)	1445	16.17 (1.70)	2737	16.20 (1.65)	–0.52	0.60	0.02
HEI total scores (0–100 possible), 4 y	4597	45.89 (7.50)	1609	45.75 (7.44)	2988	45.97 (7.53)	–0.94	0.35	0.03
Global cognitive scores (0–100 possible), 8 y	4258	50.01 (24.70)	1330	47.71 (25.55)	2928	51.05 (24.24)	–4.02	<0.001	0.13
Intellectual activities (*n* of 3), 11 y	3573	0.79 (0.60)	1087	0.78 (0.60)	2486	0.80 (0.60)	–0.97	0.33	0.04
Social activities (*n* of 2), 11 y	4222	0.79 (0.71)	1299	0.74 (0.70)	2923	0.81 (0.71)	–2.76	0.006	0.09

Abbreviations: HEI, Healthy Eating Index.

1 Comparisons between excluded and included participants were conducted with the following tests. For categorical measures: Pearson’s chi-squared tests with Cramér’s *V* effect sizes. For continuous measures: Independent means *t*-tests with unequal variances assumed and Cohen’s *d* effect sizes.

Of the participants included in this study, 96% contributed data up to age 60 to 64 y, whereas 64% contributed data to age 68 to 69 y. At baseline, sex was evenly represented (49.8% female), but by age 68 to 69 y, the distribution shifted slightly toward females (51.6%). For all 5 data collection periods with dietary assessments, the analytical sample showed good response rates with the greatest variability in dietary reporting at age 43 y (Table 2). Overall, average daily caloric intake was the lowest at age 4 y (1444.32 kcal/d), peaked at age 43 y (2063 kcal/d), then decreased again by the age 60 to 64 y (1870 kcal/d). Mean (SD) HEI-2020 total scores increased in stepwise fashion from 45.97 (7.53) at age 4 y to 63.55 (12.58) at age 60 to 64 y, which suggests improvements in overall diet quality but with greater variability over time. Supplemental Figure 5 presents radar plots depicting mean HEI component scores for the analytical cohort at each data collection period. Over time, mean scores improved for most dietary components, but scores for fatty acids, saturated fats, total protein foods, and greens and beans remained relatively low. For cognitive measures, participant response rates were again high in the analytical cohort. The 3 data collection periods in childhood showed nearly complete responses, and response rates were slightly lower during the 4 data collection periods in adulthood (Table 3). Because global cognitive ability scores were based on percentile ranks, mean scores remained fairly stable over time. However, scores in childhood were higher on average and variability was greater (mean range: 51.05–51.42; SD range: 24.24–24.84) compared with adulthood (mean range: 49.63–49.88; SD range: 17.01–19.68).

**Table 2:** Dietary assessments, daily energy intake, and healthy eating index-2020 total scores across data collection periods for the full analytical cohort and trajectory groups.

Measures	Age at data collection					*F*_within_^1^
	4 y	36 y	43 y	53 y	60–64 y	
Dietary assessments						
Tools used	24-h recall by caregiver	5-d food diaries; 48-h recall	5-d food diaries; 48-h recall	5-d food diaries	5-d food diaries	
Reporting days						
Total days possible, count^2^	1	7	7	5	5	
Total days reported by participating cohort members						
≥2 d, *n* (%)	–	2162 (99.91)	2895 (99.42)	1634 (99.88)	1730 (100.00)	
All days possible, *n* (%)	2988 (100.00)	1788 (82.62)	1914 (65.73)	1622 (99.14)	1682 (97.23)	
Total days used for HEI-2020 scores, mean (SD)	1.00 (0.00)	6.67 (0.78)	5.48 (2.24)	4.99 (0.18)	4.97 (0.21)	
Weekdays (Mon-Fri), mean (SD)	0.96 (0.19)	4.79 (0.58)	3.94 (1.67)	3.54 (0.73)	3.19 (0.55)	
Weekend days (Sat-Sun), mean (SD)	0.04 (0.19)	1.88 (0.42)	1.54 (0.79)	1.45 (0.73)	1.78 (0.54)	
Daily energy intake (kcal), mean (SD)	1444.32 (344.65)	2035.90 (633.62)	2062.88 (653.81)	1985.13 (513.03)	1869.94 (460.31)	
HEI-2020 Total scores (0–100)						
Full analytical cohort						
*n*	2988	2164	2912	1636	1730	924.00
Mean (SD)	45.97 (7.53)	48.17 (10.36)	50.15 (11.56)	57.18 (12.31)	63.55 (12.58)	
Diet quality trajectory						
Group 1 (lower)						
*n*	891	600	860	423	436	166.51
Mean (SD)	44.49 (7.56)	39.68 (6.79)	39.46 (7.22)	43.95 (7.46)	49.29 (8.42)	
Group 2 (moderate)						
*n*	1596	1159	1550	874	943	1063.69
Mean (SD)	46.44 (7.30)	48.22 (7.99)	51.23 (7.91)	58.17 (8.47)	65.47 (8.49)	
Group 3 (higher)						
*n*	501	405	502	339	351	810.26
Mean (SD)	47.08 (7.80)	60.58 (7.86)	65.10 (8.38)	71.13 (7.84)	76.13 (8.80)	
All groups, *F*_between_^3^	26.18	902.57	1745.73	1075.40	1012.27	
Cognitive ability trajectory						
Group 1 (lower)						
*n*	763	504	748	334	321	175.95
Mean (SD)	44.50 (7.22)	44.18 (9.63)	45.57 (10.91)	53.72 (12.58)	59.58 (13.18)	
Group 2 (low-moderate)						
*n*	616	452	600	343	369	185.18
Mean (SD)	45.98 (7.54)	46.72 (10.29)	49.80 (11.54)	56.09 (12.00)	62.66 (12.40)	
Group 3 (high-moderate)						
*n*	755	550	731	409	449	227.64
Mean (SD)	46.32 (7.37)	48.83 (10.02)	50.60 (11.22)	56.83 (12.52)	63.65 (12.59)	
Group 4 (higher)						
*n*	854	658	833	550	591	358.78
Mean (SD)	46.96 (7.73)	51.65 (9.98)	54.11 (10.94)	60.20 (11.48)	66.20 (11.70)	
All groups, *F*_between_^3^	15.40	57.61	78.15	21.62	20.69	

Abbreviations: ANOVA, analysis of variance; HEI, Healthy Eating Index.

1 Within-group mean comparisons performed with repeated-measures ANOVA (*F*) tests; *P*_trend_ < 0.0001 for all.

2 Up to 5 d of dietary intake data were collected at age 60 to 64 y, but 1 cohort member provided 7 d of data.

3 Between-group mean comparisons performed with 1-way ANOVA (*F*) tests; *P*_trend_ < 0.0001 for all.

**TABLE 3:** Cognitive measures and global cognitive ability scores across data collection periods for the full analytical cohort and trajectory groups.

Measures	Age at data collection							*F*_within_^1^
	8 y	11 y	15 y	43 y	53 y	60–64 y	68–69 y	
Cognitive measures								
Total possible, count^2^	4	5	4	4	6	4	5	
Used for GCA scores, mean (SD)	4.00 (0.04)	4.99 (0.14)	4.00 (0.08)	3.91 (0.31)	5.71 (0.55)	3.94 (0.30)	4.72 (0.62)	
GCA scores (0–100)^3^								
Full analytical cohort								
*n*	2928	2825	2816	2936	2625	2003	1920	
Mean (SD)	51.05 (24.24)	51.39 (24.84)	51.42 (24.28)	49.88 (17.01)	49.72 (17.40)	49.63 (19.68)	49.71 (18.21)	4.01^4^
Cognitive ability trajectory								
Group 1 (lower)								
*n*	745	716	718	752	641	404	392	
Mean (SD)	24.27 (13.94)	21.03 (11.55)	22.38 (12.92)	37.78 (15.60)	30.51 (11.21)	29.21 (13.16)	32.26 (14.78)	141.99
Group 2 (low-moderate)								
*n*	605	582	580	603	546	416	388	
Mean (SD)	38.95 (13.01)	39.68 (11.47)	41.84 (13.18)	54.65 (13.61)	49.94 (11.18)	52.75 (13.75)	52.58 (13.32)	154.38
Group 3 (high-moderate)								
*n*	742	729	718	740	665	528	498	
Mean (SD)	60.97 (14.23)	62.03 (11.97)	60.82 (12.65)	44.79 (14.17)	47.78 (12.01)	41.34 (13.43)	43.40 (13.88)	320.47
Group 4 (higher)								
*n*	836	798	800	841	773	655	642	
Mean (SD)	74.87 (13.52)	77.44 (11.35)	75.98 (12.25)	61.76 (13.14)	67.17 (9.88)	66.91 (13.95)	63.54 (13.91)	198.49
All groups, *F*_between_^5^	2075.86	3386.55	2483.46	435.16	1296.06	727.36	455.79	
Diet quality trajectory								
Group 1 (lower)								
*n*	864	854	841	865	754	514	476	
Mean (SD)	43.56 (23.65)	42.76 (24.59)	43.48 (24.34)	46.16 (16.49)	43.27 (16.72)	42.64 (19.08)	45.52 (18.63)	3.05^6^
Group 2 (moderate)								
*n*	1564	1502	1502	1567	1391	1087	1062	
Mean (SD)	52.48 (23.74)	53.12 (24.19)	53.34 (23.75)	50.37 (17.03)	50.76 (16.88)	50.52 (19.37)	50.26 (17.91)	6.02
Group 3 (higher)								
*n*	500	469	473	504	480	402	382	
Mean (SD)	59.53 (23.21)	61.55 (22.26)	59.44 (21.90)	54.76 (16.44)	56.84 (16.50)	56.15 (18.53)	53.42 (17.55)	9.68
All groups, *F*_between_^5^	78.47	101.03	79.70	43.31	101.68	58.84	21.45	

Abbreviations: ANOVA, analysis of variance; GCA, global cognitive ability.

1 Within-group mean comparisons performed with repeated-measures ANOVA (*F*) tests; *P*_trend_ < 0.0001 for all except where noted.

2 At age 43 y, 1 of 5 available measures was deemed unsuitable for analysis; at age 53 y, 1 of 7 available measures produced no score.

3 Scores were calculated as an average of ≥2 available cognitive measures each year; the single measure at age 26 y was excluded.

4 *P*_trend_ < 0.01.

5 Between-group mean comparisons performed with 1-way ANOVA (*F*) tests; *P*_trend_ < 0.0001 for all.

6 *P*_trend_ < 0.001.

### Diet quality trajectory groups

**FIGURE 2:**
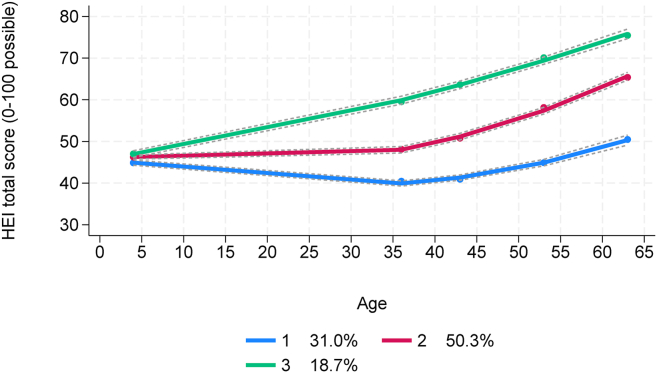
Diet trajectory groups from unadjusted group-based trajectory modeling analysis (*n* = 3059) with trends in hei-2020 total scores from age 4 y to 60 to 64 y. Lines = estimated trajectories with 95% CIs, dots = observed group means, legend presents expected group membership percentages. CI, confidence interval; HEI, Healthy Eating Index.

Using group-based trajectory modeling, participants were assigned to 1 of 3 distinct diet quality trajectories based on their trends in HEI-2020 total scores, as depicted in Figure 2. These trends differentiated participants whose diet quality was consistently lower (group 1), moderate (group 2), or higher (group 3) over time. Expected group membership was greatest for those with moderate diet quality (50.3%), and this was followed by those with lower (31%) and then higher (18.7%) diet quality. Table 2 presents mean HEI-2020 total scores for the trajectory groups at each data collection period along with results from 1-way ANOVAs (examining means between groups at each age) and repeated-measures ANOVAs (examining means within groups over time). Although diet quality appeared similar for all groups at age 4 y, their mean HEI scores differed significantly (*F*_between_ = 26.18, *P*_trend_ < 0.0001) and increased in stepwise fashion from group 1 to group 3. After age 4 y, the stepwise pattern persisted, but the difference between mean HEI scores became more pronounced. Except for slightly lower HEI mean scores at age 36 y and 43 y in group 1, all 3 groups generally showed improvements in diet quality over time.

Unadjusted multinomial logit models identified sex, birth region, and childhood social class as significant predictors of diet trajectories (*P* < 0.001 for all), but BMI at age 4 y (*P* = 0.10) was not a significant predictor. Results from the final (step 3) diet trajectory model adjusted for the significant predictors are presented in Table 4. Findings indicated that participants had a greater chance of membership in the moderate and higher diet quality trajectory groups, compared with the lower diet quality group (group 1), if they were female (group 2 OR = 2.25, 95% CI: 1.75, 2.89; group 3 OR = 6.25, 95% CI: 4.66, 8.40), born in Southeast England (group 2 OR = 1.81, 95% CI: 1.38, 2.38; group 3 OR = 1.43, 95% CI: 1.05, 1.94), or from the skilled (nonmanual) social class or higher in childhood (group 2 OR = 2.31, 95% CI: 1.78, 3.00; group 3 OR = 4.54, 95% CI: 3.41, 6.04).

**TABLE 4:** Early-life, time-stable predictors of membership in dietary and cognitive trajectory groups^1^.

Early-life predictors	Group 1	Group 2		Group 3		Group 4	
	Reference	OR (95% CI)	*P*	OR (95% CI)	*P*	OR (95% CI)	*P*
Diet quality model *n =* 3059							
Sex: female	Ref	2.25 (1.75, 2.89)	<0.001	6.25 (4.66, 8.40)	<0.001	—	—
Region of birth: SE England	Ref	1.81 (1.38, 2.38)	<0.001	1.43 (1.05, 1.94)	0.02	—	—
Childhood social class: skilled (nonmanual) and higher	Ref	2.31 (1.78, 3.00)	<0.001	4.54 (3.41, 6.04)	<0.001	—	—
Cognitive ability model^2^*n* = 2431							
Sex: female	Ref	1.41 (1.04, 1.92)	0.03	0.91 (0.69, 1.19)	0.48	1.30 (1.00, 1.70)	0.05
HEI-2020 total score, age 4 y	Ref	1.02 (1.00, 1.04)	0.12	1.02 (1.00, 1.04)	0.03	1.03 (1.02, 1.04)	0.002
Childhood social class: skilled (nonmanual) and higher	Ref	2.82 (1.98, 4.02)	<0.001	3.94 (2.85, 5.45)	<0.001	11.16 (8.23, 15.14)	<0.001
Intellectual activities, age 11 y	Ref	1.81 (1.36, 2.41)	<0.001	1.68 (1.30, 2.18)	<0.001	2.45 (1.91, 3.15)	<0.001
Social activities, age 11 y	Ref	1.55 (1.23, 1.96)	<0.001	1.43 (1.15, 1.77)	0.001	1.79 (1.46, 2.20)	<0.001

Abbreviations: CI, confidence interval; HEI, Healthy Eating Index; OR, odds ratio; ref, reference group; y, year(s).

1 Factors showing significant associations with trajectory group membership in unadjusted multinomial logit models were subsequently added as time-stable covariates in the final group-based trajectory models for diet and cognitive ability.

2 Cohort members missing predictors (*n* = 628) were dropped from the adjusted group-based trajectory model for cognitive ability.

### Global cognitive ability trajectory groups

**FIGURE 3:**
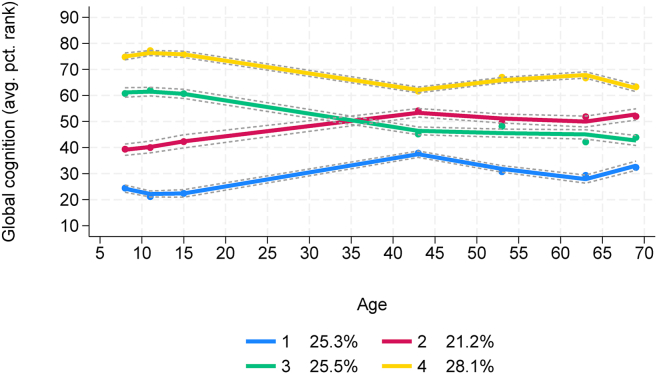
Cognitive trajectory groups from unadjusted group-based trajectory modeling analysis (*n* = 3059) with trends in global cognitive ability scores from age 8 y to 68 to 69 y. Lines = estimated trajectories with 95% CIs, dots = observed group means, legend presents expected group membership percentages. avg. pct., average percentile; CI, confidence interval.

Through group-based trajectory modeling, participants were assigned to 1 of 4 cognitive trajectories based on their trends in global cognitive ability scores, as depicted in Figure 3. Sensitivity analyses showed nearly identical trajectory group classification when ACE-III total scores were included or removed from age 68 to 69 y global cognitive ability scores (agreement = 96.50%, κ = 0.95, SE = 0.01), so ACE-III scores were retained. Because the global cognitive ability scores were based on percentile ranks, each trajectory indicates a group’s trend in cognitive ability relative to their peers. Trends identified participants with consistently lower cognitive ability (group 1), low-to-moderate ability (group 2), high-to-moderate ability (group 3), and consistently higher cognitive ability (group 4). Participants were fairly evenly distributed across these 4 groups with expected membership ranging from 21.2% in group 2 to 28.1% in group 4. For each trajectory, mean percentile rank scores were fairly stable throughout childhood and suggested clear divisions in global cognitive ability. In adulthood, cognitive tests focused more on function than ability, and group means became more similar. Despite this, the trajectory group mean percentile rank scores varied significantly at every data collection period (*P* < 0.0001 for all), and trends were still distinguishable. Mean global cognitive ability scores at each data collection period and for each trajectory group are presented in Table 3 along with results from 1-way ANOVAs (comparisons between groups) and repeated-measures ANOVAs (comparisons within groups over time).

Unadjusted multinomial logit models identified sex, HEI total scores at age 4 y, and childhood social class, and number of intellectual and social activities reported at age 11 y as significant predictors of cognitive trajectory group membership (*P* = 0.01 for sex; *P* < 0.0001 for all others), but birth region (*P* = 0.07) and age 4 y BMI (*P* = 0.50) were not significant predictors. Results from the final (step 3) cognitive trajectory model adjusted for the significant predictors indicated that participants had a greater chance of membership in the all 3 higher cognitive trajectory groups (groups 2–4), compared with the lower cognitive group (group 1), if they were from the skilled (nonmanual) social class or higher in childhood and if they reported engagement in more intellectual activities and more social activities at age 11 (Table 4). Being female predicted a greater chance of being in the low-moderate cognitive trajectory group compared with the lower group (group 2 OR = 1.41, 95% CI: 1.04, 1.92; *P* = 0.03) but did not predict membership in the other 2 groups (group 3 and group 4, *P* ≥ 0.05). Higher HEI-2020 total scores at age 4 predicted a greater chance of being in the high-moderate (group 3 OR = 1.02, 95% CI: 1.00, 1.04; *P* = 0.03) and higher cognitive trajectory groups (group 4 OR = 1.03, 95% CI: 1.02, 1.04; *P* = 0.002), but not the low-moderate group (group 2 OR = 1.02, 95% CI: 1.00, 1.04; *P* = 0.12), compared with the lower cognitive trajectory group.

### Connections between diet quality and global cognitive ability

**FIGURE 4:**
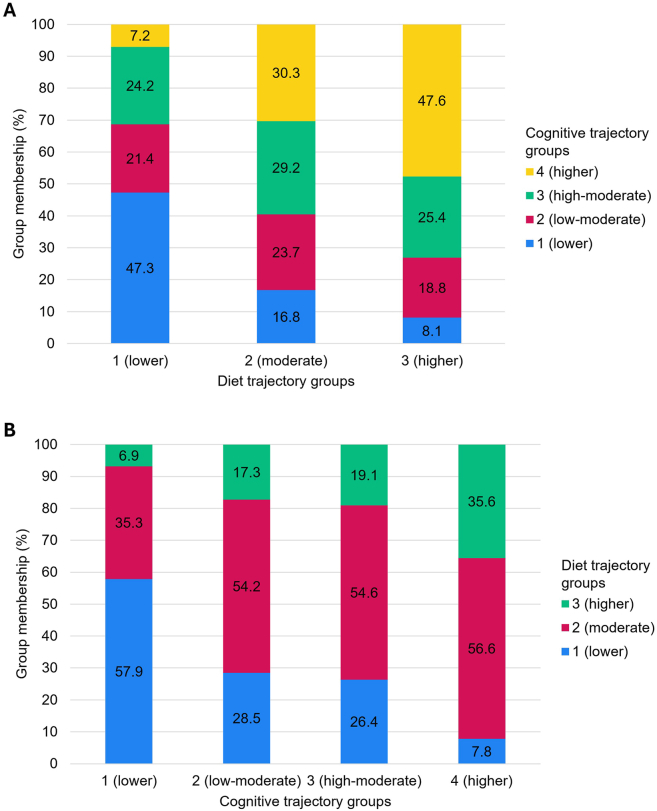
Conditional probabilities of group membership from a joint trajectory analysis simultaneously modeling the diet and cognitive trajectories (*n* = 3059). (A) Distribution of participants from cognitive trajectory groups across diet trajectory groups. (B) Distribution of participants from diet trajectory groups across cognitive trajectory groups.

Results from the joint trajectory model (entropy = 0.78) indicated links between diet quality and cognitive abilities throughout the life course. Supplemental Figure 6 presents line graphs for the joint analysis trajectories which do not differ visually from the original models; however, group membership percentages changed slightly for both the diet and cognitive models, as is typical. Figure 4 shows the conditional probabilities for group membership (i.e. probability of membership in a given diet trajectory group conditional on membership in a given cognitive trajectory group, and vice versa). As seen in the top portion of the figure, the largest proportion of participants (47%) with lower diet quality (group 1) were from the lower cognitive ability trajectory (group 1). Conversely, the largest proportion of participants (48%) with higher diet quality (group 3) were from the higher cognitive ability trajectory (group 4). As seen in the bottom portion of the figure, participants in the lower cognitive trajectory (group 1) were predominantly (58%) from the lower diet trajectory (group 1), and few (7%) were from the higher diet trajectory (group 3). The groups showing low-moderate (group 2) and high-moderate (group 3) cognitive ability were both made up mostly (54%–55%) of participants from the moderate diet trajectory (group 2). Although the group with higher cognitive ability (group 4) was also mostly (57%) from the moderate diet trajectory (group 2), this group comprised the largest proportion of participants (36%) from the higher diet quality trajectory (group 3). Results from a Pearson’s χ^*2*^ test indicated the observed group distributions differed significantly from expected (χ^*2*^ = 725.6, *P <* 0.001), and the magnitude of this difference was large (*df* = 2, *V* = 0.34).

**FIGURE 5:**
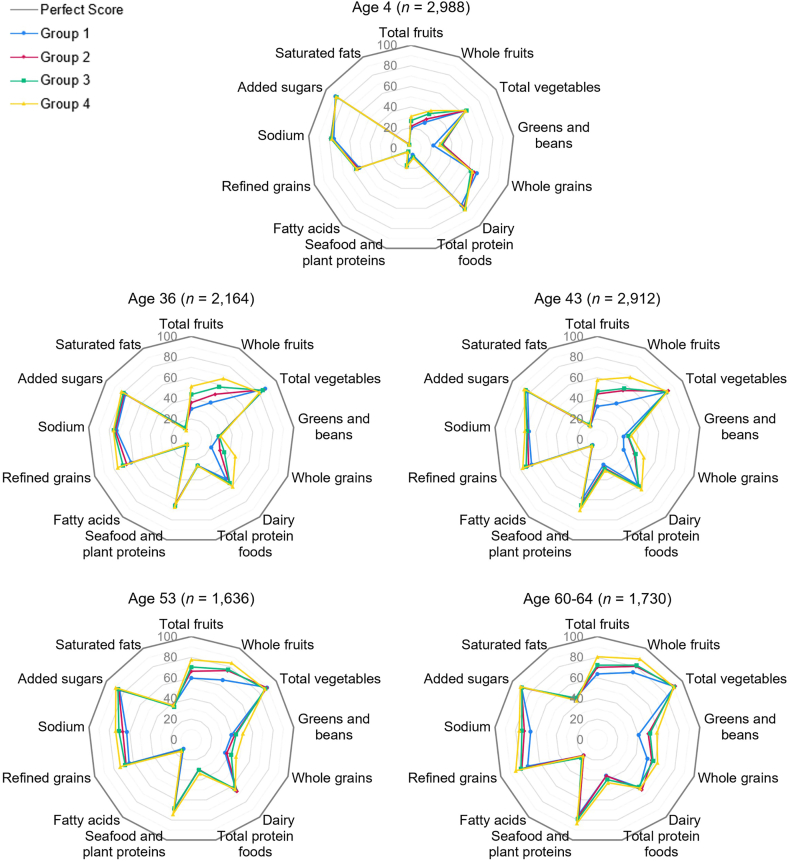
Mean hei-2020 component scores for cognitive trajectory groups 1 to 4 at each data collection period where diet was assessed. Higher scores for moderation items (refined grains, sodium, added sugars, and saturated fats) indicate lower intake. HEI, Healthy Eating Index.

As seen in Table 2, results from 1-way ANOVAs indicated significant variations in mean HEI-2020 total scores across the cognitive trajectory groups such that mean HEI scores increased in stepwise fashion from group 1 to group 4, and this pattern persisted at every time point (*P <* 0.0001 for all). Similarly, Table 3 shows significant variations in global cognitive ability percentile rank scores across the diet trajectory groups. Here, mean cognitive scores increased in stepwise fashion across the diet trajectories at every data collection period (*P <* 0.0001 for all). In Figure 5, radar plots depict HEI component scores for the cognitive trajectory groups at each data collection period. As seen in these plots, intake across components at age 4 was similar for the cognitive trajectory groups, but participants in the high-moderate (group 3) and higher (group 4) cognitive trajectories showed slightly greater mean intakes of total fruits, whole fruits, and greens and beans compared with participants in the lower cognitive trajectory (group 1). Throughout adulthood, participants in the higher cognitive trajectory had greater mean intake of total fruits, whole fruits, and whole grains with lower mean intake of refined grains compared with the other cognitive groups. At ages 53 y and 60 to 64 y, those in the higher cognitive group also had lower sodium intake, higher intake of greens and beans, and slightly higher intake of total protein foods, on average.

### Likely dementia in diet quality trajectory groups

At age 68 to 69 y, ACE-III total scores were available for 1649 cohort members. In this subgroup, 102 participants (6.2%) had ACE-III total scores <82 and were categorized as showing indications of likely dementia. This dementia indicator was added as a subsequent outcome to the unadjusted diet quality trajectory model and to the model adjusted for sex, birth region, and childhood social class. Results from the unadjusted and adjusted models were nearly identical, and the adjusted results are presented here. Findings indicated a greater chance of likely dementia in participants with consistently lower HEI-2020 total scores (OR = 0.12, 95% CI: 0.09, 0.16) compared with those with consistently moderate (OR = 0.05, 95% CI: 0.04, 0.07) or higher scores (OR = 0.02, 95% CI: 0.01, 0.04). Results from a subsequent χ^*2*^ test identified significantly different proportions of participants with likely dementia than what would be expected across the 3 groups (χ^*2*^ = 17.40, *P* < 0.001). In group 1, 9.8% of participants showed signs of likely dementia followed by 6% of group 2 and 2.4% of group 3 participants.

## Discussion

Using longitudinal data from the 1946 British Birth Cohort of the NSHD, this study aimed to investigate the association between diet quality, measured with HEI-2020 scores, and global cognitive ability by uncovering the relationship between these 2 factors throughout the life course. Three diet trajectories were identified, where sex, region of birth, and childhood social class significantly predicted group membership. Four cognitive trajectories were identified, and significant early-life predictors of trajectory group membership included sex, childhood social class, age 4 y HEI scores, and number of social and intellectual activities at age 11 y. In support of our hypothesis, a dual trajectory analysis indicated links between trends in diet quality and global cognitive ability over time and suggested a positive association. Participants with consistently lower global cognitive ability compared with their peers were more likely to show consistently lower diet quality over time, whereas participants with consistently higher cognitive ability were more likely to show moderate or higher diet quality.

To our knowledge, this is the first study to identify trends in diet quality and cognitive ability across the life course and to examine links between these factors using longitudinal data from a single cohort. However, prior research has similarly indicated positive associations between diet quality and cognitive health in later life. In a large systematic review, closer alignment with healthy dietary patterns (e.g. Mediterranean, DASH, and especially the MIND diet) was found to be associated with less cognitive decline and lower risk of Alzheimer’s across different study designs [8]. Most of these studies took place in populations aged ≥60 y. Other reviews have proposed potential mechanisms for the association between healthy dietary patterns and cognitive health. These especially include the contribution of fatty acids, B vitamins (B6, B12, and folate), antioxidants (e.g. vitamins E and C), and plant phytochemicals to the synthesis, function, and preservation of neuronal membranes and nerve synapses, and against neurodegeneration [47,48]. Although several studies have examined associations between later-life diet and cognitive health, future research should investigate the contribution of diet at various life stages to trends in cognitive ability over time and to later-life cognitive outcomes.

In this cohort, mean HEI-2020 total scores for all 3 trajectory groups were similar at age 4 y, in 1950. This was not surprising due to World War II food rationing that continued until mid-1954 in the United Kingdom [49] and due to global trends showing low dietary diversity in childhood [50]. After age 4 y, the trajectories branched off into 3 distinct trends with varying degrees of improvement in HEI scores from age 36 y to 60 to64 y. These findings are consistent with a recent study that used principal components analysis to identify generally improved diet quality trends throughout adulthood in this same cohort [40]. Dietary data were not captured throughout adolescence and early adulthood in the NSHD, so it is not clear how dietary patterns may have changed during that part of the life course. However, the pattern of mean HEI-2020 total scores in the lower diet trajectory reflected patterns identified in the general United States population [21], where diet quality trends follow a slight U-shaped curve with the highest mean scores in early and later stages of life.

The cognitive trajectory model identified generally stable global cognitive ability scores in childhood for all 4 trajectory groups. However, in adulthood, participants in 2 of the groups showed steeper changes in cognitive ability relative to their peers, where mean global cognitive ability scores increased in group 2 and decreased in group 3. The other 2 groups maintained consistently lower (group 1) and higher (group 4) cognitive function relative to their peers. These variations in cognitive ability trajectories may be due to individual differences in 3 related but distinctive concepts: brain reserve (brain structure characteristics), brain maintenance (brain structure changes over time), and cognitive reserve (adaptability of cognitive processes) [41,51]. Of these 3, cognitive reserve is thought to be responsible for “cognitive performance that is better than expected given the degree of life-course related brain changes and brain injury or disease,” according to The Collaboratory on Research Definitions for Reserve and Resilience in Cognitive Aging and Dementia [52]. Although the cohort participants were the same age at each data collection period and were all born around the end of World War II, they were from households with different resources and social classes, they had unique life experiences related to cognitive demands (e.g. years and level of education, occupational complexity, playing musical instruments or games like chess), and they had individual lifestyle habits. Each of these factors can facilitate different levels of neural resource development, including neural pathways and number of neurons, which ultimately define an individual’s cognitive reserve, or ability to adapt when cognitive decline inevitably begins to occur in later years [51]. Our study provides additional evidence to support this since early-life factors, including sex, childhood social class, diet quality, and both social and intellectual leisure activities, were predictive of cognitive trajectory group membership. Among these factors, being from a household in the skilled (nonmanual) social class or higher and having greater involvement in leisure activities in childhood most strongly predicted a participant’s chance of being in any of the higher cognitive trajectories compared with the consistently lower cognitive trajectory. Being female and having higher HEI total scores at age 4 y were less influential but did predict membership in some of the higher cognitive trajectory groups.

Although early-life diet was not a strong predictor of trends in cognitive ability, our exploratory analysis found that diet trajectories spanning age 4 y to 60 to 64 y were associated with subsequent indications of likely dementia at age 68 to 69 y. The percentage of participants with indications of likely dementia was 3.8% to 7.4% greater among those with consistently lower diet quality compared with moderate or higher diet quality, respectively. Additional studies examining the role of diet up to midlife on later-life cognitive health are needed to confirm these findings.

### Strengths and limitations

The primary strength of this study is the use of 7 decades of data from an ongoing birth cohort that included multiple repeated dietary and cognitive assessments throughout life. Truly longitudinal studies such as this are necessary to determine whether and how dietary patterns and global cognitive ability are linked throughout the life course. However, higher attrition is a common limitation in prospective cohorts with such long follow-up periods. In the NSHD, most attrition has occurred from premature mortality and emigration from the United Kingdom, whereas fewer participants have withdrawn from the study or become untraceable over the 75+ y of follow-up [13]. To maximize available data, we used GBTM analysis which requires participants to contribute just 2 periods of data to determine trends. As a result, our analytical sample retained 57% of the original cohort. Despite this, our findings may not be generalizable to the entire cohort due to baseline differences between included and excluded participants.

Another limitation in this study was the lack of racial and ethnic diversity in the British cohort (100% Caucasian presumed), which further limits the generalizability of our results to other populations. However, the original cohort was representative of the population in post-war Britain, a time predating mass immigration to the United Kingdom, and represented all regions of Great Britain and diverse family backgrounds. This diversity was retained in our analytical sample. Furthermore, our use of a dietary index that measures culturally neutral food groups increases the potential relevance of our findings for other populations. Harmonizing across the United Kingdom and United States food systems involved various assumptions and potential errors which we minimized by combining nutrient data (40% of HEI points possible) derived from United Kingdom food composition tables with food group equivalents from United States databases.

Finally, although we followed generally accepted methods to average reported dietary intakes, we must acknowledge the inherent limitations of self-reported dietary data and our inability to estimate usual intakes in the study population given the consecutive (not random) days of dietary records. The single 24-h dietary recall collected at participant age 4 y further limited the assessment of usual intake at that age. Our decision to use all available dietary data also resulted in some participants having more robust dietary estimates than others due to the number of days food diaries were recorded; although most participants had complete diaries.

In conclusions, healthy dietary patterns are associated with a lower risk of cognitive decline and dementia, but current studies focus primarily on older adult populations. In this longitudinal analysis of participants from the 1946 British Birth Cohort, consistently closer alignment with dietary guidelines from childhood to adulthood was associated with better global cognitive ability trends over time compared with dietary patterns less aligned with guidelines. Despite similar diet quality at age 4 y, higher HEI-2020 total scores at this age were predictive of being in higher cognitive ability trajectories. Thus, early-life diet quality may be an important modifiable risk factor for cognitive ability throughout the life course. Future prospective studies should include assessments of dietary intake at earlier life stages (before age 60 y) in various races, ethnicities, and cultures to investigate potential causal associations between diet and cognitive outcomes.

## Author contributions

The authors’ responsibilities were as follows – KCC: had primary responsibility for final content, designed and conducted the research, analyzed the data, and wrote the paper; PFJ, TMS, MC: advised KCC throughout the entire research process; and all authors: read and approved the final manuscript.

## Data availability

Data described in the manuscript, code book, and analytic code will be made available to bona fide researchers upon request to the NSHD Data Sharing Committee via a standard application procedure. Further details can be found at http://www.nshd.mrc.ac.uk/data.

## Funding

This study received no external funding. KCC was supported by a PhD stipend from the Gerald J. and Dorothy R. Friedman School of Nutrition Science and Policy at Tufts University. Tufts had no role in the study design; the collection, analysis, and interpretation of data; the writing of the manuscript; or the decision where to submit the paper for publication. Effort for PFJ on this study was supported by USDA Agricultural Research Service Cooperative Agreement number 58-8050-9-004.

## Conflict of interest

PFF reports financial support was provided by USDA Agricultural Research Service. If there are other authors, they declare that they have no known competing financial interests or personal relationships that could have appeared to influence the work reported in this paper.
