# Supplementary material for: Associations between Diet Quality and Global Cognitive Ability across the Life Course: Longitudinal Analysis of the 1946 British Birth Cohort
Source: Curr Dev Nutr. 2025 Dec 20;10(2):107619. doi: 10.1016/j.cdnut.2025.107619 (PMC12860707; doi:10.1016/j.cdnut.2025.107619)
Supplement: multimedia component 1 [file mmc1.pdf]

Supplemental file: Diet quality and cognitive ability, Cara et al.

Crosswalk linking food codes from the UK National Survey of Health and Development with the USDA Food Patterns Equivalents/Ingredients Databases

	National Survey of Health and Development (NSHD) foods & beverages, 1950-2009, based on UK food tables						USDA Food Patterns Equivalents/Ingredients Databases (FPED/FPID 2015-2016)	
SEQ	Food code 1	Food code 2	Food code 3	Food description	Food group code	Food group description	FPED/ FPID code	Item description *NFS = not further specified* *NS = not specified*
1	02-10556			0.75% MILK PASTUERISED	05.08.00	Milk - 1% milk	11100000	Milk, NFS
2	02-09991			7 UP CANNED NOT LOW CAL	27.03.00	Beverages - Carbonated soft drinks	92400000	Soft drink, NFS
3	02-09992			7 UP LIGHT LOW CAL CANNED	27.03.00	Beverages - Carbonated soft drinks	92400000	Soft drink, NFS
4	02-09994			7-UP LIGHT LOW CALORIE BOTTLED	27.03.00	Beverages - Carbonated soft drinks	92400000	Soft drink, NFS
5	02-09993			7-UP NOT CANNED NOT LOW CALORIE	27.03.00	Beverages - Carbonated soft drinks	92400000	Soft drink, NFS
6	00-03613			95%fat free. Macaroni cheese	01.02.00	Cereals & cereal dishes - Pasta & pasta dishes	58145110	Macaroni or noodles with cheese
7	02-02257			AFTER EIGHT MINTS	24.01.00	Confectionary - Chocolate based products	91705300	Chocolate, sweet or dark
8	02-05142			ALCOHOLIC SOFT DRINKS FRUIT FLAVOURED NOT SPIRIT BASED	27.01.05	Beverages - Alcohol - Alcopops	93106000	Alcoholic malt beverage, sweetened
9	02-10426			ALDI HARVEST MORN FRUIT AND GRAIN BAR	04.05.00	Sweet cereal products - Cereal bars	53710500	Cereal or granola bar (Kellogg's Nutri-Grain Cereal Bar)
10	02-10418			ALDI MULTIVITAMIN FRUIT JUICE DRINK NAS	27.02.02	Beverages - Fruit based drinks - Fruit juice drinks	92531030	Fruit juice drink (Sunny D)
11	02-08191			ALL BUTTER BISCUITS	04.01.00	Sweet cereal products - Biscuits	53239000	Cookie, shortbread
12	02-05151			AME SPARKLING DRINK WITH FRUIT JUICE & HERBS	27.03.00	Beverages - Carbonated soft drinks	92433000	Fruit juice drink, noncitrus, carbonated
13	02-10074			AMERICAN MUFFINS LOW FAT, PURCHASED, ANY FLAVOUR	04.02.00	Sweet cereal products - Pastries, Buns & Pies	52301000	Muffin, NFS

Diet quality and cognitive ability, Cara et al.

Crosswalk linking food codes from the UK National Survey of Health and Development with the USDA Food Patterns Equivalents/Ingredients Databases

14	02-10285			APPLE AND BLACKCURRANT FRUIT JUICE, NOT FROM CONCENTRATE	27.02.01	Beverages - Fruit based drinks - Pure fruit juice & smoothies	61210000	Orange juice, 100%, NFS
15	02-02320			APPLE JUICE DRINK CARBONATED NOT LOW CAL NOT CANNED	27.03.00	Beverages - Carbonated soft drinks	92433000	Fruit juice drink, noncitrus, carbonated
16	02-08691			APPLE JUICE DRINK RTD NOT LOW CALORIE	27.02.02	Beverages - Fruit based drinks - Fruit juice drinks	92531030	Fruit juice drink (Sunny D)
17	02-02319			APPLE JUICE UNSWEETENED UHT	27.02.02	Beverages - Fruit based drinks - Fruit juice drinks	92531030	Fruit juice drink (Sunny D)
18	02-02160			APPLE SAUCE CANNED	18.02.00	Fruit - Canned & cooked	63101110	Applesauce, stewed apples, NS as to sweetened or unsweetened; sweetened, NS as to type of sweetener
19	02-09543			ARTIFICIAL SWEETENER GRANULATED 1/2 TSP	26.03.00	Miscellaneous - Artificial sweeteners	91200000	Sugar substitute, powder, NFS
20	02-09612			ARTIFICIAL SWEETENER GRANULATED PER 100G (RECIPES)	26.03.00	Miscellaneous - Artificial sweeteners	91200000	Sugar substitute, powder, NFS
21	02-10708			ASDA VITALITY CEREAL BAR	04.05.00	Sweet cereal products - Cereal bars	53712100	Cereal or Granola bar, NFS
22	02-08471			AUBERGINE BOILED IN UNSALTED WATER	15.04.00	Vegetables - Other	75217000	Eggplant, cooked, NS as to fat added in cooking
23	00-03366			Actimel 0% (fat free) Danone	06.03.02	Dairy products - Yoghurt & drinking yoghurts, incl. buttermilk and probiotics - reduced or low fat products	11400000	Yogurt, NFS
24	00-03387			Actimel L.Casei imunitass, Danone	06.03.02	Dairy products - Yoghurt & drinking yoghurts, incl. buttermilk and probiotics - reduced or low fat products	11400000	Yogurt, NFS

Diet quality and cognitive ability, Cara et al.
Crosswalk linking food codes from the UK National Survey of Health and Development with the USDA Food Patterns Equivalents/Ingredients Databases

25	13-0042	A-13-0042		Aduki beans, dried, boiled in unsalted water	16.01.00	Pulses/Lentils - Pulses/lentils	41101000	Beans, dry, cooked, NS as to type and as to fat added in cooking
26	A-00-03003	00-03003		Advantage, Weetabix	02.02.00	Breakfast cereals - Other breakfast cereals - high fibre (equal or >3g/40g portion)	57100100	Cereal, ready-to-eat, NFS
27	17-0241	A-00-00916	A-17-0241	Advocaat	27.01.04	Beverages - Alcohol - Spirits & Liqueur	93501000	Brandy
28	00-09579	A-00-09579		Aerated slimmers white bread	03.01.00	Breads - White	51122000	Bread, reduced calorie and/or high fiber, white or NFS
29	00-03875			Alcopops, clear or lemonade based	27.01.05	Beverages - Alcohol - Alcopops	93106000	Alcoholic malt beverage, sweetened
30	00-05733			Aldi Harvest Morn Fruit and Fibre, Fortified ONLY	02.02.00	Breakfast cereals - Other breakfast cereals - high fibre (equal or >3g/40g portion)	57100100	Cereal, ready-to-eat, NFS
31	00-05823			Aldi Premium Cereal Bar containing fruit, fortified	04.05.00	Sweet cereal products - Cereal bars	53710500	Cereal or granola bar (Kellogg's Nutri-Grain Cereal Bar)
32	13-0148	A-13-0148		Alfalfa sprouts, raw	15.04.00	Vegetables - Other	75100500	Alfalfa sprouts, raw
33	00-03339			All in one medium sliced bread WARBURTONS (RISCK-LA)	03.03.00	Breads - Brown/Granary/Wheatgerm	51301010	Bread, wheat or cracked wheat
34	11-0126	A-11-0126		All-Bran	02.02.00	Breakfast cereals - Other breakfast cereals - high fibre (equal or >3g/40g portion)	57101000	Cereal (Kellogg's All-Bran)
35	A-11-0189			All-Bran loaf	03.04.00	Breads - Other bread	51000400	Roll, bran, NS as to type of bran
36	A-00-00047			All-bran	02.02.00	Breakfast cereals - Other breakfast cereals - high fibre (equal or >3g/40g portion)	57101000	Cereal (Kellogg's All-Bran)
37	A-00-01010			All-bran Loaf	03.04.00	Breads - Other bread	51000400	Roll, bran, NS as to type of bran

38	A-00-03820			Allbran Loaf, no sugar 82/1915	03.04.00	Breads - Other bread	51000400	Roll, bran, NS as to type of bran
39	00-03004	A-00-03004		Allbran, Kellogs	02.02.00	Breakfast cereals - Other breakfast cereals - high fibre (equal or >3g/40g portion)	57101000	Cereal (Kellogg's All-Bran)
40	00-05169			Allspice Powder	26.01.00	Miscellaneous - Dried herbs & spices & pastes	2011	Allspice, ground
41	A-14-0801	14-0801	A-00-00822	Almonds	19.00.00	Nuts & Seeds (incl. peanut butter)	42100100	Almonds, NFS
42	A-00-00823			Almonds (Weighed with Shells)	19.00.00	Nuts & Seeds (incl. peanut butter)	42100100	Almonds, NFS
43	14-0803	A-14-0803		Almonds, toasted	19.00.00	Nuts & Seeds (incl. peanut butter)	42100100	Almonds, NFS
44	00-05680			Alpen Light Cereal Bar	04.05.00	Sweet cereal products - Cereal bars	53712200	Cereal or granola bar, lowfat, NFS
45	00-03155			Alpen cereal bars with yogurt	04.05.00	Sweet cereal products - Cereal bars	53710902	Cereal or granola bar, with yogurt coating (General Mills Nature Valley Chewy Granola Bar)
46	00-05350			Alpro Soya Dessert eg vanilla, chocolate	05.04.00	Milk - Other - plant based, e.g. rice, soy	11321000	Soy milk, chocolate
47	00-05423			Alpro Soya yogurt	06.03.01	Dairy products - Yoghurt & drinking yoghurts, incl. buttermilk and probiotics - full fat products	41420380	Yogurt, soy
48	A-00-05057			Anchovies, Canned in Oil, Fish only	09.02.00	Fish & fish dishes - Oily fish	26101180	Anchovy, canned
49	16-0168	A-16-0168		Anchovies, canned in oil, drained	09.02.00	Fish & fish dishes - Oily fish	26101180	Anchovy, canned
50	A-00-01062			Angel Delight	04.04.00	Sweet cereal products - Milk based puddings	13220110	Pudding, flavors other than chocolate, prepared from dry mix, milk added

Diet quality and cognitive ability, Cara et al.

Crosswalk linking food codes from the UK National Survey of Health and Development with the USDA Food Patterns Equivalents/Ingredients Databases

51	A-00-03858			Any other Cola (Own Brand)	27.03.00	Beverages - Carbonated soft drinks	92400000	Soft drink, NFS
52	A-00-00097			Apple Crumble	04.03.00	Sweet cereal products - Cereal based puddings (not milk)	53415100	Crisp, apple, apple dessert
53	A-00-01146			Apple Juice, Canned, Sweetened	27.02.00	Beverages - Fruit based drinks	92550350	Orange juice beverage, 40-50% juice, light
54	A-00-01147			Apple Juice, Canned, Unsweetened	27.02.00	Beverages - Fruit based drinks	61210220	Orange juice, 100%, canned, bottled or in a carton
55	A-00-03134	00-03134		Apple Turnover	04.02.00	Sweet cereal products - Pastries, Buns & Pies	53450000	Turnover or dumpling, apple
56	A-14-0272			Apple juice concentrate, unsweetened	27.02.03	Beverages - Fruit based drinks - Squashes & fruit concentrates	9214	Orange juice, frozen concentrate, unsweetened, undiluted
57	14-0272			Apple juice concentrate, unsweetened, needs diluting	27.02.03	Beverages - Fruit based drinks - Squashes & fruit concentrates	9215	Orange juice, frozen concentrate, unsweetened, diluted with 3 volume water
58	14-0271	A-14-0271		Apple juice, unsweetened	27.02.01	Beverages - Fruit based drinks - Pure fruit juice & smoothies	61210000	Orange juice, 100%, NFS
59	11-0278	A-11-0278		Apple pie, one crust	04.02.00	Sweet cereal products - Pastries, Buns & Pies	53301500	Pie, apple, one crust
60	A-00-06350			Apple pie, one crust 50	04.02.00	Sweet cereal products - Pastries, Buns & Pies	53301500	Pie, apple, one crust
61	A-11-0279			Apple pie, pastry top and bottom	04.02.00	Sweet cereal products - Pastries, Buns & Pies	53301000	Pie, apple, two crust
62	A-00-06351			Apple pie, pastry top and bottom 50	04.02.00	Sweet cereal products - Pastries, Buns & Pies	53301000	Pie, apple, two crust
63	A-00-06084			Apple pie, pastry, top and bottom 50	04.02.00	Sweet cereal products - Pastries, Buns & Pies	53301000	Pie, apple, two crust
64	00-09618	A-00-09618		Apple pie, two crusts	04.02.00	Sweet cereal products - Pastries, Buns & Pies	53301000	Pie, apple, two crust
65	A-11-0280			Apple pie, wholemeal, one crust	04.02.00	Sweet cereal products - Pastries, Buns & Pies	53301500	Pie, apple, one crust

66	A-00-06083			Apple pie, wholemeal, one crust 50	04.02.00	Sweet cereal products - Pastries, Buns & Pies	53301500	Pie, apple, one crust
67	A-11-0281	11-0281		Apple pie, wholemeal, pastry top and bottom	04.02.00	Sweet cereal products - Pastries, Buns & Pies	53301000	Pie, apple, two crust
68	A-17-0288	17-0288		Apple sauce, homemade	18.02.00	Fruit - Canned & cooked	63101110	Applesauce, stewed apples, NS as to sweetened or unsweetened; sweetened, NS as to type of sweetener
69	00-05743			Apple, eating, pureed (stewed), no sugar	18.02.00	Fruit - Canned & cooked	63101110	Applesauce, stewed apples, NS as to sweetened or unsweetened; sweetened, NS as to type of sweetener
70	A-00-00679			Apples, Baked (Weighed with Skin)	18.02.00	Fruit - Canned & cooked	63101310	Apple, baked, NS as to added sweetener
71	A-00-01121			Apples, Baked with Sugar	18.02.00	Fruit - Canned & cooked	63101330	Apple, baked, with sugar
72	A-00-00678			Apples, Baked without Sugar	18.02.00	Fruit - Canned & cooked	63101320	Apple, baked, unsweetened
73	A-00-00675			Apples, Eating	18.01.00	Fruit - Fresh	63101000	Apple, raw
74	A-00-00676			Apples, Eating (Weighed with Skin and Core)	18.01.00	Fruit - Fresh	63101000	Apple, raw
75	A-00-01164			Apples, Raw, with Peel	18.01.00	Fruit - Fresh	63101000	Apple, raw
76	A-00-00681			Apples, Stewed with Sugar	18.02.00	Fruit - Canned & cooked	63101110	Applesauce, stewed apples, NS as to sweetened or unsweetened; sweetened, NS as to type of sweetener

77	A-00-00680			Apples, Stewed without Sugar	18.02.00	Fruit - Canned & cooked	63101110	Applesauce, stewed apples, NS as to sweetened or unsweetened; sweetened, NS as to type of sweetener
78	A-14-0006	14-0006		Apples, cooking, baked with sugar, flesh and skin	18.02.00	Fruit - Canned & cooked	63101330	Apple, baked, with sugar
79	A-14-0009	14-0009		Apples, cooking, baked without sugar, flesh and skin	18.02.00	Fruit - Canned & cooked	63101320	Apple, baked, unsweetened
80	14-0004	A-14-0004		Apples, cooking, stewed with sugar	18.02.00	Fruit - Canned & cooked	63101110	Applesauce, stewed apples, NS as to sweetened or unsweetened; sweetened, NS as to type of sweetener
81	14-0005	A-14-0005		Apples, cooking, stewed without sugar	18.02.00	Fruit - Canned & cooked	63101110	Applesauce, stewed apples, NS as to sweetened or unsweetened; sweetened, NS as to type of sweetener
82	14-0017	A-14-0017		Apples, eating, Cox's Pippin, raw	18.01.00	Fruit - Fresh	63101000	Apple, raw
83	A-14-0019	14-0019		Apples, eating, Golden Delicious, raw	18.01.00	Fruit - Fresh	63101000	Apple, raw
84	14-0021	A-14-0021		Apples, eating, Granny Smith, raw	18.01.00	Fruit - Fresh	63101000	Apple, raw
85	14-0012	A-14-0012		Apples, eating, average, raw	18.01.00	Fruit - Fresh	63101000	Apple, raw
86	14-0014	A-14-0014		Apples, eating, average, raw, peeled	18.01.00	Fruit - Fresh	63101000	Apple, raw
87	A-14-0016	14-0016		Apples, eating, dried	18.03.00	Fruit - Dried	62101100	Apple, dried, uncooked

88	14-0023	A-14-0023		Apples, eating, red dessert, raw	18.01.00	Fruit - Fresh	63101000	Apple, raw
89	A-00-03822			Apricot Oaties U/R 82/1923	04.01.00	Sweet cereal products - Biscuits	53220000	Cookie, fruit-filled bar
90	A-00-00691			Apricots, Canned	18.02.00	Fruit - Canned & cooked	63103110	Apricot, cooked or canned, NS as to sweetened or unsweetened; sweetened, NS as to type of sweetener
91	A-00-00688			Apricots, Dried, Raw	18.03.00	Fruit - Dried	62104100	Apricot, dried, uncooked
92	A-00-00690			Apricots, Dried, Stewed with Sugar	18.03.00	Fruit - Dried	62104230	Apricot, dried, cooked, with sugar
93	A-00-00689			Apricots, Dried, Stewed without Sugar	18.03.00	Fruit - Dried	62104220	Apricot, dried, cooked, unsweetened
94	A-00-00682			Apricots, Fresh, Raw	18.01.00	Fruit - Fresh	63103010	Apricot, raw
95	A-00-00683			Apricots, Fresh, Raw (Weighed with Stones)	18.01.00	Fruit - Fresh	63103010	Apricot, raw
96	A-00-00686			Apricots, Fresh, Stewed with Sugar	18.02.00	Fruit - Canned & cooked	63103110	Apricot, cooked or canned, NS as to sweetened or unsweetened; sweetened, NS as to type of sweetener
97	A-00-00687			Apricots, Fresh, Stewed with Sugar (Weighed with Stones)	18.02.00	Fruit - Canned & cooked	63103110	Apricot, cooked or canned, NS as to sweetened or unsweetened; sweetened, NS as to type of sweetener

98	A-00-00684			Apricots, Fresh, Stewed without Sugar	18.02.00	Fruit - Canned & cooked	63103110	Apricot, cooked or canned, NS as to sweetened or unsweetened; sweetened, NS as to type of sweetener
99	14-0035			Apricots, canned in juice	18.02.00	Fruit - Canned & cooked	63103170	Apricot, cooked or canned, juice pack
100	A-00-09922			Apricots, canned in juice (MW6 carq)	18.02.00	Fruit - Canned & cooked	63103170	Apricot, cooked or canned, juice pack
101	14-0034			Apricots, canned in syrup	18.02.00	Fruit - Canned & cooked	63103110	Apricot, cooked or canned, NS as to sweetened or unsweetened; sweetened, NS as to type of sweetener
102	A-00-09921			Apricots, canned in syrup (MW6 carq)	18.02.00	Fruit - Canned & cooked	63103110	Apricot, cooked or canned, NS as to sweetened or unsweetened; sweetened, NS as to type of sweetener
103	14-0031	A-14-0031		Apricots, dried	18.03.00	Fruit - Dried	62104100	Apricot, dried, uncooked
104	14-0032	A-14-0032		Apricots, dried, stewed with sugar	18.03.00	Fruit - Dried	62104230	Apricot, dried, cooked, with sugar
105	14-0033			Apricots, dried, stewed without sugar	18.03.00	Fruit - Dried	62104220	Apricot, dried, cooked, unsweetened
106	14-0025			Apricots, raw	18.01.00	Fruit - Fresh	63103010	Apricot, raw
107	A-00-09923			Apricots, raw (MW6 carq)	18.01.00	Fruit - Fresh	63103010	Apricot, raw
108	A-14-0036	14-0036		Apricots, ready-to-eat	18.03.00	Fruit - Dried	62104100	Apricot, dried, uncooked

109	14-0027	A-14-0027		Apricots, stewed with sugar	18.02.00	Fruit - Canned & cooked	63103110	Apricot, cooked or canned, NS as to sweetened or unsweetened; sweetened, NS as to type of sweetener
110	A-14-0029	14-0029		Apricots, stewed without sugar	18.02.00	Fruit - Canned & cooked	63103110	Apricot, cooked or canned, NS as to sweetened or unsweetened; sweetened, NS as to type of sweetener
111	A-00-03751			Aprotein flour 82/1376	01.04.00	Cereals & cereal dishes - Other cereals & dishes	20080	Wheat flour, whole-grain
112	A-12-0198	12-0198		Arctic roll	06.04.02	Dairy products - Ice cream & dairy desserts - reduced fat products	13120300	Ice cream bar, cake covered
113	A-00-03761			Arctic roll 82/1400	06.04.01	Dairy products - Ice cream & dairy desserts - full fat products	13120300	Ice cream bar, cake covered
114	00-09530	A-00-09530		Aromatic crispy duck	11.02.00	Meat - white - Other game birds, (e.g. duck, goose, pheasant) & dishes	24301210	Duck, coated, fried
115	13-0156	A-13-0156		Artichoke, Jerusalem, boiled in unsalted water	15.04.00	Vegetables - Other	75201000	Artichoke, cooked, NS as to form, NS as to fat added in cooking
116	A-13-0155	13-0155		Artichoke, globe, boiled in unsalted water	15.04.00	Vegetables - Other	75201000	Artichoke, cooked, NS as to form, NS as to fat added in cooking
117	A-00-00555			Artichokes, Globe, Boiled	15.04.00	Vegetables - Other	75201000	Artichoke, cooked, NS as to form, NS as to fat added in cooking
118	A-00-00556			Artichokes, Globe, Boiled (Weighed as Served)	15.04.00	Vegetables - Other	75201000	Artichoke, cooked, NS as to form, NS as to fat added in cooking

119	A-00-00557			Artichokes, Jerusalem, Boiled	15.04.00	Vegetables - Other	75201000	Artichoke, cooked, NS as to form, NS as to fat added in cooking
120	00-05745			Asda Fruit and Grain bar, all Flavours, Fortified	04.05.00	Sweet cereal products - Cereal bars	53710500	Cereal or granola bar (Kellogg's Nutri-Grain Cereal Bar)
121	00-05588			Asda Hot & Spicy Chicken	11.01.00	Meat - white - Chicken & turkey & dishes	24168020	Chicken "wings" with hot sauce, from other sources
122	00-05818			Asda Mini Classics Fish Pie	09.01.00	Fish & fish dishes - White fish, incl. tuna	28350050	Fish chowder
123	A-00-03560	00-03560		Asda mixed leaf salad	15.03.00	Vegetables - Yellow & red & dark green leafy vegetables	75114000	Mixed salad greens, raw
124	A-00-00558			Asparagus, Boiled	15.04.00	Vegetables - Other	75202000	Asparagus, cooked, NS as to form, NS as to fat added in cooking
125	A-00-00559			Asparagus, Boiled (Weighed as Served)	15.04.00	Vegetables - Other	75202000	Asparagus, cooked, NS as to form, NS as to fat added in cooking
126	A-13-0158	13-0158		Asparagus, boiled, weighed as served	15.04.00	Vegetables - Other	75202000	Asparagus, cooked, NS as to form, NS as to fat added in cooking
127	A-13-0160	13-0160		Asparagus, canned, re-heated, drained	15.04.00	Vegetables - Other	75202000	Asparagus, cooked, NS as to form, NS as to fat added in cooking
128	13-0157	A-13-0157		Asparagus, raw	15.04.00	Vegetables - Other	75100800	Asparagus, raw
129	A-00-00560			Aubergine, Raw	15.04.00	Vegetables - Other	75111200	Eggplant, raw
130	13-0162	A-13-0162		Aubergine, fried in corn oil	15.04.00	Vegetables - Other	75217000	Eggplant, cooked, NS as to fat added in cooking
131	13-0161			Aubergine, raw	15.04.00	Vegetables - Other	75111200	Eggplant, raw
132	A-00-00692			Avocado Pears	18.01.00	Fruit - Fresh	63105010	Avocado, raw
133	A-14-0037	14-0037		Avocado, average	18.01.00	Fruit - Fresh	63105010	Avocado, raw

134	02-01315			BACON AND EGG IN A MUFFIN, BAGEL OR ROLL TAKEAWAY	12.01.00	Processed meat - Bacon & ham	27520140	Bacon and egg sandwich
135	02-01296			BACON AND EGG PIE	10.03.00	Meat - red - Pork & dishes	58125110	Quiche with meat, poultry or fish
136	02-08232			BACON COLLAR SMOKED BOILED L&F	12.01.00	Processed meat - Bacon & ham	22600100	Bacon, NS as to type of meat, cooked
137	02-00904			BACON GAMMON JOINT BOILED LEAN ONLY	12.01.00	Processed meat - Bacon & ham	22600100	Bacon, NS as to type of meat, cooked
138	02-00907			BACON GAMMON RASHERS GRILLED LEAN ONLY	12.01.00	Processed meat - Bacon & ham	22600100	Bacon, NS as to type of meat, cooked
139	02-08234			BACON JOINT SMOKED BOILED LEAN ONLY	12.01.00	Processed meat - Bacon & ham	22600100	Bacon, NS as to type of meat, cooked
140	02-07839			BAKED BEANS LOW FAT SAUSAGE	16.02.00	Pulses/Lentils - Baked beans	41201010	Baked beans, NFS
141	02-10003			BAKED POTATO CRISPS IN SUNFLOWER OIL EG WALKERS	25.01.00	Savoury Snacks - Potato based snacks	71200400	Potato chips, baked, plain
142	02-02502			BAKING POWDER	26.01.00	Miscellaneous - Dried herbs & spices & pastes	18369	Leavening agents, baking powder, double-acting, sodium aluminum sulfate
143	02-08957			BAMBU CHICORY DRINK - COFFEE SUBSTITUTE	27.06.00	Beverages - Coffee	92100000	Coffee, NS as to type
144	02-04032			BANOFFEE PIE, MADE WITH ARTIFICIAL SWEETNER, WEIGHT WATCHERS ONLY	04.03.00	Sweet cereal products - Cereal based puddings (not milk)	53341000	Pie, banana cream
145	02-08491			BARLEY WATER ANY CONC NOT LOW CALORIE	27.02.03	Beverages - Fruit based drinks - Squashes & fruit concentrates	91301050	Fruit syrup
146	02-09228			BASIL (DRIED)	26.01.00	Miscellaneous - Dried herbs & spices & pastes	2003	Spices, basil, dried
147	02-09284			BEAN SALAD RETAIL	16.01.00	Pulses/Lentils - Pulses/lentils	41203030	Black bean salad

148	02-04731			BEANSPROUTS-COOKED	15.04.00	Vegetables - Other	75207000	Bean sprouts, cooked, NS as to form, NS as to fat added in cooking
149	02-01294			BEEF & POTATO PIE 2 CRUSTS	10.01.00	Meat - red - Beef & veal & dishes	27317010	Beef pot pie
150	02-06790			BEEF AND POTATO SOUP WITH ONIONS AND CARROTS	20.01.00	Soups - Canned & fresh & homemade	28315050	Beef vegetable soup with potato, pasta, or rice, chunky style, canned, or ready-to-serve
151	02-00936			BEEF BRISKET POT-ROASTED OR BRAISED LEAN AND FAT	10.01.00	Meat - red - Beef & veal & dishes	21407110	Beef, pot roast, braised or boiled, lean and fat eaten
152	02-03011			BEEF CHILLI WITH POTATOES, READY MEAL	10.01.00	Meat - red - Beef & veal & dishes	27211550	Stewed, seasoned, ground beef with potatoes, Mexican style
153	02-08107			BEEF CURRY TAKEAWAY	10.01.00	Meat - red - Beef & veal & dishes	27116100	Beef curry
154	02-08010			BEEF CURRY, TINNED ONLY	10.01.00	Meat - red - Beef & veal & dishes	27116100	Beef curry
155	02-00946			BEEF MINCE SKIM GRAVY & VEG	10.01.00	Meat - red - Beef & veal & dishes	27311600	Beef, potatoes, and vegetables including carrots, broccoli, and/or dark-green leafy; gravy
156	02-00945			BEEF MINCED THICK GRAVY & VEG	10.01.00	Meat - red - Beef & veal & dishes	27311600	Beef, potatoes, and vegetables including carrots, broccoli, and/or dark-green leafy; gravy
157	02-06236			BEEF RISSOLES	10.01.00	Meat - red - Beef & veal & dishes	27260050	Meatballs, with breading, NS as to type of meat, with gravy
158	02-03369			BEEF WELLINGTON	10.01.00	Meat - red - Beef & veal & dishes	27214300	Beef wellington

159	02-03848			BENECOL BUTTERY TASTE SPREAD ONLY	08.04.01	Fats - Plant based fats (solid) - Full fat	81102000	Margarine, NFS
160	02-02742			BENECOL CREAM CHEESE SPREAD	06.02.00	Dairy products - Cheese, incl. cottage cheese	14420200	Cheese spread, cream cheese, regular
161	02-03364			BENECOL OLIVE OIL SPREAD	08.04.02	Fats - Plant based fats (solid) - Reduced fat	81102000	Margarine, NFS
162	02-10152			BENECOL YOGURT DRINKS	06.03.02	Dairy products - Yoghurt & drinking yoghurts, incl. buttermilk and probiotics - reduced or low fat products	11436000	Yogurt, liquid
163	02-08145			BIRDS WHISK AND SERVE CUSTARD MADE WITH WATER	04.04.00	Sweet cereal products - Milk based puddings	12220200	Whipped topping
164	02-00272			BISCUITS WAFER SANDWICH CREAM FILLED	04.01.00	Sweet cereal products - Biscuits	53242000	Cookie, sugar wafer
165	02-08541			BISCUITS WITH CREAM AND JAM	04.01.00	Sweet cereal products - Biscuits	53237010	Cookie, raisin sandwich, cream-filled
166	02-10161			BISTO SAUCE GRANULES	21.02.00	Sauces & accompaniment - Cooking sauces, incl. gravies, pesto, cooking sauces for pasta and rice dishes	6011	Soup, cheese, canned, condensed
167	02-08455			BLACKCURRANT JUICE DRINK RTD NOT LOW CALORIE	27.02.02	Beverages - Fruit based drinks - Fruit juice drinks	92531030	Fruit juice drink (Sunny D)
168	02-10209			BLUEBERRY, APPLE AND GRAPE FRUIT JUICE 100% JUICE	27.02.01	Beverages - Fruit based drinks - Pure fruit juice & smoothies	61210000	Orange juice, 100%, NFS
169	02-07780			BOLOGNESE SAUCE CANNED	10.01.00	Meat - red - Beef & veal & dishes	27162040	Spaghetti sauce with meat
170	02-05314			BOLOGNESE SAUCE MADE WITH BOTTLED PASTA SAUCE	10.01.00	Meat - red - Beef & veal & dishes	27162040	Spaghetti sauce with meat

171	02-08311			BOURNVITA LOW FAT DRY WEIGHT	27.05.00	Beverages - Powdered Beverages (cocoa, Horlicks, Bonvita, Ovaltine, etc)	11830260	Milk, malted, dry mix, not reconstituted
172	02-05131			BRAISING STEAK IN GRAVY NO VEG	10.01.00	Meat - red - Beef & veal & dishes	27112000	Beef with gravy
173	02-03245			BREAD & BUTTER PUDDING WITH CREAM	04.04.00	Sweet cereal products - Milk based puddings	13210150	Bread pudding made with evaporated milk and rum
174	02-07618			BREAD OATMEAL TOASTED	03.04.00	Breads - Other bread	51501020	Bread, oatmeal, toasted
175	02-07614			BREAD WHOLEMEAL SLIMMERS EG NIMBLE, WEIGHT WATCHERS DANISH BROWN	03.02.00	Breads - Wholemeal	51301510	Bread, wheat or cracked wheat, reduced calorie and/or high fiber
176	02-08635			BREADED ONION RINGS FRIED IN BLENDED VEG OIL	15.04.00	Vegetables - Other	75415020	Onion rings, NS as to form, batter-dipped, baked or fried
177	02-01291			BRIDIES SCOTCH PIES	12.02.00	Processed meat - Processed pies	27360050	Meat pie, NFS
178	02-07028			BULGAR WHEAT COOKED	01.04.00	Cereals & cereal dishes - Other cereals & dishes	20013	Bulgur, cooked
179	A-00-06201			Bacon 50 streaky fried	12.01.00	Processed meat - Bacon & ham	22600100	Bacon, NS as to type of meat, cooked
180	A-00-03567	00-03567		Bacon Collar, Lean only, boiled	12.01.00	Processed meat - Bacon & ham	22600100	Bacon, NS as to type of meat, cooked
181	A-00-03739			Bacon Fat (100% fat) 82/980	08.03.00	Fats - Animal based fats (solid)	81201000	Animal fat or drippings
182	00-03564	A-00-03564		Bacon back, L&F, fried, fat specified	12.01.00	Processed meat - Bacon & ham	22600100	Bacon, NS as to type of meat, cooked
183	A-00-03593	00-03593		Bacon middle, L & F, fried fat specified	12.01.00	Processed meat - Bacon & ham	22600100	Bacon, NS as to type of meat, cooked
184	A-00-01369			Bacon rashers, any, fried	12.01.00	Processed meat - Bacon & ham	22600100	Bacon, NS as to type of meat, cooked
185	A-00-01371			Bacon rashers, any, fried or grilled	12.01.00	Processed meat - Bacon & ham	22600100	Bacon, NS as to type of meat, cooked

186	A-00-01370			Bacon rashers, any, grilled	12.01.00	Processed meat - Bacon & ham	22600100	Bacon, NS as to type of meat, cooked
187	A-19-0002	19-0002		Bacon rashers, back, dry-fried	12.01.00	Processed meat - Bacon & ham	22600100	Bacon, NS as to type of meat, cooked
188	19-0008	A-19-0008		Bacon rashers, back, fat trimmed, grilled	12.01.00	Processed meat - Bacon & ham	22600100	Bacon, NS as to type of meat, cooked
189	A-19-0003	19-0003		Bacon rashers, back, grilled	12.01.00	Processed meat - Bacon & ham	22600100	Bacon, NS as to type of meat, cooked
190	19-0009			Bacon rashers, back, reduced salt, grilled	12.01.00	Processed meat - Bacon & ham	22600100	Bacon, NS as to type of meat, cooked
191	19-0010			Bacon rashers, back, smoked, grilled	12.01.00	Processed meat - Bacon & ham	22600100	Bacon, NS as to type of meat, cooked
192	19-0014			Bacon rashers, middle, fried	12.01.00	Processed meat - Bacon & ham	22600100	Bacon, NS as to type of meat, cooked
193	19-0015	A-19-0015		Bacon rashers, middle, grilled	12.01.00	Processed meat - Bacon & ham	22600100	Bacon, NS as to type of meat, cooked
194	A-19-0017	19-0017		Bacon rashers, streaky, fried	12.01.00	Processed meat - Bacon & ham	22600100	Bacon, NS as to type of meat, cooked
195	19-0018	A-19-0018		Bacon rashers, streaky, grilled	12.01.00	Processed meat - Bacon & ham	22600100	Bacon, NS as to type of meat, cooked
196	A-00-00215			Bacon, Collar Joint, Boiled, Lean Only	12.01.00	Processed meat - Bacon & ham	22600100	Bacon, NS as to type of meat, cooked
197	A-00-00214			Bacon, Collar Joint, Boiled, Lean and Fat	12.01.00	Processed meat - Bacon & ham	22600100	Bacon, NS as to type of meat, cooked
198	A-00-00213			Bacon, Collar Joint, Raw, Lean and Fat	12.01.00	Processed meat - Bacon & ham	22600100	Bacon, NS as to type of meat, cooked
199	A-00-00218			Bacon, Gammon Joint, Boiled, Lean Only	12.01.00	Processed meat - Bacon & ham	22600100	Bacon, NS as to type of meat, cooked
200	A-00-00217			Bacon, Gammon Joint, Boiled, Lean and Fat	12.01.00	Processed meat - Bacon & ham	22600100	Bacon, NS as to type of meat, cooked
201	A-00-00220			Bacon, Gammon Rashers, Grilled Lean Only	12.01.00	Processed meat - Bacon & ham	22600100	Bacon, NS as to type of meat, cooked
202	A-00-00219			Bacon, Gammon Rashers, Grilled Lean and Fat	12.01.00	Processed meat - Bacon & ham	22600100	Bacon, NS as to type of meat, cooked

203	A-00-01367			Bacon, Gammon joint, boiled, average	12.01.00	Processed meat - Bacon & ham	22600100	Bacon, NS as to type of meat, cooked
204	A-00-01368			Bacon, Gammon rashers, grilled, average	12.01.00	Processed meat - Bacon & ham	22600100	Bacon, NS as to type of meat, cooked
205	A-00-00210			Bacon, Lean, Average, Raw	12.01.00	Processed meat - Bacon & ham	22600100	Bacon, NS as to type of meat, cooked
206	A-00-00225			Bacon, Rashers, Fried, Average, Lean Only	12.01.00	Processed meat - Bacon & ham	22600100	Bacon, NS as to type of meat, cooked
207	A-00-00226			Bacon, Rashers, Fried, Back, Lean and Fat	12.01.00	Processed meat - Bacon & ham	22600100	Bacon, NS as to type of meat, cooked
208	A-00-00227			Bacon, Rashers, Fried, Middle, Lean and Fat	12.01.00	Processed meat - Bacon & ham	22600100	Bacon, NS as to type of meat, cooked
209	A-00-00228			Bacon, Rashers, Fried, Streaky, Lean and Fat	12.01.00	Processed meat - Bacon & ham	22600100	Bacon, NS as to type of meat, cooked
210	A-00-00230			Bacon, Rashers, Grilled, Average, Lean Only	12.01.00	Processed meat - Bacon & ham	22600100	Bacon, NS as to type of meat, cooked
211	A-00-00231			Bacon, Rashers, Grilled, Back, Lean and Fat	12.01.00	Processed meat - Bacon & ham	22600100	Bacon, NS as to type of meat, cooked
212	A-00-00232			Bacon, Rashers, Grilled, Middle, Lean and Fat	12.01.00	Processed meat - Bacon & ham	22600100	Bacon, NS as to type of meat, cooked
213	A-00-00233			Bacon, Rashers, Grilled, Streaky, Lean and Fat	12.01.00	Processed meat - Bacon & ham	22600100	Bacon, NS as to type of meat, cooked
214	A-00-00221			Bacon, Rashers, Raw, Back, Lean and Fat	12.01.00	Processed meat - Bacon & ham	22600100	Bacon, NS as to type of meat, cooked
215	A-00-00223			Bacon, Rashers, Raw, Streaky, Lean and Fat	12.01.00	Processed meat - Bacon & ham	22600100	Bacon, NS as to type of meat, cooked
216	00-03570	A-00-03570		Bacon, average, Lean only, fried fat specified	12.01.00	Processed meat - Bacon & ham	22600100	Bacon, NS as to type of meat, cooked
217	A-00-03591	00-03591		Bacon, average, Lean only, fried fat unspecified	12.01.00	Processed meat - Bacon & ham	22600100	Bacon, NS as to type of meat, cooked
218	00-03568	A-00-03568		Bacon, average, unspecified	12.01.00	Processed meat - Bacon & ham	22600100	Bacon, NS as to type of meat, cooked
219	00-03569	A-00-03569		Bacon, streaky, Lean + fat, fried fat specified	12.01.00	Processed meat - Bacon & ham	22600100	Bacon, NS as to type of meat, cooked

220	00-09608	A-00-09608		Bagels, plain	03.04.00	Breads - Other bread	51180010	Bagel
221	A-00-01100			Baked Beans with Meat	10.03.00	Meat - red - Pork & dishes	41201010	Baked beans, NFS
222	00-05924			Baked Potato Crisps in Vegetable Oil	25.01.00	Savoury Snacks - Potato based snacks	71200400	Potato chips, baked, plain
223	13-0043			Baked beans, canned in tomato sauce	16.02.00	Pulses/Lentils - Baked beans	41201010	Baked beans, NFS
224	A-13-0044	13-0044		Baked beans, canned in tomato sauce, re-heated	16.02.00	Pulses/Lentils - Baked beans	41201010	Baked beans, NFS
225	13-0045	A-13-0045		Baked beans, canned in tomato sauce, reduced sugar	16.02.00	Pulses/Lentils - Baked beans	41201010	Baked beans, NFS
226	A-13-0046	13-0046		Baked beans, canned in tomato sauce, reduced sugar, reduced salt	16.02.00	Pulses/Lentils - Baked beans	41201010	Baked beans, NFS
227	13-0048	A-13-0048		Baked beans, canned in tomato sauce, with pork sausages	16.02.00	Pulses/Lentils - Baked beans	41201010	Baked beans, NFS
228	A-00-01383			Baked beans, no added sugar	16.02.00	Pulses/Lentils - Baked beans	41201010	Baked beans, NFS
229	A-00-01047			Bakewell Tart, Frangipane Tart (Pastry and Almond Mixture)	04.02.00	Sweet cereal products - Pastries, Buns & Pies	53452100	Pastry, fruit-filled
230	A-11-0283	11-0283		Bakewell tart	04.02.00	Sweet cereal products - Pastries, Buns & Pies	53452100	Pastry, fruit-filled
231	A-00-06085			Bakewell tart 50	04.02.00	Sweet cereal products - Pastries, Buns & Pies	53452100	Pastry, fruit-filled
232	17-0355			Baking powder	26.01.00	Miscellaneous - Dried herbs & spices & pastes	18369	Leavening agents, baking powder, double-acting, sodium aluminum sulfate
233	00-03038			Balance SAINSBURYS (RISCK-LA)	02.03.00	Breakfast cereals - Other breakfast cereals - low fibre	57100100	Cereal, ready-to-eat, NFS

234	13-0163	A-13-0163		Bamboo shoots, canned, drained	15.04.00	Vegetables - Other	75203028	Bamboo shoots, cooked, NS as to fat added in cooking
235	A-00-09605	00-09605		Banana cake	04.02.00	Sweet cereal products - Pastries, Buns & Pies	53102600	Cake or cupcake, banana, without icing or filling
236	14-0044	A-14-0044		Banana chips	18.03.00	Fruit - Dried	62107200	Banana chips
237	A-00-03825			Banana nut loaf U/R 82/1929	03.04.00	Breads - Other bread	52405010	Bread, fruit
238	12-0199	A-12-0199		Banana split	06.04.01	Dairy products - Ice cream & dairy desserts - full fat products	13120400	Ice cream bar or stick with fruit
239	00-05711			Banana, dried	18.03.00	Fruit - Dried	62107200	Banana chips
240	A-14-0045	14-0045		Bananas	18.01.00	Fruit - Fresh	63107010	Banana, raw
241	A-00-00693			Bananas, Raw	18.01.00	Fruit - Fresh	63107010	Banana, raw
242	A-00-00694			Bananas, Raw (Weighed with Skins)	18.01.00	Fruit - Fresh	63107010	Banana, raw
243	A-00-09612	00-09612		Banoffee pie,	04.02.00	Sweet cereal products - Pastries, Buns & Pies	53341000	Pie, banana cream
244	A-00-03778			Barbecue Sauce (cook in) 82/1420	21.03.00	Sauces & accompaniment - Other sauces, incl. brown sauce, soy sauce, ketchup, mint sauce, vinegar	74406010	Barbecue sauce
245	17-0289	A-17-0289		Barbecue sauce	21.03.00	Sauces & accompaniment - Other sauces, incl. brown sauce, soy sauce, ketchup, mint sauce, vinegar	74406010	Barbecue sauce
246	17-0185	A-17-0185		Barley water, concentrated	27.02.03	Beverages - Fruit based drinks - Squashes & fruit concentrates	91301050	Fruit syrup
247	A-17-0186	17-0186		Barley water, concentrated, made up	27.02.03	Beverages - Fruit based drinks - Squashes & fruit concentrates	92510610	Fruit juice drink
248	A-00-00003			Barley, Pearl Boiled	01.04.00	Cereals & cereal dishes - Other cereals & dishes	56200390	Barley, NS as to fat added in cooking
249	11-0003	A-11-0003		Barley, pearl, boiled	01.04.00	Cereals & cereal dishes - Other cereals & dishes	56200390	Barley, NS as to fat added in cooking

Diet quality and cognitive ability, Cara et al.

Crosswalk linking food codes from the UK National Survey of Health and Development with the USDA Food Patterns Equivalents/Ingredients Databases

250	11-0004			Barley, whole grain, raw	01.04.00	Cereals & cereal dishes - Other cereals & dishes	20005	Barley, pearled, raw
251	A-13-0804	13-0804		Basil, fresh	15.04.00	Vegetables - Other	75109400	Basil, raw
252	00-03524			Basmati rice, boiled	01.03.00	Cereals & cereal dishes - Rice & rice dishes	56205001	Rice, white, cooked, NS as to fat added in cooking
253	16-0002			Bass, Sea, raw	09.01.00	Fish & fish dishes - White fish, incl. tuna	15091	Fish, sea bass, mixed species, raw
254	A-00-01034			Bath Bun, Chelsea Bun	04.02.00	Sweet cereal products - Pastries, Buns & Pies	51160000	Roll, sweet, no frosting
255	A-00-01029			Battenburg Cake	04.02.00	Sweet cereal products - Pastries, Buns & Pies	53102200	Cake or cupcake, applesauce, with icing or filling
256	A-11-0190	11-0190		Battenburg cake	04.02.00	Sweet cereal products - Pastries, Buns & Pies	53102200	Cake or cupcake, applesauce, with icing or filling
257	A-00-01158			Batter, Deep Fried	01.04.00	Cereals & cereal dishes - Other cereals & dishes	53452500	Pastry, mainly flour and water, fried
258	00-03608	A-00-03608		Batter, cooked in known fat	01.04.00	Cereals & cereal dishes - Other cereals & dishes	53452500	Pastry, mainly flour and water, fried
259	A-00-03607	00-03607		Batter, cooked in unknown fat	01.04.00	Cereals & cereal dishes - Other cereals & dishes	53452500	Pastry, mainly flour and water, fried
260	00-05574			Baxters Vegetarian squash & red pepper soup	20.01.00	Soups - Canned & fresh & homemade	75654010	Vegetarian vegetable soup, prepared with water
261	A-13-0806	13-0806		Bay leaf, dried	26.01.00	Miscellaneous - Dried herbs & spices & pastes	2003	Spices, basil, dried
262	A-00-01282			Bean and Gram Curry	16.01.00	Pulses/Lentils - Pulses/lentils	41311020	Sambar, vegetable stew
263	A-00-00571			Bean, Mung, Cooked, Dahl	16.01.00	Pulses/Lentils - Pulses/lentils	41101000	Beans, dry, cooked, NS as to type and as to fat added in cooking
264	A-00-00572			Bean, Red Kidney, Raw	16.01.00	Pulses/Lentils - Pulses/lentils	16069	Lentils, raw
265	15-0007			Beanburger, red kidney bean, fried in vegetable oil	16.01.00	Pulses/Lentils - Pulses/lentils	41209000	Falafel

266	A-00-00569			Beans, Baked, Canned in Tomato Sauce	16.02.00	Pulses/Lentils - Baked beans	41201010	Baked beans, NFS
267	A-00-00564			Beans, Broad, Boiled	16.01.00	Pulses/Lentils - Pulses/lentils	41101000	Beans, dry, cooked, NS as to type and as to fat added in cooking
268	A-00-00566			Beans, Butter, Boiled	16.01.00	Pulses/Lentils - Pulses/lentils	41101000	Beans, dry, cooked, NS as to type and as to fat added in cooking
269	A-00-00561			Beans, French,boiled	16.01.00	Pulses/Lentils - Pulses/lentils	41101000	Beans, dry, cooked, NS as to type and as to fat added in cooking
270	A-00-00568			Beans, Haricot, Boiled	16.01.00	Pulses/Lentils - Pulses/lentils	41101000	Beans, dry, cooked, NS as to type and as to fat added in cooking
271	A-00-00567			Beans, Haricot, Raw	16.01.00	Pulses/Lentils - Pulses/lentils	16069	Lentils, raw
272	A-00-01283			Beans, Red Kidney - Cooked	16.01.00	Pulses/Lentils - Pulses/lentils	41101000	Beans, dry, cooked, NS as to type and as to fat added in cooking
273	A-00-00563			Beans, Runner, Boiled	15.04.00	Vegetables - Other	75204960	Beans, string, cooked, NS as to form, NS as to color, made with oil
274	A-00-00562			Beans, Runner, Raw	15.04.00	Vegetables - Other	75101800	Beans, string, green, raw
275	A-00-01187			Beans, Soya, Boiled	16.01.00	Pulses/Lentils - Pulses/lentils	41101000	Beans, dry, cooked, NS as to type and as to fat added in cooking
276	A-00-00573			Beansprouts, Canned	16.01.00	Pulses/Lentils - Pulses/lentils	41101000	Beans, dry, cooked, NS as to type and as to fat added in cooking
277	A-13-0052	13-0052		Beansprouts, mung, raw	15.04.00	Vegetables - Other	75101000	Bean sprouts, raw
278	A-13-0054	13-0054		Beansprouts, mung, stir-fried in blended oil	15.04.00	Vegetables - Other	75207000	Bean sprouts, cooked, NS as to form, NS as to fat added in cooking
279	A-00-06202			Beef 50 roast topside lean and fat	10.01.00	Meat - red - Beef & veal & dishes	21401110	Beef, roast, roasted, lean and fat eaten

280	A-00-03740			Beef Fat (100% fat) 82/981	08.03.00	Fats - Animal based fats (solid)	81201000	Animal fat or drippings
281	A-00-01092			Beef Pie, 2 Crusts	12.02.00	Processed meat - Processed pies	27317010	Beef pot pie
282	A-00-00426			Beef Steak Pudding	10.01.00	Meat - red - Beef & veal & dishes	58126120	Turnover, meat-filled, with gravy
283	A-00-00427			Beef Stew	10.01.00	Meat - red - Beef & veal & dishes	27211100	Beef stew with potatoes, tomato-based sauce
284	19-0181			Beef Stroganoff	10.01.00	Meat - red - Beef & veal & dishes	27113100	Beef stroganoff
285	19-0161			Beef bourguignonne	10.01.00	Meat - red - Beef & veal & dishes	27111200	Beef burgundy
286	19-0162			Beef bourguignonne, made with lean beef	10.01.00	Meat - red - Beef & veal & dishes	27111200	Beef burgundy
287	19-0165	A-19-0165		Beef chow mein, retail, reheated	10.01.00	Meat - red - Beef & veal & dishes	27313110	Beef chow mein or chop suey with noodles
288	19-0166			Beef curry	10.01.00	Meat - red - Beef & veal & dishes	27116100	Beef curry
289	19-0169	A-19-0169		Beef curry, chilled/frozen, reheated	10.01.00	Meat - red - Beef & veal & dishes	27116100	Beef curry
290	19-0170	A-19-0170		Beef curry, chilled/frozen, reheated, with rice	10.01.00	Meat - red - Beef & veal & dishes	27116100	Beef curry
291	19-0051	A-19-0051		Beef pie, chilled/frozen, baked	12.02.00	Processed meat - Processed pies	27317010	Beef pot pie
292	A-00-06213			Beef pudding 50	10.01.00	Meat - red - Beef & veal & dishes	58126120	Turnover, meat-filled, with gravy
293	19-0076	A-19-0076		Beef sausages, chilled, fried	13.00.00	Sausages & burgers & kebab	25220105	Beef sausage
294	A-19-0077	19-0077		Beef sausages, chilled, grilled	13.00.00	Sausages & burgers & kebab	25220105	Beef sausage
295	00-03566	A-00-03566		Beef sausages, fried, fat specified	13.00.00	Sausages & burgers & kebab	25220105	Beef sausage
296	19-0052	A-19-0052		Beef steak pudding, homemade	10.01.00	Meat - red - Beef & veal & dishes	58126120	Turnover, meat-filled, with gravy

297	A-19-0175	19-0175		Beef stew	10.01.00	Meat - red - Beef & veal & dishes	27211100	Beef stew with potatoes, tomato-based sauce
298	A-00-06214			Beef stew 50	10.01.00	Meat - red - Beef & veal & dishes	27211100	Beef stew with potatoes, tomato-based sauce
299	A-00-00243			Beef, Brisket, Boiled, Lean and Fat	10.01.00	Meat - red - Beef & veal & dishes	21417110	Beef brisket, cooked, lean and fat eaten
300	A-00-00242			Beef, Brisket, Raw, Lean and Fat	10.01.00	Meat - red - Beef & veal & dishes	21417110	Beef brisket, cooked, lean and fat eaten
301	A-00-00393			Beef, Corned, Canned	10.01.00	Meat - red - Beef & veal & dishes	21416150	Corned beef, canned, ready-to-eat
302	A-00-00246			Beef, Forerib Roast, Lean Only	10.01.00	Meat - red - Beef & veal & dishes	13840	Beef, rib, large end (ribs 6-9), separable lean and fat, trimmed to 1/8" fat, all grades, cooked, roasted
303	A-00-00245			Beef, Forerib Roast, Lean and Fat	10.01.00	Meat - red - Beef & veal & dishes	13829	Beef, rib, whole (ribs 6-12), separable lean and fat, trimmed to 1/8" fat, choice, cooked, roasted
304	A-00-00247			Beef, Mince, Raw	10.01.00	Meat - red - Beef & veal & dishes	23572	Beef, ground, 80% lean meat / 20% fat, raw
305	A-00-00248			Beef, Mince, Stewed	10.01.00	Meat - red - Beef & veal & dishes	23575	Beef, ground, 80% lean meat / 20% fat, crumbles, cooked, pan-browned
306	A-00-01075			Beef, Minced, Average	10.01.00	Meat - red - Beef & veal & dishes	23575	Beef, ground, 80% lean meat / 20% fat, crumbles, cooked, pan-browned
307	A-00-01072			Beef, Minced, Fat and Lean, Stewed	10.01.00	Meat - red - Beef & veal & dishes	23580	Beef, ground, 75% lean meat / 25% fat, crumbles, cooked, pan-browned
308	A-00-01073			Beef, Minced, Fat and Lean, Stewed with Onion	10.01.00	Meat - red - Beef & veal & dishes	23580	Beef, ground, 75% lean meat / 25% fat, crumbles, cooked, pan-browned

309	A-00-01074			Beef, Minced, Fat and Lean, Stewed with Vegetables	10.01.00	Meat - red - Beef & veal & dishes	23580	Beef, ground, 75% lean meat / 25% fat, crumbles, cooked, pan-browned
310	00-01069	A-00-01069		Beef, Minced, Lean, Stewed	10.01.00	Meat - red - Beef & veal & dishes	23570	Beef, ground, 85% lean meat / 15% fat, crumbles, cooked, pan-browned
311	A-00-01070			Beef, Minced, Lean, Stewed with Onion	10.01.00	Meat - red - Beef & veal & dishes	23570	Beef, ground, 85% lean meat / 15% fat, crumbles, cooked, pan-browned
312	A-00-01071			Beef, Minced, Lean, Stewed with Vegetables	10.01.00	Meat - red - Beef & veal & dishes	23570	Beef, ground, 85% lean meat / 15% fat, crumbles, cooked, pan-browned
313	A-00-00251			Beef, Rump Steak, Fried, Lean Only	10.01.00	Meat - red - Beef & veal & dishes	21102130	Beef steak, fried, lean only eaten
314	A-00-00250			Beef, Rump Steak, Fried, Lean and Fat	10.01.00	Meat - red - Beef & veal & dishes	13933	Beef, top sirloin, steak, separable lean and fat, trimmed to 1/8" fat, choice, cooked, pan-fried
315	A-00-00253			Beef, Rump Steak, Grilled, Lean Only	10.01.00	Meat - red - Beef & veal & dishes	13491	Beef, round, top round steak, boneless, separable lean only, trimmed to 0" fat, all grades, cooked, grilled
316	A-00-00252			Beef, Rump Steak, Grilled, Lean and Fat	10.01.00	Meat - red - Beef & veal & dishes	13959	Beef, round, top round steak, boneless, separable lean and fat, trimmed to 0" fat, all grades, cooked, grilled
317	A-00-00249			Beef, Rump Steak, Raw, Lean and Fat	10.01.00	Meat - red - Beef & veal & dishes	23354	Beef, round, top round steak, boneless, separable lean only, trimmed to 0" fat, all grades, raw

318	A-00-01372			Beef, Rump steak, grilled/fried average	10.01.00	Meat - red - Beef & veal & dishes	13959	Beef, round, top round steak, boneless, separable lean and fat, trimmed to 0" fat, all grades, cooked, grilled
319	A-00-00255			Beef, Silverside, Boiled, Lean Only	10.01.00	Meat - red - Beef & veal & dishes	23590	Beef, round, bottom round , roast, separable lean only, trimmed to 1/8" fat, select, cooked, roasted
320	A-00-00254			Beef, Silverside, Salted, Boiled, Lean and Fat	10.01.00	Meat - red - Beef & veal & dishes	23590	Beef, round, bottom round , roast, separable lean only, trimmed to 1/8" fat, select, cooked, roasted
321	A-00-00256			Beef, Sirloin, Raw, Lean and Fat	10.01.00	Meat - red - Beef & veal & dishes	23625	Beef, top sirloin, steak, separable lean only, trimmed to 1/8" fat, choice, raw
322	A-00-00258			Beef, Sirloin, Roast, Lean Only	10.01.00	Meat - red - Beef & veal & dishes	13454	Beef, top sirloin, steak, separable lean only, trimmed to 0" fat, all grades, cooked, broiled
323	A-00-00257			Beef, Sirloin, Roast, Lean and Fat	10.01.00	Meat - red - Beef & veal & dishes	13451	Beef, top sirloin, steak, separable lean and fat, trimmed to 0" fat, all grades, cooked, broiled
324	A-00-01076	00-01076		Beef, Stewed, Meat and Gravy	10.01.00	Meat - red - Beef & veal & dishes	27112000	Beef with gravy
325	A-00-01077			Beef, Stewed, Meat and Gravy with Onion	10.01.00	Meat - red - Beef & veal & dishes	27112010	Salisbury steak with gravy

326	A-00-00259			Beef, Stewing Steak, Raw, Lean and Fat	10.01.00	Meat - red - Beef & veal & dishes	23612	Beef, chuck, arm pot roast, separable lean only, trimmed to 1/8" fat, choice, raw
327	A-00-00260			Beef, Stewing Steak, Stewed, Lean and Fat	10.01.00	Meat - red - Beef & veal & dishes	13812	Beef, chuck, arm pot roast, separable lean and fat, trimmed to 1/8" fat, choice, cooked, braised
328	A-00-00261			Beef, Topside, Raw, Lean and Fat	10.01.00	Meat - red - Beef & veal & dishes	23327	Beef, round, top round roast, boneless, separable lean and fat, trimmed to 0" fat, all grades, raw
329	A-00-00263			Beef, Topside, Roast, Lean Only	10.01.00	Meat - red - Beef & veal & dishes	23378	Beef, round, top round roast, boneless, separable lean only, trimmed to 0" fat, all grades, cooked, roasted
330	A-00-00262			Beef, Topside, Roast, Lean and Fat	10.01.00	Meat - red - Beef & veal & dishes	23348	Beef, round, top round roast, boneless, separable lean and fat, trimmed to 0" fat, all grades, cooked, roasted
331	18-0008			Beef, braising steak, braised, lean	10.01.00	Meat - red - Beef & veal & dishes	21105130	Beef steak, braised, lean only eaten
332	18-0009			Beef, braising steak, braised, lean and fat	10.01.00	Meat - red - Beef & veal & dishes	21105120	Beef steak, braised, lean and fat eaten
333	18-0014			Beef, brisket, boiled, lean	10.01.00	Meat - red - Beef & veal & dishes	21417120	Beef brisket, cooked, lean only eaten
334	A-18-0015	18-0015		Beef, brisket, boiled, lean and fat	10.01.00	Meat - red - Beef & veal & dishes	21417110	Beef brisket, cooked, lean and fat eaten

335	18-0018			Beef, fillet steak, fried, lean	10.01.00	Meat - red - Beef & veal & dishes	13454	Beef, top sirloin, steak, separable lean only, trimmed to 0" fat, all grades, cooked, broiled
336	18-0020			Beef, fillet steak, grilled, lean	10.01.00	Meat - red - Beef & veal & dishes	13454	Beef, top sirloin, steak, separable lean only, trimmed to 0" fat, all grades, cooked, broiled
337	18-0021			Beef, fillet steak, grilled, lean and fat	10.01.00	Meat - red - Beef & veal & dishes	13451	Beef, top sirloin, steak, separable lean and fat, trimmed to 0" fat, all grades, cooked, broiled
338	18-0034			Beef, fore-rib/rib-roast, roasted, lean & fat	10.01.00	Meat - red - Beef & veal & dishes	13829	Beef, rib, whole (ribs 6-12), separable lean and fat, trimmed to 1/8" fat, choice, cooked, roasted
339	A-18-0041	18-0041		Beef, mince, extra lean, stewed	10.01.00	Meat - red - Beef & veal & dishes	23563	Beef, ground, 90% lean meat / 10% fat, patty, cooked, broiled
340	A-18-0038	18-0038		Beef, mince, stewed	10.01.00	Meat - red - Beef & veal & dishes	23575	Beef, ground, 80% lean meat / 20% fat, crumbles, cooked, pan-browned
341	A-00-01373			Beef, roast, average	10.01.00	Meat - red - Beef & veal & dishes	21401000	Beef, roast, roasted, NS as to fat eaten
342	18-0047	A-18-0047		Beef, rump steak, fried, lean	10.01.00	Meat - red - Beef & veal & dishes	21102130	Beef steak, fried, lean only eaten
343	18-0048	A-18-0048		Beef, rump steak, fried, lean & fat	10.01.00	Meat - red - Beef & veal & dishes	13933	Beef, top sirloin, steak, separable lean and fat, trimmed to 1/8" fat, choice, cooked, pan-fried

344	18-0051	A-18-0051		Beef, rump steak, from steakhouse, lean & fat	10.01.00	Meat - red - Beef & veal & dishes	13959	Beef, round, top round steak, boneless, separable lean and fat, trimmed to 0" fat, all grades, cooked, grilled
345	18-0049	A-18-0049		Beef, rump steak, grilled, lean	10.01.00	Meat - red - Beef & veal & dishes	13491	Beef, round, top round steak, boneless, separable lean only, trimmed to 0" fat, all grades, cooked, grilled
346	18-0057			Beef, silverside, pot-roasted, lean & fat	10.01.00	Meat - red - Beef & veal & dishes	23590	Beef, round, bottom round , roast, separable lean only, trimmed to 1/8" fat, select, cooked, roasted
347	A-18-0062	18-0062		Beef, sirloin joint, roasted, lean	10.01.00	Meat - red - Beef & veal & dishes	21401120	Beef, roast, roasted, lean only eaten
348	A-18-0063	18-0063		Beef, sirloin joint, roasted, lean & fat	10.01.00	Meat - red - Beef & veal & dishes	21401110	Beef, roast, roasted, lean and fat eaten
349	18-0066			Beef, sirloin steak, fried, lean	10.01.00	Meat - red - Beef & veal & dishes	21102130	Beef steak, fried, lean only eaten
350	18-0067			Beef, sirloin steak, fried, lean & fat	10.01.00	Meat - red - Beef & veal & dishes	13933	Beef, top sirloin, steak, separable lean and fat, trimmed to 1/8" fat, choice, cooked, pan-fried
351	18-0070			Beef, sirloin steak, grilled medium-rare, lean	10.01.00	Meat - red - Beef & veal & dishes	13454	Beef, top sirloin, steak, separable lean only, trimmed to 0" fat, all grades, cooked, broiled
352	18-0071			Beef, sirloin steak, grilled medium-rare, lean & fat	10.01.00	Meat - red - Beef & veal & dishes	13451	Beef, top sirloin, steak, separable lean and fat, trimmed to 0" fat, all grades, cooked, broiled

353	18-0072			Beef, sirloin steak, grilled well-done, lean	10.01.00	Meat - red - Beef & veal & dishes	13454	Beef, top sirloin, steak, separable lean only, trimmed to 0" fat, all grades, cooked, broiled
354	18-0080			Beef, stewing steak, stewed, lean	10.01.00	Meat - red - Beef & veal & dishes	23614	Beef, chuck, arm pot roast, separable lean only, trimmed to 1/8" fat, choice, cooked, braised
355	A-18-0081	18-0081		Beef, stewing steak, stewed, lean & fat	10.01.00	Meat - red - Beef & veal & dishes	13812	Beef, chuck, arm pot roast, separable lean and fat, trimmed to 1/8" fat, choice, cooked, braised
356	A-18-0090	18-0090		Beef, topside, roasted well-done, lean	10.01.00	Meat - red - Beef & veal & dishes	23378	Beef, round, top round roast, boneless, separable lean only, trimmed to 0" fat, all grades, cooked, roasted
357	18-0091	A-18-0091		Beef, topside, roasted well-done, lean & fat	10.01.00	Meat - red - Beef & veal & dishes	23348	Beef, round, top round roast, boneless, separable lean and fat, trimmed to 0" fat, all grades, cooked, roasted
358	00-09521	A-00-09521		Beef,Stir fry,peppers & B Bean sauce	10.01.00	Meat - red - Beef & veal & dishes	27416400	Stir fried beef and vegetables in soy sauce
359	A-00-00416			Beefburgers, Frozen, Fried	13.00.00	Sausages & burgers & kebab	21500310	Ground beef patty, cooked
360	A-00-00415			Beefburgers, Frozen, Raw	13.00.00	Sausages & burgers & kebab	23572	Beef, ground, 80% lean meat / 20% fat, raw
361	A-19-0029	19-0029		Beefburgers, chilled/frozen, fried	13.00.00	Sausages & burgers & kebab	21500310	Ground beef patty, cooked
362	19-0030			Beefburgers, chilled/frozen, grilled	13.00.00	Sausages & burgers & kebab	21500310	Ground beef patty, cooked
363	00-05912			Beer (3-3.9% ABV)	27.01.03	Beverages - Alcohol - Beer	93101000	Beer
364	00-05913			Beer (4-5.5% ABV)	27.01.03	Beverages - Alcohol - Beer	93101000	Beer

365	00-05914			Beer (7-9% ABV)	27.01.03	Beverages - Alcohol - Beer	93101000	Beer
366	A-00-00892			Beer, Bitter, Canned	27.01.03	Beverages - Alcohol - Beer	93101000	Beer
367	A-00-00893			Beer, Bitter, Draught	27.01.03	Beverages - Alcohol - Beer	93101000	Beer
368	A-00-00895			Beer, Bitter, Keg	27.01.03	Beverages - Alcohol - Beer	93101000	Beer
369	A-00-00894			Beer, Mild, Draught	27.01.03	Beverages - Alcohol - Beer	93101000	Beer
370	A-00-09944			Beer, bitter, average (MW6 folate)	27.01.03	Beverages - Alcohol - Beer	93101000	Beer
371	A-00-03762			Beetroot in vinegar 82/1401	15.04.00	Vegetables - Other	75500210	Beets, pickled
372	A-00-00575			Beetroot, Boiled	15.04.00	Vegetables - Other	75208000	Beets, cooked, NS as to form, NS as to fat added in cooking
373	00-03577	A-00-03577		Beetroot, boiled, unsalted water	15.04.00	Vegetables - Other	75208000	Beets, cooked, NS as to form, NS as to fat added in cooking
374	13-0166	A-13-0166		Beetroot, pickled, drained	22.02.00	Preserves - Chutney & Pickles (incl. gherkins, pickled onions etc)	75500210	Beets, pickled
375	A-13-0164	13-0164		Beetroot, raw	15.04.00	Vegetables - Other	75102500	Beets, raw
376	A-00-00004			Bemax	02.02.00	Breakfast cereals - Other breakfast cereals - high fibre (equal or >3g/40g portion)	57412000	Wheat germ, plain
377	00-05825			Benecol fruit flavoured dairy free soya drink	05.04.00	Milk - Other - plant based, e.g. rice, soy	11519040	Strawberry milk, NFS
378	00-05394			Bernard Matthews' Turkey Ham, Wafer Thin	12.01.00	Processed meat - Bacon & ham	25230800	Turkey ham, prepackaged or deli, luncheon meat
379	A-15-0027			Bhaji, mushroom	15.04.00	Vegetables - Other	75219000	Mushrooms, cooked, NS as to form, NS as to fat added in cooking
380	15-0031			Bhaji, okra, Bangladeshi, with vegetable oil	15.04.00	Vegetables - Other	75220000	Okra, cooked, NS as to form, NS as to fat added in cooking

381	15-0035	A-15-0035		Bhaji, potato, with vegetable oil	17.01.00	Potatoes - Potatoes	71403020	Potato, home fries, NFS
382	15-0041	A-15-0041		Bhaji, spinach	15.03.00	Vegetables - Yellow & red & dark green leafy vegetables	72125200	Spinach, cooked, NS as to form, NS as to fat added in cooking
383	A-15-0046	15-0046		Bhaji, vegetable, with vegetable oil	15.04.00	Vegetables - Other	75311000	Mixed vegetables, cooked, NS as to form, NS as to fat added in cooking
384	17-0356			Bicarbonate of soda	26.01.00	Miscellaneous - Dried herbs & spices & pastes	18372	Leavening agents, baking soda
385	19-0039	A-19-0039		Big Mac	13.00.00	Sausages & burgers & kebab	27510667	Double hamburger, 2 small patties, with condiments, on bun, from fast food / restaurant
386	14-0047	A-14-0047		Bilberries	18.01.00	Fruit - Fresh	63200100	Berries, raw, NFS
387	A-00-00695			Bilberries, Raw	18.01.00	Fruit - Fresh	63200100	Berries, raw, NFS
388	00-05545			Biona Spelt & almond cutlet (vegeburger)	01.04.00	Cereals & cereal dishes - Other cereals & dishes	43128	Chicken, meatless
389	00-05342			Birds Eye Steam Fresh Veg and Rice Mix	01.03.00	Cereals & cereal dishes - Rice & rice dishes	58160700	Rice, white, with other vegetables, NS as to fat added in cooking
390	00-05800			Biscuit curls eg M&S	04.01.00	Sweet cereal products - Biscuits	53236000	Cookie, Pizzelle
391	A-00-06006			Biscuits 50 plain mixed	04.01.00	Sweet cereal products - Biscuits	54102010	Graham crackers
392	A-00-00058			Biscuits, Chocolate, Full Coated	04.01.00	Sweet cereal products - Biscuits	53209005	Cookie, chocolate, with icing or coating
393	A-00-00059			Biscuits, Cream Crackers	25.04.00	Savoury Snacks - Savoury biscuits & crackers	54307000	Crackers, matzo
394	A-00-00063			Biscuits, Digestive, Chocolate	04.01.00	Sweet cereal products - Biscuits	54102020	Graham crackers, chocolate covered
395	A-00-00062			Biscuits, Digestive, Plain	04.01.00	Sweet cereal products - Biscuits	54102010	Graham crackers
396	A-00-01025	00-01025		Biscuits, Fig Roll, Fig Newtons	04.01.00	Sweet cereal products - Biscuits	53220030	Cookie, fig bar

397	00-01026	A-00-01026		Biscuits, Garibaldi	04.01.00	Sweet cereal products - Biscuits	53237000	Cookie, raisin
398	A-00-00064			Biscuits, Ginger Nuts	04.01.00	Sweet cereal products - Biscuits	53223000	Cookie, gingersnaps
399	A-00-00065			Biscuits, Home-made	04.01.00	Sweet cereal products - Biscuits	53201000	Cookie, NFS
400	A-00-01028			Biscuits, Krackawheat	04.01.00	Sweet cereal products - Biscuits	54102010	Graham crackers
401	A-00-00066			Biscuits, Matzo	04.01.00	Sweet cereal products - Biscuits	54307000	Crackers, matzo
402	A-00-00067			Biscuits, Oatcakes	04.01.00	Sweet cereal products - Biscuits	53233040	Cookie, oatmeal, reduced fat, NS as to raisins
403	A-00-00068			Biscuits, Sandwich	04.01.00	Sweet cereal products - Biscuits	53238000	Cookie, sandwich-type, not chocolate or vanilla
404	A-00-00069			Biscuits, Semi-sweet	04.01.00	Sweet cereal products - Biscuits	53241510	Marie biscuit
405	A-00-00070			Biscuits, Short-sweet	04.01.00	Sweet cereal products - Biscuits	53239000	Cookie, shortbread
406	A-00-00071			Biscuits, Shortbread	04.01.00	Sweet cereal products - Biscuits	53239000	Cookie, shortbread
407	A-00-00072			Biscuits, Wafers, Filled	04.01.00	Sweet cereal products - Biscuits	53242000	Cookie, sugar wafer
408	A-00-00073			Biscuits, Water Biscuits	04.01.00	Sweet cereal products - Biscuits	54336000	Crackers, water
409	00-05425			Bisto Cheese Sauce Granules	21.02.00	Sauces & accompaniment - Cooking sauces, incl. gravies, pesto, cooking sauces for pasta and rice dishes	6011	Soup, cheese, canned, condensed
410	17-0207			Bitter (3.7% ABV)	27.01.03	Beverages - Alcohol - Beer	93101000	Beer
411	A-00-03857	00-03857		Bitter lemon	27.03.00	Beverages - Carbonated soft drinks	92432000	Fruit juice drink, citrus, carbonated
412	00-03868	A-00-03868		Bitter lemon, slimline	27.03.00	Beverages - Carbonated soft drinks	92432000	Fruit juice drink, citrus, carbonated
413	A-17-0208			Bitter, best/premium	27.01.03	Beverages - Alcohol - Beer	93101000	Beer
414	17-0208			Bitter, best/premium (4.3% ABV)	27.01.03	Beverages - Alcohol - Beer	93101000	Beer
415	A-17-0209			Bitter, low alcohol	27.01.06	Beverages - Alcohol - Low alcohol beer	93101000	Beer
416	17-0209			Bitter, low alcohol (0.8% ABV)	27.01.06	Beverages - Alcohol - Low alcohol beer	93101000	Beer
417	A-00-03771			Black Forrest Gateau 82/1410	04.02.00	Sweet cereal products - Pastries, Buns & Pies	53102800	Cake or cupcake, Black Forest
418	A-00-00401			Black Pudding, Fried	13.00.00	Sausages & burgers & kebab	25220210	Blood sausage

419	17-0291	A-17-0291		Black bean sauce	21.03.00	Sauces & accompaniment - Other sauces, incl. brown sauce, soy sauce, ketchup, mint sauce, vinegar	41205100	Black bean sauce
420	19-0114	A-19-0114		Black pudding, dry-fried	13.00.00	Sausages & burgers & kebab	25220210	Blood sausage
421	00-03547	A-00-03547		Black pudding, fried, specified fat	13.00.00	Sausages & burgers & kebab	25220210	Blood sausage
422	A-00-03543	00-03543		Black pudding, fried, unspecified fat	13.00.00	Sausages & burgers & kebab	25220210	Blood sausage
423	A-00-00696			Blackberries, Raw	18.01.00	Fruit - Fresh	63201010	Blackberries, raw
424	A-00-00698			Blackberries, Stewed with Sugar	18.02.00	Fruit - Canned & cooked	63201110	Blackberries, cooked or canned, NS as to sweetened or unsweetened; sweetened, NS as to type of sweetener
425	A-00-00697			Blackberries, Stewed without Sugar	18.02.00	Fruit - Canned & cooked	63201110	Blackberries, cooked or canned, NS as to sweetened or unsweetened; sweetened, NS as to type of sweetener
426	14-0048	A-14-0048		Blackberries, raw	18.01.00	Fruit - Fresh	63201010	Blackberries, raw
427	14-0049	A-14-0049		Blackberries, stewed with sugar	18.02.00	Fruit - Canned & cooked	63201110	Blackberries, cooked or canned, NS as to sweetened or unsweetened; sweetened, NS as to type of sweetener

428	14-0050	A-14-0050		Blackberries, stewed without sugar	18.02.00	Fruit - Canned & cooked	63201110	Blackberries, cooked or canned, NS as to sweetened or unsweetened; sweetened, NS as to type of sweetener
429	A-14-0051	14-0051		Blackberry and apple, stewed with sugar	18.02.00	Fruit - Canned & cooked	63101150	Applesauce with other fruits
430	14-0052			Blackberry and apple, stewed without sugar	18.02.00	Fruit - Canned & cooked	63101150	Applesauce with other fruits
431	17-0187	A-17-0187		Blackcurrant juice drink, concentrated	27.02.03	Beverages - Fruit based drinks - Squashes & fruit concentrates	91301050	Fruit syrup
432	A-17-0188	17-0188		Blackcurrant juice drink, concentrated, made up	27.02.03	Beverages - Fruit based drinks - Squashes & fruit concentrates	92510610	Fruit juice drink
433	A-11-0284	11-0284		Blackcurrant pie, pastry top and bottom	04.02.00	Sweet cereal products - Pastries, Buns & Pies	53301000	Pie, apple, two crust
434	A-00-06356			Blackcurrant pie, pastry top and bottom 50	04.02.00	Sweet cereal products - Pastries, Buns & Pies	53301000	Pie, apple, two crust
435	A-00-01122			Blackcurrants, Canned, with Sugar (Heavy Syrup)	18.02.00	Fruit - Canned & cooked	63101210	Apple, cooked or canned, with syrup
436	14-0056			Blackcurrants, canned in juice	18.02.00	Fruit - Canned & cooked	63115170	Cherries, sweet, cooked or canned, juice pack
437	14-0057			Blackcurrants, canned in syrup	18.02.00	Fruit - Canned & cooked	63101210	Apple, cooked or canned, with syrup
438	14-0053	A-14-0053		Blackcurrants, raw	18.01.00	Fruit - Fresh	63117010	Currants, raw
439	14-0054	A-14-0054		Blackcurrants, stewed with sugar	18.02.00	Fruit - Canned & cooked	63101110	Applesauce, stewed apples, NS as to sweetened or unsweetened; sweetened, NS as to type of sweetener

440	14-0055	A-14-0055		Blackcurrants, stewed without sugar	18.02.00	Fruit - Canned & cooked	63101110	Applesauce, stewed apples, NS as to sweetened or unsweetened; sweetened, NS as to type of sweetener
441	13-0063	A-13-0063		Blackeye beans, dried, boiled in unsalted water	16.01.00	Pulses/Lentils - Pulses/lentils	41101000	Beans, dry, cooked, NS as to type and as to fat added in cooking
442	A-12-0217	12-0217		Blancmange	06.04.01	Dairy products - Ice cream & dairy desserts - full fat products	13200110	Pudding, NFS
443	A-00-06008			Blancmange 50	06.04.01	Dairy products - Ice cream & dairy desserts - full fat products	13200110	Pudding, NFS
444	A-17-0015	17-0015		Blended spread (70-80% fat) inc. Clover, Golden Crown and Willow	08.04.01	Fats - Plant based fats (solid) - Full fat	81102000	Margarine, NFS
445	A-17-0016	17-0016		Blended spread, (40% fat) inc. Anchor half fat butter and Clover	08.04.02	Fats - Plant based fats (solid) - Reduced fat	81102000	Margarine, NFS
446	16-0170			Bloater, grilled	09.02.00	Fish & fish dishes - Oily fish	26119190	Herring, smoked, kippered
447	00-05584			Blue Dragon Fish Sauce	21.03.00	Sauces & accompaniment - Other sauces, incl. brown sauce, soy sauce, ketchup, mint sauce, vinegar	27150210	Fish sauce
448	00-05556			Blue dragon stir fry Chow Mein sauce	21.02.00	Sauces & accompaniment - Cooking sauces, incl. gravies, pesto, cooking sauces for pasta and rice dishes	28520000	Gravy or sauce, made with soy sauce, stock or bouillon, cornstarch
449	A-00-03441			BlueBand Soft Margarine (Soya Based)	08.04.01	Fats - Plant based fats (solid) - Full fat	81102000	Margarine, NFS
450	00-05559			Blueberry juice drink	27.02.02	Beverages - Fruit based drinks - Fruit juice drinks	92531030	Fruit juice drink (Sunny D)

451	A-00-00856			Boiled Sweets	24.02.00	Confectionary - Sugar based products	91700010	Candy, NFS
452	A-17-0101	17-0101		Boiled sweets	24.02.00	Confectionary - Sugar based products	91700010	Candy, NFS
453	A-00-00428			Bolognese Sauce	10.01.00	Meat - red - Beef & veal & dishes	27162040	Spaghetti sauce with meat
454	19-0183	A-19-0183		Bolognese sauce	10.01.00	Meat - red - Beef & veal & dishes	27162040	Spaghetti sauce with meat
455	00-05868			Bolognese sauce, cooked in specified fat	10.01.00	Meat - red - Beef & veal & dishes	27162040	Spaghetti sauce with meat
456	A-00-01311			Bombay Duck	09.01.00	Fish & fish dishes - White fish, incl. tuna	26100110	Fish, NS as to type, cooked, NS as to cooking method
457	16-0005			Bombay duck	09.01.00	Fish & fish dishes - White fish, incl. tuna	26100110	Fish, NS as to type, cooked, NS as to cooking method
458	14-0807	A-14-0807		Bombay mix	19.00.00	Nuts & Seeds (incl. peanut butter)	42110000	Mixed nuts, NFS
459	A-00-00937			Bone and Vegetable Broth	20.01.00	Soups - Canned & fresh & homemade	75657000	Vegetable broth, bouillon
460	17-0082	A-17-0082		Bounty bar	24.01.00	Confectionary - Chocolate based products	91726420	3 MUSKETEERS Bar
461	A-00-00867			Bournvita	27.05.00	Beverages - Powdered Beverages (cocoa, Horlicks, Bonvita, Ovaltine, etc)	11830260	Milk, malted, dry mix, not reconstituted
462	A-12-0072	A-00-03884	12-0072	Bournvita powder	27.05.00	Beverages - Powdered Beverages (cocoa, Horlicks, Bonvita, Ovaltine, etc)	11830260	Milk, malted, dry mix, not reconstituted
463	A-12-0074			Bournvita powder, made up with semi-skimmed milk	27.05.00	Beverages - Powdered Beverages (cocoa, Horlicks, Bonvita, Ovaltine, etc)	11526000	Milk, malted
464	A-12-0075			Bournvita powder, made up with skimmed milk	27.05.00	Beverages - Powdered Beverages (cocoa, Horlicks, Bonvita, Ovaltine, etc)	11526000	Milk, malted

465	A-12-0073			Bournvita powder, made up with whole milk	27.05.00	Beverages - Powdered Beverages (cocoa, Horlicks, Bonvita, Ovaltine, etc)	11526000	Milk, malted
466	A-00-00957			Bovril	26.01.00	Miscellaneous - Dried herbs & spices & pastes	2023	Spices, marjoram, dried
467	00-05887			Braggs Aminos Liquid	21.03.00	Sauces & accompaniment - Other sauces, incl. brown sauce, soy sauce, ketchup, mint sauce, vinegar	41420300	Soy sauce
468	A-00-00354			Brain, Calf and Lamb, Raw	14.02.00	Offal - Other offal & dishes, e.g. Haggis, faggots	5027	Chicken, liver, all classes, raw
469	18-0392			Brain, lamb, boiled	14.02.00	Offal - Other offal & dishes, e.g. Haggis, faggots	25150000	Brains, cooked
470	A-11-0127			Bran Buds	02.02.00	Breakfast cereals - Other breakfast cereals - high fibre (equal or >3g/40g portion)	57110000	Cereal (Kellogg's All-Bran Bran Buds)
471	A-11-0128	A-00-01019		Bran Flakes	02.02.00	Breakfast cereals - Other breakfast cereals - high fibre (equal or >3g/40g portion)	57207000	Cereal, bran flakes
472	A-00-01018			Bran Plus, Allinson's	02.03.00	Breakfast cereals - Other breakfast cereals - low fibre	57100100	Cereal, ready-to-eat, NFS
473	A-00-00005			Bran, Wheat	01.04.00	Cereals & cereal dishes - Other cereals & dishes	57601100	Wheat bran, unprocessed
474	A-11-0005	11-0005		Bran, wheat	01.04.00	Cereals & cereal dishes - Other cereals & dishes	57601100	Wheat bran, unprocessed
475	A-00-03005			Branbuds, Kelloggs	02.02.00	Breakfast cereals - Other breakfast cereals - high fibre (equal or >3g/40g portion)	57110000	Cereal (Kellogg's All-Bran Bran Buds)
476	A-11-0165	11-0165		Brandy snaps	04.02.00	Sweet cereal products - Pastries, Buns & Pies	53242000	Cookie, sugar wafer
477	00-03036			Branflakes SAINSBURYS (RISCK-LA)	02.02.00	Breakfast cereals - Other breakfast cereals - high fibre (equal or >3g/40g portion)	57207000	Cereal, bran flakes

478	00-03006	A-00-03006		Branflakes, Kelloggs	02.02.00	Breakfast cereals - Other breakfast cereals - high fibre (equal or >3g/40g portion)	57207000	Cereal, bran flakes
479	00-03007	A-00-03007		Branflakes, Own Brand	02.02.00	Breakfast cereals - Other breakfast cereals - high fibre (equal or >3g/40g portion)	57207000	Cereal, bran flakes
480	00-05540			Branflakes, sultana, Kelloggs	02.02.00	Breakfast cereals - Other breakfast cereals - high fibre (equal or >3g/40g portion)	57329000	Cereal, raisin bran
481	A-19-0115	A-00-00417		Brawn	10.03.00	Meat - red - Pork & dishes	25220910	Head cheese
482	A-00-00826			Brazil Nuts	19.00.00	Nuts & Seeds (incl. peanut butter)	42102000	Brazil nuts
483	14-0808	A-14-0808		Brazil nuts	19.00.00	Nuts & Seeds (incl. peanut butter)	42102000	Brazil nuts
484	A-00-06001			Bread 50 National Wheatmeal	03.04.00	Breads - Other bread	51301010	Bread, wheat or cracked wheat
485	00-03334			Bread Multigrain seeded wholemeal (ave Tesco & Hovis)	03.04.00	Breads - Other bread	51601020	Bread, multigrain
486	A-00-00920			Bread Sauce	21.03.00	Sauces & accompaniment - Other sauces, incl. brown sauce, soy sauce, ketchup, mint sauce, vinegar	13412000	Milk gravy, quick gravy
487	A-00-00098			Bread and Butter Pudding	04.03.00	Sweet cereal products - Cereal based puddings (not milk)	13210110	Pudding, bread
488	11-0286	A-11-0286		Bread and butter pudding	04.03.00	Sweet cereal products - Cereal based puddings (not milk)	13210110	Pudding, bread
489	A-00-06043			Bread and butter pudding 50	04.04.00	Sweet cereal products - Milk based puddings	13210110	Pudding, bread
490	11-0287	A-11-0287		Bread pudding	04.03.00	Sweet cereal products - Cereal based puddings (not milk)	13210110	Pudding, bread
491	A-00-06044			Bread pudding 50	04.03.00	Sweet cereal products - Cereal based puddings (not milk)	13210110	Pudding, bread

492	A-12-0262			Bread sauce, made with semi-skimmed milk	21.03.00	Sauces & accompaniment - Other sauces, incl. brown sauce, soy sauce, ketchup, mint sauce, vinegar	13412000	Milk gravy, quick gravy
493	A-12-0263			Bread sauce, made with skimmed milk	21.03.00	Sauces & accompaniment - Other sauces, incl. brown sauce, soy sauce, ketchup, mint sauce, vinegar	13412000	Milk gravy, quick gravy
494	A-12-0261			Bread sauce, made with whole milk	21.03.00	Sauces & accompaniment - Other sauces, incl. brown sauce, soy sauce, ketchup, mint sauce, vinegar	13412000	Milk gravy, quick gravy
495	A-00-09828	00-09828		Bread sauce, with semi-skimmed milk	21.03.00	Sauces & accompaniment - Other sauces, incl. brown sauce, soy sauce, ketchup, mint sauce, vinegar	13412000	Milk gravy, quick gravy
496	A-00-09829	00-09829		Bread sauce, with skimmed milk	21.03.00	Sauces & accompaniment - Other sauces, incl. brown sauce, soy sauce, ketchup, mint sauce, vinegar	13412000	Milk gravy, quick gravy
497	A-00-09833			Bread sauce, with whole milk	21.03.00	Sauces & accompaniment - Other sauces, incl. brown sauce, soy sauce, ketchup, mint sauce, vinegar	13412000	Milk gravy, quick gravy
498	A-00-00031			Bread, Brown	03.03.00	Breads - Brown/Granary/Wheatgerm	51301010	Bread, wheat or cracked wheat
499	A-00-00037			Bread, Currant	03.04.00	Breads - Other bread	51129010	Bread, raisin
500	A-00-01014			Bread, Currant, Toasted	03.04.00	Breads - Other bread	51129020	Bread, raisin, toasted
501	00-01011	A-00-01011		Bread, Fried, Brown	03.03.00	Breads - Brown/Granary/Wheatgerm	51301010	Bread, wheat or cracked wheat
502	A-00-06036			Bread, Fried, Brown (bacon fat) 50	03.03.00	Breads - Brown/Granary/Wheatgerm	51301010	Bread, wheat or cracked wheat
503	A-00-00032			Bread, Hovis	03.01.00	Breads - White	51101000	Bread, white
504	A-00-00038			Bread, Malt	03.04.00	Breads - Other bread	52405010	Bread, fruit

505	A-00-00039			Bread, Soda	03.04.00	Breads - Other bread	52408000	Bread, Irish soda
506	A-00-00033			Bread, White	03.01.00	Breads - White	51101000	Bread, white
507	A-00-00036			Bread, White, Dried Crumbs	03.01.00	Breads - White	18079	Bread crumbs, dry, grated, plain
508	A-00-00034			Bread, White, Fried	03.01.00	Breads - White	51101000	Bread, white
509	A-00-00035			Bread, White, Toasted	03.01.00	Breads - White	51101010	Bread, white, toasted
510	A-00-00030			Bread, Wholemeal	03.02.00	Breads - Wholemeal	51300110	Bread, whole wheat
511	A-11-0068	11-0068		Breadcrumbs, homemade	03.01.00	Breads - White	18079	Bread crumbs, dry, grated, plain
512	11-0069	A-11-0069		Breadcrumbs, manufactured	03.01.00	Breads - White	18069	Bread, white, commercially prepared (includes soft bread crumbs)
513	00-05582			Breaded Onion Rings (e.g. Tesco)	15.04.00	Vegetables - Other	75415020	Onion rings, NS as to form, batter-dipped, baked or fried
514	17-0123	A-17-0123		Breadsticks	03.05.00	Breads - Crisp Breads, e.g. Rivetas, Grissini, Toast Melba	51184000	Breadsticks, hard, NFS
515	A-00-09737			Breakfast milk, pasteurised, summer	05.03.00	Milk - Whole milk	11100000	Milk, NFS
516	19-0053	A-19-0053		Bridie/Scotch pie, individual	12.02.00	Processed meat - Processed pies	27360050	Meat pie, NFS
517	00-09584	A-00-09584		Brioche	03.04.00	Breads - Other bread	51167000	Brioche
518	00-03867	A-00-03867		Britvic 55	27.02.02	Beverages - Fruit based drinks - Fruit juice drinks	92531030	Fruit juice drink (Sunny D)
519	13-0066	A-13-0066		Broad beans, boiled in unsalted water	15.04.00	Vegetables - Other	41102200	Fava beans, dry, cooked, NS as to fat added in cooking
520	A-13-0069	13-0069		Broad beans, canned, re-heated, drained	15.04.00	Vegetables - Other	41102200	Fava beans, dry, cooked, NS as to fat added in cooking
521	13-0068	A-13-0068		Broad beans, frozen, boiled in unsalted water	15.04.00	Vegetables - Other	41102200	Fava beans, dry, cooked, NS as to fat added in cooking

522	13-0064			Broad beans, raw	15.04.00	Vegetables - Other	41102260	Fava beans, canned, drained, fat added in cooking
523	00-03933			Broccoli & Stilton Soup (eg. Sainsburys)	20.01.00	Soups - Canned & fresh & homemade	72302100	Broccoli cheese soup, prepared with milk, home recipe, canned, or ready-to-serve
524	A-00-00577			Broccoli Tops, Boiled	15.02.00	Vegetables - Brassicacea	72201211	Broccoli, cooked, from fresh, fat not added in cooking
525	A-00-00576			Broccoli Tops, Raw	15.02.00	Vegetables - Brassicacea	72201100	Broccoli, raw
526	A-13-0172	13-0172		Broccoli, green, boiled in unsalted water	15.02.00	Vegetables - Brassicacea	72201211	Broccoli, cooked, from fresh, fat not added in cooking
527	13-0170	A-13-0170		Broccoli, green, raw	15.02.00	Vegetables - Brassicacea	72201100	Broccoli, raw
528	A-13-0176	13-0176		Broccoli, purple sprouting, boiled in unsalted water	15.02.00	Vegetables - Brassicacea	72201211	Broccoli, cooked, from fresh, fat not added in cooking
529	A-13-0174	13-0174		Broccoli, purple sprouting, raw	15.02.00	Vegetables - Brassicacea	72201100	Broccoli, raw
530	A-00-00891			Brown Ale, Bottled Beer	27.01.03	Beverages - Alcohol - Beer	93101000	Beer
531	A-00-09595	00-09595		Brown Bread toasted	03.03.00	Breads - Brown/Granary/Wheatgerm	51301020	Bread, wheat or cracked wheat, toasted
532	A-00-00921			Brown Sauce, Bottled	21.03.00	Sauces & accompaniment - Other sauces, incl. brown sauce, soy sauce, ketchup, mint sauce, vinegar	74406100	Steak sauce, tomato-base
533	A-17-0210			Brown ale, bottled	27.01.03	Beverages - Alcohol - Beer	93101000	Beer
534	17-0210			Brown ale, bottled (3.2% ABV)	27.01.03	Beverages - Alcohol - Beer	93101000	Beer
535	A-11-0070	A-00-09561	00-09561	Brown bread, average	03.03.00	Breads - Brown/Granary/Wheatgerm	51301010	Bread, wheat or cracked wheat
536	A-11-0073			Brown bread, toasted	03.03.00	Breads - Brown/Granary/Wheatgerm	51301020	Bread, wheat or cracked wheat, toasted

Diet quality and cognitive ability, Cara et al.
Crosswalk linking food codes from the UK National Survey of Health and Development with the USDA Food Patterns Equivalents/Ingredients Databases

537	11-0036	A-11-0036		Brown rice, boiled	01.03.00	Cereals & cereal dishes - Rice & rice dishes	56205011	Rice, brown, cooked, NS as to fat added in cooking
538	A-11-0118			Brown rolls, crusty	03.03.00	Breads - Brown/Granary/Wheatgerm	51320010	Roll, wheat or cracked wheat
539	A-11-0119			Brown rolls, soft	03.03.00	Breads - Brown/Granary/Wheatgerm	51320010	Roll, wheat or cracked wheat
540	A-00-09566	00-09566		Brown rolls, soft or crusty	03.03.00	Breads - Brown/Granary/Wheatgerm	51320010	Roll, wheat or cracked wheat
541	17-0293	A-17-0293		Brown sauce, sweet	21.03.00	Sauces & accompaniment - Other sauces, incl. brown sauce, soy sauce, ketchup, mint sauce, vinegar	74406100	Steak sauce, tomato-base
542	A-00-00579			Brussels Sprouts, Boiled	15.02.00	Vegetables - Brassicacea	75209000	Brussels sprouts, cooked, NS as to form, NS as to fat added in cooking
543	A-00-00578			Brussels Sprouts, Raw	15.02.00	Vegetables - Brassicacea	75102750	Brussels sprouts, raw
544	A-13-0179	13-0179		Brussels sprouts, boiled in unsalted water	15.02.00	Vegetables - Brassicacea	75209000	Brussels sprouts, cooked, NS as to form, NS as to fat added in cooking
545	A-13-0181	13-0181		Brussels sprouts, frozen, boiled in unsalted water	15.02.00	Vegetables - Brassicacea	75209000	Brussels sprouts, cooked, NS as to form, NS as to fat added in cooking
546	A-00-01103			Bubble and Squeak	15.04.00	Vegetables - Other	71403500	Potato, home fries, with vegetables
547	15-0052			Bubble and squeak, fried in lard	15.04.00	Vegetables - Other	71403500	Potato, home fries, with vegetables
548	15-0053	A-15-0053		Bubble and squeak, fried in sunflower oil	15.04.00	Vegetables - Other	71403500	Potato, home fries, with vegetables
549	A-15-0054	15-0054		Bubble and squeak, fried in vegetable oil	15.04.00	Vegetables - Other	71403500	Potato, home fries, with vegetables
550	11-0006			Buckwheat	01.04.00	Cereals & cereal dishes - Other cereals & dishes	56200490	Buckwheat groats, NS as to fat added in cooking

551	A-12-0077			Build-up powder, made up with whole milk	30.00.00	Nutrition Powders & drinks	11514110	Hot chocolate / Cocoa, made with dry mix and whole milk
552	A-00-01140			Build-up, Chocolate Flavour	30.00.00	Nutrition Powders & drinks	95220000	Nutritional powder mix, NFS
553	11-0007	A-11-0007		Bulgur wheat	01.04.00	Cereals & cereal dishes - Other cereals & dishes	20013	Bulgur, cooked
554	A-00-03332	00-03332		Burgen Bread	03.04.00	Breads - Other bread	51601020	Bread, multigrain
555	00-05461			Burgen Soya and Linseed Bread	03.04.00	Breads - Other bread	51601020	Bread, multigrain
556	A-17-0013	A-12-0256		Butter	08.01.00	Fats - Butter	81100500	Butter, NFS
557	A-00-03139	00-03139		Butter Biscuits	25.04.00	Savoury Snacks - Savoury biscuits & crackers	54301030	Crackers, butter (Ritz)
558	A-00-03772			Butter Icing 82/1411	23.02.00	Sugars - Other, incl. syrups, honey	91305020	Icing, white
559	00-05442			Butter Slightly Salted e.g. Tesco Sainsbury Lurpak	08.01.00	Fats - Butter	81100500	Butter, NFS
560	A-13-0072	13-0072		Butter beans, canned, re-heated, drained	16.01.00	Pulses/Lentils - Pulses/lentils	41101000	Beans, dry, cooked, NS as to type and as to fat added in cooking
561	A-00-06153			Butter margarine composite 50	08.04.01	Fats - Plant based fats (solid) - Full fat	81102000	Margarine, NFS
562	A-00-00140			Butter, Salted	08.01.00	Fats - Butter	81100500	Butter, NFS
563	17-0013			Butter, salted	08.01.00	Fats - Butter	81100500	Butter, NFS
564	17-0014	A-17-0014		Butter, spreadable	08.01.00	Fats - Butter	81100500	Butter, NFS
565	00-05681			Butter, spreadable, unsalted	08.01.00	Fats - Butter	81100500	Butter, NFS
566	00-09776	A-00-09776		Butter, unsalted	08.01.00	Fats - Butter	81100500	Butter, NFS
567	12-0022			Buttermilk	06.03.02	Dairy products - Yoghurt & drinking yoghurts, incl. buttermilk and probiotics - reduced or low fat products	11115300	Buttermilk, whole
568	02-04024			CAESAR SALAD DRESSING	21.01.00	Sauces & accompaniment - Dressings & Mayonnaise	83102000	Caesar dressing

569	02-06061			CAKE BARS REDUCED FAT E.G GO-AHEAD DOUBLE CARAMEL, DOUBLE CHOCOLATE	04.02.00	Sweet cereal products - Pastries, Buns & Pies	53108220	Snack cake, chocolate, with icing or filling, reduced fat and calories
570	02-03992			CAKE BARS, NOT CHOCOLATE, INDIVIDUAL, PURCHASED	04.02.00	Sweet cereal products - Pastries, Buns & Pies	53109200	Snack cake, not chocolate, with icing or filling
571	02-08122			CAKE, NOT CHOCOLATE, WITH BUTTERCREAM AND/OR ICING, PURCHASED	04.02.00	Sweet cereal products - Pastries, Buns & Pies	53114100	Cake or cupcake, lemon, with icing or filling
572	02-07965			CANDY TOTS SKITTLES TOOTY FROOTIES JELLY BEANS	24.02.00	Confectionary - Sugar based products	91700010	Candy, NFS
573	02-09389			CAPERS	15.04.00	Vegetables - Other	2054	Capers, canned
574	02-08048			CAPPUCCINO (ESPRESSO AND SKIMMED MILK) TAKEAWAY ONLY	27.06.00	Beverages - Coffee	92161000	Coffee, Cappuccino
575	02-08042			CAPPUCCINO (ESPRESSO AND WHOLE MILK) TAKEAWAY ONLY	27.06.00	Beverages - Coffee	92161000	Coffee, Cappuccino
576	02-06840			CAPPUCCINO, INSTANT, WITH WHITENER AND SUGAR, DRY WEIGHT EG. SAINSBURYS	27.06.00	Beverages - Coffee	14192	Beverages, Cocoa mix, powder
577	02-02844			CAPPUCCINO, INSTANT, WITH WHITENER, NO SUGAR, DRY WEIGHT	27.06.00	Beverages - Coffee	14192	Beverages, Cocoa mix, powder
578	02-08672			CARAMEL SHORTCAKE PURCHASED	04.01.00	Sweet cereal products - Biscuits	52105100	Scone
579	02-07902			CARBONATED BEVERAGE NO JUICE CANNED LOW CALORIE	27.03.00	Beverages - Carbonated soft drinks	92400000	Soft drink, NFS

580	02-07903			CARBONATED BEVERAGES NO JUICE LOW CAL NOT CANNED	27.03.00	Beverages - Carbonated soft drinks	92400000	Soft drink, NFS
581	02-07900			CARBONATED BEVERAGES NO JUICE NOT LOW CAL CANNED	27.03.00	Beverages - Carbonated soft drinks	92400000	Soft drink, NFS
582	02-07901			CARBONATED BEVS NO JUICE NOT LOW CAL NOT CANNED	27.03.00	Beverages - Carbonated soft drinks	92400000	Soft drink, NFS
583	02-08360			CARBONATED DRINK <50% JUICE LOW CAL CANNED	27.03.00	Beverages - Carbonated soft drinks	92433000	Fruit juice drink, noncitrus, carbonated
584	02-08328			CARBONATED DRINK <50% JUICE NOT LOW CAL	27.03.00	Beverages - Carbonated soft drinks	92433000	Fruit juice drink, noncitrus, carbonated
585	02-10073			CARROT CAKE NO ICING, PURCHASED	04.02.00	Sweet cereal products - Pastries, Buns & Pies	53104100	Cake or cupcake, carrot, without icing or filling
586	02-07929			CARTON SOUP OTHER READY SERVE	20.01.00	Soups - Canned & fresh & homemade	58400000	Soup, NFS
587	02-06883			CEREAL BAR WITH FRUITS, NO NUTS, NOT COATED (UF)	04.05.00	Sweet cereal products - Cereal bars	53710500	Cereal or granola bar (Kellogg's Nutri-Grain Cereal Bar)
588	02-10060			CEREAL BARS WITH FRUIT AND NUTS, COATED, UNFORTIFIED	04.05.00	Sweet cereal products - Cereal bars	53710504	Cereal or granola bar (Kellogg's Nutri-Grain Fruit and Nut Bar)
589	02-10059			CEREAL BARS WITH FRUIT AND NUTS, NOT COATED, UNFORTIFIED	04.05.00	Sweet cereal products - Cereal bars	53710504	Cereal or granola bar (Kellogg's Nutri-Grain Fruit and Nut Bar)
590	02-10057			CEREAL BARS WITH FRUIT, NO NUTS, COATED, UNFORTIFIED	04.05.00	Sweet cereal products - Cereal bars	53710502	Cereal or granola bar (Kellogg's Nutri-Grain Yogurt Bar)
591	02-10058			CEREAL BARS WITH NUTS, NO FRUIT, NOT COATED, UNFORTIFIED	04.05.00	Sweet cereal products - Cereal bars	53714220	Cereal or granola bar with nuts, chocolate coated
592	00-05692			CHAPATIS, WHOLEMEAL, MADE WITH BVO	03.04.00	Breads - Other bread	52215260	Tortilla, whole wheat

593	02-10543			CHEDDAR REDUCED FAT 21-23G/100G	06.02.00	Dairy products - Cheese, incl. cottage cheese	14104100	Cheese, Cheddar
594	02-09861			CHEESE & TOMATO QUICHE	07.00.00	Egg & egg dishes	58125180	Cheese quiche, meatless
595	02-04121			CHEESE AND ONION CRISPBAKES, PURCHASED EG M & S	01.04.00	Cereals & cereal dishes - Other cereals & dishes	58127150	Vegetables and cheese in pastry
596	02-09497			CHEESE AND ONION MAYONNAISE SANDWICH FILLINGS PURCHASED	06.02.00	Dairy products - Cheese, incl. cottage cheese	14420100	Cheese spread, American or Cheddar cheese base
597	02-10178			CHEESE AND VEGETABLE QUICHE PURCHASED	07.00.00	Egg & egg dishes	58125120	Spinach quiche, meatless
598	02-00688			CHEESE CREAM FULLFAT	06.02.00	Dairy products - Cheese, incl. cottage cheese	14420200	Cheese spread, cream cheese, regular
599	02-07727			CHEESE EDAM REDUCED FAT	06.02.00	Dairy products - Cheese, incl. cottage cheese	14105010	Cheese, Gouda or Edam
600	02-10033			CHEESE FEAST PIZZA & TOMATO WITH A STUFFED CRUST BASE. RETAIL	01.01.00	Cereals & cereal dishes - Pizza	58106233	Pizza, cheese, stuffed crust
601	02-06981			CHEESE HALLOUMI	06.02.00	Dairy products - Cheese, incl. cottage cheese	14104400	Cheese, Feta
602	02-06980			CHEESE PANEER	06.02.00	Dairy products - Cheese, incl. cottage cheese	14133000	Queso Fresco
603	02-00252			CHEESE SANDWICH BISCUITS	25.04.00	Savoury Snacks - Savoury biscuits & crackers	54328200	Crackers, sandwich, cheese filled
604	02-08947			CHEESE SAUCE PACKET MIX DRY	06.02.00	Dairy products - Cheese, incl. cottage cheese	14650100	Cheese sauce
605	00-05780			CHEESE SPREADS, TRIANGLES, PLAIN, DAIRYLEA ONLY	06.02.00	Dairy products - Cheese, incl. cottage cheese	14420200	Cheese spread, cream cheese, regular
606	02-10055			CHEESE STRAWS/TWISTS/CRISPIES PURCHASED	04.01.00	Sweet cereal products - Biscuits	53241500	Cookie, butter or sugar
607	02-00680			CHEESE-STILTON WHITE	06.02.00	Dairy products - Cheese, incl. cottage cheese	14101010	Cheese, Blue or Roquefort

608	02-08303			CHEWY MINTS (MILD)	24.02.00	Confectionary - Sugar based products	91700010	Candy, NFS
609	02-04123			CHICKEN AND LEMON RISOTTO FROZEN READY MEAL WEIGHTWATCHERS ONLY	01.03.00	Cereals & cereal dishes - Rice & rice dishes	27243000	Chicken or turkey and rice, no sauce
610	02-08839			CHICKEN AND MUSHROOM PASTIES PUFF PASTRY PURCHASED M&S	12.02.00	Processed meat - Processed pies	58126170	Turnover filled with meat and vegetable, no potatoes, no gravy
611	02-06423			CHICKEN AND PASTA BAKE READY MEAL EG. SAINSBURYS	01.02.00	Cereals & cereal dishes - Pasta & pasta dishes	58146323	Pasta with tomato-based sauce and meat, ready-to-heat
612	02-10270			CHICKEN AND SWEETCORN SOUP	20.01.00	Soups - Canned & fresh & homemade	28340590	Chicken or turkey corn soup with noodles, home recipe
613	02-08025			CHICKEN BHUNA TAKEAWAY OR PURCHASED	11.01.00	Meat - white - Chicken & turkey & dishes	27146150	Chicken curry
614	02-06991			CHICKEN BIRYANI WITH RICE, TAKEAWAY	01.03.00	Cereals & cereal dishes - Rice & rice dishes	27243100	Biryani with chicken
615	02-09287			CHICKEN BREAST COATED GRILLED	11.01.00	Meat - white - Chicken & turkey & dishes	24127500	Chicken breast, baked, coated, skin / coating eaten
616	02-05262			CHICKEN BURGER BUN LETTUCE AND MAYO TAKE AWAY	11.01.00	Meat - white - Chicken & turkey & dishes	27540190	Chicken patty sandwich, with lettuce and spread
617	02-04035			CHICKEN CHOW MEIN READY MEAL	11.01.00	Meat - white - Chicken & turkey & dishes	27343910	Chicken or turkey chow mein or chop suey with noodles
618	02-01103			CHICKEN CURRY CANNED NO RICE	11.01.00	Meat - white - Chicken & turkey & dishes	27146150	Chicken curry
619	02-05290			CHICKEN CURRY TAKEAWAY EG CHICKEN DUPIAZA	11.01.00	Meat - white - Chicken & turkey & dishes	27146150	Chicken curry

620	02-02957			CHICKEN CURRY, LOW FAT WITH COCONUT, TOMATO AND RICE	11.01.00	Meat - white - Chicken & turkey & dishes	27146150	Chicken curry
621	02-02706			CHICKEN ESCALOPE WITH CORONATION SAUCE EG BERNARD MATTHEWS	11.01.00	Meat - white - Chicken & turkey & dishes	27146150	Chicken curry
622	02-08254			CHICKEN FINGERS COATED FRIED IN BLENDED VEG OIL	11.01.00	Meat - white - Chicken & turkey & dishes	24198739	Chicken tenders or strips, NFS
623	02-08258			CHICKEN FINGERS COATED GRILLED	11.01.00	Meat - white - Chicken & turkey & dishes	24198739	Chicken tenders or strips, NFS
624	02-03490			CHICKEN HOTPOT, REDUCED FAT E.G. WW	11.01.00	Meat - white - Chicken & turkey & dishes	28340580	Chicken or turkey soup with vegetables, broccoli, carrots, celery, potatoes and onions, Asian style
625	02-01108			CHICKEN IN WHITE SAUCE CANNED	11.01.00	Meat - white - Chicken & turkey & dishes	27143000	Chicken or turkey with cream sauce
626	02-08259			CHICKEN KIEV MINI GRILLED	11.01.00	Meat - white - Chicken & turkey & dishes	27146400	Chicken kiev
627	02-10390			CHICKEN KIEVS REDUCED FAT OR CALORIE PURCHASED	11.01.00	Meat - white - Chicken & turkey & dishes	27146400	Chicken kiev
628	02-07094			CHICKEN KORMA WITH RICE READY MEAL	11.01.00	Meat - white - Chicken & turkey & dishes	27146150	Chicken curry
629	02-08098			CHICKEN MAYONNAISE SANDWICH FILLERS	11.01.00	Meat - white - Chicken & turkey & dishes	25240110	Chicken salad spread
630	02-05466			CHICKEN MEATBALLS IN TOMATO SAUCE	11.01.00	Meat - white - Chicken & turkey & dishes	27160100	Meatballs, NS as to type of meat, with sauce
631	02-01116			CHICKEN ROAST DINNER PURCHASED + POTATO VEG STUFFI	11.01.00	Meat - white - Chicken & turkey & dishes	28140710	Chicken, fried, with potatoes, vegetable, frozen meal
632	02-05285			CHICKEN SLICES SMOKED PREPACKED OR DELI INCL WAFER THIN	11.01.00	Meat - white - Chicken & turkey & dishes	25230320	Chicken, prepackaged or deli, luncheon meat

633	02-01107			CHICKEN SUPREME CREAMED CHICKEN NO BONES	11.01.00	Meat - white - Chicken & turkey & dishes	27143000	Chicken or turkey with cream sauce
634	02-03768			CHICKEN TIKKA SANDWICH FILLING, LOW FAT E.G. ASDA	11.01.00	Meat - white - Chicken & turkey & dishes	27146150	Chicken curry
635	02-06156			CHINESE DUMPLINGS	01.04.00	Cereals & cereal dishes - Other cereals & dishes	58110130	Egg roll, with beef and/or pork
636	02-01729			CHINESE LEAVES FRESH RAW	15.02.00	Vegetables - Brassicacea	75104000	Cabbage, Chinese, raw
637	02-08750			CHIPS OLD FRESH FRIED IN OLIVE OIL	17.02.00	Potatoes - Potato products - other	71400990	Potato, french fries, NFS
638	02-07875			CHIPSTICKS OTHER POTATO & CORN STICKS	25.02.00	Savoury Snacks - Cereal based snacks	71205020	Potato sticks, plain
639	02-08138			CHOC BREAKFAST CEREAL UNFORTIFIED eg DOVES FARM CHOC STARS	02.03.00	Breakfast cereals - Other breakfast cereals - low fibre	57100100	Cereal, ready-to-eat, NFS
640	02-07956			CHOCOLATE AND CANDY COVERED NUTS	24.01.00	Confectionary - Chocolate based products	91700500	M&M's Almond Chocolate Candies
641	02-08016			CHOCOLATE BROWNIE NO NUTS PURCHASED	04.02.00	Sweet cereal products - Pastries, Buns & Pies	53204000	Cookie, brownie, NS as to icing
642	02-08015			CHOCOLATE BROWNIE WITH PECAN NUTS PURCHASED	04.02.00	Sweet cereal products - Pastries, Buns & Pies	53204000	Cookie, brownie, NS as to icing
643	02-08161			CHOCOLATE CAKE BAR WITH CHOCOLATE CHIPS PURCHASED	04.02.00	Sweet cereal products - Pastries, Buns & Pies	53108200	Snack cake, chocolate, with icing or filling
644	02-09401			CHOCOLATE CAKE COVERING	24.01.00	Confectionary - Chocolate based products	91305010	Icing, chocolate
645	02-03082			CHOCOLATE CAKE NO FILLING OR ICING, PURCHASED	04.02.00	Sweet cereal products - Pastries, Buns & Pies	53105275	Cake or cupcake, chocolate, devil's food or fudge, without icing or filling

646	02-10273			CHOCOLATE CHIP BRIOCHE ROLLS PURCHASED	04.02.00	Sweet cereal products - Pastries, Buns & Pies	51167000	Brioche
647	02-10065			CHOCOLATE CHIP COOKIES AND BISCUITS, REDUCED FAT	04.01.00	Sweet cereal products - Biscuits	53206030	Cookie, chocolate chip, reduced fat
648	02-05201			CHOCOLATE COATED CAKE BARS, INDIVIDUAL, PURCHASED	04.02.00	Sweet cereal products - Pastries, Buns & Pies	53108200	Snack cake, chocolate, with icing or filling
649	02-00309			CHOCOLATE CUP CAKES HOMEMADE	04.02.00	Sweet cereal products - Pastries, Buns & Pies	53105275	Cake or cupcake, chocolate, devil's food or fudge, without icing or filling
650	02-00254			CHOCOLATE SHORT OR SWEET BISCUITS HALF COATED	04.01.00	Sweet cereal products - Biscuits	53244010	Cookie, butter or sugar, with chocolate icing or filling
651	02-10307			CHOCOLATE SPONGE/SWISS ROLL WITH FRESH CREAM FILLING AND CHOCOLATE SAUCE	04.02.00	Sweet cereal products - Pastries, Buns & Pies	53108200	Snack cake, chocolate, with icing or filling
652	02-04108			CHOPPED HAM AND PORK WITH EGG	12.01.00	Processed meat - Bacon & ham	25230530	Ham and pork, canned luncheon meat, chopped, minced, pressed, spiced
653	02-08131			CIABATTA / PANINI TOASTED	03.04.00	Breads - Other bread	51109010	Bread, Italian, Grecian, Armenian
654	02-08453			CITRUS/PINEAPPLE DRINK RTD NOT LOW CALORIE	27.02.03	Beverages - Fruit based drinks - Squashes & fruit concentrates	91301050	Fruit syrup
655	02-08304			CLEAR MINTS	24.02.00	Confectionary - Sugar based products	91700010	Candy, NFS
656	02-01115			COATED CHICKEN PIECES TAKEAWAY	11.01.00	Meat - white - Chicken & turkey & dishes	24107070	Chicken, NS as to part, fried, coated, skin / coating eaten

657	02-04338			COCK-A-LEEKIE SOUP CANNED	20.01.00	Soups - Canned & fresh & homemade	58403010	Chicken or turkey noodle soup, canned or ready-to-serve
658	02-07651			COCONUT COOKIES AND BISCUITS	04.01.00	Sweet cereal products - Biscuits	53215500	Cookie, coconut
659	02-10319			COCONUT MACAROONS PURCHASED	04.01.00	Sweet cereal products - Biscuits	53215500	Cookie, coconut
660	02-08078			COCONUT MILK PURCHASED	19.00.00	Nuts & Seeds (incl. peanut butter)	42401010	Coconut milk, used in cooking
661	02-07208			COCONUT MILK REDUCED FAT PURCHASED	19.00.00	Nuts & Seeds (incl. peanut butter)	42401010	Coconut milk, used in cooking
662	02-01446			COD DRIED SALTED BOILED	09.01.00	Fish & fish dishes - White fish, incl. tuna	26109180	Cod, dried, salted, salt removed in water
663	02-03412			COD/HADDOCK COATED IN BREADCRUMBS, REDUCED FAT, GRILLED	09.01.00	Fish & fish dishes - White fish, incl. tuna	26109133	Cod, coated, baked or broiled, made without fat
664	02-02307			COFFEE FRESH STRONG INFUSION	27.06.00	Beverages - Coffee	92100000	Coffee, NS as to type
665	02-08313			COFFEE NOT STRONG INFUSION DECAFFEINATED	27.06.00	Beverages - Coffee	92100000	Coffee, NS as to type
666	02-08312			COFFEE STRONG INFUSION DECAFFEINATED	27.06.00	Beverages - Coffee	92100000	Coffee, NS as to type
667	02-02462			CONSOMME (OTHER CLEAR SOUPS; BOUILLON CUBES)	20.01.00	Soups - Canned & fresh & homemade	28310110	Beef, broth, bouillon, or consomme
668	02-09393			COOK IN SAUCE, CHINESE, SWEET AND SOUR CANNED	21.02.00	Sauces & accompaniment - Cooking sauces, incl. gravies, pesto, cooking sauces for pasta and rice dishes	91361010	Sweet and sour sauce
669	02-03981			COOK IN SAUCE, INDIAN, KORMA/TIKKA MASALA ONLY, NOT CANNED	21.02.00	Sauces & accompaniment - Cooking sauces, incl. gravies, pesto, cooking sauces for pasta and rice dishes	75440600	Vegetable curry

670	02-02420			COOK IN SAUCE, INDIAN, OTHER, NOT CANNED	21.02.00	Sauces & accompaniment - Cooking sauces, incl. gravies, pesto, cooking sauces for pasta and rice dishes	75440600	Vegetable curry
671	02-03984			COOK IN SAUCE, TOMATO BASED, NOT CANNED	21.02.00	Sauces & accompaniment - Cooking sauces, incl. gravies, pesto, cooking sauces for pasta and rice dishes	74404010	Spaghetti sauce
672	02-03930			COOK-IN-SAUCE, CURRY FLAVOUR, REDUCED FAT E.G. TESCO HEALTHY LIVING TIKKA SAUCE	21.02.00	Sauces & accompaniment - Cooking sauces, incl. gravies, pesto, cooking sauces for pasta and rice dishes	74404010	Spaghetti sauce
673	02-07663			COOKIES AND BISCUITS WITH CHOCOLATE AND NUTS	04.01.00	Sweet cereal products - Biscuits	53206020	Cookie, chocolate chip, made from home recipe or purchased at a bakery
674	02-06022			COOKIES AND BISCUITS WITH NUTS	04.01.00	Sweet cereal products - Biscuits	53206020	Cookie, chocolate chip, made from home recipe or purchased at a bakery
675	02-10061			COOKIES AND BISCUITS, NOT CHOCOLATE, NOT NUT, NOT SPECIFIED ELSEWHERE	04.01.00	Sweet cereal products - Biscuits	53201000	Cookie, NFS
676	00-09984	A-00-09984		COOKS WITH LO-SALT, MARKER DUMMY	26.02.00	Miscellaneous - Salt and salt substitutes	2047	Salt, table
677	00-09986	A-00-09986		COOKS WITH SALT, MARKER DUMMY	26.02.00	Miscellaneous - Salt and salt substitutes	2047	Salt, table
678	02-02627			CORN SNACKS EG MONSTER MUNCH WOTSITS	25.02.00	Savoury Snacks - Cereal based snacks	54401055	Cheese flavored corn snacks
679	02-06574			CORNED BEEF CRISPBAKE RETAIL EG. M&S	10.01.00	Meat - red - Beef & veal & dishes	21416000	Corned beef, cooked, NS as to fat eaten
680	02-01341			CORNED BEEF NOT CANNED	10.01.00	Meat - red - Beef & veal & dishes	21416000	Corned beef, cooked, NS as to fat eaten

681	02-08846			CORNED BEEF PASTY PURCHASED	12.02.00	Processed meat - Processed pies	58126110	Turnover, meat-filled, no gravy
682	02-10197			CORNFLAKE TYPE CEREALS FROSTED UNFORTIFIED	02.03.00	Breakfast cereals - Other breakfast cereals - low fibre	57348000	Cereal, frosted corn flakes
683	02-07726			COTTAGE CHEESE, LOW FAT WITH ADDITIONS	06.02.00	Dairy products - Cheese, incl. cottage cheese	14200100	Cheese, cottage, NFS
684	02-04283			COTTAGE PIE, LOW FAT, READY MEAL, EG WEIGHTWATCHERS	10.01.00	Meat - red - Beef & veal & dishes	27311510	Shepherd's pie with beef
685	02-08466			COUS COUS WITH ADDITIONS COOKED	01.04.00	Cereals & cereal dishes - Other cereals & dishes	58148115	Macaroni or pasta salad, made with light Italian dressing
686	02-04068			CRACKERBREAD, WHOLEMEAL, RYVITA	25.04.00	Savoury Snacks - Savoury biscuits & crackers	54305010	Crackers, crispbread
687	02-03895			CRANBERRY BASED JUICE DRINK; RTD, REDUCED SUGAR/NAS INC CRANBERRY AND ANOTHER JUICE	27.02.02	Beverages - Fruit based drinks - Fruit juice drinks	92531030	Fruit juice drink (Sunny D)
688	02-03846			CRANBERRY FRUIT JUICE DRINK EG OCEAN SPRAY	27.02.02	Beverages - Fruit based drinks - Fruit juice drinks	92531030	Fruit juice drink (Sunny D)
689	02-07696			CREAM DESSERTS CHOC/CARAMEL NOT FRUIT CONTAINING	06.04.02	Dairy products - Ice cream & dairy desserts - reduced fat products	13110110	Ice cream, regular, chocolate
690	02-02464			CREAM OF CHICKEN SOUP CONDENSED CANNED	20.01.00	Soups - Canned & fresh & homemade	28345110	Chicken or turkey soup, cream of, NS as to prepared with milk or water
691	02-10242			CREAM OF MUSHROOM SOUP CONDENSED, NOT MADE UP	20.01.00	Soups - Canned & fresh & homemade	6043	Soup, cream of mushroom, canned, condensed
692	02-02709			CREME BRULEE	06.04.01	Dairy products - Ice cream & dairy desserts - full fat products	13210350	Flan

693	02-07653			CRISPBREAD RYE WITH SESAMESEEDS	03.05.00	Breads - Crisp Breads, e.g. Rivetas, Grissini, Toast Melba	54305010	Crackers, crispbread
694	02-08120			CRISPBREADS WHOLEGRAIN AND SEEDED	03.05.00	Breads - Crisp Breads, e.g. Rivetas, Grissini, Toast Melba	54305010	Crackers, crispbread
695	02-08056			CRISPY SEAWEED; PURCHASED OR TAKEAWAY	15.04.00	Vegetables - Other	72118200	Greens, cooked, NS as to form, NS as to fat added in cooking
696	02-08366			CROISSANT WITH A SAVOURY FILLING	04.02.00	Sweet cereal products - Pastries, Buns & Pies	51166100	Croissant, cheese
697	02-08176			CROISSANT WITH SWEET FILLING	04.02.00	Sweet cereal products - Pastries, Buns & Pies	51166200	Croissant, chocolate
698	02-07963			CRUNCHIE BAR	24.01.00	Confectionary - Chocolate based products	91705420	Chocolate, white, with cereal
699	02-00213			CRUNCHY CLUSTERS TYPE CEREAL WITHOUT NUTS	02.01.00	Breakfast cereals - Oat based cereals	57316380	Cereal (General Mills Cheerios Oat Cluster Crunch)
700	02-08086			CRUNCHY NUT CLUSTERS KELLOGGS	02.03.00	Breakfast cereals - Other breakfast cereals - low fibre	57100100	Cereal, ready-to-eat, NFS
701	02-10513			CRUNCHY NUT CORNFLAKES OWN BRAND, NOT KELLOGGS	02.03.00	Breakfast cereals - Other breakfast cereals - low fibre	57239100	Cereal (Kellogg's Honey Crunch Corn Flakes)
702	02-03546			CRUNCHY RICE AND WHEAT FLAKES CEREAL	02.03.00	Breakfast cereals - Other breakfast cereals - low fibre	57100100	Cereal, ready-to-eat, NFS
703	02-05328			CRUNCHY/CRISPY MUESLI TYPE CEREAL WITH NUTS	02.01.00	Breakfast cereals - Oat based cereals	57308190	Cereal, muesli
704	02-10595			CRYSTALLISED GINGER	24.02.00	Confectionary - Sugar based products	91700010	Candy, NFS
705	02-07687			CUPCAKES PURCHASED, ANY FLAVOUR	04.02.00	Sweet cereal products - Pastries, Buns & Pies	53100100	Cake or cupcake, NS as to type
706	02-05588			CURRIED CHICK PEA WITH SPICES ONIONS & TOMS	16.01.00	Pulses/Lentils - Pulses/lentils	41311020	Sambar, vegetable stew
707	02-03355			CUSTARD MADE WITH SOYA MILK, UNSWEETENED	04.04.00	Sweet cereal products - Milk based puddings	13210300	Custard

708	02-00386			CUSTARD SLICE / VANILLA SLICE, PURCHASED	04.02.00	Sweet cereal products - Pastries, Buns & Pies	53344070	Pie, custard, individual size or tart
709	00-09786	A-00-09786		Ca Fortified Milk, skimmed, Vitapin	05.01.00	Milk - Skimmed milk	11100000	Milk, NFS
710	00-03572			Cabbage, January King, boiled, unsalted	15.02.00	Vegetables - Brassicacea	75211010	Cabbage, green, cooked, NS as to fat added in cooking
711	A-00-01165			Cabbage, Red, Cooked	15.02.00	Vegetables - Brassicacea	75212000	Cabbage, red, cooked, NS as to fat added in cooking
712	A-00-00580			Cabbage, Red, Raw	15.02.00	Vegetables - Brassicacea	75105000	Cabbage, red, raw
713	A-00-00582			Cabbage, Savoy, Boiled	15.02.00	Vegetables - Brassicacea	75213000	Cabbage, savoy, cooked, NS as to fat added in cooking
714	A-00-00581			Cabbage, Savoy, Raw	15.02.00	Vegetables - Brassicacea	75105000	Cabbage, red, raw
715	00-03574	A-00-03574		Cabbage, Savoy, boiled, unsalted water	15.02.00	Vegetables - Brassicacea	75213000	Cabbage, savoy, cooked, NS as to fat added in cooking
716	A-00-00583			Cabbage, Spring, Boiled	15.02.00	Vegetables - Brassicacea	75211010	Cabbage, green, cooked, NS as to fat added in cooking
717	A-00-00584			Cabbage, White, Raw	15.02.00	Vegetables - Brassicacea	75105000	Cabbage, red, raw
718	A-00-00586			Cabbage, Winter, Boiled	15.02.00	Vegetables - Brassicacea	75211010	Cabbage, green, cooked, NS as to fat added in cooking
719	A-00-00585			Cabbage, Winter, Raw	15.02.00	Vegetables - Brassicacea	75105000	Cabbage, red, raw
720	13-0185			Cabbage, boiled in unsalted water, average	15.02.00	Vegetables - Brassicacea	75211010	Cabbage, green, cooked, NS as to fat added in cooking
721	A-00-09915			Cabbage, boiled in unsalted water, average (MW6 carq; MW6 folate)	15.02.00	Vegetables - Brassicacea	75211010	Cabbage, green, cooked, NS as to fat added in cooking
722	13-0183			Cabbage, raw, average	15.02.00	Vegetables - Brassicacea	75105000	Cabbage, red, raw
723	A-00-09917			Cabbage, raw, average (MW6 carq)	15.02.00	Vegetables - Brassicacea	75105000	Cabbage, red, raw

724	00-03573	A-00-03573		Cabbage, red boiled unsalted water	15.02.00	Vegetables - Brassicacea	75212000	Cabbage, red, cooked, NS as to fat added in cooking
725	13-0190	A-13-0190		Cabbage, red, raw	15.02.00	Vegetables - Brassicacea	75105000	Cabbage, red, raw
726	13-0196			Cabbage, white, raw	15.02.00	Vegetables - Brassicacea	75105000	Cabbage, red, raw
727	A-00-09916			Cabbage, white, raw (MW6 carq)	15.02.00	Vegetables - Brassicacea	75105000	Cabbage, red, raw
728	00-03602	A-00-03602		Caesar salad	15.04.00	Vegetables - Other	72116150	Caesar salad, with romaine, no dressing
729	00-03849	A-00-03849		Caffeine free low calorie coke	27.03.00	Beverages - Carbonated soft drinks	92400000	Soft drink, NFS
730	A-00-06007			Cake 50 economical	04.02.00	Sweet cereal products - Pastries, Buns & Pies	53100100	Cake or cupcake, NS as to type
731	A-11-0192			Cake mix, made up	04.02.00	Sweet cereal products - Pastries, Buns & Pies	53100100	Cake or cupcake, NS as to type
732	A-12-0024			Calcium-fortified milk, Calcia	05.08.00	Milk - 1% milk	11100000	Milk, NFS
733	A-00-03386	00-03386		Cambazola	06.02.00	Dairy products - Cheese, incl. cottage cheese	14010000	Cheese, NFS
734	A-12-0081			Cambridge Diet powder, made up with water	30.00.00	Nutrition Powders & drinks	95120000	Nutritional drink or shake, ready-to-drink, NFS
735	A-00-03364	00-03364		Camembert in crumbs + cranberry sauce (Tesco)	06.02.00	Dairy products - Cheese, incl. cottage cheese	14202010	Cheese, cottage, with fruit
736	19-0184	A-19-0184		Cannelloni, chilled/frozen, reheated	01.02.00	Cereals & cereal dishes - Pasta & pasta dishes	58134810	Cannelloni, cheese- and spinach-filled, no sauce
737	00-03162			Caramel Shortcakes - squares	04.02.00	Sweet cereal products - Pastries, Buns & Pies	52105100	Scone
738	00-03615	A-00-03615		Carbonara sauce with pasta	01.02.00	Cereals & cereal dishes - Pasta & pasta dishes	58145110	Macaroni or noodles with cheese
739	A-00-01308			Carbonated Soft Drinks 'pop'	27.03.00	Beverages - Carbonated soft drinks	92400000	Soft drink, NFS
740	A-00-03806			Carnation Slender (reconstituted) 82/1449	05.03.00	Milk - Whole milk	95120000	Nutritional drink or shake, ready-to-drink, NFS

741	00-05338			Carr Cheese Melts	25.04.00	Savoury Snacks - Savoury biscuits & crackers	54304150	Crackers, cheese, whole grain
742	00-03936			Carrot & Coriander soup	20.01.00	Soups - Canned & fresh & homemade	75317011	Vegetables, stew type, cooked, made with oil
743	17-0249	A-17-0249		Carrot and orange soup	20.01.00	Soups - Canned & fresh & homemade	73105010	Carrot juice, 100%
744	00-09606	A-00-09606		Carrot cake	04.02.00	Sweet cereal products - Pastries, Buns & Pies	53104260	Cake or cupcake, carrot, with icing or filling
745	13-0199	A-13-0199		Carrot juice	27.02.01	Beverages - Fruit based drinks - Pure fruit juice & smoothies	61210000	Orange juice, 100%, NFS
746	A-00-00590			Carrots, Canned	15.03.00	Vegetables - Yellow & red & dark green leafy vegetables	73102203	Carrots, cooked, from canned, NS as to fat added in cooking
747	A-00-00588			Carrots, Old, Boiled	15.03.00	Vegetables - Yellow & red & dark green leafy vegetables	73102211	Carrots, cooked, from fresh, fat not added in cooking
748	A-00-00587			Carrots, Old, Raw,	15.03.00	Vegetables - Yellow & red & dark green leafy vegetables	73101010	Carrots, raw
749	A-00-00589			Carrots, Young,Boiled	15.03.00	Vegetables - Yellow & red & dark green leafy vegetables	73102211	Carrots, cooked, from fresh, fat not added in cooking
750	13-0207			Carrots, canned, re-heated, drained	15.04.00	Vegetables - Other	73102200	Carrots, cooked, NS as to form, NS as to fat added in cooking
751	A-00-09905			Carrots, canned, re-heated, drained (MW6 carq)	15.04.00	Vegetables - Other	73102200	Carrots, cooked, NS as to form, NS as to fat added in cooking
752	13-0206	A-13-0206		Carrots, frozen, boiled in unsalted water	15.04.00	Vegetables - Other	73102200	Carrots, cooked, NS as to form, NS as to fat added in cooking
753	13-0202			Carrots, old, boiled in unsalted water	15.04.00	Vegetables - Other	73102200	Carrots, cooked, NS as to form, NS as to fat added in cooking

754	A-00-09902			Carrots, old, boiled in unsalted water (MW6 carq)	15.04.00	Vegetables - Other	73102200	Carrots, cooked, NS as to form, NS as to fat added in cooking
755	13-0200			Carrots, old, raw	15.04.00	Vegetables - Other	73101010	Carrots, raw
756	A-00-09901			Carrots, old, raw (MW6 carq)	15.04.00	Vegetables - Other	73101010	Carrots, raw
757	13-0205			Carrots, young, boiled in unsalted water	15.04.00	Vegetables - Other	73102200	Carrots, cooked, NS as to form, NS as to fat added in cooking
758	A-00-09904			Carrots, young, boiled in unsalted water (MW6 carq)	15.04.00	Vegetables - Other	73102200	Carrots, cooked, NS as to form, NS as to fat added in cooking
759	13-0203			Carrots, young, raw	15.04.00	Vegetables - Other	73101010	Carrots, raw
760	A-00-09903			Carrots, young, raw (MW6 carq)	15.04.00	Vegetables - Other	73101010	Carrots, raw
761	A-14-0811	14-0811		Cashew nuts, plain	19.00.00	Nuts & Seeds (incl. peanut butter)	42104000	Cashews, NFS
762	14-0812	A-14-0812		Cashew nuts, roasted and salted	19.00.00	Nuts & Seeds (incl. peanut butter)	42104000	Cashews, NFS
763	13-0214			Cassava chips	25.03.00	Savoury Snacks - Vegetable based snacks	71980200	Taro chips
764	00-05878			Casserole cooked in specified fat	15.04.00	Vegetables - Other	41202500	Beans and tomatoes, NS as to fat added in cooking
765	15-0060			Casserole, bean and mixed vegetable	15.04.00	Vegetables - Other	41202500	Beans and tomatoes, NS as to fat added in cooking
766	15-0061	A-15-0061		Casserole, bean and root vegetable	15.04.00	Vegetables - Other	41202500	Beans and tomatoes, NS as to fat added in cooking
767	16-0008			Catfish, steamed	09.01.00	Fish & fish dishes - White fish, incl. tuna	26107160	Catfish, steamed or poached
768	A-00-01268			Cauliflower Bhajia with Butter	15.02.00	Vegetables - Brassicacea	75214000	Cauliflower, cooked, NS as to form, NS as to fat added in cooking

769	A-00-01269			Cauliflower Bhajia with Vegetable Ghee	15.02.00	Vegetables - Brassicacea	75214000	Cauliflower, cooked, NS as to form, NS as to fat added in cooking
770	A-00-00174			Cauliflower Cheese	15.02.00	Vegetables - Brassicacea	75409011	Cauliflower, from fresh, creamed
771	12-0264	A-12-0264		Cauliflower cheese	15.02.00	Vegetables - Brassicacea	75409011	Cauliflower, from fresh, creamed
772	A-00-01104			Cauliflower in White Sauce	15.02.00	Vegetables - Brassicacea	75409011	Cauliflower, from fresh, creamed
773	00-09847	A-00-09847		Cauliflower in white sauce, semi-skimmed milk	15.02.00	Vegetables - Brassicacea	75409011	Cauliflower, from fresh, creamed
774	00-09850	A-00-09850		Cauliflower in white sauce, skimmed milk	15.02.00	Vegetables - Brassicacea	75409011	Cauliflower, from fresh, creamed
775	A-00-00592			Cauliflower, Boiled	15.02.00	Vegetables - Brassicacea	75214000	Cauliflower, cooked, NS as to form, NS as to fat added in cooking
776	A-00-00591			Cauliflower, Raw	15.02.00	Vegetables - Brassicacea	75107000	Cauliflower, raw
777	A-13-0217	13-0217		Cauliflower, boiled in unsalted water	15.02.00	Vegetables - Brassicacea	75214000	Cauliflower, cooked, NS as to form, NS as to fat added in cooking
778	13-0218			Cauliflower, frozen, boiled in unsalted water	15.02.00	Vegetables - Brassicacea	75214000	Cauliflower, cooked, NS as to form, NS as to fat added in cooking
779	13-0215	A-13-0215		Cauliflower, raw	15.02.00	Vegetables - Brassicacea	75107000	Cauliflower, raw
780	16-0272			Caviare, bottled in brine, drained	09.02.00	Fish & fish dishes - Oily fish	26211100	Roe, sturgeon
781	A-00-00593			Celeriac, Boiled	15.04.00	Vegetables - Other	71961010	Celeriac, cooked
782	00-03578	A-00-03578		Celeriac, boiled unsalted water	15.04.00	Vegetables - Other	71961010	Celeriac, cooked
783	A-00-00595			Celery, Boiled	15.04.00	Vegetables - Other	75215000	Celery, cooked, NS as to fat added in cooking
784	A-00-00594			Celery, Raw	15.04.00	Vegetables - Other	75109000	Celery, raw
785	A-00-03575	00-03575		Celery, boiled, unsalted water	15.04.00	Vegetables - Other	75215000	Celery, cooked, NS as to fat added in cooking

786	A-13-0221	13-0221		Celery, raw	15.04.00	Vegetables - Other	75109000	Celery, raw
787	00-05870			Cereal bar containing oats and fruit, fortified eg Sainsburys	04.05.00	Sweet cereal products - Cereal bars	53710500	Cereal or granola bar (Kellogg's Nutri-Grain Cereal Bar)
788	00-03156			Cereal bar reduced fat	04.05.00	Sweet cereal products - Cereal bars	53712200	Cereal or granola bar, lowfat, NFS
789	17-0102	A-17-0102		Cereal chewy bar	04.05.00	Sweet cereal products - Cereal bars	53710800	Cereal or granola bar (Kashi Chewy)
790	A-00-03137	00-03137		Cereal chewy bar with yoghurt	04.05.00	Sweet cereal products - Cereal bars	53710902	Cereal or granola bar, with yogurt coating (General Mills Nature Valley Chewy Granola Bar)
791	A-17-0103	17-0103		Cereal crunchy bar	04.05.00	Sweet cereal products - Cereal bars	53710802	Cereal or granola bar (Kashi Crunchy)
792	A-17-0226	17-0226		Champagne	27.01.01	Beverages - Alcohol - Wine	93401010	Wine, table, red
793	A-00-01285			Channa Dahl	16.01.00	Pulses/Lentils - Pulses/lentils	41101000	Beans, dry, cooked, NS as to type and as to fat added in cooking
794	12-0019	A-12-0019		Channel Island milk, whole, pasteurised, summer	05.03.00	Milk - Whole milk	11100000	Milk, NFS
795	12-0020	A-12-0020		Channel Island milk, whole, pasteurised, winter	05.03.00	Milk - Whole milk	11100000	Milk, NFS
796	A-00-01236			Chapatis, Maize Flour	03.04.00	Breads - Other bread	52215100	Tortilla, corn
797	A-00-00045			Chapatis, made with Fat	03.04.00	Breads - Other bread	52215200	Tortilla, flour
798	11-0074	A-11-0074		Chapatis, made with fat	03.04.00	Breads - Other bread	52215200	Tortilla, flour
799	A-00-00046			Chapatis, made without Fat	03.04.00	Breads - Other bread	52215200	Tortilla, flour
800	11-0075			Chapatis, made without fat	03.04.00	Breads - Other bread	52215200	Tortilla, flour
801	A-00-03008	00-03008		Cheerios, Nestle	02.03.00	Breakfast cereals - Other breakfast cereals - low fibre	57123000	Cereal (General Mills Cheerios)
802	00-05513			Cheerios-Honey nut	02.03.00	Breakfast cereals - Other breakfast cereals - low fibre	57241000	Cereal (General Mills Cheerios Honey Nut)

803	00-03389			Cheese & Chive dip (eg Tesco)	21.03.00	Sauces & accompaniment - Other sauces, incl. brown sauce, soy sauce, ketchup, mint sauce, vinegar	14620200	Cheese dip
804	00-05771			Cheese Coleslaw, Purchased	15.04.00	Vegetables - Other	75140990	Cabbage salad or coleslaw, from fast food / restaurant
805	A-00-03763			Cheese Crackers (Cheddars) 82/1402	25.04.00	Savoury Snacks - Savoury biscuits & crackers	54304000	Crackers, cheese
806	A-00-01064			Cheese Omelette	07.00.00	Egg & egg dishes	32130120	Egg omelet or scrambled egg, with cheese, made with butter
807	A-00-03766			Cheese Pancake (B.E.) fried 82/1405	01.04.00	Cereals & cereal dishes - Other cereals & dishes	58124210	Pastry, cheese-filled
808	A-00-01066			Cheese Pastry	06.02.00	Dairy products - Cheese, incl. cottage cheese	53452450	Cheese pastry puffs
809	A-00-00175			Cheese Pudding	06.02.00	Dairy products - Cheese, incl. cottage cheese	14630200	Cheese souffle
810	A-00-00922			Cheese Sauce	21.03.00	Sauces & accompaniment - Other sauces, incl. brown sauce, soy sauce, ketchup, mint sauce, vinegar	14650100	Cheese sauce
811	A-00-01067			Cheese Scones	01.04.00	Cereals & cereal dishes - Other cereals & dishes	52104100	Biscuit, cheese
812	A-00-00176			Cheese Souffle	07.00.00	Egg & egg dishes	14630200	Cheese souffle
813	A-00-00160			Cheese Spread	06.02.00	Dairy products - Cheese, incl. cottage cheese	14420200	Cheese spread, cream cheese, regular
814	A-00-01063			Cheese and Egg Flan	07.00.00	Egg & egg dishes	14630200	Cheese souffle
815	A-00-03330	00-03330		Cheese and Onion Bread	03.04.00	Breads - Other bread	51111010	Bread, cheese
816	00-03612	A-00-03612		Cheese and Onion pastie (pasty)	01.04.00	Cereals & cereal dishes - Other cereals & dishes	58127150	Vegetables and cheese in pastry
817	A-00-01065			Cheese and Potato Pie	06.02.00	Dairy products - Cheese, incl. cottage cheese	71501011	Potato, mashed, from fresh, made with milk, with cheese

818	00-09690	A-00-09690		Cheese and onion flaky pastry roll	01.04.00	Cereals & cereal dishes - Other cereals & dishes	58127150	Vegetables and cheese in pastry
819	A-12-0265	12-0265		Cheese and potato pie	06.02.00	Dairy products - Cheese, incl. cottage cheese	71501011	Potato, mashed, from fresh, made with milk, with cheese
820	A-00-01393	00-01393		Cheese biscuits, Cheddars	25.04.00	Savoury Snacks - Savoury biscuits & crackers	54304000	Crackers, cheese
821	A-11-0219	11-0219		Cheese pastry, cooked	01.04.00	Cereals & cereal dishes - Other cereals & dishes	58124210	Pastry, cheese-filled
822	A-12-0267			Cheese pudding	06.02.00	Dairy products - Cheese, incl. cottage cheese	14630200	Cheese souffle
823	12-0272			Cheese sauce packet mix, made up with whole milk	21.02.00	Sauces & accompaniment - Cooking sauces, incl. gravies, pesto, cooking sauces for pasta and rice dishes	14650100	Cheese sauce
824	A-12-0269			Cheese sauce, made with semi-skimmed milk	21.02.00	Sauces & accompaniment - Cooking sauces, incl. gravies, pesto, cooking sauces for pasta and rice dishes	14650100	Cheese sauce
825	A-12-0270			Cheese sauce, made with skimmed milk	21.02.00	Sauces & accompaniment - Cooking sauces, incl. gravies, pesto, cooking sauces for pasta and rice dishes	14650100	Cheese sauce
826	A-12-0268	00-09830	A-00-09830	Cheese sauce, made with whole milk	21.02.00	Sauces & accompaniment - Cooking sauces, incl. gravies, pesto, cooking sauces for pasta and rice dishes	14650100	Cheese sauce
827	00-09835	A-00-09835		Cheese sauce, pkt mix, with semi-skimmed milk	21.02.00	Sauces & accompaniment - Cooking sauces, incl. gravies, pesto, cooking sauces for pasta and rice dishes	14650100	Cheese sauce

828	00-09834			Cheese sauce, pkt mix, with whole milk	21.02.00	Sauces & accompaniment - Cooking sauces, incl. gravies, pesto, cooking sauces for pasta and rice dishes	14650100	Cheese sauce
829	A-00-09831	00-09831		Cheese sauce,with semi-skimmed milk	21.02.00	Sauces & accompaniment - Cooking sauces, incl. gravies, pesto, cooking sauces for pasta and rice dishes	14650100	Cheese sauce
830	A-00-09832	00-09832		Cheese sauce,with skimmed milk	21.02.00	Sauces & accompaniment - Cooking sauces, incl. gravies, pesto, cooking sauces for pasta and rice dishes	14650100	Cheese sauce
831	12-0143	A-12-0143		Cheese spread, flavoured	06.02.00	Dairy products - Cheese, incl. cottage cheese	14420200	Cheese spread, cream cheese, regular
832	A-12-0142	A-00-09722		Cheese spread, plain	06.02.00	Dairy products - Cheese, incl. cottage cheese	14420200	Cheese spread, cream cheese, regular
833	00-09722			Cheese spread, plain NOT dairylea	06.02.00	Dairy products - Cheese, incl. cottage cheese	14420200	Cheese spread, cream cheese, regular
834	A-00-09726			Cheese spread, reduced fat	06.02.00	Dairy products - Cheese, incl. cottage cheese	14420200	Cheese spread, cream cheese, regular
835	00-09726			Cheese spread, reduced fat NOT dairylea	06.02.00	Dairy products - Cheese, incl. cottage cheese	14420200	Cheese spread, cream cheese, regular
836	00-05781			Cheese spreads, triangles, plain, low fat, Dairylea ONLY	06.02.00	Dairy products - Cheese, incl. cottage cheese	14420200	Cheese spread, cream cheese, regular
837	00-09568	A-00-09568		Cheese topped white rolls	03.01.00	Breads - White	51111010	Bread, cheese
838	A-12-0131			Cheese, Brie	06.02.00	Dairy products - Cheese, incl. cottage cheese	14103020	Cheese, Brie
839	00-09735	A-00-09735		Cheese, Brie, including rind	06.02.00	Dairy products - Cheese, incl. cottage cheese	14103020	Cheese, Brie
840	12-0132	A-12-0132		Cheese, Caerphilly	06.02.00	Dairy products - Cheese, incl. cottage cheese	14010000	Cheese, NFS
841	A-00-09710	00-09710	A-12-0133	Cheese, Camembert	06.02.00	Dairy products - Cheese, incl. cottage cheese	14103010	Cheese, Camembert

842	A-00-00151			Cheese, Camembert Type	06.02.00	Dairy products - Cheese, incl. cottage cheese	14103010	Cheese, Camembert
843	A-00-00152			Cheese, Cheddar Type	06.02.00	Dairy products - Cheese, incl. cottage cheese	14104100	Cheese, Cheddar
844	00-09700			Cheese, Cheddar type, half fat	06.02.00	Dairy products - Cheese, incl. cottage cheese	14104100	Cheese, Cheddar
845	A-00-09700			Cheese, Cheddar type, reduced fat	06.02.00	Dairy products - Cheese, incl. cottage cheese	14104100	Cheese, Cheddar
846	00-09698	A-12-0134	A-00-09698	Cheese, Cheddar, average	06.02.00	Dairy products - Cheese, incl. cottage cheese	14104100	Cheese, Cheddar
847	A-12-0140	00-09699	A-00-09699	Cheese, Cheddar, vegetarian	06.02.00	Dairy products - Cheese, incl. cottage cheese	14104100	Cheese, Cheddar
848	A-12-0141			Cheese, Cheddar-type, reduced fat	06.02.00	Dairy products - Cheese, incl. cottage cheese	14104100	Cheese, Cheddar
849	A-12-0145	00-09702	A-00-09702	Cheese, Cheshire	06.02.00	Dairy products - Cheese, incl. cottage cheese	14010000	Cheese, NFS
850	A-12-0146			Cheese, Cheshire-type, reduced fat	06.02.00	Dairy products - Cheese, incl. cottage cheese	14010000	Cheese, NFS
851	A-00-00157			Cheese, Cottage	06.02.00	Dairy products - Cheese, incl. cottage cheese	14200100	Cheese, cottage, NFS
852	A-00-01330			Cheese, Cottage, with Fruits	06.02.00	Dairy products - Cheese, incl. cottage cheese	14202010	Cheese, cottage, with fruit
853	A-00-01331			Cheese, Cottage, with Vegetables	06.02.00	Dairy products - Cheese, incl. cottage cheese	14202020	Cheese, cottage, with vegetables
854	A-00-01332			Cheese, Cottage, with nuts/fish/Cheddar	06.02.00	Dairy products - Cheese, incl. cottage cheese	14610520	Cheese with nuts
855	A-00-00158			Cheese, Cream	06.02.00	Dairy products - Cheese, incl. cottage cheese	14420200	Cheese spread, cream cheese, regular
856	00-09716	A-00-09716		Cheese, Danish Blue	06.02.00	Dairy products - Cheese, incl. cottage cheese	14101010	Cheese, Blue or Roquefort
857	A-00-00153			Cheese, Danish Blue Type	06.02.00	Dairy products - Cheese, incl. cottage cheese	14101010	Cheese, Blue or Roquefort
858	A-12-0151			Cheese, Danish blue	06.02.00	Dairy products - Cheese, incl. cottage cheese	14101010	Cheese, Blue or Roquefort

859	12-0152	A-12-0152		Cheese, Derby	06.02.00	Dairy products - Cheese, incl. cottage cheese	14010000	Cheese, NFS
860	00-09703	A-12-0153	A-00-09703	Cheese, Double Gloucester	06.02.00	Dairy products - Cheese, incl. cottage cheese	14010000	Cheese, NFS
861	00-09714	A-00-09714	A-12-0154	Cheese, Edam	06.02.00	Dairy products - Cheese, incl. cottage cheese	14105010	Cheese, Gouda or Edam
862	A-00-00154			Cheese, Edam Type	06.02.00	Dairy products - Cheese, incl. cottage cheese	14105010	Cheese, Gouda or Edam
863	A-12-0155	12-0155		Cheese, Edam-type, reduced fat	06.02.00	Dairy products - Cheese, incl. cottage cheese	14105010	Cheese, Gouda or Edam
864	00-09713	A-00-09713	A-12-0156	Cheese, Emmental	06.02.00	Dairy products - Cheese, incl. cottage cheese	14109010	Cheese, Swiss
865	A-12-0157	12-0157		Cheese, Feta	06.02.00	Dairy products - Cheese, incl. cottage cheese	14104400	Cheese, Feta
866	A-00-09715	00-09715	A-12-0163	Cheese, Gouda	06.02.00	Dairy products - Cheese, incl. cottage cheese	14105010	Cheese, Gouda or Edam
867	A-12-0164	12-0164		Cheese, Gruyere	06.02.00	Dairy products - Cheese, incl. cottage cheese	14105200	Cheese, Gruyere
868	00-09704	A-00-09704	A-12-0166	Cheese, Lancashire	06.02.00	Dairy products - Cheese, incl. cottage cheese	14010000	Cheese, NFS
869	A-12-0167			Cheese, Leicester	06.02.00	Dairy products - Cheese, incl. cottage cheese	14010000	Cheese, NFS
870	00-09701	A-00-09701		Cheese, Leicester, red	06.02.00	Dairy products - Cheese, incl. cottage cheese	14010000	Cheese, NFS
871	A-12-0168			Cheese, Lymeswold	06.02.00	Dairy products - Cheese, incl. cottage cheese	14101010	Cheese, Blue or Roquefort
872	A-00-09707			Cheese, Marscapone	06.02.00	Dairy products - Cheese, incl. cottage cheese	14420200	Cheese spread, cream cheese, regular
873	00-09707			Cheese, Mascapone	06.02.00	Dairy products - Cheese, incl. cottage cheese	14420200	Cheese spread, cream cheese, regular
874	A-12-0170	12-0170		Cheese, Mozzarella	06.02.00	Dairy products - Cheese, incl. cottage cheese	14107010	Cheese, Mozzarella, NFS
875	A-00-00155	12-0171	A-12-0171	Cheese, Parmesan	06.02.00	Dairy products - Cheese, incl. cottage cheese	14108020	Cheese, Parmesan, hard

876	00-09717	A-00-09717		Cheese, Parmesan, fresh	06.02.00	Dairy products - Cheese, incl. cottage cheese	14108020	Cheese, Parmesan, hard
877	A-00-09712	00-09712		Cheese, Port Salut, St Paulin	06.02.00	Dairy products - Cheese, incl. cottage cheese	14108200	Cheese, Port du Salut
878	A-00-00159			Cheese, Processed	06.02.00	Dairy products - Cheese, incl. cottage cheese	14410500	Cheese, processed cheese food
879	A-12-0175			Cheese, Red Windsor	06.02.00	Dairy products - Cheese, incl. cottage cheese	14010000	Cheese, NFS
880	12-0176	A-12-0176		Cheese, Ricotta	06.02.00	Dairy products - Cheese, incl. cottage cheese	14201500	Cheese, Ricotta
881	12-0177	A-12-0177		Cheese, Roquefort	06.02.00	Dairy products - Cheese, incl. cottage cheese	14101010	Cheese, Blue or Roquefort
882	A-00-01333			Cheese, Soft (40% Fat)	06.02.00	Dairy products - Cheese, incl. cottage cheese	14103020	Cheese, Brie
883	A-00-00156			Cheese, Stilton	06.02.00	Dairy products - Cheese, incl. cottage cheese	14101010	Cheese, Blue or Roquefort
884	00-09705	A-12-0180	A-00-09705	Cheese, Stilton, blue	06.02.00	Dairy products - Cheese, incl. cottage cheese	14101010	Cheese, Blue or Roquefort
885	A-12-0181			Cheese, Stilton, white	06.02.00	Dairy products - Cheese, incl. cottage cheese	14101010	Cheese, Blue or Roquefort
886	A-00-09706	A-12-0182	00-09706	Cheese, Wensleydale	06.02.00	Dairy products - Cheese, incl. cottage cheese	14103020	Cheese, Brie
887	19-0040	A-19-0040		Cheeseburger, takeaway	13.00.00	Sausages & burgers & kebab	27510155	Cheeseburger, NFS
888	A-00-00099	A-12-0218		Cheesecake	06.04.01	Dairy products - Ice cream & dairy desserts - full fat products	53104500	Cheesecake
889	A-12-0219			Cheesecake, frozen	06.04.02	Dairy products - Ice cream & dairy desserts - reduced fat products	53104500	Cheesecake
890	00-09615	A-00-09615		Cheesecake, fruit, large	06.04.01	Dairy products - Ice cream & dairy desserts - full fat products	53104550	Cheesecake with fruit
891	00-09616	A-00-09616		Cheesecake, fruit, low fat, individual	06.04.02	Dairy products - Ice cream & dairy desserts - reduced fat products	53104550	Cheesecake with fruit

892	00-09667	A-00-09667		Cheesecake, individual chilled	06.04.01	Dairy products - Ice cream & dairy desserts - full fat products	53104500	Cheesecake
893	00-09614	A-00-09614		Cheesecake, not fruit (choc, toffee)	06.04.01	Dairy products - Ice cream & dairy desserts - full fat products	53104600	Cheesecake, chocolate
894	A-11-0232	11-0232		Chelsea buns	04.02.00	Sweet cereal products - Pastries, Buns & Pies	51160000	Roll, sweet, no frosting
895	A-00-06067			Chelsea buns 50	04.02.00	Sweet cereal products - Pastries, Buns & Pies	51160000	Roll, sweet, no frosting
896	A-00-00702			Cherries, Cooking, Raw (Weighed with Stones)	18.01.00	Fruit - Fresh	63113010	Cherries, sour, red, raw
897	A-00-00705			Cherries, Cooking, Stewed with Sugar	18.02.00	Fruit - Canned & cooked	63115110	Cherries, sweet, cooked or canned, NS as to sweetened or unsweetened; sweetened, NS as to type of sweetener
898	A-00-00706			Cherries, Cooking, Stewed with Sugar (Weighed with Stones)	18.02.00	Fruit - Canned & cooked	63115110	Cherries, sweet, cooked or canned, NS as to sweetened or unsweetened; sweetened, NS as to type of sweetener
899	A-00-00703			Cherries, Cooking, Stewed without Sugar	18.02.00	Fruit - Canned & cooked	63115110	Cherries, sweet, cooked or canned, NS as to sweetened or unsweetened; sweetened, NS as to type of sweetener
900	A-00-00704			Cherries, Cooking, Stewed without Sugar (Weighed with Stones	18.02.00	Fruit - Canned & cooked	63115110	Cherries, sweet, cooked or canned, NS as to sweetened or unsweetened; sweetened, NS as to type of sweetener

901	A-00-00699			Cherries, Eating, Raw	18.01.00	Fruit - Fresh	63115010	Cherries, sweet, raw
902	A-00-00700			Cherries, Eating, Raw (Weighed with Stones)	18.01.00	Fruit - Fresh	63115010	Cherries, sweet, raw
903	A-00-00846			Cherries, Glace	18.03.00	Fruit - Dried	63111010	Cherries, maraschino
904	14-0067	A-14-0067		Cherries, canned in syrup	18.02.00	Fruit - Canned & cooked	63115140	Cherries, sweet, cooked or canned, in light syrup
905	14-0068	A-14-0068		Cherries, glace	18.03.00	Fruit - Dried	63111010	Cherries, maraschino
906	14-0061	A-14-0061		Cherries, raw	18.01.00	Fruit - Fresh	63115010	Cherries, sweet, raw
907	A-14-0063	14-0063		Cherries, stewed with sugar	18.02.00	Fruit - Canned & cooked	63115110	Cherries, sweet, cooked or canned, NS as to sweetened or unsweetened; sweetened, NS as to type of sweetener
908	A-00-00917			Cherry Brandy	27.01.04	Beverages - Alcohol - Spirits & Liqueur	93501000	Brandy
909	00-05645			Cherry Coke, cherry cola, NOT low calorie	27.03.00	Beverages - Carbonated soft drinks	92400000	Soft drink, NFS
910	A-11-0193	11-0193		Cherry cake	04.02.00	Sweet cereal products - Pastries, Buns & Pies	53102800	Cake or cupcake, Black Forest
911	A-00-06058			Cherry cake 50	04.02.00	Sweet cereal products - Pastries, Buns & Pies	53102800	Cake or cupcake, Black Forest
912	14-0070	A-14-0070		Cherry pie filling	18.02.00	Fruit - Canned & cooked	63113030	Cherry pie filling
913	A-14-0813	14-0813	A-00-00828	Chestnuts	19.00.00	Nuts & Seeds (incl. peanut butter)	42105000	Chestnuts
914	A-17-0104	17-0104		Chew sweets including Opal Fruits, Chewitts,	24.02.00	Confectionary - Sugar based products	91700010	Candy, NFS
915	13-0078	A-13-0078		Chick peas, canned, re-heated, drained	16.01.00	Pulses/Lentils - Pulses/lentils	41101000	Beans, dry, cooked, NS as to type and as to fat added in cooking
916	13-0075			Chick peas, whole, dried, boiled in unsalted water	16.01.00	Pulses/Lentils - Pulses/lentils	41101000	Beans, dry, cooked, NS as to type and as to fat added in cooking

917	A-00-03557			Chicken & brocolli crisp bakes M&S	11.01.00	Meat - white - Chicken & turkey & dishes	27443150	Chicken or turkey divan
918	A-00-06207			Chicken 50 boiled	11.01.00	Meat - white - Chicken & turkey & dishes	24100000	Chicken, NS as to part and cooking method, NS as to skin eaten
919	A-00-09506			Chicken Biriani, incl rice	11.01.00	Meat - white - Chicken & turkey & dishes	27243100	Biryani with chicken
920	00-03630			Chicken Breast in crumb (eg Birds Eye Lemon Pepper Chicken)	11.01.00	Meat - white - Chicken & turkey & dishes	24127500	Chicken breast, baked, coated, skin / coating eaten
921	A-00-03783			Chicken Broth 82/1425	20.01.00	Soups - Canned & fresh & homemade	28340110	Chicken or turkey broth, bouillon, or consomme
922	A-00-01084	00-01084		Chicken Casserole with Vegetables	11.01.00	Meat - white - Chicken & turkey & dishes	27345410	Chicken or turkey, rice, and vegetables including carrots, broccoli, and/or dark-green leafy; cream sauce, white sauce, or mushroom sauce
923	A-00-01290			Chicken Curry with Bone	11.01.00	Meat - white - Chicken & turkey & dishes	27146150	Chicken curry
924	A-00-01289			Chicken Curry without Bone	11.01.00	Meat - white - Chicken & turkey & dishes	27146150	Chicken curry
925	A-00-09535	00-09535		Chicken Curry, Green	11.01.00	Meat - white - Chicken & turkey & dishes	27146150	Chicken curry
926	A-00-09507	00-09507		Chicken Dhansak	11.01.00	Meat - white - Chicken & turkey & dishes	27146150	Chicken curry
927	A-00-09512			Chicken Dupiaza	11.01.00	Meat - white - Chicken & turkey & dishes	27146150	Chicken curry
928	A-00-03743	00-03743		Chicken Fat (100% fat) 82/984	08.03.00	Fats - Animal based fats (solid)	81201000	Animal fat or drippings
929	00-05355			Chicken Mulligatawny Soup	20.01.00	Soups - Canned & fresh & homemade	28340800	Chicken or turkey soup with vegetables and fruit, Asian Style

930	A-00-00942			Chicken Noodle Soup, Dried, as Served	20.01.00	Soups - Canned & fresh & homemade	58403010	Chicken or turkey noodle soup, canned or ready-to-serve
931	A-00-03764			Chicken Pancakes (B.E.) 82/1403	11.01.00	Meat - white - Chicken & turkey & dishes	27246300	Chicken or turkey cake, patty, or croquette
932	A-00-01091			Chicken Pie, 2 Crusts, Plate Pie	12.02.00	Processed meat - Processed pies	27347100	Chicken or turkey pot pie
933	A-00-01090			Chicken Pie, One Crust/ Vol Au Vent	12.02.00	Processed meat - Processed pies	27347100	Chicken or turkey pot pie
934	00-09536	A-00-09536		Chicken Satay	11.01.00	Meat - white - Chicken & turkey & dishes	24123310	Chicken breast, grilled with sauce, skin eaten
935	A-00-00939			Chicken Soup, Condensed	20.01.00	Soups - Canned & fresh & homemade	6149	Soup, chicken mushroom, canned, condensed
936	A-00-00940			Chicken Soup, Condensed, as Served	20.01.00	Soups - Canned & fresh & homemade	28340600	Chicken or turkey vegetable soup, canned, prepared with water or ready-to-serve
937	A-00-00938			Chicken Soup, Cream Of, Canned, Ready To Serve	20.01.00	Soups - Canned & fresh & homemade	28345110	Chicken or turkey soup, cream of, NS as to prepared with milk or water
938	00-03556	A-00-03556		Chicken Supreme, Birds Eye	11.01.00	Meat - white - Chicken & turkey & dishes	27143000	Chicken or turkey with cream sauce
939	A-00-09502	00-09502		Chicken Tikka	11.01.00	Meat - white - Chicken & turkey & dishes	27146150	Chicken curry
940	00-09503	A-00-09503		Chicken Tikka Masala	11.01.00	Meat - white - Chicken & turkey & dishes	27146150	Chicken curry
941	00-05435			Chicken Tonight. Honey And Mustard	21.02.00	Sauces & accompaniment - Cooking sauces, incl. gravies, pesto, cooking sauces for pasta and rice dishes	83105500	Honey mustard dressing

942	A-00-03796			Chicken and Mushroom Pie, individual (B.E.) 82/1438	12.02.00	Processed meat - Processed pies	27347100	Chicken or turkey pot pie
943	A-00-03526			Chicken and broccoli pasta, Snackpot	01.02.00	Cereals & cereal dishes - Pasta & pasta dishes	58146323	Pasta with tomato-based sauce and meat, ready-to-heat
944	19-0054			Chicken and mushroom pie, single crust, homemade	11.01.00	Meat - white - Chicken & turkey & dishes	27347100	Chicken or turkey pot pie
945	A-19-0118	19-0118		Chicken breast in crumbs, chilled, fried	11.01.00	Meat - white - Chicken & turkey & dishes	24127200	Chicken breast, fried, coated, skin / coating eaten, from raw
946	19-0041	A-19-0041		Chicken burger, takeway	13.00.00	Sausages & burgers & kebab	27545000	Turkey or chicken burger, plain, on bun, from fast food / restaurant
947	A-19-0186	19-0186		Chicken chasseur	11.01.00	Meat - white - Chicken & turkey & dishes	27144000	Chicken or turkey with mushroom sauce
948	A-19-0188	19-0188		Chicken curry, chilled/frozen, reheated	11.01.00	Meat - white - Chicken & turkey & dishes	27146150	Chicken curry
949	A-19-0189	19-0189		Chicken curry, chilled/frozen, reheated, with rice	11.01.00	Meat - white - Chicken & turkey & dishes	27146150	Chicken curry
950	19-0190			Chicken curry, made with canned curry sauce	11.01.00	Meat - white - Chicken & turkey & dishes	27146150	Chicken curry
951	A-00-03466	00-03466		Chicken dripping	08.03.00	Fats - Animal based fats (solid)	81201000	Animal fat or drippings
952	A-00-03554			Chicken en croute	11.01.00	Meat - white - Chicken & turkey & dishes	27146250	Chicken or turkey cordon bleu
953	00-09532	A-00-09532		Chicken fajitas, meat only	11.01.00	Meat - white - Chicken & turkey & dishes	24124200	Chicken breast, sauteed, skin eaten
954	19-0191			Chicken fricassee	11.01.00	Meat - white - Chicken & turkey & dishes	27142100	Chicken or turkey fricassee
955	A-00-09527	00-09527		Chicken fried rice	01.03.00	Cereals & cereal dishes - Rice & rice dishes	58150320	Rice, fried, with chicken

956	A-00-01085	00-01085		Chicken in White Sauce	11.01.00	Meat - white - Chicken & turkey & dishes	27143000	Chicken or turkey with cream sauce
957	A-19-0195	19-0195		Chicken in white sauce, made with whole milk	11.01.00	Meat - white - Chicken & turkey & dishes	27143000	Chicken or turkey with cream sauce
958	A-00-09511	00-09511		Chicken jalfrezi	11.01.00	Meat - white - Chicken & turkey & dishes	27146150	Chicken curry
959	19-0123	A-19-0123		Chicken kiev, frozen, baked	11.01.00	Meat - white - Chicken & turkey & dishes	27146400	Chicken kiev
960	A-00-09501	00-09501		Chicken korma	11.01.00	Meat - white - Chicken & turkey & dishes	27146150	Chicken curry
961	A-17-0254	17-0254		Chicken noodle soup, dried, as served	20.01.00	Soups - Canned & fresh & homemade	58403010	Chicken or turkey noodle soup, canned or ready-to-serve
962	A-19-0055	19-0055		Chicken pie, individual, chilled/frozen, baked	12.02.00	Processed meat - Processed pies	27347100	Chicken or turkey pot pie
963	19-0198	A-19-0198		Chicken risotto	01.03.00	Cereals & cereal dishes - Rice & rice dishes	27243000	Chicken or turkey and rice, no sauce
964	A-19-0125	19-0125		Chicken roll	12.02.00	Processed meat - Processed pies	58101460	Soft taco with chicken and sour cream
965	19-0126			Chicken slices	11.01.00	Meat - white - Chicken & turkey & dishes	25230320	Chicken, prepackaged or deli, luncheon meat
966	A-17-0250	17-0250		Chicken soup, cream of, canned	20.01.00	Soups - Canned & fresh & homemade	6016	Soup, cream of chicken, canned, condensed
967	17-0252	A-17-0252		Chicken soup, cream of, canned, condensed, as served	20.01.00	Soups - Canned & fresh & homemade	28345110	Chicken or turkey soup, cream of, NS as to prepared with milk or water
968	19-0127			Chicken tandoori, chilled, reheated	11.01.00	Meat - white - Chicken & turkey & dishes	24102000	Chicken, NS as to part, baked, broiled, or roasted, NS as to skin eaten
969	19-0204	A-19-0204		Chicken wings, marinated, chilled/frozen, barbecued	11.01.00	Meat - white - Chicken & turkey & dishes	24164010	Chicken wing, grilled with sauce

970	A-00-09528	00-09528		Chicken with Cashew nuts	11.01.00	Meat - white - Chicken & turkey & dishes	27445250	Almond chicken
971	A-00-00320			Chicken, Boiled, Dark Meat	11.01.00	Meat - white - Chicken & turkey & dishes	24100000	Chicken, NS as to part and cooking method, NS as to skin eaten
972	A-00-00319			Chicken, Boiled, Light Meat	11.01.00	Meat - white - Chicken & turkey & dishes	24100000	Chicken, NS as to part and cooking method, NS as to skin eaten
973	A-00-00318			Chicken, Boiled, Meat Only	11.01.00	Meat - white - Chicken & turkey & dishes	24100000	Chicken, NS as to part and cooking method, NS as to skin eaten
974	A-00-01082	00-01082		Chicken, Fried, No Coating	11.01.00	Meat - white - Chicken & turkey & dishes	24107071	Chicken, NS as to part, fried, coated, skin / coating not eaten
975	00-01083	A-00-01083		Chicken, Fried, with Coating	11.01.00	Meat - white - Chicken & turkey & dishes	24107071	Chicken, NS as to part, fried, coated, skin / coating not eaten
976	A-00-00326			Chicken, Quarter, Leg (Weighed with Bone)	11.01.00	Meat - white - Chicken & turkey & dishes	24130210	Chicken leg, drumstick and thigh, NS as to cooking method, skin eaten
977	A-00-00325			Chicken, Quarter, Wing, (Weighed with Bone)	11.01.00	Meat - white - Chicken & turkey & dishes	24160110	Chicken wing, NS as to cooking method
978	A-00-00314			Chicken, Raw, Meat Only	11.01.00	Meat - white - Chicken & turkey & dishes	5062	Chicken, broiler or fryers, breast, skinless, boneless, meat only, raw
979	A-00-00315			Chicken, Raw, Meat and Skin	11.01.00	Meat - white - Chicken & turkey & dishes	5091	Chicken, broilers or fryers, thigh, meat and skin, raw
980	A-00-00324			Chicken, Roast, Dark Meat	11.01.00	Meat - white - Chicken & turkey & dishes	24102000	Chicken, NS as to part, baked, broiled, or roasted, NS as to skin eaten

981	A-00-00323			Chicken, Roast, Light Meat	11.01.00	Meat - white - Chicken & turkey & dishes	24102000	Chicken, NS as to part, baked, broiled, or roasted, NS as to skin eaten
982	A-00-00321			Chicken, Roast, Meat Only	11.01.00	Meat - white - Chicken & turkey & dishes	24102000	Chicken, NS as to part, baked, broiled, or roasted, NS as to skin eaten
983	A-00-00322			Chicken, Roast, Meat and Skin	11.01.00	Meat - white - Chicken & turkey & dishes	24102000	Chicken, NS as to part, baked, broiled, or roasted, NS as to skin eaten
984	00-05666			Chicken, breaded fillets, low fat	11.01.00	Meat - white - Chicken & turkey & dishes	24107070	Chicken, NS as to part, fried, coated, skin / coating eaten
985	00-03597	A-00-03597		Chicken, breaded, fried in known fat	11.01.00	Meat - white - Chicken & turkey & dishes	24107070	Chicken, NS as to part, fried, coated, skin / coating eaten
986	A-18-0307	18-0307		Chicken, breast, casseroled, meat only	11.01.00	Meat - white - Chicken & turkey & dishes	24120110	Chicken breast, NS as to cooking method, skin eaten
987	18-0324			Chicken, breast, grilled with skin, meat only	11.01.00	Meat - white - Chicken & turkey & dishes	24123300	Chicken breast, grilled without sauce, skin eaten
988	18-0323			Chicken, breast, grilled without skin, meat only	11.01.00	Meat - white - Chicken & turkey & dishes	24123301	Chicken breast, grilled without sauce, skin not eaten
989	18-0326			Chicken, breast, strips, stir-fried	11.01.00	Meat - white - Chicken & turkey & dishes	24124200	Chicken breast, sauteed, skin eaten
990	18-0329	A-18-0329		Chicken, dark meat, roasted	11.01.00	Meat - white - Chicken & turkey & dishes	24102000	Chicken, NS as to part, baked, broiled, or roasted, NS as to skin eaten

991	18-0310			Chicken, drumsticks, casseroled, meat only	11.01.00	Meat - white - Chicken & turkey & dishes	24130210	Chicken leg, drumstick and thigh, NS as to cooking method, skin eaten
992	18-0314	A-18-0314		Chicken, leg quarter, casseroled, meat and skin	11.01.00	Meat - white - Chicken & turkey & dishes	24130210	Chicken leg, drumstick and thigh, NS as to cooking method, skin eaten
993	18-0313			Chicken, leg quarter, casseroled, meat only	11.01.00	Meat - white - Chicken & turkey & dishes	24130220	Chicken leg, drumstick and thigh, NS as to cooking method, skin not eaten
994	A-18-0337	18-0337		Chicken, leg quarter, roasted, meat and skin	11.01.00	Meat - white - Chicken & turkey & dishes	24132240	Chicken leg, drumstick and thigh, rotisserie, skin eaten
995	A-18-0330	18-0330		Chicken, light meat, roasted	11.01.00	Meat - white - Chicken & turkey & dishes	24102000	Chicken, NS as to part, baked, broiled, or roasted, NS as to skin eaten
996	A-18-0331	18-0331		Chicken, meat, average, roasted	11.01.00	Meat - white - Chicken & turkey & dishes	24102000	Chicken, NS as to part, baked, broiled, or roasted, NS as to skin eaten
997	A-18-0327	18-0327		Chicken, portions, deep-fried, meat and skin	11.01.00	Meat - white - Chicken & turkey & dishes	24107071	Chicken, NS as to part, fried, coated, skin / coating not eaten
998	A-19-0200	19-0200		Chicken, stir-fried with peppers in black bean sauce	11.01.00	Meat - white - Chicken & turkey & dishes	27141500	Chili con carne with chicken or turkey and beans
999	19-0201			Chicken, stir-fried with rice and vegetables, frozen, reheated	11.01.00	Meat - white - Chicken & turkey & dishes	28141250	Chicken with rice and vegetable, diet frozen meal

Diet quality and cognitive ability, Cara et al.
Crosswalk linking food codes from the UK National Survey of Health and Development with the USDA Food Patterns Equivalents/Ingredients Databases

1000	18-0317	A-18-0317		Chicken, thighs, casseroled, meat and skin	11.01.00	Meat - white - Chicken & turkey & dishes	24152230	Chicken thigh, baked, broiled, or roasted, skin eaten, from raw
1001	18-0319			Chicken, thighs, diced, casseroled, meat only	11.01.00	Meat - white - Chicken & turkey & dishes	24152231	Chicken thigh, baked, broiled, or roasted, skin not eaten, from raw
1002	18-0339	A-18-0339		Chicken, wing quarter, roasted, meat and skin	11.01.00	Meat - white - Chicken & turkey & dishes	24162130	Chicken wing, baked, broiled, or roasted, from raw
1003	A-00-00596			Chicory, Raw	15.03.00	Vegetables - Yellow & red & dark green leafy vegetables	72124100	Radicchio, raw
1004	A-13-0225	13-0225		Chicory, raw	15.03.00	Vegetables - Yellow & red & dark green leafy vegetables	72124100	Radicchio, raw
1005	A-00-01088			Chili Con Carne	10.01.00	Meat - red - Beef & veal & dishes	27111400	Chili con carne, NS as to beans
1006	A-00-01211			Chilli Powder	26.01.00	Miscellaneous - Dried herbs & spices & pastes	2009	Spices, chili powder
1007	A-19-0206	19-0206		Chilli con carne	10.01.00	Meat - red - Beef & veal & dishes	27111400	Chili con carne, NS as to beans
1008	00-05877			Chilli con carne cooked in specified fat	10.01.00	Meat - red - Beef & veal & dishes	27111400	Chili con carne, NS as to beans
1009	19-0207			Chilli con carne, canned	10.01.00	Meat - red - Beef & veal & dishes	27111400	Chili con carne, NS as to beans
1010	19-0209			Chilli con carne, chilled/frozen, reheated, with rice	10.01.00	Meat - red - Beef & veal & dishes	27111420	Chili con carne without beans
1011	13-0812	A-13-0812		Chilli powder	26.01.00	Miscellaneous - Dried herbs & spices & pastes	2009	Spices, chili powder
1012	17-0294	A-17-0294		Chilli sauce	21.03.00	Sauces & accompaniment - Other sauces, incl. brown sauce, soy sauce, ketchup, mint sauce, vinegar	74402010	Tomato chili sauce
1013	15-0071			Chilli, bean and lentil	16.01.00	Pulses/Lentils - Pulses/lentils	41221020	Chili with beans, without meat

1014	15-0074			Chilli, vegetable, retail	15.04.00	Vegetables - Other	41812450	Vegetarian chili, made with meat substitute
1015	A-00-01220			Chillies, Green	15.04.00	Vegetables - Other	11977	Peppers, serrano, raw
1016	13-0813			Chinese 5 spice	26.01.00	Miscellaneous - Dried herbs & spices & pastes	2009	Spices, chili powder
1017	00-09531	A-00-09531		Chinese Crispy pancakes	01.04.00	Cereals & cereal dishes - Other cereals & dishes	55501000	Chinese pancake
1018	13-0023			Chips, French fries, retail	17.02.00	Potatoes - Potato products - other	71400990	Potato, french fries, NFS
1019	A-00-09937			Chips, French fries, retail (MW6 folate)	17.02.00	Potatoes - Potato products - other	71400990	Potato, french fries, NFS
1020	00-05879			Chips, homemade, cooked in specified fat	17.02.00	Potatoes - Potato products - other	71400990	Potato, french fries, NFS
1021	A-13-0407	13-0407		Chips, homemade, fried in blended oil	17.02.00	Potatoes - Potato products - other	71400990	Potato, french fries, NFS
1022	13-0021	A-13-0021		Chips, homemade, fried in corn oil	17.02.00	Potatoes - Potato products - other	71400990	Potato, french fries, NFS
1023	13-0406	A-13-0406		Chips, homemade, fried in dripping	17.02.00	Potatoes - Potato products - other	71400990	Potato, french fries, NFS
1024	13-0408	A-13-0408		Chips, homemade, fried in sunflower oil	17.02.00	Potatoes - Potato products - other	71400990	Potato, french fries, NFS
1025	A-13-0410			Chips, retail, fried in corn oil	17.02.00	Potatoes - Potato products - other	71400990	Potato, french fries, NFS
1026	A-13-0409			Chips, retail, fried in dripping	17.02.00	Potatoes - Potato products - other	71400990	Potato, french fries, NFS
1027	00-03610	A-00-03610		Chips, retail, fried in known fat	17.02.00	Potatoes - Potato products - other	71400990	Potato, french fries, NFS
1028	13-0411	A-13-0411		Chips, retail, fried in sunflower oil	17.02.00	Potatoes - Potato products - other	71400990	Potato, french fries, NFS
1029	13-0022			Chips, retail, fried in vegetable oil	17.02.00	Potatoes - Potato products - other	71400990	Potato, french fries, NFS
1030	A-00-09936			Chips, retail, fried in vegetable oil (MW6 folate)	17.02.00	Potatoes - Potato products - other	71400990	Potato, french fries, NFS

1031	A-13-0027			Chips, thick cut, frozen, fried in corn oil	17.02.00	Potatoes - Potato products - other	71400990	Potato, french fries, NFS
1032	13-0814	A-13-0814		Chives, fresh	15.03.00	Vegetables - Yellow & red & dark green leafy vegetables	75109500	Chives, raw
1033	A-00-03767			Choc Ice 82/1406	06.04.01	Dairy products - Ice cream & dairy desserts - full fat products	13120100	Ice cream bar or stick, chocolate covered
1034	00-09695	A-00-09695		Choc drinks, instant, low calorie	27.05.00	Beverages - Powdered Beverages (cocoa, Horlicks, Bonvita, Ovaltine, etc)	11830100	Hot chocolate / Cocoa, dry mix, not reconstituted
1035	A-12-0200	12-0200		Choc ice	06.04.01	Dairy products - Ice cream & dairy desserts - full fat products	13120100	Ice cream bar or stick, chocolate covered
1036	A-00-03010			Choco Cornflakes, Ownbrand	02.03.00	Breakfast cereals - Other breakfast cereals - low fibre	57100100	Cereal, ready-to-eat, NFS
1037	A-00-01030			Chocolate Cake, 1 Layer of Icing	04.02.00	Sweet cereal products - Pastries, Buns & Pies	53105270	Cake or cupcake, chocolate, devil's food or fudge, with icing or filling
1038	A-00-03768			Chocolate Crispie Cake 82/1407	04.02.00	Sweet cereal products - Pastries, Buns & Pies	57339500	Cereal (Kellogg's Rice Krispies Treats Cereal)
1039	A-00-03782			Chocolate Sauce 82/1424	21.03.00	Sauces & accompaniment - Other sauces, incl. brown sauce, soy sauce, ketchup, mint sauce, vinegar	91304020	Topping, chocolate, thick, fudge type
1040	A-00-01134			Chocolate Spread, Cadbury's	23.02.00	Sugars - Other, incl. syrups, honey	91304020	Topping, chocolate, thick, fudge type
1041	00-03158			Chocolate Tiffin biscuit bar	04.01.00	Sweet cereal products - Biscuits	53205260	Cookie, bar, with chocolate
1042	00-01136	A-00-01136		Chocolate and Nut Bars	24.01.00	Confectionary - Chocolate based products	91733200	Peanut Bar, chocolate covered candy
1043	A-00-03229	00-03229		Chocolate based dairy desserts	06.04.02	Dairy products - Ice cream & dairy desserts - reduced fat products	13110110	Ice cream, regular, chocolate
1044	A-00-09597	00-09597		Chocolate biscuits, cream filled, full coated	04.01.00	Sweet cereal products - Biscuits	53209015	Cookie, chocolate sandwich

1045	A-00-09596	A-11-0166	11-0166	Chocolate biscuits, full coated	04.01.00	Sweet cereal products - Biscuits	53209005	Cookie, chocolate, with icing or coating
1046	A-00-06013			Chocolate biscuits, full coated 50	04.01.00	Sweet cereal products - Biscuits	53209005	Cookie, chocolate, with icing or coating
1047	11-0195	A-11-0195		Chocolate cake	04.02.00	Sweet cereal products - Pastries, Buns & Pies	53105275	Cake or cupcake, chocolate, devil's food or fudge, without icing or filling
1048	A-00-06021			Chocolate cake 50	04.02.00	Sweet cereal products - Pastries, Buns & Pies	53105275	Cake or cupcake, chocolate, devil's food or fudge, without icing or filling
1049	A-11-0196	11-0196		Chocolate cake, with butter icing	04.02.00	Sweet cereal products - Pastries, Buns & Pies	53105270	Cake or cupcake, chocolate, devil's food or fudge, with icing or filling
1050	A-00-06059			Chocolate cake, with butter icing 50	04.02.00	Sweet cereal products - Pastries, Buns & Pies	53105270	Cake or cupcake, chocolate, devil's food or fudge, with icing or filling
1051	A-00-09598	00-09598		Chocolate chip cookies	04.01.00	Sweet cereal products - Biscuits	53206000	Cookie, chocolate chip
1052	17-0084	A-17-0084		Chocolate covered bar with fruit/nut wafer/biscuit - Lion Bar	24.01.00	Confectionary - Chocolate based products	91705050	Milk chocolate candy, with fruit and nuts
1053	17-0083	A-17-0083		Chocolate covered caramels including Rolo, Caramel	24.01.00	Confectionary - Chocolate based products	91703070	Rolo
1054	A-00-09636	00-09636		Chocolate covered ice cream bar, non-dairy, eg Mars	06.04.01	Dairy products - Ice cream & dairy desserts - full fat products	13120100	Ice cream bar or stick, chocolate covered
1055	A-00-09675	00-09675		Chocolate dairy dessert	06.04.01	Dairy products - Ice cream & dairy desserts - full fat products	13120100	Ice cream bar or stick, chocolate covered

1056	00-09620	A-00-09620		Chocolate fudge cake	04.02.00	Sweet cereal products - Pastries, Buns & Pies	53105275	Cake or cupcake, chocolate, devil's food or fudge, without icing or filling
1057	00-03128	A-00-03128		Chocolate mini roll	04.02.00	Sweet cereal products - Pastries, Buns & Pies	53108200	Snack cake, chocolate, with icing or filling
1058	17-0070	A-17-0070		Chocolate nut spread	23.02.00	Sugars - Other, incl. syrups, honey	91304090	Topping, chocolate flavored hazelnut spread
1059	A-12-0201			Chocolate nut sundae	06.04.01	Dairy products - Ice cream & dairy desserts - full fat products	13121300	Ice cream sundae, chocolate or fudge topping, with whipped cream
1060	00-05661			Chocolate pudding with chocolate sauce	04.03.00	Sweet cereal products - Cereal based puddings (not milk)	53118300	Cake, sponge, chocolate
1061	A-17-0069	17-0069		Chocolate spread	23.02.00	Sugars - Other, incl. syrups, honey	91304020	Topping, chocolate, thick, fudge type
1062	A-00-00860			Chocolate, Bounty Bar	24.01.00	Confectionary - Chocolate based products	91726420	3 MUSKETEERS Bar
1063	A-00-00859			Chocolate, Fancy and Filled	24.01.00	Confectionary - Chocolate based products	91760500	Truffles
1064	A-00-00861			Chocolate, Mars Bar	24.01.00	Confectionary - Chocolate based products	91726130	MILKY WAY Bar
1065	A-00-00857			Chocolate, Milk	24.01.00	Confectionary - Chocolate based products	91705010	Milk chocolate candy, plain
1066	A-00-00858			Chocolate, Plain	24.01.00	Confectionary - Chocolate based products	91705300	Chocolate, sweet or dark
1067	A-17-0087	17-0087		Chocolate, diabetic	24.01.00	Confectionary - Chocolate based products	91770030	Dietetic or low calorie candy, chocolate covered
1068	A-17-0088	17-0088		Chocolate, fancy and filled	24.01.00	Confectionary - Chocolate based products	91760500	Truffles
1069	17-0089	A-17-0089		Chocolate, milk including Dairy Milk, Galaxy, chocolate	24.01.00	Confectionary - Chocolate based products	91705010	Milk chocolate candy, plain

1070	A-17-0090	17-0090		Chocolate, plain	24.01.00	Confectionary - Chocolate based products	91705300	Chocolate, sweet or dark
1071	00-05562			Chocolate, plain (70% cocoa)	24.01.00	Confectionary - Chocolate based products	91705300	Chocolate, sweet or dark
1072	A-17-0091	17-0091		Chocolate, white	24.01.00	Confectionary - Chocolate based products	91705400	Chocolate, white
1073	00-05907			Cholesterol reducing alternative to cheese eg Minicol	06.02.00	Dairy products - Cheese, incl. cottage cheese	14502000	Imitation cheese
1074	A-00-01172			Chop Sueys, All Types	10.01.00	Meat - red - Beef & veal & dishes	27313110	Beef chow mein or chop suey with noodles
1075	A-00-09526	00-09526		Chop suey, Chicken	11.01.00	Meat - white - Chicken & turkey & dishes	27446100	Chicken or turkey chow mein or chop suey, no noodles
1076	00-05600			Chorizo (diced) e.g. Tesco/Sainsbury's	12.03.00	Processed meat - Other processed meats	25220710	Chorizo
1077	11-0233	A-11-0233		Choux buns	04.02.00	Sweet cereal products - Pastries, Buns & Pies	53521230	Doughnut, custard-filled, with icing
1078	A-00-06068			Choux buns 50	04.02.00	Sweet cereal products - Pastries, Buns & Pies	53521230	Doughnut, custard-filled, with icing
1079	A-00-01171			Chow Mein, All Types	10.01.00	Meat - red - Beef & veal & dishes	27313110	Beef chow mein or chop suey with noodles
1080	A-00-09520	00-09520		Chow Mein, Chicken,	11.01.00	Meat - white - Chicken & turkey & dishes	27343910	Chicken or turkey chow mein or chop suey with noodles
1081	A-00-00100			Christmas Pudding	04.03.00	Sweet cereal products - Cereal based puddings (not milk)	53110000	Cake, fruit cake, light or dark, holiday type cake
1082	A-00-06352			Christmas Pudding (dripping) 50	04.03.00	Sweet cereal products - Cereal based puddings (not milk)	53110000	Cake, fruit cake, light or dark, holiday type cake
1083	A-11-0290	11-0290		Christmas pudding	04.03.00	Sweet cereal products - Cereal based puddings (not milk)	53110000	Cake, fruit cake, light or dark, holiday type cake
1084	11-0291	A-11-0291		Christmas pudding, retail	04.03.00	Sweet cereal products - Cereal based puddings (not milk)	53110000	Cake, fruit cake, light or dark, holiday type cake

1085	A-00-00923			Chutney, Apple	22.02.00	Preserves - Chutney & Pickles (incl. gherkins, pickled onions etc)	63409020	Chutney
1086	A-00-00924			Chutney, Tomato	22.02.00	Preserves - Chutney & Pickles (incl. gherkins, pickled onions etc)	74402010	Tomato chili sauce
1087	17-0341	A-17-0341		Chutney, apple, homemade	22.02.00	Preserves - Chutney & Pickles (incl. gherkins, pickled onions etc)	63409020	Chutney
1088	A-17-0343	17-0343		Chutney, mango, sweet	22.02.00	Preserves - Chutney & Pickles (incl. gherkins, pickled onions etc)	63409020	Chutney
1089	17-0345	A-17-0345		Chutney, tomato	22.02.00	Preserves - Chutney & Pickles (incl. gherkins, pickled onions etc)	74402010	Tomato chili sauce
1090	A-00-09572	00-09572		Ciabatta, plain	03.01.00	Breads - White	51109010	Bread, Italian, Grecian, Armenian
1091	A-00-00901			Cider, Dry	27.01.03	Beverages - Alcohol - Beer	93101000	Beer
1092	A-00-00902			Cider, Sweet	27.01.03	Beverages - Alcohol - Beer	93101000	Beer
1093	A-00-00903			Cider, Vintage	27.01.03	Beverages - Alcohol - Beer	93101000	Beer
1094	17-0222	A-17-0222		Cider, dry	27.01.03	Beverages - Alcohol - Beer	93101000	Beer
1095	17-0223			Cider, low alcohol including Strongbow LA	27.01.06	Beverages - Alcohol - Low alcohol beer	93101000	Beer
1096	A-17-0224	17-0224		Cider, sweet	27.01.03	Beverages - Alcohol - Beer	93101000	Beer
1097	17-0225			Cider, vintage	27.01.03	Beverages - Alcohol - Beer	93101000	Beer
1098	00-05892			Cider, with cooking losses	27.01.03	Beverages - Alcohol - Beer	93101000	Beer
1099	00-05622			Cinnamon Grahams (Nestle)	02.03.00	Breakfast cereals - Other breakfast cereals - low fibre	57100100	Cereal, ready-to-eat, NFS
1100	13-0815	A-13-0815		Cinnamon, ground	26.01.00	Miscellaneous - Dried herbs & spices & pastes	2010	Spices, cinnamon, ground
1101	14-0071	A-14-0071		Clementines	18.01.00	Fruit - Fresh	61119010	Orange, raw
1102	A-00-03802			Clotted Cream 82/1444	06.01.00	Dairy products - Cream & fromage frais	12130100	Cream, heavy

1103	13-0816			Cloves, dried	26.01.00	Miscellaneous - Dried herbs & spices & pastes	2010	Spices, cinnamon, ground
1104	A-00-03011	00-03011		Clusters, Nestle	02.02.00	Breakfast cereals - Other breakfast cereals - high fibre (equal or >3g/40g portion)	57100100	Cereal, ready-to-eat, NFS
1105	A-00-00830			Cob or Hazel Nuts	19.00.00	Nuts & Seeds (incl. peanut butter)	42107000	Hazelnuts
1106	A-00-00831			Cob or Hazel Nuts (Weighed with Shells)	19.00.00	Nuts & Seeds (incl. peanut butter)	42107000	Hazelnuts
1107	A-00-00878			Coca-cola	27.03.00	Beverages - Carbonated soft drinks	92400000	Soft drink, NFS
1108	A-00-00531			Cockles, Boiled	09.03.00	Fish & fish dishes - Shellfish	26303160	Clams, steamed or boiled
1109	A-16-0252	16-0252		Cockles, boiled	09.03.00	Fish & fish dishes - Shellfish	26303160	Clams, steamed or boiled
1110	A-11-0129			Coco Pops	02.03.00	Breakfast cereals - Other breakfast cereals - low fibre	57126000	Cereal (Kellogg's Cocoa Krispies)
1111	A-00-06501			Cocoa 50 powder	27.05.00	Beverages - Powdered Beverages (cocoa, Horlicks, Bonvita, Ovaltine, etc)	11830150	Cocoa powder, not reconstituted
1112	A-00-00868			Cocoa Powder	27.05.00	Beverages - Powdered Beverages (cocoa, Horlicks, Bonvita, Ovaltine, etc)	11830150	Cocoa powder, not reconstituted
1113	A-12-0082	12-0082		Cocoa powder	27.05.00	Beverages - Powdered Beverages (cocoa, Horlicks, Bonvita, Ovaltine, etc)	11830150	Cocoa powder, not reconstituted
1114	A-12-0084			Cocoa powder, made up with semi-skimmed milk	27.05.00	Beverages - Powdered Beverages (cocoa, Horlicks, Bonvita, Ovaltine, etc)	11514120	Hot chocolate / Cocoa, made with dry mix and reduced fat milk
1115	A-12-0085			Cocoa powder, made up with skimmed milk	27.05.00	Beverages - Powdered Beverages (cocoa, Horlicks, Bonvita, Ovaltine, etc)	11514140	Hot chocolate / Cocoa, made with dry mix and fat free milk
1116	A-12-0083			Cocoa powder, made up with whole milk	27.05.00	Beverages - Powdered Beverages (cocoa, Horlicks, Bonvita, Ovaltine, etc)	11514110	Hot chocolate / Cocoa, made with dry mix and whole milk

1117	00-03805	A-00-03805		Coconut Macaroons 82/1448	04.01.00	Sweet cereal products - Biscuits	53215500	Cookie, coconut
1118	A-00-00833			Coconut Milk	19.00.00	Nuts & Seeds (incl. peanut butter)	42401010	Coconut milk, used in cooking
1119	A-00-00196			Coconut Oil	08.02.00	Fats - Oils	82101500	Coconut oil
1120	A-11-0197			Coconut cake	04.02.00	Sweet cereal products - Pastries, Buns & Pies	53104400	Cake or cupcake, coconut, with icing or filling
1121	A-00-06022			Coconut cake 50	04.02.00	Sweet cereal products - Pastries, Buns & Pies	53104400	Cake or cupcake, coconut, with icing or filling
1122	11-0197			Coconut cake, homemade	04.02.00	Sweet cereal products - Pastries, Buns & Pies	53104400	Cake or cupcake, coconut, with icing or filling
1123	A-14-0819	14-0819		Coconut cream	19.00.00	Nuts & Seeds (incl. peanut butter)	42402010	Coconut cream, canned, sweetened
1124	17-0105	A-17-0105		Coconut ice	24.02.00	Confectionary - Sugar based products	91700010	Candy, NFS
1125	A-14-0820			Coconut milk	19.00.00	Nuts & Seeds (incl. peanut butter)	42401010	Coconut milk, used in cooking
1126	A-17-0031	17-0031		Coconut oil	08.02.00	Fats - Oils	82101500	Coconut oil
1127	A-00-00834			Coconut, Desiccated	19.00.00	Nuts & Seeds (incl. peanut butter)	42106020	Coconut, packaged
1128	A-00-00832			Coconut, Fresh	18.01.00	Fruit - Fresh	42106000	Coconut, fresh
1129	14-0817	A-14-0817		Coconut, creamed block	19.00.00	Nuts & Seeds (incl. peanut butter)	42402010	Coconut cream, canned, sweetened
1130	A-14-0818	14-0818		Coconut, desiccated	19.00.00	Nuts & Seeds (incl. peanut butter)	42106020	Coconut, packaged
1131	A-14-0816	14-0816		Coconut, fresh	18.01.00	Fruit - Fresh	42106000	Coconut, fresh
1132	00-03012	A-00-03012		Cocopops, Kelloggs	02.03.00	Breakfast cereals - Other breakfast cereals - low fibre	57126000	Cereal (Kellogg's Cocoa Krispies)
1133	00-03013	A-00-03013		Cocopops, Own Brand	02.03.00	Breakfast cereals - Other breakfast cereals - low fibre	57126000	Cereal (Kellogg's Cocoa Krispies)

1134	A-00-03808			Cod Crispy Steaks (B.E.) 82/1451	09.01.00	Fish & fish dishes - White fish, incl. tuna	26109121	Cod, baked or broiled, made with butter
1135	00-05362			Cod Steak in Butter Sauce	09.01.00	Fish & fish dishes - White fish, incl. tuna	26109133	Cod, coated, baked or broiled, made without fat
1136	00-03634			Cod fishcakes from Carmel Moore's Study	09.01.00	Fish & fish dishes - White fish, incl. tuna	26100270	Fish stick, patty or nugget from restaurant, home, or other place
1137	A-00-03795			Cod in cheese sauce (B.E.) 82/1437	09.01.00	Fish & fish dishes - White fish, incl. tuna	27150510	Scallops with cheese sauce
1138	00-03606			Cod in crumbs, ovenbaked	09.01.00	Fish & fish dishes - White fish, incl. tuna	26109133	Cod, coated, baked or broiled, made without fat
1139	A-00-00440			Cod, Baked	09.01.00	Fish & fish dishes - White fish, incl. tuna	26109123	Cod, baked or broiled, made without fat
1140	A-00-00450			Cod, Dried, Salt, Boiled	09.01.00	Fish & fish dishes - White fish, incl. tuna	26109180	Cod, dried, salted, salt removed in water
1141	A-00-00442			Cod, Fried in Batter	09.01.00	Fish & fish dishes - White fish, incl. tuna	26109140	Cod, coated, fried, made with oil
1142	A-00-06250			Cod, Fried in Batter (dripping) 50	09.01.00	Fish & fish dishes - White fish, incl. tuna	26109140	Cod, coated, fried, made with oil
1143	A-00-00443			Cod, Grilled	09.01.00	Fish & fish dishes - White fish, incl. tuna	26109123	Cod, baked or broiled, made without fat
1144	A-00-00444			Cod, Poached	09.01.00	Fish & fish dishes - White fish, incl. tuna	26107160	Catfish, steamed or poached
1145	A-00-00445			Cod, Poached (Weighed with Bones and Skin)	09.01.00	Fish & fish dishes - White fish, incl. tuna	26107160	Catfish, steamed or poached
1146	A-00-00438			Cod, Raw, Fresh Fillets	09.01.00	Fish & fish dishes - White fish, incl. tuna	15019	Fish, cod, Pacific, raw (may have been previously frozen)
1147	A-00-00439			Cod, Raw, Frozen Steaks	09.01.00	Fish & fish dishes - White fish, incl. tuna	15019	Fish, cod, Pacific, raw (may have been previously frozen)

1148	A-00-00449			Cod, Smoked, Poached	09.01.00	Fish & fish dishes - White fish, incl. tuna	26109160	Cod, steamed or poached
1149	A-00-00446			Cod, Steamed	09.01.00	Fish & fish dishes - White fish, incl. tuna	26109160	Cod, steamed or poached
1150	A-00-00447			Cod, Steamed (Weighed with Bones and Skin)	09.01.00	Fish & fish dishes - White fish, incl. tuna	26109160	Cod, steamed or poached
1151	A-16-0013	16-0013		Cod, baked	09.01.00	Fish & fish dishes - White fish, incl. tuna	26109123	Cod, baked or broiled, made without fat
1152	16-0025	A-16-0025		Cod, coated in batter, frozen, baked	09.01.00	Fish & fish dishes - White fish, incl. tuna	26109133	Cod, coated, baked or broiled, made without fat
1153	16-0027	A-16-0027		Cod, coated in crumbs, frozen, fried in blended oil	09.01.00	Fish & fish dishes - White fish, incl. tuna	26109140	Cod, coated, fried, made with oil
1154	A-16-0020	16-0020		Cod, frozen, grilled	09.01.00	Fish & fish dishes - White fish, incl. tuna	26109123	Cod, baked or broiled, made without fat
1155	16-0021	A-16-0021		Cod, in batter, fried in blended oil	09.01.00	Fish & fish dishes - White fish, incl. tuna	26109140	Cod, coated, fried, made with oil
1156	A-16-0022			Cod, in batter, fried in dripping	09.01.00	Fish & fish dishes - White fish, incl. tuna	26109140	Cod, coated, fried, made with oil
1157	16-0024	A-16-0024		Cod, in batter, fried in sunflower oil	09.01.00	Fish & fish dishes - White fish, incl. tuna	26109140	Cod, coated, fried, made with oil
1158	A-16-0030	16-0030		Cod, in parsley sauce, frozen, boiled	09.01.00	Fish & fish dishes - White fish, incl. tuna	26109160	Cod, steamed or poached
1159	A-16-0015	16-0015		Cod, poached	09.01.00	Fish & fish dishes - White fish, incl. tuna	26107160	Catfish, steamed or poached
1160	A-00-03859	00-03859		Coffee BLACK, average, made up	27.06.00	Beverages - Coffee	92100000	Coffee, NS as to type
1161	A-12-0026	12-0026		Coffee Compliment	27.05.00	Beverages - Powdered Beverages (cocoa, Horlicks, Bonvita, Ovaltine, etc)	12210400	Coffee creamer, powder
1162	A-00-00869			Coffee and Chicory Essence	27.06.00	Beverages - Coffee	92100000	Coffee, NS as to type
1163	17-0162	A-17-0162		Coffee and chicory essence	27.06.00	Beverages - Coffee	92100000	Coffee, NS as to type

1164	00-09749	A-00-09749		Coffee creamer, liquid, gluc + fat	05.04.00	Milk - Other - plant based, e.g. rice, soy	12210200	Coffee creamer, liquid
1165	A-00-03861	00-03861		Coffee decaffinated instant, made up	27.06.00	Beverages - Coffee	92100000	Coffee, NS as to type
1166	00-09748	A-00-09748		Coffee whitener, liquid, skimmed milk+fat	05.01.00	Milk - Skimmed milk	12210200	Coffee creamer, liquid
1167	A-00-09747	00-09747		Coffee whitener, powder, low fat	27.05.00	Beverages - Powdered Beverages (cocoa, Horlicks, Bonvita, Ovaltine, etc)	12210400	Coffee creamer, powder
1168	A-00-00870			Coffee, Ground, Roasted	27.05.00	Beverages - Powdered Beverages (cocoa, Horlicks, Bonvita, Ovaltine, etc)	92100500	Coffee, NS as to brewed or instant
1169	A-00-00871			Coffee, Infusion, 5 Minutes	27.06.00	Beverages - Coffee	92100000	Coffee, NS as to type
1170	A-00-00872			Coffee, Instant	27.06.00	Beverages - Coffee	92100000	Coffee, NS as to type
1171	A-00-01143			Coffee, Instant, Made-up	27.06.00	Beverages - Coffee	92100000	Coffee, NS as to type
1172	00-03846	A-00-03846		Coffee, decaffinated granules	27.06.00	Beverages - Coffee	92100000	Coffee, NS as to type
1173	A-00-01384			Coffee, decaffinated instant, made up	27.06.00	Beverages - Coffee	92100000	Coffee, NS as to type
1174	A-17-0152	17-0152		Coffee, infusion, average	27.06.00	Beverages - Coffee	92100000	Coffee, NS as to type
1175	17-0158	A-17-0158		Coffee, instant	27.06.00	Beverages - Coffee	92100000	Coffee, NS as to type
1176	17-0159	A-17-0159		Coffee, instant, made up with water	27.06.00	Beverages - Coffee	92100000	Coffee, NS as to type
1177	A-00-01157	12-0027	A-12-0027	Coffeemate	27.05.00	Beverages - Powdered Beverages (cocoa, Horlicks, Bonvita, Ovaltine, etc)	12210400	Coffee creamer, powder
1178	A-17-0175	17-0175		Cola	27.03.00	Beverages - Carbonated soft drinks	92400000	Soft drink, NFS
1179	A-00-01105			Cole-slaw (shop Bought)	15.04.00	Vegetables - Other	75140990	Cabbage salad or coleslaw, from fast food / restaurant
1180	15-0077	A-15-0077		Coleslaw, with mayonnaise, retail	15.04.00	Vegetables - Other	75140990	Cabbage salad or coleslaw, from fast food / restaurant

1181	A-15-0078	15-0078		Coleslaw, with reduced calorie dressing, retail	15.04.00	Vegetables - Other	75141005	Cabbage salad or coleslaw, made with light coleslaw dressing
1182	15-0079	A-15-0079		Coleslaw, with vinaigrette, retail	15.04.00	Vegetables - Other	75141020	Cabbage salad or coleslaw, made with Italian dressing
1183	16-0032	A-16-0032		Coley, steamed	09.01.00	Fish & fish dishes - White fish, incl. tuna	26109160	Cod, steamed or poached
1184	00-05326			Colmans Casserole Mixes	21.02.00	Sauces & accompaniment - Cooking sauces, incl. gravies, pesto, cooking sauces for pasta and rice dishes	28500040	Gravy, beef or meat
1185	A-00-01056			Commercial Desserts, Mousse Type	06.04.02	Dairy products - Ice cream & dairy desserts - reduced fat products	13250000	Mousse, chocolate
1186	A-00-01057			Commercial Desserts, with Fresh Cream	06.04.01	Dairy products - Ice cream & dairy desserts - full fat products	13110000	Ice cream, NFS
1187	A-00-03059	00-03059		Common Sense Oatbran Flakes, Kelloggs	02.01.00	Breakfast cereals - Oat based cereals	57000100	Cereal, oat, NFS
1188	A-12-0086			Complan powder, savoury	30.00.00	Nutrition Powders & drinks	95220000	Nutritional powder mix, NFS
1189	A-12-0087			Complan powder, savoury, made up with water	30.00.00	Nutrition Powders & drinks	95120000	Nutritional drink or shake, ready-to-drink, NFS
1190	A-12-0088			Complan powder, sweet	30.00.00	Nutrition Powders & drinks	95220000	Nutritional powder mix, NFS
1191	A-12-0091			Complan powder, sweet, made up with semi-skimmed milk	30.00.00	Nutrition Powders & drinks	11514120	Hot chocolate / Cocoa, made with dry mix and reduced fat milk
1192	A-12-0089			Complan powder, sweet, made up with water	30.00.00	Nutrition Powders & drinks	95120000	Nutritional drink or shake, ready-to-drink, NFS

1193	A-12-0090			Complan powder, sweet, made up with whole milk	30.00.00	Nutrition Powders & drinks	11514110	Hot chocolate / Cocoa, made with dry mix and whole milk
1194	A-00-03748			Complan, chocolate flavour 82/1331	30.00.00	Nutrition Powders & drinks	95220000	Nutritional powder mix, NFS
1195	A-00-03827			Compliment 82/1933	05.06.00	Milk - Milk based drinks, e.g. flavoured milks	11810000	Milk, dry, not reconstituted, NS as to fat content
1196	00-05840			Compound Cooking Fat, vegetable Fats. E.g. Cookeen , Trex	08.04.01	Fats - Plant based fats (solid) - Full fat	81102000	Margarine, NFS
1197	17-0004	A-17-0004		Compound cooking fat, e.g. Cookeen and White Cap	08.03.00	Fats - Animal based fats (solid)	81201000	Animal fat or drippings
1198	17-0005	A-17-0005		Compound cooking fat, polyunsaturated, White Flora	08.04.01	Fats - Plant based fats (solid) - Full fat	81102000	Margarine, NFS
1199	12-0028	A-12-0028		Condensed milk, skimmed, sweetened	05.01.00	Milk - Skimmed milk	11220000	Milk, condensed, sweetened
1200	12-0029	A-12-0029		Condensed milk, whole, sweetened	05.03.00	Milk - Whole milk	11220000	Milk, condensed, sweetened
1201	00-05713			Cook in Sauce, White based	21.02.00	Sauces & accompaniment - Cooking sauces, incl. gravies, pesto, cooking sauces for pasta and rice dishes	13411000	White sauce, milk sauce
1202	17-0295	A-17-0295		Cook-in-sauces, canned	21.03.00	Sauces & accompaniment - Other sauces, incl. brown sauce, soy sauce, ketchup, mint sauce, vinegar	74404010	Spaghetti sauce
1203	13-0817	A-13-0817		Coriander leaves, fresh	15.04.00	Vegetables - Other	11165	Coriander (cilantro) leaves, raw
1204	13-0819			Coriander seeds	19.00.00	Nuts & Seeds (incl. peanut butter)	2014	Spices, cumin seed

1205	A-11-0130			Corn Flakes	02.03.00	Breakfast cereals - Other breakfast cereals - low fibre	57134000	Cereal, corn flakes
1206	A-00-03058			Corn Pops, Kelloggs	02.03.00	Breakfast cereals - Other breakfast cereals - low fibre	57347000	Cereal (Kellogg's Corn Pops)
1207	A-17-0126			Corn and starch snacks including Skips	25.02.00	Savoury Snacks - Cereal based snacks	54401055	Cheese flavored corn snacks
1208	15-0081	A-15-0081		Corn fritters, fried in vegetable oil	01.04.00	Cereals & cereal dishes - Other cereals & dishes	75411020	Corn fritter
1209	17-0033	A-17-0033		Corn oil	08.02.00	Fats - Oils	82102000	Corn oil
1210	A-17-0125			Corn snacks including Wotsits, Monster Munch and Nik-Naks	25.02.00	Savoury Snacks - Cereal based snacks	54401055	Cheese flavored corn snacks
1211	00-03161			Corn thins - crackers	25.04.00	Savoury Snacks - Savoury biscuits & crackers	54339000	Crackers, corn
1212	A-00-06208			Corned Beef 50	10.01.00	Meat - red - Beef & veal & dishes	21416000	Corned beef, cooked, NS as to fat eaten
1213	19-0212	A-19-0212		Corned beef hash	10.01.00	Meat - red - Beef & veal & dishes	21416000	Corned beef, cooked, NS as to fat eaten
1214	A-19-0128	19-0128		Corned beef, canned	10.01.00	Meat - red - Beef & veal & dishes	21416150	Corned beef, canned, ready-to-eat
1215	A-12-0202			Cornetto	06.04.02	Dairy products - Ice cream & dairy desserts - reduced fat products	13120710	Ice cream cone, chocolate covered, with nuts, flavors other than chocolate
1216	A-00-09637	00-09637		Cornetto, chocolate and nut	06.04.02	Dairy products - Ice cream & dairy desserts - reduced fat products	13120710	Ice cream cone, chocolate covered, with nuts, flavors other than chocolate
1217	00-09638	A-00-09638		Cornetto, strawberry	06.04.02	Dairy products - Ice cream & dairy desserts - reduced fat products	13120720	Ice cream cone, chocolate covered or dipped, flavors other than chocolate
1218	A-00-00048			Cornflakes	02.03.00	Breakfast cereals - Other breakfast cereals - low fibre	57134000	Cereal, corn flakes

1219	A-00-06002			Cornflakes 50	02.03.00	Breakfast cereals - Other breakfast cereals - low fibre	57134000	Cereal, corn flakes
1220	00-03014	A-00-03014		Cornflakes, Kelloggs	02.03.00	Breakfast cereals - Other breakfast cereals - low fibre	57134000	Cereal, corn flakes
1221	00-03015	A-00-03015		Cornflakes, Own Brand	02.03.00	Breakfast cereals - Other breakfast cereals - low fibre	57134000	Cereal, corn flakes
1222	A-00-01023			Cornflakes, Frosted (`Frosties')	02.03.00	Breakfast cereals - Other breakfast cereals - low fibre	57348000	Cereal, frosted corn flakes
1223	11-0010	A-11-0010	A-00-00006	Cornflour	01.04.00	Cereals & cereal dishes - Other cereals & dishes	20017	Corn flour, masa, enriched, white
1224	A-00-00420			Cornish Pastie	12.02.00	Processed meat - Processed pies	58126110	Turnover, meat-filled, no gravy
1225	19-0056	A-19-0056		Cornish pastie	12.02.00	Processed meat - Processed pies	58126110	Turnover, meat-filled, no gravy
1226	11-0011	A-11-0011		Cornmeal, sifted	01.04.00	Cereals & cereal dishes - Other cereals & dishes	20022	Cornmeal, degermed, enriched, yellow
1227	19-0213	A-19-0213		Coronation chicken	11.01.00	Meat - white - Chicken & turkey & dishes	27146150	Chicken curry
1228	A-00-01118			Cottage Cheese, Fatless	06.02.00	Dairy products - Cheese, incl. cottage cheese	14204010	Cheese, cottage, low fat
1229	A-00-09720	00-09720	A-12-0147	Cottage cheese, plain	06.02.00	Dairy products - Cheese, incl. cottage cheese	14200100	Cheese, cottage, NFS
1230	A-12-0149			Cottage cheese, plain, reduced fat	06.02.00	Dairy products - Cheese, incl. cottage cheese	14204010	Cheese, cottage, low fat
1231	12-0148	A-12-0148		Cottage cheese, plain, with additions	06.02.00	Dairy products - Cheese, incl. cottage cheese	14200100	Cheese, cottage, NFS
1232	00-09719	A-00-09719		Cottage cheese, reduced fat	06.02.00	Dairy products - Cheese, incl. cottage cheese	14204010	Cheese, cottage, low fat
1233	19-0215			Cottage pie, homemade	10.01.00	Meat - red - Beef & veal & dishes	27311510	Shepherd's pie with beef
1234	A-19-0215			Cottage/Shepherd's pie	10.01.00	Meat - red - Beef & veal & dishes	27311510	Shepherd's pie with beef
1235	19-0216			Cottage/Shepherd's pie, chilled/frozen, reheated	10.01.00	Meat - red - Beef & veal & dishes	27311510	Shepherd's pie with beef

1236	A-00-03016			Country Crisp (Banana), Jordans	02.03.00	Breakfast cereals - Other breakfast cereals - low fibre	57100100	Cereal, ready-to-eat, NFS
1237	A-00-03017			Country Crisp (Raspberry), Jordans	02.03.00	Breakfast cereals - Other breakfast cereals - low fibre	57100100	Cereal, ready-to-eat, NFS
1238	00-03017			Country Crisp, raspberry, Jordans	02.03.00	Breakfast cereals - Other breakfast cereals - low fibre	57100100	Cereal, ready-to-eat, NFS
1239	13-0231	A-13-0231		Courgette, boiled in unsalted water	15.04.00	Vegetables - Other	75233000	Squash, summer, yellow or green, cooked, NS as to form, NS as to fat added in cooking
1240	A-13-0232			Courgette, fried in corn oil	15.04.00	Vegetables - Other	75233000	Squash, summer, yellow or green, cooked, NS as to form, NS as to fat added in cooking
1241	13-0230	A-13-0230		Courgette, raw	15.04.00	Vegetables - Other	75128010	Squash, summer, green, raw
1242	00-03609	A-00-03609		Courgettes, fried in known fat	15.04.00	Vegetables - Other	75233000	Squash, summer, yellow or green, cooked, NS as to form, NS as to fat added in cooking
1243	00-05572			Cous cous salad (eg Tesco)	01.04.00	Cereals & cereal dishes - Other cereals & dishes	58148115	Macaroni or pasta salad, made with light Italian dressing
1244	11-0339	A-11-0339		Couscous,cooked, millet based	01.04.00	Cereals & cereal dishes - Other cereals & dishes	20029	Couscous, cooked
1245	00-09992			Couscous,cooked,wheat based	01.04.00	Cereals & cereal dishes - Other cereals & dishes	20029	Couscous, cooked
1246	00-05679			Covent Garden Chicken Soup	20.01.00	Soups - Canned & fresh & homemade	28340600	Chicken or turkey vegetable soup, canned, prepared with water or ready-to-serve
1247	A-00-00518			Crab, Boiled	09.03.00	Fish & fish dishes - Shellfish	26305160	Crab, hard shell, steamed
1248	A-00-00519			Crab, Boiled (Weighed with Shell)	09.03.00	Fish & fish dishes - Shellfish	26305160	Crab, hard shell, steamed

1249	A-00-00520			Crab, Canned	09.03.00	Fish & fish dishes - Shellfish	26305180	Crab, canned
1250	16-0232	A-16-0232		Crab, boiled	09.03.00	Fish & fish dishes - Shellfish	26305160	Crab, hard shell, steamed
1251	A-16-0234	16-0234		Crab, canned in brine, drained	09.03.00	Fish & fish dishes - Shellfish	26305180	Crab, canned
1252	16-0273	A-16-0273		Crabsticks	09.03.00	Fish & fish dishes - Shellfish	26100110	Fish, NS as to type, cooked, NS as to cooking method
1253	A-14-0073	14-0073		Cranberries	18.01.00	Fruit - Fresh	63207010	Cranberries, raw
1254	A-00-00707			Cranberries, Raw	18.01.00	Fruit - Fresh	63207010	Cranberries, raw
1255	A-00-03850	00-03850		Cranberry juice	27.02.01	Beverages - Fruit based drinks - Pure fruit juice & smoothies	61210000	Orange juice, 100%, NFS
1256	A-17-0296	17-0296		Cranberry sauce	21.03.00	Sauces & accompaniment - Other sauces, incl. brown sauce, soy sauce, ketchup, mint sauce, vinegar	9081	Cranberry sauce, canned, sweetened
1257	A-00-01036			Cream Horn (Flaky Pastry with Fresh Cream and Jam)	04.02.00	Sweet cereal products - Pastries, Buns & Pies	53452420	Pastry, puff, custard or cream filled, iced or not iced
1258	A-12-0150	12-0150		Cream cheese	06.02.00	Dairy products - Cheese, incl. cottage cheese	14420200	Cheese spread, cream cheese, regular
1259	A-11-0167	11-0167		Cream crackers	25.04.00	Savoury Snacks - Savoury biscuits & crackers	54307000	Crackers, matzo
1260	A-00-06014			Cream crackers 50	25.04.00	Savoury Snacks - Savoury biscuits & crackers	54307000	Crackers, matzo
1261	A-11-0234	11-0234		Cream horns	04.02.00	Sweet cereal products - Pastries, Buns & Pies	53452420	Pastry, puff, custard or cream filled, iced or not iced
1262	A-00-06069			Cream horns 50	04.02.00	Sweet cereal products - Pastries, Buns & Pies	53452420	Pastry, puff, custard or cream filled, iced or not iced
1263	17-0242	A-17-0242		Cream liqueurs - Baileys Original Irish Cream	27.01.04	Beverages - Alcohol - Spirits & Liqueur	93501000	Brandy
1264	A-00-00145			Cream, Double, Summer	06.01.00	Dairy products - Cream & fromage frais	12130100	Cream, heavy

1265	A-00-00146			Cream, Double, Winter	06.01.00	Dairy products - Cream & fromage frais	12130100	Cream, heavy
1266	A-00-00142			Cream, Single, Summer	06.01.00	Dairy products - Cream & fromage frais	12110100	Cream, light
1267	A-00-00143			Cream, Single, Winter	06.01.00	Dairy products - Cream & fromage frais	12110100	Cream, light
1268	A-00-00150			Cream, Sterilized, Canned	06.01.00	Dairy products - Cream & fromage frais	12100100	Cream, NS as to light, heavy, or half and half
1269	A-12-0124			Cream, UHT, canned spray	06.01.00	Dairy products - Cream & fromage frais	12100100	Cream, NS as to light, heavy, or half and half
1270	12-0121	A-12-0121		Cream, UHT, half	06.01.00	Dairy products - Cream & fromage frais	12120100	Cream, half and half
1271	A-12-0122	12-0122		Cream, UHT, single	06.01.00	Dairy products - Cream & fromage frais	12110100	Cream, light
1272	12-0123	A-12-0123		Cream, UHT, whipping	06.01.00	Dairy products - Cream & fromage frais	12130100	Cream, heavy
1273	A-00-00148			Cream, Whipping, Summer	06.01.00	Dairy products - Cream & fromage frais	12130100	Cream, heavy
1274	A-00-00149			Cream, Whipping, Winter	06.01.00	Dairy products - Cream & fromage frais	12130100	Cream, heavy
1275	A-12-0117	12-0117		Cream, fresh, clotted	06.01.00	Dairy products - Cream & fromage frais	12130100	Cream, heavy
1276	A-12-0116			Cream, fresh, double	06.01.00	Dairy products - Cream & fromage frais	12130100	Cream, heavy
1277	12-0112	A-12-0112		Cream, fresh, half	06.01.00	Dairy products - Cream & fromage frais	12120100	Cream, half and half
1278	A-12-0113			Cream, fresh, single	06.01.00	Dairy products - Cream & fromage frais	12110100	Cream, light
1279	12-0114	A-12-0114		Cream, fresh, soured	06.01.00	Dairy products - Cream & fromage frais	12310100	Sour cream
1280	A-12-0115			Cream, fresh, whipping	06.01.00	Dairy products - Cream & fromage frais	12130100	Cream, heavy
1281	A-12-0120			Cream, sterilised, canned	06.01.00	Dairy products - Cream & fromage frais	12100100	Cream, NS as to light, heavy, or half and half

1282	A-12-0220	12-0220		Creme caramel	06.04.02	Dairy products - Ice cream & dairy desserts - reduced fat products	13210350	Flan
1283	12-0221	A-12-0221		Creme caramel, homemade	06.04.01	Dairy products - Ice cream & dairy desserts - full fat products	13210350	Flan
1284	17-0092	A-17-0092		Creme eggs	24.01.00	Confectionary - Chocolate based products	91746150	Easter egg, candy coated chocolate
1285	00-09762	A-00-09762		Creme fraiche	06.01.00	Dairy products - Cream & fromage frais	12130100	Cream, heavy
1286	00-09763	A-00-09763		Creme fraiche, half fat	06.01.00	Dairy products - Cream & fromage frais	12120100	Cream, half and half
1287	A-00-00060			Crispbread, Rye	03.05.00	Breads - Crisp Breads, e.g. Rivetas, Grissini, Toast Melba	54305010	Crackers, crispbread
1288	A-00-00061			Crispbread, Wheat, Starch Reduced	03.05.00	Breads - Crisp Breads, e.g. Rivetas, Grissini, Toast Melba	54305010	Crackers, crispbread
1289	11-0168	A-11-0168		Crispbread, rye	03.05.00	Breads - Crisp Breads, e.g. Rivetas, Grissini, Toast Melba	54305010	Crackers, crispbread
1290	A-00-09945			Crispbread, rye (MW6 Vit Eq)	03.05.00	Breads - Crisp Breads, e.g. Rivetas, Grissini, Toast Melba	54305010	Crackers, crispbread
1291	11-0198	A-11-0198		Crispie cakes	04.02.00	Sweet cereal products - Pastries, Buns & Pies	57339500	Cereal (Kellogg's Rice Krispies Treats Cereal)
1292	00-05852			Crisps, lightly salted eg M&S, Tesco Finest	25.01.00	Savoury Snacks - Potato based snacks	71200010	Potato chips, NFS
1293	A-00-01388			Crisps, lower fat	25.01.00	Savoury Snacks - Potato based snacks	71201050	Potato chips, reduced fat
1294	00-09585	A-00-09585		Croissant	04.02.00	Sweet cereal products - Pastries, Buns & Pies	51166000	Croissant
1295	A-11-0120			Croissants	01.04.00	Cereals & cereal dishes - Other cereals & dishes	51166000	Croissant
1296	11-0293	A-11-0293		Crumble, apple	04.03.00	Sweet cereal products - Cereal based puddings (not milk)	53415100	Crisp, apple, apple dessert
1297	A-00-06086			Crumble, apple 50	04.03.00	Sweet cereal products - Cereal based puddings (not milk)	53415100	Crisp, apple, apple dessert

1298	00-09613	A-00-09613	A-11-0294	Crumble, fruit	04.03.00	Sweet cereal products - Cereal based puddings (not milk)	53415100	Crisp, apple, apple dessert
1299	A-00-06087			Crumble, fruit 50	04.03.00	Sweet cereal products - Cereal based puddings (not milk)	53415100	Crisp, apple, apple dessert
1300	A-11-0295	11-0295		Crumble, fruit, wholemeal	04.03.00	Sweet cereal products - Cereal based puddings (not milk)	53415100	Crisp, apple, apple dessert
1301	A-11-0292			Crumble, with pie filling	04.03.00	Sweet cereal products - Cereal based puddings (not milk)	53415100	Crisp, apple, apple dessert
1302	A-00-01037			Crumpets, Pikelets	04.02.00	Sweet cereal products - Pastries, Buns & Pies	52101040	Crumpet
1303	A-00-09587	00-09587	A-11-0236	Crumpets, toasted	04.02.00	Sweet cereal products - Pastries, Buns & Pies	52101050	Crumpet, toasted
1304	A-00-03791			Crunch and Slim Biscuits 82/1433	04.01.00	Sweet cereal products - Biscuits	53201000	Cookie, NFS
1305	A-11-0131			Crunchy Nut Corn Flakes	02.03.00	Breakfast cereals - Other breakfast cereals - low fibre	57239100	Cereal (Kellogg's Honey Crunch Corn Flakes)
1306	A-00-03018	00-03018		Crunchy Nut Cornflakes, Kelloggs	02.03.00	Breakfast cereals - Other breakfast cereals - low fibre	57239100	Cereal (Kellogg's Honey Crunch Corn Flakes)
1307	00-03019	A-00-03019		Crunchy Oat Cereal, Own Brand	02.01.00	Breakfast cereals - Oat based cereals	57000100	Cereal, oat, NFS
1308	A-00-00597			Cucumber, Raw	15.04.00	Vegetables - Other	75111000	Cucumber, raw
1309	A-13-0233	13-0233		Cucumber, raw	15.04.00	Vegetables - Other	75111000	Cucumber, raw
1310	13-0820	A-13-0820		Cumin seeds	19.00.00	Nuts & Seeds (incl. peanut butter)	2014	Spices, cumin seed
1311	A-00-03784			Cup-a-Soup, all types, made up 82/1426	20.02.00	Soups - Dried	58400000	Soup, NFS
1312	A-00-00918			Curacao	27.01.04	Beverages - Alcohol - Spirits & Liqueur	93501000	Brandy
1313	A-00-01061			Curd Cheese	06.02.00	Dairy products - Cheese, incl. cottage cheese	14200100	Cheese, cottage, NFS
1314	A-00-03576	00-03576		Curly kale, boiled, unsalted water	15.02.00	Vegetables - Brassicacea	72119200	Kale, cooked, NS as to form, NS as to fat added in cooking

1315	A-00-00084			Currant Buns	04.02.00	Sweet cereal products - Pastries, Buns & Pies	51160100	Roll, sweet, cinnamon bun, no frosting
1316	A-11-0076	11-0076		Currant bread	03.04.00	Breads - Other bread	51129010	Bread, raisin
1317	A-00-06010			Currant bread 50	03.04.00	Breads - Other bread	51129010	Bread, raisin
1318	A-11-0077	11-0077		Currant bread, toasted	03.04.00	Breads - Other bread	51129020	Bread, raisin, toasted
1319	A-00-09591	00-09591		Currant bun	04.02.00	Sweet cereal products - Pastries, Buns & Pies	51160100	Roll, sweet, cinnamon bun, no frosting
1320	A-11-0237			Currant buns	04.02.00	Sweet cereal products - Pastries, Buns & Pies	51160100	Roll, sweet, cinnamon bun, no frosting
1321	A-00-06032			Currant buns 50	04.02.00	Sweet cereal products - Pastries, Buns & Pies	51160100	Roll, sweet, cinnamon bun, no frosting
1322	A-14-0074	14-0074		Currants	18.03.00	Fruit - Dried	62108100	Currants, dried
1323	A-00-00708			Currants, Black, Raw	18.01.00	Fruit - Fresh	63117010	Currants, raw
1324	A-00-00710			Currants, Black, Stewed with Sugar	18.02.00	Fruit - Canned & cooked	63101110	Applesauce, stewed apples, NS as to sweetened or unsweetened; sweetened, NS as to type of sweetener
1325	A-00-00709			Currants, Black, Stewed without Sugar	18.02.00	Fruit - Canned & cooked	63101110	Applesauce, stewed apples, NS as to sweetened or unsweetened; sweetened, NS as to type of sweetener
1326	A-00-00717			Currants, Dried	18.03.00	Fruit - Dried	62108100	Currants, dried
1327	A-00-00711			Currants, Red, Raw	18.01.00	Fruit - Fresh	63117010	Currants, raw
1328	A-00-00713			Currants, Red, Stewed with Sugar	18.02.00	Fruit - Canned & cooked	63101110	Applesauce, stewed apples, NS as to sweetened or unsweetened; sweetened, NS as to type of sweetener

1329	A-00-00712			Currants, Red, Stewed without Sugar	18.02.00	Fruit - Canned & cooked	63101110	Applesauce, stewed apples, NS as to sweetened or unsweetened; sweetened, NS as to type of sweetener
1330	A-00-00715			Currants, White, Stewed without Sugar	18.02.00	Fruit - Canned & cooked	63101110	Applesauce, stewed apples, NS as to sweetened or unsweetened; sweetened, NS as to type of sweetener
1331	A-00-00429			Curried Meat	10.01.00	Meat - red - Beef & veal & dishes	27116100	Beef curry
1332	00-03938			Curry Paste	26.01.00	Miscellaneous - Dried herbs & spices & pastes	2015	Spices, curry powder
1333	A-00-00958			Curry Powder	26.01.00	Miscellaneous - Dried herbs & spices & pastes	2015	Spices, curry powder
1334	A-00-01234			Curry Sauce	21.03.00	Sauces & accompaniment - Other sauces, incl. brown sauce, soy sauce, ketchup, mint sauce, vinegar	74402100	Salsa, NFS
1335	00-03942			Curry Sauce, cream based	21.02.00	Sauces & accompaniment - Cooking sauces, incl. gravies, pesto, cooking sauces for pasta and rice dishes	75440600	Vegetable curry
1336	A-00-03809			Curry Sauce, pkt made up 82/1452	21.02.00	Sauces & accompaniment - Cooking sauces, incl. gravies, pesto, cooking sauces for pasta and rice dishes	75440600	Vegetable curry
1337	00-03941			Curry Sauce, yogurt based eg Thai	21.02.00	Sauces & accompaniment - Cooking sauces, incl. gravies, pesto, cooking sauces for pasta and rice dishes	75440600	Vegetable curry

1338	13-0822	A-13-0822		Curry powder	26.01.00	Miscellaneous - Dried herbs & spices & pastes	2015	Spices, curry powder
1339	17-0298	A-17-0298		Curry sauce, canned	21.02.00	Sauces & accompaniment - Cooking sauces, incl. gravies, pesto, cooking sauces for pasta and rice dishes	75440600	Vegetable curry
1340	A-15-0096	15-0096		Curry, Bombay potato	17.01.00	Potatoes - Potatoes	75440600	Vegetable curry
1341	15-0122			Curry, lentil, red/masoor dahl and tomato, with vegetable oil	16.01.00	Pulses/Lentils - Pulses/lentils	41311020	Sambar, vegetable stew
1342	15-0126			Curry, lentil, red/masoor dahl and vegetable	16.01.00	Pulses/Lentils - Pulses/lentils	41311020	Sambar, vegetable stew
1343	15-0154			Curry, vegetable, Pakistani	15.04.00	Vegetables - Other	75440600	Vegetable curry
1344	A-15-0152			Curry, vegetable, in sweet sauce	15.04.00	Vegetables - Other	75440600	Vegetable curry
1345	A-15-0155	15-0155		Curry, vegetable, retail, with rice	15.04.00	Vegetables - Other	75440600	Vegetable curry
1346	15-0156	A-15-0156		Curry, vegetable, takeaway	15.04.00	Vegetables - Other	75440600	Vegetable curry
1347	00-05331			Custard Dairy Free, Low fat e.g. Provamel	04.04.00	Sweet cereal products - Milk based puddings	13210300	Custard
1348	A-00-00007			Custard Powder	01.04.00	Cereals & cereal dishes - Other cereals & dishes	20017	Corn flour, masa, enriched, white
1349	A-00-00103			Custard Tart	04.02.00	Sweet cereal products - Pastries, Buns & Pies	53344000	Pie, custard
1350	A-00-00102			Custard made with Powder	04.04.00	Sweet cereal products - Milk based puddings	13220110	Pudding, flavors other than chocolate, prepared from dry mix, milk added
1351	11-0013			Custard powder	01.04.00	Cereals & cereal dishes - Other cereals & dishes	20017	Corn flour, masa, enriched, white
1352	11-0238	A-11-0238		Custard tart, large	04.02.00	Sweet cereal products - Pastries, Buns & Pies	53344000	Pie, custard
1353	A-00-06070			Custard tart, large 50	04.02.00	Sweet cereal products - Pastries, Buns & Pies	53344000	Pie, custard

1354	11-0239	A-11-0239		Custard tarts, individual	04.02.00	Sweet cereal products - Pastries, Buns & Pies	53344070	Pie, custard, individual size or tart
1355	A-00-09957			Custard tarts, individual (MW6 Vit Eq)	04.02.00	Sweet cereal products - Pastries, Buns & Pies	53344070	Pie, custard, individual size or tart
1356	A-00-06033			Custard tarts, individual 50	04.02.00	Sweet cereal products - Pastries, Buns & Pies	53344070	Pie, custard, individual size or tart
1357	A-00-00101			Custard, Egg	04.04.00	Sweet cereal products - Milk based puddings	13210300	Custard
1358	A-12-0225			Custard, canned	04.04.00	Sweet cereal products - Milk based puddings	13210300	Custard
1359	A-00-09668			Custard, canned, or pots etc	04.04.00	Sweet cereal products - Milk based puddings	13210300	Custard
1360	00-09668			Custard, canned, or pots etc, any flavour	04.04.00	Sweet cereal products - Milk based puddings	13210300	Custard
1361	A-12-0226	12-0226		Custard, confectioners'	04.04.00	Sweet cereal products - Milk based puddings	13210300	Custard
1362	A-12-0227	12-0227		Custard, egg	04.04.00	Sweet cereal products - Milk based puddings	13210300	Custard
1363	A-12-0223			Custard, made up with semi-skimmed milk	04.04.00	Sweet cereal products - Milk based puddings	13210300	Custard
1364	A-12-0224			Custard, made up with skimmed milk	04.04.00	Sweet cereal products - Milk based puddings	13210300	Custard
1365	A-12-0222			Custard, made up with whole milk	04.04.00	Sweet cereal products - Milk based puddings	13210300	Custard
1366	A-00-09813	00-09813		Custard, made with semi-skim milk	04.04.00	Sweet cereal products - Milk based puddings	13210300	Custard
1367	00-09814	A-00-09814		Custard, made with skimmed milk	04.04.00	Sweet cereal products - Milk based puddings	13210300	Custard
1368	A-00-09812	00-09812		Custard, made with whole milk	04.04.00	Sweet cereal products - Milk based puddings	13210300	Custard
1369	00-09669	A-00-09669		Custard,low fat canned, or pots etc	04.04.00	Sweet cereal products - Milk based puddings	13210300	Custard
1370	A-00-03814			D/M Jam (M.N. 850) 82/1901	22.01.00	Preserves - Jam & Marmalade	91402000	Jam, preserve, all flavors

1371	A-00-03815			D/M Sweets (M.N. 862) 82/1903	24.02.00	Confectionary - Sugar based products	91700010	Candy, NFS
1372	02-07137			DALEPAK VEGETARIAN GRILLS	15.04.00	Vegetables - Other	41811890	Vegetarian burger or patty, meatless, no bun
1373	02-10075			DATE AND WALNUT LOAF PURCHASED, NOT LOW FAT	04.02.00	Sweet cereal products - Pastries, Buns & Pies	52405010	Bread, fruit
1374	17-0327			DO NOT USE Salad cream, reduced calorie	21.01.00	Sauces & accompaniment - Dressings & Mayonnaise	83200100	Salad dressing, light, NFS
1375	00-03450			DO NOT USEFat Spread (70% fat) Flora Buttery	08.04.01	Fats - Plant based fats (solid) - Full fat	81102000	Margarine, NFS
1376	00-03469			DO NOT USEFlora Pro.activ, 35% fat spread	08.04.03	Fats - Plant based fats (solid) - Low fat	81102000	Margarine, NFS
1377	02-03415			DORSET CEREAL WITH FRUIT AND NUTS	02.02.00	Breakfast cereals - Other breakfast cereals - high fibre (equal or >3g/40g portion)	57308190	Cereal, muesli
1378	02-10302			DORSET CEREALS WITH FRUIT	02.03.00	Breakfast cereals - Other breakfast cereals - low fibre	57308190	Cereal, muesli
1379	02-00325			DOUGHNUTS FRESH CREAM FILLED	04.02.00	Sweet cereal products - Pastries, Buns & Pies	53521210	Doughnut, custard-filled
1380	02-08788			DRIED BLUEBERRIES/BILBERRIES	18.03.00	Fruit - Dried	62105000	Blueberries, dried
1381	02-08134			DRIED CHERRIES	18.03.00	Fruit - Dried	62106000	Cherries, dried
1382	02-09764			DRIED MIXED HERBS	26.01.00	Miscellaneous - Dried herbs & spices & pastes	2027	Spices, oregano, dried
1383	02-08094			DRIED STRAWBERRIES	18.03.00	Fruit - Dried	62101000	Fruit, dried, NFS, uncooked
1384	02-09405			DUCK BRAISED MEAT ONLY	11.02.00	Meat - white - Other game birds, (e.g. duck, goose, pheasant) & dishes	24301020	Duck, roasted, skin not eaten
1385	02-05421			DUCK CRISPY CHINESE WITH PANCAKES SAUCE ONION	11.02.00	Meat - white - Other game birds, (e.g. duck, goose, pheasant) & dishes	24301210	Duck, coated, fried
1386	02-00074			DUMPLINGS MADE WITH ANIMAL SUET	01.04.00	Cereals & cereal dishes - Other cereals & dishes	55610300	Dumpling, plain

1387	02-08719			DUMPLINGS MADE WITH VEGETABLE SUET	01.04.00	Cereals & cereal dishes - Other cereals & dishes	55610300	Dumpling, plain
1388	A-12-0258			Dairy/fat spread	08.03.00	Fats - Animal based fats (solid)	81201000	Animal fat or drippings
1389	A-00-00718			Damsons, Raw	18.01.00	Fruit - Fresh	63143010	Plum, raw
1390	A-00-00719			Damsons, Raw (Weighed with Stones)	18.01.00	Fruit - Fresh	63143010	Plum, raw
1391	A-00-00722			Damsons, Stewed with Sugar	18.02.00	Fruit - Canned & cooked	63143110	Plum, cooked or canned, NS as to sweetened or unsweetened; sweetened, NS as to type of sweetener
1392	A-00-00720			Damsons, Stewed without Sugar	18.02.00	Fruit - Canned & cooked	63143110	Plum, cooked or canned, NS as to sweetened or unsweetened; sweetened, NS as to type of sweetener
1393	14-0077			Damsons, raw	18.01.00	Fruit - Fresh	63143010	Plum, raw
1394	14-0079	A-14-0079		Damsons, stewed with sugar	18.02.00	Fruit - Canned & cooked	63143110	Plum, cooked or canned, NS as to sweetened or unsweetened; sweetened, NS as to type of sweetener
1395	14-0081			Damsons, stewed without sugar	18.02.00	Fruit - Canned & cooked	63143110	Plum, cooked or canned, NS as to sweetened or unsweetened; sweetened, NS as to type of sweetener
1396	A-00-01035			Danish Pastry	04.02.00	Sweet cereal products - Pastries, Buns & Pies	53510000	Danish pastry, plain or spice
1397	A-11-0240	11-0240		Danish pastries	04.02.00	Sweet cereal products - Pastries, Buns & Pies	53510000	Danish pastry, plain or spice
1398	A-00-09958			Danish pastries (MW6 Vit Eq)	04.02.00	Sweet cereal products - Pastries, Buns & Pies	53510000	Danish pastry, plain or spice

1399	A-00-06034			Danish pastries 50	04.02.00	Sweet cereal products - Pastries, Buns & Pies	53510000	Danish pastry, plain or spice
1400	A-00-01242			Date, Fresh, Raw, Edible Portion	18.01.00	Fruit - Fresh	62110100	Date
1401	A-00-00724			Dates, Dried	18.03.00	Fruit - Dried	62110100	Date
1402	A-00-00725			Dates, Dried (Weighed with Stones)	18.03.00	Fruit - Dried	62110100	Date
1403	A-14-0085	14-0085		Dates, dried	18.03.00	Fruit - Dried	62110100	Date
1404	14-0083	A-14-0083		Dates, raw	18.01.00	Fruit - Fresh	62110100	Date
1405	A-12-0125			Dessert Top	06.01.00	Dairy products - Cream & fromage frais	12100100	Cream, NS as to light, heavy, or half and half
1406	A-00-01168			Desserts, Home-made, with Evaporated Milk	04.04.00	Sweet cereal products - Milk based puddings	13210150	Bread pudding made with evaporated milk and rum
1407	A-00-01167			Desserts, Home-made, with Fresh Cream	04.04.00	Sweet cereal products - Milk based puddings	53123070	Cake, shortcake, sponge type, with whipped cream and fruit
1408	00-05647			Diet Cherry Coke, cherry cola low calorie ONLY	27.03.00	Beverages - Carbonated soft drinks	92400000	Soft drink, NFS
1409	00-05401			Diet Ginger Beer eg Sainsburys	27.01.06	Beverages - Alcohol - Low alcohol beer	93101000	Beer
1410	A-00-03870	00-03870		Diet fizzy fruit drink eg Tango, fanta	27.03.00	Beverages - Carbonated soft drinks	92433000	Fruit juice drink, noncitrus, carbonated
1411	00-03848	A-00-03848		Diet lemonade	27.03.00	Beverages - Carbonated soft drinks	92432000	Fruit juice drink, citrus, carbonated
1412	11-0169	A-11-0169		Digestive biscuits, chocolate	04.01.00	Sweet cereal products - Biscuits	54102020	Graham crackers, chocolate covered
1413	A-00-09946			Digestive biscuits, chocolate (MW6 Vit Eq)	04.01.00	Sweet cereal products - Biscuits	54102020	Graham crackers, chocolate covered
1414	A-00-06016			Digestive biscuits, chocolate 50	04.01.00	Sweet cereal products - Biscuits	54102020	Graham crackers, chocolate covered
1415	11-0170	A-11-0170		Digestive biscuits, plain	04.01.00	Sweet cereal products - Biscuits	54102010	Graham crackers
1416	A-00-09947			Digestive biscuits, plain (MW6 Vit Eq)	04.01.00	Sweet cereal products - Biscuits	54102010	Graham crackers

1417	A-00-06054			Digestive biscuits, plain 50	04.01.00	Sweet cereal products - Biscuits	54102010	Graham crackers
1418	A-00-00512			Dogfish, Fried (Weighed with Waste)	09.01.00	Fish & fish dishes - White fish, incl. tuna	26141120	Sea bass, baked or broiled, fat added in cooking
1419	A-00-00511			Dogfish, Fried in Batter	09.01.00	Fish & fish dishes - White fish, incl. tuna	26141140	Sea bass, coated, fried
1420	00-05303			Dolmio Express Minced Beef Bolognese Sauce	21.02.00	Sauces & accompaniment - Cooking sauces, incl. gravies, pesto, cooking sauces for pasta and rice dishes	27162040	Spaghetti sauce with meat
1421	A-19-0130	19-0130		Doner kebab in pitta bread with salad	13.00.00	Sausages & burgers & kebab	27516010	Gyro sandwich (pita bread, beef, lamb, onion, condiments), with tomato and spread
1422	19-0129			Doner kebabs, meat only	13.00.00	Sausages & burgers & kebab	23120100	Lamb, roast, cooked, NS as to fat eaten
1423	00-05339			Dorset Cereal Berries and Cherries	02.03.00	Breakfast cereals - Other breakfast cereals - low fibre	57308190	Cereal, muesli
1424	00-05844			Dorset Cereals Tasty Fruit and Fibre	02.02.00	Breakfast cereals - Other breakfast cereals - high fibre (equal or >3g/40g portion)	57308190	Cereal, muesli
1425	A-00-00085			Doughnuts	04.02.00	Sweet cereal products - Pastries, Buns & Pies	53520000	Doughnut, NS as to cake or yeast
1426	A-11-0241	11-0241		Doughnuts, custard-filled	04.02.00	Sweet cereal products - Pastries, Buns & Pies	53521210	Doughnut, custard-filled
1427	A-11-0242	11-0242		Doughnuts, jam	04.02.00	Sweet cereal products - Pastries, Buns & Pies	53521140	Doughnut, jelly
1428	A-00-09961			Doughnuts, jam (MW6 Vit Eq)	04.02.00	Sweet cereal products - Pastries, Buns & Pies	53521140	Doughnut, jelly
1429	A-00-06035			Doughnuts, jam 50	04.02.00	Sweet cereal products - Pastries, Buns & Pies	53521140	Doughnut, jelly
1430	11-0243	A-11-0243		Doughnuts, ring	04.02.00	Sweet cereal products - Pastries, Buns & Pies	53521110	Doughnut, raised or yeast

1431	A-00-09959			Doughnuts, ring (MW6 Vit Eq)	04.02.00	Sweet cereal products - Pastries, Buns & Pies	53521110	Doughnut, raised or yeast
1432	A-00-06071			Doughnuts, ring 50	04.02.00	Sweet cereal products - Pastries, Buns & Pies	53521110	Doughnut, raised or yeast
1433	A-11-0244	11-0244		Doughnuts, ring, iced	04.02.00	Sweet cereal products - Pastries, Buns & Pies	53521110	Doughnut, raised or yeast
1434	A-00-09960			Doughnuts, ring, iced (MW6 Vit Eq)	04.02.00	Sweet cereal products - Pastries, Buns & Pies	53521110	Doughnut, raised or yeast
1435	00-05414			Doves Farm Buckwheat Flour	01.04.00	Cereals & cereal dishes - Other cereals & dishes	20080	Wheat flour, whole-grain
1436	A-12-0230			Dream Topping, made up with semi-skimmed milk	06.01.00	Dairy products - Cream & fromage frais	12100100	Cream, NS as to light, heavy, or half and half
1437	A-12-0231			Dream Topping, made up with skimmed milk	06.01.00	Dairy products - Cream & fromage frais	12100100	Cream, NS as to light, heavy, or half and half
1438	A-12-0229			Dream Topping, made up with whole milk	06.01.00	Dairy products - Cream & fromage frais	12100100	Cream, NS as to light, heavy, or half and half
1439	A-00-09815			Dream Topping, made with whole milk	06.01.00	Dairy products - Cream & fromage frais	12100100	Cream, NS as to light, heavy, or half and half
1440	A-00-09816	00-09816		Dream Topping, with semi-skimmed milk	06.01.00	Dairy products - Cream & fromage frais	12100100	Cream, NS as to light, heavy, or half and half
1441	A-00-03746			Dream topping (with cream) 82/1179	06.01.00	Dairy products - Cream & fromage frais	12100100	Cream, NS as to light, heavy, or half and half
1442	A-17-0301	17-0301		Dressing, 'fat free'	21.01.00	Sauces & accompaniment - Dressings & Mayonnaise	83300900	Salad dressing, fat free, NFS
1443	17-0302	A-17-0302		Dressing, French	21.01.00	Sauces & accompaniment - Dressings & Mayonnaise	83202020	French or Catalina dressing, light
1444	A-17-0303	17-0303		Dressing, French, homemade	21.01.00	Sauces & accompaniment - Dressings & Mayonnaise	83202020	French or Catalina dressing, light
1445	17-0300	A-17-0300		Dressing, blue cheese	21.01.00	Sauces & accompaniment - Dressings & Mayonnaise	83101000	Blue or roquefort cheese dressing
1446	17-0306	A-17-0306		Dressing, thousand island	21.01.00	Sauces & accompaniment - Dressings & Mayonnaise	83114000	Thousand Island dressing
1447	17-0307			Dressing, thousand island, reduced calorie	21.01.00	Sauces & accompaniment - Dressings & Mayonnaise	83207000	Thousand Island dressing, light

1448	00-05358			Dried Cranberries	18.03.00	Fruit - Dried	62109100	Cranberries, dried
1449	14-0087	A-14-0087		Dried mixed fruit	18.03.00	Fruit - Dried	62101050	Fruit mixture, dried
1450	12-0030	A-12-0030		Dried skimmed milk	05.01.00	Milk - Skimmed milk	11810000	Milk, dry, not reconstituted, NS as to fat content
1451	A-12-0031			Dried skimmed milk, with vegetable fat	05.01.00	Milk - Skimmed milk	11810000	Milk, dry, not reconstituted, NS as to fat content
1452	A-12-0032	12-0032		Dried whole milk	05.03.00	Milk - Whole milk	11810000	Milk, dry, not reconstituted, NS as to fat content
1453	A-00-00873			Drinking Chocolate	27.05.00	Beverages - Powdered Beverages (cocoa, Horlicks, Bonvita, Ovaltine, etc)	11830150	Cocoa powder, not reconstituted
1454	A-00-09696	00-09696		Drinking choc, low calorie, instant, made up with water	27.05.00	Beverages - Powdered Beverages (cocoa, Horlicks, Bonvita, Ovaltine, etc)	11830150	Cocoa powder, not reconstituted
1455	A-00-09805	00-09805		Drinking choc, with semi-skim milk	27.05.00	Beverages - Powdered Beverages (cocoa, Horlicks, Bonvita, Ovaltine, etc)	11514120	Hot chocolate / Cocoa, made with dry mix and reduced fat milk
1456	A-00-09806	00-09806		Drinking choc, with skimmed milk	27.05.00	Beverages - Powdered Beverages (cocoa, Horlicks, Bonvita, Ovaltine, etc)	11514140	Hot chocolate / Cocoa, made with dry mix and fat free milk
1457	A-00-09691	00-09691	A-12-0093	Drinking chocolate powder	27.05.00	Beverages - Powdered Beverages (cocoa, Horlicks, Bonvita, Ovaltine, etc)	11830150	Cocoa powder, not reconstituted
1458	A-12-0095			Drinking chocolate powder, made up with semi-skimmed milk	27.05.00	Beverages - Powdered Beverages (cocoa, Horlicks, Bonvita, Ovaltine, etc)	11514120	Hot chocolate / Cocoa, made with dry mix and reduced fat milk
1459	A-12-0096			Drinking chocolate powder, made up with skimmed milk	27.05.00	Beverages - Powdered Beverages (cocoa, Horlicks, Bonvita, Ovaltine, etc)	11514140	Hot chocolate / Cocoa, made with dry mix and fat free milk

1460	A-12-0094			Drinking chocolate powder, made up with whole milk	27.05.00	Beverages - Powdered Beverages (cocoa, Horlicks, Bonvita, Ovaltine, etc)	11514110	Hot chocolate / Cocoa, made with dry mix and whole milk
1461	00-09694	A-00-09694		Drinking chocolate powder, reduced fat	27.05.00	Beverages - Powdered Beverages (cocoa, Horlicks, Bonvita, Ovaltine, etc)	11830150	Cocoa powder, not reconstituted
1462	00-03879			Drinking chocolate, instant with water	27.05.00	Beverages - Powdered Beverages (cocoa, Horlicks, Bonvita, Ovaltine, etc)	11830150	Cocoa powder, not reconstituted
1463	00-09804	A-00-09804		Drinking chocolate, with whole milk	27.05.00	Beverages - Powdered Beverages (cocoa, Horlicks, Bonvita, Ovaltine, etc)	11514110	Hot chocolate / Cocoa, made with dry mix and whole milk
1464	12-0193	A-12-0193		Drinking yogurt	06.03.02	Dairy products - Yoghurt & drinking yoghurts, incl. buttermilk and probiotics - reduced or low fat products	11436000	Yogurt, liquid
1465	A-00-06154			Dripping 50	08.03.00	Fats - Animal based fats (solid)	81201000	Animal fat or drippings
1466	A-00-00184			Dripping, Beef	08.03.00	Fats - Animal based fats (solid)	81201000	Animal fat or drippings
1467	17-0006	A-17-0006		Dripping, beef	08.03.00	Fats - Animal based fats (solid)	81201000	Animal fat or drippings
1468	00-05543			Duchy Originals Spinach Soup	20.01.00	Soups - Canned & fresh & homemade	72307000	Spinach soup
1469	A-00-03745			Duck Fat (100% fat) 82/986	08.03.00	Fats - Animal based fats (solid)	81201000	Animal fat or drippings
1470	A-00-00327			Duck, Raw, Meat Only	11.02.00	Meat - white - Other game birds, (e.g. duck, goose, pheasant) & dishes	5141	Duck, domesticated, meat only, raw
1471	A-00-00329			Duck, Roast, Meat Only	11.02.00	Meat - white - Other game birds, (e.g. duck, goose, pheasant) & dishes	24301020	Duck, roasted, skin not eaten
1472	A-00-00330			Duck, Roast, Meat, Fat and Skin	11.02.00	Meat - white - Other game birds, (e.g. duck, goose, pheasant) & dishes	24301010	Duck, roasted, skin eaten
1473	18-0372	A-18-0372		Duck, roasted, meat only	11.02.00	Meat - white - Other game birds, (e.g. duck, goose, pheasant) & dishes	24301020	Duck, roasted, skin not eaten

1474	18-0374	A-18-0374		Duck, roasted, meat, fat and skin	11.02.00	Meat - white - Other game birds, (e.g. duck, goose, pheasant) & dishes	24301010	Duck, roasted, skin eaten
1475	A-00-00104			Dumpling	01.04.00	Cereals & cereal dishes - Other cereals & dishes	55610300	Dumpling, plain
1476	A-11-0340	11-0340		Dumplings	01.04.00	Cereals & cereal dishes - Other cereals & dishes	55610300	Dumpling, plain
1477	A-00-06053			Dumplings (dripping) 50	01.04.00	Cereals & cereal dishes - Other cereals & dishes	55610300	Dumpling, plain
1478	A-00-03598	00-03598		Dumplings, Vegetarian	01.04.00	Cereals & cereal dishes - Other cereals & dishes	55610300	Dumpling, plain
1479	00-03333			Dutch Crisp Bakes	03.05.00	Breads - Crisp Breads, e.g. Rivetas, Grissini, Toast Melba	54305010	Crackers, crispbread
1480	02-08066			EGG MAYONNAISE PURCHASED	07.00.00	Egg & egg dishes	83107000	Mayonnaise, regular
1481	02-00786			EGG WHITE ONLY BOILED	07.00.00	Egg & egg dishes	31108110	Egg, white, cooked, fat not added in cooking
1482	00-05822			ELECTRIFIRE Energy Drink	27.03.00	Beverages - Carbonated soft drinks	95310560	Energy drink (NOS)
1483	02-10448			ELMLEA LIGHT DOUBLE CREAM	06.01.00	Dairy products - Cream & fromage frais	12130100	Cream, heavy
1484	02-06828			ELMLEA LIGHT SINGLE CREAM	06.01.00	Dairy products - Cream & fromage frais	12110100	Cream, light
1485	02-09206			EXTRA LIGHT VERY LOW FAT MAYONNAISE RETAIL	21.01.00	Sauces & accompaniment - Dressings & Mayonnaise	83204030	Mayonnaise, reduced fat, with olive oil
1486	02-07982			EXTRA STRONG MINTS	24.02.00	Confectionary - Sugar based products	91700010	Candy, NFS
1487	A-00-01038			Eccles Cake	04.02.00	Sweet cereal products - Pastries, Buns & Pies	53309070	Pie, raisin, individual size or tart
1488	A-11-0245	11-0245		Eccles cake	04.02.00	Sweet cereal products - Pastries, Buns & Pies	53309070	Pie, raisin, individual size or tart
1489	A-00-06072			Eccles cake 50	04.02.00	Sweet cereal products - Pastries, Buns & Pies	53309070	Pie, raisin, individual size or tart

1490	A-00-00086			Eclairs	04.02.00	Sweet cereal products - Pastries, Buns & Pies	53521230	Doughnut, custard-filled, with icing
1491	11-0246	A-11-0246		Eclairs, fresh	04.02.00	Sweet cereal products - Pastries, Buns & Pies	53521230	Doughnut, custard-filled, with icing
1492	A-00-06073			Eclairs, fresh 50	04.02.00	Sweet cereal products - Pastries, Buns & Pies	53521230	Doughnut, custard-filled, with icing
1493	11-0247	A-11-0247		Eclairs, frozen	04.02.00	Sweet cereal products - Pastries, Buns & Pies	53521230	Doughnut, custard-filled, with icing
1494	A-00-00481			Eel, Stewed	09.02.00	Fish & fish dishes - Oily fish	26113160	Eel, steamed or poached
1495	16-0174	A-16-0174		Eel, jellied	09.02.00	Fish & fish dishes - Oily fish	26113160	Eel, steamed or poached
1496	A-00-01169			Egg Fried Rice	01.03.00	Cereals & cereal dishes - Rice & rice dishes	58150110	Rice, fried, meatless
1497	A-00-03800			Egg Mayonnaise 82/1442	07.00.00	Egg & egg dishes	83107000	Mayonnaise, regular
1498	A-00-01264			Egg Pilau	01.03.00	Cereals & cereal dishes - Rice & rice dishes	58150110	Rice, fried, meatless
1499	A-00-09523	A-12-0818	00-09523	Egg fried rice	01.03.00	Cereals & cereal dishes - Rice & rice dishes	58150110	Rice, fried, meatless
1500	A-12-0819	12-0819		Egg fu yung	07.00.00	Egg & egg dishes	32105200	Egg foo yung, NFS
1501	00-03553	A-00-03553		Egg mayonnaise, homemade	07.00.00	Egg & egg dishes	83107000	Mayonnaise, regular
1502	17-0243			Egg nog	05.06.00	Milk - Milk based drinks, e.g. flavoured milks	11531000	Eggnog, regular
1503	00-05646			Egg yolk fried in BVO, yolk only	07.00.00	Egg & egg dishes	31111020	Egg, yolk only, cooked, fat added in cooking
1504	A-00-00169			Egg, Boiled	07.00.00	Egg & egg dishes	31103010	Egg, whole, boiled or poached
1505	A-00-00168			Egg, Dried	07.00.00	Egg & egg dishes	1133	Egg, whole, dried
1506	A-00-00170			Egg, Fried	07.00.00	Egg & egg dishes	31105005	Egg, whole, fried, NS as to fat added in cooking
1507	A-00-00172			Egg, Omelette	07.00.00	Egg & egg dishes	32129990	Egg omelet or scrambled egg, NS as to fat added in cooking
1508	A-00-00171			Egg, Poached	07.00.00	Egg & egg dishes	31103010	Egg, whole, boiled or poached

1509	A-00-00173			Egg, Scrambled	07.00.00	Egg & egg dishes	32129990	Egg omelet or scrambled egg, NS as to fat added in cooking
1510	A-00-00166			Egg, White, Raw	07.00.00	Egg & egg dishes	31108010	Egg, white only, raw
1511	A-00-00165			Egg, Whole, Raw	07.00.00	Egg & egg dishes	31101010	Egg, whole, raw
1512	A-00-00167			Egg, Yolk, Raw	07.00.00	Egg & egg dishes	31110010	Egg, yolk only, raw
1513	00-05682			Egg, yolk only, boiled	07.00.00	Egg & egg dishes	31111010	Egg, yolk only, cooked, fat not added in cooking
1514	A-00-06100			Eggs, Chicken, Fried with Fat (bacon fat) 50	07.00.00	Egg & egg dishes	31105060	Egg, whole, fried with animal fat or meat drippings
1515	12-0806	A-12-0806		Eggs, chicken, boiled	07.00.00	Egg & egg dishes	31103010	Egg, whole, boiled or poached
1516	A-12-0808	12-0808		Eggs, chicken, fried, with fat	07.00.00	Egg & egg dishes	31105060	Egg, whole, fried with animal fat or meat drippings
1517	12-0809	A-12-0809		Eggs, chicken, fried, without fat	07.00.00	Egg & egg dishes	31105010	Egg, whole, fried without fat
1518	12-0810	A-12-0810		Eggs, chicken, poached	07.00.00	Egg & egg dishes	31103010	Egg, whole, boiled or poached
1519	12-0811	A-12-0811		Eggs, chicken, scrambled, with milk	07.00.00	Egg & egg dishes	32129990	Egg omelet or scrambled egg, NS as to fat added in cooking
1520	A-12-0812	12-0812		Eggs, chicken, scrambled, without milk	07.00.00	Egg & egg dishes	32129990	Egg omelet or scrambled egg, NS as to fat added in cooking
1521	12-0804	A-12-0804		Eggs, chicken, white, raw	07.00.00	Egg & egg dishes	31108010	Egg, white only, raw
1522	A-12-0801	12-0801		Eggs, chicken, whole, raw	07.00.00	Egg & egg dishes	31101010	Egg, whole, raw
1523	12-0805	A-12-0805		Eggs, chicken, yolk, raw	07.00.00	Egg & egg dishes	31110010	Egg, yolk only, raw
1524	A-12-0814	12-0814		Eggs, duck, boiled and salted	07.00.00	Egg & egg dishes	31103010	Egg, whole, boiled or poached
1525	00-09994			Eggs, scrambled with whole milk, no added salt	07.00.00	Egg & egg dishes	32129990	Egg omelet or scrambled egg, NS as to fat added in cooking

1526	00-05368			Elderflower Cordial, Diluted	27.02.03	Beverages - Fruit based drinks - Squashes & fruit concentrates	92510610	Fruit juice drink
1527	A-12-0128	00-09769	A-00-09769	Elmlea, double	06.01.00	Dairy products - Cream & fromage frais	12130100	Cream, heavy
1528	00-09767	A-00-09767	A-12-0126	Elmlea, single	06.01.00	Dairy products - Cream & fromage frais	12110100	Cream, light
1529	00-09768	A-00-09768	A-12-0127	Elmlea, whipping	06.01.00	Dairy products - Cream & fromage frais	12130100	Cream, heavy
1530	A-00-00598			Endive, Raw	15.04.00	Vegetables - Other	11213	Endive, raw
1531	13-0240	A-13-0240		Endive, raw	15.04.00	Vegetables - Other	11213	Endive, raw
1532	A-00-03779			Espagnole Sauce 82/1421	21.02.00	Sauces & accompaniment - Cooking sauces, incl. gravies, pesto, cooking sauces for pasta and rice dishes	28500010	Gravy, meat or poultry, with wine
1533	12-0033	A-12-0033		Evaporated milk, whole	05.03.00	Milk - Whole milk	11210050	Milk, evaporated, NS as to fat content
1534	A-00-09746	00-09746		Evaporated, light, milk, Carnation light	05.02.00	Milk - Semi-skimmed milk	11210050	Milk, evaporated, NS as to fat content
1535	11-0303	A-11-0303		Eve's pudding	04.03.00	Sweet cereal products - Cereal based puddings (not milk)	53119000	Cake, pineapple, upside down
1536	A-00-06088			Eve's pudding 50	04.03.00	Sweet cereal products - Cereal based puddings (not milk)	53119000	Cake, pineapple, upside down
1537	00-09761	A-00-09761		Extra thick dairy cream	06.01.00	Dairy products - Cream & fromage frais	12130100	Cream, heavy
1538	02-07689			FAIRY CAKE, PLAIN, ICED, PURCHASED, not chocolate eg cup cakes	04.02.00	Sweet cereal products - Pastries, Buns & Pies	53114100	Cake or cupcake, lemon, with icing or filling
1539	02-10040			FAT SPREAD (62-72% FAT) NOT POLYUNSATURATED	08.04.01	Fats - Plant based fats (solid) - Full fat	81102000	Margarine, NFS
1540	02-08080			FILO PASTRY PURCHASED READY MADE COOKED	01.04.00	Cereals & cereal dishes - Other cereals & dishes	53452400	Pastry, puff
1541	02-10296			FISHERMANS PIE REDUCED CALORIE AND FAT RETAIL	09.01.00	Fish & fish dishes - White fish, incl. tuna	28350050	Fish chowder

1542	02-03115			FLAKY / PUFF PASTRY PURCHASED, COOKED	01.04.00	Cereals & cereal dishes - Other cereals & dishes	53452400	Pastry, puff
1543	02-08160			FLAPJACK WITH CHOCOLATE PURCHASED	04.01.00	Sweet cereal products - Biscuits	53711000	Cereal or granola bar (Quaker Chewy Granola Bar)
1544	02-10052			FLORA EXTRA LIGHT	08.04.02	Fats - Plant based fats (solid) - Reduced fat	81102000	Margarine, NFS
1545	02-10046			FLORA NO SALT	08.04.03	Fats - Plant based fats (solid) - Low fat	81102000	Margarine, NFS
1546	02-02849			FLORA PRO ACTIV LIGHT SPREAD ONLY	08.04.03	Fats - Plant based fats (solid) - Low fat	81102000	Margarine, NFS
1547	02-10053			FLORA PRO ACTIV OLIVE OIL ONLY	08.04.01	Fats - Plant based fats (solid) - Full fat	81102000	Margarine, NFS
1548	02-07183			FLORA PRO ACTIV SKIMMED MILK	05.01.00	Milk - Skimmed milk	11100000	Milk, NFS
1549	02-00330			FLORENTINES	04.01.00	Sweet cereal products - Biscuits	53241600	Cookie, butter or sugar, with fruit and/or nuts
1550	02-00016			FLOUR WHITE SELF RAISING	01.04.00	Cereals & cereal dishes - Other cereals & dishes	20081	Wheat flour, white, all-purpose, enriched, bleached
1551	02-06974			FOCACCIA, PLAIN, GARLIC OR HERBS	03.04.00	Breads - Other bread	51108010	Focaccia, Italian flatbread, plain
1552	02-02704			FRENCH DRESSING-LOW FAT E.G. M&S	21.01.00	Sauces & accompaniment - Dressings & Mayonnaise	83202020	French or Catalina dressing, light
1553	02-08030			FRENCH FRIES EG WALKERS	25.01.00	Savoury Snacks - Potato based snacks	71205040	Potato sticks, fry shaped
1554	02-10147			FRESH DILL	26.01.00	Miscellaneous - Dried herbs & spices & pastes	11297	Parsley, fresh
1555	02-09824			FRESH EGG PASTA RAVIOLI (FILLED WITH MEAT)	01.02.00	Cereals & cereal dishes - Pasta & pasta dishes	58131310	Ravioli, meat-filled, no sauce
1556	02-05254			FROMAGE FRAIS, LOW FAT, FRUIT, UNFORTIFIED	06.01.00	Dairy products - Cream & fromage frais	14201200	Cottage cheese, farmer's
1557	02-08302			FRUIT AND NUT CHOCOLATE BAR	24.01.00	Confectionary - Chocolate based products	91705050	Milk chocolate candy, with fruit and nuts

1558	02-07082			FRUIT BASED PANNACOTTA	06.04.01	Dairy products - Ice cream & dairy desserts - full fat products	13120400	Ice cream bar or stick with fruit
1559	02-00262			FRUIT BISCUITS NOT WHOLEMEAL	04.01.00	Sweet cereal products - Biscuits	53237000	Cookie, raisin
1560	02-07915			FRUIT DRINK CONC B/C NOT LOW CAL, NOT ASDA OR SAINSBURY OWN BRANDS	27.02.03	Beverages - Fruit based drinks - Squashes & fruit concentrates	91301050	Fruit syrup
1561	02-08464			FRUIT DRINK CONC BLACKCURRANT LOW CALORIE	27.02.03	Beverages - Fruit based drinks - Squashes & fruit concentrates	91301050	Fruit syrup
1562	02-10231			FRUIT DRINK DOUBLE CONCENTRATED, NO ADDED SUGAR, NOT BLACKCURRANT	27.02.03	Beverages - Fruit based drinks - Squashes & fruit concentrates	91301050	Fruit syrup
1563	02-08144			FRUIT JUICE DRINK WITH 5% FRUIT JUICE RTD	27.02.02	Beverages - Fruit based drinks - Fruit juice drinks	92531030	Fruit juice drink (Sunny D)
1564	02-00108			FRUIT LOAF, PURCHASED	04.02.00	Sweet cereal products - Pastries, Buns & Pies	51129010	Bread, raisin
1565	02-00586			FRUIT PIE/FRIED EG. MC DONALDS	04.02.00	Sweet cereal products - Pastries, Buns & Pies	53301080	Pie, apple, fried pie
1566	02-07886			FRUIT SPREAD WITH EDIBLE SEEDS	22.01.00	Preserves - Jam & Marmalade	91402000	Jam, preserve, all flavors
1567	02-07887			FRUIT SPREADS STONE FRUIT	22.01.00	Preserves - Jam & Marmalade	91402000	Jam, preserve, all flavors
1568	02-09474			FRUIT SUGAR; FRUCTOSE EG. FRUISANA	23.01.00	Sugars - Pure sugars	91101000	Sugar, NFS
1569	02-00253			FULLY COATED CHOC BISCUITS WITH BISCUIT FILLING, GOLD BAR	04.01.00	Sweet cereal products - Biscuits	53209005	Cookie, chocolate, with icing or coating
1570	A-00-00402			Faggots	14.02.00	Offal - Other offal & dishes, e.g. Haggis, faggots	25120000	Heart, cooked
1571	A-19-0131	19-0131		Faggots in gravy, chilled/frozen, reheated	10.03.00	Meat - red - Pork & dishes	25170110	Tripe, cooked

1572	A-15-0162			Falafel, fried in vegetable oil	16.01.00	Pulses/Lentils - Pulses/lentils	41209000	Falafel
1573	00-03642			Falafel, oven baked (eg Cauldron organic)	16.01.00	Pulses/Lentils - Pulses/lentils	41209000	Falafel
1574	A-00-00074			Fancy Iced Cakes	04.02.00	Sweet cereal products - Pastries, Buns & Pies	53109200	Snack cake, not chocolate, with icing or filling
1575	A-11-0199	11-0199		Fancy iced cakes, individual	04.02.00	Sweet cereal products - Pastries, Buns & Pies	53109200	Snack cake, not chocolate, with icing or filling
1576	A-00-09953			Fancy iced cakes, individual (MW6 Vit Eq)	04.02.00	Sweet cereal products - Pastries, Buns & Pies	53109200	Snack cake, not chocolate, with icing or filling
1577	A-00-06023			Fancy iced cakes, individual 50	04.02.00	Sweet cereal products - Pastries, Buns & Pies	53109200	Snack cake, not chocolate, with icing or filling
1578	A-00-06003			Farex 50	02.02.00	Breakfast cereals - Other breakfast cereals - high fibre (equal or >3g/40g portion)	57805000	Rice cereal, baby food, dry, instant
1579	A-11-0132			Farmhouse Bran	02.02.00	Breakfast cereals - Other breakfast cereals - high fibre (equal or >3g/40g portion)	57100100	Cereal, ready-to-eat, NFS
1580	A-00-03020			Farmhouse Bran, Weetabix	02.02.00	Breakfast cereals - Other breakfast cereals - high fibre (equal or >3g/40g portion)	57100100	Cereal, ready-to-eat, NFS
1581	A-00-03465			Fat Spread (20-25%fat) Not polyunsaturated. EG Delight Diet	08.04.03	Fats - Plant based fats (solid) - Low fat	81102000	Margarine, NFS
1582	A-00-03460			Fat Spread (25-30%) St Ivel Gold Lowest (Extra Light) Very Low Fat Sp	08.04.03	Fats - Plant based fats (solid) - Low fat	81102000	Margarine, NFS
1583	A-00-03464			Fat Spread (35-40%fat) Polunsaturated. EG Flora Light	08.04.03	Fats - Plant based fats (solid) - Low fat	81102000	Margarine, NFS

1584	00-03464			Fat Spread (35-40%fat) Polunsaturated. NOT Flora Light	08.04.03	Fats - Plant based fats (solid) - Low fat	81102000	Margarine, NFS
1585	A-00-03459	00-03459		Fat Spread (40% fat) St Ivel Gold Light Low Fat Spread	08.04.03	Fats - Plant based fats (solid) - Low fat	81102000	Margarine, NFS
1586	A-00-03461			Fat Spread (40% fat) St Ivel Gold Unsalted Light Low Fat Spread	08.04.03	Fats - Plant based fats (solid) - Low fat	81102000	Margarine, NFS
1587	A-00-03462	00-03462		Fat Spread (40%) St Ivel Vitalite Light	08.04.03	Fats - Plant based fats (solid) - Low fat	81102000	Margarine, NFS
1588	00-03458	A-00-03458		Fat Spread (40%fat) Not Polyunsaturated. EG Delight Low Fat Spread	08.04.03	Fats - Plant based fats (solid) - Low fat	81102000	Margarine, NFS
1589	A-00-03457	00-03457		Fat Spread (55%fat) EG. Summer County	08.04.02	Fats - Plant based fats (solid) - Reduced fat	81102000	Margarine, NFS
1590	A-00-03452	00-03452		Fat Spread (60% fat) Utterly Butterly	08.04.02	Fats - Plant based fats (solid) - Reduced fat	81102000	Margarine, NFS
1591	A-00-03455			Fat Spread (60-65%fat) Vegetable Fat Spread. EG Krona 63%	08.04.01	Fats - Plant based fats (solid) - Full fat	81102000	Margarine, NFS
1592	00-03456	A-00-03456		Fat Spread (60-65%fat) With Olive Oil. EG Olivio	08.04.01	Fats - Plant based fats (solid) - Full fat	81102000	Margarine, NFS
1593	A-00-03450			Fat Spread (70% fat) Flora Buttery	08.04.01	Fats - Plant based fats (solid) - Full fat	81102000	Margarine, NFS
1594	A-00-03451			Fat Spread (70% fat) St Ivel Golden Churn	08.04.01	Fats - Plant based fats (solid) - Full fat	81102000	Margarine, NFS
1595	A-00-03453	00-03453		Fat Spread (70% fat) St Ivel Vitalite	08.04.01	Fats - Plant based fats (solid) - Full fat	81102000	Margarine, NFS
1596	00-03449	A-00-03449		Fat Spread (70%) Vegetable Fat Spread. EG I Can't Believe It's Not Butter	08.04.01	Fats - Plant based fats (solid) - Full fat	81102000	Margarine, NFS

1597	A-00-03448			Fat Spread (70%fat) Vegetable Fat Spread, Low salt. EG Flora Low salt	08.04.01	Fats - Plant based fats (solid) - Full fat	81102000	Margarine, NFS
1598	00-03448			Fat Spread (70%fat) Vegetable Fat Spread, Low salt. NOT Flora Low salt	08.04.01	Fats - Plant based fats (solid) - Full fat	81102000	Margarine, NFS
1599	A-00-03447			Fat Spread (70%fat) Vegetable Fat Spread. EG Flora Original	08.04.01	Fats - Plant based fats (solid) - Full fat	81102000	Margarine, NFS
1600	00-03447			Fat Spread (70%fat) Vegetable Fat Spread. NOT Flora Original	08.04.01	Fats - Plant based fats (solid) - Full fat	81102000	Margarine, NFS
1601	00-03445	A-00-03445		Fat Spread (70-80%) Not Polyunsaturated. EG krona Gold	08.04.01	Fats - Plant based fats (solid) - Full fat	81102000	Margarine, NFS
1602	00-03446			Fat Spread (75% fat) St Ivel Mono	08.04.01	Fats - Plant based fats (solid) - Full fat	81102000	Margarine, NFS
1603	A-00-03463	00-03463		Fat Spread(40%)Not Polyunsaturated.EG I Can't Beleive It's Not Butter	08.04.03	Fats - Plant based fats (solid) - Low fat	81102000	Margarine, NFS
1604	00-03468	A-00-03468		Fat Spread. Benecol Light	08.04.03	Fats - Plant based fats (solid) - Low fat	81102000	Margarine, NFS
1605	00-03467	A-00-03467		Fat Spread. Benecol Regular	08.04.01	Fats - Plant based fats (solid) - Full fat	81102000	Margarine, NFS
1606	00-03476			Fat spread (56%) Vitalite	08.04.02	Fats - Plant based fats (solid) - Reduced fat	81102000	Margarine, NFS
1607	17-0023			Fat spread (70% fat), polyunsat. inc. 'I Can't Believe...'	08.04.01	Fats - Plant based fats (solid) - Full fat	81102000	Margarine, NFS
1608	A-00-00183			Fat, Compound, Cooking	08.04.01	Fats - Plant based fats (solid) - Full fat	81102000	Margarine, NFS
1609	00-05164			Fennel Seeds	19.00.00	Nuts & Seeds (incl. peanut butter)	2014	Spices, cumin seed

1610	00-03581	A-00-03581		Fennel, Florence, boiled, unsalted water	15.04.00	Vegetables - Other	75215100	Fennel bulb, cooked, NS as to fat added in cooking
1611	A-13-0241	13-0241		Fennel, Florence, raw	15.04.00	Vegetables - Other	75109010	Fennel bulb, raw
1612	A-00-00727			Figs, Dried, Raw	18.03.00	Fruit - Dried	62113100	Fig, dried, uncooked
1613	A-00-00729			Figs, Dried, Stewed with Sugar	18.03.00	Fruit - Dried	62113230	Fig, dried, cooked, with sugar
1614	A-00-00728			Figs, Dried, Stewed without Sugar	18.03.00	Fruit - Dried	62113220	Fig, dried, cooked, unsweetened
1615	A-00-00726			Figs, Green, Raw	18.01.00	Fruit - Fresh	63119010	Fig, raw
1616	14-0092	A-14-0092		Figs, dried	18.03.00	Fruit - Dried	62113100	Fig, dried, uncooked
1617	14-0094			Figs, dried, stewed without sugar	18.03.00	Fruit - Dried	62113220	Fig, dried, cooked, unsweetened
1618	A-14-0091	14-0091		Figs, raw	18.01.00	Fruit - Fresh	63119010	Fig, raw
1619	14-0095	A-14-0095		Figs, ready-to-eat	18.01.00	Fruit - Fresh	63119010	Fig, raw
1620	00-05669			Filo wrapped prawns	09.03.00	Fish & fish dishes - Shellfish	26319161	Shrimp, coated, baked or broiled, made with butter
1621	A-00-03540			Findus crispy pancakes, all flavours	01.04.00	Cereals & cereal dishes - Other cereals & dishes	55501000	Chinese pancake
1622	A-00-03530			Findus, Pasta Choice meals	01.02.00	Cereals & cereal dishes - Pasta & pasta dishes	58146323	Pasta with tomato-based sauce and meat, ready-to-heat
1623	A-00-03525	00-03525		Findus, lean cuisine meals, any flavour	11.01.00	Meat - white - Chicken & turkey & dishes	28141250	Chicken with rice and vegetable, diet frozen meal
1624	A-00-00544			Fish Cakes, Fried	09.01.00	Fish & fish dishes - White fish, incl. tuna	27250030	Codfish ball or cake
1625	A-00-00543			Fish Cakes, Frozen	09.01.00	Fish & fish dishes - White fish, incl. tuna	27250030	Codfish ball or cake
1626	A-00-01284			Fish Curry	09.01.00	Fish & fish dishes - White fish, incl. tuna	27150320	Fish curry
1627	00-05219			Fish Curry, Cod/Haddock, with canned curry sauce	09.01.00	Fish & fish dishes - White fish, incl. tuna	27150320	Fish curry

1628	A-00-00546			Fish Fingers, Fried	09.01.00	Fish & fish dishes - White fish, incl. tuna	26100270	Fish stick, patty or nugget from restaurant, home, or other place
1629	A-00-00545			Fish Fingers, Frozen	09.01.00	Fish & fish dishes - White fish, incl. tuna	26100270	Fish stick, patty or nugget from restaurant, home, or other place
1630	A-00-01183			Fish Fingers, Grilled	09.01.00	Fish & fish dishes - White fish, incl. tuna	26100270	Fish stick, patty or nugget from restaurant, home, or other place
1631	A-00-00547			Fish Paste	26.01.00	Miscellaneous - Dried herbs & spices & pastes	15138	Crustaceans, crab, alaska king, imitation, made from surimi
1632	A-00-00548			Fish Pie	09.01.00	Fish & fish dishes - White fish, incl. tuna	28350050	Fish chowder
1633	16-0279			Fish balls, steamed	09.01.00	Fish & fish dishes - White fish, incl. tuna	27250030	Codfish ball or cake
1634	A-00-06256			Fish cakes, Fried (dripping) 50	09.01.00	Fish & fish dishes - White fish, incl. tuna	27250030	Codfish ball or cake
1635	16-0285	A-16-0285		Fish cakes, cod, homemade	09.01.00	Fish & fish dishes - White fish, incl. tuna	27250030	Codfish ball or cake
1636	A-16-0282	16-0282		Fish cakes, fried in blended oil	09.01.00	Fish & fish dishes - White fish, incl. tuna	27250030	Codfish ball or cake
1637	A-16-0284			Fish cakes, fried in sunflower oil	09.01.00	Fish & fish dishes - White fish, incl. tuna	27250030	Codfish ball or cake
1638	16-0281	A-16-0281		Fish cakes, grilled	09.01.00	Fish & fish dishes - White fish, incl. tuna	27250030	Codfish ball or cake
1639	A-16-0286	16-0286		Fish cakes, salmon, homemade	09.02.00	Fish & fish dishes - Oily fish	27250070	Salmon cake or patty
1640	A-16-0289			Fish fingers, cod, fried in blended oil	09.01.00	Fish & fish dishes - White fish, incl. tuna	26100270	Fish stick, patty or nugget from restaurant, home, or other place
1641	16-0291	A-16-0291		Fish fingers, cod, fried in sunflower oil	09.01.00	Fish & fish dishes - White fish, incl. tuna	26100270	Fish stick, patty or nugget from restaurant, home, or other place

1642	16-0288	A-16-0288		Fish fingers, cod, grilled	09.01.00	Fish & fish dishes - White fish, incl. tuna	26100270	Fish stick, patty or nugget from restaurant, home, or other place
1643	00-03935			Fish or Seafood soup	20.01.00	Soups - Canned & fresh & homemade	28355450	Seafood soup with potatoes and vegetables including carrots, broccoli, and/or dark-green leafy
1644	16-0293			Fish paste	26.01.00	Miscellaneous - Dried herbs & spices & pastes	15138	Crustaceans, crab, alaska king, imitation, made from surimi
1645	A-00-09966			Fish paste (MW6 iron)	26.01.00	Miscellaneous - Dried herbs & spices & pastes	15138	Crustaceans, crab, alaska king, imitation, made from surimi
1646	A-16-0294	16-0294		Fish pie	09.01.00	Fish & fish dishes - White fish, incl. tuna	28350050	Fish chowder
1647	A-00-03595			Fish stew	09.01.00	Fish & fish dishes - White fish, incl. tuna	27350110	Bouillabaisse
1648	16-0295	A-16-0295		Fisherman's pie, retail	09.01.00	Fish & fish dishes - White fish, incl. tuna	28350050	Fish chowder
1649	00-05687			Fitnesse breakfast cereal Nestle	02.03.00	Breakfast cereals - Other breakfast cereals - low fibre	57100100	Cereal, ready-to-eat, NFS
1650	11-0223	A-11-0223		Flaky pastry, cooked	01.04.00	Cereals & cereal dishes - Other cereals & dishes	53452400	Pastry, puff
1651	A-00-06065			Flaky pastry, cooked 50	04.02.00	Sweet cereal products - Pastries, Buns & Pies	53344070	Pie, custard, individual size or tart
1652	15-0163	A-15-0163		Flan, broccoli	15.02.00	Vegetables - Brassicacea	72125240	Spinach souffle
1653	A-15-0164			Flan, broccoli, wholemeal	15.02.00	Vegetables - Brassicacea	72125240	Spinach souffle
1654	A-15-0167			Flan, cheese and mushroom	06.02.00	Dairy products - Cheese, incl. cottage cheese	14630200	Cheese souffle
1655	15-0169	A-15-0169		Flan, cheese, onion and potato	06.02.00	Dairy products - Cheese, incl. cottage cheese	14630200	Cheese souffle

1656	15-0172			Flan, lentil and tomato, wholemeal	16.01.00	Pulses/Lentils - Pulses/lentils	58100020	Burrito, taco, or quesadilla with egg, beans, and breakfast meat
1657	A-00-06089			Flan, pastry with fruit 50	04.02.00	Sweet cereal products - Pastries, Buns & Pies	53344200	Mixed fruit tart filled with custard or cream cheese
1658	A-11-0304	11-0304		Flan, pastry, with fruit	04.02.00	Sweet cereal products - Pastries, Buns & Pies	53344200	Mixed fruit tart filled with custard or cream cheese
1659	A-15-0173			Flan, spinach	15.03.00	Vegetables - Yellow & red & dark green leafy vegetables	72125240	Spinach souffle
1660	A-15-0174			Flan, spinach, wholemeal	15.03.00	Vegetables - Yellow & red & dark green leafy vegetables	72125240	Spinach souffle
1661	A-00-06090			Flan, sponge with fruit 50	04.02.00	Sweet cereal products - Pastries, Buns & Pies	53123080	Cake, shortcake, sponge type, with fruit
1662	11-0305	A-11-0305		Flan, sponge, with fruit	04.02.00	Sweet cereal products - Pastries, Buns & Pies	53123080	Cake, shortcake, sponge type, with fruit
1663	15-0175	A-15-0175		Flan, vegetable	15.04.00	Vegetables - Other	72125240	Spinach souffle
1664	A-15-0176			Flan, vegetable, wholemeal	15.04.00	Vegetables - Other	72125240	Spinach souffle
1665	A-00-01027	11-0171	A-11-0171	Flapjacks	04.01.00	Sweet cereal products - Biscuits	53711000	Cereal or granola bar (Quaker Chewy Granola Bar)
1666	A-00-06055			Flapjacks 50	04.01.00	Sweet cereal products - Biscuits	53711000	Cereal or granola bar (Quaker Chewy Granola Bar)
1667	00-05787			Flat Bread, flavoured, fat free	03.05.00	Breads - Crisp Breads, e.g. Rivetas, Grissini, Toast Melba	54305020	Crackers, flatbread
1668	A-12-0034			Flavoured milk	05.06.00	Milk - Milk based drinks, e.g. flavoured milks	11100000	Milk, NFS
1669	00-09754	A-00-09754		Flavoured milk, chocolate	05.06.00	Milk - Milk based drinks, e.g. flavoured milks	11100000	Milk, NFS

1670	00-09755	A-00-09755		Flavoured milk, not chocolate	05.06.00	Milk - Milk based drinks, e.g. flavoured milks	11100000	Milk, NFS
1671	00-05465			Flora Omega 3 Plus Spread	08.04.02	Fats - Plant based fats (solid) - Reduced fat	81102000	Margarine, NFS
1672	16-0041			Flounder, steamed	09.01.00	Fish & fish dishes - White fish, incl. tuna	26115160	Flounder, steamed or poached
1673	A-00-00010			Flour, Brown (85%)	01.04.00	Cereals & cereal dishes - Other cereals & dishes	20080	Wheat flour, whole-grain
1674	00-01235			Flour, Maize	01.04.00	Cereals & cereal dishes - Other cereals & dishes	20017	Corn flour, masa, enriched, white
1675	A-00-00011			Flour, White (72%), Breadmaking	01.04.00	Cereals & cereal dishes - Other cereals & dishes	20081	Wheat flour, white, all-purpose, enriched, bleached
1676	A-00-00012			Flour, White, Household, Plain	01.04.00	Cereals & cereal dishes - Other cereals & dishes	20081	Wheat flour, white, all-purpose, enriched, bleached
1677	A-00-00014			Flour, White, Patent (40%)	01.04.00	Cereals & cereal dishes - Other cereals & dishes	20081	Wheat flour, white, all-purpose, enriched, bleached
1678	A-00-00013			Flour, White, Self-raising	01.04.00	Cereals & cereal dishes - Other cereals & dishes	20081	Wheat flour, white, all-purpose, enriched, bleached
1679	A-00-00009			Flour, Wholemeal (100%)	01.04.00	Cereals & cereal dishes - Other cereals & dishes	20080	Wheat flour, whole-grain
1680	17-0106			Foam sweets	24.02.00	Confectionary - Sugar based products	91700010	Candy, NFS
1681	01-00010			Formula 1 Protein drink HERBALIFE	30.00.00	Nutrition Powders & drinks	95201500	Nutritional powder mix, high protein (Herbalife)
1682	A-19-0100	19-0100		Frankfurter	13.00.00	Sausages & burgers & kebab	25210110	Frankfurter, wiener, or hot dog, NFS
1683	A-00-00405			Frankfurters	13.00.00	Sausages & burgers & kebab	25210110	Frankfurter, wiener, or hot dog, NFS
1684	00-09574	A-00-09574		French Baguette	03.01.00	Breads - White	51107010	Bread, French or Vienna

1685	A-00-00925			French Dressing	21.01.00	Sauces & accompaniment - Dressings & Mayonnaise	83202020	French or Catalina dressing, light
1686	A-00-03321	00-03321		French Toast	03.04.00	Breads - Other bread	55300010	French toast, NFS
1687	A-00-09576	00-09576		French baton, malted wheat (granary)	03.01.00	Breads - White	51107010	Bread, French or Vienna
1688	17-0256	A-17-0256		French onion soup	20.01.00	Soups - Canned & fresh & homemade	75608100	Onion soup, French
1689	00-05385			Fresh cheese sauce	21.02.00	Sauces & accompaniment - Cooking sauces, incl. gravies, pesto, cooking sauces for pasta and rice dishes	14650100	Cheese sauce
1690	00-09760	A-00-09760		Fresh cream, double (summer & winter)	06.01.00	Dairy products - Cream & fromage frais	12130100	Cream, heavy
1691	A-00-09758	00-09758		Fresh cream, single (summer & winter)	06.01.00	Dairy products - Cream & fromage frais	12110100	Cream, light
1692	A-00-09759	00-09759		Fresh cream, whipping(summer & winter)	06.01.00	Dairy products - Cream & fromage frais	12130100	Cream, heavy
1693	A-00-01170			Fried Rices, other than Egg	01.03.00	Cereals & cereal dishes - Rice & rice dishes	58150310	Rice, fried, NFS
1694	A-00-03811			Fried rice, no additions 82/1454	01.03.00	Cereals & cereal dishes - Rice & rice dishes	58150310	Rice, fried, NFS
1695	A-12-0159	00-09659	A-00-09659	Fromage frais, fruit	06.01.00	Dairy products - Cream & fromage frais	14201200	Cottage cheese, farmer's
1696	00-09658	A-12-0158	A-00-09658	Fromage frais, plain	06.01.00	Dairy products - Cream & fromage frais	14201200	Cottage cheese, farmer's
1697	12-0160	A-12-0160		Fromage frais, very low fat	06.01.00	Dairy products - Cream & fromage frais	14201200	Cottage cheese, farmer's
1698	00-09657	A-00-09657		Fromage frais, very low fat + fruit	06.01.00	Dairy products - Cream & fromage frais	14201200	Cottage cheese, farmer's
1699	A-00-09656	00-09656		Fromage frais, very low fat,natural	06.01.00	Dairy products - Cream & fromage frais	14201200	Cottage cheese, farmer's

1700	00-05605			Frosted Shreddies, Nestle	02.02.00	Breakfast cereals - Other breakfast cereals - high fibre (equal or >3g/40g portion)	57348000	Cereal, frosted corn flakes
1701	A-11-0133			Frosties	02.03.00	Breakfast cereals - Other breakfast cereals - low fibre	57348000	Cereal, frosted corn flakes
1702	00-03060			Frosties Cereal Milk Bar, Kelloggs	04.05.00	Sweet cereal products - Cereal bars	53710600	Milk 'n Cereal bar
1703	00-03022	A-00-03022		Frosties, Kelloggs	02.03.00	Breakfast cereals - Other breakfast cereals - low fibre	57348000	Cereal, frosted corn flakes
1704	A-00-03023	00-03023		Frosties, Own Brand	02.03.00	Breakfast cereals - Other breakfast cereals - low fibre	57348000	Cereal, frosted corn flakes
1705	A-12-0203			Frozen ice cream desserts	06.04.02	Dairy products - Ice cream & dairy desserts - reduced fat products	13110000	Ice cream, NFS
1706	A-00-09626	00-09626		Frozen ice cream desserts, choc/toffee, Vienetta	06.04.02	Dairy products - Ice cream & dairy desserts - reduced fat products	13110110	Ice cream, regular, chocolate
1707	00-05433			Frubes (Tube)	06.01.00	Dairy products - Cream & fromage frais	11411200	Yogurt, low fat milk, plain
1708	A-11-0134			Fruit 'n Fibre	02.03.00	Breakfast cereals - Other breakfast cereals - low fibre	57219000	Cereal, fruit and fiber
1709	A-00-01055			Fruit Fritters	18.02.00	Fruit - Canned & cooked	53415120	Fritter, apple
1710	A-00-00862			Fruit Gums	24.02.00	Confectionary - Sugar based products	91700010	Candy, NFS
1711	A-00-03826			Fruit Loaf U/R 82/1932	03.04.00	Breads - Other bread	52405010	Bread, fruit
1712	A-00-00730			Fruit Pie Filling, Canned	18.02.00	Fruit - Canned & cooked	63203700	Blueberry pie filling
1713	A-00-01052			Fruit Pie, (2 Crusts, Plain Flour)	04.02.00	Sweet cereal products - Pastries, Buns & Pies	53301000	Pie, apple, two crust
1714	A-00-01053			Fruit Pie, (2 Crusts, Wholemeal Flour)	04.02.00	Sweet cereal products - Pastries, Buns & Pies	53301000	Pie, apple, two crust
1715	A-00-01031			Fruit Pie, 1 Crust, Wholemeal Flour	04.02.00	Sweet cereal products - Pastries, Buns & Pies	53301500	Pie, apple, one crust
1716	A-00-00105			Fruit Pie, Individual with Pastry Top and Bottom	04.02.00	Sweet cereal products - Pastries, Buns & Pies	53301070	Pie, apple, individual size or tart

1717	A-00-00106			Fruit Pie, with Pastry Top	04.02.00	Sweet cereal products - Pastries, Buns & Pies	53301500	Pie, apple, one crust
1718	A-00-00731			Fruit Salad, Canned	18.02.00	Fruit - Canned & cooked	63311110	Fruit cocktail, cooked or canned, NS as to sweetened or unsweetened; sweetened, NS as to type of sweetener
1719	A-00-01124			Fruit Salad, Canned, without Sugar	18.02.00	Fruit - Canned & cooked	63311110	Fruit cocktail, cooked or canned, NS as to sweetened or unsweetened; sweetened, NS as to type of sweetener
1720	A-00-01123			Fruit Salad, Fresh	18.01.00	Fruit - Fresh	63311000	Fruit salad, fresh or raw, excluding citrus fruits, no dressing
1721	A-00-01059			Fruit Sponge Pudding (spotted Dick)	04.03.00	Sweet cereal products - Cereal based puddings (not milk)	53110000	Cake, fruit cake, light or dark, holiday type cake
1722	A-00-01054			Fruit Sponge, Eve's Pudding, Pineapple Upside Down Cake	04.03.00	Sweet cereal products - Cereal based puddings (not milk)	53119000	Cake, pineapple, upside down
1723	A-00-01149			Fruit Squash, Low Calorie, Diluted	27.02.03	Beverages - Fruit based drinks - Squashes & fruit concentrates	92510610	Fruit juice drink
1724	A-00-01148			Fruit Squash, Low Calorie, Undiluted	27.02.03	Beverages - Fruit based drinks - Squashes & fruit concentrates	91301050	Fruit syrup
1725	A-00-01127			Fruit Squash, Low Calorie, with Added Vitamin C, Diluted 1:4	27.02.03	Beverages - Fruit based drinks - Squashes & fruit concentrates	92510610	Fruit juice drink
1726	A-00-01150			Fruit Squash, Low Calorie, with Added Vitamin C, Undiluted	27.02.03	Beverages - Fruit based drinks - Squashes & fruit concentrates	91301050	Fruit syrup
1727	A-00-01351			Fruit Squash,High Juice Diluted 1:4	27.02.03	Beverages - Fruit based drinks - Squashes & fruit concentrates	92510610	Fruit juice drink

1728	A-00-01350			Fruit Squash,High Juice (40% Juice) Undiluted	27.02.03	Beverages - Fruit based drinks - Squashes & fruit concentrates	91301050	Fruit syrup
1729	A-00-03025	00-03025		Fruit and Fibre (Optima), Kelloggs	02.03.00	Breakfast cereals - Other breakfast cereals - low fibre	57219000	Cereal, fruit and fiber
1730	00-03024	A-00-03024		Fruit and Fibre, Own Brand	02.03.00	Breakfast cereals - Other breakfast cereals - low fibre	57219000	Cereal, fruit and fiber
1731	A-00-03823			Fruit and Nut Loaf U/R 82/1926	03.04.00	Breads - Other bread	52405010	Bread, fruit
1732	A-00-03233			Fruit based dairy dessert	06.04.02	Dairy products - Ice cream & dairy desserts - reduced fat products	13120400	Ice cream bar or stick with fruit
1733	11-0200	A-11-0200		Fruit cake, plain, retail	04.02.00	Sweet cereal products - Pastries, Buns & Pies	53110000	Cake, fruit cake, light or dark, holiday type cake
1734	A-00-09954			Fruit cake, plain, retail (MW6 Vit Eq)	04.02.00	Sweet cereal products - Pastries, Buns & Pies	53110000	Cake, fruit cake, light or dark, holiday type cake
1735	A-00-06024			Fruit cake, plain, retail 50	04.02.00	Sweet cereal products - Pastries, Buns & Pies	53110000	Cake, fruit cake, light or dark, holiday type cake
1736	11-0201	A-11-0201		Fruit cake, rich	04.02.00	Sweet cereal products - Pastries, Buns & Pies	53110000	Cake, fruit cake, light or dark, holiday type cake
1737	11-0203	A-11-0203		Fruit cake, rich, iced	04.02.00	Sweet cereal products - Pastries, Buns & Pies	53110000	Cake, fruit cake, light or dark, holiday type cake
1738	A-00-06025			Fruit cake, rich, iced 50	04.02.00	Sweet cereal products - Pastries, Buns & Pies	53110000	Cake, fruit cake, light or dark, holiday type cake
1739	11-0202	A-11-0202		Fruit cake, rich, retail	04.02.00	Sweet cereal products - Pastries, Buns & Pies	53110000	Cake, fruit cake, light or dark, holiday type cake
1740	A-11-0204	11-0204		Fruit cake, wholemeal	04.02.00	Sweet cereal products - Pastries, Buns & Pies	53110000	Cake, fruit cake, light or dark, holiday type cake
1741	A-00-06026			Fruit cake, wholemeal 50	04.02.00	Sweet cereal products - Pastries, Buns & Pies	53110000	Cake, fruit cake, light or dark, holiday type cake
1742	A-14-0096	14-0096		Fruit cocktail, canned in juice	18.02.00	Fruit - Canned & cooked	63311170	Fruit cocktail, cooked or canned, juice pack
1743	A-14-0097	14-0097		Fruit cocktail, canned in syrup	18.02.00	Fruit - Canned & cooked	63311140	Fruit cocktail, cooked or canned, in light syrup

1744	00-05835			Fruit cordials, not low cal eg Belvoir	27.02.03	Beverages - Fruit based drinks - Squashes & fruit concentrates	91301050	Fruit syrup
1745	A-00-03824			Fruit crumble (apple) U/R 82/1928	04.03.00	Sweet cereal products - Cereal based puddings (not milk)	53415100	Crisp, apple, apple dessert
1746	00-05909			Fruit drink, blackcurrant flavour, with multivitamins NAS, made up eg C-Vit	27.02.03	Beverages - Fruit based drinks - Squashes & fruit concentrates	92510610	Fruit juice drink
1747	A-17-0191			Fruit drink, low calorie, concentrated	27.02.03	Beverages - Fruit based drinks - Squashes & fruit concentrates	91301050	Fruit syrup
1748	A-17-0192	17-0192		Fruit drink, low calorie, concentrated, made up	27.02.03	Beverages - Fruit based drinks - Squashes & fruit concentrates	92510610	Fruit juice drink
1749	A-17-0193			Fruit drink, low sugar, concentrated, fortified	27.02.03	Beverages - Fruit based drinks - Squashes & fruit concentrates	91301050	Fruit syrup
1750	A-17-0194	17-0194		Fruit drink, low sugar, concentrated, fortified, made up	27.02.03	Beverages - Fruit based drinks - Squashes & fruit concentrates	92510610	Fruit juice drink
1751	17-0189	A-17-0189		Fruit drink/squash, concentrated	27.02.03	Beverages - Fruit based drinks - Squashes & fruit concentrates	91301050	Fruit syrup
1752	A-17-0190	17-0190		Fruit drink/squash, concentrated, made up	27.02.03	Beverages - Fruit based drinks - Squashes & fruit concentrates	92510610	Fruit juice drink
1753	A-00-03770			Fruit flan, sponge 82/1409	04.03.00	Sweet cereal products - Cereal based puddings (not milk)	53123080	Cake, shortcake, sponge type, with fruit
1754	A-00-09665	A-12-0232	00-09665	Fruit fool	06.04.01	Dairy products - Ice cream & dairy desserts - full fat products	13121100	Ice cream sundae, fruit topping, with whipped cream
1755	A-00-09666			Fruit fool, low fat	06.04.02	Dairy products - Ice cream & dairy desserts - reduced fat products	13140630	Light ice cream, sundae, soft serve, fruit topping, with whipped cream
1756	00-05519			Fruit fritters, fried in veg oil, from takeaway	18.02.00	Fruit - Canned & cooked	53415120	Fritter, apple
1757	00-05518			Fruit fritters, fried in veg oil, not from takeaway	18.02.00	Fruit - Canned & cooked	53415120	Fritter, apple
1758	17-0107	A-17-0107		Fruit gums/jellies	24.02.00	Confectionary - Sugar based products	91700010	Candy, NFS

1759	A-17-0177	17-0177		Fruit juice drink, carbonated, ready to drink	27.03.00	Beverages - Carbonated soft drinks	92433000	Fruit juice drink, noncitrus, carbonated
1760	17-0195	A-17-0195		Fruit juice drink, ready to drink	27.02.02	Beverages - Fruit based drinks - Fruit juice drinks	92531030	Fruit juice drink (Sunny D)
1761	A-17-0108	17-0108		Fruit pastilles	24.02.00	Confectionary - Sugar based products	91700010	Candy, NFS
1762	A-00-03149	00-03149		Fruit pie bars, Bramley Apple, Mr Kipling	04.02.00	Sweet cereal products - Pastries, Buns & Pies	53220000	Cookie, fruit-filled bar
1763	14-0098			Fruit pie filling	18.02.00	Fruit - Canned & cooked	63203700	Blueberry pie filling
1764	A-11-0310	11-0310		Fruit pie, individual	04.02.00	Sweet cereal products - Pastries, Buns & Pies	53301070	Pie, apple, individual size or tart
1765	A-00-09963			Fruit pie, individual (MW6 Vit Eq)	04.02.00	Sweet cereal products - Pastries, Buns & Pies	53301070	Pie, apple, individual size or tart
1766	A-11-0308	11-0308		Fruit pie, one crust	04.02.00	Sweet cereal products - Pastries, Buns & Pies	53301500	Pie, apple, one crust
1767	11-0309	A-11-0309		Fruit pie, pastry top and bottom	04.02.00	Sweet cereal products - Pastries, Buns & Pies	53301000	Pie, apple, two crust
1768	A-00-06354			Fruit pie, pastry top and bottom 50	04.02.00	Sweet cereal products - Pastries, Buns & Pies	53301000	Pie, apple, two crust
1769	A-11-0312			Fruit pie, wholemeal, pastry top and bottom	04.02.00	Sweet cereal products - Pastries, Buns & Pies	53301000	Pie, apple, two crust
1770	A-00-06353			Fruit pie. one crust 50	04.02.00	Sweet cereal products - Pastries, Buns & Pies	53301500	Pie, apple, one crust
1771	14-0099	A-14-0099		Fruit salad, homemade	18.01.00	Fruit - Fresh	63311000	Fruit salad, fresh or raw, excluding citrus fruits, no dressing
1772	A-00-00077			Fruitcake, Plain	04.02.00	Sweet cereal products - Pastries, Buns & Pies	53110000	Cake, fruit cake, light or dark, holiday type cake
1773	A-00-00075			Fruitcake, Rich	04.02.00	Sweet cereal products - Pastries, Buns & Pies	53110000	Cake, fruit cake, light or dark, holiday type cake
1774	A-00-00076			Fruitcake, Rich, Iced	04.02.00	Sweet cereal products - Pastries, Buns & Pies	53110000	Cake, fruit cake, light or dark, holiday type cake
1775	00-03035	A-00-03035		Fruitful, from Shredded Wheat, Nestle	02.03.00	Breakfast cereals - Other breakfast cereals - low fibre	57100100	Cereal, ready-to-eat, NFS

1776	A-17-0109	17-0109		Fudge	24.02.00	Confectionary - Sugar based products	91713070	Fudge, vanilla
1777	A-12-0161			Full fat soft cheese	06.02.00	Dairy products - Cheese, incl. cottage cheese	14420200	Cheese spread, cream cheese, regular
1778	00-09723	A-00-09723		Full fat soft cheese (Philadelphia)	06.02.00	Dairy products - Cheese, incl. cottage cheese	14420200	Cheese spread, cream cheese, regular
1779	02-07615			GARLIC (& HERB) BREAD	03.04.00	Breads - Other bread	51121015	Garlic bread, NFS
1780	02-06839			GARLIC BREAD. LOWER FAT	03.04.00	Breads - Other bread	51121015	Garlic bread, NFS
1781	02-10459			GLUTEN FREE BROWN BREAD	03.03.00	Breads - Brown/Granary/Wheatgerm	51808000	Bread, gluten free
1782	02-08869			GLUTEN FREE FLOUR MIX	01.04.00	Cereals & cereal dishes - Other cereals & dishes	20061	Rice flour, white, unenriched
1783	02-08872			GLUTEN FREE SWEET BISCUITS	04.01.00	Sweet cereal products - Biscuits	53261000	Cookie, gluten free
1784	01-08394			GLUTEN FREE WHITE BREAD WITH ADDED FIBRE	03.04.00	Breads - Other bread	51808000	Bread, gluten free
1785	02-10327			GOJI BERRIES / WOLFBERRIES DRIED	18.03.00	Fruit - Dried	62109100	Cranberries, dried
1786	02-09020			GOLDEN SYRUP CAKE PURCHASED	04.02.00	Sweet cereal products - Pastries, Buns & Pies	53116000	Cake, pound, without icing or filling
1787	00-05699			GRANARY BREAD TOASTED	03.03.00	Breads - Brown/Granary/Wheatgerm	51301020	Bread, wheat or cracked wheat, toasted
1788	02-09294			GRANULATED TABLE TOP SWEETENER 1/2 TSP	26.03.00	Miscellaneous - Artificial sweeteners	91200000	Sugar substitute, powder, NFS
1789	02-02208			GRANULATED TABLE TOP SWEETENERS	26.03.00	Miscellaneous - Artificial sweeteners	91200000	Sugar substitute, powder, NFS
1790	02-02325			GRAPE JUICE CARBONATED GRAPE JUICE NOT CANNED	27.03.00	Beverages - Carbonated soft drinks	92433000	Fruit juice drink, noncitrus, carbonated
1791	02-02425			GRAVY THICKENED NO FAT	21.02.00	Sauces & accompaniment - Cooking sauces, incl. gravies, pesto, cooking sauces for pasta and rice dishes	28500040	Gravy, beef or meat

1792	02-02427			GRAVY UNTHICKENED NO FAT	21.02.00	Sauces & accompaniment - Cooking sauces, incl. gravies, pesto, cooking sauces for pasta and rice dishes	28500040	Gravy, beef or meat
1793	19-0058			Game pie	10.04.00	Meat - red - Other red meat, e.g. rabbit, venison	27360050	Meat pie, NFS
1794	A-00-03592	00-03592		Gammon, rashers, grilled, unspecified if lean or fat	12.01.00	Processed meat - Bacon & ham	22600100	Bacon, NS as to type of meat, cooked
1795	A-00-01226			Garam Masala	26.01.00	Miscellaneous - Dried herbs & spices & pastes	2010	Spices, cinnamon, ground
1796	A-13-0829			Garam masala	26.01.00	Miscellaneous - Dried herbs & spices & pastes	2010	Spices, cinnamon, ground
1797	A-00-09578	00-09578		Garlic bread	03.04.00	Breads - Other bread	51121015	Garlic bread, NFS
1798	13-0830	A-13-0830		Garlic powder	26.01.00	Miscellaneous - Dried herbs & spices & pastes	2020	Spices, garlic powder
1799	17-0359			Garlic puree	15.04.00	Vegetables - Other	75111500	Garlic, raw
1800	A-19-0103	19-0103		Garlic sausage	13.00.00	Sausages & burgers & kebab	25221350	Italian sausage
1801	A-00-01218			Garlic, Peeled Bulb	15.04.00	Vegetables - Other	75111500	Garlic, raw
1802	A-13-0244	13-0244		Garlic, raw	15.04.00	Vegetables - Other	75111500	Garlic, raw
1803	A-11-0205			Gateau	04.02.00	Sweet cereal products - Pastries, Buns & Pies	53118200	Cake, sponge, with icing or filling
1804	A-00-01033			Gateau (Fatless Sponge with Synthetic Cream)	04.02.00	Sweet cereal products - Pastries, Buns & Pies	53118200	Cake, sponge, with icing or filling
1805	00-09671	A-00-09671		Gateau, chocolate	04.02.00	Sweet cereal products - Pastries, Buns & Pies	53118300	Cake, sponge, chocolate
1806	A-00-09672	00-09672		Gateau, fruit	04.02.00	Sweet cereal products - Pastries, Buns & Pies	53123080	Cake, shortcake, sponge type, with fruit
1807	A-00-01281			Gatia Ghantia - Gram Flour Drops	16.01.00	Pulses/Lentils - Pulses/lentils	41310900	Bean chips
1808	17-0257			Gazpacho	20.01.00	Soups - Canned & fresh & homemade	75604600	Gazpacho
1809	A-00-00959			Gelatin	26.01.00	Miscellaneous - Dried herbs & spices & pastes	19177	Gelatins, dry powder, unsweetened

1810	A-17-0360	17-0360		Gelatine	26.01.00	Miscellaneous - Dried herbs & spices & pastes	19177	Gelatins, dry powder, unsweetened
1811	A-00-01230			Ghee - Butter Ghee	08.03.00	Fats - Animal based fats (solid)	81201000	Animal fat or drippings
1812	13-0246	A-13-0246		Gherkins, pickled, drained	22.02.00	Preserves - Chutney & Pickles (incl. gherkins, pickled onions etc)	75503030	Cucumber pickles, sour
1813	00-05402			Ginger Beer eg Tesco	27.01.03	Beverages - Alcohol - Beer	93101000	Beer
1814	A-00-01219			Ginger Root, Fresh	15.04.00	Vegetables - Other	11216	Ginger root, raw
1815	A-17-0178	17-0178		Ginger ale, dry	27.01.03	Beverages - Alcohol - Beer	93101000	Beer
1816	A-00-00960			Ginger, Ground	26.01.00	Miscellaneous - Dried herbs & spices & pastes	2021	Spices, ginger, ground
1817	A-13-0831	13-0831		Ginger, fresh	19.00.00	Nuts & Seeds (incl. peanut butter)	11216	Ginger root, raw
1818	13-0832	A-13-0832		Ginger, ground	26.01.00	Miscellaneous - Dried herbs & spices & pastes	2021	Spices, ginger, ground
1819	11-0206	A-00-00078	A-11-0206	Gingerbread	04.02.00	Sweet cereal products - Pastries, Buns & Pies	53111000	Cake or cupcake, gingerbread
1820	A-00-06027			Gingerbread 50	04.02.00	Sweet cereal products - Pastries, Buns & Pies	53111000	Cake or cupcake, gingerbread
1821	11-0172	A-11-0172		Gingernut biscuits	04.01.00	Sweet cereal products - Biscuits	53223000	Cookie, gingersnaps
1822	A-00-09948			Gingernut biscuits (MW6 Vit Eq)	04.01.00	Sweet cereal products - Biscuits	53223000	Cookie, gingersnaps
1823	A-00-06017			Gingernut biscuits 50	04.01.00	Sweet cereal products - Biscuits	53223000	Cookie, gingersnaps
1824	A-00-00841			Glucose Liquid B.P.	23.02.00	Sugars - Other, incl. syrups, honey	91301100	Sugar, white, and water syrup
1825	17-0049			Glucose liquid, BP	23.02.00	Sugars - Other, incl. syrups, honey	91301100	Sugar, white, and water syrup
1826	00-05890			Gluten Free Muesli	02.03.00	Breakfast cereals - Other breakfast cereals - low fibre	57308190	Cereal, muesli
1827	00-03159			Gluten free cake	04.02.00	Sweet cereal products - Pastries, Buns & Pies	53116600	Cake, rice flour, without icing or filling
1828	00-05641			Gluten free corn pasta boiled	01.02.00	Cereals & cereal dishes - Pasta & pasta dishes	56140100	Pasta, gluten free

1829	00-05832			Gluten free fish fingers	09.01.00	Fish & fish dishes - White fish, incl. tuna	26100270	Fish stick, patty or nugget from restaurant, home, or other place
1830	00-05642			Gluten free rice and millet pasta, boiled	01.02.00	Cereals & cereal dishes - Pasta & pasta dishes	56140100	Pasta, gluten free
1831	00-05640			Gluten free white bread toasted	03.01.00	Breads - White	51808010	Bread, gluten free, toasted
1832	00-05639			Gluten free white bread, not low protein, not high fibre	03.01.00	Breads - White	51808000	Bread, gluten free
1833	A-00-03750			Gluten-free/low protein bread, Rite-diet 82/1352	03.04.00	Breads - Other bread	51808000	Bread, gluten free
1834	00-03650			Gnocchi, Italian potato dumplings	17.02.00	Potatoes - Potato products - other	58122220	Gnocchi, potato
1835	00-03122	A-00-03122		Go Ahead Golden Crunch biscuit	04.01.00	Sweet cereal products - Biscuits	53233040	Cookie, oatmeal, reduced fat, NS as to raisins
1836	00-03136	A-00-03136		Go Ahead, Berry Bake	04.02.00	Sweet cereal products - Pastries, Buns & Pies	53123080	Cake, shortcake, sponge type, with fruit
1837	A-00-03135			Go Ahead,Crispy fruit slices apple & sultana	04.02.00	Sweet cereal products - Pastries, Buns & Pies	53123080	Cake, shortcake, sponge type, with fruit
1838	00-03135			Go Ahead,Crispy fruit slices, any flavour	04.02.00	Sweet cereal products - Pastries, Buns & Pies	53123080	Cake, shortcake, sponge type, with fruit
1839	A-00-09711	00-09711	A-12-0162	Goats milk soft cheese	06.02.00	Dairy products - Cheese, incl. cottage cheese	14104700	Cheese, goat
1840	A-12-0037	12-0037		Goats milk, pasteurised	05.05.00	Milk - Other - animal based, e.g. goat	11100000	Milk, NFS
1841	A-00-03026			Golden Grahams, Nestle	02.03.00	Breakfast cereals - Other breakfast cereals - low fibre	57224000	Cereal (General Mills Golden Grahams)
1842	00-05848			Good Oil Hemp Seed Oil	08.02.00	Fats - Oils	82101000	Vegetable oil, NFS
1843	00-05565			Goose fat	08.03.00	Fats - Animal based fats (solid)	81201000	Animal fat or drippings
1844	A-00-00331			Goose, Roast	11.02.00	Meat - white - Other game birds, (e.g. duck, goose, pheasant) & dishes	24311010	Goose, wild, roasted

1845	A-00-01125			Gooseberries, Canned, with Sugar (Heavy Syrup)	18.02.00	Fruit - Canned & cooked	63123110	Grapes, seedless, cooked or canned, NS as to sweetened or unsweetened; sweetened, NS as to type of sweetener
1846	A-00-00732			Gooseberries, Green, Raw	18.01.00	Fruit - Fresh	63123000	Grapes, raw, NS as to type
1847	A-00-00734			Gooseberries, Green, Stewed with Sugar	18.02.00	Fruit - Canned & cooked	63123110	Grapes, seedless, cooked or canned, NS as to sweetened or unsweetened; sweetened, NS as to type of sweetener
1848	A-00-00733			Gooseberries, Green, Stewed without Sugar	18.02.00	Fruit - Canned & cooked	63123110	Grapes, seedless, cooked or canned, NS as to sweetened or unsweetened; sweetened, NS as to type of sweetener
1849	A-00-00735			Gooseberries, Ripe, Raw	18.01.00	Fruit - Fresh	63123000	Grapes, raw, NS as to type
1850	A-14-0101	14-0101		Gooseberries, cooking, stewed with sugar	18.02.00	Fruit - Canned & cooked	63123110	Grapes, seedless, cooked or canned, NS as to sweetened or unsweetened; sweetened, NS as to type of sweetener
1851	14-0102			Gooseberries, cooking, stewed without sugar	18.02.00	Fruit - Canned & cooked	63123110	Grapes, seedless, cooked or canned, NS as to sweetened or unsweetened; sweetened, NS as to type of sweetener

1852	14-0104	A-14-0104		Gooseberries, dessert, canned in syrup	18.02.00	Fruit - Canned & cooked	63123110	Grapes, seedless, cooked or canned, NS as to sweetened or unsweetened; sweetened, NS as to type of sweetener
1853	A-14-0103	14-0103		Gooseberries, dessert, raw	18.01.00	Fruit - Fresh	63123000	Grapes, raw, NS as to type
1854	19-0221			Goulash	10.01.00	Meat - red - Beef & veal & dishes	27111100	Beef goulash
1855	A-11-0078	A-00-09559	00-09559	Granary bread	03.03.00	Breads - Brown/Granary/Wheatgerm	51301010	Bread, wheat or cracked wheat
1856	00-09567	A-00-09567		Granary roll	03.03.00	Breads - Brown/Granary/Wheatgerm	51320010	Roll, wheat or cracked wheat
1857	00-05660			Granola, oat based	02.01.00	Breakfast cereals - Oat based cereals	57227000	Cereal, granola
1858	A-14-0273	14-0273		Grape juice, unsweetened	27.02.01	Beverages - Fruit based drinks - Pure fruit juice & smoothies	61210000	Orange juice, 100%, NFS
1859	A-00-00880			Grapefruit Juice, Canned, Sweetened	27.02.00	Beverages - Fruit based drinks	92550350	Orange juice beverage, 40-50% juice, light
1860	A-00-00879			Grapefruit Juice, Canned, Unsweetened	27.02.00	Beverages - Fruit based drinks	61210220	Orange juice, 100%, canned, bottled or in a carton
1861	A-14-0275	14-0275		Grapefruit juice, unsweetened	27.02.01	Beverages - Fruit based drinks - Pure fruit juice & smoothies	61210000	Orange juice, 100%, NFS
1862	A-00-00742			Grapefruit, Canned	18.02.00	Fruit - Canned & cooked	61101200	Grapefruit, canned or frozen, NS as to sweetened or unsweetened; sweetened, NS as to type of sweetener
1863	A-00-01126			Grapefruit, Canned, No Sugar	18.02.00	Fruit - Canned & cooked	61101220	Grapefruit, canned or frozen, unsweetened, water pack

1864	A-00-00740			Grapefruit, Raw	18.01.00	Fruit - Fresh	61101010	Grapefruit, raw
1865	14-0107	A-14-0107		Grapefruit, canned in juice	18.02.00	Fruit - Canned & cooked	61101200	Grapefruit, canned or frozen, NS as to sweetened or unsweetened; sweetened, NS as to type of sweetener
1866	A-14-0108	14-0108		Grapefruit, canned in syrup	18.02.00	Fruit - Canned & cooked	61101230	Grapefruit, canned or frozen, in light syrup
1867	A-14-0105	14-0105		Grapefruit, raw	18.01.00	Fruit - Fresh	61101010	Grapefruit, raw
1868	A-00-00049	A-11-0135		Grapenuts	02.02.00	Breakfast cereals - Other breakfast cereals - high fibre (equal or >3g/40g portion)	57230000	Cereal (Post Grape-Nuts)
1869	00-03027	A-00-03027		Grapenuts, General Foods	02.03.00	Breakfast cereals - Other breakfast cereals - low fibre	57230000	Cereal (Post Grape-Nuts)
1870	A-00-00736			Grapes, Black, Raw	18.01.00	Fruit - Fresh	63123000	Grapes, raw, NS as to type
1871	A-00-00738			Grapes, White, Raw	18.01.00	Fruit - Fresh	63123000	Grapes, raw, NS as to type
1872	A-00-00739			Grapes, White, Raw (whole Grapes Weighed)	18.01.00	Fruit - Fresh	63123000	Grapes, raw, NS as to type
1873	14-0109	A-14-0109		Grapes, average	18.01.00	Fruit - Fresh	63123000	Grapes, raw, NS as to type
1874	A-17-0036	17-0036		Grapeseed oil	08.02.00	Fats - Oils	82101000	Vegetable oil, NFS
1875	00-05464			Gravy Granules Reduced Salt	21.02.00	Sauces & accompaniment - Cooking sauces, incl. gravies, pesto, cooking sauces for pasta and rice dishes	6124	Gravy, pork, dry, powder
1876	00-05466			Gravy Reduced Salt, made up	21.02.00	Sauces & accompaniment - Cooking sauces, incl. gravies, pesto, cooking sauces for pasta and rice dishes	28500040	Gravy, beef or meat

1877	00-03939			Gravy granules, dry, vegetarian	21.02.00	Sauces & accompaniment - Cooking sauces, incl. gravies, pesto, cooking sauces for pasta and rice dishes	6122	Gravy, mushroom, dry, powder
1878	00-03940			Gravy granules, made up,vegetarian	21.02.00	Sauces & accompaniment - Cooking sauces, incl. gravies, pesto, cooking sauces for pasta and rice dishes	28500100	Gravy, mushroom
1879	A-17-0310	17-0310		Gravy instant granules	21.02.00	Sauces & accompaniment - Cooking sauces, incl. gravies, pesto, cooking sauces for pasta and rice dishes	6124	Gravy, pork, dry, powder
1880	A-17-0311	17-0311		Gravy instant granules, made up	21.02.00	Sauces & accompaniment - Cooking sauces, incl. gravies, pesto, cooking sauces for pasta and rice dishes	28500040	Gravy, beef or meat
1881	A-00-01161			Gravy, Average	21.02.00	Sauces & accompaniment - Cooking sauces, incl. gravies, pesto, cooking sauces for pasta and rice dishes	28500040	Gravy, beef or meat
1882	A-00-01159			Gravy, Bisto	21.02.00	Sauces & accompaniment - Cooking sauces, incl. gravies, pesto, cooking sauces for pasta and rice dishes	28500040	Gravy, beef or meat
1883	A-00-01160			Gravy, made with Meat Drippings	21.02.00	Sauces & accompaniment - Cooking sauces, incl. gravies, pesto, cooking sauces for pasta and rice dishes	28500040	Gravy, beef or meat
1884	A-00-03571	00-03571		Gravy, with meat juice and flour	21.03.00	Sauces & accompaniment - Other sauces, incl. brown sauce, soy sauce, ketchup, mint sauce, vinegar	28500040	Gravy, beef or meat

1885	A-00-09643	00-09643		Greek Yogurt with fruit	06.03.01	Dairy products - Yoghurt & drinking yoghurts, incl. buttermilk and probiotics - full fat products	11433990	Yogurt, Greek, NS as to type of milk, fruit
1886	00-09644	A-00-09644		Greek Yogurt with honey	06.03.01	Dairy products - Yoghurt & drinking yoghurts, incl. buttermilk and probiotics - full fat products	11400010	Yogurt, Greek, NS as to type of milk or flavor
1887	11-0248	A-11-0248		Greek pastries	04.02.00	Sweet cereal products - Pastries, Buns & Pies	53441110	Baklava
1888	A-12-0194			Greek yogurt, cows	06.03.01	Dairy products - Yoghurt & drinking yoghurts, incl. buttermilk and probiotics - full fat products	11400010	Yogurt, Greek, NS as to type of milk or flavor
1889	A-00-09642	00-09642		Greek yogurt, cows, whole milk	06.03.01	Dairy products - Yoghurt & drinking yoghurts, incl. buttermilk and probiotics - full fat products	11400010	Yogurt, Greek, NS as to type of milk or flavor
1890	A-12-0195	12-0195		Greek yogurt, sheep	06.03.01	Dairy products - Yoghurt & drinking yoghurts, incl. buttermilk and probiotics - full fat products	11400010	Yogurt, Greek, NS as to type of milk or flavor
1891	A-13-0083	13-0083		Green beans/French beans, boiled in unsalted water	15.04.00	Vegetables - Other	75205010	Beans, string, green, cooked, NS as to form, NS as to fat added in cooking
1892	A-13-0085	13-0085		Green beans/French beans, canned, re-heated, drained	15.04.00	Vegetables - Other	75205003	Beans, string, cooked, from canned, NS as to color, NS as to fat added in cooking
1893	00-05657			Green pesto, purchased or retail only	21.02.00	Sauces & accompaniment - Cooking sauces, incl. gravies, pesto, cooking sauces for pasta and rice dishes	81302070	Pesto sauce

1894	A-00-00743			Greengages, Raw	18.01.00	Fruit - Fresh	63143010	Plum, raw
1895	A-00-00747			Greengages, Stewed with Sugar	18.02.00	Fruit - Canned & cooked	63143110	Plum, cooked or canned, NS as to sweetened or unsweetened; sweetened, NS as to type of sweetener
1896	A-00-00748			Greengages, Stewed with Sugar (Weighed with Stones)	18.02.00	Fruit - Canned & cooked	63143110	Plum, cooked or canned, NS as to sweetened or unsweetened; sweetened, NS as to type of sweetener
1897	19-0046	A-19-0046		Grillsteaks, beef, chilled/frozen, grilled	10.01.00	Meat - red - Beef & veal & dishes	23573	Beef, ground, 80% lean meat / 20% fat, patty, cooked, broiled
1898	A-00-00332			Grouse, Roast	11.02.00	Meat - white - Other game birds, (e.g. duck, goose, pheasant) & dishes	24402100	Dove, cooked, NS as to cooking method
1899	A-00-00333			Grouse, Roast (Weighed with Bone)	11.02.00	Meat - white - Other game birds, (e.g. duck, goose, pheasant) & dishes	24402100	Dove, cooked, NS as to cooking method
1900	A-17-0312	17-0312		Guacamole	15.04.00	Vegetables - Other	63409010	Guacamole, NFS
1901	A-00-01243			Guava, Fresh	18.01.00	Fruit - Fresh	63125010	Guava, raw
1902	14-0118			Guava, raw	18.01.00	Fruit - Fresh	63125010	Guava, raw
1903	A-00-00749			Guavas, Canned	18.02.00	Fruit - Canned & cooked	63125100	Guava shell, canned in heavy syrup
1904	02-10223			HAKE GRILLED OR OVEN BAKED	09.01.00	Fish & fish dishes - White fish, incl. tuna	26109123	Cod, baked or broiled, made without fat
1905	02-09616			HALO REDUCED CALORIE AND FAT CHOCOLATE BAR	24.01.00	Confectionary - Chocolate based products	91705010	Milk chocolate candy, plain
1906	02-08697			HAM LOW FAT EG. DELIGHT	12.01.00	Processed meat - Bacon & ham	22301120	Ham, fresh, cooked, lean only eaten
1907	02-00860			HARD BLOCK MARGARINES AND FATS (75-90% FAT)	08.04.01	Fats - Plant based fats (solid) - Full fat	81102000	Margarine, NFS

1908	02-01674			HARICOT BEANS/CANNED BOILED DRAINED WEIGHT	16.01.00	Pulses/Lentils - Pulses/lentils	41101000	Beans, dry, cooked, NS as to type and as to fat added in cooking
1909	02-07656			HARVEST CHEWEEE CEREAL BARS ONLY, FORTIFIED	04.05.00	Sweet cereal products - Cereal bars	53712100	Cereal or Granola bar, NFS
1910	02-05627			HEINZ WEIGHT WATCHERS CHICKEN CURRY WITH RICE READY MEAL	11.01.00	Meat - white - Chicken & turkey & dishes	27146150	Chicken curry
1911	02-01498			HERRING CANNED IN OIL FISH ONLY	09.02.00	Fish & fish dishes - Oily fish	26119110	Herring, cooked, NS as to cooking method
1912	02-10036			HIGH JUICE DRINK CONCENTRATE, NO ADDED SUGAR, BLACKCURRANT	27.02.03	Beverages - Fruit based drinks - Squashes & fruit concentrates	91301050	Fruit syrup
1913	02-10037			HIGH JUICE DRINK CONCENTRATE, NO ADDED SUGAR, NOT BLACKCURRANT	27.02.03	Beverages - Fruit based drinks - Squashes & fruit concentrates	91301050	Fruit syrup
1914	02-07911			HIGH JUICE DRINK; SQUASH CONC NOT B/C NOT LOW CAL	27.02.03	Beverages - Fruit based drinks - Squashes & fruit concentrates	91301050	Fruit syrup
1915	02-08659			HOMEMADE MUSHROOM SOUP	20.01.00	Soups - Canned & fresh & homemade	75607000	Mushroom soup, NFS
1916	02-10468			HONEY MONSTER HONEY WAFFLE BREAKFAST CEREAL FORTIFIED	02.02.00	Breakfast cereals - Other breakfast cereals - high fibre (equal or >3g/40g portion)	57100100	Cereal, ready-to-eat, NFS
1917	02-10265			HONEY MUSTARD DRESSINGS AND MARINADES PURCHASED	21.01.00	Sauces & accompaniment - Dressings & Mayonnaise	83105500	Honey mustard dressing
1918	02-06011			HONEY ROASTED PEANUTS	19.00.00	Nuts & Seeds (incl. peanut butter)	42111500	Peanuts, honey roasted
1919	02-09278			HORLICKS CHOCOLATE NOT INSTANT NOT LOW FAT DRY WEI	27.05.00	Beverages - Powdered Beverages (cocoa, Horlicks, Bonvita, Ovaltine, etc)	11830260	Milk, malted, dry mix, not reconstituted

1920	02-09277			HORLICKS LIGHT MALT CHOCOLATE INSTANT DRY WEIGHT	27.05.00	Beverages - Powdered Beverages (cocoa, Horlicks, Bonvita, Ovaltine, etc)	11830260	Milk, malted, dry mix, not reconstituted
1921	02-07675			HOT CROSS BUNS, CURRANT BUNS, WHOLEMEAL	04.02.00	Sweet cereal products - Pastries, Buns & Pies	51160100	Roll, sweet, cinnamon bun, no frosting
1922	A-00-03142			HRT cake	04.02.00	Sweet cereal products - Pastries, Buns & Pies	53110000	Cake, fruit cake, light or dark, holiday type cake
1923	02-01717			HUMMUS CANNED	16.01.00	Pulses/Lentils - Pulses/lentils	41205070	Hummus, plain
1924	02-01817			HUMMUS, NOT CANNED	16.01.00	Pulses/Lentils - Pulses/lentils	41205070	Hummus, plain
1925	00-03653			Haddock in crumbs Ovenbaked	09.01.00	Fish & fish dishes - White fish, incl. tuna	26117131	Haddock, coated, baked or broiled, fat not added in cooking
1926	A-00-03794			Haddock steak in crumbs, grilled/baked (B.E.) 82/1436	09.01.00	Fish & fish dishes - White fish, incl. tuna	26117131	Haddock, coated, baked or broiled, fat not added in cooking
1927	A-00-00452			Haddock, Fresh, Fried	09.01.00	Fish & fish dishes - White fish, incl. tuna	26117120	Haddock, baked or broiled, fat added in cooking
1928	A-00-06251			Haddock, Fresh, In crumbs, Fried (dripping) 50	09.01.00	Fish & fish dishes - White fish, incl. tuna	26117140	Haddock, coated, fried
1929	A-00-00451			Haddock, Fresh, Raw	09.01.00	Fish & fish dishes - White fish, incl. tuna	15033	Fish, haddock, raw
1930	A-00-00454			Haddock, Fresh, Steamed	09.01.00	Fish & fish dishes - White fish, incl. tuna	26117160	Haddock, steamed or poached
1931	A-00-00455			Haddock, Fresh, Steamed (Weighed with Bones and Skin)	09.01.00	Fish & fish dishes - White fish, incl. tuna	26117160	Haddock, steamed or poached
1932	A-00-00456			Haddock, Smoked, Steamed	09.01.00	Fish & fish dishes - White fish, incl. tuna	26117160	Haddock, steamed or poached
1933	A-00-00457			Haddock, Smoked, Steamed (Weighed with Bones and Skin)	09.01.00	Fish & fish dishes - White fish, incl. tuna	26117160	Haddock, steamed or poached

1934	16-0063	A-16-0063		Haddock, coated in crumbs, frozen, fried in blended oil	09.01.00	Fish & fish dishes - White fish, incl. tuna	26117140	Haddock, coated, fried
1935	A-16-0045	16-0045		Haddock, grilled	09.01.00	Fish & fish dishes - White fish, incl. tuna	26117121	Haddock, baked or broiled, fat not added in cooking
1936	A-16-0054	16-0054		Haddock, in batter, fried in retail blend oil	09.01.00	Fish & fish dishes - White fish, incl. tuna	26117140	Haddock, coated, fried
1937	16-0047	A-16-0047		Haddock, poached	09.01.00	Fish & fish dishes - White fish, incl. tuna	26117160	Haddock, steamed or poached
1938	A-16-0068	16-0068		Haddock, smoked, steamed	09.01.00	Fish & fish dishes - White fish, incl. tuna	26117160	Haddock, steamed or poached
1939	A-16-0049	16-0049		Haddock, steamed	09.01.00	Fish & fish dishes - White fish, incl. tuna	26117160	Haddock, steamed or poached
1940	A-00-00403			Haggis, Boiled	14.02.00	Offal - Other offal & dishes, e.g. Haggis, faggots	25112200	Liver paste or pate, chicken
1941	A-19-0132	19-0132		Haggis, boiled	14.02.00	Offal - Other offal & dishes, e.g. Haggis, faggots	25112200	Liver paste or pate, chicken
1942	00-05894			Half fat butter, spreadable eg M&S	08.01.00	Fats - Butter	81100500	Butter, NFS
1943	A-00-00459			Halibut, Steamed	09.01.00	Fish & fish dishes - White fish, incl. tuna	26118050	Halibut, steamed or poached
1944	16-0074	A-16-0074		Halibut, grilled	09.01.00	Fish & fish dishes - White fish, incl. tuna	26118023	Halibut, baked or broiled, made without fat
1945	A-16-0076	16-0076		Halibut, poached	09.01.00	Fish & fish dishes - White fish, incl. tuna	26118050	Halibut, steamed or poached
1946	A-00-01312	11-0251	A-11-0251	Halva	04.02.00	Sweet cereal products - Pastries, Buns & Pies	91716010	Halvah, plain
1947	19-0023	A-19-0023		Ham	12.01.00	Processed meat - Bacon & ham	22301000	Ham, fresh, cooked, NS as to fat eaten
1948	A-00-06209			Ham 50 boiled lean and fat	12.01.00	Processed meat - Bacon & ham	22301110	Ham, fresh, cooked, lean and fat eaten

1949	A-00-00395			Ham and Pork, Chopped, Canned	12.01.00	Processed meat - Bacon & ham	22311500	Ham, smoked or cured, canned, NS as to fat eaten
1950	A-19-0133	19-0133		Ham and pork, chopped, canned	12.01.00	Processed meat - Bacon & ham	22311500	Ham, smoked or cured, canned, NS as to fat eaten
1951	A-00-00394			Ham, Canned	12.01.00	Processed meat - Bacon & ham	22311500	Ham, smoked or cured, canned, NS as to fat eaten
1952	19-0025	A-19-0025		Ham, Parma	12.01.00	Processed meat - Bacon & ham	22301000	Ham, fresh, cooked, NS as to fat eaten
1953	19-0024	A-19-0024		Ham, canned	12.01.00	Processed meat - Bacon & ham	22311500	Ham, smoked or cured, canned, NS as to fat eaten
1954	19-0021	A-19-0021		Ham, gammon joint, boiled	12.01.00	Processed meat - Bacon & ham	22301000	Ham, fresh, cooked, NS as to fat eaten
1955	19-0022	A-19-0022		Ham, gammon rashers, grilled	12.01.00	Processed meat - Bacon & ham	22301000	Ham, fresh, cooked, NS as to fat eaten
1956	A-00-01098			Ham/ Bacon and Egg Pie	12.02.00	Processed meat - Processed pies	58125110	Quiche with meat, poultry or fish
1957	11-0121	A-11-0121		Hamburger buns	03.04.00	Breads - Other bread	51154100	Roll, white, hamburger bun
1958	19-0047	A-19-0047		Hamburger, takeaway	13.00.00	Sausages & burgers & kebab	27510521	Hamburger, NFS
1959	A-12-0165			Hard cheese, average	06.02.00	Dairy products - Cheese, incl. cottage cheese	14102010	Cheese, Brick
1960	A-00-00348			Hare, Stewed	10.04.00	Meat - red - Other red meat, e.g. rabbit, venison	23310000	Rabbit, NS as to domestic or wild, cooked
1961	13-0087	A-13-0087		Haricot beans, dried, boiled in unsalted water	16.01.00	Pulses/Lentils - Pulses/lentils	41101000	Beans, dry, cooked, NS as to type and as to fat added in cooking
1962	A-00-03804			Harvest Crunch 82/1447	02.03.00	Breakfast cereals - Other breakfast cereals - low fibre	57100100	Cereal, ready-to-eat, NFS
1963	00-03649			Hash Browns, rosti oven baked	17.02.00	Potatoes - Potato products - other	71404000	Potato, hash brown, NFS

1964	14-0821	A-14-0821		Hazelnuts	19.00.00	Nuts & Seeds (incl. peanut butter)	42107000	Hazelnuts
1965	A-00-00358			Heart, Lamb, Raw	14.02.00	Offal - Other offal & dishes, e.g. Haggis, faggots	5027	Chicken, liver, all classes, raw
1966	A-00-00361			Heart, Ox, Stewed	14.02.00	Offal - Other offal & dishes, e.g. Haggis, faggots	25120000	Heart, cooked
1967	A-00-00359			Heart, Sheep, Roast	14.02.00	Offal - Other offal & dishes, e.g. Haggis, faggots	25120000	Heart, cooked
1968	A-18-0397	18-0397		Heart, lamb, roasted	14.02.00	Offal - Other offal & dishes, e.g. Haggis, faggots	25120000	Heart, cooked
1969	18-0399			Heart, ox, stewed	14.02.00	Offal - Other offal & dishes, e.g. Haggis, faggots	25120000	Heart, cooked
1970	00-05476			Heinz Alphabetti Pasta Shapes	28.02.02	Baby & infant foods/drinks - Ready meals - Cereal based	58146223	Pasta with tomato-based sauce, ready-to-heat
1971	00-03153			Heinz Weight Watchers Sultana & Cinnamon Cookies	04.01.00	Sweet cereal products - Biscuits	53237000	Cookie, raisin
1972	A-00-00483			Herring, Fried	09.02.00	Fish & fish dishes - Oily fish	26119120	Herring, baked or broiled, fat added in cooking
1973	A-00-00485			Herring, Grilled	09.02.00	Fish & fish dishes - Oily fish	26119121	Herring, baked or broiled, fat not added in cooking
1974	A-00-00486			Herring, Grilled (Weighed with Bones)	09.02.00	Fish & fish dishes - Oily fish	26119121	Herring, baked or broiled, fat not added in cooking
1975	A-00-00482			Herring, Raw	09.02.00	Fish & fish dishes - Oily fish	26119100	Herring, raw
1976	16-0182	A-16-0182		Herring, canned in tomato sauce	09.02.00	Fish & fish dishes - Oily fish	27150350	Sardines with tomato-based sauce
1977	A-16-0176	16-0176		Herring, grilled	09.02.00	Fish & fish dishes - Oily fish	26119121	Herring, baked or broiled, fat not added in cooking
1978	16-0178	A-16-0178		Herring, in oatmeal, fried in vegetable oil	09.02.00	Fish & fish dishes - Oily fish	26119140	Herring, coated, fried

1979	A-16-0183	16-0183		Herring, pickled	09.02.00	Fish & fish dishes - Oily fish	26119180	Herring, pickled
1980	A-00-03855			Hi-juice blackcurrant, undiluted	27.02.03	Beverages - Fruit based drinks - Squashes & fruit concentrates	91301050	Fruit syrup
1981	00-03878			Hi-juice, blackcurrant made up	27.02.03	Beverages - Fruit based drinks - Squashes & fruit concentrates	92510610	Fruit juice drink
1982	00-03322	A-00-03322		High Fibre White Bread Toasted	03.01.00	Breads - White	51122010	Bread, reduced calorie and/or high fiber, white or NFS, toasted
1983	01-00213			High Protein Energy Supplement eg Nature Plus Spiru-tein	30.00.00	Nutrition Powders & drinks	95230020	Nutritional powder mix, protein, light, NFS
1984	00-05895			High fibre bran eg Morrisons	02.02.00	Breakfast cereals - Other breakfast cereals - high fibre (equal or >3g/40g portion)	57100100	Cereal, ready-to-eat, NFS
1985	00-03326	A-00-03326		High fibre white bread (mighty white)	03.01.00	Breads - White	51122000	Bread, reduced calorie and/or high fiber, white or NFS
1986	17-0197	A-17-0197		High juice drink, concentrated	27.02.03	Beverages - Fruit based drinks - Squashes & fruit concentrates	91301050	Fruit syrup
1987	A-17-0198	17-0198		High juice drink, concentrated, made up	27.02.03	Beverages - Fruit based drinks - Squashes & fruit concentrates	92510610	Fruit juice drink
1988	00-05382			Hipp Apple and Grape Juice	28.06.00	Baby & infant foods/drinks - Drinks	67203500	Apple-grape juice, baby food
1989	A-00-03144			Hob Nob Biscuits (cream filled)	04.01.00	Sweet cereal products - Biscuits	53233050	Cookie, oatmeal sandwich, with creme filling
1990	A-00-03143	00-03143		Hob Nobs, chocolate coated	04.01.00	Sweet cereal products - Biscuits	54102020	Graham crackers, chocolate covered
1991	A-00-03141	00-03141		Hob Nobs, plain	04.01.00	Sweet cereal products - Biscuits	53233040	Cookie, oatmeal, reduced fat, NS as to raisins

1992	17-0313	A-17-0313		Hollandaise sauce, homemade	21.02.00	Sauces & accompaniment - Cooking sauces, incl. gravies, pesto, cooking sauces for pasta and rice dishes	81302010	Hollandaise sauce
1993	11-0174	A-11-0174		Homemade biscuits, creaming method	04.01.00	Sweet cereal products - Biscuits	53241500	Cookie, butter or sugar
1994	A-17-0050	17-0050		Honey	23.02.00	Sugars - Other, incl. syrups, honey	91302010	Honey
1995	00-05903			Honey Mustard dressing, low fat, purchased	21.01.00	Sauces & accompaniment - Dressings & Mayonnaise	83204500	Honey mustard dressing, light
1996	A-00-03002	00-03002		Honey Nut Shredded Wheat	02.02.00	Breakfast cereals - Other breakfast cereals - high fibre (equal or >3g/40g portion)	57241200	Cereal (Post Shredded Wheat Honey Nut)
1997	A-11-0136			Honey Smacks	02.03.00	Breakfast cereals - Other breakfast cereals - low fibre	57243000	Cereal (Kellogg's Honey Smacks)
1998	A-00-03028			Honey Smacks, Kelloggs	02.03.00	Breakfast cereals - Other breakfast cereals - low fibre	57243000	Cereal (Kellogg's Honey Smacks)
1999	A-00-00847			Honey, Comb	23.02.00	Sugars - Other, incl. syrups, honey	91302010	Honey
2000	A-00-00848			Honey, in Jars	23.02.00	Sugars - Other, incl. syrups, honey	91302010	Honey
2001	A-17-0051	17-0051		Honeycomb	23.02.00	Sugars - Other, incl. syrups, honey	91302010	Honey
2002	00-03882			Horlicks original powder, fortified	27.05.00	Beverages - Powdered Beverages (cocoa, Horlicks, Bonvita, Ovaltine, etc)	11830260	Milk, malted, dry mix, not reconstituted
2003	A-00-03882	A-12-0097		Horlicks powder	27.05.00	Beverages - Powdered Beverages (cocoa, Horlicks, Bonvita, Ovaltine, etc)	11830260	Milk, malted, dry mix, not reconstituted
2004	A-12-0099			Horlicks powder, made up with semi-skimmed milk	27.05.00	Beverages - Powdered Beverages (cocoa, Horlicks, Bonvita, Ovaltine, etc)	11526000	Milk, malted

2005	A-12-0100			Horlicks powder, made up with skimmed milk	27.05.00	Beverages - Powdered Beverages (cocoa, Horlicks, Bonvita, Ovaltine, etc)	11526000	Milk, malted
2006	A-12-0098			Horlicks powder, made up with whole milk	27.05.00	Beverages - Powdered Beverages (cocoa, Horlicks, Bonvita, Ovaltine, etc)	11526000	Milk, malted
2007	A-00-03844			Horlicks, LF instant powder	27.05.00	Beverages - Powdered Beverages (cocoa, Horlicks, Bonvita, Ovaltine, etc)	11830260	Milk, malted, dry mix, not reconstituted
2008	00-03843	A-00-03843		Horlicks, LF, made up with water	27.05.00	Beverages - Powdered Beverages (cocoa, Horlicks, Bonvita, Ovaltine, etc)	11514100	Hot chocolate / Cocoa, made with dry mix and water
2009	00-03844			Horlicks, Low Fat, instant powder, fortifed	27.05.00	Beverages - Powdered Beverages (cocoa, Horlicks, Bonvita, Ovaltine, etc)	11830260	Milk, malted, dry mix, not reconstituted
2010	A-00-00874			Horlicks, Malted Milk	27.05.00	Beverages - Powdered Beverages (cocoa, Horlicks, Bonvita, Ovaltine, etc)	11830260	Milk, malted, dry mix, not reconstituted
2011	00-09803			Horlicks, made with whole milk	27.05.00	Beverages - Powdered Beverages (cocoa, Horlicks, Bonvita, Ovaltine, etc)	11526000	Milk, malted
2012	00-09807	A-00-09807		Horlicks, with semi-skimmed milk	27.05.00	Beverages - Powdered Beverages (cocoa, Horlicks, Bonvita, Ovaltine, etc)	11526000	Milk, malted
2013	A-00-09808	00-09808		Horlicks, with skimmed milk	27.05.00	Beverages - Powdered Beverages (cocoa, Horlicks, Bonvita, Ovaltine, etc)	11526000	Milk, malted
2014	A-00-09803			Horlicks, with whole milk	27.05.00	Beverages - Powdered Beverages (cocoa, Horlicks, Bonvita, Ovaltine, etc)	11526000	Milk, malted
2015	17-0314	A-17-0314		Horseradish sauce	21.03.00	Sauces & accompaniment - Other sauces, incl. brown sauce, soy sauce, ketchup, mint sauce, vinegar	81302060	Horseradish sauce

2016	A-00-00599			Horseradish, Raw	15.04.00	Vegetables - Other	75503090	Horseradish
2017	13-0258			Horseradish, raw	15.04.00	Vegetables - Other	75503090	Horseradish
2018	A-00-00430			Hot Pot	10.01.00	Meat - red - Beef & veal & dishes	75652040	Vegetable beef soup with noodles or pasta, home recipe
2019	00-03885			Hot chocolate, instant, powder	27.05.00	Beverages - Powdered Beverages (cocoa, Horlicks, Bonvita, Ovaltine, etc)	11830100	Hot chocolate / Cocoa, dry mix, not reconstituted
2020	11-0253	A-11-0253		Hot cross buns	04.02.00	Sweet cereal products - Pastries, Buns & Pies	51160100	Roll, sweet, cinnamon bun, no frosting
2021	A-00-06074			Hot cross buns 50	04.02.00	Sweet cereal products - Pastries, Buns & Pies	51160100	Roll, sweet, cinnamon bun, no frosting
2022	17-0315			Hot pepper sauce (Tabasco, Encona, Calypso)	21.03.00	Sauces & accompaniment - Other sauces, incl. brown sauce, soy sauce, ketchup, mint sauce, vinegar	75511010	Hot pepper sauce
2023	A-11-0079			Hovis, average	03.01.00	Breads - White	51101000	Bread, white
2024	A-11-0083			Hovis, toasted	03.01.00	Breads - White	51101010	Bread, white, toasted
2025	A-13-0088	13-0088		Hummus	16.01.00	Pulses/Lentils - Pulses/lentils	41205070	Hummus, plain
2026	A-00-05131			Hummus (Chickpea Spread)	16.01.00	Pulses/Lentils - Pulses/lentils	41205070	Hummus, plain
2027	02-09814			ICE CREAM - HOMEMADE WITH DOUBLE CREAM	06.04.01	Dairy products - Ice cream & dairy desserts - full fat products	13110120	Ice cream, rich, flavors other than chocolate
2028	02-09927			ICE CREAM ALTERNATIVE, VIRTUALLY FAT FREE E.G. SKINNY COW STICK PRODUCTS	06.04.02	Dairy products - Ice cream & dairy desserts - reduced fat products	13160420	Fat free ice cream, NS as to flavor
2029	02-00729			ICE CREAM LOLLIES EG MIVVI	24.03.00	Confectionary - Sorbets & lollies	13120400	Ice cream bar or stick with fruit
2030	02-00728			ICE CREAM MR WHIPPY TYPE SOFT	06.04.02	Dairy products - Ice cream & dairy desserts - reduced fat products	13110200	Ice cream, soft serve, flavors other than chocolate

2031	02-07758			ICE CREAM REDUCED CALORIE	06.04.02	Dairy products - Ice cream & dairy desserts - reduced fat products	13130100	Light ice cream, NS as to flavor
2032	02-00720			ICE CREAM, DAIRY, VANILLA, HARD, BLOCK	06.04.02	Dairy products - Ice cream & dairy desserts - reduced fat products	13110000	Ice cream, NFS
2033	02-00723			ICE CREAM, STANDARD, NON DAIRY (MADE WITH WHEY PROTEINS), VANILLA SOFT SCOOP	06.04.02	Dairy products - Ice cream & dairy desserts - reduced fat products	13110000	Ice cream, NFS
2034	02-02262			ICE LOLLIES, JUICE BASED, NOT FORTIFIED WITH VITAMIN C	24.03.00	Confectionary - Sorbets & lollies	63420110	Fruit juice bar, frozen, flavor other than orange
2035	02-08201			ICED BISCUITS	04.01.00	Sweet cereal products - Biscuits	53240010	Cookie, animal, with frosting or icing
2036	02-04429			ICED CHERRY BAKEWELL TART, LARGE OR SMALL , PURCHASED	04.02.00	Sweet cereal products - Pastries, Buns & Pies	53452100	Pastry, fruit-filled
2037	02-08046			INSTANT LATTE POWDER NOT MADE UP	27.06.00	Beverages - Coffee	14192	Beverages, Cocoa mix, powder
2038	02-09368			INSTANT MALTED DRINKS DRY WEIGHT NOT FORTIFIED	27.05.00	Beverages - Powdered Beverages (cocoa, Horlicks, Bonvita, Ovaltine, etc)	11830260	Milk, malted, dry mix, not reconstituted
2039	02-08045			INSTANT MOCHA POWDER (NOT MADE UP)	27.06.00	Beverages - Coffee	14192	Beverages, Cocoa mix, powder
2040	02-05329			INSTANT OAT CEREAL WITH FRUIT AND/ OR NUTS EG. OATSO SIMPLE BAKED APPLE, DRY WEIGHT	02.01.00	Breakfast cereals - Oat based cereals	57000100	Cereal, oat, NFS
2041	02-03805			ITALIAN PANNA COTTA DESSERT SAINSBURYS AND WAITROSE	06.04.02	Dairy products - Ice cream & dairy desserts - reduced fat products	13110000	Ice cream, NFS

2042	02-05723			ITALIAN TUNA TWISTS PASTA WITH TUNA IN TOMATO SAUCE	01.02.00	Cereals & cereal dishes - Pasta & pasta dishes	58146363	Pasta with tomato-based sauce and seafood, ready-to-heat
2043	A-00-06051			Ice Cream 50	06.04.01	Dairy products - Ice cream & dairy desserts - full fat products	13110000	Ice cream, NFS
2044	A-00-00107			Ice Cream, Dairy	06.04.02	Dairy products - Ice cream & dairy desserts - reduced fat products	13110000	Ice cream, NFS
2045	A-00-00108			Ice Cream, Non-dairy	06.04.02	Dairy products - Ice cream & dairy desserts - reduced fat products	41420380	Yogurt, soy
2046	A-00-01253			Ice Lolly, Orange Fruitie	24.03.00	Confectionary - Sorbets & lollies	63420100	Fruit juice bar, frozen, orange flavor
2047	A-00-01252			Ice Lolly, Strawberry Split	24.03.00	Confectionary - Sorbets & lollies	63420110	Fruit juice bar, frozen, flavor other than orange
2048	A-00-09631	00-09631		Ice cream dairy, chocolate	06.04.02	Dairy products - Ice cream & dairy desserts - reduced fat products	13110110	Ice cream, regular, chocolate
2049	00-09630	A-00-09630		Ice cream dairy, vanilla	06.04.02	Dairy products - Ice cream & dairy desserts - reduced fat products	13110100	Ice cream, regular, flavors other than chocolate
2050	00-03071			Ice cream luxury, dairy, vanilla	06.04.01	Dairy products - Ice cream & dairy desserts - full fat products	13110120	Ice cream, rich, flavors other than chocolate
2051	A-00-09628	00-09628		Ice cream non-dairy, chocolate	06.04.02	Dairy products - Ice cream & dairy desserts - reduced fat products	41420380	Yogurt, soy
2052	00-09627	A-00-09627		Ice cream non-dairy, vanilla	06.04.02	Dairy products - Ice cream & dairy desserts - reduced fat products	41420380	Yogurt, soy
2053	A-00-09629	00-09629		Ice cream non-diary, fruit flavoured	06.04.02	Dairy products - Ice cream & dairy desserts - reduced fat products	41420380	Yogurt, soy

2054	17-0053	A-17-0053		Ice cream sauce, topping	21.03.00	Sauces & accompaniment - Other sauces, incl. brown sauce, soy sauce, ketchup, mint sauce, vinegar	91304020	Topping, chocolate, thick, fudge type
2055	00-09634	A-00-09634		Ice cream soya, dairy free	06.04.02	Dairy products - Ice cream & dairy desserts - reduced fat products	41420380	Yogurt, soy
2056	A-12-0212	12-0212		Ice cream wafers	04.01.00	Sweet cereal products - Biscuits	13120500	Ice cream sandwich
2057	A-12-0205			Ice cream, dairy, flavoured	06.04.02	Dairy products - Ice cream & dairy desserts - reduced fat products	13110000	Ice cream, NFS
2058	A-12-0204			Ice cream, dairy, vanilla	06.04.02	Dairy products - Ice cream & dairy desserts - reduced fat products	13110000	Ice cream, NFS
2059	00-03072			Ice cream, luxury, dairy, flavoured	06.04.01	Dairy products - Ice cream & dairy desserts - full fat products	13110120	Ice cream, rich, flavors other than chocolate
2060	A-12-0207			Ice cream, non-dairy, flavoured	06.04.02	Dairy products - Ice cream & dairy desserts - reduced fat products	41420380	Yogurt, soy
2061	A-12-0208	12-0208		Ice cream, non-dairy, mixes	06.04.02	Dairy products - Ice cream & dairy desserts - reduced fat products	41420380	Yogurt, soy
2062	12-0209	A-12-0209		Ice cream, non-dairy, reduced calorie	06.04.02	Dairy products - Ice cream & dairy desserts - reduced fat products	41420380	Yogurt, soy
2063	A-12-0206			Ice cream, non-dairy, vanilla	06.04.02	Dairy products - Ice cream & dairy desserts - reduced fat products	41420380	Yogurt, soy
2064	12-0210	A-12-0210		Ice cream, with cone	06.04.02	Dairy products - Ice cream & dairy desserts - reduced fat products	13120740	Ice cream cone, no topping, NS as to flavor
2065	A-12-0211			Ice cream, with wafers	06.04.02	Dairy products - Ice cream & dairy desserts - reduced fat products	13120740	Ice cream cone, no topping, NS as to flavor

2066	A-00-09639	00-09639		Ice lolly, Orange Fruity	24.03.00	Confectionary - Sorbets & lollies	63420100	Fruit juice bar, frozen, orange flavor
2067	A-00-09674	00-09674		Ice lolly, Strawberry Split	24.03.00	Confectionary - Sorbets & lollies	63420110	Fruit juice bar, frozen, flavor other than orange
2068	A-00-03127	00-03127		Iced Biscuits	04.01.00	Sweet cereal products - Biscuits	53240010	Cookie, animal, with frosting or icing
2069	00-03131	A-00-03131		Iced bun	04.02.00	Sweet cereal products - Pastries, Buns & Pies	51160110	Roll, sweet, cinnamon bun, frosted
2070	00-05431			Iceland Sweet Chilli Chicken Pizza(ovenbaked)	01.01.00	Cereals & cereal dishes - Pizza	58106700	Pizza with meat and vegetables, from frozen, thin crust
2071	A-17-0057	17-0057		Icing, Royal	23.02.00	Sugars - Other, incl. syrups, honey	91305020	Icing, white
2072	A-17-0054	17-0054		Icing, butter	23.02.00	Sugars - Other, incl. syrups, honey	91305020	Icing, white
2073	A-17-0055	17-0055		Icing, fondant	23.02.00	Sugars - Other, incl. syrups, honey	91707000	Fondant
2074	A-17-0056	17-0056		Icing, glace	23.01.00	Sugars - Pure sugars	91305020	Icing, white
2075	A-00-01176			Indian Meat Curry, All Types	10.01.00	Meat - red - Beef & veal & dishes	27116100	Beef curry
2076	A-00-03773			Instant Whip, made up 82/1412	06.01.00	Dairy products - Cream & fromage frais	12100100	Cream, NS as to light, heavy, or half and half
2077	00-05612			Instant cappuccino powder	27.06.00	Beverages - Coffee	14192	Beverages, Cocoa mix, powder
2078	00-05897			Instant custard powder eg Birds	01.04.00	Cereals & cereal dishes - Other cereals & dishes	20017	Corn flour, masa, enriched, white
2079	A-00-09819	00-09819		Instant dessert & semi-skimmed milk	06.04.02	Dairy products - Ice cream & dairy desserts - reduced fat products	13220110	Pudding, flavors other than chocolate, prepared from dry mix, milk added
2080	A-00-09820	00-09820		Instant dessert & skimmed milk	06.04.02	Dairy products - Ice cream & dairy desserts - reduced fat products	13220110	Pudding, flavors other than chocolate, prepared from dry mix, milk added

2081	A-12-0235			Instant dessert powder, made up with semi-skimmed milk	06.04.02	Dairy products - Ice cream & dairy desserts - reduced fat products	13220110	Pudding, flavors other than chocolate, prepared from dry mix, milk added
2082	A-12-0236			Instant dessert powder, made up with skimmed milk	06.04.02	Dairy products - Ice cream & dairy desserts - reduced fat products	13220110	Pudding, flavors other than chocolate, prepared from dry mix, milk added
2083	A-12-0234			Instant dessert powder, made up with whole milk	06.04.01	Dairy products - Ice cream & dairy desserts - full fat products	13220110	Pudding, flavors other than chocolate, prepared from dry mix, milk added
2084	00-03259	A-00-03259		Instant dessert powder, not made up	04.04.00	Sweet cereal products - Milk based puddings	19202	Puddings, vanilla, dry mix, instant
2085	A-00-09818			Instant dessert powder,& whole milk	06.04.01	Dairy products - Ice cream & dairy desserts - full fat products	13220110	Pudding, flavors other than chocolate, prepared from dry mix, milk added
2086	00-05776			Instant hot oat cereal, plain, dry weight, fortified e.g. Ready brek and own brand	02.01.00	Breakfast cereals - Oat based cereals	56202960	Oatmeal, NS as to regular, quick, or instant, NS as to fat added in cooking
2087	00-05611			Instant low fat/skinny cappuccino powder	27.06.00	Beverages - Coffee	14192	Beverages, Cocoa mix, powder
2088	A-13-0032	13-0032		Instant potato powder, made up with water	17.02.00	Potatoes - Potato products - other	71501035	Potato, mashed, from dry mix, NFS
2089	17-0259			Instant soup powder	20.02.00	Soups - Dried	6128	Soup, chicken noodle, dry, mix
2090	A-17-0260	17-0260		Instant soup powder, as served	20.02.00	Soups - Dried	58400000	Soup, NFS
2091	17-0262	A-17-0262		Instant soup powder, calorie controlled, as served	20.02.00	Soups - Dried	58400000	Soup, NFS
2092	A-00-00431			Irish Stew	10.01.00	Meat - red - Beef & veal & dishes	27211200	Beef stew with potatoes, gravy
2093	19-0222	A-19-0222		Irish stew	10.01.00	Meat - red - Beef & veal & dishes	27211200	Beef stew with potatoes, gravy

2094	A-00-06215			Irish stew 50	10.02.00	Meat - red - Lamb & dishes	27330030	Lamb or mutton stew with potatoes and vegetables including carrots, broccoli, and/or dark-green leafy; gravy
2095	00-03862	A-00-03862		Irn Bru	27.03.00	Beverages - Carbonated soft drinks	92400000	Soft drink, NFS
2096	A-00-03864			Irn Bru, diet	27.03.00	Beverages - Carbonated soft drinks	92400000	Soft drink, NFS
2097	A-00-03872			Isostar Lemon	30.00.00	Nutrition Powders & drinks	95321000	Sports drink, NFS
2098	02-07704			JELLY LOW IN SUGAR MADE WITH WATER	24.02.00	Confectionary - Sugar based products	91501010	Gelatin dessert
2099	02-00552			JELLY PACKET CUBES	24.02.00	Confectionary - Sugar based products	91501010	Gelatin dessert
2100	A-00-01694	00-01694		JS reduced sugar digestive finger	04.01.00	Sweet cereal products - Biscuits	54102100	Graham crackers, reduced fat
2101	A-11-0177	11-0177		Jaffa cakes	04.01.00	Sweet cereal products - Biscuits	53118200	Cake, sponge, with icing or filling
2102	00-09600	A-00-09600		Jam Sandwich biscuits (jammy dodger)	04.01.00	Sweet cereal products - Biscuits	53239050	Cookie, shortbread, with icing or filling
2103	A-00-01039			Jam Tart, Wholemeal Flour	04.02.00	Sweet cereal products - Pastries, Buns & Pies	53300170	Pie, individual size or tart, NFS
2104	A-00-00087			Jam Tarts	04.02.00	Sweet cereal products - Pastries, Buns & Pies	53300170	Pie, individual size or tart, NFS
2105	11-0254	A-11-0254		Jam tarts	04.02.00	Sweet cereal products - Pastries, Buns & Pies	53300170	Pie, individual size or tart, NFS
2106	A-00-06355			Jam tarts 50	04.02.00	Sweet cereal products - Pastries, Buns & Pies	53300170	Pie, individual size or tart, NFS
2107	11-0255	A-11-0255		Jam tarts, retail	04.02.00	Sweet cereal products - Pastries, Buns & Pies	53300170	Pie, individual size or tart, NFS
2108	A-00-09962			Jam tarts, retail (MW6 Vit Eq)	04.02.00	Sweet cereal products - Pastries, Buns & Pies	53300170	Pie, individual size or tart, NFS
2109	A-00-06075			Jam tarts, retail 50	04.02.00	Sweet cereal products - Pastries, Buns & Pies	53300170	Pie, individual size or tart, NFS

2110	A-11-0256			Jam tarts, wholemeal	04.02.00	Sweet cereal products - Pastries, Buns & Pies	53300170	Pie, individual size or tart, NFS
2111	A-00-06076			Jam tarts, wholemeal 50	04.02.00	Sweet cereal products - Pastries, Buns & Pies	53300170	Pie, individual size or tart, NFS
2112	A-00-00849			Jam, Fruit, with Edible Seeds	22.01.00	Preserves - Jam & Marmalade	91402000	Jam, preserve, all flavors
2113	A-00-00850			Jam, Stone Fruit	22.01.00	Preserves - Jam & Marmalade	91402000	Jam, preserve, all flavors
2114	A-17-0073	17-0073		Jam, fruit with edible seeds	22.01.00	Preserves - Jam & Marmalade	91402000	Jam, preserve, all flavors
2115	A-17-0075	17-0075		Jam, reduced sugar	22.01.00	Preserves - Jam & Marmalade	91402000	Jam, preserve, all flavors
2116	A-17-0074	17-0074		Jam, stone fruit	22.01.00	Preserves - Jam & Marmalade	91402000	Jam, preserve, all flavors
2117	00-05404			Jelly Beans	24.02.00	Confectionary - Sugar based products	91700010	Candy, NFS
2118	00-05354			Jelly Pot Low Sugar	24.03.00	Confectionary - Sorbets & lollies	91501010	Gelatin dessert
2119	A-00-00109			Jelly, Packet, Cubes	24.02.00	Confectionary - Sugar based products	91501010	Gelatin dessert
2120	A-00-00111			Jelly, made with Milk	06.04.02	Dairy products - Ice cream & dairy desserts - reduced fat products	13110000	Ice cream, NFS
2121	A-00-00110			Jelly, made with Water	24.02.00	Confectionary - Sugar based products	91501010	Gelatin dessert
2122	00-09822	A-00-09822		Jelly, made with semi-skimmed milk	06.04.02	Dairy products - Ice cream & dairy desserts - reduced fat products	13130100	Light ice cream, NS as to flavor
2123	A-12-0240			Jelly, made with skimmed milk	06.04.02	Dairy products - Ice cream & dairy desserts - reduced fat products	13160420	Fat free ice cream, NS as to flavor
2124	12-0237	A-12-0237		Jelly, made with water	24.02.00	Confectionary - Sugar based products	91501010	Gelatin dessert
2125	A-12-0238	00-09821		Jelly, made with whole milk	06.04.01	Dairy products - Ice cream & dairy desserts - full fat products	13110000	Ice cream, NFS
2126	00-03061	A-00-03061		Just Right, Kelloggs	02.03.00	Breakfast cereals - Other breakfast cereals - low fibre	57100100	Cereal, ready-to-eat, NFS

2127	A-00-01357			Juvela G-F Fibre Loaf	03.04.00	Breads - Other bread	51808000	Bread, gluten free
2128	A-00-01358			Juvela G-F Fibre Loaf, Toasted	03.04.00	Breads - Other bread	51808000	Bread, gluten free
2129	02-10596			KELLOGGS SPECIAL K FRUIT AND NUT CLUSTERS	02.03.00	Breakfast cereals - Other breakfast cereals - low fibre	57100100	Cereal, ready-to-eat, NFS
2130	02-10330			KELLOGGS SPECIAL K OATS AND HONEY	02.01.00	Breakfast cereals - Oat based cereals	57000100	Cereal, oat, NFS
2131	02-08021			KINDER; BUENO, MAXI, HAPPY HIPPO, HAPPY HIPPO COCOA CREAM	24.01.00	Confectionary - Chocolate based products	91705010	Milk chocolate candy, plain
2132	00-05627			Kallo savoury rice cakes	25.04.00	Savoury Snacks - Savoury biscuits & crackers	54318500	Rice cake
2133	16-0296	A-00-00549	A-16-0296	Kedgeree	09.01.00	Fish & fish dishes - White fish, incl. tuna	58150520	Dukboki or Tteokbokki, Korean
2134	00-05429			Kelloggs Coco Pops Straws	02.03.00	Breakfast cereals - Other breakfast cereals - low fibre	57126000	Cereal (Kellogg's Cocoa Krispies)
2135	00-05608			Kelloggs Optivita Berry cereal	02.02.00	Breakfast cereals - Other breakfast cereals - high fibre (equal or >3g/40g portion)	57100100	Cereal, ready-to-eat, NFS
2136	A-00-03121	00-03121		Kelloggs Rice Krispie Squares	02.03.00	Breakfast cereals - Other breakfast cereals - low fibre	57339500	Cereal (Kellogg's Rice Krispies Treats Cereal)
2137	00-05880			Kelloggs Special K Sustain	02.02.00	Breakfast cereals - Other breakfast cereals - high fibre (equal or >3g/40g portion)	57100100	Cereal, ready-to-eat, NFS
2138	00-05524			Kelloggs coco pops Mega Munchers	02.03.00	Breakfast cereals - Other breakfast cereals - low fibre	57126000	Cereal (Kellogg's Cocoa Krispies)
2139	A-00-00365			Kidney, Lamb, Fried	14.02.00	Offal - Other offal & dishes, e.g. Haggis, faggots	25130000	Kidney, cooked
2140	A-00-06224			Kidney, Lamb, Fried (dripping) 50	14.02.00	Offal - Other offal & dishes, e.g. Haggis, faggots	25130000	Kidney, cooked
2141	A-00-00364			Kidney, Lamb, Raw	14.02.00	Offal - Other offal & dishes, e.g. Haggis, faggots	5027	Chicken, liver, all classes, raw
2142	A-00-00367			Kidney, Ox, Stewed	14.02.00	Offal - Other offal & dishes, e.g. Haggis, faggots	25130000	Kidney, cooked

2143	A-00-00368			Kidney, Pig, Raw	14.02.00	Offal - Other offal & dishes, e.g. Haggis, faggots	5027	Chicken, liver, all classes, raw
2144	A-00-00369			Kidney, Pig, Stewed	14.02.00	Offal - Other offal & dishes, e.g. Haggis, faggots	25130000	Kidney, cooked
2145	A-18-0403	18-0403		Kidney, lamb, fried	14.02.00	Offal - Other offal & dishes, e.g. Haggis, faggots	25130000	Kidney, cooked
2146	18-0405			Kidney, ox, stewed	14.02.00	Offal - Other offal & dishes, e.g. Haggis, faggots	25130000	Kidney, cooked
2147	18-0408	A-18-0408		Kidney, pig, stewed	14.02.00	Offal - Other offal & dishes, e.g. Haggis, faggots	25130000	Kidney, cooked
2148	A-00-00489			Kipper, Baked	09.02.00	Fish & fish dishes - Oily fish	26119190	Herring, smoked, kippered
2149	A-00-00490			Kipper, Baked (Weighed with Bones)	09.02.00	Fish & fish dishes - Oily fish	26119190	Herring, smoked, kippered
2150	16-0190	A-16-0190		Kipper, boil in bag, boiled	09.02.00	Fish & fish dishes - Oily fish	26119190	Herring, smoked, kippered
2151	16-0188	A-16-0188		Kipper, grilled	09.02.00	Fish & fish dishes - Oily fish	26119190	Herring, smoked, kippered
2152	17-0093	A-17-0093		Kit Kat	24.01.00	Confectionary - Chocolate based products	91705030	Kit Kat
2153	A-00-01360			Kiwi Fruit (Chinese Gooseberry)	18.01.00	Fruit - Fresh	63126500	Kiwi fruit, raw
2154	14-0123			Kiwi fruit	18.01.00	Fruit - Fresh	63126500	Kiwi fruit, raw
2155	A-00-09924			Kiwi fruit (MW6 carq)	18.01.00	Fruit - Fresh	63126500	Kiwi fruit, raw
2156	A-12-0213			Knickerbocker glory	06.04.02	Dairy products - Ice cream & dairy desserts - reduced fat products	13121100	Ice cream sundae, fruit topping, with whipped cream
2157	13-0261			Kohl rabi, raw	15.04.00	Vegetables - Other	75112000	Kohlrabi, raw
2158	14-0125			Kumquats, raw	18.01.00	Fruit - Fresh	61110010	Kumquat, raw
2159	02-09493			LACTOSE FREE SEMI SKIMMED MILK	05.02.00	Milk - Semi-skimmed milk	11100000	Milk, NFS
2160	02-08344			LAGER LOW CARBOHYDRATE PILS TYPE NOT CANNED (4.3% ABV)	27.01.03	Beverages - Alcohol - Beer	93101000	Beer

2161	02-02368			LAGER: NON PREMIUM CANNED (APPROX.3.3% ABV)	27.01.03	Beverages - Alcohol - Beer	93101000	Beer
2162	02-05217			LAMB BURGER	10.02.00	Meat - red - Lamb & dishes	23132000	Lamb, ground or patty, cooked
2163	02-00984			LAMB CHUMP CHOPS STEAKS GRILLED LEAN & FAT NO BONE	10.02.00	Meat - red - Lamb & dishes	23104010	Lamb, loin chop, cooked, lean and fat eaten
2164	02-00986			LAMB CHUMP CHOPS STEAKS GRILLED LEAN ONLY NO BONE	10.02.00	Meat - red - Lamb & dishes	23104020	Lamb, loin chop, cooked, lean only eaten
2165	02-09477			LAMB KORMA WITH CREAM/COCONUT SAUCE NO RICE	10.02.00	Meat - red - Lamb & dishes	27130100	Lamb or mutton curry
2166	02-03248			LAMB SHEPHERDS PIE E.G. WW	10.02.00	Meat - red - Lamb & dishes	27330010	Shepherd's pie with lamb
2167	02-09547			LAMB SHISH KEBAB FRIED IN VEGETABLE OIL	10.02.00	Meat - red - Lamb & dishes	27430610	Lamb shish kabob with vegetables, excluding potatoes
2168	02-02656			LAMBS LIVER WITH LOSSES	14.01.00	Offal - Liver & dishes	25110120	Beef liver, braised
2169	02-05249			LASAGNE MADE WITH PORK	10.03.00	Meat - red - Pork & dishes	58130011	Lasagna with meat
2170	02-03187			LASAGNE, REDUCED FAT, RETAIL	01.02.00	Cereals & cereal dishes - Pasta & pasta dishes	58301050	Lasagna with cheese and meat sauce, diet frozen meal
2171	02-03901			LASSI, SWEETENED	06.03.02	Dairy products - Yoghurt & drinking yoghurts, incl. buttermilk and probiotics - reduced or low fat products	11436000	Yogurt, liquid
2172	02-08058			LATTE (ESPRESSO AND SKIMMED MILK) TAKEAWAY ONLY	27.06.00	Beverages - Coffee	92101900	Coffee, Latte

2173	02-08053			LATTE (ESPRESSO AND WHOLE MILK) TAKEAWAY ONLY	27.06.00	Beverages - Coffee	92101900	Coffee, Latte
2174	02-10484			LAUGHING COW EXTRA LIGHT CHEESE TRIANGLES	06.02.00	Dairy products - Cheese, incl. cottage cheese	14107030	Cheese, Mozzarella, part skim
2175	02-06651			LEMON CHICKEN - CHICKEN BREASTS IN SAUCE	11.01.00	Meat - white - Chicken & turkey & dishes	27146350	Orange chicken
2176	02-00346			LEMON CURD TART	04.03.00	Sweet cereal products - Cereal based puddings (not milk)	53224250	Cookie, lemon bar
2177	02-08706			LEMON TEA POWDER REDUCED SWEETNESS ADDED VITAMIN C	27.07.00	Beverages - Tea	92302000	Tea, hot, leaf, black
2178	02-03556			LIDL ACE VITAMIN FRUIT JUICE DRINK	27.02.02	Beverages - Fruit based drinks - Fruit juice drinks	92531030	Fruit juice drink (Sunny D)
2179	02-08330			LIGHT CREAM CRACKERS LOW FAT	25.04.00	Savoury Snacks - Savoury biscuits & crackers	54307000	Crackers, matzo
2180	02-02429			LIME PICKLE OILY	22.02.00	Preserves - Chutney & Pickles (incl. gherkins, pickled onions etc)	61116010	Lime, raw
2181	02-09741			LINSEEDS	19.00.00	Nuts & Seeds (incl. peanut butter)	43104000	Flax seeds
2182	02-08299			LIQUID TABLE TOP SWEETENERS	26.03.00	Miscellaneous - Artificial sweeteners	91200000	Sugar substitute, powder, NFS
2183	02-01258			LIVER PATE PLASTIC WRAPPED	14.01.00	Offal - Liver & dishes	25112200	Liver paste or pate, chicken
2184	00-09983	A-00-09983		LO-SALT added at table MARKER DUMMY	26.02.00	Miscellaneous - Salt and salt substitutes	2047	Salt, table
2185	02-01382			LOW FAT BEEFBURGERS GRILLED	13.00.00	Sausages & burgers & kebab	23563	Beef, ground, 90% lean meat / 10% fat, patty, cooked, broiled
2186	02-08153			LOW FAT CONDENSED SOUP NOT MADE UP	20.01.00	Soups - Canned & fresh & homemade	58400000	Soup, NFS

2187	02-08041			LOW FAT FRUIT YOGURT ADDED FIBRE (LASTING SATISFACTION)	06.03.02	Dairy products - Yoghurt & drinking yoghurts, incl. buttermilk and probiotics - reduced or low fat products	11400000	Yogurt, NFS
2188	02-10590			LOW FAT MOZZARELLA	06.02.00	Dairy products - Cheese, incl. cottage cheese	14107030	Cheese, Mozzarella, part skim
2189	02-02691			LOW FAT NOT BAKED CRISPS	25.01.00	Savoury Snacks - Potato based snacks	71201050	Potato chips, reduced fat
2190	02-10049			LOW FAT SPREAD (26-39% FAT) POLYUNSATURATED	08.04.03	Fats - Plant based fats (solid) - Low fat	81102000	Margarine, NFS
2191	02-10050			LOW FAT SPREAD (26-39% FAT) POLYUNSATURATED, FORTIFIED WITH B6, B12, FOLIC ACID	08.04.03	Fats - Plant based fats (solid) - Low fat	81102000	Margarine, NFS
2192	02-03149			LOW FAT TRIFLE E.G. ST IVEL	06.04.02	Dairy products - Ice cream & dairy desserts - reduced fat products	13252600	Tiramisu
2193	02-02730			LOW FAT YOGURT, CONTAINING FRUIT, BENECOL ONLY	06.03.02	Dairy products - Yoghurt & drinking yoghurts, incl. buttermilk and probiotics - reduced or low fat products	11430000	Yogurt, NS as to type of milk, fruit
2194	02-08867			LOW PROTEIN GLUTEN FREE WAFER CREAM BISCUITS	04.01.00	Sweet cereal products - Biscuits	53261000	Cookie, gluten free
2195	02-08226			LUXURY CHOC ICES CONTAINING CARAMEL OR NUTS EG MARS SNICKERS	06.04.01	Dairy products - Ice cream & dairy desserts - full fat products	13120130	Ice cream bar or stick, rich ice cream, chocolate covered, with nuts
2196	02-10272			LUXURY FRESH CUSTARD READY TO SERVE PURCHASED	04.04.00	Sweet cereal products - Milk based puddings	13210300	Custard
2197	00-05908			Lactofree soft white cheese	06.02.00	Dairy products - Cheese, incl. cottage cheese	14107030	Cheese, Mozzarella, part skim
2198	A-17-0211			Lager	27.01.03	Beverages - Alcohol - Beer	93101000	Beer
2199	A-00-03813			Lager 82/1900	27.01.03	Beverages - Alcohol - Beer	93101000	Beer

2200	17-0211			Lager, 5% ABV	27.01.03	Beverages - Alcohol - Beer	93101000	Beer
2201	A-00-00896			Lager, Bottled Beer	27.01.03	Beverages - Alcohol - Beer	93101000	Beer
2202	17-0212	A-17-0212		Lager, alcohol-free, Kaliber and Barbican	27.01.06	Beverages - Alcohol - Low alcohol beer	93101000	Beer
2203	A-17-0213			Lager, low alcohol	27.01.06	Beverages - Alcohol - Low alcohol beer	93101000	Beer
2204	17-0213			Lager, low alcohol (0.6% ABV)	27.01.06	Beverages - Alcohol - Low alcohol beer	93101000	Beer
2205	A-17-0214			Lager, premium	27.01.03	Beverages - Alcohol - Beer	93101000	Beer
2206	17-0214			Lager, premium (8.7%ABV)	27.01.03	Beverages - Alcohol - Beer	93101000	Beer
2207	00-09508	A-00-09508		Lamb Balti	10.02.00	Meat - red - Lamb & dishes	27130100	Lamb or mutton curry
2208	A-00-00277			Lamb Chops, Grilled, Lean Only	10.02.00	Meat - red - Lamb & dishes	23101020	Lamb chop, NS as to cut, cooked, lean only eaten
2209	A-00-00278			Lamb Chops, Grilled, Lean Only (Weighed with Fat and Bone)	10.02.00	Meat - red - Lamb & dishes	23101020	Lamb chop, NS as to cut, cooked, lean only eaten
2210	A-00-00275			Lamb Chops, Grilled, Lean and Fat	10.02.00	Meat - red - Lamb & dishes	23101010	Lamb chop, NS as to cut, cooked, lean and fat eaten
2211	A-00-00276			Lamb Chops, Grilled, Lean and Fat (Weighed with Bone)	10.02.00	Meat - red - Lamb & dishes	23101010	Lamb chop, NS as to cut, cooked, lean and fat eaten
2212	A-00-00282			Lamb Cutlets, Grilled, Lean Only	10.02.00	Meat - red - Lamb & dishes	23101020	Lamb chop, NS as to cut, cooked, lean only eaten
2213	A-00-00283			Lamb Cutlets, Grilled, Lean Only (Weighed with Fat and Bone)	10.02.00	Meat - red - Lamb & dishes	23101020	Lamb chop, NS as to cut, cooked, lean only eaten
2214	A-00-00280			Lamb Cutlets, Grilled, Lean and Fat	10.02.00	Meat - red - Lamb & dishes	23101010	Lamb chop, NS as to cut, cooked, lean and fat eaten
2215	A-00-00281			Lamb Cutlets, Grilled, Lean and Fat (Weighed with Bone)	10.02.00	Meat - red - Lamb & dishes	23101010	Lamb chop, NS as to cut, cooked, lean and fat eaten

2216	A-00-00279			Lamb Cutlets, Raw, Lean and Fat	10.02.00	Meat - red - Lamb & dishes	17035	Lamb, domestic, shoulder, whole (arm and blade), separable lean and fat, trimmed to 1/4" fat, choice, raw
2217	A-00-03741			Lamb Fat (100% fat) 82/982	08.03.00	Fats - Animal based fats (solid)	81201000	Animal fat or drippings
2218	A-00-01306			Lamb Kheema	10.02.00	Meat - red - Lamb & dishes	27130100	Lamb or mutton curry
2219	A-00-09504	00-09504		Lamb Rogan Josh	10.02.00	Meat - red - Lamb & dishes	27130100	Lamb or mutton curry
2220	A-19-0225			Lamb biryani	10.02.00	Meat - red - Lamb & dishes	27213010	Biryani with meat
2221	A-00-01375			Lamb chop, average, grilled	10.02.00	Meat - red - Lamb & dishes	23101000	Lamb chop, NS as to cut, cooked, NS as to fat eaten
2222	A-19-0227			Lamb curry, made with canned curry sauce	10.02.00	Meat - red - Lamb & dishes	27130100	Lamb or mutton curry
2223	00-03454	A-00-03454		Lamb dripping	08.03.00	Fats - Animal based fats (solid)	81201000	Animal fat or drippings
2224	A-00-00273			Lamb, Breast, Roast, Lean Only	10.02.00	Meat - red - Lamb & dishes	23110000	Lamb, ribs, cooked, lean only eaten
2225	A-00-00272			Lamb, Breast, Roast, Lean and Fat	10.02.00	Meat - red - Lamb & dishes	23110050	Lamb, ribs, cooked, lean and fat eaten
2226	A-00-00274			Lamb, Chops, Loin, Raw, Lean and Fat	10.02.00	Meat - red - Lamb & dishes	17035	Lamb, domestic, shoulder, whole (arm and blade), separable lean and fat, trimmed to 1/4" fat, choice, raw
2227	A-00-00284			Lamb, Leg, Raw, Lean and Fat	10.02.00	Meat - red - Lamb & dishes	17035	Lamb, domestic, shoulder, whole (arm and blade), separable lean and fat, trimmed to 1/4" fat, choice, raw
2228	A-00-00286			Lamb, Leg, Roast, Lean Only	10.02.00	Meat - red - Lamb & dishes	17014	Lamb, domestic, leg, whole (shank and sirloin), separable lean only, trimmed to 1/4" fat, choice, cooked, roasted

2229	A-00-00285			Lamb, Leg, Roast, Lean and Fat	10.02.00	Meat - red - Lamb & dishes	17012	Lamb, domestic, leg, whole (shank and sirloin), separable lean and fat, trimmed to 1/4" fat, choice, cooked, roasted
2230	A-00-00289			Lamb, Scrag and Neck, Stewed, Lean Only	10.02.00	Meat - red - Lamb & dishes	23107020	Lamb, shoulder chop, cooked, lean only eaten
2231	A-00-00290			Lamb, Scrag and Neck, Stewed, Lean Only (with Fat and Bone)	10.02.00	Meat - red - Lamb & dishes	23107020	Lamb, shoulder chop, cooked, lean only eaten
2232	A-00-00288			Lamb, Scrag and Neck, Stewed, Lean and Fat	10.02.00	Meat - red - Lamb & dishes	23107010	Lamb, shoulder chop, cooked, lean and fat eaten
2233	A-00-00293			Lamb, Shoulder, Roast, Lean Only	10.02.00	Meat - red - Lamb & dishes	23107020	Lamb, shoulder chop, cooked, lean only eaten
2234	A-00-00292			Lamb, Shoulder, Roast, Lean and Fat	10.02.00	Meat - red - Lamb & dishes	23107010	Lamb, shoulder chop, cooked, lean and fat eaten
2235	A-18-0107	18-0107		Lamb, best end neck cutlets, grilled, lean	10.02.00	Meat - red - Lamb & dishes	23107020	Lamb, shoulder chop, cooked, lean only eaten
2236	A-18-0109	18-0109		Lamb, best end neck cutlets, grilled, lean & fat	10.02.00	Meat - red - Lamb & dishes	23107010	Lamb, shoulder chop, cooked, lean and fat eaten
2237	18-0113			Lamb, breast, roasted, lean	10.02.00	Meat - red - Lamb & dishes	23110000	Lamb, ribs, cooked, lean only eaten
2238	18-0114	A-18-0114		Lamb, breast, roasted, lean & fat	10.02.00	Meat - red - Lamb & dishes	23110050	Lamb, ribs, cooked, lean and fat eaten
2239	18-0124			Lamb, leg chops, grilled, lean & fat	10.02.00	Meat - red - Lamb & dishes	17012	Lamb, domestic, leg, whole (shank and sirloin), separable lean and fat, trimmed to 1/4" fat, choice, cooked, roasted

2240	18-0134			Lamb, leg steaks, grilled, lean & fat	10.02.00	Meat - red - Lamb & dishes	17012	Lamb, domestic, leg, whole (shank and sirloin), separable lean and fat, trimmed to 1/4" fat, choice, cooked, roasted
2241	18-0137	A-18-0137		Lamb, leg, whole, roasted well done, lean	10.02.00	Meat - red - Lamb & dishes	17014	Lamb, domestic, leg, whole (shank and sirloin), separable lean only, trimmed to 1/4" fat, choice, cooked, roasted
2242	A-18-0138	18-0138		Lamb, leg, whole, roasted well done, lean & fat	10.02.00	Meat - red - Lamb & dishes	17012	Lamb, domestic, leg, whole (shank and sirloin), separable lean and fat, trimmed to 1/4" fat, choice, cooked, roasted
2243	18-0141	A-18-0141		Lamb, loin chops, grilled, lean	10.02.00	Meat - red - Lamb & dishes	23104020	Lamb, loin chop, cooked, lean only eaten
2244	18-0143	A-18-0143		Lamb, loin chops, grilled, lean & fat	10.02.00	Meat - red - Lamb & dishes	23104010	Lamb, loin chop, cooked, lean and fat eaten
2245	18-0159			Lamb, mince, stewed	10.02.00	Meat - red - Lamb & dishes	23132000	Lamb, ground or patty, cooked
2246	00-01374	A-00-01374		Lamb, roast, average	10.02.00	Meat - red - Lamb & dishes	23120100	Lamb, roast, cooked, NS as to fat eaten
2247	18-0179	A-18-0179		Lamb, shoulder, whole, roasted, lean	10.02.00	Meat - red - Lamb & dishes	23107020	Lamb, shoulder chop, cooked, lean only eaten
2248	A-18-0180	18-0180		Lamb, shoulder, whole, roasted, lean & fat	10.02.00	Meat - red - Lamb & dishes	23107010	Lamb, shoulder chop, cooked, lean and fat eaten

2249	19-0231	A-19-0231		Lamb/Beef hot pot with potatoes, chilled/frozen, retail, reheated	10.04.00	Meat - red - Other red meat, e.g. rabbit, venison	27330110	Lamb or mutton stew with potatoes and vegetables excluding carrots, broccoli, and dark-green leafy; gravy
2250	A-17-0010	A-00-00185	17-0010	Lard	08.03.00	Fats - Animal based fats (solid)	81201000	Animal fat or drippings
2251	11-0208	A-11-0208		Lardy cake	04.02.00	Sweet cereal products - Pastries, Buns & Pies	51129010	Bread, raisin
2252	A-00-06060			Lardy cake 50	04.02.00	Sweet cereal products - Pastries, Buns & Pies	51129010	Bread, raisin
2253	11-0052	A-11-0052		Lasagna, boiled	01.02.00	Cereals & cereal dishes - Pasta & pasta dishes	56130000	Pasta, cooked
2254	19-0237	A-19-0237	A-00-01002	Lasagne	10.01.00	Meat - red - Beef & veal & dishes	58130011	Lasagna with meat
2255	A-19-0238	19-0238		Lasagne, chilled/frozen, reheated	10.01.00	Meat - red - Beef & veal & dishes	58130011	Lasagna with meat
2256	A-15-0185			Lasagne, spinach	01.02.00	Cereals & cereal dishes - Pasta & pasta dishes	56104000	Pasta, vegetable, cooked
2257	15-0187	A-15-0187		Lasagne, vegetable	15.04.00	Vegetables - Other	58301110	Vegetable lasagna, frozen meal
2258	A-15-0189	15-0189		Lasagne, vegetable, retail	15.04.00	Vegetables - Other	58301110	Vegetable lasagna, frozen meal
2259	00-05634			Lasagne,egg,spinach flavoured, cooked	01.02.00	Cereals & cereal dishes - Pasta & pasta dishes	20112	Noodles, egg, spinach, enriched, cooked
2260	A-15-0190	A-00-00600		Laverbread	15.04.00	Vegetables - Other	75232100	Seaweed, cooked, NS as to fat added in cooking
2261	A-00-00602			Leeks, Boiled	15.04.00	Vegetables - Other	75218400	Leek, cooked, NS as to fat added in cooking
2262	A-00-00601			Leeks, Raw	15.04.00	Vegetables - Other	75112500	Leek, raw
2263	13-0265			Leeks, boiled in unsalted water	15.04.00	Vegetables - Other	75218400	Leek, cooked, NS as to fat added in cooking
2264	A-00-09919			Leeks, boiled in unsalted water (MW6 carq)	15.04.00	Vegetables - Other	75218400	Leek, cooked, NS as to fat added in cooking
2265	13-0263			Leeks, raw	15.04.00	Vegetables - Other	75112500	Leek, raw

2266	A-00-01040			Lemon Curd Tart, Homemade Filling	04.03.00	Sweet cereal products - Cereal based puddings (not milk)	53224250	Cookie, lemon bar
2267	A-00-00852			Lemon Curd, Home-made	23.02.00	Sugars - Other, incl. syrups, honey	61113500	Lemon pie filling
2268	A-00-00851			Lemon Curd, Starch Base	23.02.00	Sugars - Other, incl. syrups, honey	61113500	Lemon pie filling
2269	A-00-00751			Lemon Juice, Fresh	27.02.01	Beverages - Fruit based drinks - Pure fruit juice & smoothies	61210000	Orange juice, 100%, NFS
2270	A-00-00112			Lemon Meringue Pie	04.02.00	Sweet cereal products - Pastries, Buns & Pies	53381000	Pie, lemon meringue
2271	A-00-00462			Lemon Sole, Fried	09.01.00	Fish & fish dishes - White fish, incl. tuna	26115120	Flounder, baked or broiled, made with oil
2272	A-00-00463			Lemon Sole, Fried (Weighed with Bones)	09.01.00	Fish & fish dishes - White fish, incl. tuna	26115120	Flounder, baked or broiled, made with oil
2273	A-00-06252			Lemon Sole, Fried (Weighed with bones) (dripping) 50	09.01.00	Fish & fish dishes - White fish, incl. tuna	26115120	Flounder, baked or broiled, made with oil
2274	A-00-00461			Lemon Sole, Raw	09.01.00	Fish & fish dishes - White fish, incl. tuna	26115000	Flounder, raw
2275	A-00-00464			Lemon Sole, Steamed	09.01.00	Fish & fish dishes - White fish, incl. tuna	26115160	Flounder, steamed or poached
2276	A-00-00465			Lemon Sole, Steamed (Weighed with Bones and Skin)	09.01.00	Fish & fish dishes - White fish, incl. tuna	26115160	Flounder, steamed or poached
2277	A-17-0076	17-0076		Lemon curd	23.02.00	Sugars - Other, incl. syrups, honey	61113500	Lemon pie filling
2278	17-0077	A-17-0077		Lemon curd, homemade	23.01.00	Sugars - Pure sugars	61113500	Lemon pie filling
2279	A-14-0277	14-0277		Lemon juice, fresh	27.02.01	Beverages - Fruit based drinks - Pure fruit juice & smoothies	61210000	Orange juice, 100%, NFS
2280	A-00-09611	00-09611	A-11-0316	Lemon meringue pie	04.02.00	Sweet cereal products - Pastries, Buns & Pies	53381000	Pie, lemon meringue
2281	A-00-06091			Lemon meringue pie 50	04.02.00	Sweet cereal products - Pastries, Buns & Pies	53381000	Pie, lemon meringue
2282	A-14-0127	14-0127		Lemon peel	18.01.00	Fruit - Fresh	91708000	Fruit peel, candied

2283	16-0087	A-16-0087		Lemon sole, goujons, baked	09.01.00	Fish & fish dishes - White fish, incl. tuna	26115123	Flounder, baked or broiled, made without fat
2284	A-16-0088			Lemon sole, goujons, fried in blended oil	09.01.00	Fish & fish dishes - White fish, incl. tuna	26115140	Flounder, coated, fried, made with oil
2285	16-0083	A-16-0083		Lemon sole, grilled	09.01.00	Fish & fish dishes - White fish, incl. tuna	26115123	Flounder, baked or broiled, made without fat
2286	A-16-0085	16-0085		Lemon sole, steamed	09.01.00	Fish & fish dishes - White fish, incl. tuna	26115160	Flounder, steamed or poached
2287	17-0179	A-17-0179		Lemonade	27.03.00	Beverages - Carbonated soft drinks	92432000	Fruit juice drink, citrus, carbonated
2288	A-00-00881			Lemonade, Bottled	27.03.00	Beverages - Carbonated soft drinks	92432000	Fruit juice drink, citrus, carbonated
2289	A-00-00750			Lemons, Whole	18.01.00	Fruit - Fresh	61113010	Lemon, raw
2290	A-00-00943			Lentil Soup	20.01.00	Soups - Canned & fresh & homemade	41603010	Lentil soup, home recipe, canned, or ready-to-serve
2291	15-0198			Lentil roast	16.01.00	Pulses/Lentils - Pulses/lentils	41306000	Loaf, lentil
2292	A-15-0199			Lentil roast, with egg	16.01.00	Pulses/Lentils - Pulses/lentils	41306000	Loaf, lentil
2293	A-17-0264	17-0264		Lentil soup	20.01.00	Soups - Canned & fresh & homemade	41603010	Lentil soup, home recipe, canned, or ready-to-serve
2294	A-17-0263	17-0263		Lentil soup, canned	20.01.00	Soups - Canned & fresh & homemade	41603010	Lentil soup, home recipe, canned, or ready-to-serve
2295	A-00-00605			Lentils Masur, Dahl, Cooked	16.01.00	Pulses/Lentils - Pulses/lentils	41101000	Beans, dry, cooked, NS as to type and as to fat added in cooking
2296	A-00-00603			Lentils, Raw	16.01.00	Pulses/Lentils - Pulses/lentils	16069	Lentils, raw
2297	A-00-00604			Lentils, Split, Boiled	16.01.00	Pulses/Lentils - Pulses/lentils	41101000	Beans, dry, cooked, NS as to type and as to fat added in cooking

2298	13-0090			Lentils, green and brown, whole, dried, boiled in salted water	16.01.00	Pulses/Lentils - Pulses/lentils	41101000	Beans, dry, cooked, NS as to type and as to fat added in cooking
2299	13-0092	A-13-0092		Lentils, red, split, dried, boiled in unsalted water	16.01.00	Pulses/Lentils - Pulses/lentils	41101000	Beans, dry, cooked, NS as to type and as to fat added in cooking
2300	13-0268			Lettuce, Cos, raw	15.04.00	Vegetables - Other	72116000	Romaine lettuce, raw
2301	13-0269	A-13-0269		Lettuce, Iceberg, raw	15.04.00	Vegetables - Other	75113000	Lettuce, raw
2302	A-00-00606			Lettuce, Raw	15.03.00	Vegetables - Yellow & red & dark green leafy vegetables	75113000	Lettuce, raw
2303	13-0266			Lettuce, average, raw	15.04.00	Vegetables - Other	75113000	Lettuce, raw
2304	A-00-09920			Lettuce, average, raw (MW6 carq)	15.04.00	Vegetables - Other	75113000	Lettuce, raw
2305	13-0267	A-13-0267		Lettuce, butterhead, raw	15.04.00	Vegetables - Other	75113060	Lettuce, Boston, raw
2306	00-05917			Light Spreadable Butter (60% fat), unsalted	08.01.00	Fats - Butter	81100500	Butter, NFS
2307	00-05821			Light Vegetable Suet	08.04.02	Fats - Plant based fats (solid) - Reduced fat	81102000	Margarine, NFS
2308	A-00-00882			Lime Juice Cordial, Undiluted	27.03.00	Beverages - Carbonated soft drinks	93201000	Cordial or liqueur
2309	A-17-0200	17-0200		Lime juice cordial, concentrated	27.02.03	Beverages - Fruit based drinks - Squashes & fruit concentrates	91301050	Fruit syrup
2310	17-0201	A-17-0201		Lime juice cordial, concentrated, made up	27.02.03	Beverages - Fruit based drinks - Squashes & fruit concentrates	92510610	Fruit juice drink
2311	A-14-0279	14-0279		Lime juice, fresh	27.02.01	Beverages - Fruit based drinks - Pure fruit juice & smoothies	61210000	Orange juice, 100%, NFS
2312	A-00-01245			Limes, Stewed, with Salt	18.02.00	Fruit - Canned & cooked	61122300	Orange, mandarin, canned or frozen, NS as to sweetened or unsweetened; sweetened, NS as to type of sweetener
2313	A-00-03792			Limmits - slimming biscuits 82/1434	04.01.00	Sweet cereal products - Biscuits	54102100	Graham crackers, reduced fat

2314	00-05516			Linda McCartney deep country pie	15.04.00	Vegetables - Other	41812400	Vegetarian pot pie
2315	17-0244	A-17-0244		Liqueurs, high strength inc. Pernod, Drambuie, Cointreau	27.01.04	Beverages - Alcohol - Spirits & Liqueur	93501000	Brandy
2316	17-0245	A-17-0245		Liqueurs, low-medium strength inc Cherry brandy, Tia Maria	27.01.04	Beverages - Alcohol - Spirits & Liqueur	93501000	Brandy
2317	A-00-00863			Liquorice Allsorts	24.02.00	Confectionary - Sugar based products	91700010	Candy, NFS
2318	17-0112	A-17-0112		Liquorice allsorts	24.02.00	Confectionary - Sugar based products	91700010	Candy, NFS
2319	17-0113	A-17-0113		Liquorice shapes	24.02.00	Confectionary - Sugar based products	91700010	Candy, NFS
2320	00-03920			Little Rachels Organic Fruit Yogurt/My First Yogurt	28.02.04	Baby & infant foods/drinks - Ready meals - Desserts	67404500	Mixed fruit yogurt dessert, baby food, strained
2321	A-00-03776			Liver Pate, shop bought (pork+turkey/Ardennes/Brussels) 82/1418	14.01.00	Offal - Liver & dishes	25112200	Liver paste or pate, chicken
2322	A-00-00404			Liver Sausage	14.01.00	Offal - Liver & dishes	25230560	Liverwurst
2323	A-19-0241	19-0241		Liver and onions, stewed	14.01.00	Offal - Liver & dishes	27460750	Liver, beef or calves, and onions
2324	A-19-0106	19-0106		Liver sausage	14.01.00	Offal - Liver & dishes	25230560	Liverwurst
2325	A-00-00371			Liver, Calf, Raw	14.01.00	Offal - Liver & dishes	5027	Chicken, liver, all classes, raw
2326	A-00-00374			Liver, Chicken, Fried	14.01.00	Offal - Liver & dishes	25110450	Chicken liver, fried
2327	A-00-00373			Liver, Chicken, Raw	14.01.00	Offal - Liver & dishes	5027	Chicken, liver, all classes, raw
2328	A-00-00376			Liver, Lamb, Fried	14.01.00	Offal - Liver & dishes	25110140	Beef liver, fried
2329	A-00-06218			Liver, Lamb, Fried (dripping) 50	14.01.00	Offal - Liver & dishes	25110140	Beef liver, fried
2330	A-00-00375			Liver, Lamb, Raw	14.01.00	Offal - Liver & dishes	5027	Chicken, liver, all classes, raw

2331	A-00-00377			Liver, Ox, Raw	14.01.00	Offal - Liver & dishes	5027	Chicken, liver, all classes, raw
2332	A-00-00378			Liver, Ox, Stewed	14.01.00	Offal - Liver & dishes	25110120	Beef liver, braised
2333	A-00-00379			Liver, Pig, Raw	14.01.00	Offal - Liver & dishes	5027	Chicken, liver, all classes, raw
2334	A-00-00380			Liver, Pig, Stewed	14.01.00	Offal - Liver & dishes	25110120	Beef liver, braised
2335	A-00-01086			Liver, Stewed with Gravy and Onion	14.01.00	Offal - Liver & dishes	27460750	Liver, beef or calves, and onions
2336	A-18-0410	18-0410		Liver, calf, fried	14.01.00	Offal - Liver & dishes	25110140	Beef liver, fried
2337	A-18-0412	18-0412		Liver, chicken, fried	14.01.00	Offal - Liver & dishes	25110450	Chicken liver, fried
2338	18-0411			Liver, chicken, raw	14.01.00	Offal - Liver & dishes	5027	Chicken, liver, all classes, raw
2339	18-0414	A-18-0414		Liver, lamb, fried	14.01.00	Offal - Liver & dishes	25110140	Beef liver, fried
2340	18-0416	A-18-0416		Liver, ox, stewed	14.01.00	Offal - Liver & dishes	25110120	Beef liver, braised
2341	A-18-0418	18-0418		Liver, pig, stewed	14.01.00	Offal - Liver & dishes	25110120	Beef liver, braised
2342	A-00-00521			Lobster, Boiled	09.03.00	Fish & fish dishes - Shellfish	26311160	Lobster, steamed or boiled
2343	A-00-00522			Lobster, Boiled (Weighed with Shell)	09.03.00	Fish & fish dishes - Shellfish	26311160	Lobster, steamed or boiled
2344	16-0236	A-16-0236		Lobster, boiled	09.03.00	Fish & fish dishes - Shellfish	26311160	Lobster, steamed or boiled
2345	A-00-00755			Loganberries, Canned	18.02.00	Fruit - Canned & cooked	63201110	Blackberries, cooked or canned, NS as to sweetened or unsweetened; sweetened, NS as to type of sweetener
2346	A-00-00752			Loganberries, Raw	18.01.00	Fruit - Fresh	63200100	Berries, raw, NFS
2347	A-00-00754			Loganberries, Stewed with Sugar	18.02.00	Fruit - Canned & cooked	63201110	Blackberries, cooked or canned, NS as to sweetened or unsweetened; sweetened, NS as to type of sweetener

2348	A-00-00753			Loganberries, Stewed without Sugar	18.02.00	Fruit - Canned & cooked	63201110	Blackberries, cooked or canned, NS as to sweetened or unsweetened; sweetened, NS as to type of sweetener
2349	14-0133	A-14-0133		Loganberries, raw	18.01.00	Fruit - Fresh	63200100	Berries, raw, NFS
2350	A-14-0134			Loganberries, stewed with sugar	18.02.00	Fruit - Canned & cooked	63201110	Blackberries, cooked or canned, NS as to sweetened or unsweetened; sweetened, NS as to type of sweetener
2351	00-03865	A-00-03865		Low Alcohol Wine	27.01.01	Beverages - Alcohol - Wine	93401010	Wine, table, red
2352	A-00-01382	00-01382		Low Calorie coke	27.03.00	Beverages - Carbonated soft drinks	92400000	Soft drink, NFS
2353	00-03927	A-00-03927		Low Fat Cheese and Tomato Pizza	01.01.00	Cereals & cereal dishes - Pizza	58106200	Pizza, cheese, from frozen, thin crust
2354	00-05716			Low Fat Chicken Tikka Masala with Rice, ready meal	11.01.00	Meat - white - Chicken & turkey & dishes	27146150	Chicken curry
2355	A-00-01392			Low alcohol lager or beer	27.01.06	Beverages - Alcohol - Low alcohol beer	93101000	Beer
2356	A-00-03818			Low calorie cola/Diet Pepsi 82/1913	27.03.00	Beverages - Carbonated soft drinks	92400000	Soft drink, NFS
2357	17-0265	A-17-0265		Low calorie soup, canned	20.01.00	Soups - Canned & fresh & homemade	58400000	Soup, NFS
2358	A-00-03817			Low calorie squash, diluted 82/1912	27.02.03	Beverages - Fruit based drinks - Squashes & fruit concentrates	92510610	Fruit juice drink
2359	12-0192	A-12-0192		Low calorie yogurt	06.03.02	Dairy products - Yoghurt & drinking yoghurts, incl. buttermilk and probiotics - reduced or low fat products	11400000	Yogurt, NFS
2360	00-03928			Low fat humous, houmous	16.01.00	Pulses/Lentils - Pulses/lentils	41205070	Hummus, plain

2361	00-03471			Low fat olive spread, 38% fat (eg Tesco, Sainsburys Olive Light)	08.04.03	Fats - Plant based fats (solid) - Low fat	81102000	Margarine, NFS
2362	00-03929			Low fat salad dressing	21.01.00	Sauces & accompaniment - Dressings & Mayonnaise	83200100	Salad dressing, light, NFS
2363	00-09725	A-00-09725		Low fat soft cheese (less than 10%)	06.02.00	Dairy products - Cheese, incl. cottage cheese	14303010	Cheese, cream, light or lite
2364	A-12-0189			Low fat yogurt, flavoured	06.03.02	Dairy products - Yoghurt & drinking yoghurts, incl. buttermilk and probiotics - reduced or low fat products	11400000	Yogurt, NFS
2365	00-09650	A-00-09650	A-12-0190	Low fat yogurt, fruit	06.03.02	Dairy products - Yoghurt & drinking yoghurts, incl. buttermilk and probiotics - reduced or low fat products	11430000	Yogurt, NS as to type of milk, fruit
2366	A-00-09651	00-09651		Low fat yogurt, muesli or nut	06.03.02	Dairy products - Yoghurt & drinking yoghurts, incl. buttermilk and probiotics - reduced or low fat products	11435100	Yogurt, Greek, with oats
2367	A-12-0191			Low fat yogurt, muesli/nut	06.03.02	Dairy products - Yoghurt & drinking yoghurts, incl. buttermilk and probiotics - reduced or low fat products	11435100	Yogurt, Greek, with oats
2368	00-09652	A-00-09652		Low fat yogurt, non-fruit (toffee)	06.03.02	Dairy products - Yoghurt & drinking yoghurts, incl. buttermilk and probiotics - reduced or low fat products	11400000	Yogurt, NFS
2369	A-12-0188	00-09648	A-00-09648	Low fat yogurt, plain	06.03.02	Dairy products - Yoghurt & drinking yoghurts, incl. buttermilk and probiotics - reduced or low fat products	11400000	Yogurt, NFS
2370	A-12-0259			Low-fat spread	08.04.03	Fats - Plant based fats (solid) - Low fat	81102000	Margarine, NFS

2371	17-0180	A-00-00883	A-17-0180	Lucozade	27.03.00	Beverages - Carbonated soft drinks	95310560	Energy drink (NOS)
2372	00-05648			Lucozade Sport any flavour	27.03.00	Beverages - Carbonated soft drinks	95310560	Energy drink (NOS)
2373	A-00-06210			Luncheon meat 50	12.03.00	Processed meat - Other processed meats	25230110	Luncheon meat, NFS
2374	19-0135	A-19-0135	A-00-00396	Luncheon meat, canned	10.03.00	Meat - red - Pork & dishes	25230110	Luncheon meat, NFS
2375	A-00-00757			Lychees, Canned	18.02.00	Fruit - Canned & cooked	63126600	Lychee, cooked or canned, in sugar or syrup
2376	A-00-00756			Lychees, Raw	18.01.00	Fruit - Fresh	63126510	Lychee, raw
2377	14-0144	A-14-0144		Lychees, canned in syrup	18.02.00	Fruit - Canned & cooked	63126600	Lychee, cooked or canned, in sugar or syrup
2378	A-14-0142	14-0142		Lychees, raw	18.01.00	Fruit - Fresh	63126510	Lychee, raw
2379	02-07961			M & MS CHOCOLATE CENTRE NOT PEANUT SMARTIES MINSTRELS	24.01.00	Confectionary - Chocolate based products	91746100	M&M's Milk Chocolate Candies
2380	02-08049			MACARONI CHEESE PURCHASED READY MEAL	01.02.00	Cereals & cereal dishes - Pasta & pasta dishes	58145110	Macaroni or noodles with cheese
2381	02-00348			MACAROONS OR AMARETTI	04.01.00	Sweet cereal products - Biscuits	53202000	Cookie, almond
2382	02-10305			MALT WHEAT CEREAL, SHREDDIES TYPE, FORTIFIED, OWN BRAND	02.02.00	Breakfast cereals - Other breakfast cereals - high fibre (equal or >3g/40g portion)	57100100	Cereal, ready-to-eat, NFS
2383	02-08460			MALTED FLAKE BREAKFAST CEREAL OWN BRAND, FORTIFIED	02.03.00	Breakfast cereals - Other breakfast cereals - low fibre	57100100	Cereal, ready-to-eat, NFS
2384	02-02275			MALTESERS	24.01.00	Confectionary - Chocolate based products	91705020	Milk chocolate candy, with cereal
2385	02-10441			MARMITE FLAVOUR RICE CAKES	25.04.00	Savoury Snacks - Savoury biscuits & crackers	54318500	Rice cake
2386	02-02394			MARTINI; CINZANO; CAMPARI; RICCADONNA DRY EXTRA DR	27.01.02	Beverages - Alcohol - Fortified wine	93402000	Wine, dessert, sweet

2387	02-10410			MASHED POTATO PURCHASED MADE WITH WHOLE MILK AND BUTTER	17.01.00	Potatoes - Potatoes	71501010	Potato, mashed, from fresh, made with milk
2388	02-04202			MCDONALD'S MCFLURRY - ICECREAM WITH ADDED CHOCOLATE TOPPINGS	06.04.02	Dairy products - Ice cream & dairy desserts - reduced fat products	13140670	Light ice cream, sundae, soft serve, fruit topping, without whipped cream
2389	02-01332			MEAT CHOP SUEY PORK BEEF LAMB	10.04.00	Meat - red - Other red meat, e.g. rabbit, venison	27460010	Chow mein or chop suey, NS as to type of meat, no noodles
2390	02-10531			MEXICAN SPICE MIXES	26.01.00	Miscellaneous - Dried herbs & spices & pastes	2009	Spices, chili powder
2391	02-07958			MILK CHOCOLATE RAISINS	24.01.00	Confectionary - Chocolate based products	91739010	Raisins, chocolate covered
2392	02-00699			MILK SEMI SKIMMED AFTER BOILING	05.02.00	Milk - Semi-skimmed milk	11100000	Milk, NFS
2393	02-00700			MILK SKIMMED AFTER BOILING	05.02.00	Milk - Semi-skimmed milk	11100000	Milk, NFS
2394	02-03259			MILLET BOILED	01.04.00	Cereals & cereal dishes - Other cereals & dishes	20032	Millet, cooked
2395	02-08381			MINERAL WATER CARBONATED FLAVOURED NO ADDED SUGAR	27.04.00	Beverages - Water (still, tap, sparkling, flavoured)	94000100	Water, tap
2396	02-05253			MINERAL WATER STILL OR CARB WITH ARTI SWEETENER	27.04.00	Beverages - Water (still, tap, sparkling, flavoured)	94000100	Water, tap
2397	02-08155			MINI CRISPBREAD SNACKS FLAVOURED EG. RYVITA MINIS	03.05.00	Breads - Crisp Breads, e.g. Rivetas, Grissini, Toast Melba	54305010	Crackers, crispbread
2398	02-09232			MINT (FRESH)	15.04.00	Vegetables - Other	75109400	Basil, raw
2399	02-08307			MINT IMPERIALS	24.02.00	Confectionary - Sugar based products	91700010	Candy, NFS

2400	02-02436			MINT JELLY	21.03.00	Sauces & accompaniment - Other sauces, incl. brown sauce, soy sauce, ketchup, mint sauce, vinegar	91501010	Gelatin dessert
2401	02-07983			MINTOES	24.02.00	Confectionary - Sugar based products	91700010	Candy, NFS
2402	02-02358			MIXED FRUIT JUICE DRINK RTD NOT LOW CALORIE	27.02.02	Beverages - Fruit based drinks - Fruit juice drinks	92531030	Fruit juice drink (Sunny D)
2403	02-02357			MIXED FRUIT JUICE PASTEURISED	27.02.02	Beverages - Fruit based drinks - Fruit juice drinks	92531030	Fruit juice drink (Sunny D)
2404	02-08084			MIXED LEAF SALAD	15.03.00	Vegetables - Yellow & red & dark green leafy vegetables	75114000	Mixed salad greens, raw
2405	02-02188			MIXED NUTS UNROASTED UNSALTED	19.00.00	Nuts & Seeds (incl. peanut butter)	42110000	Mixed nuts, NFS
2406	02-08298			MIXED SEEDS	19.00.00	Nuts & Seeds (incl. peanut butter)	43107000	Mixed seeds
2407	02-08059			MOCHA (NO CREAM) ESPRESSO AND WHOLE MILK TAKEAWAY ONLY	27.06.00	Beverages - Coffee	92101950	Coffee, Cafe Mocha
2408	02-08061			MOCHA (WITH CREAM) ESPRESSO AND WHOLE MILK TAKEAWAY ONLY	27.06.00	Beverages - Coffee	92101950	Coffee, Cafe Mocha
2409	02-10322			MORRISONS TRIM FLAKES BREAKFAST CEREAL	02.03.00	Breakfast cereals - Other breakfast cereals - low fibre	57100100	Cereal, ready-to-eat, NFS
2410	02-08118			MUESLI WITH 55% FRUIT	02.01.00	Breakfast cereals - Oat based cereals	57308190	Cereal, muesli
2411	02-07629			MUESLI WITH ADDED SUGAR, WITH EXTRA FRUIT AND NUTS E.G. SAINSBURY'S LUXURY MUESLI	02.02.00	Breakfast cereals - Other breakfast cereals - high fibre (equal or >3g/40g portion)	57308190	Cereal, muesli
2412	02-00173			MUFFINS ENGLISH WHOLEMEAL OR BRAN	04.02.00	Sweet cereal products - Pastries, Buns & Pies	51303030	Muffin, English, whole wheat

2413	02-10133			MULLER VITALITY PROBIOTIC DRINK WITH OMEGA 3	06.03.02	Dairy products - Yoghurt & drinking yoghurts, incl. buttermilk and probiotics - reduced or low fat products	11436000	Yogurt, liquid
2414	02-10123			MULTIGRAIN HOOPS BREAKFAST CEREAL SUPERMARKET BRAND	02.03.00	Breakfast cereals - Other breakfast cereals - low fibre	57100100	Cereal, ready-to-eat, NFS
2415	02-08020			MULTISEED BREAD WHITE ONLY	03.01.00	Breads - White	51601020	Bread, multigrain
2416	02-10203			MULTISEED BREAD WHITE ONLY TOASTED	03.01.00	Breads - White	51601010	Bread, multigrain, toasted
2417	02-08019			MULTISEED BREAD WHOLEMEAL ONLY	03.02.00	Breads - Wholemeal	51601020	Bread, multigrain
2418	02-10204			MULTISEED BREAD WHOLEMEAL ONLY TOASTED	03.02.00	Breads - Wholemeal	51601010	Bread, multigrain, toasted
2419	02-06847			MUSHROOM IN BREADCRUMB AND BATTER COATING,FRIED IN OIL	15.04.00	Vegetables - Other	75414030	Mushrooms, batter-dipped, fried
2420	02-07926			MUSHROOM SOUP, PREMIUM, CHILLED, CARTON	20.01.00	Soups - Canned & fresh & homemade	75607000	Mushroom soup, NFS
2421	02-01780			MUSHROOMS CHINESE DRIED	15.04.00	Vegetables - Other	11268	Mushrooms, shiitake, dried
2422	14-0823			Macadamia nuts, salted	19.00.00	Nuts & Seeds (incl. peanut butter)	42109100	Macadamia nuts
2423	A-00-00177			Macaroni Cheese	01.02.00	Cereals & cereal dishes - Pasta & pasta dishes	58145110	Macaroni or noodles with cheese
2424	12-0275	A-12-0275		Macaroni cheese	01.02.00	Cereals & cereal dishes - Pasta & pasta dishes	58145110	Macaroni or noodles with cheese
2425	A-12-0276	12-0276		Macaroni cheese, canned	01.02.00	Cereals & cereal dishes - Pasta & pasta dishes	58145110	Macaroni or noodles with cheese
2426	A-00-00016			Macaroni, Boiled	01.02.00	Cereals & cereal dishes - Pasta & pasta dishes	56130000	Pasta, cooked

2427	A-00-00015			Macaroni, Raw	01.02.00	Cereals & cereal dishes - Pasta & pasta dishes	20120	Pasta, dry, enriched
2428	A-11-0054	11-0054		Macaroni, boiled	01.02.00	Cereals & cereal dishes - Pasta & pasta dishes	56130000	Pasta, cooked
2429	A-11-0341			Macaroni, canned in cheese sauce	01.02.00	Cereals & cereal dishes - Pasta & pasta dishes	58145110	Macaroni or noodles with cheese
2430	A-12-0828	12-0828		Macaroon	04.01.00	Sweet cereal products - Biscuits	53225000	Cookie, macaroon
2431	00-05400			Mackerel Fillets in Sunflower Oil eg Tesco	09.02.00	Fish & fish dishes - Oily fish	26121180	Mackerel, canned
2432	16-0297	A-16-0297		Mackerel pate, smoked	09.02.00	Fish & fish dishes - Oily fish	26121190	Mackerel, smoked
2433	A-00-00492			Mackerel, Fried	09.02.00	Fish & fish dishes - Oily fish	26121120	Mackerel, baked or broiled, fat added in cooking
2434	A-00-00493			Mackerel, Fried (Weighed with Bones)	09.02.00	Fish & fish dishes - Oily fish	26121120	Mackerel, baked or broiled, fat added in cooking
2435	A-00-00491			Mackerel, Raw	09.02.00	Fish & fish dishes - Oily fish	26121100	Mackerel, raw
2436	16-0197	A-16-0197		Mackerel, canned in brine, drained	09.02.00	Fish & fish dishes - Oily fish	26121180	Mackerel, canned
2437	A-16-0198	16-0198		Mackerel, canned in tomato sauce	09.02.00	Fish & fish dishes - Oily fish	27150350	Sardines with tomato-based sauce
2438	A-16-0192			Mackerel, fried in blended oil	09.02.00	Fish & fish dishes - Oily fish	26121120	Mackerel, baked or broiled, fat added in cooking
2439	16-0194			Mackerel, grilled	09.02.00	Fish & fish dishes - Oily fish	26121121	Mackerel, baked or broiled, fat not added in cooking
2440	A-00-09968			Mackerel, grilled (MW6 vit D)	09.02.00	Fish & fish dishes - Oily fish	26121121	Mackerel, baked or broiled, fat not added in cooking
2441	A-16-0196	16-0196		Mackerel, smoked	09.02.00	Fish & fish dishes - Oily fish	26121190	Mackerel, smoked
2442	A-00-03816			Macvita 82/1905	03.05.00	Breads - Crisp Breads, e.g. Rivetas, Grissini, Toast Melba	54305010	Crackers, crispbread

2443	A-00-00079			Madeira Cake	04.02.00	Sweet cereal products - Pastries, Buns & Pies	53116000	Cake, pound, without icing or filling
2444	11-0209	A-11-0209		Madeira cake	04.02.00	Sweet cereal products - Pastries, Buns & Pies	53116000	Cake, pound, without icing or filling
2445	A-00-09955			Madeira cake (MW6 Vit Eq)	04.02.00	Sweet cereal products - Pastries, Buns & Pies	53116000	Cake, pound, without icing or filling
2446	A-00-06061			Madeira cake 50	04.02.00	Sweet cereal products - Pastries, Buns & Pies	53116000	Cake, pound, without icing or filling
2447	A-00-00198			Maize (Corn) Oil	08.02.00	Fats - Oils	82102000	Corn oil
2448	17-0127	A-17-0127		Maize and rice flour snacks Frazzles and Bacon Streaks	25.02.00	Savoury Snacks - Cereal based snacks	54420210	Multigrain chips (Sun Chips)
2449	A-00-09580	00-09580		Malt Bread	03.04.00	Breads - Other bread	52405010	Bread, fruit
2450	A-11-0084			Malt bread	03.04.00	Breads - Other bread	52405010	Bread, fruit
2451	A-00-06011			Malt bread 50	03.04.00	Breads - Other bread	52405010	Bread, fruit
2452	A-00-00758			Mandarin Oranges, Canned	18.02.00	Fruit - Canned & cooked	61122300	Orange, mandarin, canned or frozen, NS as to sweetened or unsweetened; sweetened, NS as to type of sweetener
2453	14-0146	A-14-0146		Mandarin oranges, canned in juice	18.02.00	Fruit - Canned & cooked	61122320	Orange, mandarin, canned or frozen, juice pack
2454	14-0147	A-14-0147		Mandarin oranges, canned in syrup	18.02.00	Fruit - Canned & cooked	61122300	Orange, mandarin, canned or frozen, NS as to sweetened or unsweetened; sweetened, NS as to type of sweetener
2455	00-03614	A-00-03614		Mange-tout peas, boiled, unsalted	15.04.00	Vegetables - Other	75231000	Snowpea, cooked, NS as to form, NS as to fat added in cooking
2456	13-0122	A-13-0122		Mange-tout peas, raw	15.04.00	Vegetables - Other	75127750	Snowpeas, raw

2457	A-13-0124			Mange-tout peas, stir-fried in blended oil	15.04.00	Vegetables - Other	75231000	Snowpea, cooked, NS as to form, NS as to fat added in cooking
2458	A-00-01246			Mango Juice, Canned	27.02.01	Beverages - Fruit based drinks - Pure fruit juice & smoothies	61210000	Orange juice, 100%, NFS
2459	14-0280	A-14-0280		Mango juice, canned	27.02.01	Beverages - Fruit based drinks - Pure fruit juice & smoothies	61210000	Orange juice, 100%, NFS
2460	A-00-00760			Mangoes, Canned	18.02.00	Fruit - Canned & cooked	63129030	Mango, cooked
2461	A-00-00759			Mangoes, Raw	18.01.00	Fruit - Fresh	63129010	Mango, raw
2462	14-0150	A-14-0150		Mangoes, ripe, canned in syrup	18.02.00	Fruit - Canned & cooked	63129030	Mango, cooked
2463	14-0148			Mangoes, ripe, raw	18.01.00	Fruit - Fresh	63129010	Mango, raw
2464	A-00-09925			Mangoes, ripe, raw (MW6 carq)	18.01.00	Fruit - Fresh	63129010	Mango, raw
2465	A-00-00188			Margarine, Hard, Animal and Vegetable Oils	08.03.00	Fats - Animal based fats (solid)	81201000	Animal fat or drippings
2466	A-00-00189			Margarine, Hard, Vegetable Oils Only	08.04.01	Fats - Plant based fats (solid) - Full fat	81102000	Margarine, NFS
2467	A-00-03443	00-03443		Margarine, Hard. Animal and Vegetable. EG Stork from a packet	08.04.01	Fats - Plant based fats (solid) - Full fat	81102000	Margarine, NFS
2468	A-00-00186			Margarine, Low Fat (Low Fat Spread)	08.04.03	Fats - Plant based fats (solid) - Low fat	81102000	Margarine, NFS
2469	A-00-00192			Margarine, Polyunsaturated	08.04.01	Fats - Plant based fats (solid) - Full fat	81102000	Margarine, NFS
2470	A-00-00190			Margarine, Soft, Animal and Vegetable Oils	08.04.01	Fats - Plant based fats (solid) - Full fat	81102000	Margarine, NFS
2471	A-00-00191			Margarine, Soft, Vegetable Oils Only	08.04.01	Fats - Plant based fats (solid) - Full fat	81102000	Margarine, NFS
2472	A-00-03442	00-03442		Margarine, hard, Animal and Vegetable. EG ECHO	08.04.01	Fats - Plant based fats (solid) - Full fat	81102000	Margarine, NFS
2473	17-0019			Margarine, hard, vegetable fats only, Tomor	08.04.01	Fats - Plant based fats (solid) - Full fat	81102000	Margarine, NFS

2474	A-00-03444	00-03444		Margarine, soft, Animal and Vegetable. EG Stork from a tub	08.04.01	Fats - Plant based fats (solid) - Full fat	81102000	Margarine, NFS
2475	00-05512			Marigold Swiss Vegetable Bouillon Powder	26.01.00	Miscellaneous - Dried herbs & spices & pastes	6981	Soup, bouillon cubes and granules, low sodium, dry
2476	00-05796			Marigold Swiss Vegetable Bouillon Powder Reduced Salt	26.01.00	Miscellaneous - Dried herbs & spices & pastes	6981	Soup, bouillon cubes and granules, low sodium, dry
2477	13-0835			Marjoram, dried	26.01.00	Miscellaneous - Dried herbs & spices & pastes	2023	Spices, marjoram, dried
2478	A-17-0078	A-00-00853	17-0078	Marmalade	22.01.00	Preserves - Jam & Marmalade	91404000	Marmalade, all flavors
2479	A-17-0079	17-0079		Marmalade, diabetic	22.01.00	Preserves - Jam & Marmalade	91406600	Jam, preserve, marmalade, reduced sugar, all flavors
2480	00-03923	A-00-03923		Marmalade, reduced sugar	22.01.00	Preserves - Jam & Marmalade	91406600	Jam, preserve, marmalade, reduced sugar, all flavors
2481	A-00-00961			Marmite	26.01.00	Miscellaneous - Dried herbs & spices & pastes	43406	Yeast extract spread
2482	00-05926			Marmite Cereal Bar ONLY	04.05.00	Sweet cereal products - Cereal bars	53712100	Cereal or Granola bar, NFS
2483	00-05643			Marmite breadsticks	25.04.00	Savoury Snacks - Savoury biscuits & crackers	51184000	Breadsticks, hard, NFS
2484	A-00-00608			Marrow, Boiled	15.04.00	Vegetables - Other	75233000	Squash, summer, yellow or green, cooked, NS as to form, NS as to fat added in cooking
2485	A-00-00607			Marrow, Raw	15.04.00	Vegetables - Other	75128010	Squash, summer, green, raw
2486	13-0276	A-13-0276		Marrow, boiled in unsalted water	15.04.00	Vegetables - Other	75233000	Squash, summer, yellow or green, cooked, NS as to form, NS as to fat added in cooking

2487	A-13-0274			Marrow, raw	15.04.00	Vegetables - Other	75128010	Squash, summer, green, raw
2488	13-0125			Marrowfat peas, canned, re-heated, drained	15.04.00	Vegetables - Other	75224013	Peas, green, cooked, from canned, NS as to fat added in cooking
2489	A-17-0094	17-0094		Mars bar	24.01.00	Confectionary - Chocolate based products	91726130	MILKY WAY Bar
2490	A-17-0114	17-0114		Marshmallows	24.02.00	Confectionary - Sugar based products	91723000	Marshmallow
2491	A-00-00854			Marzipan, Almond Paste	19.00.00	Nuts & Seeds (incl. peanut butter)	42200600	Almond paste
2492	A-14-0824	14-0824		Marzipan, homemade	19.00.00	Nuts & Seeds (incl. peanut butter)	42200600	Almond paste
2493	14-0825	A-14-0825		Marzipan, retail	19.00.00	Nuts & Seeds (incl. peanut butter)	42200600	Almond paste
2494	00-03148	A-00-03148		Matzo Crackers	25.04.00	Savoury Snacks - Savoury biscuits & crackers	54307000	Crackers, matzo
2495	A-11-0178			Matzos	03.05.00	Breads - Crisp Breads, e.g. Rivetas, Grissini, Toast Melba	54307000	Crackers, matzo
2496	17-0316	A-12-0277	A-00-00926	Mayonnaise	21.01.00	Sauces & accompaniment - Dressings & Mayonnaise	83107000	Mayonnaise, regular
2497	A-00-09964			Mayonnaise (MW6 Vit Eq)	21.01.00	Sauces & accompaniment - Dressings & Mayonnaise	83107000	Mayonnaise, regular
2498	A-17-0317	17-0317		Mayonnaise, homemade	21.01.00	Sauces & accompaniment - Dressings & Mayonnaise	83107000	Mayonnaise, regular
2499	17-0318	A-17-0318		Mayonnaise, reduced calorie	21.01.00	Sauces & accompaniment - Dressings & Mayonnaise	83204000	Mayonnaise, light
2500	A-12-0278			Mayonnaise, retail	21.01.00	Sauces & accompaniment - Dressings & Mayonnaise	83107000	Mayonnaise, regular
2501	A-12-0279			Mayonnaise, retail, reduced calorie	21.01.00	Sauces & accompaniment - Dressings & Mayonnaise	83204000	Mayonnaise, light
2502	00-05505			McVities Krackawheat	25.04.00	Savoury Snacks - Savoury biscuits & crackers	54326000	Crackers, multigrain

2503	A-00-01097			Meat Loaf	10.01.00	Meat - red - Beef & veal & dishes	27214100	Meat loaf made with beef
2504	A-00-00418			Meat Paste	12.03.00	Processed meat - Other processed meats	25240000	Meat spread or potted meat, NFS
2505	A-00-03812			Meat Pie, 1 crust 82/1455	12.02.00	Processed meat - Processed pies	27360050	Meat pie, NFS
2506	A-00-01095			Meat Pie, 2 Crusts, Short Pastry/ Plate Pie made with Mince	12.02.00	Processed meat - Processed pies	27360050	Meat pie, NFS
2507	A-00-01093			Meat Pie, Individual	12.02.00	Processed meat - Processed pies	27360050	Meat pie, NFS
2508	00-09516	A-00-09516		Meat Spring Roll	11.01.00	Meat - white - Chicken & turkey & dishes	58110170	Egg roll, with chicken or turkey
2509	A-00-06204			Meat composite 50	10.01.00	Meat - red - Beef & veal & dishes	13796	Beef, composite of trimmed retail cuts, separable lean and fat, trimmed to 1/8" fat, all grades, cooked
2510	17-0361			Meat extract	21.03.00	Sauces & accompaniment - Other sauces, incl. brown sauce, soy sauce, ketchup, mint sauce, vinegar	28310110	Beef, broth, bouillon, or consomme
2511	A-00-09943			Meat extract (MW6 folate)	21.03.00	Sauces & accompaniment - Other sauces, incl. brown sauce, soy sauce, ketchup, mint sauce, vinegar	28310110	Beef, broth, bouillon, or consomme
2512	19-0138	A-19-0138		Meat loaf, chilled/frozen, reheated	10.04.00	Meat - red - Other red meat, e.g. rabbit, venison	27260010	Meat loaf, NS as to type of meat
2513	A-19-0137			Meat loaf, homemade	10.04.00	Meat - red - Other red meat, e.g. rabbit, venison	27260010	Meat loaf, NS as to type of meat
2514	A-00-06211			Meat paste 50	12.03.00	Processed meat - Other processed meats	25240000	Meat spread or potted meat, NFS
2515	A-00-06217			Meat pie, 2 crusts, Short pastry 50	12.02.00	Processed meat - Processed pies	27360050	Meat pie, NFS

2516	A-19-0139	19-0139		Meat spread	12.03.00	Processed meat - Other processed meats	25240000	Meat spread or potted meat, NFS
2517	A-00-01175			Meat with Vegetables and/or Nuts	10.01.00	Meat - red - Beef & veal & dishes	27415170	Kung Pao beef
2518	A-00-01094			Meat/ Steak Pie, 1 Crust, Flaky Pastry	12.02.00	Processed meat - Processed pies	27360050	Meat pie, NFS
2519	A-00-06219			Meat/Steak Pie, 1 Crust, Flaky pastry 50	12.02.00	Processed meat - Processed pies	27360050	Meat pie, NFS
2520	A-12-0169			Medium fat soft cheese	06.02.00	Dairy products - Cheese, incl. cottage cheese	14303010	Cheese, cream, light or lite
2521	00-09724	A-00-09724		Medium fat soft cheese (Philadelphia light)	06.02.00	Dairy products - Cheese, incl. cottage cheese	14303010	Cheese, cream, light or lite
2522	A-00-00761			Medlars, Raw	18.01.00	Fruit - Fresh	63101000	Apple, raw
2523	A-00-01231			Melon Seeds, Kernels Only	19.00.00	Nuts & Seeds (incl. peanut butter)	43101050	Pumpkin seeds, NFS
2524	14-0156			Melon, Canteloupe-type	18.01.00	Fruit - Fresh	63109010	Cantaloupe, raw
2525	A-00-09926			Melon, Canteloupe-type (MW6 carq)	18.01.00	Fruit - Fresh	63109010	Cantaloupe, raw
2526	14-0159	A-14-0159		Melon, Galia	18.01.00	Fruit - Fresh	63127010	Honeydew melon, raw
2527	14-0162	A-14-0162		Melon, Honeydew	18.01.00	Fruit - Fresh	63127010	Honeydew melon, raw
2528	14-0153	A-14-0153		Melon, average	18.01.00	Fruit - Fresh	63109010	Cantaloupe, raw
2529	A-00-09927			Melon, watermelon (MW6 carq)	18.01.00	Fruit - Fresh	63149010	Watermelon, raw
2530	14-0165			Melon,water	18.01.00	Fruit - Fresh	63149010	Watermelon, raw
2531	A-00-00762			Melons, Canteloupe, Raw	18.01.00	Fruit - Fresh	63109010	Cantaloupe, raw
2532	A-00-00766			Melons, Watermelon, Raw	18.01.00	Fruit - Fresh	63149010	Watermelon, raw
2533	A-00-00767			Melons, Watermelon, Raw (Weighed with Skin)	18.01.00	Fruit - Fresh	63149010	Watermelon, raw
2534	A-00-00764			Melons, Yellow, Honeydew, Raw	18.01.00	Fruit - Fresh	63127010	Honeydew melon, raw
2535	A-00-00765			Melons, Yellow, Raw (Weighed with Skin)	18.01.00	Fruit - Fresh	63127010	Honeydew melon, raw
2536	A-11-0179			Melting moments	04.01.00	Sweet cereal products - Biscuits	53241500	Cookie, butter or sugar
2537	12-0829	A-12-0829		Meringue	07.00.00	Egg & egg dishes	32401000	Meringues

2538	A-00-01041			Meringue, Cream Filling	07.00.00	Egg & egg dishes	32401000	Meringues
2539	12-0830	A-12-0830		Meringue, with cream	07.00.00	Egg & egg dishes	32401000	Meringues
2540	A-00-00113			Meringues	07.00.00	Egg & egg dishes	32401000	Meringues
2541	A-00-01318			Methi Baji with Brains	14.02.00	Offal - Other offal & dishes, e.g. Haggis, faggots	25150000	Brains, cooked
2542	A-12-0102			Microdiet powder, made up with water	30.00.00	Nutrition Powders & drinks	95120010	Nutritional drink or shake, high protein, ready-to-drink, NFS
2543	13-0028	A-13-0028		Microwave chips, cooked	17.02.00	Potatoes - Potato products - other	71400990	Potato, french fries, NFS
2544	A-00-03932	00-03932		Mignons morceaux, crispy garlic bread snack	03.04.00	Breads - Other bread	54402700	Pita chips
2545	17-0215	A-17-0215		Mild, draught	27.01.03	Beverages - Alcohol - Beer	93101000	Beer
2546	00-01137	A-00-01137		Milk Chocolate with Fruit and Nuts	24.01.00	Confectionary - Chocolate based products	91705050	Milk chocolate candy, with fruit and nuts
2547	00-03388			Milk Goats Semi-skimmed	05.05.00	Milk - Other - animal based, e.g. goat	11100000	Milk, NFS
2548	A-00-00114			Milk Pudding	04.04.00	Sweet cereal products - Milk based puddings	13230110	Pudding, ready-to-eat, flavors other than chocolate
2549	A-00-00115			Milk Pudding, Canned, Rice	04.04.00	Sweet cereal products - Milk based puddings	13210410	Pudding, rice
2550	00-03860			Milk Shake Syrup e.g. Crusha	23.02.00	Sugars - Other, incl. syrups, honey	91301080	Chocolate syrup, thin type
2551	A-00-09756	00-09756		Milk Shake, purchased, thick, any flavour	27.05.00	Beverages - Powdered Beverages (cocoa, Horlicks, Bonvita, Ovaltine, etc)	11542100	Milk shake, fast food, chocolate
2552	A-00-09582	A-11-0085	00-09582	Milk bread	03.04.00	Breads - Other bread	51101050	Bread, white, made from home recipe or purchased at a bakery
2553	A-00-09662			Milk chocolate trifle	06.04.02	Dairy products - Ice cream & dairy desserts - reduced fat products	13252600	Tiramisu

2554	00-09662			Milk chocolate trifle, purchased	06.04.02	Dairy products - Ice cream & dairy desserts - reduced fat products	13252600	Tiramisu
2555	00-09825	A-00-09825		Milk pudding, made semi-skimmed milk	04.04.00	Sweet cereal products - Milk based puddings	13230110	Pudding, ready-to-eat, flavors other than chocolate
2556	A-12-0242			Milk pudding, made with semi-skimmed milk	04.04.00	Sweet cereal products - Milk based puddings	13230110	Pudding, ready-to-eat, flavors other than chocolate
2557	A-12-0243			Milk pudding, made with skimmed milk	04.04.00	Sweet cereal products - Milk based puddings	13230110	Pudding, ready-to-eat, flavors other than chocolate
2558	A-00-09824	00-09824	A-12-0241	Milk pudding, made with whole milk	04.04.00	Sweet cereal products - Milk based puddings	13230110	Pudding, ready-to-eat, flavors other than chocolate
2559	00-09826	A-00-09826		Milk pudding,made with skimmed milk	04.04.00	Sweet cereal products - Milk based puddings	13230110	Pudding, ready-to-eat, flavors other than chocolate
2560	A-12-0104			Milk shake powder	27.05.00	Beverages - Powdered Beverages (cocoa, Horlicks, Bonvita, Ovaltine, etc)	11830150	Cocoa powder, not reconstituted
2561	A-12-0106			Milk shake powder, made up with semi-skimmed milk	27.05.00	Beverages - Powdered Beverages (cocoa, Horlicks, Bonvita, Ovaltine, etc)	11513380	Chocolate milk, made from dry mix, NS as to type of milk (Nesquik)
2562	A-12-0107			Milk shake powder, made up with skimmed milk	27.05.00	Beverages - Powdered Beverages (cocoa, Horlicks, Bonvita, Ovaltine, etc)	11513380	Chocolate milk, made from dry mix, NS as to type of milk (Nesquik)
2563	A-12-0105			Milk shake powder, made up with whole milk	27.05.00	Beverages - Powdered Beverages (cocoa, Horlicks, Bonvita, Ovaltine, etc)	11513380	Chocolate milk, made from dry mix, NS as to type of milk (Nesquik)
2564	A-12-0103	12-0103		Milk shake, purchased	27.05.00	Beverages - Powdered Beverages (cocoa, Horlicks, Bonvita, Ovaltine, etc)	11542100	Milk shake, fast food, chocolate

2565	00-09810	A-00-09810		Milk shake, with semi-skimmed milk	05.06.00	Milk - Milk based drinks, e.g. flavoured milks	11541120	Milk shake, home recipe, flavors other than chocolate
2566	00-09811	A-00-09811		Milk shake, with skimmed milk	05.06.00	Milk - Milk based drinks, e.g. flavoured milks	11541120	Milk shake, home recipe, flavors other than chocolate
2567	A-00-09809			Milk shake, with whole milk	05.06.00	Milk - Milk based drinks, e.g. flavoured milks	11541120	Milk shake, home recipe, flavors other than chocolate
2568	A-00-00132			Milk, Condensed, Whole, Sweetened	05.03.00	Milk - Whole milk	11220000	Milk, condensed, sweetened
2569	A-00-00124			Milk, Cows', Fresh, Whole Summer	05.03.00	Milk - Whole milk	11100000	Milk, NFS
2570	A-00-00125			Milk, Cows', Fresh, Whole Winter.	05.03.00	Milk - Whole milk	11100000	Milk, NFS
2571	A-00-00127			Milk, Cows', Fresh, Whole, Channel Isles, Summer	05.03.00	Milk - Whole milk	11100000	Milk, NFS
2572	A-00-00128			Milk, Cows', Fresh, Whole, Channel Isles, Winter	05.03.00	Milk - Whole milk	11100000	Milk, NFS
2573	A-00-00136			Milk, Dried, Skimmed	05.01.00	Milk - Skimmed milk	11810000	Milk, dry, not reconstituted, NS as to fat content
2574	A-00-00135			Milk, Dried, Whole	05.03.00	Milk - Whole milk	11810000	Milk, dry, not reconstituted, NS as to fat content
2575	A-00-00134			Milk, Evaporated, Whole, Unsweetened	05.03.00	Milk - Whole milk	11210050	Milk, evaporated, NS as to fat content
2576	A-00-00131			Milk, Fresh, Skimmed	05.01.00	Milk - Skimmed milk	11100000	Milk, NFS
2577	A-00-00137			Milk, Goats'	05.05.00	Milk - Other - animal based, e.g. goat	11100000	Milk, NFS
2578	A-00-00130			Milk, Longlife (UHT Treated)	05.03.00	Milk - Whole milk	11100000	Milk, NFS
2579	A-00-00129			Milk, Sterilized	05.03.00	Milk - Whole milk	11100000	Milk, NFS

2580	A-00-01380			Milk, dried, average filled and skimmed	05.01.00	Milk - Skimmed milk	11810000	Milk, dry, not reconstituted, NS as to fat content
2581	A-00-01379			Milk, fresh average	05.02.00	Milk - Semi-skimmed milk	11100000	Milk, NFS
2582	00-05689			Milkshake, thick style, takeaway	05.06.00	Milk - Milk based drinks, e.g. flavoured milks	11542200	Milk shake, fast food, flavors other than chocolate
2583	17-0095	A-17-0095		Milky Way	24.01.00	Confectionary - Chocolate based products	91726420	3 MUSKETEERS Bar
2584	A-00-03765			Mince Pancakes (B.E.) fried 82/1404	10.01.00	Meat - red - Beef & veal & dishes	58120120	Crepe, filled with meat, poultry, or seafood, no sauce
2585	A-00-00088			Mince Pies	04.02.00	Sweet cereal products - Pastries, Buns & Pies	53306000	Pie, mince, two crust
2586	A-11-0258	11-0258		Mince pies, individual	04.02.00	Sweet cereal products - Pastries, Buns & Pies	53306070	Pie, mince, individual size or tart
2587	A-00-06037			Mince pies, individual 50	04.02.00	Sweet cereal products - Pastries, Buns & Pies	53306070	Pie, mince, individual size or tart
2588	19-0244	A-19-0244		Minced beef with gravy with/without onions, canned	10.01.00	Meat - red - Beef & veal & dishes	27112000	Beef with gravy
2589	A-19-0243	19-0243		Minced beef, extra lean, stewed	10.01.00	Meat - red - Beef & veal & dishes	23563	Beef, ground, 90% lean meat / 10% fat, patty, cooked, broiled
2590	A-00-00855	17-0080		Mincemeat	22.01.00	Preserves - Jam & Marmalade	53306000	Pie, mince, two crust
2591	A-00-01042			Mincemeat Tart, Large, One Crust	04.02.00	Sweet cereal products - Pastries, Buns & Pies	53306000	Pie, mince, two crust
2592	A-11-0259	11-0259		Mincemeat tart, one crust	04.02.00	Sweet cereal products - Pastries, Buns & Pies	53306000	Pie, mince, two crust
2593	A-00-06038			Mincemeat tart, one crust 50	04.02.00	Sweet cereal products - Pastries, Buns & Pies	53306000	Pie, mince, two crust
2594	A-00-01837	00-01837		Mineral water, high Ca (Aquaid)	27.04.00	Beverages - Water (still, tap, sparkling, flavoured)	94000100	Water, tap

2595	A-00-01835	00-01835		Mineral water, low Ca (Evian, Vitel)	27.04.00	Beverages - Water (still, tap, sparkling, flavoured)	94000100	Water, tap
2596	00-01836	A-00-01836		Mineral water, medium Ca (464)	27.04.00	Beverages - Water (still, tap, sparkling, flavoured)	94000100	Water, tap
2597	00-01838	A-00-01838		Mineral water, unspecified (as medium Ca)	27.04.00	Beverages - Water (still, tap, sparkling, flavoured)	94000100	Water, tap
2598	00-01839	A-00-01839		Mineral water, very low Ca (Highland)	27.04.00	Beverages - Water (still, tap, sparkling, flavoured)	94000100	Water, tap
2599	A-00-00945			Minestrone Soup, Dried, as Served	20.02.00	Soups - Dried	75651010	Minestrone soup, canned, prepared with water, or ready-to-serve
2600	17-0267			Minestrone soup	20.01.00	Soups - Canned & fresh & homemade	75651000	Minestrone soup, home recipe
2601	A-17-0266	17-0266		Minestrone soup, canned. Manufacturer's data (Heinz)	20.01.00	Soups - Canned & fresh & homemade	75651010	Minestrone soup, canned, prepared with water, or ready-to-serve
2602	17-0269	A-17-0269		Minestrone soup, dried, as served	20.02.00	Soups - Dried	75651010	Minestrone soup, canned, prepared with water, or ready-to-serve
2603	00-05432			Mini Milk Ice Cream (Walls)	06.04.01	Dairy products - Ice cream & dairy desserts - full fat products	13120050	Ice cream bar or stick, not chocolate covered or cake covered
2604	A-00-01221			Mint Leaves	15.04.00	Vegetables - Other	75109400	Basil, raw
2605	A-17-0319	17-0319		Mint sauce	21.03.00	Sauces & accompaniment - Other sauces, incl. brown sauce, soy sauce, ketchup, mint sauce, vinegar	75119000	Parsley, raw
2606	A-17-0320			Mint sauce, homemade	21.03.00	Sauces & accompaniment - Other sauces, incl. brown sauce, soy sauce, ketchup, mint sauce, vinegar	75119000	Parsley, raw
2607	13-0837			Mint, dried	26.01.00	Miscellaneous - Dried herbs & spices & pastes	2003	Spices, basil, dried
2608	13-0836	A-13-0836		Mint, fresh	15.04.00	Vegetables - Other	75109400	Basil, raw

2609	00-01128	A-00-01128		Mixed Dried Fruit, Stewed	18.02.00	Fruit - Canned & cooked	62113200	Fig, dried, cooked, NS as to sweetened or unsweetened; sweetened, NS as to type of sweetener
2610	A-00-01129			Mixed Dried Fruit, Stewed with Sugar	18.03.00	Fruit - Dried	62113230	Fig, dried, cooked, with sugar
2611	A-00-01227			Mixed Flavourings For Curry	26.01.00	Miscellaneous - Dried herbs & spices & pastes	2015	Spices, curry powder
2612	00-05867			Mixed fruit, stewed, without sugar	18.02.00	Fruit - Canned & cooked	63311120	Fruit cocktail, cooked or canned, unsweetened, water pack
2613	A-14-0827	14-0827		Mixed nuts	19.00.00	Nuts & Seeds (incl. peanut butter)	42110000	Mixed nuts, NFS
2614	A-14-0828	14-0828		Mixed nuts and raisins	19.00.00	Nuts & Seeds (incl. peanut butter)	42501000	Trail mix with nuts and fruit
2615	A-14-0167	14-0167		Mixed peel	18.03.00	Fruit - Dried	91708000	Fruit peel, candied
2616	A-17-0059	17-0059		Molasses	23.01.00	Sugars - Pure sugars	91303000	Molasses
2617	16-0093	A-16-0093		Monkfish, grilled	09.01.00	Fish & fish dishes - White fish, incl. tuna	26109123	Cod, baked or broiled, made without fat
2618	A-11-0122			Morning rolls	03.04.00	Breads - Other bread	51150000	Roll, white, soft
2619	19-0247	A-00-00433	A-19-0247	Moussaka	10.01.00	Meat - red - Beef & veal & dishes	77316600	Eggplant and meat casserole
2620	19-0248			Moussaka, chilled/frozen/longlife, reheated	10.01.00	Meat - red - Beef & veal & dishes	77316600	Eggplant and meat casserole
2621	12-0244	A-12-0244		Mousse, chocolate	06.04.02	Dairy products - Ice cream & dairy desserts - reduced fat products	13250000	Mousse, chocolate
2622	00-09663	A-00-09663		Mousse, chocolate, low-fat	06.04.02	Dairy products - Ice cream & dairy desserts - reduced fat products	13250200	Mousse, chocolate, lowfat, reduced calorie, prepared from dry mix, water added

2623	A-12-0245	12-0245		Mousse, chocolate, rich	06.04.01	Dairy products - Ice cream & dairy desserts - full fat products	13250000	Mousse, chocolate
2624	12-0247	A-12-0247		Mousse, frozen	06.04.01	Dairy products - Ice cream & dairy desserts - full fat products	13250000	Mousse, chocolate
2625	A-12-0246	12-0246		Mousse, fruit	06.04.01	Dairy products - Ice cream & dairy desserts - full fat products	13250100	Mousse, not chocolate
2626	A-00-03147	00-03147		Mr Kipling Slices	04.02.00	Sweet cereal products - Pastries, Buns & Pies	53118200	Cake, sponge, with icing or filling
2627	A-00-03146			Mr Kipling Strawberry Sundae's	04.02.00	Sweet cereal products - Pastries, Buns & Pies	53344200	Mixed fruit tart filled with custard or cream cheese
2628	A-00-00050			Muesli	02.01.00	Breakfast cereals - Oat based cereals	57308190	Cereal, muesli
2629	A-00-01390			Muesli bars, average	04.05.00	Sweet cereal products - Cereal bars	53710504	Cereal or granola bar (Kellogg's Nutri-Grain Fruit and Nut Bar)
2630	A-00-03032	00-03032		Muesli with added fruit, Weetabix, Alpen	02.01.00	Breakfast cereals - Oat based cereals	57308190	Cereal, muesli
2631	A-00-03029	00-03029		Muesli, No Added Sugar, Kelloggs	02.01.00	Breakfast cereals - Oat based cereals	57308190	Cereal, muesli
2632	00-03030	A-00-03030		Muesli, Swiss Style, Weetabix/Kelloggs	02.01.00	Breakfast cereals - Oat based cereals	57308190	Cereal, muesli
2633	A-11-0137			Muesli, Swiss style	02.01.00	Breakfast cereals - Oat based cereals	57308190	Cereal, muesli
2634	00-05881			Muesli, Swiss style, no added salt/sugar eg Sainsburys	02.01.00	Breakfast cereals - Oat based cereals	57308190	Cereal, muesli
2635	A-00-03803			Muesli, unsweetened 82/1446	02.01.00	Breakfast cereals - Oat based cereals	57308190	Cereal, muesli
2636	A-00-03031	00-03031		Muesli, unsweetned, with wheat, Own Brand	02.01.00	Breakfast cereals - Oat based cereals	57308190	Cereal, muesli
2637	A-11-0138			Muesli, with extra fruit	02.01.00	Breakfast cereals - Oat based cereals	57308190	Cereal, muesli

2638	11-0139	A-11-0139		Muesli, with no added sugar	02.01.00	Breakfast cereals - Oat based cereals	57308190	Cereal, muesli
2639	A-11-0260			Muffins	04.02.00	Sweet cereal products - Pastries, Buns & Pies	51186010	Muffin, English
2640	A-00-06039			Muffins 50	04.02.00	Sweet cereal products - Pastries, Buns & Pies	51186010	Muffin, English
2641	00-09607	A-00-09607		Muffins, American style	04.02.00	Sweet cereal products - Pastries, Buns & Pies	52301000	Muffin, NFS
2642	A-11-0261			Muffins, bran	04.02.00	Sweet cereal products - Pastries, Buns & Pies	52304150	Muffin, oat bran
2643	00-09588	A-00-09588		Muffins, plain (bread type)	03.01.00	Breads - White	51186010	Muffin, English
2644	14-0168			Mulberries, raw	18.01.00	Fruit - Fresh	63217010	Mulberries, raw
2645	A-17-0227			Mulled wine, homemade	27.01.01	Beverages - Alcohol - Wine	93401010	Wine, table, red
2646	17-0227			Mulled wine, homemade (18%ABV)	27.01.01	Beverages - Alcohol - Wine	93401010	Wine, table, red
2647	A-17-0271	17-0271		Mulligatawny soup	20.01.00	Soups - Canned & fresh & homemade	28340800	Chicken or turkey soup with vegetables and fruit, Asian Style
2648	00-03340			Multigrain batch bread TESCO FINEST (RISCK-LA)	03.04.00	Breads - Other bread	51601020	Bread, multigrain
2649	00-05683			Multigrain crispbread	03.05.00	Breads - Crisp Breads, e.g. Rivetas, Grissini, Toast Melba	54305010	Crackers, crispbread
2650	A-13-0099			Mung beans, dahl, dried, boiled in unsalted water	16.01.00	Pulses/Lentils - Pulses/lentils	41101000	Beans, dry, cooked, NS as to type and as to fat added in cooking
2651	13-0097			Mung beans, whole, dried, boiled in unsalted water	16.01.00	Pulses/Lentils - Pulses/lentils	41101000	Beans, dry, cooked, NS as to type and as to fat added in cooking
2652	A-00-00946			Mushroom Soup, Cream of, Canned, Ready to Serve	20.01.00	Soups - Canned & fresh & homemade	75607060	Mushroom soup, cream of, NS as to made with milk or water
2653	00-03551			Mushroom pasta salad, Tesco or M&S	01.02.00	Cereals & cereal dishes - Pasta & pasta dishes	58148114	Macaroni or pasta salad, made with Italian dressing

2654	00-03652			Mushroom pate	15.04.00	Vegetables - Other	75414010	Mushrooms, NS as to form, creamed
2655	02-08584			Mushroom sauce	21.03.00	Sauces & accompaniment - Other sauces, incl. brown sauce, soy sauce, ketchup, mint sauce, vinegar	28500100	Gravy, mushroom
2656	A-17-0270	17-0270		Mushroom soup, cream of, canned	20.01.00	Soups - Canned & fresh & homemade	75607060	Mushroom soup, cream of, NS as to made with milk or water
2657	A-00-03611	00-03611		Mushrooms & garlic, crumbed, fried known fat	15.04.00	Vegetables - Other	75414030	Mushrooms, batter-dipped, fried
2658	00-05632			Mushrooms fried in blended oil	15.04.00	Vegetables - Other	75219000	Mushrooms, cooked, NS as to form, NS as to fat added in cooking
2659	A-00-00610			Mushrooms, Fried	15.04.00	Vegetables - Other	75219000	Mushrooms, cooked, NS as to form, NS as to fat added in cooking
2660	A-00-06309			Mushrooms, Fried (bacon fat) 50	15.04.00	Vegetables - Other	75219000	Mushrooms, cooked, NS as to form, NS as to fat added in cooking
2661	A-00-00609			Mushrooms, Raw	15.04.00	Vegetables - Other	75115000	Mushrooms, raw
2662	A-00-03590	00-03590		Mushrooms, common, boiled, unsalted	15.04.00	Vegetables - Other	75219000	Mushrooms, cooked, NS as to form, NS as to fat added in cooking
2663	13-0287			Mushrooms, common, canned, re-heated, drained	15.04.00	Vegetables - Other	75219000	Mushrooms, cooked, NS as to form, NS as to fat added in cooking
2664	13-0416	A-13-0416		Mushrooms, common, fried in butter	15.04.00	Vegetables - Other	75219000	Mushrooms, cooked, NS as to form, NS as to fat added in cooking
2665	A-13-0286			Mushrooms, common, fried in corn oil	15.04.00	Vegetables - Other	75219000	Mushrooms, cooked, NS as to form, NS as to fat added in cooking
2666	A-13-0284	13-0284		Mushrooms, common, raw	15.04.00	Vegetables - Other	75115000	Mushrooms, raw

2667	00-03585	A-00-03585		Mushrooms, fried in specified fat	15.04.00	Vegetables - Other	75219000	Mushrooms, cooked, NS as to form, NS as to fat added in cooking
2668	13-0126	A-13-0126		Mushy peas, canned, re-heated	15.04.00	Vegetables - Other	75224013	Peas, green, cooked, from canned, NS as to fat added in cooking
2669	A-00-00533			Mussels, Boiled	09.03.00	Fish & fish dishes - Shellfish	26313160	Mussels, steamed or poached
2670	A-00-00534			Mussels, Boiled (Weighed with Shell)	09.03.00	Fish & fish dishes - Shellfish	26313160	Mussels, steamed or poached
2671	A-00-00532			Mussels, Raw	09.03.00	Fish & fish dishes - Shellfish	26313100	Mussels, raw
2672	16-0256	A-16-0256		Mussels, boiled	09.03.00	Fish & fish dishes - Shellfish	26313160	Mussels, steamed or poached
2673	A-00-00963			Mustard Powder	26.01.00	Miscellaneous - Dried herbs & spices & pastes	2024	Spices, mustard seed, ground
2674	A-00-01222			Mustard Seed	26.01.00	Miscellaneous - Dried herbs & spices & pastes	2024	Spices, mustard seed, ground
2675	A-00-00611			Mustard and Cress, Raw	15.03.00	Vegetables - Yellow & red & dark green leafy vegetables	72122100	Mustard greens, raw
2676	13-0297	A-13-0297		Mustard and cress, raw	15.03.00	Vegetables - Yellow & red & dark green leafy vegetables	72122100	Mustard greens, raw
2677	17-0362	A-17-0362		Mustard powder	26.01.00	Miscellaneous - Dried herbs & spices & pastes	2024	Spices, mustard seed, ground
2678	A-13-0839	13-0839		Mustard seeds	19.00.00	Nuts & Seeds (incl. peanut butter)	2024	Spices, mustard seed, ground
2679	17-0364	A-17-0364		Mustard, smooth	15.04.00	Vegetables - Other	75506010	Mustard
2680	A-17-0365	17-0365		Mustard, wholegrain	15.04.00	Vegetables - Other	75506010	Mustard
2681	A-00-06203			Mutton 50 roast leg	10.02.00	Meat - red - Lamb & dishes	23120100	Lamb, roast, cooked, NS as to fat eaten
2682	A-00-05227			Mutton Biriani	10.02.00	Meat - red - Lamb & dishes	27213010	Biryani with meat
2683	A-00-01296			Mutton Curry (without Bone)	10.02.00	Meat - red - Lamb & dishes	27130100	Lamb or mutton curry
2684	A-00-01304			Mutton and Greens Curry	10.02.00	Meat - red - Lamb & dishes	27130100	Lamb or mutton curry

2685	02-07235			NESQUIK MILK SHAKE MILK DRINK POWDER ANY FLAVOUR	27.05.00	Beverages - Powdered Beverages (cocoa, Horlicks, Bonvita, Ovaltine, etc)	11830150	Cocoa powder, not reconstituted
2686	02-08441			NESTLE ALMOND OATS AND MORE CEREAL FORTIFIED	02.01.00	Breakfast cereals - Oat based cereals	57316450	Cereal (General Mills Oatmeal Crisp with Almonds)
2687	02-08163			NESTLE HONEY OATS AND MORE FORTIFIED	02.01.00	Breakfast cereals - Oat based cereals	57000100	Cereal, oat, NFS
2688	02-10254			NESTLE OATS AND MORE RAISIN CEREAL	02.01.00	Breakfast cereals - Oat based cereals	57316500	Cereal (General Mills Oatmeal Crisp with Raisins)
2689	02-10257			NEW DAY HONEY HOOPS CEREAL FORTIFIED	02.03.00	Breakfast cereals - Other breakfast cereals - low fibre	57100100	Cereal, ready-to-eat, NFS
2690	02-10232			NIK NAKS ANY FLAVOUR	25.02.00	Savoury Snacks - Cereal based snacks	54401055	Cheese flavored corn snacks
2691	02-10312			NON DAIRY SOYA CREAM ALTERNATIVE	05.04.00	Milk - Other - plant based, e.g. rice, soy	12210520	Coffee creamer, soy, liquid
2692	02-06826			NON FRUIT NON LOW CAL CONCENTRATED CORDIALS	27.02.03	Beverages - Fruit based drinks - Squashes & fruit concentrates	91301050	Fruit syrup
2693	02-02266			NUT & SUGAR SWEETS	24.02.00	Confectionary - Sugar based products	91731100	Peanuts, sugar-coated
2694	02-10260			NUTRIGRAIN SOFT OATIES COOKIES FORTIFIED	04.01.00	Sweet cereal products - Biscuits	53710500	Cereal or granola bar (Kellogg's Nutri-Grain Cereal Bar)
2695	A-11-0086			Naan bread	03.04.00	Breads - Other bread	51108100	Naan, Indian flatbread
2696	00-03329	A-00-03329		Naan bread, Peswari	03.04.00	Breads - Other bread	51108100	Naan, Indian flatbread
2697	00-09570	A-00-09570		Naan bread, plain, coriander or garlic	03.04.00	Breads - Other bread	51108100	Naan, Indian flatbread
2698	00-05330			Nairns Mixed Berries Oat Biscuits	04.01.00	Sweet cereal products - Biscuits	53233010	Cookie, oatmeal, with raisins
2699	00-05834			Nature's path crispy rice cereal	02.03.00	Breakfast cereals - Other breakfast cereals - low fibre	57148500	Cereal, crispy brown rice
2700	14-0171			Nectarines	18.01.00	Fruit - Fresh	63131010	Nectarine, raw
2701	A-00-09928			Nectarines (MW6 carq)	18.01.00	Fruit - Fresh	63131010	Nectarine, raw

2702	A-00-00769			Nectarines, Raw	18.01.00	Fruit - Fresh	63131010	Nectarine, raw
2703	A-00-00770			Nectarines, Raw (Weighed with Stones)	18.01.00	Fruit - Fresh	63131010	Nectarine, raw
2704	A-00-01145			Nesquick Milk Shake Powder	27.05.00	Beverages - Powdered Beverages (cocoa, Horlicks, Bonvita, Ovaltine, etc)	11830150	Cocoa powder, not reconstituted
2705	A-13-0003	13-0003		New potatoes, boiled in unsalted water	17.01.00	Potatoes - Potatoes	71102980	Potato, boiled, NFS
2706	13-0007	A-13-0007		New potatoes, canned, re-heated, drained	17.01.00	Potatoes - Potatoes	71103300	Potato, canned, NS as to fat added in cooking
2707	13-0005			New potatoes, in skins, boiled in unsalted water	17.01.00	Potatoes - Potatoes	71103105	Potato, boiled, from fresh, peel eaten, NS as to fat added in cooking
2708	A-00-09934			New potatoes, in skins, boiled in unsalted water (MW6 folate)	17.01.00	Potatoes - Potatoes	71103105	Potato, boiled, from fresh, peel eaten, NS as to fat added in cooking
2709	00-03874			No added sugar fruit squash, made up	27.02.03	Beverages - Fruit based drinks - Squashes & fruit concentrates	92510610	Fruit juice drink
2710	00-03873			No added sugar fruit squash, undiluted	27.02.03	Beverages - Fruit based drinks - Squashes & fruit concentrates	91301050	Fruit syrup
2711	A-00-03731			NoTea at all 1982	27.04.00	Beverages - Water (still, tap, sparkling, flavoured)	94000100	Water, tap
2712	A-00-03730			NoTea today 1982	27.04.00	Beverages - Water (still, tap, sparkling, flavoured)	94000100	Water, tap
2713	00-09770	A-00-09770		Non dairy cream, UHT, aerosol	06.01.00	Dairy products - Cream & fromage frais	12210520	Coffee creamer, soy, liquid
2714	A-11-0056	11-0056		Noodles, egg, boiled	01.02.00	Cereals & cereal dishes - Pasta & pasta dishes	20110	Noodles, egg, enriched, cooked
2715	11-0057	A-11-0057		Noodles, fried	01.02.00	Cereals & cereal dishes - Pasta & pasta dishes	56130000	Pasta, cooked
2716	A-11-0059	11-0059		Noodles, plain, boiled	01.02.00	Cereals & cereal dishes - Pasta & pasta dishes	56130000	Pasta, cooked
2717	A-17-0115	17-0115		Nougat	24.02.00	Confectionary - Sugar based products	91726000	Nougat, plain

2718	A-15-0219	15-0219		Nut and vegetable roast	19.00.00	Nuts & Seeds (incl. peanut butter)	41812000	Sandwich spread, meat substitute type
2719	A-15-0212			Nut cutlets, retail, grilled	19.00.00	Nuts & Seeds (incl. peanut butter)	41812000	Sandwich spread, meat substitute type
2720	13-0840	A-13-0840		Nutmeg, ground	26.01.00	Miscellaneous - Dried herbs & spices & pastes	2025	Spices, nutmeg, ground
2721	A-11-0140			Nutri-Grain	04.05.00	Sweet cereal products - Cereal bars	53710500	Cereal or granola bar (Kellogg's Nutri-Grain Cereal Bar)
2722	00-03021	A-00-03021		Nutrigrain Fruit Bar, including Twist, Kelloggs	04.05.00	Sweet cereal products - Cereal bars	53710700	Cereal or granola bar (Kellogg's Special K bar)
2723	00-05537			Nutrigrain Oat Baked Bar	04.05.00	Sweet cereal products - Cereal bars	53710500	Cereal or granola bar (Kellogg's Nutri-Grain Cereal Bar)
2724	A-00-01133			Nuts, Average	19.00.00	Nuts & Seeds (incl. peanut butter)	42110000	Mixed nuts, NFS
2725	02-04084			OAT AND BRAN FLAKES NO ADDITIONS OWN BRAND EG SAINSBURYS	02.01.00	Breakfast cereals - Oat based cereals	57000100	Cereal, oat, NFS
2726	02-10159			OAT BASED MILK ALTERNATIVE FORTIFIED	05.04.00	Milk - Other - plant based, e.g. rice, soy	11360000	Rice milk
2727	02-08171			OAT BRAN	01.04.00	Cereals & cereal dishes - Other cereals & dishes	57602500	Oat bran, uncooked
2728	02-09363			OATCAKES PANCAKE TYPE NOT BISCUIT	25.02.00	Savoury Snacks - Cereal based snacks	55106000	Pancakes, gluten free
2729	02-07617			OATMEAL BREAD	03.04.00	Breads - Other bread	51501010	Bread, oatmeal
2730	02-09770			OATMEAL COOKIES AND BISCUITS	04.01.00	Sweet cereal products - Biscuits	53233040	Cookie, oatmeal, reduced fat, NS as to raisins
2731	02-04039			OCEAN PIE WEIGHT WATCHERS ONLY	09.01.00	Fish & fish dishes - White fish, incl. tuna	28350050	Fish chowder
2732	02-10121			OLIVE TAPENADE	18.02.00	Fruit - Canned & cooked	75510000	Olives, NFS
2733	02-10251			ONE PERCENT (1%) MILK, PASTEURISED	05.08.00	Milk - 1% milk	11100000	Milk, NFS

2734	02-03826			ONION GRAVY HOMEMADE	21.02.00	Sauces & accompaniment - Cooking sauces, incl. gravies, pesto, cooking sauces for pasta and rice dishes	75608100	Onion soup, French
2735	02-10129			ONION RELISH, chutney	22.02.00	Preserves - Chutney & Pickles (incl. gherkins, pickled onions etc)	74405010	Tomato relish
2736	02-06751			ORIENTAL CHICKEN WITH NOODLES AND VEGETABLES	11.01.00	Meat - white - Chicken & turkey & dishes	27343910	Chicken or turkey chow mein or chop suey with noodles
2737	00-03041			OUT OF DATEReady Brek, WeetabixDO NOT USE	02.01.00	Breakfast cereals - Oat based cereals	57000100	Cereal, oat, NFS
2738	02-02670			OVALTINE LIGHT INSTANT LOW FAT DRY WEIGHTS	27.05.00	Beverages - Powdered Beverages (cocoa, Horlicks, Bonvita, Ovaltine, etc)	11830260	Milk, malted, dry mix, not reconstituted
2739	02-04036			OVEN READY CHIPS, REDUCED FAT, E.G.WEIGHT WATCHERS	17.02.00	Potatoes - Potato products - other	71400990	Potato, french fries, NFS
2740	A-00-01363			Oat Bran Flakes, Kellogg's, Commonsense	02.03.00	Breakfast cereals - Other breakfast cereals - low fibre	57316300	Cereal (Health Valley Oat Bran Flakes)
2741	00-09601			Oat based biscuits	04.01.00	Sweet cereal products - Biscuits	53233040	Cookie, oatmeal, reduced fat, NS as to raisins
2742	00-05618			Oat so simple Oat bars (Original & Cranberry & Blueberry variety)	04.05.00	Sweet cereal products - Cereal bars	53710700	Cereal or granola bar (Kellogg's Special K bar)
2743	A-11-0181	11-0181		Oatcakes, retail	25.04.00	Savoury Snacks - Savoury biscuits & crackers	52215260	Tortilla, whole wheat
2744	A-00-06018			Oatcakes, retail 50	25.04.00	Savoury Snacks - Savoury biscuits & crackers	52215260	Tortilla, whole wheat
2745	00-05370			Oatibix	02.01.00	Breakfast cereals - Oat based cereals	57000100	Cereal, oat, NFS
2746	00-05371			Oatibix bitesize sultana and apple	02.01.00	Breakfast cereals - Oat based cereals	57000100	Cereal, oat, NFS

2747	A-00-00017			Oatmeal, Raw	02.01.00	Breakfast cereals - Oat based cereals	57602100	Oats, raw
2748	11-0017	A-11-0017		Oatmeal, raw	02.01.00	Breakfast cereals - Oat based cereals	57602100	Oats, raw
2749	A-00-01256			Oats, Porridge, with Milk and Butter	02.01.00	Breakfast cereals - Oat based cereals	56203067	Oatmeal, regular or quick, made with milk, fat added in cooking
2750	A-00-00612			Okra, Raw	15.04.00	Vegetables - Other	75220001	Okra, cooked, from fresh, NS as to fat added in cooking
2751	13-0301	A-13-0301		Okra, boiled in unsalted water	15.04.00	Vegetables - Other	75220001	Okra, cooked, from fresh, NS as to fat added in cooking
2752	13-0010	A-13-0010		Old potatoes, baked, flesh and skin	17.01.00	Potatoes - Potatoes	71508001	Potato, baked, peel eaten
2753	13-0011	A-13-0011		Old potatoes, baked, flesh only	17.01.00	Potatoes - Potatoes	71507005	Potato, baked, peel not eaten, with butter
2754	13-0014			Old potatoes, boiled in unsalted water	17.01.00	Potatoes - Potatoes	71102980	Potato, boiled, NFS
2755	A-00-09935			Old potatoes, boiled in unsalted water (MW6 folate)	17.01.00	Potatoes - Potatoes	71102980	Potato, boiled, NFS
2756	13-0402	A-13-0402		Old potatoes, mashed with butter	17.01.00	Potatoes - Potatoes	71501000	Potato, mashed, NFS
2757	A-13-0015	13-0015		Old potatoes, mashed with margarine	17.01.00	Potatoes - Potatoes	71501000	Potato, mashed, NFS
2758	13-0405	A-13-0405		Old potatoes, mashed with polyunsatd. margarine	17.01.00	Potatoes - Potatoes	71501000	Potato, mashed, NFS
2759	00-03994			Old potatoes, roast in blended oil MW5/671	17.01.00	Potatoes - Potatoes	71104030	Potato, roasted, NFS
2760	13-0016			Old potatoes, roast in corn oil	17.01.00	Potatoes - Potatoes	71104030	Potato, roasted, NFS
2761	A-00-00199			Olive Oil	08.02.00	Fats - Oils	82104000	Olive oil
2762	A-17-0038	17-0038		Olive oil	08.02.00	Fats - Oils	82104000	Olive oil

2763	A-00-00771			Olives, in Brine	18.01.00	Fruit - Fresh	75510000	Olives, NFS
2764	A-00-00772			Olives, in Brine (Weighed with Stones)	18.02.00	Fruit - Canned & cooked	75510000	Olives, NFS
2765	A-14-0173	14-0173		Olives, in brine	18.01.00	Fruit - Fresh	75510000	Olives, NFS
2766	12-0823	A-12-0823		Omelette, Spanish	07.00.00	Egg & egg dishes	32131220	Egg omelet or scrambled egg, with potatoes and/or onions, NS as to fat added in cooking
2767	12-0822	A-12-0822		Omelette, cheese	07.00.00	Egg & egg dishes	32130120	Egg omelet or scrambled egg, with cheese, made with butter
2768	12-0821	A-12-0821		Omelette, plain	07.00.00	Egg & egg dishes	32129990	Egg omelet or scrambled egg, NS as to fat added in cooking
2769	A-00-06101			Omelette, plain 50	07.00.00	Egg & egg dishes	32129990	Egg omelet or scrambled egg, NS as to fat added in cooking
2770	A-00-00927			Onion Sauce	21.03.00	Sauces & accompaniment - Other sauces, incl. brown sauce, soy sauce, ketchup, mint sauce, vinegar	75612010	Zucchini soup, cream of, prepared with milk
2771	A-00-03619	00-03619		Onion ring, battered, fried, fat known	15.04.00	Vegetables - Other	75415020	Onion rings, NS as to form, batter-dipped, baked or fried
2772	A-00-03621	00-03621		Onion ring, battered, fried, fat unknown	15.04.00	Vegetables - Other	75415020	Onion rings, NS as to form, batter-dipped, baked or fried
2773	A-12-0281	12-0281		Onion sauce, made with semi-skimmed milk	21.03.00	Sauces & accompaniment - Other sauces, incl. brown sauce, soy sauce, ketchup, mint sauce, vinegar	75612010	Zucchini soup, cream of, prepared with milk

2774	A-12-0282			Onion sauce, made with skimmed milk	21.03.00	Sauces & accompaniment - Other sauces, incl. brown sauce, soy sauce, ketchup, mint sauce, vinegar	75612010	Zucchini soup, cream of, prepared with milk
2775	A-12-0280			Onion sauce, made with whole milk	21.03.00	Sauces & accompaniment - Other sauces, incl. brown sauce, soy sauce, ketchup, mint sauce, vinegar	75612010	Zucchini soup, cream of, prepared with milk
2776	00-09836	A-00-09836		Onion sauce, with semi-skimmed milk	21.03.00	Sauces & accompaniment - Other sauces, incl. brown sauce, soy sauce, ketchup, mint sauce, vinegar	75612010	Zucchini soup, cream of, prepared with milk
2777	A-00-09837	00-09837		Onion sauce, with skimmed milk	21.03.00	Sauces & accompaniment - Other sauces, incl. brown sauce, soy sauce, ketchup, mint sauce, vinegar	75612010	Zucchini soup, cream of, prepared with milk
2778	A-00-09827			Onion sauce, with whole milk	21.03.00	Sauces & accompaniment - Other sauces, incl. brown sauce, soy sauce, ketchup, mint sauce, vinegar	75612010	Zucchini soup, cream of, prepared with milk
2779	00-03620	A-00-03620		Onions fried in unspecified fat	15.04.00	Vegetables - Other	75221000	Onions, cooked, NS as to form, NS as to fat added in cooking
2780	A-00-00614			Onions, Boiled	15.04.00	Vegetables - Other	75221000	Onions, cooked, NS as to form, NS as to fat added in cooking
2781	A-00-00615			Onions, Fried	15.04.00	Vegetables - Other	75221000	Onions, cooked, NS as to form, NS as to fat added in cooking
2782	A-00-06300			Onions, Fried (dripping) 50	15.04.00	Vegetables - Other	75221000	Onions, cooked, NS as to form, NS as to fat added in cooking
2783	A-00-00613			Onions, Raw	15.04.00	Vegetables - Other	75117020	Onions, mature, raw

2784	A-00-00616			Onions, Spring, Raw	15.04.00	Vegetables - Other	75117010	Onions, young green, raw
2785	A-13-0306	13-0306		Onions, boiled in unsalted water	15.04.00	Vegetables - Other	75221000	Onions, cooked, NS as to form, NS as to fat added in cooking
2786	A-13-0307	13-0307		Onions, fried in corn oil	15.04.00	Vegetables - Other	75221000	Onions, cooked, NS as to form, NS as to fat added in cooking
2787	13-0418	A-13-0418		Onions, fried in lard	15.04.00	Vegetables - Other	75221000	Onions, cooked, NS as to form, NS as to fat added in cooking
2788	A-00-03586	00-03586		Onions, fried in specified fat	15.04.00	Vegetables - Other	75221000	Onions, cooked, NS as to form, NS as to fat added in cooking
2789	13-0310	A-13-0310		Onions, pickled, cocktail/silverskin, drained	22.02.00	Preserves - Chutney & Pickles (incl. gherkins, pickled onions etc)	75221033	Onions, pearl, cooked, from canned
2790	13-0309	A-13-0309		Onions, pickled, drained	22.02.00	Preserves - Chutney & Pickles (incl. gherkins, pickled onions etc)	75221033	Onions, pearl, cooked, from canned
2791	13-0304	A-13-0304		Onions, raw	15.04.00	Vegetables - Other	75117020	Onions, mature, raw
2792	00-05392			Onken Wholegrain Biopot Strawberry Yogurt	06.03.01	Dairy products - Yoghurt & drinking yoghurts, incl. buttermilk and probiotics - full fat products	11435100	Yogurt, Greek, with oats
2793	00-05712			Optifit Probiotic Yogurt Drink (Aldi), fortified, any flavour	06.03.02	Dairy products - Yoghurt & drinking yoghurts, incl. buttermilk and probiotics - reduced or low fat products	11436000	Yogurt, liquid
2794	A-00-00884			Orange Drink, Undiluted	27.02.03	Beverages - Fruit based drinks - Squashes & fruit concentrates	91301050	Fruit syrup
2795	A-00-00886			Orange Juice, Canned, Sweetened	27.02.00	Beverages - Fruit based drinks	92550350	Orange juice beverage, 40-50% juice, light

2796	A-00-00885			Orange Juice, Canned, Unsweetened	27.02.00	Beverages - Fruit based drinks	61210220	Orange juice, 100%, canned, bottled or in a carton
2797	A-00-00775			Orange Juice, Fresh	27.02.01	Beverages - Fruit based drinks - Pure fruit juice & smoothies	61210000	Orange juice, 100%, NFS
2798	A-00-01132			Orange Squash with Added Vit. C Diluted 1:4	27.02.03	Beverages - Fruit based drinks - Squashes & fruit concentrates	92510610	Fruit juice drink
2799	A-00-01152			Orange Squash with Added Vit. C, Undiluted	27.02.03	Beverages - Fruit based drinks - Squashes & fruit concentrates	91301050	Fruit syrup
2800	A-00-01151			Orange Squash, Diluted	27.02.03	Beverages - Fruit based drinks - Squashes & fruit concentrates	92510610	Fruit juice drink
2801	A-00-06503			Orange juice 50 diluted	27.02.03	Beverages - Fruit based drinks - Squashes & fruit concentrates	9215	Orange juice, frozen concentrate, unsweetened, diluted with 3 volume water
2802	14-0284			Orange juice concentrate, unsweetened	27.02.03	Beverages - Fruit based drinks - Squashes & fruit concentrates	9214	Orange juice, frozen concentrate, unsweetened, undiluted
2803	00-03033			Orange juice drink no added sugar egTesco	27.02.02	Beverages - Fruit based drinks - Fruit juice drinks	92531030	Fruit juice drink (Sunny D)
2804	A-14-0281	14-0281		Orange juice, freshly squeezed	27.02.01	Beverages - Fruit based drinks - Pure fruit juice & smoothies	61210000	Orange juice, 100%, NFS
2805	A-14-0283	14-0283		Orange juice, unsweetened	27.02.01	Beverages - Fruit based drinks - Pure fruit juice & smoothies	61210000	Orange juice, 100%, NFS
2806	14-0175			Oranges	18.01.00	Fruit - Fresh	61119010	Orange, raw
2807	A-00-09929			Oranges (MW6 carq)	18.01.00	Fruit - Fresh	61119010	Orange, raw
2808	A-00-00773			Oranges, Raw	18.01.00	Fruit - Fresh	61119010	Orange, raw
2809	A-00-00774			Oranges, Raw (Weighed with Peel and Pips)	18.01.00	Fruit - Fresh	61119010	Orange, raw
2810	A-13-0842	13-0842		Oregano, dried, ground	26.01.00	Miscellaneous - Dried herbs & spices & pastes	2027	Spices, oregano, dried
2811	13-0841	A-13-0841		Oregano, fresh	15.04.00	Vegetables - Other	75119000	Parsley, raw
2812	00-03916			Organix Moon biscuits	28.04.00	Baby & infant foods/drinks - Biscuits	53803100	Cookie, baby food

2813	A-00-00875			Ovaltine	27.05.00	Beverages - Powdered Beverages (cocoa, Horlicks, Bonvita, Ovaltine, etc)	11830260	Milk, malted, dry mix, not reconstituted
2814	A-00-06502			Ovaltine 50	27.05.00	Beverages - Powdered Beverages (cocoa, Horlicks, Bonvita, Ovaltine, etc)	11830260	Milk, malted, dry mix, not reconstituted
2815	00-05916			Ovaltine light powder, chocolate ONLY	27.05.00	Beverages - Powdered Beverages (cocoa, Horlicks, Bonvita, Ovaltine, etc)	11830260	Milk, malted, dry mix, not reconstituted
2816	A-00-03883	12-0108	A-12-0108	Ovaltine powder	27.05.00	Beverages - Powdered Beverages (cocoa, Horlicks, Bonvita, Ovaltine, etc)	11830260	Milk, malted, dry mix, not reconstituted
2817	12-0110	A-12-0110		Ovaltine powder, made up with semi-skimmed milk	27.05.00	Beverages - Powdered Beverages (cocoa, Horlicks, Bonvita, Ovaltine, etc)	11526000	Milk, malted
2818	A-12-0111			Ovaltine powder, made up with skimmed milk	27.05.00	Beverages - Powdered Beverages (cocoa, Horlicks, Bonvita, Ovaltine, etc)	11526000	Milk, malted
2819	A-12-0109			Ovaltine powder, made up with whole milk	27.05.00	Beverages - Powdered Beverages (cocoa, Horlicks, Bonvita, Ovaltine, etc)	11526000	Milk, malted
2820	A-00-01391			Oven chips	17.02.00	Potatoes - Potato products - other	71400990	Potato, french fries, NFS
2821	A-00-03775			Oven chips 82/1415	17.02.00	Potatoes - Potato products - other	71400990	Potato, french fries, NFS
2822	13-0029	A-13-0029		Oven chips, frozen, baked	17.02.00	Potatoes - Potato products - other	71400990	Potato, french fries, NFS
2823	A-13-0030	13-0030		Oven chips, thick cut, frozen, baked	17.02.00	Potatoes - Potato products - other	71400990	Potato, french fries, NFS
2824	00-05836			Oxo Concentrated Liquid Beef Stock	26.01.00	Miscellaneous - Dried herbs & spices & pastes	6170	Soup, stock, beef, home-prepared
2825	00-05837			Oxo Concentrated Liquid Chicken Stock	26.01.00	Miscellaneous - Dried herbs & spices & pastes	6172	Soup, stock, chicken, home-prepared

2826	A-00-00962			Oxo Cubes	26.01.00	Miscellaneous - Dried herbs & spices & pastes	6981	Soup, bouillon cubes and granules, low sodium, dry
2827	A-00-00947			Oxtail Soup, Canned, Ready To Serve	20.01.00	Soups - Canned & fresh & homemade	28310150	Oxtail soup
2828	A-00-00949			Oxtail Soup, Dried, as Served	20.01.00	Soups - Canned & fresh & homemade	28310150	Oxtail soup
2829	A-17-0272	17-0272		Oxtail soup, canned	20.01.00	Soups - Canned & fresh & homemade	28310150	Oxtail soup
2830	A-00-00383			Oxtail, Stewed (Weighed with Fat and Bones)	14.02.00	Offal - Other offal & dishes, e.g. Haggis, faggots	21301000	Beef, oxtails, cooked
2831	18-0420	A-18-0420		Oxtail, stewed	14.02.00	Offal - Other offal & dishes, e.g. Haggis, faggots	21301000	Beef, oxtails, cooked
2832	A-00-01840			Oxtail, stewed with gravy, onions and carrots	14.02.00	Offal - Other offal & dishes, e.g. Haggis, faggots	21301000	Beef, oxtails, cooked
2833	17-0321	A-17-0321		Oyster sauce	21.03.00	Sauces & accompaniment - Other sauces, incl. brown sauce, soy sauce, ketchup, mint sauce, vinegar	27150200	Oyster sauce
2834	A-00-00535			Oysters, Raw	09.03.00	Fish & fish dishes - Shellfish	26315100	Oysters, raw
2835	A-00-00536			Oysters, Raw (Weighed with Shell)	09.03.00	Fish & fish dishes - Shellfish	26315100	Oysters, raw
2836	02-07129			PAELLA MADE WITH RICE, CHICKEN, COD, PRAWN, PEAS AND RED PEPPER. READYMEAL CO-OP	01.03.00	Cereals & cereal dishes - Rice & rice dishes	27360090	Paella, NFS
2837	02-08627			PANCAKES MADE WITH SEMI SKIMMED MILK	04.02.00	Sweet cereal products - Pastries, Buns & Pies	55101015	Pancakes, plain, reduced fat
2838	02-05493			PANCAKES MADE WITH SUGAR & SEMI SKIMMED MILK	04.02.00	Sweet cereal products - Pastries, Buns & Pies	55101015	Pancakes, plain, reduced fat
2839	02-06176			PANCAKES WITH EGGS AND SEMI-SKIMMED MILK	04.02.00	Sweet cereal products - Pastries, Buns & Pies	55101015	Pancakes, plain, reduced fat

2840	02-00154			PAPADUMS/POPPADOMS FRIED IN BUTTER GHEE	25.02.00	Savoury Snacks - Cereal based snacks	41311000	Papad, grilled or broiled
2841	02-02517			PARSLEY DRIED	26.01.00	Miscellaneous - Dried herbs & spices & pastes	2029	Spices, parsley, dried
2842	00-05670			PASSATA WITH COOKING LOSSES	21.02.00	Sauces & accompaniment - Cooking sauces, incl. gravies, pesto, cooking sauces for pasta and rice dishes	11549	Tomato products, canned, sauce
2843	02-04003			PASTA AND SAUCE MIXES, COOKED	01.02.00	Cereals & cereal dishes - Pasta & pasta dishes	58146210	Pasta with sauce, NFS
2844	02-00032			PASTA NOODLES EGG BOILED	01.02.00	Cereals & cereal dishes - Pasta & pasta dishes	20110	Noodles, egg, enriched, cooked
2845	02-09099			PASTA SALAD SAINSBURYS ITALIAN STYLE	01.02.00	Cereals & cereal dishes - Pasta & pasta dishes	58148114	Macaroni or pasta salad, made with Italian dressing
2846	02-03999			PASTA SAUCE, CHEESE BASED	21.02.00	Sauces & accompaniment - Cooking sauces, incl. gravies, pesto, cooking sauces for pasta and rice dishes	14650100	Cheese sauce
2847	02-08611			PASTA SHAPES IN TOMATO SAUCE FORT. WITH VITS/MINS	01.02.00	Cereals & cereal dishes - Pasta & pasta dishes	58146223	Pasta with tomato-based sauce, ready-to-heat
2848	02-03995			PASTA, EGG, FRESH, FILLED WITH CHEESE AND TOMATO ONLY	01.02.00	Cereals & cereal dishes - Pasta & pasta dishes	58131520	Ravioli, cheese-filled, with tomato sauce
2849	02-03994			PASTA, EGG, FRESH, FILLED WITH CHEESE ONLY, BOILED	01.02.00	Cereals & cereal dishes - Pasta & pasta dishes	58131510	Ravioli, cheese-filled, no sauce
2850	02-03996			PASTA, EGG, FRESH, FILLED, WITH MUSHROOMS, BOILED	01.02.00	Cereals & cereal dishes - Pasta & pasta dishes	58131590	Ravioli, cheese and spinach-filled, no sauce
2851	02-08043			PASTRAMI	12.03.00	Processed meat - Other processed meats	25221215	Pastrami, NFS
2852	02-02196			PEANUT BUTTER CRUNCHY NOT WHOLENUT	19.00.00	Nuts & Seeds (incl. peanut butter)	42202000	Peanut butter

2853	00-05671			PEANUT BUTTER REDUCED FAT	19.00.00	Nuts & Seeds (incl. peanut butter)	42202000	Peanut butter
2854	02-01818			PEAS FREEZE DRIED BOILED	15.04.00	Vegetables - Other	75224010	Peas, green, cooked, NS as to form, NS as to fat added in cooking
2855	02-02271			PEPPERMINT CREAMS DOLLY MIXTU	24.01.00	Confectionary - Chocolate based products	91760500	Truffles
2856	02-07989			PEPPERS YELLOW FRESH BOILED	15.03.00	Vegetables - Yellow & red & dark green leafy vegetables	75226040	Peppers, red, cooked, NS as to fat added in cooking
2857	02-07828			PILCHARDS CANNED IN BRINE FISH ONLY	09.02.00	Fish & fish dishes - Oily fish	26139190	Sardines, skinless, boneless, packed in water
2858	02-02406			PIMMS	27.01.04	Beverages - Alcohol - Spirits & Liqueur	93501000	Brandy
2859	02-07682			PINNI; DABRA	24.02.00	Confectionary - Sugar based products	53520150	Doughnut, cake type, chocolate covered, dipped in peanuts
2860	02-03148			PITTA BREAD, WHITE, TOASTED	03.01.00	Breads - White	51109110	Bread, pita, toasted
2861	02-03487			PITTA BREAD, WHOLEMEAL, TOASTED	03.02.00	Breads - Wholemeal	51301610	Bread, pita, whole wheat, toasted
2862	02-08574			PIZZA BASE WITH LOSSES	01.01.00	Cereals & cereal dishes - Pizza	58106514	Pizza with pepperoni, from frozen, medium crust
2863	02-07821			PLAICE BAKED GRILLED NO BUTTER NO BONES NO SKIN	09.01.00	Fish & fish dishes - White fish, incl. tuna	26115123	Flounder, baked or broiled, made without fat
2864	02-09353			PLAICE IN BREADCRUMBS FROZEN GRILLED OR BAKED NO F	09.01.00	Fish & fish dishes - White fish, incl. tuna	26115133	Flounder, coated, baked or broiled, made without fat
2865	02-08210			PLJ LEMON JUICE CONCENTRATE WITH VITAMIN C	27.02.03	Beverages - Fruit based drinks - Squashes & fruit concentrates	61210720	Orange juice, 100%, frozen, not reconstituted

2866	A-00-03819			PLJ, undiluted 82/1914	27.02.03	Beverages - Fruit based drinks - Squashes & fruit concentrates	91301050	Fruit syrup
2867	02-08403			PLUM SAUCE PURCHASED	21.03.00	Sauces & accompaniment - Other sauces, incl. brown sauce, soy sauce, ketchup, mint sauce, vinegar	91361070	Plum sauce, Asian style
2868	02-08115			POMEGRANATE FRUIT, FLESH AND PIPS	18.01.00	Fruit - Fresh	63145010	Pomegranate, raw
2869	02-08114			POMEGRANATE JUICE PURCHASED FORTIFIED	27.02.02	Beverages - Fruit based drinks - Fruit juice drinks	92531030	Fruit juice drink (Sunny D)
2870	02-01303			PORK AND EGG PIE	10.03.00	Meat - red - Pork & dishes	58125110	Quiche with meat, poultry or fish
2871	02-09402			PORK CRACKLING COOKED	10.03.00	Meat - red - Pork & dishes	22704010	Pork, cracklings, cooked
2872	02-10208			PORK MEATBALLS CANNED IN TOMATO SAUCE OR GRAVY	10.03.00	Meat - red - Pork & dishes	27160100	Meatballs, NS as to type of meat, with sauce
2873	02-08269			PORK SAUSAGE SMOKED GRILLED	13.00.00	Sausages & burgers & kebab	25221405	Pork sausage
2874	02-03784			PORK SAUSAGES, VERY LOW FAT, GRILLED	13.00.00	Sausages & burgers & kebab	25221406	Pork sausage, reduced fat
2875	02-09549			PORRIDGE MADE WITH 1/2 SKIMMED MILK & 1/2 WATER	02.01.00	Breakfast cereals - Oat based cereals	56203065	Oatmeal, regular or quick, made with milk, NS as to fat added in cooking
2876	02-10338			PORRIDGE MADE WITH ALL WHOLE MILK NO ADDED SALT	02.01.00	Breakfast cereals - Oat based cereals	56203065	Oatmeal, regular or quick, made with milk, NS as to fat added in cooking
2877	00-05693			PORRIDGE MADE WITH SEMI-SKIMMED MILK, NO WATER, NO SALT	02.01.00	Breakfast cereals - Oat based cereals	56203065	Oatmeal, regular or quick, made with milk, NS as to fat added in cooking

2878	02-10004			POTATO CRISPS FRIED IN SUNFLOWER OIL INC PREMIUM NOT WALKERS	25.01.00	Savoury Snacks - Potato based snacks	71200010	Potato chips, NFS
2879	02-08610			POTATO CRISPS VERY LOW FAT WITH ARTIFICIAL SWEETENER	25.01.00	Savoury Snacks - Potato based snacks	71200010	Potato chips, NFS
2880	02-10000			POTATO CRISPS, FRIED IN VEG OIL NOT WALKERS NOT PREMIUM CRISPS NOT FRIED IN SUNFLOWER OIL.	25.01.00	Savoury Snacks - Potato based snacks	71200010	Potato chips, NFS
2881	02-10002			POTATO CRISPS, PREMIUM, FRIED IN VEGETABLE OIL, EG PRINGLES GOURMET	25.01.00	Savoury Snacks - Potato based snacks	54402610	Potato chips, restructured, multigrain
2882	02-07227			POTATO FARL, PURCHASED	17.02.00	Potatoes - Potato products - other	51127010	Bread, potato
2883	02-07872			POTATO RINGS EG HULA HOOPS	25.01.00	Savoury Snacks - Potato based snacks	71203030	Potato chips, popped, NFS
2884	02-07085			POTATO SKINS WITH CHEESE AND BACON	17.02.00	Potatoes - Potato products - other	71411000	Potato skins, with cheese and bacon
2885	02-01884			POTATO SLICES BATTERED FR BLEN	17.02.00	Potatoes - Potato products - other	71403020	Potato, home fries, NFS
2886	02-01892			POTATOES NEW SAUTEED IN BLENDED OIL	17.02.00	Potatoes - Potato products - other	71403020	Potato, home fries, NFS
2887	02-01888			POTATOES OLD SAUTEED IN BLENDED OIL	17.02.00	Potatoes - Potato products - other	71403020	Potato, home fries, NFS
2888	02-01641			PRAWN BIRYANI	01.03.00	Cereals & cereal dishes - Rice & rice dishes	58150510	Rice, fried, with shrimp
2889	02-01621			PRAWN CHOP SUEY	09.03.00	Fish & fish dishes - Shellfish	27350050	Shrimp chow mein or chop suey with noodles
2890	02-01642			PRAWN CHOW MEIN	09.03.00	Fish & fish dishes - Shellfish	27350050	Shrimp chow mein or chop suey with noodles
2891	02-10072			PRAWN COCKTAIL SNACKS, MAIZE / RICE FLOUR CORN SNACKS, EG SKIPS	25.02.00	Savoury Snacks - Cereal based snacks	54406200	Shrimp chips

2892	02-09088			PRAWN CURRY	09.03.00	Fish & fish dishes - Shellfish	27150100	Shrimp curry
2893	02-08154			PRAWN MAYONNAISE PURCHASED	09.03.00	Fish & fish dishes - Shellfish	27450070	Shrimp salad
2894	02-07729			PROCESSED CHEESE SLICES LOW FAT	06.02.00	Dairy products - Cheese, incl. cottage cheese	14410500	Cheese, processed cheese food
2895	02-07732			PROCESSED CHEESE TYPE SLICES DAIRYLEA	06.02.00	Dairy products - Cheese, incl. cottage cheese	14410500	Cheese, processed cheese food
2896	01-10354			PROMAX PROTEIN MEAL BAR	30.00.00	Nutrition Powders & drinks	53729000	Nutrition bar or meal replacement bar, NFS
2897	02-10301			PUTTANESCA STYLE PASTA SAUCES CONTAINING TOMATOES AND OLIVES	21.02.00	Sauces & accompaniment - Cooking sauces, incl. gravies, pesto, cooking sauces for pasta and rice dishes	74404010	Spaghetti sauce
2898	00-03335			Pain au chocolat or chocolate croissant	04.02.00	Sweet cereal products - Pastries, Buns & Pies	51166200	Croissant, chocolate
2899	15-0226	A-15-0226		Pakora/bhajia, onion, fried in vegetable oil	15.04.00	Vegetables - Other	75440400	Vegetables, dipped in chick-pea flour batter, fried, Pakora
2900	15-0227	A-15-0227		Pakora/bhajia, onion, retail	15.04.00	Vegetables - Other	75440400	Vegetables, dipped in chick-pea flour batter, fried, Pakora
2901	A-00-01272			Pakorah - Mixed Vegetable Fritter	15.04.00	Vegetables - Other	75440400	Vegetables, dipped in chick-pea flour batter, fried, Pakora
2902	11-0345	A-11-0345		Pakoras	15.04.00	Vegetables - Other	75440400	Vegetables, dipped in chick-pea flour batter, fried, Pakora
2903	A-00-00897			Pale Ale, Bottled	27.01.03	Beverages - Alcohol - Beer	93101000	Beer
2904	A-17-0216			Pale ale, bottled	27.01.03	Beverages - Alcohol - Beer	93101000	Beer
2905	17-0216			Pale ale, bottled (3.5% ABV)	27.01.03	Beverages - Alcohol - Beer	93101000	Beer
2906	A-00-00200			Palm Oil	08.02.00	Fats - Oils	82101000	Vegetable oil, NFS
2907	17-0039			Palm oil	08.02.00	Fats - Oils	82101000	Vegetable oil, NFS

2908	A-00-01174			Pancake Roll,Spring Roll	10.03.00	Meat - red - Pork & dishes	58110130	Egg roll, with beef and/or pork
2909	A-00-01058			Pancake, Plain	04.02.00	Sweet cereal products - Pastries, Buns & Pies	55101000	Pancakes, plain
2910	00-09801	A-00-09801		Pancake, savoury, with skimmed milk	01.04.00	Cereals & cereal dishes - Other cereals & dishes	55100005	Pancakes, NFS
2911	A-00-00116			Pancakes	04.02.00	Sweet cereal products - Pastries, Buns & Pies	55101000	Pancakes, plain
2912	A-19-0249			Pancakes, beef, frozen, shallow-fried	10.01.00	Meat - red - Beef & veal & dishes	21104110	Beef steak, battered, fried, NS as to fat eaten
2913	A-11-0346	11-0346		Pancakes, savoury	01.04.00	Cereals & cereal dishes - Other cereals & dishes	55100005	Pancakes, NFS
2914	A-11-0347			Pancakes, savoury, made with skimmed milk	01.04.00	Cereals & cereal dishes - Other cereals & dishes	55100005	Pancakes, NFS
2915	A-11-0319			Pancakes, sweet	04.02.00	Sweet cereal products - Pastries, Buns & Pies	55100005	Pancakes, NFS
2916	A-00-06045			Pancakes, sweet 50	04.02.00	Sweet cereal products - Pastries, Buns & Pies	55100005	Pancakes, NFS
2917	A-11-0320			Pancakes, sweet, made with skimmed milk	04.02.00	Sweet cereal products - Pastries, Buns & Pies	55101015	Pancakes, plain, reduced fat
2918	00-09849	A-00-09849		Pancakes, sweet, made with whole milk	04.02.00	Sweet cereal products - Pastries, Buns & Pies	55100005	Pancakes, NFS
2919	A-00-09800	00-09800		Pancakes, sweet, with skimmed milk	04.02.00	Sweet cereal products - Pastries, Buns & Pies	55101015	Pancakes, plain, reduced fat
2920	00-05606			Panini, toasted	03.01.00	Breads - White	51109040	Bread, Italian, Grecian, Armenian, toasted
2921	A-11-0088			Papadums, fried	01.04.00	Cereals & cereal dishes - Other cereals & dishes	41311000	Papad, grilled or broiled
2922	A-00-09514	00-09514		Papadums, fried in unspecified oil	25.02.00	Savoury Snacks - Cereal based snacks	41311000	Papad, grilled or broiled
2923	00-03937			Papadums, snack or microwave-oven	25.02.00	Savoury Snacks - Cereal based snacks	41311000	Papad, grilled or broiled
2924	00-01130	A-00-01130		Papaya, Fresh, Raw	18.01.00	Fruit - Fresh	63133010	Papaya, raw

2925	13-0843	A-13-0843		Paprika	26.01.00	Miscellaneous - Dried herbs & spices & pastes	2028	Spices, paprika
2926	A-00-01223			Paprika, Tomato Powder	26.01.00	Miscellaneous - Dried herbs & spices & pastes	2028	Spices, paprika
2927	11-0089	A-00-01237	A-11-0089	Paratha	03.04.00	Breads - Other bread	51300185	Bread, paratha, wheat
2928	A-00-00617			Parsley, Raw	15.04.00	Vegetables - Other	75119000	Parsley, raw
2929	A-13-0844	13-0844		Parsley, fresh	15.04.00	Vegetables - Other	75119000	Parsley, raw
2930	A-13-0314	13-0314		Parsnip, boiled in unsalted water	15.04.00	Vegetables - Other	75222000	Parsnips, cooked, NS as to fat added in cooking
2931	13-0312			Parsnip, raw	15.04.00	Vegetables - Other	75129000	Turnip, raw
2932	A-00-00619			Parsnips, Boiled	15.04.00	Vegetables - Other	75222000	Parsnips, cooked, NS as to fat added in cooking
2933	A-00-00618			Parsnips, Raw	15.04.00	Vegetables - Other	75129000	Turnip, raw
2934	A-00-01108			Parsnips, Roast	15.04.00	Vegetables - Other	75222000	Parsnips, cooked, NS as to fat added in cooking
2935	00-03588	A-00-03588		Parsnips, roast in specified fat	15.04.00	Vegetables - Other	75222000	Parsnips, cooked, NS as to fat added in cooking
2936	A-00-00334			Partridge, Roast	11.02.00	Meat - white - Other game birds, (e.g. duck, goose, pheasant) & dishes	24403100	Quail, cooked
2937	18-0381	A-18-0381		Partridge, roasted, meat only	11.02.00	Meat - white - Other game birds, (e.g. duck, goose, pheasant) & dishes	24403100	Quail, cooked
2938	A-00-00776			Passion Fruit, Raw	18.01.00	Fruit - Fresh	63134010	Passion fruit, raw
2939	A-14-0178	14-0178		Passion fruit	18.01.00	Fruit - Fresh	63134010	Passion fruit, raw
2940	14-0285	A-14-0285		Passion fruit juice	27.02.01	Beverages - Fruit based drinks - Pure fruit juice & smoothies	61210000	Orange juice, 100%, NFS
2941	00-05366			Pasta Sauce Tomato Based, Light	21.02.00	Sauces & accompaniment - Cooking sauces, incl. gravies, pesto, cooking sauces for pasta and rice dishes	74404010	Spaghetti sauce

2942	17-0323	A-17-0323		Pasta sauce, tomato based	21.02.00	Sauces & accompaniment - Cooking sauces, incl. gravies, pesto, cooking sauces for pasta and rice dishes	74404010	Spaghetti sauce
2943	00-05459			Pasta sauce, tomato-based, with extra vegetables e.g. Tesco, Dolmio	21.02.00	Sauces & accompaniment - Cooking sauces, incl. gravies, pesto, cooking sauces for pasta and rice dishes	74404010	Spaghetti sauce
2944	19-0251	A-19-0251		Pasta with ham and mushroom sauce	01.02.00	Cereals & cereal dishes - Pasta & pasta dishes	58146323	Pasta with tomato-based sauce and meat, ready-to-heat
2945	A-19-0252			Pasta with meat and tomato sauce	10.01.00	Meat - red - Beef & veal & dishes	58146322	Pasta with tomato-based sauce and meat, home recipe
2946	19-0252			Pasta with minced beef and tomato sauce	10.01.00	Meat - red - Beef & veal & dishes	58146322	Pasta with tomato-based sauce and meat, home recipe
2947	A-00-01006			Pasta, Wholemeal, Cooked	01.02.00	Cereals & cereal dishes - Pasta & pasta dishes	56132990	Pasta, whole grain, cooked
2948	A-00-01005			Pasta, Wholemeal, Raw	01.02.00	Cereals & cereal dishes - Pasta & pasta dishes	20120	Pasta, dry, enriched (KC moved grain content from refined to wholemeal)
2949	A-00-09540			Pasta, fresh, cheese & vegetable, stuffed	01.02.00	Cereals & cereal dishes - Pasta & pasta dishes	58131590	Ravioli, cheese and spinach-filled, no sauce
2950	00-09540			Pasta, fresh, filled, cheese & vegetable, boiled	01.02.00	Cereals & cereal dishes - Pasta & pasta dishes	58131590	Ravioli, cheese and spinach-filled, no sauce
2951	00-09539	A-00-09539		Pasta, plain, fresh, cooked	01.02.00	Cereals & cereal dishes - Pasta & pasta dishes	56130000	Pasta, cooked
2952	A-00-00864			Pastilles	24.02.00	Confectionary - Sugar based products	91700010	Candy, NFS
2953	A-00-00090			Pastry, Choux, Cooked	04.02.00	Sweet cereal products - Pastries, Buns & Pies	53420250	Cream puff, no filling or icing

2954	A-00-00089			Pastry, Choux, Raw	04.02.00	Sweet cereal products - Pastries, Buns & Pies	53420250	Cream puff, no filling or icing
2955	A-00-00092			Pastry, Flaky, Cooked	04.02.00	Sweet cereal products - Pastries, Buns & Pies	53452400	Pastry, puff
2956	A-00-00091			Pastry, Flaky, Raw	04.02.00	Sweet cereal products - Pastries, Buns & Pies	18211	Puff pastry, frozen, ready-to-bake, baked
2957	A-00-00094			Pastry, Shortcrust, Cooked	04.02.00	Sweet cereal products - Pastries, Buns & Pies	53239000	Cookie, shortbread
2958	A-00-00093			Pastry, Shortcrust, Raw	04.02.00	Sweet cereal products - Pastries, Buns & Pies	53200100	Cookie, batter or dough, raw
2959	A-00-01043			Pastry, Wholemeal, Cooked	04.02.00	Sweet cereal products - Pastries, Buns & Pies	53391100	Pie shell, graham cracker
2960	19-0143	A-19-0143		Pate, liver	14.01.00	Offal - Liver & dishes	25112200	Liver paste or pate, chicken
2961	A-19-0145	19-0145		Pate, meat, low fat	10.01.00	Meat - red - Beef & veal & dishes	25112200	Liver paste or pate, chicken
2962	A-00-09622	00-09622		Pavlova, meringue with fruit & cream	07.00.00	Egg & egg dishes	32401000	Meringues
2963	A-00-00778			Paw Paw, Canned	18.02.00	Fruit - Canned & cooked	61122300	Orange, mandarin, canned or frozen, NS as to sweetened or unsweetened; sweetened, NS as to type of sweetener
2964	14-0182			Paw-paw, canned in juice	18.02.00	Fruit - Canned & cooked	61122320	Orange, mandarin, canned or frozen, juice pack
2965	A-14-0180			Paw-paw, raw	18.01.00	Fruit - Fresh	63107010	Banana, raw
2966	17-0275	A-17-0275		Pea and ham soup	20.01.00	Soups - Canned & fresh & homemade	41602010	Pea and ham soup, chunky style, canned or ready-to-serve
2967	A-12-0215			Peach melba	06.04.01	Dairy products - Ice cream & dairy desserts - full fat products	13120400	Ice cream bar or stick with fruit

2968	A-00-00784			Peaches, Canned	18.02.00	Fruit - Canned & cooked	63135110	Peach, cooked or canned, NS as to sweetened or unsweetened; sweetened, NS as to type of sweetener
2969	A-00-01131			Peaches, Canned, without Sugar	18.02.00	Fruit - Canned & cooked	63135110	Peach, cooked or canned, NS as to sweetened or unsweetened; sweetened, NS as to type of sweetener
2970	A-00-00781			Peaches, Dried, Raw	18.03.00	Fruit - Dried	62116100	Peach, dried, uncooked
2971	A-00-00783			Peaches, Dried, Stewed with Sugar	18.03.00	Fruit - Dried	62116230	Peach, dried, cooked, with sugar
2972	A-00-00782			Peaches, Dried, Stewed without Sugar	18.03.00	Fruit - Dried	62116220	Peach, dried, cooked, unsweetened
2973	A-00-00779			Peaches, Fresh, Raw	18.01.00	Fruit - Fresh	63135010	Peach, raw
2974	A-00-00780			Peaches, Fresh, Raw (Weighed with Stones)	18.01.00	Fruit - Fresh	63135010	Peach, raw
2975	A-14-0188	14-0188		Peaches, canned in juice	18.02.00	Fruit - Canned & cooked	63135170	Peach, cooked or canned, juice pack
2976	A-14-0189	14-0189		Peaches, canned in syrup	18.02.00	Fruit - Canned & cooked	63135110	Peach, cooked or canned, NS as to sweetened or unsweetened; sweetened, NS as to type of sweetener
2977	14-0185	A-14-0185		Peaches, dried	18.03.00	Fruit - Dried	62116100	Peach, dried, uncooked
2978	A-14-0187	14-0187		Peaches, dried, stewed without sugar	18.03.00	Fruit - Dried	62116220	Peach, dried, cooked, unsweetened
2979	14-0183			Peaches, raw	18.01.00	Fruit - Fresh	63135010	Peach, raw
2980	A-00-09930			Peaches, raw (MW6 carq)	18.01.00	Fruit - Fresh	63135010	Peach, raw
2981	A-00-00838			Peanut Butter, Smooth	19.00.00	Nuts & Seeds (incl. peanut butter)	42202000	Peanut butter
2982	17-0116	A-17-0116		Peanut brittle	19.00.00	Nuts & Seeds (incl. peanut butter)	91733000	Peanut brittle

2983	00-05649			Peanut butter chunky Kitkat	24.01.00	Confectionary - Chocolate based products	91718050	Honey-combed hard candy with peanut butter, chocolate covered
2984	14-0829	A-14-0829		Peanut butter, smooth	19.00.00	Nuts & Seeds (incl. peanut butter)	42202000	Peanut butter
2985	14-0830	A-14-0830		Peanut butter, wholegrain	19.00.00	Nuts & Seeds (incl. peanut butter)	42202000	Peanut butter
2986	A-17-0040	17-0040		Peanut oil	08.02.00	Fats - Oils	82105000	Peanut oil
2987	A-00-00201			Peanut, Groundnut, Arachis Oil	08.02.00	Fats - Oils	82105000	Peanut oil
2988	14-0836			Peanuts and raisins	19.00.00	Nuts & Seeds (incl. peanut butter)	42501000	Trail mix with nuts and fruit
2989	A-00-00835			Peanuts, Fresh	19.00.00	Nuts & Seeds (incl. peanut butter)	42111000	Peanuts, NFS
2990	A-00-00836			Peanuts, Fresh (Weighed with Shells)	19.00.00	Nuts & Seeds (incl. peanut butter)	42111000	Peanuts, NFS
2991	A-00-00837			Peanuts, Roasted and Salted	19.00.00	Nuts & Seeds (incl. peanut butter)	42111000	Peanuts, NFS
2992	14-0833			Peanuts, dry roasted	19.00.00	Nuts & Seeds (incl. peanut butter)	42111000	Peanuts, NFS
2993	A-00-09941			Peanuts, dry roasted (MW6 folate)	19.00.00	Nuts & Seeds (incl. peanut butter)	42111000	Peanuts, NFS
2994	14-0831	A-14-0831		Peanuts, plain	19.00.00	Nuts & Seeds (incl. peanut butter)	42111000	Peanuts, NFS
2995	A-14-0835			Peanuts, raisins and chocolate chips	19.00.00	Nuts & Seeds (incl. peanut butter)	42501500	Trail mix with chocolate
2996	14-0834	A-14-0834		Peanuts, roasted and salted	19.00.00	Nuts & Seeds (incl. peanut butter)	42111000	Peanuts, NFS
2997	A-00-00790			Pears, Canned	18.02.00	Fruit - Canned & cooked	63137110	Pear, cooked or canned, NS as to sweetened or unsweetened; sweetened, NS as to type of sweetener

2998	A-00-00789			Pears, Cooking, Stewed with Sugar	18.02.00	Fruit - Canned & cooked	63137110	Pear, cooked or canned, NS as to sweetened or unsweetened; sweetened, NS as to type of sweetener
2999	A-00-00788			Pears, Cooking, Stewed without Sugar	18.02.00	Fruit - Canned & cooked	63137110	Pear, cooked or canned, NS as to sweetened or unsweetened; sweetened, NS as to type of sweetener
3000	A-00-00785			Pears, Eating	18.01.00	Fruit - Fresh	63137010	Pear, raw
3001	A-00-01153			Pears, Raw, with Peel	18.01.00	Fruit - Fresh	63137010	Pear, raw
3002	A-14-0190	14-0190		Pears, average, raw	18.01.00	Fruit - Fresh	63137010	Pear, raw
3003	14-0192	A-14-0192		Pears, average, raw, peeled	18.01.00	Fruit - Fresh	63137010	Pear, raw
3004	14-0194	A-14-0194		Pears, average, stewed with sugar	18.02.00	Fruit - Canned & cooked	63137110	Pear, cooked or canned, NS as to sweetened or unsweetened; sweetened, NS as to type of sweetener
3005	14-0195	A-14-0195		Pears, average, stewed without sugar	18.02.00	Fruit - Canned & cooked	63137110	Pear, cooked or canned, NS as to sweetened or unsweetened; sweetened, NS as to type of sweetener
3006	A-14-0197	14-0197		Pears, canned in juice	18.02.00	Fruit - Canned & cooked	63137170	Pear, cooked or canned, juice pack
3007	A-14-0198	14-0198		Pears, canned in syrup	18.02.00	Fruit - Canned & cooked	63137110	Pear, cooked or canned, NS as to sweetened or unsweetened; sweetened, NS as to type of sweetener

3008	A-00-00624			Peas, Canned, Garden	15.04.00	Vegetables - Other	75224013	Peas, green, cooked, from canned, NS as to fat added in cooking
3009	A-00-00625			Peas, Canned, Processed	16.01.00	Pulses/Lentils - Pulses/lentils	41101000	Beans, dry, cooked, NS as to type and as to fat added in cooking
3010	A-00-00630			Peas, Chick, Bengal Gram, Raw	16.01.00	Pulses/Lentils - Pulses/lentils	16069	Lentils, raw
3011	A-00-00632			Peas, Chick, Channa Dahl	16.01.00	Pulses/Lentils - Pulses/lentils	41101000	Beans, dry, cooked, NS as to type and as to fat added in cooking
3012	A-00-00631			Peas, Chick, Cooked, Dahl	16.01.00	Pulses/Lentils - Pulses/lentils	41101000	Beans, dry, cooked, NS as to type and as to fat added in cooking
3013	A-00-00627			Peas, Dried, Boiled	16.01.00	Pulses/Lentils - Pulses/lentils	41101000	Beans, dry, cooked, NS as to type and as to fat added in cooking
3014	A-00-00621			Peas, Fresh, Boiled	15.04.00	Vegetables - Other	75224010	Peas, green, cooked, NS as to form, NS as to fat added in cooking
3015	A-00-00620			Peas, Fresh,raw	15.04.00	Vegetables - Other	75120000	Peas, green, raw
3016	A-00-00623			Peas, Frozen, Boiled	15.04.00	Vegetables - Other	75224010	Peas, green, cooked, NS as to form, NS as to fat added in cooking
3017	A-00-00622			Peas, Frozen, Raw	15.04.00	Vegetables - Other	75120000	Peas, green, raw
3018	A-00-00633			Peas, Red, Pigeon, Raw	16.01.00	Pulses/Lentils - Pulses/lentils	16069	Lentils, raw
3019	A-00-00629			Peas, Split, Boiled	16.01.00	Pulses/Lentils - Pulses/lentils	41101000	Beans, dry, cooked, NS as to type and as to fat added in cooking
3020	13-0129	A-13-0129		Peas, boiled in unsalted water	15.04.00	Vegetables - Other	75224010	Peas, green, cooked, NS as to form, NS as to fat added in cooking

3021	13-0135			Peas, canned, re-heated, drained	15.04.00	Vegetables - Other	75224013	Peas, green, cooked, from canned, NS as to fat added in cooking
3022	A-00-09907			Peas, canned, re-heated, drained (MW6 carq; MW6 folate)	15.04.00	Vegetables - Other	75224013	Peas, green, cooked, from canned, NS as to fat added in cooking
3023	A-00-01381			Peas, chick, boiled	16.01.00	Pulses/Lentils - Pulses/lentils	41101000	Beans, dry, cooked, NS as to type and as to fat added in cooking
3024	13-0134			Peas, frozen, boiled in unsalted water	15.04.00	Vegetables - Other	75224010	Peas, green, cooked, NS as to form, NS as to fat added in cooking
3025	A-00-09906			Peas, frozen, boiled in unsalted water (MW6 carq; MW6 folate)	15.04.00	Vegetables - Other	75224010	Peas, green, cooked, NS as to form, NS as to fat added in cooking
3026	13-0136	A-13-0136		Pease pudding, canned, re-heated, drained	16.01.00	Pulses/Lentils - Pulses/lentils	41304030	Peas, dry, cooked with pork
3027	14-0837	A-14-0837		Pecan nuts	19.00.00	Nuts & Seeds (incl. peanut butter)	42112000	Pecans, NFS
3028	00-05846			Penguin Chocolate Biscuit Bars, Fortified	04.01.00	Sweet cereal products - Biscuits	91703200	TWIX Caramel Cookie Bars
3029	A-00-00964			Pepper	26.01.00	Miscellaneous - Dried herbs & spices & pastes	2030	Spices, pepper, black
3030	A-13-0846	13-0846		Pepper, black	26.01.00	Miscellaneous - Dried herbs & spices & pastes	2030	Spices, pepper, black
3031	13-0847			Pepper, cayenne, ground	26.01.00	Miscellaneous - Dried herbs & spices & pastes	2031	Spices, pepper, red or cayenne
3032	13-0848	A-13-0848		Pepper, white	26.01.00	Miscellaneous - Dried herbs & spices & pastes	2030	Spices, pepper, black
3033	A-19-0108	19-0108		Pepperami	12.03.00	Processed meat - Other processed meats	22002800	Pork jerky
3034	17-0118	A-17-0118		Peppermint creams	24.01.00	Confectionary - Chocolate based products	91760500	Truffles

3035	17-0117	A-17-0117	A-00-00865	Peppermints	24.01.00	Confectionary - Chocolate based products	91760500	Truffles
3036	A-00-00635			Peppers, Boiled	15.04.00	Vegetables - Other	75226090	Peppers, hot, cooked, NS as to form, NS as to fat added in cooking
3037	A-00-00634			Peppers, Green, Raw	15.04.00	Vegetables - Other	75122100	Pepper, sweet, green, raw
3038	A-13-0316	13-0316		Peppers, capsicum, chilli, green, raw	15.04.00	Vegetables - Other	11977	Peppers, serrano, raw
3039	A-13-0317	13-0317		Peppers, capsicum, chilli, red, raw	15.03.00	Vegetables - Yellow & red & dark green leafy vegetables	75122200	Pepper, sweet, red, raw
3040	13-0318	A-13-0318		Peppers, capsicum, green, raw	15.04.00	Vegetables - Other	11977	Peppers, serrano, raw
3041	13-0320	A-13-0320		Peppers, capsicum, red, raw	15.03.00	Vegetables - Yellow & red & dark green leafy vegetables	75122200	Pepper, sweet, red, raw
3042	13-0322	A-13-0322		Peppers, capsicum, yellow, raw	15.03.00	Vegetables - Yellow & red & dark green leafy vegetables	75122200	Pepper, sweet, red, raw
3043	00-03582	A-00-03582		Peppers, green, boiled, unsalted water	15.04.00	Vegetables - Other	75226000	Peppers, green, cooked, NS as to fat added in cooking
3044	A-00-03617	00-03617		Peppers, green, fried in known fat	15.04.00	Vegetables - Other	75226000	Peppers, green, cooked, NS as to fat added in cooking
3045	00-03583	A-00-03583		Peppers, red, boiled, unsalted water	15.03.00	Vegetables - Yellow & red & dark green leafy vegetables	75226040	Peppers, red, cooked, NS as to fat added in cooking
3046	A-00-03618	00-03618		Peppers, red, fried in known fat	15.03.00	Vegetables - Yellow & red & dark green leafy vegetables	75226060	Peppers, red, cooked, fat added in cooking, NS as to type of fat
3047	A-00-03063			Perfect Balance Breakfast Cereal, Heinz Weight Watchers	02.02.00	Breakfast cereals - Other breakfast cereals - high fibre (equal or >3g/40g portion)	57100100	Cereal, ready-to-eat, NFS

3048	00-03922	A-00-03922		Pesto sauce	21.02.00	Sauces & accompaniment - Cooking sauces, incl. gravies, pesto, cooking sauces for pasta and rice dishes	81302070	Pesto sauce
3049	00-05720			Pesto, green, low fat, e.g. Sainsburys BGTY, NOT Asda	21.02.00	Sauces & accompaniment - Cooking sauces, incl. gravies, pesto, cooking sauces for pasta and rice dishes	81302070	Pesto sauce
3050	00-05731			Petit Filous Fromage Frais, fortified with vitamin D	06.01.00	Dairy products - Cream & fromage frais	14201200	Cottage cheese, farmer's
3051	A-13-0138	13-0138		Petit pois, frozen, boiled in unsalted water	15.04.00	Vegetables - Other	75224010	Peas, green, cooked, NS as to form, NS as to fat added in cooking
3052	00-03914			Petits Filous Fruity Smooth Yogurts	06.03.01	Dairy products - Yoghurt & drinking yoghurts, incl. buttermilk and probiotics - full fat products	11430000	Yogurt, NS as to type of milk, fruit
3053	A-00-00336			Pheasant, Roast	11.02.00	Meat - white - Other game birds, (e.g. duck, goose, pheasant) & dishes	24404100	Pheasant, cooked
3054	A-00-00337			Pheasant, Roast (Weighed with Bone)	11.02.00	Meat - white - Other game birds, (e.g. duck, goose, pheasant) & dishes	24404100	Pheasant, cooked
3055	18-0383	A-18-0383		Pheasant, roasted, meat only	11.02.00	Meat - white - Other game birds, (e.g. duck, goose, pheasant) & dishes	24404100	Pheasant, cooked
3056	A-17-0347	A-00-00928	17-0347	Piccalilli	22.02.00	Preserves - Chutney & Pickles (incl. gherkins, pickled onions etc)	75503100	Mustard pickles
3057	A-00-01228			Pickle, Chilli, Fresh	22.02.00	Preserves - Chutney & Pickles (incl. gherkins, pickled onions etc)	75511020	Peppers, pickled

3058	A-00-00929			Pickle, Sweet	22.02.00	Preserves - Chutney & Pickles (incl. gherkins, pickled onions etc)	75503040	Cucumber pickles, sweet
3059	17-0352	A-17-0352		Pickle, sweet	22.02.00	Preserves - Chutney & Pickles (incl. gherkins, pickled onions etc)	75503040	Cucumber pickles, sweet
3060	A-00-01049			Pie, Blackcurrant, (1 Crust, Plain Flour)	04.02.00	Sweet cereal products - Pastries, Buns & Pies	53301500	Pie, apple, one crust
3061	A-00-01051			Pie, Blackcurrant, (1 Crust, Wholemeal Flour)	04.02.00	Sweet cereal products - Pastries, Buns & Pies	53301500	Pie, apple, one crust
3062	A-00-01050			Pie, Blackcurrant, (2 Crusts, Plain Flour)	04.02.00	Sweet cereal products - Pastries, Buns & Pies	53301000	Pie, apple, two crust
3063	A-15-0243	15-0243		Pie, vegetable	15.04.00	Vegetables - Other	41812400	Vegetarian pot pie
3064	A-00-00338			Pigeon, Roast	11.02.00	Meat - white - Other game birds, (e.g. duck, goose, pheasant) & dishes	24402100	Dove, cooked, NS as to cooking method
3065	A-00-00339			Pigeon, Roast, (Weighed with Bone)	11.02.00	Meat - white - Other game birds, (e.g. duck, goose, pheasant) & dishes	24402100	Dove, cooked, NS as to cooking method
3066	18-0385			Pigeon, roasted, meat only	11.02.00	Meat - white - Other game birds, (e.g. duck, goose, pheasant) & dishes	24402100	Dove, cooked, NS as to cooking method
3067	A-11-0348			Pilau rice	01.03.00	Cereals & cereal dishes - Rice & rice dishes	56205001	Rice, white, cooked, NS as to fat added in cooking
3068	15-0249			Pilau, mushroom	01.03.00	Cereals & cereal dishes - Rice & rice dishes	58160000	Biryani with vegetables
3069	A-15-0250	15-0250		Pilau, plain	01.03.00	Cereals & cereal dishes - Rice & rice dishes	58160000	Biryani with vegetables
3070	A-16-0298			Pilau, prawn	01.03.00	Cereals & cereal dishes - Rice & rice dishes	58150510	Rice, fried, with shrimp
3071	15-0251	A-15-0251		Pilau, vegetable	01.03.00	Cereals & cereal dishes - Rice & rice dishes	58160000	Biryani with vegetables
3072	A-00-00494			Pilchards, Canned in Tomato Sauce	09.02.00	Fish & fish dishes - Oily fish	27150350	Sardines with tomato-based sauce

3073	16-0201	A-16-0201		Pilchards, canned in tomato sauce	09.02.00	Fish & fish dishes - Oily fish	27150350	Sardines with tomato-based sauce
3074	14-0839	A-14-0839		Pine nuts	19.00.00	Nuts & Seeds (incl. peanut butter)	42113000	Pine nuts
3075	A-00-00887			Pineapple Juice, Canned	27.02.00	Beverages - Fruit based drinks	61210220	Orange juice, 100%, canned, bottled or in a carton
3076	A-14-0286	14-0286		Pineapple juice, unsweetened	27.02.01	Beverages - Fruit based drinks - Pure fruit juice & smoothies	61210000	Orange juice, 100%, NFS
3077	A-00-00792			Pineapple, Canned	18.02.00	Fruit - Canned & cooked	63141110	Pineapple, cooked or canned, NS as to sweetened or unsweetened; sweetened, NS as to type of sweetener
3078	A-00-00791			Pineapple, Fresh	18.01.00	Fruit - Fresh	63141010	Pineapple, raw
3079	A-14-0211	14-0211		Pineapple, canned in juice	18.02.00	Fruit - Canned & cooked	63141110	Pineapple, cooked or canned, NS as to sweetened or unsweetened; sweetened, NS as to type of sweetener
3080	A-14-0212	14-0212		Pineapple, canned in syrup	18.02.00	Fruit - Canned & cooked	63141140	Pineapple, cooked or canned, in light syrup
3081	A-14-0210	14-0210		Pineapple, dried	18.03.00	Fruit - Dried	62120100	Pineapple, dried
3082	A-14-0208	14-0208		Pineapple, raw	18.01.00	Fruit - Fresh	63141010	Pineapple, raw
3083	A-00-01266			Pinni	04.02.00	Sweet cereal products - Pastries, Buns & Pies	53520150	Doughnut, cake type, chocolate covered, dipped in peanuts
3084	A-00-01315			Pistachio Nuts	19.00.00	Nuts & Seeds (incl. peanut butter)	42114130	Pistachio nuts, NFS
3085	A-14-0840	14-0840		Pistachio nuts, roasted and salted	19.00.00	Nuts & Seeds (incl. peanut butter)	42114130	Pistachio nuts, NFS
3086	A-00-09569	00-09569		Pitta Bread, white	03.01.00	Breads - White	51109100	Bread, pita

3087	A-00-03324	00-03324		Pitta Bread, wholemeal	03.02.00	Breads - Wholemeal	51301600	Bread, pita, whole wheat
3088	A-11-0090			Pitta bread, white	03.01.00	Breads - White	51109100	Bread, pita
3089	A-11-0349			Pizza	01.01.00	Cereals & cereal dishes - Pizza	58106514	Pizza with pepperoni, from frozen, medium crust
3090	00-05393			Pizza Hut Medium Italian Pepperoni Feast	01.01.00	Cereals & cereal dishes - Pizza	58106555	Pizza with pepperoni, from restaurant or fast food, medium crust
3091	A-00-00178			Pizza, Cheese and Tomato	01.01.00	Cereals & cereal dishes - Pizza	58106210	Pizza, cheese, from restaurant or fast food, NS as to type of crust
3092	A-00-09543			Pizza, French bread, cheese & Tomato	01.01.00	Cereals & cereal dishes - Pizza	58106305	Pizza, cheese with vegetables, from frozen, thick crust
3093	15-0254	A-15-0254		Pizza, cheese and tomato, retail, frozen	01.01.00	Cereals & cereal dishes - Pizza	58106205	Pizza, cheese, from frozen, thick crust
3094	A-00-09544	00-09544		Pizza, cheese and tomato, takeaway	01.01.00	Cereals & cereal dishes - Pizza	58106210	Pizza, cheese, from restaurant or fast food, NS as to type of crust
3095	A-15-0253			Pizza, cheese and tomato, wholemeal	01.01.00	Cereals & cereal dishes - Pizza	58109020	Pizza, cheese, whole wheat thick crust
3096	A-00-09548	00-09548		Pizza, chicken, possibly with vegetables or fruit	01.01.00	Cereals & cereal dishes - Pizza	58106725	Pizza with meat and vegetables, from restaurant or fast food, medium crust
3097	00-09549	A-00-09549		Pizza, fish, possibly with vegetables or fruit	01.01.00	Cereals & cereal dishes - Pizza	58106725	Pizza with meat and vegetables, from restaurant or fast food, medium crust
3098	A-11-0350			Pizza, frozen	01.01.00	Cereals & cereal dishes - Pizza	58106514	Pizza with pepperoni, from frozen, medium crust
3099	00-09547	A-00-09547		Pizza, ham & pineapple, frozen retail	01.01.00	Cereals & cereal dishes - Pizza	58106755	Pizza with meat and fruit, medium crust

3100	00-09545	A-00-09545		Pizza, meat,possibly with vegetable or fruit	01.01.00	Cereals & cereal dishes - Pizza	58106725	Pizza with meat and vegetables, from restaurant or fast food, medium crust
3101	15-0256			Pizza, tomato, wholemeal	01.01.00	Cereals & cereal dishes - Pizza	58109060	Pizza, cheese and vegetables, whole wheat thick crust
3102	A-00-09546	00-09546		Pizza, vegetarian	01.01.00	Cereals & cereal dishes - Pizza	58106347	Pizza with cheese and extra vegetables, medium crust
3103	A-00-00467			Plaice, Fried in Batter	09.01.00	Fish & fish dishes - White fish, incl. tuna	26115140	Flounder, coated, fried, made with oil
3104	A-00-00468			Plaice, Fried in Crumbs	09.01.00	Fish & fish dishes - White fish, incl. tuna	26115140	Flounder, coated, fried, made with oil
3105	A-00-06254			Plaice, Fried in Crumbs (dripping) 50	09.01.00	Fish & fish dishes - White fish, incl. tuna	26115140	Flounder, coated, fried, made with oil
3106	A-00-06253			Plaice, Fried in batter (dripping) 50	09.01.00	Fish & fish dishes - White fish, incl. tuna	26115140	Flounder, coated, fried, made with oil
3107	A-00-00466			Plaice, Raw	09.01.00	Fish & fish dishes - White fish, incl. tuna	26115000	Flounder, raw
3108	A-00-00469			Plaice, Steamed	09.01.00	Fish & fish dishes - White fish, incl. tuna	26115160	Flounder, steamed or poached
3109	16-0108	A-16-0108		Plaice, frozen, steamed	09.01.00	Fish & fish dishes - White fish, incl. tuna	26115160	Flounder, steamed or poached
3110	A-16-0110			Plaice, in batter, fried in blended oil	09.01.00	Fish & fish dishes - White fish, incl. tuna	26115140	Flounder, coated, fried, made with oil
3111	16-0117	A-16-0117		Plaice, in crumbs, frozen, fried in blended oil	09.01.00	Fish & fish dishes - White fish, incl. tuna	26115140	Flounder, coated, fried, made with oil
3112	A-00-01068			Plain Souffle	07.00.00	Egg & egg dishes	32129990	Egg omelet or scrambled egg, NS as to fat added in cooking
3113	A-00-00638			Plantain, Ripe, Fried	15.04.00	Vegetables - Other	71905110	Fried ripe plantain, Puerto Rican style

3114	A-00-00799			Plums, Cooking, Stewed with Sugar	18.02.00	Fruit - Canned & cooked	63143110	Plum, cooked or canned, NS as to sweetened or unsweetened; sweetened, NS as to type of sweetener
3115	A-00-00800			Plums, Cooking, Stewed with Sugar (Weighed with Stones)	18.02.00	Fruit - Canned & cooked	63143110	Plum, cooked or canned, NS as to sweetened or unsweetened; sweetened, NS as to type of sweetener
3116	A-00-00797			Plums, Cooking, Stewed without Sugar	18.02.00	Fruit - Canned & cooked	63143110	Plum, cooked or canned, NS as to sweetened or unsweetened; sweetened, NS as to type of sweetener
3117	A-00-00798			Plums, Cooking, Stewed without Sugar (Weighed with Stones)	18.02.00	Fruit - Canned & cooked	63143110	Plum, cooked or canned, NS as to sweetened or unsweetened; sweetened, NS as to type of sweetener
3118	A-00-00793			Plums, Victoria Dessert, Raw	18.01.00	Fruit - Fresh	63143010	Plum, raw
3119	A-00-00794			Plums, Victoria Dessert, Raw (Weighed with Stones)	18.01.00	Fruit - Fresh	63143010	Plum, raw
3120	14-0213			Plums, average, raw	18.01.00	Fruit - Fresh	63143010	Plum, raw
3121	A-00-09931			Plums, average, raw (MW6 carq)	18.01.00	Fruit - Fresh	63143010	Plum, raw
3122	14-0215	A-14-0215		Plums, average, stewed with sugar	18.02.00	Fruit - Canned & cooked	63143110	Plum, cooked or canned, NS as to sweetened or unsweetened; sweetened, NS as to type of sweetener

3123	14-0217	A-14-0217		Plums, average, stewed without sugar	18.02.00	Fruit - Canned & cooked	63143110	Plum, cooked or canned, NS as to sweetened or unsweetened; sweetened, NS as to type of sweetener
3124	A-14-0224	14-0224		Plums, yellow, raw	18.01.00	Fruit - Fresh	63143010	Plum, raw
3125	A-00-03622	00-03622		Polenta	01.04.00	Cereals & cereal dishes - Other cereals & dishes	56200990	Grits, NS as to regular, quick, or instant, NS as to fat added in cooking
3126	A-00-00406	19-0109	A-19-0109	Polony	12.03.00	Processed meat - Other processed meats	25221460	Pork and beef sausage
3127	A-00-01247			Pomegranate	18.01.00	Fruit - Fresh	63145010	Pomegranate, raw
3128	A-00-00801			Pomegranate Juice	27.02.01	Beverages - Fruit based drinks - Pure fruit juice & smoothies	61210000	Orange juice, 100%, NFS
3129	A-14-0288	14-0288		Pomegranate juice, fresh	27.02.01	Beverages - Fruit based drinks - Pure fruit juice & smoothies	61210000	Orange juice, 100%, NFS
3130	A-00-01138			Popcorn	24.02.00	Confectionary - Sugar based products	54403001	Popcorn, NFS
3131	A-17-0130	A-11-0019		Popcorn, candied	24.02.00	Confectionary - Sugar based products	54403059	Popcorn, microwave, kettle corn
3132	17-0131	A-11-0020	A-17-0131	Popcorn, plain	25.02.00	Savoury Snacks - Cereal based snacks	54403001	Popcorn, NFS
3133	00-05873			Popcorn, toffee flavoured, light eg Butterkist	24.02.00	Confectionary - Sugar based products	54403110	Popcorn, caramel coated
3134	13-0849	A-13-0849		Poppy seeds	19.00.00	Nuts & Seeds (incl. peanut butter)	43108010	Chia seeds
3135	A-00-06205			Pork 50 chops grilled lean and fat	10.03.00	Meat - red - Pork & dishes	22101120	Pork chop, broiled or baked, lean only eaten
3136	A-00-06206			Pork 50 leg roast lean and fat	10.03.00	Meat - red - Pork & dishes	22400110	Pork roast, NS as to cut, cooked, lean and fat eaten
3137	A-00-00306			Pork Chops, Grilled, Lean Only	10.03.00	Meat - red - Pork & dishes	22101120	Pork chop, broiled or baked, lean only eaten

3138	A-00-00307			Pork Chops, Grilled, Lean Only (Weighed with Fat and Bone)	10.03.00	Meat - red - Pork & dishes	22101120	Pork chop, broiled or baked, lean only eaten
3139	A-00-00304			Pork Chops, Grilled, Lean and Fat	10.03.00	Meat - red - Pork & dishes	22101110	Pork chop, broiled or baked, lean and fat eaten
3140	A-00-00305			Pork Chops, Grilled, Lean and Fat (Weighed with Bone)	10.03.00	Meat - red - Pork & dishes	22101110	Pork chop, broiled or baked, lean and fat eaten
3141	A-00-03742			Pork Fat (100% fat) 82/983	08.03.00	Fats - Animal based fats (solid)	81201000	Animal fat or drippings
3142	A-00-00300			Pork Fat, Average, Cooked	08.03.00	Fats - Animal based fats (solid)	81201000	Animal fat or drippings
3143	A-00-00421			Pork Pie, Individual	12.02.00	Processed meat - Processed pies	27360050	Meat pie, NFS
3144	A-00-06223			Pork Pie, Individual (dripping) 50	12.02.00	Processed meat - Processed pies	27360050	Meat pie, NFS
3145	19-0253			Pork and apple casserole	10.03.00	Meat - red - Pork & dishes	27120060	Sweet and sour pork
3146	19-0254	A-19-0254		Pork and beef meatballs in tomato sauce	10.04.00	Meat - red - Other red meat, e.g. rabbit, venison	27160100	Meatballs, NS as to type of meat, with sauce
3147	00-05684			Pork and beef meatballs, grilled or oven baked	10.03.00	Meat - red - Pork & dishes	27118110	Meatballs, Puerto Rican style
3148	A-19-0088	19-0088		Pork and beef sausages, chilled, grilled	13.00.00	Sausages & burgers & kebab	25221460	Pork and beef sausage
3149	19-0255	A-19-0255		Pork and chicken chow mein	01.02.00	Cereals & cereal dishes - Pasta & pasta dishes	27320310	Pork chow mein or chop suey with noodles
3150	19-0259			Pork and pineapple kebabs	13.00.00	Sausages & burgers & kebab	27120060	Sweet and sour pork
3151	A-00-01377			Pork chops, loin, average, grilled	10.03.00	Meat - red - Pork & dishes	22101100	Pork chop, broiled or baked, NS as to fat eaten
3152	A-19-0063	19-0063		Pork pie, individual	12.02.00	Processed meat - Processed pies	27360050	Meat pie, NFS
3153	19-0147	A-19-0147		Pork roast, frozen, cooked	10.03.00	Meat - red - Pork & dishes	22400100	Pork roast, NS as to cut, cooked, NS as to fat eaten
3154	A-19-0079	19-0079		Pork sausages, chilled, fried	13.00.00	Sausages & burgers & kebab	25221405	Pork sausage

3155	A-19-0080	19-0080		Pork sausages, chilled, grilled	13.00.00	Sausages & burgers & kebab	25221405	Pork sausage
3156	00-03565	A-00-03565		Pork sausages, fried, fat specified	13.00.00	Sausages & burgers & kebab	25221405	Pork sausage
3157	19-0086	A-19-0086		Pork sausages, reduced fat, chilled/frozen, grilled	13.00.00	Sausages & burgers & kebab	25221405	Pork sausage
3158	17-0132			Pork scratchings	10.03.00	Meat - red - Pork & dishes	22709010	Pork skin rinds
3159	A-00-00302			Pork, Belly, Grilled, Lean and Fat	10.03.00	Meat - red - Pork & dishes	22000110	Pork, NS as to cut, cooked, lean and fat eaten
3160	A-00-00301			Pork, Belly, Rashers, Raw, Lean and Fat	10.03.00	Meat - red - Pork & dishes	10123	Pork, cured, bacon, unprepared
3161	A-00-00294			Pork, Dressed Carcase, Raw	10.03.00	Meat - red - Pork & dishes	10024	Pork, fresh, loin, whole, separable lean only, raw
3162	00-01081	A-00-01081		Pork, Lean and Fat, Stewed with Gravy and Vegetables	10.03.00	Meat - red - Pork & dishes	27320140	Pork, potatoes, and vegetables including carrots, broccoli, and/or dark-green leafy; gravy
3163	A-00-00296			Pork, Lean, Average, Raw	10.03.00	Meat - red - Pork & dishes	10024	Pork, fresh, loin, whole, separable lean only, raw
3164	00-01080	A-00-01080		Pork, Lean, Stewed with Gravy and Vegetables	10.03.00	Meat - red - Pork & dishes	27320140	Pork, potatoes, and vegetables including carrots, broccoli, and/or dark-green leafy; gravy
3165	A-00-00308			Pork, Leg, Raw, Lean and Fat	10.03.00	Meat - red - Pork & dishes	22000110	Pork, NS as to cut, cooked, lean and fat eaten
3166	A-00-00310			Pork, Leg, Roast, Lean Only	10.03.00	Meat - red - Pork & dishes	22000120	Pork, NS as to cut, cooked, lean only eaten
3167	A-00-00309			Pork, Leg, Roast, Lean and Fat	10.03.00	Meat - red - Pork & dishes	22000110	Pork, NS as to cut, cooked, lean and fat eaten

3168	18-0208			Pork, belly joint, roasted, lean & fat	10.03.00	Meat - red - Pork & dishes	22000110	Pork, NS as to cut, cooked, lean and fat eaten
3169	18-0209	A-18-0209		Pork, belly slices, grilled, lean & fat	10.03.00	Meat - red - Pork & dishes	22000110	Pork, NS as to cut, cooked, lean and fat eaten
3170	18-0219			Pork, diced, casseroled, lean	10.03.00	Meat - red - Pork & dishes	22000120	Pork, NS as to cut, cooked, lean only eaten
3171	18-0226			Pork, fillet slices, grilled, lean	10.03.00	Meat - red - Pork & dishes	22000120	Pork, NS as to cut, cooked, lean only eaten
3172	18-0227	A-18-0227		Pork, fillet slices, grilled, lean & fat	10.03.00	Meat - red - Pork & dishes	22000110	Pork, NS as to cut, cooked, lean and fat eaten
3173	A-18-0233	18-0233		Pork, hand, shoulder joint, roasted, lean	10.03.00	Meat - red - Pork & dishes	22000120	Pork, NS as to cut, cooked, lean only eaten
3174	A-18-0234	18-0234		Pork, hand, shoulder joint, roasted, lean & fat	10.03.00	Meat - red - Pork & dishes	22000110	Pork, NS as to cut, cooked, lean and fat eaten
3175	A-18-0242	18-0242		Pork, leg joint, roasted well done, lean	10.03.00	Meat - red - Pork & dishes	22000120	Pork, NS as to cut, cooked, lean only eaten
3176	18-0243	A-18-0243		Pork, leg joint, roasted well done, lean & fat	10.03.00	Meat - red - Pork & dishes	22000110	Pork, NS as to cut, cooked, lean and fat eaten
3177	A-00-01376			Pork, leg, roast, average	10.03.00	Meat - red - Pork & dishes	22400100	Pork roast, NS as to cut, cooked, NS as to fat eaten
3178	A-18-0251	18-0251		Pork, loin chops, grilled, lean	10.03.00	Meat - red - Pork & dishes	22000120	Pork, NS as to cut, cooked, lean only eaten
3179	18-0252	A-18-0252		Pork, loin chops, grilled, lean & fat	10.03.00	Meat - red - Pork & dishes	22000110	Pork, NS as to cut, cooked, lean and fat eaten
3180	18-0263			Pork, loin joint, roasted, lean & fat	10.03.00	Meat - red - Pork & dishes	22000110	Pork, NS as to cut, cooked, lean and fat eaten

3181	18-0268			Pork, mince, stewed	10.03.00	Meat - red - Pork & dishes	22002000	Pork, ground or patty, cooked
3182	00-03544	A-00-03544		Pork, sausages, battered	13.00.00	Sausages & burgers & kebab	25221405	Pork sausage
3183	00-09517	A-00-09517		Pork, spare ribs	10.03.00	Meat - red - Pork & dishes	22701000	Pork, spareribs, cooked, NS as to fat eaten
3184	00-03624			Porridge Oats, Raw	02.01.00	Breakfast cereals - Oat based cereals	57602100	Oats, raw
3185	A-00-01004			Porridge, made with Milk and Water,salt added	02.01.00	Breakfast cereals - Oat based cereals	56203065	Oatmeal, regular or quick, made with milk, NS as to fat added in cooking
3186	A-00-01003			Porridge, made with Milk,salt added	02.01.00	Breakfast cereals - Oat based cereals	56203065	Oatmeal, regular or quick, made with milk, NS as to fat added in cooking
3187	00-09799	A-00-09799		Porridge, made with milk and water, no salt	02.01.00	Breakfast cereals - Oat based cereals	56203065	Oatmeal, regular or quick, made with milk, NS as to fat added in cooking
3188	A-11-0142			Porridge, made with milk and water,salt added	02.01.00	Breakfast cereals - Oat based cereals	56203065	Oatmeal, regular or quick, made with milk, NS as to fat added in cooking
3189	A-00-09798	00-09798		Porridge, made with milk, no salt	02.01.00	Breakfast cereals - Oat based cereals	56203065	Oatmeal, regular or quick, made with milk, NS as to fat added in cooking
3190	A-11-0141			Porridge, made with milk,salt added	02.01.00	Breakfast cereals - Oat based cereals	56203065	Oatmeal, regular or quick, made with milk, NS as to fat added in cooking

3191	00-03039	A-00-03039		Porridge, made with water, Recipe, no salt	02.01.00	Breakfast cereals - Oat based cereals	56203055	Oatmeal, regular or quick, made with water, NS as to fat added in cooking
3192	11-0143	A-11-0143		Porridge, made with water,salt added	02.01.00	Breakfast cereals - Oat based cereals	56203055	Oatmeal, regular or quick, made with water, NS as to fat added in cooking
3193	00-05655			Porridge, oat based, no fruit, dry, Plum, Heinz Org or Holle	28.05.00	Baby & infant foods/drinks - Dried Cereals	57804000	Oatmeal cereal, baby food, dry, instant
3194	A-00-00018			Porridge,salt added	02.01.00	Breakfast cereals - Oat based cereals	56202960	Oatmeal, NS as to regular, quick, or instant, NS as to fat added in cooking
3195	A-00-00910	A-17-0234	17-0234	Port	27.01.02	Beverages - Alcohol - Fortified wine	93402000	Wine, dessert, sweet
3196	A-17-0144	17-0144		Pot savouries, made up	01.04.00	Cereals & cereal dishes - Other cereals & dishes	22709010	Pork skin rinds
3197	A-00-01276			Potato Bhajia	15.04.00	Vegetables - Other	75440400	Vegetables, dipped in chick-pea flour batter, fried, Pakora
3198	A-00-00652			Potato Crisps	25.01.00	Savoury Snacks - Potato based snacks	71200010	Potato chips, NFS
3199	A-00-06307			Potato Crisps (dripping) 50	25.01.00	Savoury Snacks - Potato based snacks	71200010	Potato chips, NFS
3200	00-03632			Potato Croquettes oven baked	17.02.00	Potatoes - Potato products - other	71503010	Potato patty
3201	A-00-01110			Potato Croquettes, Potato Cakes	17.02.00	Potatoes - Potato products - other	71503010	Potato patty
3202	A-00-01111			Potato Salad	17.01.00	Potatoes - Potatoes	71603010	Potato salad, made with mayonnaise
3203	A-00-03810			Potato Soup 82/1453	20.01.00	Soups - Canned & fresh & homemade	71801000	Potato soup, NS as to made with milk or water

3204	00-03623			Potato Wedges, frozen	17.02.00	Potatoes - Potato products - other	11398	Potato puffs, frozen, unprepared
3205	A-00-01277			Potato and Greens Bhajia	15.04.00	Vegetables - Other	75440400	Vegetables, dipped in chick-pea flour batter, fried, Pakora
3206	A-17-0140			Potato and corn sticks	25.01.00	Savoury Snacks - Potato based snacks	71205020	Potato sticks, plain
3207	A-17-0276	17-0276		Potato and leek soup	20.01.00	Soups - Canned & fresh & homemade	71801000	Potato soup, NS as to made with milk or water
3208	17-0141	A-17-0141		Potato and tapioca snacks	25.01.00	Savoury Snacks - Potato based snacks	54402610	Potato chips, restructured, multigrain
3209	00-03336			Potato cakes (not Warburtons)	17.02.00	Potatoes - Potato products - other	71503010	Potato patty
3210	A-15-0257			Potato cakes, fried in lard	17.02.00	Potatoes - Potato products - other	71503010	Potato patty
3211	15-0258	A-15-0258		Potato cakes, fried in vegetable oil	17.02.00	Potatoes - Potato products - other	71503010	Potato patty
3212	A-17-0133	17-0133		Potato crisps	25.01.00	Savoury Snacks - Potato based snacks	71200010	Potato chips, NFS
3213	17-0134	A-17-0134		Potato crisps, crinkle cut	25.01.00	Savoury Snacks - Potato based snacks	71200010	Potato chips, NFS
3214	17-0135	A-17-0135		Potato crisps, jacket	25.01.00	Savoury Snacks - Potato based snacks	71200010	Potato chips, NFS
3215	17-0136	A-17-0136		Potato crisps, low fat	25.01.00	Savoury Snacks - Potato based snacks	71200010	Potato chips, NFS
3216	A-17-0137			Potato crisps, square	25.01.00	Savoury Snacks - Potato based snacks	71200010	Potato chips, NFS
3217	17-0139			Potato crisps, thick, crinkle-cut	25.01.00	Savoury Snacks - Potato based snacks	71200010	Potato chips, NFS
3218	A-17-0138			Potato crisps, thick-cut	25.01.00	Savoury Snacks - Potato based snacks	71200010	Potato chips, NFS
3219	A-13-0038			Potato croquettes, fried in blended oil	17.02.00	Potatoes - Potato products - other	71503010	Potato patty

3220	13-0039			Potato flour	17.02.00	Potatoes - Potato products - other	11413	Potato flour
3221	A-17-0142			Potato rings, Hula Hoop type	25.01.00	Savoury Snacks - Potato based snacks	71203030	Potato chips, popped, NFS
3222	13-0040	A-13-0040		Potato waffles, frozen, cooked	17.02.00	Potatoes - Potato products - other	71400990	Potato, french fries, NFS
3223	A-00-09845	00-09845		Potato, instant made up with semi-skimmed milk	17.02.00	Potatoes - Potato products - other	71501040	Potato, mashed, from dry mix, made with milk
3224	A-00-09846			Potato, instant made up with skimmed milk	17.02.00	Potatoes - Potato products - other	71501040	Potato, mashed, from dry mix, made with milk
3225	00-09844			Potato, instant made up with whole milk	17.02.00	Potatoes - Potato products - other	71501040	Potato, mashed, from dry mix, made with milk
3226	A-00-01109			Potatoes in Batter, Fried	17.02.00	Potatoes - Potato products - other	71403020	Potato, home fries, NFS
3227	A-00-06308			Potatoes in Batter, Fried (dripping) 50	17.02.00	Potatoes - Potato products - other	71403020	Potato, home fries, NFS
3228	A-00-00651			Potatoes, Instant, made Up	17.02.00	Potatoes - Potato products - other	71501035	Potato, mashed, from dry mix, NFS
3229	A-00-00648			Potatoes, New, Boiled	17.01.00	Potatoes - Potatoes	71102980	Potato, boiled, NFS
3230	A-00-00649			Potatoes, New, Canned	17.01.00	Potatoes - Potatoes	71103300	Potato, canned, NS as to fat added in cooking
3231	A-00-00642			Potatoes, Old, Baked	17.01.00	Potatoes - Potatoes	71100100	Potato, baked, NFS
3232	A-00-00643			Potatoes, Old, Baked (Weighed with Skins)	17.01.00	Potatoes - Potatoes	71508001	Potato, baked, peel eaten
3233	A-00-00640			Potatoes, Old, Boiled	17.01.00	Potatoes - Potatoes	71102980	Potato, boiled, NFS
3234	A-00-00645			Potatoes, Old, Chips	17.02.00	Potatoes - Potato products - other	71400990	Potato, french fries, NFS
3235	A-00-06305			Potatoes, Old, Chips (dripping) 50	17.02.00	Potatoes - Potato products - other	71400990	Potato, french fries, NFS
3236	A-00-00646			Potatoes, Old, Chips, Frozen	17.02.00	Potatoes - Potato products - other	71400990	Potato, french fries, NFS
3237	A-00-00647			Potatoes, Old, Chips, Frozen, Fried	17.02.00	Potatoes - Potato products - other	71400990	Potato, french fries, NFS
3238	A-00-00641			Potatoes, Old, Mashed	17.01.00	Potatoes - Potatoes	71501000	Potato, mashed, NFS

3239	A-00-00639			Potatoes, Old, Raw	17.01.00	Potatoes - Potatoes	11352	Potatoes, flesh and skin, raw
3240	A-00-00644			Potatoes, Old, Roast	17.01.00	Potatoes - Potatoes	71104030	Potato, roasted, NFS
3241	A-00-06306			Potatoes, Old, Roast (dripping) 50	17.02.00	Potatoes - Potato products - other	71400990	Potato, french fries, NFS
3242	15-0260	A-15-0260		Potatoes, duchesse	17.01.00	Potatoes - Potatoes	71503010	Potato patty
3243	00-03604	A-00-03604		Potatoes, roast in known fat	17.02.00	Potatoes - Potato products - other	71104030	Potato, roasted, NFS
3244	00-03655	A-00-03655		Potatoes, roast in unknown fat (veg oil:dripping 50:50)	17.01.00	Potatoes - Potatoes	71104030	Potato, roasted, NFS
3245	A-00-03799			Potted Meat 82/1441	10.01.00	Meat - red - Beef & veal & dishes	25240000	Meat spread or potted meat, NFS
3246	00-09524	A-00-09524		Prawn Crackers	25.04.00	Savoury Snacks - Savoury biscuits & crackers	54406200	Shrimp chips
3247	A-00-09513	00-09513		Prawn Madras	09.03.00	Fish & fish dishes - Shellfish	27150100	Shrimp curry
3248	A-00-03777			Prawn cocktail 82/1419	09.03.00	Fish & fish dishes - Shellfish	27150110	Shrimp cocktail
3249	00-03558	A-00-03558		Prawn cocktail WITHOUT the lettuce	09.03.00	Fish & fish dishes - Shellfish	27150110	Shrimp cocktail
3250	A-00-03559	00-03559		Prawn spring roll	09.03.00	Fish & fish dishes - Shellfish	58110200	Roll with meat and/or shrimp, vegetables and rice paper, not fried
3251	A-00-00523			Prawns, Boiled	09.03.00	Fish & fish dishes - Shellfish	26319130	Shrimp, steamed or boiled
3252	A-00-00524			Prawns, Boiled (Weighed with Shell)	09.03.00	Fish & fish dishes - Shellfish	26319130	Shrimp, steamed or boiled
3253	A-00-01313			Prawns, Fresh, Cooked	09.03.00	Fish & fish dishes - Shellfish	26319110	Shrimp, cooked, NS as to cooking method
3254	16-0239	A-16-0239		Prawns, boiled	09.03.00	Fish & fish dishes - Shellfish	26319130	Shrimp, steamed or boiled
3255	A-17-0145	17-0145		Pretzels	03.04.00	Breads - Other bread	54408400	Pretzels, soft, NFS
3256	00-05539			Primula cheese spread, plain	06.02.00	Dairy products - Cheese, incl. cottage cheese	14420200	Cheese spread, cream cheese, regular

3257	00-03721	A-00-03721		Pringles, any flavour	25.01.00	Savoury Snacks - Potato based snacks	54402610	Potato chips, restructured, multigrain
3258	00-09728	A-00-09728		Processed cheese, low fat	06.02.00	Dairy products - Cheese, incl. cottage cheese	14410500	Cheese, processed cheese food
3259	A-00-09721	A-12-0172	00-09721	Processed cheese, plain	06.02.00	Dairy products - Cheese, incl. cottage cheese	14410500	Cheese, processed cheese food
3260	A-12-0173	12-0173		Processed cheese, smoked	06.02.00	Dairy products - Cheese, incl. cottage cheese	14410500	Cheese, processed cheese food
3261	13-0140	A-13-0140		Processed peas, canned, re-heated, drained	15.04.00	Vegetables - Other	75224013	Peas, green, cooked, from canned, NS as to fat added in cooking
3262	00-09621	A-00-09621		Profiterole, with sauce	04.02.00	Sweet cereal products - Pastries, Buns & Pies	53420200	Cream puff, eclair, custard or cream filled, iced
3263	14-0289	A-14-0289		Prune juice	27.02.01	Beverages - Fruit based drinks - Pure fruit juice & smoothies	61210000	Orange juice, 100%, NFS
3264	A-00-00802			Prunes, Dried, Raw	18.03.00	Fruit - Dried	62122100	Prune, dried, uncooked
3265	A-00-00803			Prunes, Raw (Weighed with Stones)	18.01.00	Fruit - Fresh	63143010	Plum, raw
3266	A-00-00806			Prunes, Stewed, with Sugar	18.02.00	Fruit - Canned & cooked	62122230	Prune, dried, cooked, with sugar
3267	A-00-00807			Prunes, Stewed, with Sugar (Weighed with Stones)	18.02.00	Fruit - Canned & cooked	62122230	Prune, dried, cooked, with sugar
3268	A-00-00804			Prunes, Stewed, without Sugar	18.02.00	Fruit - Canned & cooked	62122200	Prune, dried, cooked, NS as to sweetened or unsweetened; sweetened, NS as to type of sweetener
3269	A-00-00805			Prunes, Stewed, without Sugar (Weighed with Stones)	18.02.00	Fruit - Canned & cooked	62122200	Prune, dried, cooked, NS as to sweetened or unsweetened; sweetened, NS as to type of sweetener

3270	A-14-0237	14-0237		Prunes, canned in juice	18.02.00	Fruit - Canned & cooked	63143170	Plum, cooked or canned, juice pack
3271	14-0238	A-14-0238		Prunes, canned in syrup	18.02.00	Fruit - Canned & cooked	62122230	Prune, dried, cooked, with sugar
3272	14-0239	A-14-0239		Prunes, ready-to-eat	18.01.00	Fruit - Fresh	62122100	Prune, dried, uncooked
3273	14-0233	A-14-0233		Prunes, stewed with sugar	18.02.00	Fruit - Canned & cooked	62122230	Prune, dried, cooked, with sugar
3274	14-0235	A-14-0235		Prunes, stewed without sugar	18.02.00	Fruit - Canned & cooked	62122200	Prune, dried, cooked, NS as to sweetened or unsweetened; sweetened, NS as to type of sweetener
3275	A-00-00051	A-11-0144		Puffed Wheat	02.03.00	Breakfast cereals - Other breakfast cereals - low fibre	57416000	Cereal, puffed wheat, plain
3276	A-00-03040	00-03040		Puffed Wheat, Own Brand	02.03.00	Breakfast cereals - Other breakfast cereals - low fibre	57416000	Cereal, puffed wheat, plain
3277	17-0147	A-17-0147		Puffed potato products e.g. Quavers	25.01.00	Savoury Snacks - Potato based snacks	71203030	Potato chips, popped, NFS
3278	A-00-01016			Pumpernickel	03.04.00	Breads - Other bread	51404010	Bread, pumpernickel
3279	A-00-09583			Pumpernickel bread	03.04.00	Breads - Other bread	51404010	Bread, pumpernickel
3280	14-0842	A-14-0842		Pumpkin seeds	19.00.00	Nuts & Seeds (incl. peanut butter)	43101050	Pumpkin seeds, NFS
3281	A-00-01112			Pumpkin, Boiled	15.04.00	Vegetables - Other	73201000	Pumpkin, cooked, NS as to form, NS as to fat added in cooking
3282	A-00-00653			Pumpkin, Raw	15.04.00	Vegetables - Other	73302010	Squash, winter type, raw
3283	00-03584	A-00-03584		Pumpkin, boiled, unsalted water	15.04.00	Vegetables - Other	73201000	Pumpkin, cooked, NS as to form, NS as to fat added in cooking
3284	00-05831			Pure Soya Soft and Creamy Spread	08.02.00	Fats - Oils	82101000	Vegetable oil, NFS
3285	00-05700			QUAKER OAT GRANOLA	02.01.00	Breakfast cereals - Oat based cereals	57227000	Cereal, granola

3286	02-08565			QUICHE, MEAT BASED, PURCHASED, EG. QUICHE LORRAINE NOT LOW FAT	07.00.00	Egg & egg dishes	58125110	Quiche with meat, poultry or fish
3287	02-10239			QUORN COTTAGE PIE, PURCHASED	15.04.00	Vegetables - Other	75414030	Mushrooms, batter-dipped, fried
3288	02-07103			QUORN PIE	15.04.00	Vegetables - Other	75414030	Mushrooms, batter-dipped, fried
3289	02-03859			QUORN SLICED MEATS, ALL VARIETIES	15.04.00	Vegetables - Other	75414030	Mushrooms, batter-dipped, fried
3290	02-06323			QUORN VEGETARIAN LASAGNE FROZEN/CHILLED READY MEAL	15.04.00	Vegetables - Other	58301150	Zucchini lasagna, diet frozen meal
3291	00-05430			Quaker Oat Bar	04.05.00	Sweet cereal products - Cereal bars	53711000	Cereal or granola bar (Quaker Chewy Granola Bar)
3292	00-05591			Quaker Oatso Simple - Golden Syrup	02.01.00	Breakfast cereals - Oat based cereals	57000100	Cereal, oat, NFS
3293	A-12-0174	12-0174		Quark	06.02.00	Dairy products - Cheese, incl. cottage cheese	14201200	Cottage cheese, farmer's
3294	A-00-00117			Queen of Puddings	04.03.00	Sweet cereal products - Cereal based puddings (not milk)	53344200	Mixed fruit tart filled with custard or cream cheese
3295	11-0323	A-11-0323		Queen of puddings	04.03.00	Sweet cereal products - Cereal based puddings (not milk)	53344200	Mixed fruit tart filled with custard or cream cheese
3296	A-00-06092			Queen of puddings 50	04.03.00	Sweet cereal products - Cereal based puddings (not milk)	53344200	Mixed fruit tart filled with custard or cream cheese
3297	A-00-00179			Quiche Lorraine	07.00.00	Egg & egg dishes	58125110	Quiche with meat, poultry or fish
3298	A-12-0285	12-0285		Quiche, Lorraine	07.00.00	Egg & egg dishes	58125110	Quiche with meat, poultry or fish
3299	A-12-0286			Quiche, Lorraine, wholemeal	07.00.00	Egg & egg dishes	58125110	Quiche with meat, poultry or fish

3300	00-05721			Quiche, Meat based, Low fat, e.g. Asda, Tesco, Weight Watchers	07.00.00	Egg & egg dishes	58125110	Quiche with meat, poultry or fish
3301	00-05542			Quiche, Spinach & Ricotta, Waitrose	07.00.00	Egg & egg dishes	58125120	Spinach quiche, meatless
3302	12-0283	A-12-0283		Quiche, cheese and egg	07.00.00	Egg & egg dishes	58125180	Cheese quiche, meatless
3303	A-12-0284			Quiche, cheese and egg, wholemeal	07.00.00	Egg & egg dishes	58125180	Cheese quiche, meatless
3304	00-05667			Quiche, cheese and onion, average Co-op,Morrisons	07.00.00	Egg & egg dishes	58125180	Cheese quiche, meatless
3305	A-12-0287	12-0287		Quiche, mushroom	07.00.00	Egg & egg dishes	58125120	Spinach quiche, meatless
3306	A-12-0288			Quiche, mushroom, wholemeal	07.00.00	Egg & egg dishes	58125120	Spinach quiche, meatless
3307	14-0241			Quinces	18.01.00	Fruit - Fresh	63137010	Pear, raw
3308	00-09993			Quinoa, cooked	01.04.00	Cereals & cereal dishes - Other cereals & dishes	56204000	Quinoa, NS as to fat added in cooking
3309	14-0843			Quinoa, raw	01.04.00	Cereals & cereal dishes - Other cereals & dishes	20036	Rice, brown, long-grain, raw
3310	00-05583			Quorn Chicken Style Pieces	15.04.00	Vegetables - Other	75414030	Mushrooms, batter-dipped, fried
3311	00-05579			Quorn Deli Ham Slices	15.04.00	Vegetables - Other	75414030	Mushrooms, batter-dipped, fried
3312	00-03629			Quorn Fillets in Breadcrumbs, e.g. Lemon & Pepper	15.04.00	Vegetables - Other	75414030	Mushrooms, batter-dipped, fried
3313	00-05824			Quorn mini savoury/scotch eggs	15.04.00	Vegetables - Other	75418060	Squash, summer, souffle
3314	00-03654	A-00-03654		Quorn sausages	15.04.00	Vegetables - Other	75414030	Mushrooms, batter-dipped, fried
3315	17-0366			Quorn, myco-protein	15.04.00	Vegetables - Other	75414030	Mushrooms, batter-dipped, fried
3316	02-08005			READY BREK CHOCOLATE FLAVOUR, DRY WEIGHT	02.03.00	Breakfast cereals - Other breakfast cereals - low fibre	57100100	Cereal, ready-to-eat, NFS

3317	02-10142			REALEAT VEGE MINCE COOKED	15.04.00	Vegetables - Other	59003000	Meat substitute, cereal- and vegetable protein-based, fried
3318	02-05545			RED BULL STIMULATION, CANNED ONLY	27.03.00	Beverages - Carbonated soft drinks	95310560	Energy drink (NOS)
3319	02-04176			RED BULL SUGAR FREE	27.03.00	Beverages - Carbonated soft drinks	95310560	Energy drink (NOS)
3320	02-10017			RED RICE - COOKED	01.03.00	Cereals & cereal dishes - Rice & rice dishes	56205011	Rice, brown, cooked, NS as to fat added in cooking
3321	02-08632			REDCURRANT JELLY FRESH HOMEMADE	22.01.00	Preserves - Jam & Marmalade	91403000	Fruit butter, all flavors
3322	02-09396			REDCURRANT JELLY PURCHASED	22.01.00	Preserves - Jam & Marmalade	91403000	Fruit butter, all flavors
3323	02-08432			REDUCED FAT CROISSANTS	04.02.00	Sweet cereal products - Pastries, Buns & Pies	51166000	Croissant
3324	02-08989			REDUCED FAT DIGESTIVES	04.01.00	Sweet cereal products - Biscuits	54102100	Graham crackers, reduced fat
3325	02-10044			REDUCED FAT SPREAD (41-62% FAT) POLYUNSATURATED, FORTIFIED WITH B6, B12, FOLIC ACID	08.04.02	Fats - Plant based fats (solid) - Reduced fat	81102000	Margarine, NFS
3326	02-07775			REDUCED FAT SPREAD (41-62%) NOT POLYUNSATURATED	08.04.02	Fats - Plant based fats (solid) - Reduced fat	81102000	Margarine, NFS
3327	02-03571			RHUBARB CRUMBLE WITH BUTTER	04.03.00	Sweet cereal products - Cereal based puddings (not milk)	53415600	Crisp, rhubarb
3328	02-05503			RIBENA JUICE DRINK RTD, APPLE, STRAWBERRY, NOT LIGHT, NOT CANNED	27.02.02	Beverages - Fruit based drinks - Fruit juice drinks	92531030	Fruit juice drink (Sunny D)
3329	02-05505			RIBENA NO ADDED SUGAR BLACKCURRANT RTD LOW CALORIE	27.02.03	Beverages - Fruit based drinks - Squashes & fruit concentrates	91301050	Fruit syrup

3330	02-02735			RICE CAKES WITH ADDED SUGAR , CARAMEL NOT CHOCOLATE	04.02.00	Sweet cereal products - Pastries, Buns & Pies	54318500	Rice cake
3331	02-03267			RICE CAKES, NOT FLAVOURED	25.04.00	Savoury Snacks - Savoury biscuits & crackers	54318500	Rice cake
3332	02-03236			RICE CAKES, SAVOURY FLAVOURED, NO ADDED SUGAR	25.04.00	Savoury Snacks - Savoury biscuits & crackers	54318500	Rice cake
3333	02-08027			RICE PUDDING MADE WITH CLOTTED CREAM	04.04.00	Sweet cereal products - Milk based puddings	13210410	Pudding, rice
3334	02-10447			RICH CHOCOLATE TART PURCHASED	04.02.00	Sweet cereal products - Pastries, Buns & Pies	53342070	Pie, chocolate cream, individual size or tart
3335	02-01320			ROAST BEEF DINNER WITH YORKSHIRE PUD POTATOES VEG	10.01.00	Meat - red - Beef & veal & dishes	28110220	Sirloin, chopped, with gravy, mashed potatoes, vegetable, frozen meal
3336	02-05065			ROAST POTATOES FROZEN IN A LIGHT BATTER, BAKED E.G. AUNT BESSIE	17.02.00	Potatoes - Potato products - other	71403020	Potato, home fries, NFS
3337	02-09358			ROAST TURKEY PLATTER READY MEAL WITH POTATOES VEG STUFFING	11.01.00	Meat - white - Chicken & turkey & dishes	28145210	Turkey with gravy, dressing, potatoes, vegetable, frozen meal
3338	02-06602			ROASTED VEGETABLE MIX	15.04.00	Vegetables - Other	75311000	Mixed vegetables, cooked, NS as to form, NS as to fat added in cooking
3339	02-10482			ROBINSONS BE NATURAL CONCENTRATED SQUASH	27.02.03	Beverages - Fruit based drinks - Squashes & fruit concentrates	91301050	Fruit syrup
3340	02-10035			ROBINSONS FRUIT AND BARLEY CONCENTRATE, ANY FLAVOUR	27.02.03	Beverages - Fruit based drinks - Squashes & fruit concentrates	91301050	Fruit syrup
3341	02-06963			ROBINSONS NO ADDED SUGAR CONCENTRATES	27.02.03	Beverages - Fruit based drinks - Squashes & fruit concentrates	91301050	Fruit syrup

3342	02-06961			ROBINSONS ORIGINAL FRUIT CONCENTRATES, NOT NAS	27.02.03	Beverages - Fruit based drinks - Squashes & fruit concentrates	91301050	Fruit syrup
3343	02-03834			ROSS JAM ROLY-POLY FROZEN	04.02.00	Sweet cereal products - Pastries, Buns & Pies	53113000	Cake, jelly roll
3344	02-00115			RYE BREAD, TOASTED	03.04.00	Breads - Other bread	51401020	Bread, rye, toasted
3345	A-00-00350			Rabbit, Raw	10.04.00	Meat - red - Other red meat, e.g. rabbit, venison	17343	Game meat, deer, ground, raw
3346	A-00-00351			Rabbit, Stewed	10.04.00	Meat - red - Other red meat, e.g. rabbit, venison	23310000	Rabbit, NS as to domestic or wild, cooked
3347	A-00-00352			Rabbit, Stewed (Weighed with Bone)	10.04.00	Meat - red - Other red meat, e.g. rabbit, venison	23310000	Rabbit, NS as to domestic or wild, cooked
3348	A-18-0388	18-0388		Rabbit, stewed, meat only	10.04.00	Meat - red - Other red meat, e.g. rabbit, venison	23310000	Rabbit, NS as to domestic or wild, cooked
3349	13-0330	A-13-0330		Radish, red, raw	15.04.00	Vegetables - Other	75125000	Radish, raw
3350	A-13-0331	13-0331		Radish, white/mooli, raw	15.04.00	Vegetables - Other	75125000	Radish, raw
3351	A-00-00654			Radishes, Raw	15.04.00	Vegetables - Other	75125000	Radish, raw
3352	A-00-03056	00-03056		Raisin Splitz/Wheats, Kelloggs	02.02.00	Breakfast cereals - Other breakfast cereals - high fibre (equal or >3g/40g portion)	57100100	Cereal, ready-to-eat, NFS
3353	A-14-0242	14-0242		Raisins	18.03.00	Fruit - Dried	62125100	Raisins
3354	A-00-00809			Raisins, Dried	18.03.00	Fruit - Dried	62125100	Raisins
3355	17-0324	A-17-0324		Raita	21.03.00	Sauces & accompaniment - Other sauces, incl. brown sauce, soy sauce, ketchup, mint sauce, vinegar	83115000	Yogurt dressing
3356	A-00-01257			Raita - A Spiced Curd	21.01.00	Sauces & accompaniment - Dressings & Mayonnaise	83115000	Yogurt dressing
3357	A-12-0289			Raita, plain	21.01.00	Sauces & accompaniment - Dressings & Mayonnaise	83115000	Yogurt dressing
3358	A-00-00203			Rapeseed Oil, Low Erucic Acid	08.02.00	Fats - Oils	82105500	Rapeseed oil
3359	A-17-0041	17-0041		Rapeseed oil	08.02.00	Fats - Oils	82105500	Rapeseed oil

3360	A-00-00813			Raspberries, Canned	18.02.00	Fruit - Canned & cooked	63219110	Raspberries, cooked or canned, NS as to sweetened or unsweetened; sweetened, NS as to type of sweetener
3361	A-00-00810			Raspberries, Raw	18.01.00	Fruit - Fresh	63219000	Raspberries, raw, NS as to color
3362	A-00-00812			Raspberries, Stewed with Sugar	18.02.00	Fruit - Canned & cooked	63219110	Raspberries, cooked or canned, NS as to sweetened or unsweetened; sweetened, NS as to type of sweetener
3363	A-00-00811			Raspberries, Stewed without Sugar	18.02.00	Fruit - Canned & cooked	63219110	Raspberries, cooked or canned, NS as to sweetened or unsweetened; sweetened, NS as to type of sweetener
3364	14-0248	A-14-0248		Raspberries, canned in syrup	18.02.00	Fruit - Canned & cooked	63219110	Raspberries, cooked or canned, NS as to sweetened or unsweetened; sweetened, NS as to type of sweetener
3365	A-14-0247	14-0247		Raspberries, frozen	18.01.00	Fruit - Fresh	63219610	Raspberries, frozen, unsweetened
3366	14-0244	A-14-0244		Raspberries, raw	18.01.00	Fruit - Fresh	63219000	Raspberries, raw, NS as to color

3367	A-14-0245	14-0245		Raspberries, stewed with sugar	18.02.00	Fruit - Canned & cooked	63219110	Raspberries, cooked or canned, NS as to sweetened or unsweetened; sweetened, NS as to type of sweetener
3368	A-14-0246	14-0246		Raspberries, stewed without sugar	18.02.00	Fruit - Canned & cooked	63219110	Raspberries, cooked or canned, NS as to sweetened or unsweetened; sweetened, NS as to type of sweetener
3369	A-00-01113	15-0263		Ratatouille	15.04.00	Vegetables - Other	75316050	Ratatouille
3370	15-0264	A-15-0264		Ratatouille, retail	15.04.00	Vegetables - Other	75316050	Ratatouille
3371	A-00-01009			Ravioli, Canned in Tomato Sauce	01.02.00	Cereals & cereal dishes - Pasta & pasta dishes	58131110	Ravioli, NS as to filling, with tomato sauce
3372	A-11-0351	11-0351		Ravioli, canned in tomato sauce	01.02.00	Cereals & cereal dishes - Pasta & pasta dishes	58131110	Ravioli, NS as to filling, with tomato sauce
3373	A-11-0145	A-00-00052		Ready Brek	02.01.00	Breakfast cereals - Oat based cereals	57000100	Cereal, oat, NFS
3374	A-00-03041			Ready Brek, Weetabix	02.01.00	Breakfast cereals - Oat based cereals	57410000	Cereal (Weetabix Whole Grain)
3375	00-05585			Realeat VegeMince (raw)	15.04.00	Vegetables - Other	41440000	Textured vegetable protein, dry
3376	00-03780	A-00-03780		Red Wine, Bourgignon, Sauce (cook in) 82/1422	21.02.00	Sauces & accompaniment - Cooking sauces, incl. gravies, pesto, cooking sauces for pasta and rice dishes	28500010	Gravy, meat or poultry, with wine
3377	13-0111	A-13-0111		Red kidney beans, canned, re-heated, drained	16.01.00	Pulses/Lentils - Pulses/lentils	41101000	Beans, dry, cooked, NS as to type and as to fat added in cooking
3378	A-13-0110	13-0110		Red kidney beans, dried, boiled in unsalted water	16.01.00	Pulses/Lentils - Pulses/lentils	41101000	Beans, dry, cooked, NS as to type and as to fat added in cooking

3379	00-05668			Red pesto, includes purchased	21.03.00	Sauces & accompaniment - Other sauces, incl. brown sauce, soy sauce, ketchup, mint sauce, vinegar	81302070	Pesto sauce (adapted by KC)
3380	A-17-0228			Red wine	27.01.01	Beverages - Alcohol - Wine	93401010	Wine, table, red
3381	17-0228			Red wine (12% ABV)	27.01.01	Beverages - Alcohol - Wine	93401010	Wine, table, red
3382	00-03926	A-00-03926		Red wine sauce, homemade	21.02.00	Sauces & accompaniment - Cooking sauces, incl. gravies, pesto, cooking sauces for pasta and rice dishes	28500010	Gravy, meat or poultry, with wine
3383	A-14-0249	14-0249		Redcurrants, raw	18.01.00	Fruit - Fresh	63117010	Currants, raw
3384	14-0250	A-14-0250		Redcurrants, stewed with sugar	18.02.00	Fruit - Canned & cooked	63101110	Applesauce, stewed apples, NS as to sweetened or unsweetened; sweetened, NS as to type of sweetener
3385	A-14-0251	14-0251		Redcurrants, stewed without sugar	18.02.00	Fruit - Canned & cooked	63101110	Applesauce, stewed apples, NS as to sweetened or unsweetened; sweetened, NS as to type of sweetener
3386	17-0354			Relish, burger/chilli/tomato	22.02.00	Preserves - Chutney & Pickles (incl. gherkins, pickled onions etc)	74405010	Tomato relish
3387	A-00-00814			Rhubarb, Raw	18.01.00	Fruit - Fresh	63147010	Rhubarb, raw
3388	A-00-00816			Rhubarb, Stewed with Sugar	18.02.00	Fruit - Canned & cooked	63147110	Rhubarb, cooked or canned, NS as to sweetened or unsweetened; sweetened, NS as to type of sweetener

3389	A-00-00815			Rhubarb, Stewed without Sugar	18.02.00	Fruit - Canned & cooked	63147110	Rhubarb, cooked or canned, NS as to sweetened or unsweetened; sweetened, NS as to type of sweetener
3390	14-0253	A-14-0253		Rhubarb, stewed with sugar	18.02.00	Fruit - Canned & cooked	63147110	Rhubarb, cooked or canned, NS as to sweetened or unsweetened; sweetened, NS as to type of sweetener
3391	A-14-0254	14-0254		Rhubarb, stewed without sugar	18.02.00	Fruit - Canned & cooked	63147110	Rhubarb, cooked or canned, NS as to sweetened or unsweetened; sweetened, NS as to type of sweetener
3392	00-01354	A-00-01354		Ribena Light/Low Sugar, Ready To Drink	27.02.02	Beverages - Fruit based drinks - Fruit juice drinks	92531030	Fruit juice drink (Sunny D)
3393	A-00-01353	00-01353		Ribena Light/Low Sugar, Undiluted	27.02.03	Beverages - Fruit based drinks - Squashes & fruit concentrates	91301050	Fruit syrup
3394	A-00-01352	00-01352		Ribena, Ready To Drink	27.02.02	Beverages - Fruit based drinks - Fruit juice drinks	92531030	Fruit juice drink (Sunny D)
3395	A-00-00888			Ribena, Undiluted	27.02.03	Beverages - Fruit based drinks - Squashes & fruit concentrates	91301050	Fruit syrup
3396	A-00-03842			Ribena, Vimto, Sugarfree, undiluted	27.02.03	Beverages - Fruit based drinks - Squashes & fruit concentrates	91301050	Fruit syrup
3397	00-03877			Ribena, vimto, sugar free, made up	27.02.03	Beverages - Fruit based drinks - Squashes & fruit concentrates	92510610	Fruit juice drink
3398	A-00-00053	A-11-0146		Rice Krispies	02.03.00	Breakfast cereals - Other breakfast cereals - low fibre	57339000	Cereal (Kellogg's Rice Krispies)
3399	00-03042	A-00-03042		Rice Krispies, Kelloggs	02.03.00	Breakfast cereals - Other breakfast cereals - low fibre	57339000	Cereal (Kellogg's Rice Krispies)

3400	A-00-03043	00-03043		Rice Krispies, Own Brand	02.03.00	Breakfast cereals - Other breakfast cereals - low fibre	57339000	Cereal (Kellogg's Rice Krispies)
3401	00-05862			Rice bran oil	08.02.00	Fats - Oils	82101000	Vegetable oil, NFS
3402	A-00-03132	00-03132		Rice cakes	25.04.00	Savoury Snacks - Savoury biscuits & crackers	54318500	Rice cake
3403	A-00-09664	00-09664		Rice dessert,chilled,fruity (Muller)	04.04.00	Sweet cereal products - Milk based puddings	13210410	Pudding, rice
3404	00-03367			Rice drink non-dairy e.g Rice Dream	05.04.00	Milk - Other - plant based, e.g. rice, soy	11360000	Rice milk
3405	11-0021			Rice flour	01.03.00	Cereals & cereal dishes - Rice & rice dishes	20061	Rice flour, white, unenriched
3406	00-05635			Rice noodles, boiled	01.03.00	Cereals & cereal dishes - Rice & rice dishes	56117090	Rice noodles, cooked
3407	00-05662			Rice pudding made with cream, purchased, ready to eat	04.04.00	Sweet cereal products - Milk based puddings	13210410	Pudding, rice
3408	A-12-0248	00-09676	A-00-09676	Rice pudding, canned	04.04.00	Sweet cereal products - Milk based puddings	13210410	Pudding, rice
3409	00-09677	A-00-09677		Rice pudding, canned, low fat	04.04.00	Sweet cereal products - Milk based puddings	13210410	Pudding, rice
3410	A-00-01008			Rice, Brown, Boiled	01.03.00	Cereals & cereal dishes - Rice & rice dishes	56205011	Rice, brown, cooked, NS as to fat added in cooking
3411	A-00-01007			Rice, Brown, Raw	01.03.00	Cereals & cereal dishes - Rice & rice dishes	20036	Rice, brown, long-grain, raw
3412	A-00-00020			Rice, Polished, Boiled	01.03.00	Cereals & cereal dishes - Rice & rice dishes	56205001	Rice, white, cooked, NS as to fat added in cooking
3413	A-00-00019			Rice, Polished, Raw	01.03.00	Cereals & cereal dishes - Rice & rice dishes	20044	Rice, white, long-grain, regular, raw, enriched
3414	A-00-03793			Rice, Sweetcorn and Peppers (B.E.) 82/1435	01.03.00	Cereals & cereal dishes - Rice & rice dishes	58160400	Rice, white, with corn, NS as to fat added in cooking
3415	A-11-0147	A-00-01024		Ricicles	02.03.00	Breakfast cereals - Other breakfast cereals - low fibre	57100100	Cereal, ready-to-eat, NFS

3416	A-00-03044			Ricicles, Kelloggs	02.03.00	Breakfast cereals - Other breakfast cereals - low fibre	57100100	Cereal, ready-to-eat, NFS
3417	00-05610			Risoto rice, Arborio, RAW	01.03.00	Cereals & cereal dishes - Rice & rice dishes	20044	Rice, white, long-grain, regular, raw, enriched
3418	00-05609			Risotto rice, Arborio, boiled	01.03.00	Cereals & cereal dishes - Rice & rice dishes	56205001	Rice, white, cooked, NS as to fat added in cooking
3419	11-0352	A-11-0352		Risotto, plain	01.03.00	Cereals & cereal dishes - Rice & rice dishes	56205001	Rice, white, cooked, NS as to fat added in cooking
3420	A-00-03797			Rissoles (B.E.) 82/1439	01.04.00	Cereals & cereal dishes - Other cereals & dishes	27260050	Meatballs, with breading, NS as to type of meat, with gravy
3421	00-03130	A-00-03130		Ritz crackers, original flavour	25.04.00	Savoury Snacks - Savoury biscuits & crackers	54301030	Crackers, butter (Ritz)
3422	00-05783			Roast parsnips in a light batter, Frozen, Baked eg Aunt Bessie	15.04.00	Vegetables - Other	75222000	Parsnips, cooked, NS as to fat added in cooking
3423	A-00-00080			Rock Cakes	04.02.00	Sweet cereal products - Pastries, Buns & Pies	52105200	Scone, with fruit
3424	A-16-0134	16-0134		Rock Salmon/Dogfish, in batter, fried in blended oil	09.01.00	Fish & fish dishes - White fish, incl. tuna	26141140	Sea bass, coated, fried
3425	A-16-0133			Rock Salmon/Dogfish, raw	09.01.00	Fish & fish dishes - White fish, incl. tuna	15091	Fish, sea bass, mixed species, raw
3426	11-0210	A-11-0210		Rock cakes	04.02.00	Sweet cereal products - Pastries, Buns & Pies	52105200	Scone, with fruit
3427	A-00-06062			Rock cakes 50	04.02.00	Sweet cereal products - Pastries, Buns & Pies	52105200	Scone, with fruit
3428	A-00-00551			Roe, Cod, Hard, Fried	09.01.00	Fish & fish dishes - White fish, incl. tuna	26207110	Roe, shad, cooked
3429	A-00-00550			Roe, Cod, Hard, Raw	09.01.00	Fish & fish dishes - White fish, incl. tuna	26209100	Roe, herring
3430	A-00-00553			Roe, Herring Soft,fried	09.02.00	Fish & fish dishes - Oily fish	26207110	Roe, shad, cooked
3431	A-00-00552			Roe, Herring,soft,raw	09.02.00	Fish & fish dishes - Oily fish	26209100	Roe, herring
3432	16-0300	A-16-0300		Roe, cod, hard, fried in blended oil	09.01.00	Fish & fish dishes - White fish, incl. tuna	26207110	Roe, shad, cooked

3433	A-16-0303	16-0303		Roe, herring, soft, fried in blended oil	09.02.00	Fish & fish dishes - Oily fish	26207110	Roe, shad, cooked
3434	A-00-06012			Rolls (as white bread) 50	03.01.00	Breads - White	51150000	Roll, white, soft
3435	A-00-00040			Rolls, Brown, Crusty	03.03.00	Breads - Brown/Granary/Wheatgerm	51320010	Roll, wheat or cracked wheat
3436	A-00-00041			Rolls, Brown, Soft	03.03.00	Breads - Brown/Granary/Wheatgerm	51320010	Roll, wheat or cracked wheat
3437	A-00-00044			Rolls, Starch Reduced	03.01.00	Breads - White	51150000	Roll, white, soft
3438	A-00-00042			Rolls, White, Crusty	03.01.00	Breads - White	51153000	Roll, white, hard
3439	A-00-00043			Rolls, White, Soft	03.01.00	Breads - White	51150000	Roll, white, soft
3440	A-17-0229			Rose wine, medium	27.01.01	Beverages - Alcohol - Wine	93401010	Wine, table, red
3441	17-0229			Rose wine, medium (11% ABV)	27.01.01	Beverages - Alcohol - Wine	93401010	Wine, table, red
3442	A-00-00889			Rosehip Syrup, Undiluted	27.02.03	Beverages - Fruit based drinks - Squashes & fruit concentrates	91301050	Fruit syrup
3443	13-0851	A-13-0851		Rosemary, dried	26.01.00	Miscellaneous - Dried herbs & spices & pastes	2027	Spices, oregano, dried
3444	13-0850			Rosemary, fresh	15.04.00	Vegetables - Other	75119000	Parsley, raw
3445	00-05573			Rubicon juice (eg Guava, Mango)	27.02.01	Beverages - Fruit based drinks - Pure fruit juice & smoothies	61210000	Orange juice, 100%, NFS
3446	A-11-0263			Rum baba	04.02.00	Sweet cereal products - Pastries, Buns & Pies	53118410	Rum cake, without icing
3447	13-0114	A-13-0114		Runner beans, boiled in unsalted water	15.04.00	Vegetables - Other	75204960	Beans, string, cooked, NS as to form, NS as to color, made with oil
3448	A-00-00021			Rye Flour (100%)	01.04.00	Cereals & cereal dishes - Other cereals & dishes	20080	Wheat flour, whole-grain
3449	11-0091	A-11-0091		Rye bread	03.04.00	Breads - Other bread	51401010	Bread, rye
3450	11-0022			Rye flour, whole	01.04.00	Cereals & cereal dishes - Other cereals & dishes	20080	Wheat flour, whole-grain
3451	00-05535			Ryvita Goodness bar	04.05.00	Sweet cereal products - Cereal bars	53712100	Cereal or Granola bar, NFS
3452	00-03168			Ryvita, Sunflower seeds and oats	03.05.00	Breads - Crisp Breads, e.g. Rivetas, Grissini, Toast Melba	54305010	Crackers, crispbread

3453	02-08616			SAINSBURYS FRUIT AND YOGURT BALANCE BAR FORTIFIED	04.05.00	Sweet cereal products - Cereal bars	53710502	Cereal or granola bar (Kellogg's Nutri-Grain Yogurt Bar)
3454	00-03943			SAINSBURYS mayonnaise (RISCK-LA)	21.01.00	Sauces & accompaniment - Dressings & Mayonnaise	83107000	Mayonnaise, regular
3455	02-06644			SALMON FISHCAKES RETAIL	09.02.00	Fish & fish dishes - Oily fish	27250070	Salmon cake or patty
3456	02-02831			SALMON OCEAN PIE E.G. YOUNGS	09.01.00	Fish & fish dishes - White fish, incl. tuna	27350070	Tuna pot pie
3457	00-09985	A-00-09985		SALT added at table MARKER DUMMY	26.02.00	Miscellaneous - Salt and salt substitutes	2047	Salt, table
3458	02-02443			SANDWICH SPREAD	21.01.00	Sauces & accompaniment - Dressings & Mayonnaise	83100200	Salad dressing, NFS, for sandwiches
3459	02-10428			SARDINES, FRESH, GRILLED	09.02.00	Fish & fish dishes - Oily fish	26139110	Sardines, cooked
3460	02-01912			SAUERKRAUT	15.02.00	Vegetables - Brassicacea	75230000	Sauerkraut, cooked, NS as to fat added in cooking
3461	02-10067			SAUSAGE AND EGG IN A MUFFIN, BAGEL OR ROLL, TAKEAWAY	13.00.00	Sausages & burgers & kebab	32202060	Egg and sausage on biscuit
3462	02-08772			SAUSAGE MEAT STUFFING	13.00.00	Sausages & burgers & kebab	58128250	Dressing with meat and vegetables
3463	02-07787			SAUSAGES PORK SKINLESS GRILLED	13.00.00	Sausages & burgers & kebab	25221405	Pork sausage
3464	02-01283			SAUSAGES, LOW FAT, PORK, GRILLED	13.00.00	Sausages & burgers & kebab	25221406	Pork sausage, reduced fat
3465	02-07785			SAUSAGES, PORK, ECONOMY, GRILLED	13.00.00	Sausages & burgers & kebab	25221405	Pork sausage
3466	02-10062			SAVOURY CRACKERS, NOT CREAM, NOT WATER BISCUITS, NOT WHOLEMEAL	25.04.00	Savoury Snacks - Savoury biscuits & crackers	54001000	Crackers, NFS
3467	02-10063			SAVOURY CRACKERS, REDUCED FAT	25.04.00	Savoury Snacks - Savoury biscuits & crackers	54301100	Crackers, butter, reduced fat
3468	02-01576			SCALLOPS STEAMED	09.03.00	Fish & fish dishes - Shellfish	26317130	Scallops, steamed or boiled

3469	02-07764			SCOTCH EGG MINI	07.00.00	Egg & egg dishes	32202060	Egg and sausage on biscuit
3470	02-08711			SCRAMBLED EGG NO FAT SEMI SKIMMED MILK	07.00.00	Egg & egg dishes	32130070	Egg omelet or scrambled egg, made without fat
3471	02-10149			SEA BASS BAKED OR GRILLED	09.01.00	Fish & fish dishes - White fish, incl. tuna	26141121	Sea bass, baked or broiled, fat not added in cooking
3472	02-10271			SEAFOOD COCKTAIL SANDWICH FILLER	09.01.00	Fish & fish dishes - White fish, incl. tuna	27450080	Seafood salad
3473	02-08137			SEASONED FRIES/CHIPS OVEN BAKED	17.02.00	Potatoes - Potato products - other	71400990	Potato, french fries, NFS
3474	02-08143			SEEDED OR MULTISEED BAGELS	03.04.00	Breads - Other bread	51180010	Bagel
3475	02-10022			SELF RAISING WHOLEMEAL FLOUR	01.04.00	Cereals & cereal dishes - Other cereals & dishes	20080	Wheat flour, whole-grain
3476	02-10498			SEMI-SKIMMED DRIED MILK POWDER	05.02.00	Milk - Semi-skimmed milk	11810000	Milk, dry, not reconstituted, NS as to fat content
3477	02-08190			SHREDDED WHEAT FRUITFUL MINIWHEAT AND OWN BRANDS. NOT KELLOGGS RAISIN WHEATS	02.02.00	Breakfast cereals - Other breakfast cereals - high fibre (equal or >3g/40g portion)	57417000	Cereal (Post Shredded Wheat)
3478	02-10510			SHREDDIES OWN BRAND, NOT FROSTED, NOT COCO, NOT NESTLE	02.02.00	Breakfast cereals - Other breakfast cereals - high fibre (equal or >3g/40g portion)	57411000	Cereal (General Mills Chex Wheat)
3479	02-09788			SIMNEL CAKE	04.02.00	Sweet cereal products - Pastries, Buns & Pies	53118100	Cake, sponge, without icing or filling
3480	02-10416			SKIMMED GOATS MILK PASTEURISED	05.05.00	Milk - Other - animal based, e.g. goat	11100000	Milk, NFS
3481	02-10299			SLIMFAST BARS; CHOCOLATE PEANUT AND CHOCOLATE CARAMEL	24.01.00	Confectionary - Chocolate based products	53720400	Nutrition bar (Slim Fast Original Meal Bar)

3482	00-05691			SMOOTHIES MADE WITH DAIRY AND FRUIT, BOTTLED, PURCHASED	27.02.01	Beverages - Fruit based drinks - Pure fruit juice & smoothies	11553110	Fruit smoothie, with whole fruit and dairy
3483	02-05384			SOUPS WITH PASTA	20.01.00	Soups - Canned & fresh & homemade	58400100	Noodle soup, NFS
3484	02-09366			SOUR CREAM BASED DIPS EG. ST IVEL OWN BRAND	21.03.00	Sauces & accompaniment - Other sauces, incl. brown sauce, soy sauce, ketchup, mint sauce, vinegar	12350010	Dip, NFS
3485	02-03451			SOUR CREAM DIP, REDUCED FAT E.G ASDA	21.03.00	Sauces & accompaniment - Other sauces, incl. brown sauce, soy sauce, ketchup, mint sauce, vinegar	12350010	Dip, NFS
3486	02-10245			SOYA ALTERNATIVE TO MILK, LIGHT, UNSWEETENED, FORTIFIED	05.04.00	Milk - Other - plant based, e.g. rice, soy	11320000	Soy milk
3487	02-08369			SOYA BEAN CURD / TOFU SMOKED	16.01.00	Pulses/Lentils - Pulses/lentils	41421010	Soybean curd, deep fried
3488	02-01376			SOYA MINCE AS MADE UP	16.01.00	Pulses/Lentils - Pulses/lentils	41421010	Soybean curd, deep fried
3489	02-09300			SPAGHETTI BOLOGNAISE LOW FAT READY MEAL	10.01.00	Meat - red - Beef & veal & dishes	58146322	Pasta with tomato-based sauce and meat, home recipe
3490	02-07602			SPAGHETTI, CANNED IN TOMATO SAUCE, REDUCED SUGAR	01.02.00	Cereals & cereal dishes - Pasta & pasta dishes	58146223	Pasta with tomato-based sauce, ready-to-heat
3491	02-05623			SPAM FRITTERS	12.01.00	Processed meat - Bacon & ham	77121110	Potato and ham fritters, Puerto Rican style
3492	02-01331			SPARE RIBS IN BARBECUE SAUCE NO BONES	10.03.00	Meat - red - Pork & dishes	22701030	Pork, spareribs, barbecued, with sauce, NS as to fat eaten
3493	02-01334			SPECIAL FRIED RICE	01.03.00	Cereals & cereal dishes - Rice & rice dishes	58150310	Rice, fried, NFS

3494	02-08013			SPECIAL K BERRIES ANY FRUIT ADDITION NOT CHOC OR YOGURT	02.03.00	Breakfast cereals - Other breakfast cereals - low fibre	57344001	Cereal (Kellogg's Special K Blueberry)
3495	02-08014			SPECIAL K BLISS WITH CHOC OR YOGURT PIECES	02.03.00	Breakfast cereals - Other breakfast cereals - low fibre	57344015	Cereal (Kellogg's Special K Fruit & Yogurt)
3496	02-10187			SPECIAL K CEREAL BARS, FRUIT WITH YOGURT TOPPING ONLY	04.05.00	Sweet cereal products - Cereal bars	53710700	Cereal or granola bar (Kellogg's Special K bar)
3497	02-10216			SPECIAL K MINI BREAKS	04.01.00	Sweet cereal products - Biscuits	53231400	Cookie, multigrain, high fiber
3498	02-02970			SPECIAL K WITH RED BERRIES	02.03.00	Breakfast cereals - Other breakfast cereals - low fibre	57344010	Cereal (Kellogg's Special K Red Berries)
3499	02-05856			SPICY RED KIDNEY BEANS	16.01.00	Pulses/Lentils - Pulses/lentils	41101000	Beans, dry, cooked, NS as to type and as to fat added in cooking
3500	02-08167			SPINACH AND POTATO CURRY PURCHASED OR TAKEAWAY	15.04.00	Vegetables - Other	75440600	Vegetable curry
3501	02-04023			SPINACH AND RICOTTA CANNELLONI, READY MEAL EG SAINSBURY'S	01.02.00	Cereals & cereal dishes - Pasta & pasta dishes	58134810	Cannelloni, cheese- and spinach-filled, no sauce
3502	02-09548			SPONGE CAKE MADE WITH BUTTER	04.02.00	Sweet cereal products - Pastries, Buns & Pies	53118100	Cake, sponge, without icing or filling
3503	02-00566			SPONGE PUDDING CANNED (ANY)	04.03.00	Sweet cereal products - Cereal based puddings (not milk)	53118100	Cake, sponge, without icing or filling
3504	02-01526			SPRATS FRIED IN BLENDED OIL	09.02.00	Fish & fish dishes - Oily fish	26139110	Sardines, cooked
3505	02-07871			SQUARE CRISPS	25.01.00	Savoury Snacks - Potato based snacks	71200010	Potato chips, NFS
3506	02-08104			SQUID BOILED	09.03.00	Fish & fish dishes - Shellfish	26213160	Squid, steamed or boiled
3507	02-04037			STARTING RIGHT CEREAL, ASDA	02.03.00	Breakfast cereals - Other breakfast cereals - low fibre	57100100	Cereal, ready-to-eat, NFS
3508	02-01381			STEAK & KIDNEY PUDD NOT CANNED	10.01.00	Meat - red - Beef & veal & dishes	27214300	Beef wellington

3509	02-01309			STEAK PIE PASTRY TOP ONLY	10.01.00	Meat - red - Beef & veal & dishes	27360050	Meat pie, NFS
3510	02-08808			STEAK PIE PUFF PASTRY 2 CRUSTS	12.02.00	Processed meat - Processed pies	27360050	Meat pie, NFS
3511	02-08087			STEAK PIE, SHORT CRUST, PURCHASED	10.01.00	Meat - red - Beef & veal & dishes	27360050	Meat pie, NFS
3512	02-10318			STICKY TOFFEE PUDDING PURCHASED	04.03.00	Sweet cereal products - Cereal based puddings (not milk)	13210150	Bread pudding made with evaporated milk and rum
3513	02-03982			STIR FRY SAUCE, CHINESE	21.02.00	Sauces & accompaniment - Cooking sauces, incl. gravies, pesto, cooking sauces for pasta and rice dishes	41420400	Teriyaki sauce
3514	02-08034			SUGAR / SWEETENER MIXES	26.03.00	Miscellaneous - Artificial sweeteners	91200000	Sugar substitute, powder, NFS
3515	02-03857			SUGAR CONFECTIONARY WITH ADDED CREAM EG WERTHERS ORIGINAL, CAMPINO SWEETS	24.02.00	Confectionary - Sugar based products	91702010	Butterscotch morsels
3516	02-08646			SUGAR FREE FRUIT GUMS EG. BOOTS	24.02.00	Confectionary - Sugar based products	91700010	Candy, NFS
3517	02-08857			SUGAR FREE INSTANT CUSTARD MADE UP	04.04.00	Sweet cereal products - Milk based puddings	13210300	Custard
3518	02-07968			SUGAR FREE MINTS	24.02.00	Confectionary - Sugar based products	91700010	Candy, NFS
3519	02-06832			SUMMERFRUIT COMPOTE (M&S)	18.01.00	Fruit - Fresh	63311140	Fruit cocktail, cooked or canned, in light syrup
3520	02-02853			SUPERMALT/MIGHTY MALT PREMIUM-NON ALCOHOLIC ENERGY MALT DRINK	27.01.06	Beverages - Alcohol - Low alcohol beer	93101000	Beer
3521	02-09153			SUSHI, SALMON BASED	09.02.00	Fish & fish dishes - Oily fish	58151420	Sushi, topped with salmon
3522	02-02820			SUSHI, TUNA BASED	09.01.00	Fish & fish dishes - White fish, incl. tuna	58151440	Sushi, topped with tuna

3523	02-02821			SUSHI, VEGETARIAN	15.04.00	Vegetables - Other	58151230	Sushi roll, vegetable
3524	02-06042			SWEET & SOUR SAUCE WITH PINEAPPLE	21.02.00	Sauces & accompaniment - Cooking sauces, incl. gravies, pesto, cooking sauces for pasta and rice dishes	91361010	Sweet and sour sauce
3525	02-02734			SWEET AND SOUR CHICKEN, LOW FAT, READY MEAL, E.G. ASDA HEALTHY CHOICE	11.01.00	Meat - white - Chicken & turkey & dishes	27146100	Sweet and sour chicken or turkey
3526	02-08074			SWEET CHILLI SAUCE DIPPING SAUCE	21.03.00	Sauces & accompaniment - Other sauces, incl. brown sauce, soy sauce, ketchup, mint sauce, vinegar	75511010	Hot pepper sauce
3527	00-05694			SWEETENERS, CALORIE FREE, TABLETS (per tablet)	26.03.00	Miscellaneous - Artificial sweeteners	91200000	Sugar substitute, powder, NFS
3528	A-17-0042	17-0042		Safflower oil	08.02.00	Fats - Oils	82106000	Safflower oil
3529	A-13-0852	13-0852		Saffron	26.01.00	Miscellaneous - Dried herbs & spices & pastes	2034	Saffron
3530	A-13-0854	13-0854		Sage, dried, ground	26.01.00	Miscellaneous - Dried herbs & spices & pastes	2038	Spices, sage, ground
3531	13-0853			Sage, fresh	19.00.00	Nuts & Seeds (incl. peanut butter)	75119000	Parsley, raw
3532	00-05412			Sainsbury's Malties Cereal	02.02.00	Breakfast cereals - Other breakfast cereals - high fibre (equal or >3g/40g portion)	57100100	Cereal, ready-to-eat, NFS
3533	00-05538			Sainsbury's Roulade Dessert Lemon	04.03.00	Sweet cereal products - Cereal based puddings (not milk)	53113000	Cake, jelly roll
3534	00-05557			Sainsbury's Toffee Pavlova	07.00.00	Egg & egg dishes	32401000	Meringues
3535	00-05552			Sainsburys Belgian Waffles	04.02.00	Sweet cereal products - Pastries, Buns & Pies	55200010	Waffle, NFS
3536	A-00-00472			Saithe, Steamed	09.01.00	Fish & fish dishes - White fish, incl. tuna	26109160	Cod, steamed or poached
3537	A-00-00930			Salad Cream	21.01.00	Sauces & accompaniment - Dressings & Mayonnaise	83100100	Salad dressing, NFS, for salads

3538	A-00-01155			Salad Cream, Low Calorie	21.01.00	Sauces & accompaniment - Dressings & Mayonnaise	83200100	Salad dressing, light, NFS
3539	00-05724			Salad Cream, reduced calorie e.g.Asda, Sains, Tesco, WW, Heinz extra light	21.01.00	Sauces & accompaniment - Dressings & Mayonnaise	83200100	Salad dressing, light, NFS
3540	A-00-06303			Salad composite 50	15.04.00	Vegetables - Other	75114000	Mixed salad greens, raw
3541	17-0326	A-12-0291		Salad cream	21.01.00	Sauces & accompaniment - Dressings & Mayonnaise	83100100	Salad dressing, NFS, for salads
3542	A-00-09965			Salad cream (MW6 Vit Eq)	21.01.00	Sauces & accompaniment - Dressings & Mayonnaise	83100100	Salad dressing, NFS, for salads
3543	A-17-0327	A-12-0292		Salad cream, reduced calorie	21.01.00	Sauces & accompaniment - Dressings & Mayonnaise	83200100	Salad dressing, light, NFS
3544	15-0290	A-15-0290		Salad, Florida, retail	15.04.00	Vegetables - Other	75141200	Cabbage salad or coleslaw with pineapple, with dressing
3545	A-15-0291	15-0291		Salad, Greek	15.04.00	Vegetables - Other	75146000	Greek Salad, no dressing
3546	15-0303	A-15-0303		Salad, Waldorf	15.04.00	Vegetables - Other	63401010	Apple salad with dressing
3547	A-15-0304	15-0304		Salad, Waldorf, retail	15.04.00	Vegetables - Other	63401010	Apple salad with dressing
3548	A-15-0288	15-0288		Salad, carrot and nut with French dressing, retail	15.04.00	Vegetables - Other	73101110	Carrots, raw, salad
3549	15-0292	A-15-0292		Salad, green	15.04.00	Vegetables - Other	75114000	Mixed salad greens, raw
3550	A-15-0293	15-0293		Salad, pasta	01.02.00	Cereals & cereal dishes - Pasta & pasta dishes	58148114	Macaroni or pasta salad, made with Italian dressing
3551	A-15-0295			Salad, potato, with French dressing	17.01.00	Potatoes - Potatoes	71603030	Potato salad, made with creamy dressing
3552	A-15-0296	15-0296		Salad, potato, with mayonnaise	17.01.00	Potatoes - Potatoes	71603010	Potato salad, made with mayonnaise
3553	15-0297	A-15-0297		Salad, potato, with mayonnaise, retail	17.01.00	Potatoes - Potatoes	71603010	Potato salad, made with mayonnaise

3554	15-0298	A-15-0298		Salad, potato, with reduced calorie dressing, retail	17.01.00	Potatoes - Potatoes	71603015	Potato salad, made with light mayonnaise
3555	A-15-0299	15-0299		Salad, rice	01.03.00	Cereals & cereal dishes - Rice & rice dishes	56205001	Rice, white, cooked, NS as to fat added in cooking
3556	A-15-0301	15-0301		Salad, tomato and onion	15.04.00	Vegetables - Other	74506000	Tomato and cucumber salad made with tomato, cucumber, oil, and vinegar
3557	A-15-0302	15-0302		Salad, vegetable, canned	15.04.00	Vegetables - Other	75302080	Bean salad, yellow and/or green string beans
3558	A-19-0110	A-00-00407	19-0110	Salami	12.03.00	Processed meat - Other processed meats	25221500	Salami, NFS
3559	16-0304	A-16-0304		Salmon en croute, retail	09.02.00	Fish & fish dishes - Oily fish	26137131	Salmon, coated, baked or broiled, made with butter
3560	00-03637			Salmon fishcakes from Carmel Moore's Study	09.02.00	Fish & fish dishes - Oily fish	27250070	Salmon cake or patty
3561	00-03646			Salmon mousse	09.02.00	Fish & fish dishes - Oily fish	27150050	Fish timbale or mousse
3562	00-03644			Salmon terrine or pate	09.02.00	Fish & fish dishes - Oily fish	27150050	Fish timbale or mousse
3563	A-00-00498			Salmon, Canned	09.02.00	Fish & fish dishes - Oily fish	26137180	Salmon, canned
3564	A-00-00495			Salmon, Raw	09.02.00	Fish & fish dishes - Oily fish	26137100	Salmon, raw
3565	A-00-00499			Salmon, Smoked	09.02.00	Fish & fish dishes - Oily fish	26137190	Salmon, smoked
3566	A-00-00496			Salmon, Steamed	09.02.00	Fish & fish dishes - Oily fish	26137160	Salmon, steamed or poached
3567	A-00-00497			Salmon, Steamed (Weighed with Bones and Skin)	09.02.00	Fish & fish dishes - Oily fish	26137160	Salmon, steamed or poached
3568	A-16-0203	16-0203		Salmon, grilled	09.02.00	Fish & fish dishes - Oily fish	26137123	Salmon, baked or broiled, made without fat
3569	16-0208			Salmon, pink, canned in brine, flesh only, drained	09.02.00	Fish & fish dishes - Oily fish	26137180	Salmon, canned

3570	A-00-09969			Salmon, pink, canned in brine, flesh only, drained (MW6 vit D)	09.02.00	Fish & fish dishes - Oily fish	26137180	Salmon, canned
3571	16-0210			Salmon, red, canned in brine, flesh only, drained	09.02.00	Fish & fish dishes - Oily fish	26137180	Salmon, canned
3572	A-16-0207	16-0207		Salmon, smoked	09.02.00	Fish & fish dishes - Oily fish	26137190	Salmon, smoked
3573	A-16-0205	16-0205		Salmon, steamed	09.02.00	Fish & fish dishes - Oily fish	26137160	Salmon, steamed or poached
3574	A-00-03924	00-03924		Salsa dip	21.03.00	Sauces & accompaniment - Other sauces, incl. brown sauce, soy sauce, ketchup, mint sauce, vinegar	74402100	Salsa, NFS
3575	17-0367			Salt	26.02.00	Miscellaneous - Salt and salt substitutes	2047	Salt, table
3576	A-00-00966			Salt, Table	26.02.00	Miscellaneous - Salt and salt substitutes	2047	Salt, table
3577	A-00-01239			Samoosa Curried Mince in Dough	10.02.00	Meat - red - Lamb & dishes	58121510	Dumpling, meat-filled
3578	00-09515	A-00-09515		Samosa, meat	10.02.00	Meat - red - Lamb & dishes	23132000	Lamb, ground or patty, cooked
3579	A-11-0353			Samosas, meat	10.02.00	Meat - red - Lamb & dishes	23132000	Lamb, ground or patty, cooked
3580	A-11-0354			Samosas, vegetable	15.04.00	Vegetables - Other	58121620	Dumpling, vegetable
3581	15-0305	A-15-0305		Samosas, vegetable, retail	15.04.00	Vegetables - Other	58121620	Dumpling, vegetable
3582	A-00-03798			Sandwich Spread 82/1440	21.01.00	Sauces & accompaniment - Dressings & Mayonnaise	83100200	Salad dressing, NFS, for sandwiches
3583	A-11-0182			Sandwich biscuits	04.01.00	Sweet cereal products - Biscuits	53238000	Cookie, sandwich-type, not chocolate or vanilla
3584	00-09602	A-00-09602		Sandwich biscuits (cream filled)	04.01.00	Sweet cereal products - Biscuits	53238000	Cookie, sandwich-type, not chocolate or vanilla
3585	A-00-06056			Sandwich biscuits 50	04.01.00	Sweet cereal products - Biscuits	53238000	Cookie, sandwich-type, not chocolate or vanilla
3586	A-17-0328			Sandwich spread	21.01.00	Sauces & accompaniment - Dressings & Mayonnaise	83100200	Salad dressing, NFS, for sandwiches

3587	A-00-00501			Sardines, Canned, Fish Plus Oil	09.02.00	Fish & fish dishes - Oily fish	26139180	Sardines, canned in oil
3588	A-00-00500			Sardines, Canned, in Oil, Fish Only	09.02.00	Fish & fish dishes - Oily fish	26139180	Sardines, canned in oil
3589	A-00-00502			Sardines, Canned, in Tomato Sauce	09.02.00	Fish & fish dishes - Oily fish	27150350	Sardines with tomato-based sauce
3590	A-16-0215	16-0215		Sardines, canned in brine, drained	09.02.00	Fish & fish dishes - Oily fish	26139190	Sardines, skinless, boneless, packed in water
3591	16-0216	A-16-0216		Sardines, canned in oil, drained	09.02.00	Fish & fish dishes - Oily fish	26139180	Sardines, canned in oil
3592	16-0217	A-16-0217		Sardines, canned in tomato sauce	09.02.00	Fish & fish dishes - Oily fish	27150350	Sardines with tomato-based sauce
3593	00-05820			Satay stir fry sauce eg Amoy	21.02.00	Sauces & accompaniment - Cooking sauces, incl. gravies, pesto, cooking sauces for pasta and rice dishes	41420400	Teriyaki sauce
3594	A-14-0257	14-0257		Satsumas	18.01.00	Fruit - Fresh	61119010	Orange, raw
3595	00-03640			Sauce stir-in eg Dolmio, Knorr	21.02.00	Sauces & accompaniment - Cooking sauces, incl. gravies, pesto, cooking sauces for pasta and rice dishes	6124	Gravy, pork, dry, powder
3596	00-05210	A-00-05210		Sauce, Soy, Light, Thin	21.03.00	Sauces & accompaniment - Other sauces, incl. brown sauce, soy sauce, ketchup, mint sauce, vinegar	41420300	Soy sauce
3597	17-0332			Sauce, dry mix, made up	21.02.00	Sauces & accompaniment - Cooking sauces, incl. gravies, pesto, cooking sauces for pasta and rice dishes	6116	Gravy, beef, canned, ready-to-serve
3598	17-0329			Sauce, dry, casserole mix	21.02.00	Sauces & accompaniment - Cooking sauces, incl. gravies, pesto, cooking sauces for pasta and rice dishes	6124	Gravy, pork, dry, powder

3599	17-0330			Sauce, dry, casserole mix, made up	21.02.00	Sauces & accompaniment - Cooking sauces, incl. gravies, pesto, cooking sauces for pasta and rice dishes	28500040	Gravy, beef or meat
3600	15-0310			Sauce, tomato base	21.02.00	Sauces & accompaniment - Cooking sauces, incl. gravies, pesto, cooking sauces for pasta and rice dishes	74404010	Spaghetti sauce
3601	17-0333	A-17-0333		Sauce, tomato base, homemade	21.02.00	Sauces & accompaniment - Cooking sauces, incl. gravies, pesto, cooking sauces for pasta and rice dishes	74404010	Spaghetti sauce
3602	A-00-06212			Sausage 50 pork fried	13.00.00	Sausages & burgers & kebab	25221405	Pork sausage
3603	A-00-00422			Sausage Roll, Flaky Pastry	12.02.00	Processed meat - Processed pies	27560350	Pig in a blanket, frankfurter or hot dog wrapped in dough
3604	A-00-00423			Sausage Roll, Short Pastry	12.02.00	Processed meat - Processed pies	27560350	Pig in a blanket, frankfurter or hot dog wrapped in dough
3605	A-00-06222			Sausage Roll, Short Pastry 50	13.00.00	Sausages & burgers & kebab	58124230	Pastry, meat / poultry-filled
3606	A-19-0269			Sausage casserole	13.00.00	Sausages & burgers & kebab	32105190	Egg casserole with bread, cheese, milk and meat
3607	19-0067	A-19-0067		Sausage rolls, flaky pastry, homemade	12.02.00	Processed meat - Processed pies	27560350	Pig in a blanket, frankfurter or hot dog wrapped in dough
3608	19-0068	A-19-0068		Sausage rolls, short pastry, homemade	12.02.00	Processed meat - Processed pies	27560350	Pig in a blanket, frankfurter or hot dog wrapped in dough
3609	A-00-01378			Sausage, beef or pork, fried or grilled	13.00.00	Sausages & burgers & kebab	25220105	Beef sausage
3610	A-00-00409			Sausages, Beef, Fried	13.00.00	Sausages & burgers & kebab	25220105	Beef sausage
3611	A-00-00410			Sausages, Beef, Grilled	13.00.00	Sausages & burgers & kebab	25220105	Beef sausage

3612	A-00-00408			Sausages, Beef, Raw	13.00.00	Sausages & burgers & kebab	23572	Beef, ground, 80% lean meat / 20% fat, raw
3613	A-00-00412			Sausages, Pork, Fried	13.00.00	Sausages & burgers & kebab	25221405	Pork sausage
3614	A-00-00413			Sausages, Pork, Grilled	13.00.00	Sausages & burgers & kebab	25221405	Pork sausage
3615	A-00-00411			Sausages, Pork, Raw	13.00.00	Sausages & burgers & kebab	10020	Pork, fresh, loin, whole, separable lean and fat, raw
3616	A-00-01395			Sausages, low fat	13.00.00	Sausages & burgers & kebab	25221406	Pork sausage, reduced fat
3617	A-00-00414			Saveloy	12.03.00	Processed meat - Other processed meats	25221400	Sausage, NFS
3618	19-0111			Saveloy, unbattered, takeaway	12.03.00	Processed meat - Other processed meats	25221400	Sausage, NFS
3619	A-00-01396			Savoury pancakes (Birds Eye), average	01.04.00	Cereals & cereal dishes - Other cereals & dishes	55100005	Pancakes, NFS
3620	11-0040	A-11-0040		Savoury rice, cooked	01.03.00	Cereals & cereal dishes - Rice & rice dishes	56205001	Rice, white, cooked, NS as to fat added in cooking
3621	A-00-00538			Scallops, Steamed	09.03.00	Fish & fish dishes - Shellfish	26317130	Scallops, steamed or boiled
3622	A-00-00525			Scampi, Fried	09.03.00	Fish & fish dishes - Shellfish	26319140	Shrimp, coated, fried, made with oil
3623	16-0243	A-16-0243		Scampi, in breadcrumbs, frozen, fried in blended oil	09.03.00	Fish & fish dishes - Shellfish	26319140	Shrimp, coated, fried, made with oil
3624	A-16-0244	16-0244		Scampi, in breadcrumbs, frozen, fried in sunflower oil	09.03.00	Fish & fish dishes - Shellfish	26319140	Shrimp, coated, fried, made with oil
3625	00-03651			Scampi, in crumbs, frozen, oven baked	09.03.00	Fish & fish dishes - Shellfish	26319163	Shrimp, coated, baked or broiled, made without fat
3626	A-00-06551			School 50 Meat	10.01.00	Meat - red - Beef & veal & dishes	21000100	Beef, NS as to cut, cooked, NS as to fat eaten

3627	A-00-06553			School 50 cabbage boiled source MW4	15.02.00	Vegetables - Brassicacea	75211010	Cabbage, green, cooked, NS as to fat added in cooking
3628	A-00-06555			School 50 custard source MW4	04.04.00	Sweet cereal products - Milk based puddings	13210300	Custard
3629	A-00-06554			School 50 gravy source 1160	21.02.00	Sauces & accompaniment - Cooking sauces, incl. gravies, pesto, cooking sauces for pasta and rice dishes	28500040	Gravy, beef or meat
3630	A-00-06558			School 50 milk source MW4	05.03.00	Milk - Whole milk	11100000	Milk, NFS
3631	A-00-06552			School 50 potatoes mashed source MW4	17.01.00	Potatoes - Potatoes	71501000	Potato, mashed, NFS
3632	A-00-06556			School 50 rhubarb stewed wuth sugar source MW4	18.02.00	Fruit - Canned & cooked	63147110	Rhubarb, cooked or canned, NS as to sweetened or unsweetened; sweetened, NS as to type of sweetener
3633	A-00-06557			School 50 sponge pudding source MW4	04.03.00	Sweet cereal products - Cereal based puddings (not milk)	53118100	Cake, sponge, without icing or filling
3634	00-05424			Schwartz dry mixes	21.02.00	Sauces & accompaniment - Cooking sauces, incl. gravies, pesto, cooking sauces for pasta and rice dishes	6124	Gravy, pork, dry, powder
3635	A-00-01044			Scone, Plain, Wholemeal	04.02.00	Sweet cereal products - Pastries, Buns & Pies	52105100	Scone
3636	A-00-00095			Scones	04.02.00	Sweet cereal products - Pastries, Buns & Pies	52105100	Scone
3637	11-0264	A-11-0264		Scones, cheese	01.04.00	Cereals & cereal dishes - Other cereals & dishes	52104100	Biscuit, cheese
3638	A-00-06077			Scones, cheese 50	04.02.00	Sweet cereal products - Pastries, Buns & Pies	52104100	Biscuit, cheese
3639	A-11-0265			Scones, fruit	04.02.00	Sweet cereal products - Pastries, Buns & Pies	52105200	Scone, with fruit

3640	A-00-06078			Scones, fruit 50	04.02.00	Sweet cereal products - Pastries, Buns & Pies	52105200	Scone, with fruit
3641	A-11-0266	11-0266		Scones, plain	01.04.00	Cereals & cereal dishes - Other cereals & dishes	52105100	Scone
3642	A-00-06040			Scones, plain 50	04.02.00	Sweet cereal products - Pastries, Buns & Pies	52105100	Scone
3643	11-0267	A-11-0267		Scones, potato	17.02.00	Potatoes - Potato products - other	51127010	Bread, potato
3644	00-09592	A-00-09592		Scones, white, fruit	04.02.00	Sweet cereal products - Pastries, Buns & Pies	52105200	Scone, with fruit
3645	A-11-0268	11-0268		Scones, wholemeal	01.04.00	Cereals & cereal dishes - Other cereals & dishes	52104040	Biscuit, whole wheat
3646	A-00-06079			Scones, wholemeal 50	04.02.00	Sweet cereal products - Pastries, Buns & Pies	52105100	Scone
3647	11-0269	A-11-0269		Scones, wholemeal, fruit	04.02.00	Sweet cereal products - Pastries, Buns & Pies	52105200	Scone, with fruit
3648	A-00-03786			Scotch Broth 82/1428	20.01.00	Soups - Canned & fresh & homemade	75656010	Vegetable soup, Spanish style, stew type
3649	A-00-00180			Scotch Egg	07.00.00	Egg & egg dishes	32202060	Egg and sausage on biscuit
3650	A-00-00096			Scotch Pancakes	04.02.00	Sweet cereal products - Pastries, Buns & Pies	55100005	Pancakes, NFS
3651	17-0277	A-17-0277		Scotch broth	20.01.00	Soups - Canned & fresh & homemade	75656010	Vegetable soup, Spanish style, stew type
3652	A-12-0824	12-0824		Scotch eggs, retail	07.00.00	Egg & egg dishes	32202060	Egg and sausage on biscuit
3653	A-00-03774			Scotch meat pie 82/1413	12.02.00	Processed meat - Processed pies	27360050	Meat pie, NFS
3654	A-11-0270			Scotch pancakes	04.02.00	Sweet cereal products - Pastries, Buns & Pies	55100005	Pancakes, NFS
3655	A-00-09590	00-09590		Scotch pancakes (drop scones)	04.02.00	Sweet cereal products - Pastries, Buns & Pies	55100005	Pancakes, NFS
3656	A-00-06041			Scotch pancakes 50	04.02.00	Sweet cereal products - Pastries, Buns & Pies	55100005	Pancakes, NFS

3657	16-0306	A-16-0306		Seafood cocktail	09.03.00	Fish & fish dishes - Shellfish	27150110	Shrimp cocktail
3658	00-03647			Seafood mousse (crab, prawn, etc)	09.03.00	Fish & fish dishes - Shellfish	27150050	Fish timbale or mousse
3659	A-00-00656			Seakale, Boiled	15.02.00	Vegetables - Brassicacea	72119200	Kale, cooked, NS as to form, NS as to fat added in cooking
3660	13-0340			Seaweed, nori, dried, raw	15.04.00	Vegetables - Other	75232000	Seaweed, dried
3661	A-00-09742	00-09742		Semi-skimmed milk, UHT	05.02.00	Milk - Semi-skimmed milk	11100000	Milk, NFS
3662	A-12-0008			Semi-skimmed milk, average	05.02.00	Milk - Semi-skimmed milk	11100000	Milk, NFS
3663	00-09790	A-00-09790		Semi-skimmed milk, pasteurised, ave	05.02.00	Milk - Semi-skimmed milk	11100000	Milk, NFS
3664	A-00-09797	00-09797		Semi-skimmed milk, sterilized	05.02.00	Milk - Semi-skimmed milk	11100000	Milk, NFS
3665	00-09788	A-00-09788		Semi-skimmed milk, summer (May-Oct)	05.02.00	Milk - Semi-skimmed milk	11100000	Milk, NFS
3666	00-09792	A-00-09792		Semi-skimmed milk, winter (Nov-April)	05.02.00	Milk - Semi-skimmed milk	11100000	Milk, NFS
3667	A-11-0183	11-0183		Semi-sweet biscuits	04.01.00	Sweet cereal products - Biscuits	53241510	Marie biscuit
3668	A-00-09949			Semi-sweet biscuits (MW6 Vit Eq)	04.01.00	Sweet cereal products - Biscuits	53241510	Marie biscuit
3669	A-00-00023			Semolina, Raw	01.04.00	Cereals & cereal dishes - Other cereals & dishes	20081	Wheat flour, white, all-purpose, enriched, bleached
3670	11-0024			Semolina, raw	01.04.00	Cereals & cereal dishes - Other cereals & dishes	20081	Wheat flour, white, all-purpose, enriched, bleached
3671	A-00-01179			Sesame Seeds	19.00.00	Nuts & Seeds (incl. peanut butter)	43103000	Sesame seeds
3672	A-00-03801			Sesame Seeds 82/1443	19.00.00	Nuts & Seeds (incl. peanut butter)	43103000	Sesame seeds
3673	00-03070			Sesame and poppy thins eg Tesco	25.04.00	Savoury Snacks - Savoury biscuits & crackers	54340100	Crackers, gluten free, plain
3674	A-17-0043	17-0043		Sesame oil	08.02.00	Fats - Oils	82107000	Sesame oil

3675	A-00-09529	00-09529		Sesame prawn toasts	09.03.00	Fish & fish dishes - Shellfish	27250450	Shrimp toast, fried
3676	A-14-0844	14-0844		Sesame seeds	19.00.00	Nuts & Seeds (incl. peanut butter)	43103000	Sesame seeds
3677	00-05522			Sesame snaps	04.01.00	Sweet cereal products - Biscuits	91742010	Sesame Crunch, Sahadi
3678	17-0217	A-17-0217	A-00-01154	Shandy	27.01.03	Beverages - Alcohol - Beer	93101000	Beer
3679	A-00-03379			Shape fromage frais (Low fat)	06.01.00	Dairy products - Cream & fromage frais	14201200	Cottage cheese, farmer's
3680	A-00-03382			Shape mousse, any fruit flavour	06.03.02	Dairy products - Yoghurt & drinking yoghurts, incl. buttermilk and probiotics - reduced or low fat products	13250100	Mousse, not chocolate
3681	A-00-00434			Shepherd's Pie	10.02.00	Meat - red - Lamb & dishes	27330010	Shepherd's pie with lamb
3682	A-19-0270			Shepherd's pie	10.02.00	Meat - red - Lamb & dishes	27330010	Shepherd's pie with lamb
3683	19-0270			Shepherd's pie, homemade	10.02.00	Meat - red - Lamb & dishes	27330010	Shepherd's pie with lamb
3684	A-15-0313			Shepherd's pie, vegetable, retail	10.02.00	Meat - red - Lamb & dishes	27330010	Shepherd's pie with lamb
3685	A-00-06216			Shepherds pie 50	10.02.00	Meat - red - Lamb & dishes	27330010	Shepherd's pie with lamb
3686	A-17-0119	17-0119		Sherbert sweets	24.02.00	Confectionary - Sugar based products	91700010	Candy, NFS
3687	A-00-00911			Sherry, Dry	27.01.02	Beverages - Alcohol - Fortified wine	93402000	Wine, dessert, sweet
3688	A-00-00912			Sherry, Medium	27.01.02	Beverages - Alcohol - Fortified wine	93402000	Wine, dessert, sweet
3689	A-00-00913			Sherry, Sweet	27.01.02	Beverages - Alcohol - Fortified wine	93402000	Wine, dessert, sweet
3690	17-0235	A-17-0235		Sherry, dry	27.01.02	Beverages - Alcohol - Fortified wine	93402000	Wine, dessert, sweet
3691	17-0236	A-17-0236		Sherry, medium including Spanish, British, Cyprus, own label	27.01.02	Beverages - Alcohol - Fortified wine	93402000	Wine, dessert, sweet
3692	17-0237	A-17-0237		Sherry, sweet	27.01.02	Beverages - Alcohol - Fortified wine	93402000	Wine, dessert, sweet

3693	19-0151			Shish kebab in pitta bread with salad	13.00.00	Sausages & burgers & kebab	27516010	Gyro sandwich (pita bread, beef, lamb, onion, condiments), with tomato and spread
3694	19-0150			Shish kebab, meat only	13.00.00	Sausages & burgers & kebab	23120100	Lamb, roast, cooked, NS as to fat eaten
3695	11-0184	A-11-0184		Short-sweet biscuits	04.01.00	Sweet cereal products - Biscuits	53239000	Cookie, shortbread
3696	A-00-09950			Short-sweet biscuits (MW6 Vit Eq)	04.01.00	Sweet cereal products - Biscuits	53239000	Cookie, shortbread
3697	00-09604	A-00-09604	A-11-0185	Shortbread	04.02.00	Sweet cereal products - Pastries, Buns & Pies	53239000	Cookie, shortbread
3698	A-00-06019			Shortbread 50	04.01.00	Sweet cereal products - Biscuits	53239000	Cookie, shortbread
3699	00-03164			Shortbread fingers SAINSBURYS (RISCK-LA)	04.02.00	Sweet cereal products - Pastries, Buns & Pies	53239000	Cookie, shortbread
3700	A-00-03821			Shortcrust pastry U/R 82/1919	01.04.00	Cereals & cereal dishes - Other cereals & dishes	18335	Pie crust, standard-type, frozen, ready-to-bake, enriched, baked
3701	A-11-0226	11-0226		Shortcrust pastry, cooked	01.04.00	Cereals & cereal dishes - Other cereals & dishes	18335	Pie crust, standard-type, frozen, ready-to-bake, enriched, baked
3702	A-00-06031			Shortcrust pastry, cooked 50	04.02.00	Sweet cereal products - Pastries, Buns & Pies	53239000	Cookie, shortbread
3703	A-00-00054	A-11-0148		Shredded Wheat	02.02.00	Breakfast cereals - Other breakfast cereals - high fibre (equal or >3g/40g portion)	57417000	Cereal (Post Shredded Wheat)
3704	A-00-06004			Shredded Wheat 50	02.03.00	Breakfast cereals - Other breakfast cereals - low fibre	57417000	Cereal (Post Shredded Wheat)
3705	00-03045	A-00-03045		Shredded Wheat, Nestle	02.02.00	Breakfast cereals - Other breakfast cereals - high fibre (equal or >3g/40g portion)	57417000	Cereal (Post Shredded Wheat)
3706	A-11-0149			Shreddies	02.02.00	Breakfast cereals - Other breakfast cereals - high fibre (equal or >3g/40g portion)	57411000	Cereal (General Mills Chex Wheat)

3707	A-00-03046	00-03046		Shreddies, Nestle	02.02.00	Breakfast cereals - Other breakfast cereals - high fibre (equal or >3g/40g portion)	57411000	Cereal (General Mills Chex Wheat)
3708	A-00-01254			Shreddies, Malted	02.03.00	Breakfast cereals - Other breakfast cereals - low fibre	57100100	Cereal, ready-to-eat, NFS
3709	A-00-00527			Shrimps, Boiled	09.03.00	Fish & fish dishes - Shellfish	26319130	Shrimp, steamed or boiled
3710	A-00-00528			Shrimps, Boiled (Weighed with Shell)	09.03.00	Fish & fish dishes - Shellfish	26319130	Shrimp, steamed or boiled
3711	A-00-00529			Shrimps, Canned	09.03.00	Fish & fish dishes - Shellfish	26319180	Shrimp, canned
3712	A-16-0247	16-0247		Shrimps, canned in brine, drained	09.03.00	Fish & fish dishes - Shellfish	26319180	Shrimp, canned
3713	A-16-0248	16-0248		Shrimps, frozen	09.03.00	Fish & fish dishes - Shellfish	26319110	Shrimp, cooked, NS as to cooking method
3714	A-00-00515			Skate, Fried (Weighed with Waste)	09.01.00	Fish & fish dishes - White fish, incl. tuna	26135120	Ray, baked or broiled, fat added in cooking
3715	16-0144			Skate, grilled	09.01.00	Fish & fish dishes - White fish, incl. tuna	26135121	Ray, baked or broiled, fat not added in cooking
3716	16-0150	A-16-0150		Skate, in batter, fried in retail blend oil	09.01.00	Fish & fish dishes - White fish, incl. tuna	26135140	Ray, coated, fried
3717	A-16-0143			Skate, raw	09.01.00	Fish & fish dishes - White fish, incl. tuna	15095	Ray, raw
3718	A-00-03769			Skim Milk Powder + veg fat (5 pints) 82/1408	05.01.00	Milk - Skimmed milk	11810000	Milk, dry, not reconstituted, NS as to fat content
3719	A-00-09741	00-09741		Skimmed milk, UHT	05.01.00	Milk - Skimmed milk	11100000	Milk, NFS
3720	A-12-0001			Skimmed milk, average	05.01.00	Milk - Skimmed milk	11100000	Milk, NFS
3721	00-09783	A-00-09783		Skimmed milk, pasteurised, average	05.01.00	Milk - Skimmed milk	11100000	Milk, NFS
3722	00-09785	A-00-09785		Skimmed milk, pasteurised, summer	05.01.00	Milk - Skimmed milk	11100000	Milk, NFS
3723	00-09784	A-00-09784		Skimmed milk, pasteurised, winter	05.01.00	Milk - Skimmed milk	11100000	Milk, NFS
3724	A-00-09744	00-09744		Skimmed milk, sterilized	05.01.00	Milk - Skimmed milk	11100000	Milk, NFS

3725	00-05885			Slimfast Meal Replacement Shakes, RTD	30.00.00	Nutrition Powders & drinks	95110000	Nutritional drink or shake, ready-to-drink (Slim Fast)
3726	00-05889			Slimfast Meal Replacement Shakes, RTD Banana ONLY	30.00.00	Nutrition Powders & drinks	95110000	Nutritional drink or shake, ready-to-drink (Slim Fast)
3727	17-0096	A-17-0096		Smartie-type sweets	24.01.00	Confectionary - Chocolate based products	91746100	M&M's Milk Chocolate Candies
3728	00-05300			Smoothie, Red, 100% Fruit, not with coconut	27.02.01	Beverages - Fruit based drinks - Pure fruit juice & smoothies	64134015	Fruit smoothie, with whole fruit, no dairy
3729	00-04382			Smoothies, Yellow, 100% fruit, not with coconut	27.02.01	Beverages - Fruit based drinks - Pure fruit juice & smoothies	64134015	Fruit smoothie, with whole fruit, no dairy
3730	00-05775			Smoothies, containing coconut, Yellow, 100% fruit	27.02.01	Beverages - Fruit based drinks - Pure fruit juice & smoothies	64134015	Fruit smoothie, with whole fruit, no dairy
3731	00-04236			Snack hard cheese	06.02.00	Dairy products - Cheese, incl. cottage cheese	14102010	Cheese, Brick
3732	00-03930			Snack-a-Jacks, savoury, Quaker	25.02.00	Savoury Snacks - Cereal based snacks	54318500	Rice cake
3733	00-03931			Snack-a-Jacks, sweet, Quaker	04.01.00	Sweet cereal products - Biscuits	54318500	Rice cake
3734	17-0097	A-17-0097		Snickers	24.01.00	Confectionary - Chocolate based products	91715100	SNICKERS Bar
3735	A-11-0092	11-0092		Soda bread	03.04.00	Breads - Other bread	52408000	Bread, Irish soda
3736	A-00-09581	00-09581		Soda bread, brown	03.04.00	Breads - Other bread	52408000	Bread, Irish soda
3737	17-0182			Soda, club	27.03.00	Beverages - Carbonated soft drinks	92400000	Soft drink, NFS
3738	A-17-0183			Soda, cream	27.03.00	Beverages - Carbonated soft drinks	92400000	Soft drink, NFS
3739	A-00-01139			Soft Sugar Sweets, Fondants	24.02.00	Confectionary - Sugar based products	91726000	Nougat, plain
3740	A-00-01394			Soft grain white bread	03.01.00	Breads - White	51101000	Bread, white
3741	A-00-03738			Solid Vegetable Oil 82/979	08.04.01	Fats - Plant based fats (solid) - Full fat	81102000	Margarine, NFS

3742	00-05742			Something Xtra Cereal bars, yogurt coated, fortifed NOT Mixed berry	04.05.00	Sweet cereal products - Cereal bars	53710902	Cereal or granola bar, with yogurt coating (General Mills Nature Valley Chewy Granola Bar)
3743	A-00-09640	00-09640		Sorbet, fruit	24.03.00	Confectionary - Sorbets & lollies	63430100	Sorbet, fruit, noncitrus flavor
3744	A-12-0216			Sorbet, lemon	24.03.00	Confectionary - Sorbets & lollies	63430110	Sorbet, fruit, citrus flavor
3745	12-0827	A-12-0827		Souffle, cheese	07.00.00	Egg & egg dishes	14630200	Cheese souffle
3746	A-12-0826	12-0826		Souffle, plain	07.00.00	Egg & egg dishes	32129990	Egg omelet or scrambled egg, NS as to fat added in cooking
3747	00-03934			Soup Cream of Asparagus (eg Baxters canned)	20.01.00	Soups - Canned & fresh & homemade	75601000	Asparagus soup, cream of, NS as to made with milk or water
3748	A-00-03785			Soup, low calorie, canned, average 82/1427	20.01.00	Soups - Canned & fresh & homemade	58400000	Soup, NFS
3749	A-00-01156			Soups Diluted with Milk	20.01.00	Soups - Canned & fresh & homemade	75600150	Soup, cream of, NFS
3750	17-0334	A-17-0334		Soy sauce	21.03.00	Sauces & accompaniment - Other sauces, incl. brown sauce, soy sauce, ketchup, mint sauce, vinegar	41420300	Soy sauce
3751	A-00-05136			Soya Bean Curd/Tofu, Steamed	15.04.00	Vegetables - Other	41420010	Soybean curd
3752	A-00-00024			Soya Flour, Full Fat	16.01.00	Pulses/Lentils - Pulses/lentils	16115	Soy flour, full-fat, raw
3753	A-00-00025			Soya Flour, Low Fat	01.04.00	Cereals & cereal dishes - Other cereals & dishes	16115	Soy flour, full-fat, raw
3754	A-00-09653			Soya Yogurt, fruit	06.03.01	Dairy products - Yoghurt & drinking yoghurts, incl. buttermilk and probiotics - full fat products	41420380	Yogurt, soy

3755	00-03441			Soya based Soft Margarine e.g. BlueBand	08.04.01	Fats - Plant based fats (solid) - Full fat	81102000	Margarine, NFS
3756	13-0116	A-13-0116		Soya beans, dried, boiled in unsalted water	16.01.00	Pulses/Lentils - Pulses/lentils	41101000	Beans, dry, cooked, NS as to type and as to fat added in cooking
3757	13-0115			Soya beans, dried, raw	16.01.00	Pulses/Lentils - Pulses/lentils	16069	Lentils, raw
3758	11-0025			Soya flour, full fat	16.01.00	Pulses/Lentils - Pulses/lentils	16115	Soy flour, full-fat, raw
3759	A-12-0043			Soya milk, flavoured	05.04.00	Milk - Other - plant based, e.g. rice, soy	11320000	Soy milk
3760	00-05872			Soya milk, flavoured, unfortified eg Provamel	05.04.00	Milk - Other - plant based, e.g. rice, soy	11320000	Soy milk
3761	00-09751	A-00-09751	A-12-0042	Soya milk, plain	05.04.00	Milk - Other - plant based, e.g. rice, soy	11320000	Soy milk
3762	00-05896			Soya milk, sweetened, fortified, organic	05.04.00	Milk - Other - plant based, e.g. rice, soy	11320000	Soy milk
3763	00-09752	A-00-09752		Soya milk, sweetned, + calcium	05.04.00	Milk - Other - plant based, e.g. rice, soy	11320000	Soy milk
3764	00-05871			Soya milk, unsweetened, fortified eg Alpro	05.04.00	Milk - Other - plant based, e.g. rice, soy	11320000	Soy milk
3765	15-0314			Soya mince, granules	16.01.00	Pulses/Lentils - Pulses/lentils	41440000	Textured vegetable protein, dry
3766	A-17-0044			Soya oil	08.02.00	Fats - Oils	82108000	Soybean oil
3767	A-12-0196			Soya yogurt	06.03.01	Dairy products - Yoghurt & drinking yoghurts, incl. buttermilk and probiotics - full fat products	41420380	Yogurt, soy
3768	00-05899			Soya yogurt, plain eg Alpro	06.03.01	Dairy products - Yoghurt & drinking yoghurts, incl. buttermilk and probiotics - full fat products	41420380	Yogurt, soy
3769	A-00-09773			Soya, dessert topping	06.01.00	Dairy products - Cream & fromage frais	12210520	Coffee creamer, soy, liquid
3770	A-00-00205			Soyabean Oil	08.02.00	Fats - Oils	82108000	Soybean oil

3771	A-19-0273	19-0273		Spaghetti bolognese, chilled/frozen, reheated	10.01.00	Meat - red - Beef & veal & dishes	58146322	Pasta with tomato-based sauce and meat, home recipe
3772	A-00-00027			Spaghetti, Boiled	01.02.00	Cereals & cereal dishes - Pasta & pasta dishes	56130000	Pasta, cooked
3773	A-00-00028			Spaghetti, Canned in Tomato Sauce	01.02.00	Cereals & cereal dishes - Pasta & pasta dishes	58146223	Pasta with tomato-based sauce, ready-to-heat
3774	A-00-00026			Spaghetti, Raw	01.02.00	Cereals & cereal dishes - Pasta & pasta dishes	20120	Pasta, dry, enriched
3775	11-0356	A-11-0356		Spaghetti, canned in bolognese sauce	01.02.00	Cereals & cereal dishes - Pasta & pasta dishes	58146323	Pasta with tomato-based sauce and meat, ready-to-heat
3776	A-11-0357	11-0357		Spaghetti, canned in tomato sauce	01.02.00	Cereals & cereal dishes - Pasta & pasta dishes	58146223	Pasta with tomato-based sauce, ready-to-heat
3777	11-0062	A-11-0062		Spaghetti, white, boiled	01.02.00	Cereals & cereal dishes - Pasta & pasta dishes	56130000	Pasta, cooked
3778	A-00-09933			Spaghetti, white, boiled (MW6 folate)	01.02.00	Cereals & cereal dishes - Pasta & pasta dishes	56130000	Pasta, cooked
3779	A-11-0064	11-0064		Spaghetti, wholemeal, boiled	01.02.00	Cereals & cereal dishes - Pasta & pasta dishes	56132990	Pasta, whole grain, cooked
3780	A-00-00055	A-11-0150		Special K	02.03.00	Breakfast cereals - Other breakfast cereals - low fibre	57344000	Cereal (Kellogg's Special K)
3781	00-03047	A-00-03047		Special K, Kelloggs	02.03.00	Breakfast cereals - Other breakfast cereals - low fibre	57344000	Cereal (Kellogg's Special K)
3782	00-03552			Spinach pasta salad, Tesco or M&S	01.02.00	Cereals & cereal dishes - Pasta & pasta dishes	58148114	Macaroni or pasta salad, made with Italian dressing
3783	A-00-00657			Spinach, Boiled	15.03.00	Vegetables - Yellow & red & dark green leafy vegetables	72125211	Spinach, cooked, from fresh, fat not added in cooking
3784	13-0345			Spinach, boiled in unsalted water	15.03.00	Vegetables - Yellow & red & dark green leafy vegetables	72125211	Spinach, cooked, from fresh, fat not added in cooking

3785	A-00-09913			Spinach, boiled in unsalted water (MW6 carq)	15.03.00	Vegetables - Yellow & red & dark green leafy vegetables	72125211	Spinach, cooked, from fresh, fat not added in cooking
3786	13-0346			Spinach, frozen, boiled in unsalted water	15.03.00	Vegetables - Yellow & red & dark green leafy vegetables	72125202	Spinach, cooked, from frozen, NS as to fat added in cooking
3787	A-00-09914			Spinach, frozen, boiled in unsalted water (MW6 carq; MW6 folate)	15.03.00	Vegetables - Yellow & red & dark green leafy vegetables	72125202	Spinach, cooked, from frozen, NS as to fat added in cooking
3788	13-0343			Spinach, raw	15.03.00	Vegetables - Yellow & red & dark green leafy vegetables	72125100	Spinach, raw
3789	A-00-09912			Spinach, raw (MW6 folate)	15.03.00	Vegetables - Yellow & red & dark green leafy vegetables	72125100	Spinach, raw
3790	00-05893			Spirits with cooking losses	27.01.04	Beverages - Alcohol - Spirits & Liqueur	93501000	Brandy
3791	A-17-0246	17-0246		Spirits, 37.5% volume	27.01.04	Beverages - Alcohol - Spirits & Liqueur	93501000	Brandy
3792	17-0247	A-17-0247		Spirits, 40% volume	27.01.04	Beverages - Alcohol - Spirits & Liqueur	93501000	Brandy
3793	A-00-00919			Spirits, 70% Proof	27.01.04	Beverages - Alcohol - Spirits & Liqueur	93501000	Brandy
3794	13-0142	A-13-0142		Split peas, dried, boiled in unsalted water	16.01.00	Pulses/Lentils - Pulses/lentils	41101000	Beans, dry, cooked, NS as to type and as to fat added in cooking
3795	A-00-00118			Sponge Pudding, Steamed	04.03.00	Sweet cereal products - Cereal based puddings (not milk)	53118100	Cake, sponge, without icing or filling
3796	11-0211	A-11-0211		Sponge cake	04.02.00	Sweet cereal products - Pastries, Buns & Pies	53118100	Cake, sponge, without icing or filling
3797	A-11-0212			Sponge cake, fatless	04.02.00	Sweet cereal products - Pastries, Buns & Pies	53118100	Cake, sponge, without icing or filling
3798	A-11-0215			Sponge cake, frozen	04.02.00	Sweet cereal products - Pastries, Buns & Pies	53118100	Cake, sponge, without icing or filling

3799	00-05534			Sponge cake, jam & buttercream filling, purchased	04.02.00	Sweet cereal products - Pastries, Buns & Pies	53123070	Cake, shortcake, sponge type, with whipped cream and fruit
3800	00-09625	A-00-09625		Sponge cake, jam and cream	04.02.00	Sweet cereal products - Pastries, Buns & Pies	53123070	Cake, shortcake, sponge type, with whipped cream and fruit
3801	11-0213	A-11-0213		Sponge cake, jam filled	04.02.00	Sweet cereal products - Pastries, Buns & Pies	53123080	Cake, shortcake, sponge type, with fruit
3802	A-00-06028			Sponge cake, jam filled 50	04.02.00	Sweet cereal products - Pastries, Buns & Pies	53123080	Cake, shortcake, sponge type, with fruit
3803	11-0212			Sponge cake, low fat	04.02.00	Sweet cereal products - Pastries, Buns & Pies	53118100	Cake, sponge, without icing or filling
3804	11-0214	A-11-0214		Sponge cake, with butter icing	04.02.00	Sweet cereal products - Pastries, Buns & Pies	53118200	Cake, sponge, with icing or filling
3805	A-00-06029			Sponge cake, with butter icing 50	04.02.00	Sweet cereal products - Pastries, Buns & Pies	53118200	Cake, sponge, with icing or filling
3806	A-00-03129	00-03129		Sponge fingers (boudoir biscuits)	04.01.00	Sweet cereal products - Biscuits	53224000	Cookie, ladyfinger
3807	A-11-0325	11-0325		Sponge pudding	04.02.00	Sweet cereal products - Pastries, Buns & Pies	53118100	Cake, sponge, without icing or filling
3808	A-00-06046			Sponge pudding 50	04.03.00	Sweet cereal products - Cereal based puddings (not milk)	53118100	Cake, sponge, without icing or filling
3809	A-11-0328	11-0328		Sponge pudding, canned	04.02.00	Sweet cereal products - Pastries, Buns & Pies	53118100	Cake, sponge, without icing or filling
3810	11-0326	A-11-0326		Sponge pudding, with dried fruit	04.02.00	Sweet cereal products - Pastries, Buns & Pies	53110000	Cake, fruit cake, light or dark, holiday type cake
3811	A-00-06047			Sponge pudding, with dried fruit 50	04.03.00	Sweet cereal products - Cereal based puddings (not milk)	53110000	Cake, fruit cake, light or dark, holiday type cake
3812	A-11-0327	11-0327		Sponge pudding, with jam or treacle	04.02.00	Sweet cereal products - Pastries, Buns & Pies	53113000	Cake, jelly roll
3813	A-00-06048			Sponge pudding, with jam or treacle 50	04.03.00	Sweet cereal products - Cereal based puddings (not milk)	53113000	Cake, jelly roll
3814	A-00-00083			Spongecake, Jam Filled	04.02.00	Sweet cereal products - Pastries, Buns & Pies	53123080	Cake, shortcake, sponge type, with fruit

Diet quality and cognitive ability, Cara et al.

Crosswalk linking food codes from the UK National Survey of Health and Development with the USDA Food Patterns Equivalents/Ingredients Databases

3815	00-03154			Spongecake, fatfree (Tesco healty eating)	04.02.00	Sweet cereal products - Pastries, Buns & Pies	53118100	Cake, sponge, without icing or filling
3816	A-00-00081			Spongecake, with Fat	04.02.00	Sweet cereal products - Pastries, Buns & Pies	53118100	Cake, sponge, without icing or filling
3817	A-00-00082			Spongecake, without Fat	04.02.00	Sweet cereal products - Pastries, Buns & Pies	53118100	Cake, sponge, without icing or filling
3818	11-0329	A-11-0329		Spotted dick	04.03.00	Sweet cereal products - Cereal based puddings (not milk)	53110000	Cake, fruit cake, light or dark, holiday type cake
3819	A-16-0219			Sprats, fried	09.02.00	Fish & fish dishes - Oily fish	26139110	Sardines, cooked
3820	00-05658			Spreadable butter, light, 60% fat eg Lurpak Lighter Spreadable	08.01.00	Fats - Butter	81100500	Butter, NFS
3821	A-00-00658			Spring Greens, Boiled	15.03.00	Vegetables - Yellow & red & dark green leafy vegetables	72118200	Greens, cooked, NS as to form, NS as to fat added in cooking
3822	13-0350	A-13-0350		Spring greens, boiled in unsalted water	15.02.00	Vegetables - Brassicacea	72118200	Greens, cooked, NS as to form, NS as to fat added in cooking
3823	13-0352	A-13-0352		Spring onions, bulbs and tops, raw	15.04.00	Vegetables - Other	75117010	Onions, young green, raw
3824	16-0265	A-16-0265		Squid, in batter, fried in blended oil	09.03.00	Fish & fish dishes - Shellfish	26213140	Squid, coated, fried
3825	A-16-0263			Squid, raw	09.03.00	Fish & fish dishes - Shellfish	26213100	Squid, raw
3826	A-00-03732			St Ivel Gold (40% fat) 82/970	08.04.03	Fats - Plant based fats (solid) - Low fat	81102000	Margarine, NFS
3827	A-11-0151			Start	02.03.00	Breakfast cereals - Other breakfast cereals - low fibre	57100100	Cereal, ready-to-eat, NFS
3828	00-03048	A-00-03048		Start, Kelloggs	02.03.00	Breakfast cereals - Other breakfast cereals - low fibre	57100100	Cereal, ready-to-eat, NFS
3829	A-00-00425			Steak and Kidney Pie, Individual	12.02.00	Processed meat - Processed pies	27360050	Meat pie, NFS
3830	A-00-00424			Steak and Kidney Pie, Pastry on Top Only	12.02.00	Processed meat - Processed pies	27360050	Meat pie, NFS

3831	A-00-06221			Steak and Kidney Pie, Pastry on top only 50	13.00.00	Sausages & burgers & kebab	27214300	Beef wellington
3832	A-00-01096			Steak and Kidney Pudding	10.01.00	Meat - red - Beef & veal & dishes	27214300	Beef wellington
3833	A-00-01078			Steak and Kidney, Stewed with Gravy	10.01.00	Meat - red - Beef & veal & dishes	27112000	Beef with gravy
3834	00-01079	A-00-01079		Steak and Kidney, Stewed with Gravy and Onion	10.01.00	Meat - red - Beef & veal & dishes	27112000	Beef with gravy
3835	19-0071	A-19-0071		Steak and kidney pie, double crust, homemade	10.01.00	Meat - red - Beef & veal & dishes	27214300	Beef wellington
3836	A-19-0070	19-0070		Steak and kidney pie, single crust, homemade	12.02.00	Processed meat - Processed pies	27360050	Meat pie, NFS
3837	A-19-0072	19-0072		Steak and kidney pudding, canned	10.01.00	Meat - red - Beef & veal & dishes	27214300	Beef wellington
3838	19-0069	A-19-0069		Steak and kidney/Beef pie, individual, chilled/frozen, baked	12.02.00	Processed meat - Processed pies	27360050	Meat pie, NFS
3839	A-00-00397			Stewed Steak with Gravy, Canned	10.01.00	Meat - red - Beef & veal & dishes	27112000	Beef with gravy
3840	19-0152	A-19-0152		Stewed steak with gravy, canned	10.01.00	Meat - red - Beef & veal & dishes	27112000	Beef with gravy
3841	A-18-0186	18-0186		Stewing lamb, stewed, lean	10.02.00	Meat - red - Lamb & dishes	23120120	Lamb, roast, cooked, lean only eaten
3842	A-18-0187	18-0187		Stewing lamb, stewed, lean & fat	10.02.00	Meat - red - Lamb & dishes	23120110	Lamb, roast, cooked, lean and fat eaten
3843	17-0368	A-17-0368		Stock cubes, beef including Bovril, Oxo and own brands	26.01.00	Miscellaneous - Dried herbs & spices & pastes	6981	Soup, bouillon cubes and granules, low sodium, dry
3844	00-05815			Stock cubes, beef, Oxo, Asda and Bovril ONLY	26.01.00	Miscellaneous - Dried herbs & spices & pastes	6981	Soup, bouillon cubes and granules, low sodium, dry
3845	00-05814			Stock cubes, beef, eg Knorr, Tesco, Kallo NOT OXO, ASDA	26.01.00	Miscellaneous - Dried herbs & spices & pastes	6981	Soup, bouillon cubes and granules, low sodium, dry

3846	00-05813			Stock cubes, chicken OXO and ASDA ONLY	26.01.00	Miscellaneous - Dried herbs & spices & pastes	6981	Soup, bouillon cubes and granules, low sodium, dry
3847	00-05812			Stock cubes, chicken eg Knorr, Tesco, Kallo NOT Oxo, Asda	26.01.00	Miscellaneous - Dried herbs & spices & pastes	6981	Soup, bouillon cubes and granules, low sodium, dry
3848	17-0369			Stock cubes, chicken, including Oxo	26.01.00	Miscellaneous - Dried herbs & spices & pastes	6981	Soup, bouillon cubes and granules, low sodium, dry
3849	00-05905			Stock cubes, lamb, OXO ONLY	26.01.00	Miscellaneous - Dried herbs & spices & pastes	6981	Soup, bouillon cubes and granules, low sodium, dry
3850	00-05901			Stock cubes, pork, Knorr ONLY	26.01.00	Miscellaneous - Dried herbs & spices & pastes	6981	Soup, bouillon cubes and granules, low sodium, dry
3851	00-05817			Stock cubes, vegetable, OXO and Asda ONLY	26.01.00	Miscellaneous - Dried herbs & spices & pastes	6981	Soup, bouillon cubes and granules, low sodium, dry
3852	00-05816			Stock cubes, vegetable, eg Knorr, Tesco, Kallo NOT OXO, Asda	26.01.00	Miscellaneous - Dried herbs & spices & pastes	6981	Soup, bouillon cubes and granules, low sodium, dry
3853	17-0370	A-17-0370		Stock cubes, vegetable, including Oxo	26.01.00	Miscellaneous - Dried herbs & spices & pastes	6981	Soup, bouillon cubes and granules, low sodium, dry
3854	00-05576			Stonebaked garlic flatbread/pizza	01.01.00	Cereals & cereal dishes - Pizza	51108010	Focaccia, Italian flatbread, plain
3855	A-00-00898			Stout, Bottled	27.01.03	Beverages - Alcohol - Beer	93101000	Beer
3856	A-00-00899			Stout, Extra	27.01.03	Beverages - Alcohol - Beer	93101000	Beer
3857	A-17-0219	17-0219		Stout, Guinness	27.01.03	Beverages - Alcohol - Beer	93101000	Beer
3858	A-00-03788			Stovies (potato, onion, gravy) 82/1430	17.01.00	Potatoes - Potatoes	71501012	Potato, mashed, from fresh, made with milk, with gravy

3859	A-00-00818			Strawberries, Canned	18.02.00	Fruit - Canned & cooked	63223110	Strawberries, cooked or canned, NS as to sweetened or unsweetened; sweetened, NS as to type of sweetener
3860	A-00-00817			Strawberries, Raw	18.01.00	Fruit - Fresh	63223020	Strawberries, raw
3861	A-14-0262	14-0262		Strawberries, canned in syrup	18.02.00	Fruit - Canned & cooked	63223110	Strawberries, cooked or canned, NS as to sweetened or unsweetened; sweetened, NS as to type of sweetener
3862	A-14-0261	14-0261		Strawberries, frozen	18.01.00	Fruit - Fresh	63223610	Strawberries, frozen, unsweetened
3863	14-0260	A-14-0260		Strawberries, raw	18.01.00	Fruit - Fresh	63223020	Strawberries, raw
3864	00-05356			Strawberry Ribena	27.02.02	Beverages - Fruit based drinks - Fruit juice drinks	92531030	Fruit juice drink (Sunny D)
3865	11-0272	A-11-0272		Strawberry tartlets	04.02.00	Sweet cereal products - Pastries, Buns & Pies	53314000	Pie, strawberry, individual size or tart
3866	A-00-06080			Strawberry tartlets 50	04.02.00	Sweet cereal products - Pastries, Buns & Pies	53314000	Pie, strawberry, individual size or tart
3867	A-00-00900			Strong Ale	27.01.03	Beverages - Alcohol - Beer	93101000	Beer
3868	A-17-0221			Strong ale/barley wine	27.01.01	Beverages - Alcohol - Wine	93401010	Wine, table, red
3869	17-0221			Strong ale/barley wine (7.2% ABV)	27.01.01	Beverages - Alcohol - Wine	93401010	Wine, table, red
3870	00-09617	A-00-09617		Strudel, fruit filled	04.02.00	Sweet cereal products - Pastries, Buns & Pies	53440000	Strudel, apple
3871	A-00-01166			Stuffed Heart, Heart Casserole	14.02.00	Offal - Other offal & dishes, e.g. Haggis, faggots	25112200	Liver paste or pate, chicken
3872	A-00-01162			Stuffing	01.04.00	Cereals & cereal dishes - Other cereals & dishes	51182010	Bread stuffing
3873	17-0372	A-17-0372		Stuffing mix, dried, made up	01.04.00	Cereals & cereal dishes - Other cereals & dishes	51182010	Bread stuffing

3874	A-11-0358			Stuffing, sage and onion	01.04.00	Cereals & cereal dishes - Other cereals & dishes	51182010	Bread stuffing
3875	A-00-06093			Stuffing, sage and onion 50	01.04.00	Cereals & cereal dishes - Other cereals & dishes	51182010	Bread stuffing
3876	17-0373	A-17-0373		Stuffing, sage and onion, homemade	01.04.00	Cereals & cereal dishes - Other cereals & dishes	51182010	Bread stuffing
3877	A-00-03787			Stuffing, sausagemeat 82/1429	13.00.00	Sausages & burgers & kebab	58128250	Dressing with meat and vegetables
3878	A-00-06052			Suet Pudding (dripping) 50	04.03.00	Sweet cereal products - Cereal based puddings (not milk)	53118100	Cake, sponge, without icing or filling
3879	A-00-00119			Suet Pudding, Steamed	04.03.00	Sweet cereal products - Cereal based puddings (not milk)	53118100	Cake, sponge, without icing or filling
3880	A-11-0330			Suet pudding	04.03.00	Sweet cereal products - Cereal based puddings (not milk)	53118100	Cake, sponge, without icing or filling
3881	A-00-00193			Suet, Block	08.03.00	Fats - Animal based fats (solid)	81201000	Animal fat or drippings
3882	A-00-00194			Suet, Shredded	08.03.00	Fats - Animal based fats (solid)	81201000	Animal fat or drippings
3883	17-0011			Suet, shredded	08.03.00	Fats - Animal based fats (solid)	81201000	Animal fat or drippings
3884	17-0012	A-17-0012		Suet, vegetable	08.04.01	Fats - Plant based fats (solid) - Full fat	81102000	Margarine, NFS
3885	A-00-00056	A-11-0152		Sugar Puffs	02.03.00	Breakfast cereals - Other breakfast cereals - low fibre	57100100	Cereal, ready-to-eat, NFS
3886	A-00-03049	00-03049		Sugar Puffs, Quaker	02.03.00	Breakfast cereals - Other breakfast cereals - low fibre	57100100	Cereal, ready-to-eat, NFS
3887	A-00-03789			Sugar Smacks 82/1431	02.03.00	Breakfast cereals - Other breakfast cereals - low fibre	57100100	Cereal, ready-to-eat, NFS
3888	00-05767			Sugar free mints/sweets	24.02.00	Confectionary - Sugar based products	91700010	Candy, NFS
3889	A-17-0061	17-0061	A-00-00842	Sugar, Demerara	23.01.00	Sugars - Pure sugars	91104100	Sugar, cinnamon
3890	A-00-01250			Sugar, Soft Brown (Light Brown)	23.01.00	Sugars - Pure sugars	91102010	Sugar, brown
3891	A-00-00843			Sugar, White	23.01.00	Sugars - Pure sugars	91101010	Sugar, white, granulated or lump
3892	17-0060	A-17-0060		Sugar, brown	23.01.00	Sugars - Pure sugars	91102010	Sugar, brown

3893	17-0062			Sugar, icing	23.01.00	Sugars - Pure sugars	91101020	Sugar, white, confectioner's, powdered
3894	17-0063	A-17-0063		Sugar, white	23.01.00	Sugars - Pure sugars	91101010	Sugar, white, granulated or lump
3895	A-11-0153	A-00-01020		Sultana Bran	02.02.00	Breakfast cereals - Other breakfast cereals - high fibre (equal or >3g/40g portion)	57329000	Cereal, raisin bran
3896	00-03050	A-00-03050		Sultana Bran, Kelloggs	02.02.00	Breakfast cereals - Other breakfast cereals - high fibre (equal or >3g/40g portion)	57329000	Cereal, raisin bran
3897	A-00-03051	00-03051		Sultana Bran, Own Brand	02.02.00	Breakfast cereals - Other breakfast cereals - high fibre (equal or >3g/40g portion)	57329000	Cereal, raisin bran
3898	14-0263	A-14-0263		Sultanas	18.03.00	Fruit - Dried	62125100	Raisins
3899	A-00-00819			Sultanas, Dried	18.03.00	Fruit - Dried	62125100	Raisins
3900	00-05803			Suma sunflower spread, fortified	08.04.01	Fats - Plant based fats (solid) - Full fat	81102000	Margarine, NFS
3901	00-03234			Summer pudding	04.03.00	Sweet cereal products - Cereal based puddings (not milk)	53101250	Cake, angel food, with fruit and icing or filling
3902	A-00-00206			Sunflower Seed Oil	08.02.00	Fats - Oils	82108500	Sunflower oil
3903	A-00-01186			Sunflower Seeds	19.00.00	Nuts & Seeds (incl. peanut butter)	43102400	Sunflower seeds, NFS
3904	A-17-0045	17-0045		Sunflower oil	08.02.00	Fats - Oils	82108500	Sunflower oil
3905	14-0845	A-14-0845		Sunflower seeds	19.00.00	Nuts & Seeds (incl. peanut butter)	43102400	Sunflower seeds, NFS
3906	14-0846			Sunflower seeds, toasted	19.00.00	Nuts & Seeds (incl. peanut butter)	43102400	Sunflower seeds, NFS
3907	00-03863	A-00-03863		Sunny delight any flavour RTD	27.02.02	Beverages - Fruit based drinks - Fruit juice drinks	92531030	Fruit juice drink (Sunny D)
3908	00-03034	A-00-03034		Sustain, Kelloggs	02.03.00	Breakfast cereals - Other breakfast cereals - low fibre	57100100	Cereal, ready-to-eat, NFS
3909	A-13-0361	13-0361		Swede, boiled in unsalted water	15.04.00	Vegetables - Other	75228000	Rutabaga, cooked, NS as to fat added in cooking

3910	A-00-00660			Swedes, Boiled	15.04.00	Vegetables - Other	75228000	Rutabaga, cooked, NS as to fat added in cooking
3911	A-00-00659			Swedes, Raw	15.04.00	Vegetables - Other	75127000	Rutabaga, raw
3912	A-00-03781			Sweet & Sour Sauce (packet mix, made up)	21.02.00	Sauces & accompaniment - Cooking sauces, incl. gravies, pesto, cooking sauces for pasta and rice dishes	91361010	Sweet and sour sauce
3913	00-09518	A-00-09518		Sweet & Sour, Chicken	11.01.00	Meat - white - Chicken & turkey & dishes	27146100	Sweet and sour chicken or turkey
3914	00-09519	A-00-09519		Sweet & Sour, pork, battered	10.03.00	Meat - red - Pork & dishes	27120060	Sweet and sour pork
3915	A-00-00665			Sweet Potatoes, Boiled	15.04.00	Vegetables - Other	73401000	Sweet potato, NFS
3916	A-00-00664			Sweet Potatoes, Raw	15.04.00	Vegetables - Other	73401000	Sweet potato, NFS
3917	A-00-01173			Sweet and Sour with Sauce, All Types	10.03.00	Meat - red - Pork & dishes	27120060	Sweet and sour pork
3918	A-17-0335			Sweet and sour sauce, canned	21.02.00	Sauces & accompaniment - Cooking sauces, incl. gravies, pesto, cooking sauces for pasta and rice dishes	91361010	Sweet and sour sauce
3919	A-17-0336	17-0336		Sweet and sour sauce, take-away	21.03.00	Sauces & accompaniment - Other sauces, incl. brown sauce, soy sauce, ketchup, mint sauce, vinegar	91361010	Sweet and sour sauce
3920	00-05638			Sweet chilli sauce	21.03.00	Sauces & accompaniment - Other sauces, incl. brown sauce, soy sauce, ketchup, mint sauce, vinegar	75511010	Hot pepper sauce
3921	00-03579	A-00-03579		Sweet potato, boiled, unsalted water	15.04.00	Vegetables - Other	73401000	Sweet potato, NFS
3922	A-00-00385			Sweetbread, Lamb, Fried	14.02.00	Offal - Other offal & dishes, e.g. Haggis, faggots	25140110	Sweetbreads, cooked
3923	A-00-00384			Sweetbread, Lamb, Raw	14.02.00	Offal - Other offal & dishes, e.g. Haggis, faggots	5027	Chicken, liver, all classes, raw

3924	A-00-00662			Sweetcorn, (Corn on the Cob), Boiled	15.04.00	Vegetables - Other	75216000	Corn, cooked, NS as to form, NS as to color, NS as to fat added in cooking
3925	A-00-00663			Sweetcorn, Canned, Kernels	15.04.00	Vegetables - Other	75216000	Corn, cooked, NS as to form, NS as to color, NS as to fat added in cooking
3926	A-00-00661			Sweetcorn, On the Cob, Raw	15.04.00	Vegetables - Other	75109600	Corn, raw
3927	A-13-0367	13-0367		Sweetcorn, baby, canned, drained	15.04.00	Vegetables - Other	75216000	Corn, cooked, NS as to form, NS as to color, NS as to fat added in cooking
3928	13-0366	A-13-0366		Sweetcorn, baby, fresh and frozen, boiled in salted water	15.04.00	Vegetables - Other	75216000	Corn, cooked, NS as to form, NS as to color, NS as to fat added in cooking
3929	13-0370			Sweetcorn, kernels, boiled in unsalted water	15.04.00	Vegetables - Other	75216000	Corn, cooked, NS as to form, NS as to color, NS as to fat added in cooking
3930	00-05911			Sweetcorn, kernels, canned, drained, no added salt/sugar	15.04.00	Vegetables - Other	75216000	Corn, cooked, NS as to form, NS as to color, NS as to fat added in cooking
3931	13-0371			Sweetcorn, kernels, canned, re-heated, drained	15.04.00	Vegetables - Other	75216000	Corn, cooked, NS as to form, NS as to color, NS as to fat added in cooking
3932	A-00-09939			Sweetcorn, kernels, canned, re-heated, drained (MW6 folate)	15.04.00	Vegetables - Other	75216000	Corn, cooked, NS as to form, NS as to color, NS as to fat added in cooking
3933	A-13-0374	13-0374		Sweetcorn, on-the-cob, whole, boiled in unsalted water	15.04.00	Vegetables - Other	75216000	Corn, cooked, NS as to form, NS as to color, NS as to fat added in cooking
3934	00-05629			Sweetener, artificial, low calorie	26.03.00	Miscellaneous - Artificial sweeteners	91200000	Sugar substitute, powder, NFS
3935	A-11-0216	11-0216		Swiss roll	04.02.00	Sweet cereal products - Pastries, Buns & Pies	53113000	Cake, jelly roll

3936	A-00-06030			Swiss roll 50	04.02.00	Sweet cereal products - Pastries, Buns & Pies	53113000	Cake, jelly roll
3937	11-0217	A-11-0217		Swiss rolls, chocolate, individual	04.02.00	Sweet cereal products - Pastries, Buns & Pies	53108200	Snack cake, chocolate, with icing or filling
3938	A-00-09956			Swiss rolls, chocolate, individual (MW6 Vit Eq)	04.02.00	Sweet cereal products - Pastries, Buns & Pies	53108200	Snack cake, chocolate, with icing or filling
3939	A-00-06063			Swiss rolls, chocolate, individual 50	04.02.00	Sweet cereal products - Pastries, Buns & Pies	53108200	Snack cake, chocolate, with icing or filling
3940	A-00-00844			Syrup, Golden	23.02.00	Sugars - Other, incl. syrups, honey	91301030	Corn syrup, light or dark
3941	17-0065	A-17-0065		Syrup, golden	23.02.00	Sugars - Other, incl. syrups, honey	91301030	Corn syrup, light or dark
3942	17-0067	A-17-0067		Syrup, maple	23.02.00	Sugars - Other, incl. syrups, honey	91301060	Maple syrup
3943	A-00-09525	00-09525		Szechuan prawns and vegetable	09.03.00	Fish & fish dishes - Shellfish	27450400	Shrimp and vegetables including carrots, broccoli, and/or dark-green leafy; no potatoes, no sauce
3944	02-02209			TABLE TOP SWEETENERS IN TABLETS OR MINI CUBES	26.03.00	Miscellaneous - Artificial sweeteners	91200000	Sugar substitute, powder, NFS
3945	02-09364			TABLE TOP SWEETENERS PER 100G	26.03.00	Miscellaneous - Artificial sweeteners	91200000	Sugar substitute, powder, NFS
3946	02-02726			TAGLIATELLE CARBONARA, REDUCED FAT, READY MEAL, E.G. ASDA HEALTHY CHOICE	01.02.00	Cereals & cereal dishes - Pasta & pasta dishes	58145110	Macaroni or noodles with cheese
3947	02-01087			TAKEAWAY CHICKEN PIECES BATTERED DEEP FRIED BONE NOT WEIGHED	11.01.00	Meat - white - Chicken & turkey & dishes	24107070	Chicken, NS as to part, fried, coated, skin / coating eaten
3948	02-01086			TAKEAWAY CHICKEN PIECES BATTERED DEEP FRIED EG KFC (BONE WEIGHED)	11.01.00	Meat - white - Chicken & turkey & dishes	24107070	Chicken, NS as to part, fried, coated, skin / coating eaten

3949	02-08318			TEA INSTANT WITH MILK POWDER EG TYPHOO QT	27.07.00	Beverages - Tea	92306800	Tea, hot, chai, with milk
3950	02-10310			TESCO FRUIT JUICE DRINK RTD FORTIFIED	27.02.02	Beverages - Fruit based drinks - Fruit juice drinks	92531030	Fruit juice drink (Sunny D)
3951	02-10374			TESCO SPECIAL FLAKES BREAKFAST CEREAL	02.03.00	Breakfast cereals - Other breakfast cereals - low fibre	57100100	Cereal, ready-to-eat, NFS
3952	02-08095			THAI CURRY SAUCE PURCHASED (YELLOW OR GREEN)	21.02.00	Sauces & accompaniment - Cooking sauces, incl. gravies, pesto, cooking sauces for pasta and rice dishes	75440600	Vegetable curry
3953	02-10015			THAI FRAGRANT RICE - COOKED	01.03.00	Cereals & cereal dishes - Rice & rice dishes	56205001	Rice, white, cooked, NS as to fat added in cooking
3954	02-06181			THROAT LOZENGES SUGAR FREE E.G HALLS	24.02.00	Confectionary - Sugar based products	91700010	Candy, NFS
3955	02-08309			TIC TAC	24.02.00	Confectionary - Sugar based products	91700010	Candy, NFS
3956	02-03013			TOFFEE AND CHOCOLATE DESSERT E.G WEIGHT WATCHERS	06.04.02	Dairy products - Ice cream & dairy desserts - reduced fat products	91760100	Toffee, chocolate covered
3957	02-02450			TOMATO SAUCE HOMEMADE	21.02.00	Sauces & accompaniment - Cooking sauces, incl. gravies, pesto, cooking sauces for pasta and rice dishes	11549	Tomato products, canned, sauce
3958	02-02480			TOMATO SOUP CONDENSED DILUTED WITH WATER	20.01.00	Soups - Canned & fresh & homemade	74602200	Tomato soup, canned, reduced sodium, prepared with water, or ready-to-serve
3959	02-09374			TORTES NOT CHOCOLATE BASED PURCHASED FROZEN	04.02.00	Sweet cereal products - Pastries, Buns & Pies	53118500	Cake, torte
3960	02-10070			TORTILLA CHIPS IN SUNSEED OR HIGH OLEIC SUNFLOWER OIL, EG DORITOS	25.02.00	Savoury Snacks - Cereal based snacks	54401075	Tortilla chips, plain

3961	02-07967			TRACKER BAR CHOCOLATE CHIP	04.05.00	Sweet cereal products - Cereal bars	53714200	Cereal or granola bar, chocolate coated, NFS
3962	02-07883			TRANSFORM-A-SNACK AND OTHER CEREAL MAINLY MAIZE AND POTATO SNACK	25.04.00	Savoury Snacks - Savoury biscuits & crackers	54401081	Cheese flavored corn snacks (Cheetos)
3963	02-06834			TUNA AND RED PEPPER FISH CAKES EG WAITROSE	09.01.00	Fish & fish dishes - White fish, incl. tuna	27250160	Tuna cake or patty
3964	02-08099			TUNA MAYONNAISE SANDWICH FILLERS	09.01.00	Fish & fish dishes - White fish, incl. tuna	27450060	Tuna salad, made with mayonnaise
3965	02-09856			TUNA TWIST IN MEDITERRANEAN TOMATO & HERB DRESSING (JOHN WEST)	09.01.00	Fish & fish dishes - White fish, incl. tuna	27150310	Fish with tomato-based sauce
3966	02-03960			TUNA, CANNED, IN SPRING WATER, FISH ONLY	09.01.00	Fish & fish dishes - White fish, incl. tuna	26155190	Tuna, canned, water pack
3967	02-08694			TURKEY AND PORK LUNCHEON MEAT EG. SAINSBURYS BILLY BEAR	12.03.00	Processed meat - Other processed meats	25230800	Turkey ham, prepackaged or deli, luncheon meat
3968	02-05296			TURKEY BREAST MEAT ONLY GRILL NO + FAT	11.01.00	Meat - white - Chicken & turkey & dishes	24201030	Turkey, light meat, skin eaten
3969	02-05301			TURKEY LEG THIGH MEAT ONLY NO SKIN CASS	11.01.00	Meat - white - Chicken & turkey & dishes	24202500	Turkey, thigh, cooked, skin not eaten
3970	02-05300			TURKEY MINCE ONLY, STEWED	11.01.00	Meat - white - Chicken & turkey & dishes	24201420	Turkey light or dark meat, stewed, skin eaten
3971	02-05303			TURKEY SLICES UNSMOKE PREPACK OR DELI	11.01.00	Meat - white - Chicken & turkey & dishes	25230780	Turkey, prepackaged or deli, luncheon meat
3972	02-02273			TURKISH DELIGHT	24.01.00	Confectionary - Chocolate based products	91700010	Candy, NFS
3973	02-08004			TWIN POT PROBIOTIC YOGURT WITH FRUIT AND/OR CRUNCH EG MULLER HEALTHY BALANCE	06.03.02	Dairy products - Yoghurt & drinking yoghurts, incl. buttermilk and probiotics - reduced or low fat products	11430000	Yogurt, NS as to type of milk, fruit

3974	15-0316	A-15-0316		Tabouleh	01.04.00	Cereals & cereal dishes - Other cereals & dishes	20013	Bulgur, cooked
3975	19-0279	A-19-0279		Tagliatelle + ham,mushroom,cheese chilled/frozen/longlife, reheated	01.02.00	Cereals & cereal dishes - Pasta & pasta dishes	58146120	Pasta with tomato-based sauce, cheese and meat
3976	A-15-0317			Tagliatelle, with vegetables, retail	01.02.00	Cereals & cereal dishes - Pasta & pasta dishes	58146160	Pasta with vegetables, no sauce or dressing
3977	14-0847	A-14-0847		Tahini paste	19.00.00	Nuts & Seeds (incl. peanut butter)	43103300	Tahini
3978	A-14-0266	14-0266		Tangerines	18.01.00	Fruit - Fresh	61119010	Orange, raw
3979	A-00-00820			Tangerines, Raw	18.01.00	Fruit - Fresh	61119010	Orange, raw
3980	A-00-00821			Tangerines, Raw (Weighed with Peel and Pips)	18.01.00	Fruit - Fresh	61119010	Orange, raw
3981	16-0307	A-16-0307	A-00-05076	Taramasalata	09.01.00	Fish & fish dishes - White fish, incl. tuna	27150020	Crab, deviled
3982	17-0337	A-17-0337		Tartare sauce	21.03.00	Sauces & accompaniment - Other sauces, incl. brown sauce, soy sauce, ketchup, mint sauce, vinegar	81302050	Tartar sauce
3983	00-03064			Taste the difference Jumbo Oats SAINSBURYS (RISCK-LA)	02.01.00	Breakfast cereals - Oat based cereals	57000100	Cereal, oat, NFS
3984	A-00-03847	00-03847		Tea decaffeinated, as poured	27.07.00	Beverages - Tea	92302000	Tea, hot, leaf, black
3985	A-00-09750	00-09750		Tea whitener, powder	27.05.00	Beverages - Powdered Beverages (cocoa, Horlicks, Bonvita, Ovaltine, etc)	12210400	Coffee creamer, powder
3986	A-00-03852	00-03852		Tea, BLACK, infusion	27.07.00	Beverages - Tea	92302000	Tea, hot, leaf, black
3987	A-00-00877			Tea, Indian Infusion	27.07.00	Beverages - Tea	92302000	Tea, hot, leaf, black
3988	17-0165	A-17-0165		Tea, black, infusion, average	27.07.00	Beverages - Tea	92302000	Tea, hot, leaf, black
3989	17-0167	A-17-0167		Tea, black, infusion, strong	27.07.00	Beverages - Tea	92302000	Tea, hot, leaf, black
3990	A-17-0166	17-0166		Tea, black, infusion, weak	27.07.00	Beverages - Tea	92302000	Tea, hot, leaf, black

3991	00-09697	A-00-09697		Tea, fruit flavour, made up with water	27.07.00	Beverages - Tea	92302000	Tea, hot, leaf, black
3992	17-0171	A-17-0171		Tea, green, infusion	27.07.00	Beverages - Tea	92302000	Tea, hot, leaf, black
3993	A-17-0172	17-0172		Tea, herbal, infusion	27.07.00	Beverages - Tea	92302000	Tea, hot, leaf, black
3994	A-00-06505			Tea, infusion, 50	27.07.00	Beverages - Tea	92302000	Tea, hot, leaf, black
3995	00-03853	A-00-03853		Tea, lemon powder, made up with water	27.07.00	Beverages - Tea	92302000	Tea, hot, leaf, black
3996	A-17-0174	17-0174		Tea, lemon, instant powder, with water	27.07.00	Beverages - Tea	92302000	Tea, hot, leaf, black
3997	A-00-01045			Teacake, Toasted	04.02.00	Sweet cereal products - Pastries, Buns & Pies	51160100	Roll, sweet, cinnamon bun, no frosting
3998	A-11-0273	11-0273		Teacakes, fresh	04.02.00	Sweet cereal products - Pastries, Buns & Pies	51160100	Roll, sweet, cinnamon bun, no frosting
3999	A-00-06042			Teacakes, fresh 50	04.02.00	Sweet cereal products - Pastries, Buns & Pies	51160100	Roll, sweet, cinnamon bun, no frosting
4000	A-11-0274	11-0274		Teacakes, toasted	04.02.00	Sweet cereal products - Pastries, Buns & Pies	51160100	Roll, sweet, cinnamon bun, no frosting
4001	A-00-06081			Teacakes, toasted 50	04.02.00	Sweet cereal products - Pastries, Buns & Pies	51160100	Roll, sweet, cinnamon bun, no frosting
4002	00-05782			Tesco Cereal Bar, with fruit, fortified	04.05.00	Sweet cereal products - Cereal bars	53710700	Cereal or granola bar (Kellogg's Special K bar)
4003	00-05479			Tesco Delight Vanilla No Added Sugar + Semi S Milk	06.04.02	Dairy products - Ice cream & dairy desserts - reduced fat products	13130100	Light ice cream, NS as to flavor
4004	00-05527			Tesco Finest Babyleaf salad with basil	15.04.00	Vegetables - Other	75109400	Basil, raw
4005	00-05902			Tesco Healthly Living Enriched Olive Spread Light ONLY	08.04.03	Fats - Plant based fats (solid) - Low fat	81102000	Margarine, NFS
4006	00-04280			Tesco Onion and Garlic Dip	21.03.00	Sauces & accompaniment - Other sauces, incl. brown sauce, soy sauce, ketchup, mint sauce, vinegar	12350220	Onion dip, regular

4007	00-05580			Tesco Yorkshire Pudding	01.04.00	Cereals & cereal dishes - Other cereals & dishes	52311010	Popover
4008	00-05564			Tesco cholesterol reducing margarine	08.04.01	Fats - Plant based fats (solid) - Full fat	81102000	Margarine, NFS
4009	A-00-01116			Textured Vegetable Protein, Dry	15.04.00	Vegetables - Other	41440000	Textured vegetable protein, dry
4010	00-01117	A-00-01117		Textured Vegetable Protein, Reconstituted	15.04.00	Vegetables - Other	59003000	Meat substitute, cereal- and vegetable protein-based, fried
4011	00-05715			Thai Chicken Soup, Chilled, Carton, ready to serve	20.01.00	Soups - Canned & fresh & homemade	58137230	Pad Thai with chicken
4012	00-05845			Thai Prawn Fishcakes	09.03.00	Fish & fish dishes - Shellfish	27250400	Shrimp cake or patty
4013	00-09537	A-00-09537		Thai Vegetable Curry, stir fry	15.04.00	Vegetables - Other	75440600	Vegetable curry
4014	00-09673	A-00-09673		Thick&creamy twinpot yoghurt +fruit	06.03.01	Dairy products - Yoghurt & drinking yoghurts, incl. buttermilk and probiotics - full fat products	11430000	Yogurt, NS as to type of milk, fruit
4015	13-0860	A-13-0860		Thyme, dried, ground	26.01.00	Miscellaneous - Dried herbs & spices & pastes	2042	Spices, thyme, dried
4016	13-0859			Thyme, fresh	19.00.00	Nuts & Seeds (incl. peanut butter)	75119000	Parsley, raw
4017	00-05617			Tiger Bread/Rolls	03.01.00	Breads - White	51153000	Roll, white, hard
4018	A-12-0130			Tip Top	06.01.00	Dairy products - Cream & fromage frais	12100100	Cream, NS as to light, heavy, or half and half
4019	00-09772	A-00-09772		Tip Top (Nestle)	06.01.00	Dairy products - Cream & fromage frais	12100100	Cream, NS as to light, heavy, or half and half
4020	A-00-03747			Tip top dessert topping 82/1180	06.01.00	Dairy products - Cream & fromage frais	12100100	Cream, NS as to light, heavy, or half and half
4021	A-00-09624	00-09624		Tiramisu	06.04.01	Dairy products - Ice cream & dairy desserts - full fat products	13252600	Tiramisu
4022	A-00-01089			Toad in the Hole	13.00.00	Sausages & burgers & kebab	32105190	Egg casserole with bread, cheese, milk and meat

4023	A-19-0280	19-0280		Toad in the hole	13.00.00	Sausages & burgers & kebab	32105190	Egg casserole with bread, cheese, milk and meat
4024	A-00-00866			Toffees, Mixed	24.02.00	Confectionary - Sugar based products	91702010	Butterscotch morsels
4025	17-0120	A-17-0120		Toffees, mixed	24.02.00	Confectionary - Sugar based products	91702010	Butterscotch morsels
4026	13-0119	A-13-0119		Tofu, soya bean, steamed	16.01.00	Pulses/Lentils - Pulses/lentils	16129	Tofu, fried
4027	13-0120	A-13-0120		Tofu, soya bean, steamed, fried	16.01.00	Pulses/Lentils - Pulses/lentils	16129	Tofu, fried
4028	00-05598			Tomato & Mascarpone Sauce e.g. Tesco/Sainsbury's	21.02.00	Sauces & accompaniment - Cooking sauces, incl. gravies, pesto, cooking sauces for pasta and rice dishes	75412070	Eggplant with cheese and tomato sauce
4029	A-00-00890			Tomato Juice, Canned	27.02.00	Beverages - Fruit based drinks	74302000	Tomato juice cocktail
4030	A-00-00931			Tomato Ketchup	21.03.00	Sauces & accompaniment - Other sauces, incl. brown sauce, soy sauce, ketchup, mint sauce, vinegar	74401010	Tomato catsup
4031	00-05329			Tomato Ketchup, Reduced sugar and salt e.g. Tesco	21.03.00	Sauces & accompaniment - Other sauces, incl. brown sauce, soy sauce, ketchup, mint sauce, vinegar	74401110	Tomato catsup, reduced sodium
4032	A-00-00932			Tomato Puree	15.01.02	Vegetables - Tomatoes - Puree & sun-dried	11547	Tomato products, canned, puree, without salt added
4033	A-00-00933			Tomato Sauce	21.03.00	Sauces & accompaniment - Other sauces, incl. brown sauce, soy sauce, ketchup, mint sauce, vinegar	11549	Tomato products, canned, sauce
4034	A-00-00951			Tomato Soup, Condensed	20.01.00	Soups - Canned & fresh & homemade	6159	Soup, tomato, canned, condensed

4035	A-00-00952			Tomato Soup, Condensed, as Served	20.01.00	Soups - Canned & fresh & homemade	74602200	Tomato soup, canned, reduced sodium, prepared with water, or ready-to-serve
4036	A-00-00950			Tomato Soup, Cream Of, Canned Ready To Serve	20.01.00	Soups - Canned & fresh & homemade	74601010	Tomato soup, cream of, prepared with milk
4037	A-00-00954			Tomato Soup, Dried, as Served	20.01.00	Soups - Canned & fresh & homemade	6498	Soup, tomato, dry, mix, prepared with water
4038	13-0382	A-13-0382		Tomato juice	27.02.01	Beverages - Fruit based drinks - Pure fruit juice & smoothies	74301100	Tomato juice, 100%
4039	17-0338			Tomato ketchup	21.03.00	Sauces & accompaniment - Other sauces, incl. brown sauce, soy sauce, ketchup, mint sauce, vinegar	74401010	Tomato catsup
4040	A-00-09910			Tomato ketchup (MW6 carq)	21.03.00	Sauces & accompaniment - Other sauces, incl. brown sauce, soy sauce, ketchup, mint sauce, vinegar	74401010	Tomato catsup
4041	A-13-0383	17-0374		Tomato puree	15.01.02	Vegetables - Tomatoes - Puree & sun-dried	11547	Tomato products, canned, puree, without salt added
4042	00-09911	A-00-09911		Tomato puree (MW6 carq)	15.01.02	Vegetables - Tomatoes - Puree & sun-dried	11547	Tomato products, canned, puree, without salt added
4043	00-05900			Tomato sauce, cooked in specified fat	21.02.00	Sauces & accompaniment - Cooking sauces, incl. gravies, pesto, cooking sauces for pasta and rice dishes	11549	Tomato products, canned, sauce
4044	A-17-0278	17-0278		Tomato soup, cream of, canned	20.01.00	Soups - Canned & fresh & homemade	74601010	Tomato soup, cream of, prepared with milk
4045	A-17-0280	17-0280		Tomato soup, cream of, canned, condensed, as served	20.01.00	Soups - Canned & fresh & homemade	74601010	Tomato soup, cream of, prepared with milk

4046	A-17-0282			Tomato soup, dried, as served	20.01.00	Soups - Canned & fresh & homemade	6498	Soup, tomato, dry, mix, prepared with water
4047	00-05631			Tomatoes fried in blended oil	15.01.01	Vegetables - Tomatoes - Raw & canned	74202050	Tomatoes, red, NS as to form, fried
4048	A-00-00668			Tomatoes, Canned	15.01.01	Vegetables - Tomatoes - Raw & canned	74204500	Tomatoes, canned, low sodium
4049	A-00-00667			Tomatoes, Fried	15.01.00	Vegetables - Tomatoes	74202050	Tomatoes, red, NS as to form, fried
4050	A-00-06304			Tomatoes, Fried (bacon fat) 50	15.01.00	Vegetables - Tomatoes	74202050	Tomatoes, red, NS as to form, fried
4051	A-00-00666			Tomatoes, Raw	15.01.01	Vegetables - Tomatoes - Raw & canned	74101000	Tomatoes, raw
4052	A-13-0387	13-0387		Tomatoes, canned, whole contents	15.01.01	Vegetables - Tomatoes - Raw & canned	74204500	Tomatoes, canned, low sodium
4053	A-13-0388	13-0388		Tomatoes, cherry, raw	15.01.01	Vegetables - Tomatoes - Raw & canned	74101000	Tomatoes, raw
4054	13-0385	A-13-0385		Tomatoes, fried in corn oil	15.01.01	Vegetables - Tomatoes - Raw & canned	74202050	Tomatoes, red, NS as to form, fried
4055	A-13-0419			Tomatoes, fried in lard	15.01.01	Vegetables - Tomatoes - Raw & canned	74202050	Tomatoes, red, NS as to form, fried
4056	A-00-03587	00-03587		Tomatoes, fried in specified fat	15.01.01	Vegetables - Tomatoes - Raw & canned	74202050	Tomatoes, red, NS as to form, fried
4057	13-0386			Tomatoes, grilled	15.01.01	Vegetables - Tomatoes - Raw & canned	74202010	Tomatoes, NS as to form, broiled
4058	A-00-09909			Tomatoes, grilled (MW6 carq; MW6 folate)	15.01.01	Vegetables - Tomatoes - Raw & canned	74202010	Tomatoes, NS as to form, broiled
4059	13-0384			Tomatoes, raw	15.01.01	Vegetables - Tomatoes - Raw & canned	74101000	Tomatoes, raw
4060	A-00-09908			Tomatoes, raw (MW6 carq; MW6 folate)	15.01.01	Vegetables - Tomatoes - Raw & canned	74101000	Tomatoes, raw
4061	00-03626			Tomatoes, sundried, dry	15.01.02	Vegetables - Tomatoes - Puree & sun-dried	74206000	Tomatoes, red, dried
4062	00-03625			Tomatoes, sundried, in oil	15.01.02	Vegetables - Tomatoes - Puree & sun-dried	74206000	Tomatoes, red, dried

4063	A-00-00398			Tongue, Canned	14.02.00	Offal - Other offal & dishes, e.g. Haggis, faggots	25160110	Tongue, smoked, cured, or pickled, cooked
4064	A-00-00387			Tongue, Lamb, Raw	14.02.00	Offal - Other offal & dishes, e.g. Haggis, faggots	5027	Chicken, liver, all classes, raw
4065	A-00-00390			Tongue, Ox, Pickled, Boiled	14.02.00	Offal - Other offal & dishes, e.g. Haggis, faggots	25160110	Tongue, smoked, cured, or pickled, cooked
4066	19-0153	A-19-0153		Tongue, canned	14.02.00	Offal - Other offal & dishes, e.g. Haggis, faggots	25160110	Tongue, smoked, cured, or pickled, cooked
4067	18-0425	A-18-0425		Tongue, ox, pickled, stewed	14.02.00	Offal - Other offal & dishes, e.g. Haggis, faggots	25160110	Tongue, smoked, cured, or pickled, cooked
4068	A-17-0184	17-0184		Tonic water	27.03.00	Beverages - Carbonated soft drinks	92410110	Carbonated water, sweetened
4069	17-0238	A-17-0238		Tonic wine, Sanatogen	27.01.01	Beverages - Alcohol - Wine	93401010	Wine, table, red
4070	00-09678	A-00-09678		Torte, chilled or frozen, fruit	04.02.00	Sweet cereal products - Pastries, Buns & Pies	53118500	Cake, torte
4071	00-05378			Tortelloni - Smoked Ham and Bacon	01.02.00	Cereals & cereal dishes - Pasta & pasta dishes	58134650	Tortellini, meat-filled, no sauce
4072	00-05722			Tortilla Chips, Low fat. e.g. Weight Watchers	25.02.00	Savoury Snacks - Cereal based snacks	54401170	Tortilla chips, low fat, unsalted
4073	00-09571	A-00-09571		Tortilla bread (wheat, large, soft)	03.04.00	Breads - Other bread	52215200	Tortilla, flour
4074	00-05826			Tortilla bread/wrap, low fat	03.04.00	Breads - Other bread	52215200	Tortilla, flour
4075	17-0149	A-17-0149		Tortilla chips	25.02.00	Savoury Snacks - Cereal based snacks	54401075	Tortilla chips, plain
4076	00-05597			Total 0% Fat Greek Yoghurt	06.03.02	Dairy products - Yoghurt & drinking yoghurts, incl. buttermilk and probiotics - reduced or low fat products	11400000	Yogurt, NFS
4077	00-05928			Total Balance LighterLife Foodpack, any flavour	30.00.00	Nutrition Powders & drinks	53729000	Nutrition bar or meal replacement bar, NFS
4078	00-05929			Total Balance LighterLife porridge	30.00.00	Nutrition Powders & drinks	53729000	Nutrition bar or meal replacement bar, NFS

4079	00-03125	A-00-03125		Tracker bar, peanut	04.05.00	Sweet cereal products - Cereal bars	53714220	Cereal or granola bar with nuts, chocolate coated
4080	14-0849			Trail mix	19.00.00	Nuts & Seeds (incl. peanut butter)	42500000	Trail mix, NFS
4081	A-00-00120			Treacle Tart	04.02.00	Sweet cereal products - Pastries, Buns & Pies	53390000	Pie, shoo-fly
4082	A-11-0331	11-0331		Treacle tart	04.02.00	Sweet cereal products - Pastries, Buns & Pies	53390000	Pie, shoo-fly
4083	A-00-06049			Treacle tart 50	04.02.00	Sweet cereal products - Pastries, Buns & Pies	53390000	Pie, shoo-fly
4084	A-00-00845			Treacle, Black	23.02.00	Sugars - Other, incl. syrups, honey	91303000	Molasses
4085	A-17-0068	17-0068		Treacle, black	23.02.00	Sugars - Other, incl. syrups, honey	91303000	Molasses
4086	A-00-01060			Treacle/Jam Sponge Pudding	04.03.00	Sweet cereal products - Cereal based puddings (not milk)	53113000	Cake, jelly roll
4087	A-00-00121	A-12-0249		Trifle	06.04.01	Dairy products - Ice cream & dairy desserts - full fat products	13252600	Tiramisu
4088	A-00-03790			Trifle (packet mix) 82/1432	06.04.02	Dairy products - Ice cream & dairy desserts - reduced fat products	13252600	Tiramisu
4089	A-00-06009			Trifle 50	06.04.02	Dairy products - Ice cream & dairy desserts - reduced fat products	13252600	Tiramisu
4090	A-12-0250	11-0333		Trifle, frozen	06.04.01	Dairy products - Ice cream & dairy desserts - full fat products	13252600	Tiramisu
4091	00-09661	A-00-09661		Trifle, fruit, retail	06.04.02	Dairy products - Ice cream & dairy desserts - reduced fat products	13252600	Tiramisu
4092	12-0251	A-12-0251		Trifle, with Dream Topping	06.04.02	Dairy products - Ice cream & dairy desserts - reduced fat products	13252600	Tiramisu

4093	12-0252	A-12-0252		Trifle, with fresh cream	06.04.01	Dairy products - Ice cream & dairy desserts - full fat products	13252600	Tiramisu
4094	A-19-0282			Tripe and onions, stewed	14.02.00	Offal - Other offal & dishes, e.g. Haggis, faggots	25170110	Tripe, cooked
4095	A-00-00392			Tripe, Stewed	14.02.00	Offal - Other offal & dishes, e.g. Haggis, faggots	25170110	Tripe, cooked
4096	00-03881			Tropicana tropical juice blend	27.02.01	Beverages - Fruit based drinks - Pure fruit juice & smoothies	61210000	Orange juice, 100%, NFS
4097	A-18-0429			Trotters and tails, boiled	14.02.00	Offal - Other offal & dishes, e.g. Haggis, faggots	22705010	Pork ears, tail, head, snout, miscellaneous parts, cooked
4098	A-00-00506			Trout, Brown, Steamed	09.02.00	Fish & fish dishes - Oily fish	26151160	Trout, steamed or poached
4099	A-00-00507			Trout, Steamed (Weighed with Bones)	09.02.00	Fish & fish dishes - Oily fish	26151160	Trout, steamed or poached
4100	A-16-0226	16-0226		Trout, rainbow, grilled	09.02.00	Fish & fish dishes - Oily fish	26151123	Trout, baked or broiled, made without fat
4101	A-17-0098	17-0098		Truffles, mocha	24.01.00	Confectionary - Chocolate based products	91760500	Truffles
4102	17-0099	A-17-0099		Truffles, rum	24.01.00	Confectionary - Chocolate based products	91760500	Truffles
4103	00-03548	A-00-03548		Tuna mayonnaise	09.01.00	Fish & fish dishes - White fish, incl. tuna	27450060	Tuna salad, made with mayonnaise
4104	00-03645			Tuna mayonnaise, Weight Watchers	09.01.00	Fish & fish dishes - White fish, incl. tuna	27450060	Tuna salad, made with mayonnaise
4105	A-00-03596	00-03596		Tuna pasta bake	01.02.00	Cereals & cereal dishes - Pasta & pasta dishes	27350080	Tuna noodle casserole with vegetables, cream or white sauce
4106	A-16-0308	16-0308		Tuna pate	09.01.00	Fish & fish dishes - White fish, incl. tuna	27150050	Fish timbale or mousse
4107	00-05644			Tuna steak, fresh tuna, grilled	09.01.00	Fish & fish dishes - White fish, incl. tuna	26153122	Tuna, fresh, baked or broiled, fat not added in cooking

4108	A-00-00508			Tuna, Canned, in Oil	09.02.00	Fish & fish dishes - Oily fish	26151110	Trout, cooked, NS as to cooking method
4109	16-0229	A-16-0229		Tuna, canned in brine, drained	09.01.00	Fish & fish dishes - White fish, incl. tuna	26155190	Tuna, canned, water pack
4110	A-00-01365			Tuna, canned in brine, drained solids	09.02.00	Fish & fish dishes - Oily fish	26151110	Trout, cooked, NS as to cooking method
4111	A-16-0230	16-0230		Tuna, canned in oil, drained	09.01.00	Fish & fish dishes - White fish, incl. tuna	26155180	Tuna, canned, oil pack
4112	A-00-01364			Tuna, canned in oil, drained solids	09.02.00	Fish & fish dishes - Oily fish	26151110	Trout, cooked, NS as to cooking method
4113	16-0228			Tuna, raw	09.01.00	Fish & fish dishes - White fish, incl. tuna	26153100	Tuna, fresh, raw
4114	A-00-03124	00-03124		Tunnocks Snowball (no biscuit)	04.02.00	Sweet cereal products - Pastries, Buns & Pies	91723050	Marshmallow, coconut-coated
4115	A-00-03126	00-03126		Tunnocks caramel wafer biscuit	04.01.00	Sweet cereal products - Biscuits	53209010	Cookie, sugar wafer, chocolate-covered
4116	00-03123	A-00-03123		Tunnocks, Chocolate Mashmallow Teacakes	04.02.00	Sweet cereal products - Pastries, Buns & Pies	53208000	Cookie, marshmallow, chocolate-covered
4117	00-03631			Turkey Escalopes in crumb (eg Bernard Matthews)	11.01.00	Meat - white - Chicken & turkey & dishes	24201070	Turkey, light meat, breaded, baked or fried, skin eaten
4118	A-00-03744			Turkey Fat (100% fat) 82/985	08.03.00	Fats - Animal based fats (solid)	81201000	Animal fat or drippings
4119	A-00-00347			Turkey, Roast, Dark Meat	11.01.00	Meat - white - Chicken & turkey & dishes	24201230	Turkey, dark meat, roasted, skin eaten
4120	A-00-00346			Turkey, Roast, Light Meat	11.01.00	Meat - white - Chicken & turkey & dishes	24201130	Turkey, light meat, roasted, skin eaten
4121	A-00-00344			Turkey, Roast, Meat Only	11.01.00	Meat - white - Chicken & turkey & dishes	24201330	Turkey, light and dark meat, roasted, skin eaten
4122	A-00-00345			Turkey, Roast, Meat and Skin	11.01.00	Meat - white - Chicken & turkey & dishes	24201330	Turkey, light and dark meat, roasted, skin eaten
4123	A-18-0358	18-0358		Turkey, dark meat, roasted	11.01.00	Meat - white - Chicken & turkey & dishes	24201230	Turkey, dark meat, roasted, skin eaten

4124	A-18-0359	18-0359		Turkey, light meat, roasted	11.01.00	Meat - white - Chicken & turkey & dishes	24201130	Turkey, light meat, roasted, skin eaten
4125	A-18-0361	18-0361		Turkey, meat, average, roasted	11.01.00	Meat - white - Chicken & turkey & dishes	24201330	Turkey, light and dark meat, roasted, skin eaten
4126	18-0357			Turkey, strips, stir-fried	11.01.00	Meat - white - Chicken & turkey & dishes	24201000	Turkey, NFS
4127	A-17-0121			Turkish delight, with nuts	24.02.00	Confectionary - Sugar based products	91501110	Gelatin dessert with fruit and whipped topping
4128	17-0122	A-17-0122		Turkish delight, without nuts	24.02.00	Confectionary - Sugar based products	91700010	Candy, NFS
4129	13-0861	A-13-0861		Turmeric, ground	26.01.00	Miscellaneous - Dried herbs & spices & pastes	2043	Spices, turmeric, ground
4130	A-00-01279			Turnip Bhajia	15.04.00	Vegetables - Other	75234000	Turnip, cooked, NS as to form, NS as to fat added in cooking
4131	A-00-00671			Turnip Tops, Boiled	15.04.00	Vegetables - Other	72128200	Turnip greens, cooked, NS as to form, NS as to fat added in cooking
4132	A-13-0392			Turnip tops, boiled in unsalted water	15.04.00	Vegetables - Other	72128200	Turnip greens, cooked, NS as to form, NS as to fat added in cooking
4133	13-0391	A-13-0391		Turnip, boiled in unsalted water	15.04.00	Vegetables - Other	75234000	Turnip, cooked, NS as to form, NS as to fat added in cooking
4134	A-00-00670			Turnips, Boiled	15.04.00	Vegetables - Other	75234000	Turnip, cooked, NS as to form, NS as to fat added in cooking
4135	A-00-00669			Turnips, Raw	15.04.00	Vegetables - Other	75129000	Turnip, raw
4136	A-17-0150			Twiglets	25.02.00	Savoury Snacks - Cereal based snacks	54408016	Pretzels, hard, plain, salted
4137	17-0100	A-17-0100		Twix	24.01.00	Confectionary - Chocolate based products	91703200	TWIX Caramel Cookie Bars
4138	12-0293	A-12-0293		Tzatziki	21.01.00	Sauces & accompaniment - Dressings & Mayonnaise	11440060	Tzatziki dip

4139	A-00-09765	00-09765		UHT cream, aerosol,	06.01.00	Dairy products - Cream & fromage frais	12140000	Cream, whipped
4140	A-00-09766	00-09766		UHT cream, aerosol, half fat	06.01.00	Dairy products - Cream & fromage frais	12100100	Cream, NS as to light, heavy, or half and half
4141	00-05698			UTTERLY BUTTERLY WITH OMEGA 3	08.04.02	Fats - Plant based fats (solid) - Reduced fat	81102000	Margarine, NFS
4142	A-00-03851	00-03851		Unspecified fruit juice	27.02.01	Beverages - Fruit based drinks - Pure fruit juice & smoothies	61210000	Orange juice, 100%, NFS
4143	02-10417			VANILLA EXTRACT, VANILLA ESSENCE (NO ALCOHOL)	26.01.00	Miscellaneous - Dried herbs & spices & pastes	2052	Vanilla extract, imitation, no alcohol
4144	02-01051			VEAL FILLET ESCALOPE SCHNITZEL FRIED LEAN ONLY	10.01.00	Meat - red - Beef & veal & dishes	23200120	Veal, NS as to cut, cooked, lean only eaten
4145	02-06620			VEGETABLE BEAN SOUP	20.01.00	Soups - Canned & fresh & homemade	75656020	Vegetable soup, chunky style
4146	02-10153			VEGETABLE CHOW MEIN	15.04.00	Vegetables - Other	75439500	Chow mein or chop suey, meatless, no noodles
4147	02-01943			VEGETABLE CURRY	15.04.00	Vegetables - Other	75440600	Vegetable curry
4148	02-06995			VEGETABLE ENCHILADAS	15.04.00	Vegetables - Other	58100805	Enchilada, just cheese, meatless, no beans, green-chile or enchilada sauce
4149	02-08288			VEGETABLE FINGERS BREADCRUMBS GRILLED	15.04.00	Vegetables - Other	75412010	Eggplant, batter-dipped, fried
4150	02-01944			VEGETABLE JUICE MIXED	15.04.00	Vegetables - Other	75132000	Mixed vegetable juice
4151	02-03976			VEGETABLE LASAGNE READY MEAL, LOW FAT E.G. SAINBURY'S BE GOOD TO YOURSELF	15.04.00	Vegetables - Other	58301150	Zucchini lasagna, diet frozen meal
4152	02-03669			VEGETABLE MISO SOUP, NO OIL	20.01.00	Soups - Canned & fresh & homemade	41601070	Soybean soup, miso broth
4153	02-08290			VEGETABLE MOUSSAKA READY MEAL COOKED	15.04.00	Vegetables - Other	58301150	Zucchini lasagna, diet frozen meal

4154	02-08681			VEGETABLE RISOTTO	01.03.00	Cereals & cereal dishes - Rice & rice dishes	58160700	Rice, white, with other vegetables, NS as to fat added in cooking
4155	02-07928			VEGETABLE SOUP CARTON	20.01.00	Soups - Canned & fresh & homemade	75649010	Vegetable soup, canned, prepared with water or ready-to-serve
4156	02-03411			VEGETARIAN CHEESE & ONION QUICHE, REDUCED FAT	07.00.00	Egg & egg dishes	58125180	Cheese quiche, meatless
4157	02-08291			VEGETARIAN PATE PURCHASED	15.04.00	Vegetables - Other	75219030	Mushrooms, cooked, NS as to form, made with oil
4158	02-10186			VENISON SAUSAGES, BAKED OR GRILLED	13.00.00	Sausages & burgers & kebab	23322100	Deer sausage
4159	02-09403			VENISON STEWED MEAT ONLY	10.04.00	Meat - red - Other red meat, e.g. rabbit, venison	23322400	Venison/deer, stewed
4160	02-04045			VOL AU VENTS MADE WITH MUSHROOM SAUCE AND PASTRY	01.04.00	Cereals & cereal dishes - Other cereals & dishes	58127110	Vegetables in pastry
4161	A-00-01046			Vanilla Slice	04.02.00	Sweet cereal products - Pastries, Buns & Pies	53344070	Pie, custard, individual size or tart
4162	11-0275	A-11-0275		Vanilla slices	04.02.00	Sweet cereal products - Pastries, Buns & Pies	53344070	Pie, custard, individual size or tart
4163	A-00-00311			Veal Cutlet, Fried	10.01.00	Meat - red - Beef & veal & dishes	23200120	Veal, NS as to cut, cooked, lean only eaten
4164	A-00-06200			Veal Cutlet, Fried (dripping) 50	10.01.00	Meat - red - Beef & veal & dishes	23200120	Veal, NS as to cut, cooked, lean only eaten
4165	19-0286			Veal escalope; Wiener schnitzel	10.01.00	Meat - red - Beef & veal & dishes	23200120	Veal, NS as to cut, cooked, lean only eaten
4166	00-05910			Veal shank, lean and fat, cooked	10.01.00	Meat - red - Beef & veal & dishes	23200120	Veal, NS as to cut, cooked, lean only eaten
4167	A-00-00312			Veal, Fillet, Raw	10.01.00	Meat - red - Beef & veal & dishes	17134	Veal, sirloin, separable lean and fat, raw
4168	A-00-00313			Veal, Fillet, Roast	10.01.00	Meat - red - Beef & veal & dishes	23200120	Veal, NS as to cut, cooked, lean only eaten

4169	A-00-00400			Veal, Jellied	10.01.00	Meat - red - Beef & veal & dishes	23200120	Veal, NS as to cut, cooked, lean only eaten
4170	18-0093	A-18-0093		Veal, escalope, fried	10.01.00	Meat - red - Beef & veal & dishes	23200120	Veal, NS as to cut, cooked, lean only eaten
4171	18-0095			Veal, mince, stewed	10.01.00	Meat - red - Beef & veal & dishes	23200120	Veal, NS as to cut, cooked, lean only eaten
4172	A-00-03737			Veg Oil (20% soya, 80% rapeseed, low erucic acid) 82/975	08.02.00	Fats - Oils	82101000	Vegetable oil, NFS
4173	A-00-03735			Veg Oil (50% soya, 50% palm) 82/973	08.02.00	Fats - Oils	82101000	Vegetable oil, NFS
4174	A-00-03734			Veg Oil (50% soya, 50% rapeseed) 82/972	08.02.00	Fats - Oils	82101000	Vegetable oil, NFS
4175	A-00-03733			Veg Oil (33% soya, 33% palm, 33% rapeseed) 82/971	08.02.00	Fats - Oils	82101000	Vegetable oil, NFS
4176	A-00-03736			Veg Oil (80% soya, 20% palm) 82/974	08.02.00	Fats - Oils	82101000	Vegetable oil, NFS
4177	A-00-01397			Vegeburger, average	15.04.00	Vegetables - Other	41811890	Vegetarian burger or patty, meatless, no bun
4178	15-0330	A-15-0330		Vegeburger, retail, fried in vegetable oil	16.01.00	Pulses/Lentils - Pulses/lentils	41811890	Vegetarian burger or patty, meatless, no bun
4179	A-15-0331	15-0331		Vegeburger, retail, grilled	16.01.00	Pulses/Lentils - Pulses/lentils	41811890	Vegetarian burger or patty, meatless, no bun
4180	00-09505	A-00-09505		Vegetable Balti	15.04.00	Vegetables - Other	75440600	Vegetable curry
4181	A-00-01178			Vegetable Bhaji, All Types	15.04.00	Vegetables - Other	75440400	Vegetables, dipped in chick-pea flour batter, fried, Pakora
4182	A-00-09510			Vegetable Biryani	15.04.00	Vegetables - Other	58160000	Biryani with vegetables
4183	00-05327			Vegetable Crisps, fried in sunflower oil	25.03.00	Savoury Snacks - Vegetable based snacks	71220000	Vegetable chips
4184	A-00-01177			Vegetable Curry, All Types	15.04.00	Vegetables - Other	75440600	Vegetable curry

4185	A-00-09533			Vegetable Enchiladas	15.04.00	Vegetables - Other	58100805	Enchilada, just cheese, meatless, no beans, green-chile or enchilada sauce
4186	A-00-00195			Vegetable Oils	08.02.00	Fats - Oils	82101000	Vegetable oil, NFS
4187	A-00-01120			Vegetable Salad, Canned with Salad Cream	15.04.00	Vegetables - Other	75142500	Cucumber salad, made with sour cream dressing
4188	A-00-01114			Vegetable Salad, Mixed, Summer (no Dressing)	15.04.00	Vegetables - Other	75143000	Lettuce, salad with assorted vegetables including tomatoes and/or carrots, no dressing
4189	A-00-01115			Vegetable Salad, Mixed, Winter (no Dressing)	15.04.00	Vegetables - Other	75143000	Lettuce, salad with assorted vegetables including tomatoes and/or carrots, no dressing
4190	A-00-00955			Vegetable Soup, Canned, Ready To Serve	20.01.00	Soups - Canned & fresh & homemade	75649010	Vegetable soup, canned, prepared with water or ready-to-serve
4191	17-0046	A-17-0046		Vegetable oil, blended, average	08.02.00	Fats - Oils	82101000	Vegetable oil, NFS
4192	15-0342	A-15-0342		Vegetable pancake roll	15.04.00	Vegetables - Other	58127110	Vegetables in pastry
4193	17-0283	A-17-0283		Vegetable soup	20.01.00	Soups - Canned & fresh & homemade	75649010	Vegetable soup, canned, prepared with water or ready-to-serve
4194	17-0284	A-17-0284		Vegetable soup, canned	20.01.00	Soups - Canned & fresh & homemade	75649010	Vegetable soup, canned, prepared with water or ready-to-serve
4195	15-0344	A-15-0344		Vegetable stir fry mix, fried in corn oil	15.04.00	Vegetables - Other	75311000	Mixed vegetables, cooked, NS as to form, NS as to fat added in cooking

4196	15-0345	A-15-0345		Vegetable stir fry mix, fried in sunflower oil	15.04.00	Vegetables - Other	75311000	Mixed vegetables, cooked, NS as to form, NS as to fat added in cooking
4197	15-0346	A-15-0346		Vegetable stir fry mix, fried in vegetable oil	15.04.00	Vegetables - Other	75311000	Mixed vegetables, cooked, NS as to form, NS as to fat added in cooking
4198	A-00-06302			Vegetables and potatoes composite 50	15.04.00	Vegetables - Other	75311000	Mixed vegetables, cooked, NS as to form, NS as to fat added in cooking
4199	A-00-06301			Vegetables composite 50	15.04.00	Vegetables - Other	75311000	Mixed vegetables, cooked, NS as to form, NS as to fat added in cooking
4200	A-00-01119			Vegetables, Mixed, Canned or Frozen	15.04.00	Vegetables - Other	75311000	Mixed vegetables, cooked, NS as to form, NS as to fat added in cooking
4201	00-03589	A-00-03589		Vegetables, mixed, canned or frozen, unsalted	15.04.00	Vegetables - Other	75311000	Mixed vegetables, cooked, NS as to form, NS as to fat added in cooking
4202	00-05883			Vegetables, stir fried in specified fat	15.04.00	Vegetables - Other	75311000	Mixed vegetables, cooked, NS as to form, NS as to fat added in cooking
4203	00-05619			Vegetarian meatballs, soya based, baked	15.04.00	Vegetables - Other	41811800	Meatball, meatless
4204	A-00-09688	00-09688		Vegetarian sausages, oven baked	15.04.00	Vegetables - Other	41810400	Breakfast link, pattie, or slice, meatless
4205	A-00-09689			Vegetarian sausages, shallow fried	15.04.00	Vegetables - Other	41810400	Breakfast link, pattie, or slice, meatless

4206	00-05874			Venison burgers	10.04.00	Meat - red - Other red meat, e.g. rabbit, venison	23321000	Venison/deer, NFS
4207	A-00-00353			Venison, Roast	10.04.00	Meat - red - Other red meat, e.g. rabbit, venison	23321100	Venison/deer, roasted
4208	A-18-0391	18-0391		Venison, roasted	10.04.00	Meat - red - Other red meat, e.g. rabbit, venison	23321100	Venison/deer, roasted
4209	A-00-00914			Vermouth, Dry	27.01.02	Beverages - Alcohol - Fortified wine	93402000	Wine, dessert, sweet
4210	A-00-00915			Vermouth, Sweet	27.01.02	Beverages - Alcohol - Fortified wine	93402000	Wine, dessert, sweet
4211	17-0239	A-17-0239		Vermouth, dry	27.01.02	Beverages - Alcohol - Fortified wine	93402000	Wine, dessert, sweet
4212	17-0240	A-17-0240		Vermouth, sweet	27.01.02	Beverages - Alcohol - Fortified wine	93402000	Wine, dessert, sweet
4213	A-12-0260			Very low fat spread	08.04.03	Fats - Plant based fats (solid) - Low fat	81102000	Margarine, NFS
4214	A-00-01032			Victoria Sponge, with Butter Icing	04.02.00	Sweet cereal products - Pastries, Buns & Pies	53123070	Cake, shortcake, sponge type, with whipped cream and fruit
4215	00-03145	A-00-03145		Viennesse whirl (Lyons)	04.02.00	Sweet cereal products - Pastries, Buns & Pies	53118200	Cake, sponge, with icing or filling
4216	A-00-00967	17-0339	A-17-0339	Vinegar	21.03.00	Sauces & accompaniment - Other sauces, incl. brown sauce, soy sauce, ketchup, mint sauce, vinegar	64401000	Vinegar
4217	A-00-09635			Virt fat free alternative ice cream	06.04.02	Dairy products - Ice cream & dairy desserts - reduced fat products	13160420	Fat free ice cream, NS as to flavor
4218	A-00-09655			Virt fat free yoghurt twinpot+fruit	06.03.02	Dairy products - Yoghurt & drinking yoghurts, incl. buttermilk and probiotics - reduced or low fat products	11433000	Yogurt, nonfat milk, fruit

4219	00-09646	A-00-09646		Virt fat free yoghurt, natural	06.03.02	Dairy products - Yoghurt & drinking yoghurts, incl. buttermilk and probiotics - reduced or low fat products	11400000	Yogurt, NFS
4220	A-00-09647	00-09647		Virt fat free yoghurt, with fruit	06.03.02	Dairy products - Yoghurt & drinking yoghurts, incl. buttermilk and probiotics - reduced or low fat products	11433000	Yogurt, nonfat milk, fruit
4221	00-03133	A-00-03133		Vitalinea Chocolate fruit bar	24.01.00	Confectionary - Chocolate based products	91739010	Raisins, chocolate covered
4222	A-11-0094			Vitbe, average	03.03.00	Breads - Brown/Granary/Wheatgerm	51301010	Bread, wheat or cracked wheat
4223	00-05439			Vogel's Soy and Linseed Sandwich	03.04.00	Breads - Other bread	51601020	Bread, multigrain
4224	00-03657			WALKERS Lites crisps (RISCK-LA)	25.01.00	Savoury Snacks - Potato based snacks	71200010	Potato chips, NFS
4225	02-08183			WEETABIX CRUNCHY BRAN - PREVIOUSLY ALPEN CRUNCHY BRAN	02.02.00	Breakfast cereals - Other breakfast cereals - high fibre (equal or >3g/40g portion)	57100100	Cereal, ready-to-eat, NFS
4226	02-08410			WEETABIX OATIFLAKES WITHOUT FRUIT	02.01.00	Breakfast cereals - Oat based cereals	57000100	Cereal, oat, NFS
4227	02-10536			WEETABIX OATY BARS	04.05.00	Sweet cereal products - Cereal bars	53712100	Cereal or Granola bar, NFS
4228	02-10298			WEIGHT WATCHERS CHICKEN IN TOMATO SAUCE WITH POTATO WEDGES	11.01.00	Meat - white - Chicken & turkey & dishes	27348100	Chicken fricassee, Puerto Rican style
4229	02-10347			WEIGHT WATCHERS SAUSAGES IN CIDER GRAVY WITH VEGETABLE MASH READY MEAL	13.00.00	Sausages & burgers & kebab	25221406	Pork sausage, reduced fat
4230	01-09205			WHEY PROTEIN POWDERS ANY FLAVOUR	30.00.00	Nutrition Powders & drinks	95230000	Nutritional powder mix, whey based, NFS

4231	02-08142			WHITE AND WHOLEMEAL BREAD ROLLS	03.04.00	Breads - Other bread	51320010	Roll, wheat or cracked wheat
4232	00-05697			WHITE AND WHOLEMEAL BREAD, TOASTED EG. Best of Both or 50/50	03.03.00	Breads - Brown/Granary/Wheatgerm	51301020	Bread, wheat or cracked wheat, toasted
4233	00-05696			WHITE AND WHOLEMEAL BREADS EG. Best of Both, Kingsmill 50/50	03.03.00	Breads - Brown/Granary/Wheatgerm	51301010	Bread, wheat or cracked wheat
4234	02-00698			WHOLE MILK AFTER BOILING	05.03.00	Milk - Whole milk	11100000	Milk, NFS
4235	02-03172			WHOLEMEAL BREAD, SLIMMERS, TOASTED	03.02.00	Breads - Wholemeal	51301520	Bread, wheat or cracked wheat, reduced calorie and/or high fiber, toasted
4236	02-00174			WHOLEMEAL MUFFINS TOASTED	04.02.00	Sweet cereal products - Pastries, Buns & Pies	51303030	Muffin, English, whole wheat
4237	02-10183			WHOLEMILK FRUIT YOGURT/YOGHURT WITH ADDED FIBRE	06.03.01	Dairy products - Yoghurt & drinking yoghurts, incl. buttermilk and probiotics - full fat products	11431000	Yogurt, whole milk, fruit
4238	02-08150			WINE ALCOHOL FREE EG BLUSH	27.02.03	Beverages - Fruit based drinks - Squashes & fruit concentrates	91301050	Fruit syrup
4239	02-02682			WINE OR SHERRY AFTER COOKING IN STEWS	27.01.01	Beverages - Alcohol - Wine	93401010	Wine, table, red
4240	02-02278			WISPA	24.01.00	Confectionary - Chocolate based products	91705010	Milk chocolate candy, plain
4241	00-01754			WW Vanilla ice cream	06.04.02	Dairy products - Ice cream & dairy desserts - reduced fat products	13130100	Light ice cream, NS as to flavor
4242	00-05751			Wafer Sandwich Biscuit cream filled, fortified e.g RIVINGTON PINK PANTHER	04.01.00	Sweet cereal products - Biscuits	53242000	Cookie, sugar wafer
4243	00-09599	A-00-09599		Wafer biscuits, chocolate coated	04.01.00	Sweet cereal products - Biscuits	53209010	Cookie, sugar wafer, chocolate-covered

4244	11-0186	A-11-0186		Wafer biscuits, filled	04.01.00	Sweet cereal products - Biscuits	53242000	Cookie, sugar wafer
4245	A-00-09951			Wafer biscuits, filled (MW6 Vit Eq)	04.01.00	Sweet cereal products - Biscuits	53242000	Cookie, sugar wafer
4246	A-11-0276	11-0276		Waffles	04.02.00	Sweet cereal products - Pastries, Buns & Pies	55200010	Waffle, NFS
4247	00-05628			Waitrose wafer thin smoked ham	12.01.00	Processed meat - Bacon & ham	22311500	Ham, smoked or cured, canned, NS as to fat eaten
4248	00-05841			Walkers Salt n Shake crisps, salt added	25.01.00	Savoury Snacks - Potato based snacks	71200010	Potato chips, NFS
4249	00-05842			Walkers Salt n Shake crisps, salt not added	25.01.00	Savoury Snacks - Potato based snacks	71200010	Potato chips, NFS
4250	00-05688			Walkers Sunbites	25.01.00	Savoury Snacks - Potato based snacks	54420210	Multigrain chips (Sun Chips)
4251	00-05571			Walnut bread (e.g Tesco)	03.04.00	Breads - Other bread	52403000	Bread, nut
4252	17-0047	A-17-0047		Walnut oil	08.02.00	Fats - Oils	82108700	Walnut oil
4253	14-0850	A-00-00839	A-14-0850	Walnuts	19.00.00	Nuts & Seeds (incl. peanut butter)	42116000	Walnuts, NFS
4254	A-00-00840			Walnuts (Weighed with Shells)	19.00.00	Nuts & Seeds (incl. peanut butter)	42116000	Walnuts, NFS
4255	A-00-01163			Water	27.04.00	Beverages - Water (still, tap, sparkling, flavoured)	94000100	Water, tap
4256	A-00-05123			Water Chestnuts, Canned, Drained, Solids only	15.04.00	Vegetables - Other	75235000	Water chestnut
4257	A-11-0187	11-0187		Water biscuits	25.04.00	Savoury Snacks - Savoury biscuits & crackers	54336000	Crackers, water
4258	A-00-06057			Water biscuits 50	25.04.00	Savoury Snacks - Savoury biscuits & crackers	54336000	Crackers, water
4259	A-13-0395	13-0395		Water chestnuts, canned, drained	15.04.00	Vegetables - Other	75235000	Water chestnut
4260	00-05770			Water to make up hot, instant beverages	27.04.00	Beverages - Water (still, tap, sparkling, flavoured)	94000100	Water, tap
4261	A-00-03841	00-03841		Water with a hint	27.04.00	Beverages - Water (still, tap, sparkling, flavoured)	94000100	Water, tap

4262	00-01834			Water, Cambridge tap, 1999	27.04.00	Beverages - Water (still, tap, sparkling, flavoured)	94000100	Water, tap
4263	00-03869	A-00-03869		Water, tap, average of nation	27.04.00	Beverages - Water (still, tap, sparkling, flavoured)	94000100	Water, tap
4264	00-03866	A-00-03866		Water, tonic, low cal (slim line)	27.04.00	Beverages - Water (still, tap, sparkling, flavoured)	94000100	Water, tap
4265	A-00-00672			Watercress, Raw	15.03.00	Vegetables - Yellow & red & dark green leafy vegetables	72130100	Watercress, raw
4266	13-0396			Watercress, raw	15.03.00	Vegetables - Yellow & red & dark green leafy vegetables	72130100	Watercress, raw
4267	A-00-09940			Watercress, raw (MW6 folate)	15.03.00	Vegetables - Yellow & red & dark green leafy vegetables	72130100	Watercress, raw
4268	A-11-0154	A-00-00057		Weetabix	02.02.00	Breakfast cereals - Other breakfast cereals - high fibre (equal or >3g/40g portion)	57410000	Cereal (Weetabix Whole Grain)
4269	A-00-06005			Weetabix 50	02.02.00	Breakfast cereals - Other breakfast cereals - high fibre (equal or >3g/40g portion)	57410000	Cereal (Weetabix Whole Grain)
4270	00-03001	A-00-03001		Weetabix Minis Fruit and Nut crisp, formerly Fruitibix	02.03.00	Breakfast cereals - Other breakfast cereals - low fibre	57100100	Cereal, ready-to-eat, NFS
4271	00-05714			Weetabix Weetaflakes with Raisin, cranberry and apple	02.03.00	Breakfast cereals - Other breakfast cereals - low fibre	57100100	Cereal, ready-to-eat, NFS
4272	00-03052	A-00-03052		Weetabix, Weetabix	02.02.00	Breakfast cereals - Other breakfast cereals - high fibre (equal or >3g/40g portion)	57410000	Cereal (Weetabix Whole Grain)
4273	A-11-0155	11-0155		Weetaflake	02.02.00	Breakfast cereals - Other breakfast cereals - high fibre (equal or >3g/40g portion)	57406100	Cereal (General Mills Total)
4274	A-00-03053			Weetaflake 'N' Raisin, Weetabix	02.02.00	Breakfast cereals - Other breakfast cereals - high fibre (equal or >3g/40g portion)	57332050	Cereal (General Mills Total Raisin Bran)

4275	A-11-0156			Weetaflake 'n' raisin	02.02.00	Breakfast cereals - Other breakfast cereals - high fibre (equal or >3g/40g portion)	57332050	Cereal (General Mills Total Raisin Bran)
4276	A-11-0157			Weetos	02.02.00	Breakfast cereals - Other breakfast cereals - high fibre (equal or >3g/40g portion)	57100100	Cereal, ready-to-eat, NFS
4277	A-00-03054			Weetos, Weetabix	02.03.00	Breakfast cereals - Other breakfast cereals - low fibre	57100100	Cereal, ready-to-eat, NFS
4278	00-05678			Weight Watchers White Bread	03.01.00	Breads - White	51122000	Bread, reduced calorie and/or high fiber, white or NFS
4279	A-00-03603			Weight Watchers, Tuna, Pasta, Pineapple	01.02.00	Cereals & cereal dishes - Pasta & pasta dishes	27350080	Tuna noodle casserole with vegetables, cream or white sauce
4280	A-00-01048			Welsh Cheesecake, Bakewell Tart (Pastry with Sponge and Jam)	04.02.00	Sweet cereal products - Pastries, Buns & Pies	53452100	Pastry, fruit-filled
4281	A-00-00181			Welsh Rarebit	06.02.00	Dairy products - Cheese, incl. cottage cheese	14630300	Welsh rarebit
4282	A-11-0218	11-0218		Welsh cakes	04.02.00	Sweet cereal products - Pastries, Buns & Pies	52105200	Scone, with fruit
4283	A-00-06064			Welsh cakes 50	04.02.00	Sweet cereal products - Pastries, Buns & Pies	52105200	Scone, with fruit
4284	A-11-0277	11-0277		Welsh cheesecakes	04.02.00	Sweet cereal products - Pastries, Buns & Pies	53452100	Pastry, fruit-filled
4285	A-00-06082			Welsh cheesecakes 50	04.02.00	Sweet cereal products - Pastries, Buns & Pies	53452100	Pastry, fruit-filled
4286	12-0294	A-12-0294		Welsh rarebit	06.02.00	Dairy products - Cheese, incl. cottage cheese	14630300	Welsh rarebit
4287	A-12-0295	12-0295		Welsh rarebit, wholemeal	06.02.00	Dairy products - Cheese, incl. cottage cheese	14630300	Welsh rarebit
4288	A-00-00207			Wheat Germ Oil	08.02.00	Fats - Oils	82109000	Wheat germ oil
4289	17-0151	A-17-0151		Wheat crunchies	25.02.00	Savoury Snacks - Cereal based snacks	54401055	Cheese flavored corn snacks

4290	11-0028			Wheat flour, brown	01.04.00	Cereals & cereal dishes - Other cereals & dishes	20080	Wheat flour, whole-grain
4291	11-0031	A-11-0031		Wheat flour, white, plain	01.04.00	Cereals & cereal dishes - Other cereals & dishes	20081	Wheat flour, white, all-purpose, enriched, bleached
4292	A-11-0033	11-0033		Wheat flour, wholemeal	01.04.00	Cereals & cereal dishes - Other cereals & dishes	20080	Wheat flour, whole-grain
4293	11-0034	A-11-0034		Wheatgerm	01.04.00	Cereals & cereal dishes - Other cereals & dishes	57412000	Wheat germ, plain
4294	A-11-0098			Wheatgerm bread, average	03.03.00	Breads - Brown/Granary/Wheatgerm	51301010	Bread, wheat or cracked wheat
4295	00-09562	A-00-09562		Wheatgerm bread, average, Hovis, Vitbe	03.03.00	Breads - Brown/Granary/Wheatgerm	51301010	Bread, wheat or cracked wheat
4296	A-00-00539			Whelks, Boiled	09.03.00	Fish & fish dishes - Shellfish	26321110	Snails, cooked, NS as to cooking method
4297	A-00-00540			Whelks, Boiled (Weighed with Shell)	09.03.00	Fish & fish dishes - Shellfish	26321110	Snails, cooked, NS as to cooking method
4298	A-16-0268			Whelks, boiled	09.03.00	Fish & fish dishes - Shellfish	26321110	Snails, cooked, NS as to cooking method
4299	00-10238			Whey Protein Isolate ULTIMATE NUTRITION	30.00.00	Nutrition Powders & drinks	95230000	Nutritional powder mix, whey based, NFS
4300	A-00-01102			White Fish, Fried, Coated in Flour	09.01.00	Fish & fish dishes - White fish, incl. tuna	26109140	Cod, coated, fried, made with oil
4301	A-00-01101			White Fish, Fried, No Coating	09.01.00	Fish & fish dishes - White fish, incl. tuna	26109120	Cod, baked or broiled, made with oil
4302	A-00-00419			White Pudding	10.03.00	Meat - red - Pork & dishes	27520520	Pork sandwich
4303	A-00-01249			White Radish	15.04.00	Vegetables - Other	75125000	Radish, raw
4304	A-00-00934			White Sauce, Savoury	21.03.00	Sauces & accompaniment - Other sauces, incl. brown sauce, soy sauce, ketchup, mint sauce, vinegar	13411000	White sauce, milk sauce

4305	A-00-00935			White Sauce, Sweet	21.02.00	Sauces & accompaniment - Cooking sauces, incl. gravies, pesto, cooking sauces for pasta and rice dishes	13411000	White sauce, milk sauce
4306	00-03323	A-00-03323		White bread fried in known fat	03.01.00	Breads - White	51101000	Bread, white
4307	00-03337			White bread sliced SAINSBURYS (RISCK-LA)	03.01.00	Breads - White	51101000	Bread, white
4308	A-11-0107			White bread, French stick	03.01.00	Breads - White	51107010	Bread, French or Vienna
4309	A-11-0099	11-0099		White bread, average	03.01.00	Breads - White	51101000	Bread, white
4310	A-11-0105			White bread, fried	03.01.00	Breads - White	51101000	Bread, white
4311	A-00-09593	00-09593		White bread, fried in blended oil	03.01.00	Breads - White	51101000	Bread, white
4312	00-09594	A-00-09594		White bread, fried in lard	03.01.00	Breads - White	51101000	Bread, white
4313	11-0100			White bread, large, crusty	03.01.00	Breads - White	51107010	Bread, French or Vienna
4314	00-09551	A-00-09551		White bread, sliced	03.01.00	Breads - White	51101000	Bread, white
4315	A-00-09552	00-09552	A-11-0106	White bread, toasted	03.01.00	Breads - White	51101010	Bread, white, toasted
4316	A-00-06257			White fish, Fried, No coating (dripping) 50	09.01.00	Fish & fish dishes - White fish, incl. tuna	26109120	Cod, baked or broiled, made with oil
4317	19-0159	A-19-0159		White pudding	10.03.00	Meat - red - Pork & dishes	27520520	Pork sandwich
4318	11-0043			White rice, easy cook, boiled	01.03.00	Cereals & cereal dishes - Rice & rice dishes	56205001	Rice, white, cooked, NS as to fat added in cooking
4319	A-00-09932			White rice, easy cook, boiled (MW6 folate)	01.03.00	Cereals & cereal dishes - Rice & rice dishes	56205001	Rice, white, cooked, NS as to fat added in cooking
4320	A-11-0045			White rice, fried	01.03.00	Cereals & cereal dishes - Rice & rice dishes	58150310	Rice, fried, NFS
4321	A-11-0050			White rice, polished, boiled	01.03.00	Cereals & cereal dishes - Rice & rice dishes	56205001	Rice, white, cooked, NS as to fat added in cooking
4322	A-11-0123	A-00-09563	00-09563	White rolls, crusty	03.01.00	Breads - White	51153000	Roll, white, hard
4323	A-00-09564	00-09564	A-11-0124	White rolls, soft	03.01.00	Breads - White	51150000	Roll, white, soft
4324	12-0302			White sauce packet mix	21.02.00	Sauces & accompaniment - Cooking sauces, incl. gravies, pesto, cooking sauces for pasta and rice dishes	6957	Gravy, brown instant, dry

4325	A-12-0297			White sauce, savoury, made with semi-skimmed milk	21.02.00	Sauces & accompaniment - Cooking sauces, incl. gravies, pesto, cooking sauces for pasta and rice dishes	13411000	White sauce, milk sauce
4326	A-12-0298			White sauce, savoury, made with skimmed milk	21.02.00	Sauces & accompaniment - Cooking sauces, incl. gravies, pesto, cooking sauces for pasta and rice dishes	13411000	White sauce, milk sauce
4327	A-12-0296			White sauce, savoury, made with whole milk	21.02.00	Sauces & accompaniment - Cooking sauces, incl. gravies, pesto, cooking sauces for pasta and rice dishes	13411000	White sauce, milk sauce
4328	A-12-0300			White sauce, sweet, made with semi-skimmed milk	21.02.00	Sauces & accompaniment - Cooking sauces, incl. gravies, pesto, cooking sauces for pasta and rice dishes	13411000	White sauce, milk sauce
4329	A-12-0299			White sauce, sweet, made with whole milk	21.02.00	Sauces & accompaniment - Cooking sauces, incl. gravies, pesto, cooking sauces for pasta and rice dishes	13411000	White sauce, milk sauce
4330	00-09839	A-00-09839		White sauce,savoury, semi-skimmed milk	21.02.00	Sauces & accompaniment - Cooking sauces, incl. gravies, pesto, cooking sauces for pasta and rice dishes	13411000	White sauce, milk sauce
4331	00-09840	A-00-09840		White sauce,savoury, skimmed milk	21.02.00	Sauces & accompaniment - Cooking sauces, incl. gravies, pesto, cooking sauces for pasta and rice dishes	13411000	White sauce, milk sauce
4332	A-00-09838	00-09838		White sauce,savoury, whole milk	21.02.00	Sauces & accompaniment - Cooking sauces, incl. gravies, pesto, cooking sauces for pasta and rice dishes	13411000	White sauce, milk sauce

4333	00-09842	A-00-09842		White sauce,sweet, semi-skimmed milk	21.02.00	Sauces & accompaniment - Cooking sauces, incl. gravies, pesto, cooking sauces for pasta and rice dishes	13411000	White sauce, milk sauce
4334	A-00-09843			White sauce,sweet, skimmed milk	21.02.00	Sauces & accompaniment - Cooking sauces, incl. gravies, pesto, cooking sauces for pasta and rice dishes	13411000	White sauce, milk sauce
4335	00-03925	A-00-03925		White wine sauce, homemade	21.02.00	Sauces & accompaniment - Cooking sauces, incl. gravies, pesto, cooking sauces for pasta and rice dishes	28500010	Gravy, meat or poultry, with wine
4336	A-17-0230	17-0230		White wine, dry	27.01.01	Beverages - Alcohol - Wine	93401010	Wine, table, red
4337	A-17-0231	17-0231		White wine, medium	27.01.01	Beverages - Alcohol - Wine	93401010	Wine, table, red
4338	17-0232	A-17-0232		White wine, sparkling	27.01.01	Beverages - Alcohol - Wine	93401010	Wine, table, red
4339	17-0233	A-17-0233		White wine, sweet	27.01.01	Beverages - Alcohol - Wine	93401010	Wine, table, red
4340	A-00-00509			Whitebait, Fried	09.02.00	Fish & fish dishes - Oily fish	26139110	Sardines, cooked
4341	16-0231			Whitebait, in flour, fried	09.02.00	Fish & fish dishes - Oily fish	26139110	Sardines, cooked
4342	A-00-00475			Whiting, Fried	09.01.00	Fish & fish dishes - White fish, incl. tuna	26157120	Whiting, baked or broiled, made with oil
4343	A-00-06255			Whiting, Fried (dripping) 50	09.01.00	Fish & fish dishes - White fish, incl. tuna	26157120	Whiting, baked or broiled, made with oil
4344	A-00-00477			Whiting, Steamed	09.01.00	Fish & fish dishes - White fish, incl. tuna	26157160	Whiting, steamed or poached
4345	A-00-00478			Whiting, Steamed (Weighed with Bones)	09.01.00	Fish & fish dishes - White fish, incl. tuna	26157160	Whiting, steamed or poached
4346	A-16-0162			Whiting, in crumbs, fried in blended oil	09.01.00	Fish & fish dishes - White fish, incl. tuna	26157140	Whiting, coated, fried, made with oil
4347	16-0160	A-16-0160		Whiting, steamed	09.01.00	Fish & fish dishes - White fish, incl. tuna	26157160	Whiting, steamed or poached
4348	18-0341	A-18-0341		Whole chicken, roasted, meat and skin	11.01.00	Meat - white - Chicken & turkey & dishes	24102010	Chicken, NS as to part, baked, broiled, or roasted, skin eaten

4349	00-05828			Whole milk yogurt with nuts eg Longley farm	06.03.01	Dairy products - Yoghurt & drinking yoghurts, incl. buttermilk and probiotics - full fat products	11434100	Yogurt, whole milk, flavors other than fruit
4350	A-12-0186	12-0186		Whole milk yogurt, 'organic'	06.03.01	Dairy products - Yoghurt & drinking yoghurts, incl. buttermilk and probiotics - full fat products	11411100	Yogurt, whole milk, plain
4351	00-09654	A-12-0185	A-00-09654	Whole milk yogurt, fruit	06.03.01	Dairy products - Yoghurt & drinking yoghurts, incl. buttermilk and probiotics - full fat products	11431000	Yogurt, whole milk, fruit
4352	A-12-0187	12-0187		Whole milk yogurt, goats	06.03.01	Dairy products - Yoghurt & drinking yoghurts, incl. buttermilk and probiotics - full fat products	11411100	Yogurt, whole milk, plain
4353	12-0184	A-12-0184		Whole milk yogurt, plain	06.03.01	Dairy products - Yoghurt & drinking yoghurts, incl. buttermilk and probiotics - full fat products	11411100	Yogurt, whole milk, plain
4354	A-00-09743	00-09743	A-12-0016	Whole milk, UHT	05.03.00	Milk - Whole milk	11100000	Milk, NFS
4355	A-12-0014			Whole milk, pasteurised, summer	05.03.00	Milk - Whole milk	11100000	Milk, NFS
4356	A-12-0015			Whole milk, pasteurised, winter	05.03.00	Milk - Whole milk	11100000	Milk, NFS
4357	A-00-09794	00-09794		Whole milk, pasteurized	05.03.00	Milk - Whole milk	11100000	Milk, NFS
4358	00-09745	A-00-09745		Whole milk, sterilised	05.03.00	Milk - Whole milk	11100000	Milk, NFS
4359	A-00-09795	00-09795		Whole milk, summer (May-Oct)	05.03.00	Milk - Whole milk	11100000	Milk, NFS
4360	00-09796	A-00-09796		Whole milk, winter (Nov-April)	05.03.00	Milk - Whole milk	11100000	Milk, NFS
4361	18-0367			Whole turkey, roasted	11.01.00	Meat - white - Chicken & turkey & dishes	24201330	Turkey, light and dark meat, roasted, skin eaten

4362	A-00-03325	00-03325		Wholemeal Muffin with fruit & honey (Tesco)	04.02.00	Sweet cereal products - Pastries, Buns & Pies	51303070	Muffin, English, whole wheat, with raisins
4363	00-03338			Wholemeal bread SAINSBURYS (RISCK-LA)	03.02.00	Breads - Wholemeal	51300110	Bread, whole wheat
4364	A-11-0113	A-00-09556	00-09556	Wholemeal bread, average	03.02.00	Breads - Wholemeal	51300110	Bread, whole wheat
4365	00-09557	A-00-09557	A-11-0117	Wholemeal bread, toasted	03.02.00	Breads - Wholemeal	51300120	Bread, whole wheat, toasted
4366	11-0188	A-11-0188		Wholemeal crackers	25.04.00	Savoury Snacks - Savoury biscuits & crackers	54420210	Multigrain chips (Sun Chips)
4367	A-00-06020			Wholemeal crackers 50	25.04.00	Savoury Snacks - Savoury biscuits & crackers	54420210	Multigrain chips (Sun Chips)
4368	00-03342			Wholemeal multigrain bread SAINSBURYS (RISCK-LA)	03.02.00	Breads - Wholemeal	51601020	Bread, multigrain
4369	A-11-0229	11-0229		Wholemeal pastry, cooked	01.04.00	Cereals & cereal dishes - Other cereals & dishes	52104040	Biscuit, whole wheat
4370	A-00-06066			Wholemeal pastry, cooked 50	04.02.00	Sweet cereal products - Pastries, Buns & Pies	53391100	Pie shell, graham cracker
4371	A-00-09565	00-09565		Wholemeal roll	03.02.00	Breads - Wholemeal	51320500	Roll, whole wheat
4372	A-11-0125			Wholemeal rolls	03.02.00	Breads - Wholemeal	51320500	Roll, whole wheat
4373	A-17-0287	17-0287		Wholesoup, canned	20.01.00	Soups - Canned & fresh & homemade	41603010	Lentil soup, home recipe, canned, or ready-to-serve
4374	A-00-00904			Wine, Red	27.01.01	Beverages - Alcohol - Wine	93401010	Wine, table, red
4375	A-00-00905			Wine, Rose, Medium	27.01.01	Beverages - Alcohol - Wine	93401010	Wine, table, red
4376	A-00-00906			Wine, White, Dry	27.01.01	Beverages - Alcohol - Wine	93401010	Wine, table, red
4377	A-00-00907			Wine, White, Medium	27.01.01	Beverages - Alcohol - Wine	93401010	Wine, table, red
4378	A-00-00909			Wine, White, Sparkling	27.01.01	Beverages - Alcohol - Wine	93401010	Wine, table, red
4379	A-00-00908			Wine, White, Sweet	27.01.01	Beverages - Alcohol - Wine	93401010	Wine, table, red
4380	A-00-00541			Winkles, Boiled	09.03.00	Fish & fish dishes - Shellfish	26321110	Snails, cooked, NS as to cooking method

4381	A-17-0340	17-0340		Worcestershire sauce	21.03.00	Sauces & accompaniment - Other sauces, incl. brown sauce, soy sauce, ketchup, mint sauce, vinegar	41420450	Worcestershire sauce
4382	A-00-01255			Wotsits, Golden Wonder	25.02.00	Savoury Snacks - Cereal based snacks	54401055	Cheese flavored corn snacks
4383	02-10434			YOGURT AND FROMAGE FRAIS MOUSSE WITH FRUIT AND CREAM	06.04.01	Dairy products - Ice cream & dairy desserts - full fat products	11431000	Yogurt, whole milk, fruit
4384	02-03223			YOGURT AND FROMAGE FRAIS MOUSSE, LOW FAT E.G. ONKEN LITE MOUSSE	06.04.02	Dairy products - Ice cream & dairy desserts - reduced fat products	11460150	Yogurt, frozen, NS as to flavor, lowfat milk
4385	02-07885			YOGURT COATED PEANUTS/RAISINS	19.00.00	Nuts & Seeds (incl. peanut butter)	91731150	Peanuts, yogurt covered
4386	02-09390			YOGURT DRESSINGS PURCHASED	21.01.00	Sauces & accompaniment - Dressings & Mayonnaise	83115000	Yogurt dressing
4387	02-07755			YOGURT DRINK CONTAINING FRUIT PUREE	06.03.02	Dairy products - Yoghurt & drinking yoghurts, incl. buttermilk and probiotics - reduced or low fat products	11436000	Yogurt, liquid
4388	02-07757			YOGURT ICE CREAM	06.04.02	Dairy products - Ice cream & dairy desserts - reduced fat products	11459990	Yogurt, frozen, NS as to flavor, NS as to type of milk
4389	02-09272			YOGURT, VIRTUALLY FAT FREE, ANY OTHER FLAVOUR, WITH ARTIFICAL SWEETENER	06.03.02	Dairy products - Yoghurt & drinking yoghurts, incl. buttermilk and probiotics - reduced or low fat products	11400000	Yogurt, NFS
4390	02-02701			YOGURT, VIRTUALLY FAT FREE, FRUIT WITH ARTIFICIAL SWEETNER	06.03.02	Dairy products - Yoghurt & drinking yoghurts, incl. buttermilk and probiotics - reduced or low fat products	11433000	Yogurt, nonfat milk, fruit

4391	02-05361			YOGURT, WHOLE MILK, WITH ADDED SUGAR, NO FRUIT	06.03.01	Dairy products - Yoghurt & drinking yoghurts, incl. buttermilk and probiotics - full fat products	11434100	Yogurt, whole milk, flavors other than fruit
4392	00-05685			YOGURTS, FRUIT WITH ADDED CREAM	06.03.01	Dairy products - Yoghurt & drinking yoghurts, incl. buttermilk and probiotics - full fat products	11430000	Yogurt, NS as to type of milk, fruit
4393	02-08365			YORKSHIRE PUDDING FROZEN	01.04.00	Cereals & cereal dishes - Other cereals & dishes	52311010	Popover
4394	A-00-00674			Yam, Boiled	15.04.00	Vegetables - Other	71945010	Yam, cooked, Puerto Rican
4395	13-0400			Yam, boiled in unsalted water	15.04.00	Vegetables - Other	71945010	Yam, cooked, Puerto Rican
4396	00-03628			Yeast Pate with Mushroom/Mushroom Pate (includes yeast) Tartrex	15.04.00	Vegetables - Other	75219000	Mushrooms, cooked, NS as to form, NS as to fat added in cooking
4397	17-0380			Yeast extract	26.01.00	Miscellaneous - Dried herbs & spices & pastes	43406	Yeast extract spread
4398	A-00-09942			Yeast extract (MW6 folate)	26.01.00	Miscellaneous - Dried herbs & spices & pastes	43406	Yeast extract spread
4399	A-00-00968			Yeast, Baker's, Compressed	26.01.00	Miscellaneous - Dried herbs & spices & pastes	18374	Leavening agents, yeast, baker's, compressed
4400	A-00-00969			Yeast, Dried	26.01.00	Miscellaneous - Dried herbs & spices & pastes	18375	Leavening agents, yeast, baker's, active dry
4401	17-0378			Yeast, bakers, compressed	26.01.00	Miscellaneous - Dried herbs & spices & pastes	18374	Leavening agents, yeast, baker's, compressed
4402	17-0379			Yeast, dried	26.01.00	Miscellaneous - Dried herbs & spices & pastes	18375	Leavening agents, yeast, baker's, active dry
4403	00-05390			Yoghurt, Danone Bio Activia Fibre	06.03.01	Dairy products - Yoghurt & drinking yoghurts, incl. buttermilk and probiotics - full fat products	11400000	Yogurt, NFS

4404	00-03365			Yoghurt, twinpot & crunch or crumble	06.03.01	Dairy products - Yoghurt & drinking yoghurts, incl. buttermilk and probiotics - full fat products	11434090	Yogurt, NS as to type of milk, flavors other than fruit
4405	00-03362			Yoghurt, whole milk non-fruit (toffee)	06.03.01	Dairy products - Yoghurt & drinking yoghurts, incl. buttermilk and probiotics - full fat products	11434100	Yogurt, whole milk, flavors other than fruit
4406	00-03830			Yogurt coated fruit	18.03.00	Fruit - Dried	91739600	Raisins, yogurt covered
4407	A-00-00162			Yogurt, Flavoured	06.03.02	Dairy products - Yoghurt & drinking yoghurts, incl. buttermilk and probiotics - reduced or low fat products	11400000	Yogurt, NFS
4408	A-00-00163			Yogurt, Fruit	06.03.02	Dairy products - Yoghurt & drinking yoghurts, incl. buttermilk and probiotics - reduced or low fat products	11430000	Yogurt, NS as to type of milk, fruit
4409	A-00-00164			Yogurt, Hazelnut	06.03.02	Dairy products - Yoghurt & drinking yoghurts, incl. buttermilk and probiotics - reduced or low fat products	11434090	Yogurt, NS as to type of milk, flavors other than fruit
4410	A-00-00161			Yogurt, Low-fat Natural	06.03.02	Dairy products - Yoghurt & drinking yoghurts, incl. buttermilk and probiotics - reduced or low fat products	11400000	Yogurt, NFS
4411	00-03363			Yogurt. virtually fat free, non-fruit (eg toffee)	06.03.02	Dairy products - Yoghurt & drinking yoghurts, incl. buttermilk and probiotics - reduced or low fat products	11400000	Yogurt, NFS
4412	00-05904			Yoplait Perle de Lait, coconut or lemon flavour ONLY	06.03.01	Dairy products - Yoghurt & drinking yoghurts, incl. buttermilk and probiotics - full fat products	11434090	Yogurt, NS as to type of milk, flavors other than fruit

4413	A-00-00122			Yorkshire Pudding	01.04.00	Cereals & cereal dishes - Other cereals & dishes	52311010	Popover
4414	11-0359	A-11-0359		Yorkshire pudding	01.04.00	Cereals & cereal dishes - Other cereals & dishes	52311010	Popover
4415	A-00-06050			Yorkshire pudding (dripping) 50	01.04.00	Cereals & cereal dishes - Other cereals & dishes	52311010	Popover
4416	A-11-0360			Yorkshire pudding, made with skimmed milk	01.04.00	Cereals & cereal dishes - Other cereals & dishes	52311010	Popover
4417	00-09802	A-00-09802		Yorkshire pudding, with skimmed milk	01.04.00	Cereals & cereal dishes - Other cereals & dishes	52311010	Popover
4418	00-05521			crisps potato, fried in sunseed oil eg Walkers	25.01.00	Savoury Snacks - Potato based snacks	71200010	Potato chips, NFS
4419	13-0829			garam masala	26.01.00	Miscellaneous - Dried herbs & spices & pastes	2010	Spices, cinnamon, ground
