# Supplementary material for: Associations between Diet Quality and Global Cognitive Ability across the Life Course: Longitudinal Analysis of the 1946 British Birth Cohort
Source: Curr Dev Nutr. 2025 Dec 20;10(2):107619. doi: 10.1016/j.cdnut.2025.107619 (PMC12860707; doi:10.1016/j.cdnut.2025.107619)
Supplement: multimedia component 2 [file mmc2.docx]

**Title:** Associations between diet quality and global cognitive ability across the life course: Longitudinal analysis of the 1946 British Birth Cohort

**Authors:** Kelly C. Cara, Tammy M. Scott, Mei Chung, Paul F. Jacques

**Supplemental Materials**

**Table of Contents**

[Table S1. Descriptions for individual cognitive measures in the 1946 British Birth Cohort with results from the analytical cohort 2](#_Toc208919856)

[Table S2. Leisure activities reported at age 11 divided into activity types and classified as social and/or intellectual in nature 5](#_Toc208919857)

[Table S3. Results from group-based trajectory modeling analysis Step 1 for diet quality over time in the analytical cohort 6](#_Toc208919858)

[Table S4. Results from group-based trajectory modeling analysis Step 1 for global cognitive ability scores over time in the analytical cohort 7](#_Toc208919859)

[Table S5. Adequacy of fit measures^1^ for trajectory models selected in Step 1 for diet quality (Model 7) and global cognitive ability (Model 6) in the analytical cohort^2^ 8](#_Toc208919860)

[Figure S1. Spaghetti plot showing individual trajectories by diet trajectory groups in the selected three-group model (*n* = 3,059) 9](#_Toc208919861)

[Figure S2. Scatter plot and predicted trend lines for diet trajectory groups in the selected three-group model (*n* = 3,059) 10](#_Toc208919862)

[Figure S3. Spaghetti plot showing individual trajectories by cognitive trajectory groups in the selected four-group model (*n* = 3,059) 11](#_Toc208919863)

[Figure S4. Scatter plot and predicted trend lines for cognitive trajectory groups in the selected four-group model (*n* = 3,059) 12](#_Toc208919864)

[Figure S5. Mean Healthy Eating Index (HEI)-2020 component scores in the full analytical cohort at each data collection period; Higher scores for moderation items (refined grains, sodium, added sugars, saturated fats) indicate lower intake. 13](#_Toc208919865)

[Figure S6. Joint trajectory model combining the unadjusted diet (top) and cognitive (bottom) trajectory models (n = 3,059); lines = estimated trajectories with 95% CIs, dots = observed group means, legends present expected group membership based on the joint model 14](#_Toc208919866)

**Supplemental Tables**

Table S1. Descriptions for individual cognitive measures in the 1946 British Birth Cohort with results from the analytical cohort

| **Age** | **Cognitive measure** | **Brief description ^1^** | **Points possible or full cohort’s min-max (unit)** | **Sample size, *n*** | **Mean (SD)** | **Median (IQR)** |
| --- | --- | --- | --- | --- | --- | --- |
| 8 | Picture intelligence | 60-item non-verbal reasoning test | 0-60 | 2,933 | 40.68 (9.11) | 42 (12) |
|  | Reading comprehension | Selecting appropriate words to complete 35 sentences | 0-35 | 2,928 | 14.49 (7.65) | 15 (11) |
|  | Word reading | Ability to read and pronounce 50 words | 0-50 | 2,928 | 17.34 (10.18) | 17 (16) |
|  | Vocabulary | Ability to explain the meaning of 50 words | 0-50 | 2,928 | 16.42 (5.93) | 16 (7) |
| 11 | General ability test |  |  |  |  |  |
|  | Verbal | 40-items: Selecting appropriate word to complete a series | 0-40 | 2,824 | 23.96 (9.04) | 25 (14) |
|  | Non-verbal | 40-items: Selecting appropriate image to complete a series | 0-40 | 2,824 | 22.11 (7.36) | 22 (11) |
|  | Arithmetic test | 50-item test on addition, subtraction, multiplication, division | 0-50 | 2,819 | 26.97 (11.58) | 28 (18) |
|  | Word reading | Ability to read and pronounce 50 words | 0-50 | 2,819 | 36.81 (10.13) | 40 (12) |
|  | Vocabulary | Ability to explain the meaning of 50 words | 0-50 | 2,819 | 30.28 (7.34) | 31 (11) |
| 15 | The Alice Heim Group Ability Test (AH4) | Test of series completion, mental arithmetic, vocabulary, and reasoning by analogy |  |  |  |  |
|  | Verbal | 65-items measuring verbal ability | 0-65 | 2,814 | 36.11 (11.08) | 37 (15) |
|  | Non-verbal | 65-items measuring non-verbal ability | 0-65 | 2,815 | 39.06 (10.13) | 39 (14) |
|  | The Watts-Vernon Reading Test | 35-item test of reading comprehension by selecting appropriate words to complete sentences | 0-35 | 2,811 | 24.85 (6.35) | 26 (9) |
|  | Mathematics test | 47-item test of arithmetic, geometry, trigonometry, algebra | 0-47 | 2,812 | 14.83 (10.22) | 13 (17) |
| 26 | The Watts-Vernon Reading Test | Same test given at age 15 with 10 additional items of increased difficulty | 0-45 | 2,775 | 33.02 (8.05) | 33 (13) |
|  |  |  |  |  |  |  |
|  |  |  |  |  |  |  |
|  |  |  |  |  |  |  |
| 43 | Word list recall test | Writing down as many words recalled from a list of 15 words | 0-45 | 2,799 | 24.65 (6.38) | 25 (9) |
|  | Long-term recall ^2^ | Recalling the year, month, and day of the week of last interview and 8 medical measures taken at age 36 interview | 0-11 | 2,733 | 1.77 (1.57) | 2 (3) |
|  | Visual memory | Recalling the contents of 5 images 20 minutes later | 0-5 | 2,934 | 4.42 (0.77) | 5 (1) |
|  | Timed letter search | Crossing out as many target letters as possible in 60 seconds from a block of letters | 25-450 (letters scanned) | 2,877 | 343.24 (76.48) | 313 (122) |
|  | Motor speed and praxis | 10 trials (5 per hand): Moving 10 pegs to an adjacent hole | 77-516 (seconds) | 2,886 | 104.07 (12.36) | 102.8 (14.1) |
| 53 | Word list recall test | Writing down words recalled from 3 lists of 15 words each | 0-45 | 2,579 | 23.86 (6.29) | 24 (8) |
|  | Timed letter search | Crossing out as many target letters as possible in 60 seconds from a block of letters | 64-591 (letters scanned) | 2,615 | 280.79 (75.05) | 287 (98) |
|  | Verbal learning, delayed recall | Recalling words from the verbal learning task after the timed letter search task | 0-15 | 2,047 | 8.43 (2.61) | 8 (3) |
|  | Verbal fluency (animal naming) test | Naming as many different animals as possible in one minute | 1-62 (count) | 2,625 | 23.59 (6.89) | 23 (9) |
|  | Prospective memory | Remembering a series of tasks to complete when given an envelope at a later time | 1-3 | 2,610 | 1.23 (0.55) | 1 (0) |
|  | National Adult Reading Test (NART) | Pronunciation of 50 irregular words of increased difficulty | 0-50 | 2,526 | 34.24 (9.51) | 36 (14) |
|  | Delayed verbal memory ^2^ | Recalling the name and address written in the prospective memory envelope task | No score |  |  |  |
| 60-64 | Word list recall test | Writing down as many words recalled from a list of 15 words | 0-45 | 1,968 | 24.15 (6.09) | 24 (8) |
|  | Timed letter search | Crossing out as many target letters as possible in 60 seconds from a block of letters | 98-591 (letters scanned) | 1,995 | 265.89 (71.79) | 239 (85) |
|  | Verbal learning, delayed recall | Recalling words from the verbal learning task after the timed letter search task | 0-15 | 1,964 | 8.43 (2.58) | 8 (3) |
|  | Reaction time test | 60 trials: Pressing a key as quickly as possible when certain numbers appeared on a screen | 41-849 (mean speed) | 1,982 | 286.32 (68.69) | 269.5 (63) |
| 68-69 | Word list recall test | Writing down as many words recalled from a list of 15 words | 0-45 | 1,871 | 22.11 (6.09) | 22 (8) |
|  | Timed letter search | Crossing out as many target letters as possible in 60 seconds from a block of letters | 60-591 (letters scanned) | 1,900 | 261.65 (73.23) | 239 (85) |
|  | Finger tapping test | Tapping as fast as possible with index finger |  |  |  |  |
|  | Left hand | Number of taps in 10 seconds | 4-88 (count) | 1,851 | 45.95 (11.25) | 46 (14) |
|  | Right hand | Number of taps in 10 seconds | 9-87 (count) | 1,850 | 48.72 (11.72) | 49 (15) |
|  | Addenbrooke's Cognitive Examination (ACE)-III | Test of 5 domains to detect mild dementia and distinguish between Alzheimer's Disease and Frontotemporal dementia | 0-100 | 1,591 | 91.54 (5.97) | 93 (8) |
|  | Attention/orientation | 18 items: orientation to time and place, repeating words, counting backwards | 0-18 | 1,612 | 16.75 (1.80) | 17 (2) |
|  | Memory | 26 items: recalling words, memorization, recalling current and former political figures | 0-26 | 1,613 | 23.44 (2.87) | 24 (4) |
|  | Verbal fluency | Naming as many words beginning with a specified letter as possible in one minute | 0-14 | 1,892 | 10.99 (2.12) | 11 (3) |
|  | Language | Following directions related to actions, images, and words | 0-26 | 1,593 | 25.28 (1.13) | 26 (1) |
|  | Visuospatial skills | Copying, drawing, counting, and identifying letters | 0-16 | 1,606 | 15.05 (1.29) | 15 (1) |
|  | Mini Mental State Examination (MMSE) | MMSE equivalent subset contained within the ACE-III | 0-30 | 1,614 | 26.28 (2.87) | 27 (3) |

IQR, interquartile range

^1^ Descriptions for exams adapted from <https://skylark.ucl.ac.uk/NSHD/docu.php?id=mpreo:topics:childhoodcognitiveability> and <https://closer.ac.uk/cross-study-data-guides/cognitive-measures-guide/nshd-cognition/>.

^2^ Measure was excluded from analyses in the present study.

Table S2. Leisure activities reported at age 11 divided into activity types and classified as social and/or intellectual in nature

|  |  | **Activity type** | | | | |  |
| --- | --- | --- | --- | --- | --- | --- | --- |
| **Survey item** | **Answer choices** | **Clubs** | **Education** | **Sports** | **Creative** | **Other hobbies** | **Classification** |
| Clubs | No clubs (except cinema) |  |  |  |  |  | - |
|  | Club member | X |  |  |  |  | Social |
| Hobbies | None |  |  |  |  |  | - |
|  | Academic only |  | X |  |  |  | Intellectual |
|  | Sports and/or outdoor only |  |  | X |  |  | Social |
|  | Academic and sport, etc. |  | X | X |  |  | Intellectual and social |
|  | Arts, craft and domestic only |  |  |  | X |  | Intellectual |
|  | Academic and arts, etc. |  | X |  | X |  | Intellectual |
|  | Sport, etc., and arts, etc. |  |  | X | X |  | Intellectual and social |
|  | All |  | X | X | X |  | Intellectual and social |
|  | Misc only |  |  |  |  | X | Intellectual |
| **Correlation with global cognitive ability, *r_pb_*** | | 0.13 | 0.18 | -0.001 | -0.03 | 0.03 |  |

Table S3. Results from group-based trajectory modeling analysis Step 1 for diet quality over time in the analytical cohort

|  |  |  |  |  | **Participants (*n* = 3,059)** | | |  | **Data points (*n* = 11,430)** | | |  |
| --- | --- | --- | --- | --- | --- | --- | --- | --- | --- | --- | --- | --- |
| **Model ^1^** | **Groups, *n*** | **Polynomial orders** | ***df*** |  | **Log likelihood** | **AIC** | **BIC** |  | **Log likelihood** | **AIC** | **BIC** | **Entropy** |
| **Phase 1: Number of trajectories ^2^** |  |  |  |  |  |  |  |  |  |  |  |  |
| 1 | 2 | 33 | 10 |  | -42364.5 | 84748.95 | 84809.21 |  | -42364.5 | 84748.95 | 84822.39 | 0.628 |
| **2** | **3** | **333** | **15** |  | **-42148.8** | **84327.66** | **84418.05** |  | **-42148.8** | **84327.66** | **84437.82** | **0.599** |
| 3 | 4 | 3333 | 20 |  | -42113.3 | 84266.68 | 84387.19 |  | -42113.3 | 84266.68 | 84413.56 | 0.535 |
| 5 | 6 | 333333 | 30 |  | -42061.5 | 84182.9 | 84363.68 |  | -42061.5 | 84182.9 | 84403.22 | 0.474 |
| **Phase 2: Shape of trajectories ^3^** |  |  |  |  |  |  |  |  |  |  |  |  |
| 6 | 3 | 444 | 18 |  | -42141.7 | 84319.32 | 84427.79 |  | -42141.7 | 84319.32 | 84451.51 | 0.599 |
| **7** | **3** | **222** | **12** |  | **-42154.3** | **84332.56** | **84404.87** |  | **-42154.3** | **84332.56** | **84420.69** | **0.597** |
| 8 | 3 | 221 | 11 |  | -42349.6 | 84721.28 | 84787.57 |  | -42349.6 | 84721.28 | 84802.07 | 0.538 |
| 9 | 3 | 212 | 11 |  | -42254 | 84530.08 | 84596.36 |  | -42254 | 84530.08 | 84610.86 | 0.607 |
| 10 | 3 | 122 | 11 |  | -42254 | 84530.08 | 84596.36 |  | -42254 | 84530.08 | 84610.86 | 0.607 |
| 12 | 3 | 332 | 14 |  | -42150.9 | 84329.76 | 84414.12 |  | -42150.9 | 84329.76 | 84432.57 | 0.599 |
| 13 | 3 | 323 | 14 |  | -42150.9 | 84329.76 | 84414.12 |  | -42150.9 | 84329.76 | 84432.57 | 0.599 |
| 14 | 3 | 233 | 14 |  | -42150.9 | 84329.73 | 84414.09 |  | -42150.9 | 84329.73 | 84432.54 | 0.598 |

AIC, Akaike information criterion; BIC, Bayesian information criterion; *df*, degrees of freedom

^1^ Results are not presented for model 4 (five groups with all cubic orders [33333]) and model 11 (three groups with two quadratic and one cubic order [223]) which produced warnings of a nonsymmetric or high singular variance matrix.

^2^ Phase 1 identified 3 groups as the ideal number of trajectories based on best (lowest) BIC values, less classification uncertainty (entropy > 0.8 as ideal), and interpretability.

^3^ Phase 2 adjusted the polynomial order of trajectories to identify a model with all groups significant at *P* < 0.05, lowest AIC and BIC values, and good fit based on visual inspection of graphs. Based on these criteria, Model 7 was selected.

Table S4. Results from group-based trajectory modeling analysis Step 1 for global cognitive ability scores over time in the analytical cohort

|  |  |  |  |  | **Participants (*n* = 3,059)** | | |  | **Data points (*n* = 18,053)** | | | |  |
| --- | --- | --- | --- | --- | --- | --- | --- | --- | --- | --- | --- | --- | --- |
| **Model #** | **Groups, *n*** | **Polynomial orders** | ***df*** |  | **Log likelihood** | **AIC** | **BIC** |  | **Log likelihood** | **AIC** | **BIC** | **Entropy** | |
| **Phase 1: Number of trajectories ^1^** |  |  |  |  |  |  |  |  |  |  |  |  | |
| 1 | 2 | 33 | 10 |  | -77037.78 | 154095.60 | 154155.80 |  | -77037.78 | 154095.60 | 154173.60 | 0.88 | |
| 2 | 3 | 333 | 15 |  | -76190.43 | 152410.90 | 152501.20 |  | -76190.43 | 152410.90 | 152527.90 | 0.82 | |
| **3** | **4** | **3333** | **20** |  | **-75882.43** | **151804.90** | **151925.40** |  | **-75882.43** | **151804.90** | **151960.90** | **0.77** | |
| 4 | 5 | 33333 | 25 |  | -75634.95 | 151319.90 | 151470.50 |  | -75634.95 | 151319.90 | 151514.90 | 0.76 | |
| 5 | 6 | 333333 | 30 |  | -75392.40 | 150844.80 | 151025.60 |  | -75392.40 | 150844.80 | 151078.80 | 0.76 | |
| **Phase 2: Shape of trajectories ^2^** |  |  |  |  |  |  |  |  |  |  |  |  | |
| **6** | **4** | **4444** | **24** |  | **-75696.18** | **151440.40** | **151585.00** |  | **-75696.18** | **151440.40** | **151627.60** | **0.78** | |
| 7 | 4 | 4434 | 23 |  | -75705.44 | 151456.90 | 151595.50 |  | -75705.44 | 151456.90 | 151636.30 | 0.78 | |
| 8 | 4 | 4334 | 22 |  | -75710.27 | 151464.50 | 151597.10 |  | -75710.27 | 151464.50 | 151636.20 | 0.78 | |
| 9 | 4 | 4234 | 21 |  | -75713.54 | 151469.10 | 151595.60 |  | -75713.54 | 151469.10 | 151632.90 | 0.78 | |
| 10 | 4 | 4224 | 20 |  | -75715.80 | 151471.60 | 151592.10 |  | -75715.80 | 151471.60 | 151627.60 | 0.78 | |

AIC, Akaike information criterion; BIC, Bayesian information criterion; *df*, degrees of freedom

^1^ Phase 1 identified 4 groups as the ideal number of trajectories based on best (lowest) BIC values, less classification uncertainty (entropy > 0.8 as ideal), and interpretability.

^2^ Phase 2 adjusted the polynomial order of trajectories to identify a model with all groups significant at *P* < 0.05, lowest AIC and BIC values, and good fit based on visual inspection of graphs. Based on these criteria, Model 6 was selected.

Table S5. Adequacy of fit measures^1^ for trajectory models selected in Step 1 for diet quality (Model 7) and global cognitive ability (Model 6) in the analytical cohort^2^

| **Trajectory group (description)** | **Sample size, *n*** | **Group membership, %** | **Expected group membership, %** | **Mismatch** | **APP** | **Entropy** |
| --- | --- | --- | --- | --- | --- | --- |
| **Diet quality** |  |  |  |  |  | 0.60 |
| 1 (lower) | 910 | 29.75 | 31.01 | 0.01 | 0.81 |  |
| 2 (moderate) | 1630 | 53.29 | 50.29 | -0.03 | 0.79 |  |
| 3 (higher) | 519 | 16.97 | 18.70 | 0.02 | 0.83 |  |
| **Global cognitive ability** |  |  |  |  |  | 0.78 |
| 1 (lower) | 786 | 25.69 | 25.25 | 0.00 | 0.92 |  |
| 2 (low-moderate) | 631 | 20.63 | 21.21 | 0.01 | 0.81 |  |
| 3 (high-moderate) | 769 | 25.14 | 25.47 | 0.00 | 0.83 |  |
| 4 (higher) | 873 | 28.54 | 28.07 | 0.00 | 0.91 |  |

APP, average posterior probability

^1^ Participants were assigned to whichever group they had the highest posterior probability of membership. Expected group membership was calculated as each group’s maximum posterior probability divided by the total sample size (*n* = 3,059). Mismatch represents the difference between actual and expected group membership. APP was calculated as the average of group participants’ posterior probabilities.

^2^ Refer to Table S3 and Table S4.

**Supplemental Figures**


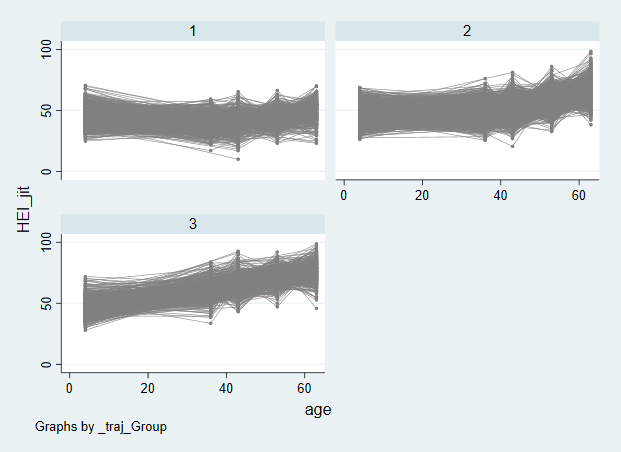


Figure S1. Spaghetti plot showing individual trajectories by diet trajectory groups in the selected three-group model (*n* = 3,059)


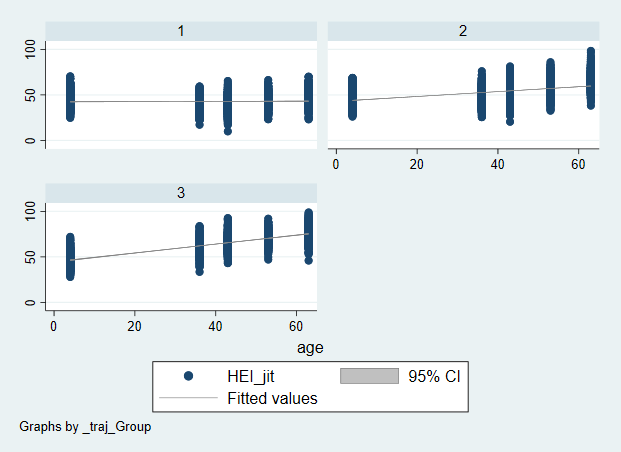


Figure S2. Scatter plot and predicted trend lines for diet trajectory groups in the selected three-group model (*n* = 3,059)


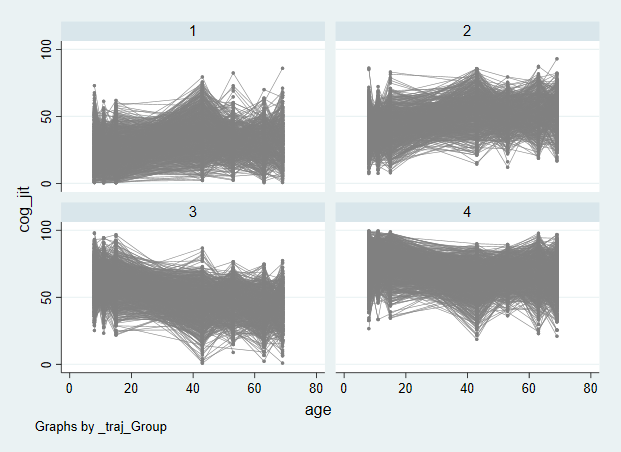


Figure S3. Spaghetti plot showing individual trajectories by cognitive trajectory groups in the selected four-group model (*n* = 3,059)


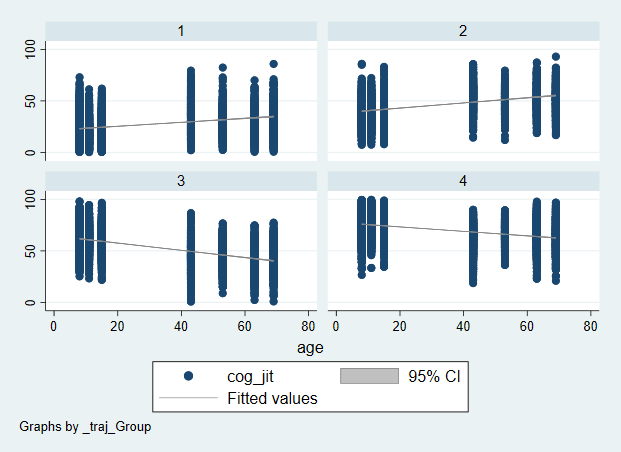


Figure S4. Scatter plot and predicted trend lines for cognitive trajectory groups in the selected four-group model (*n* = 3,059)


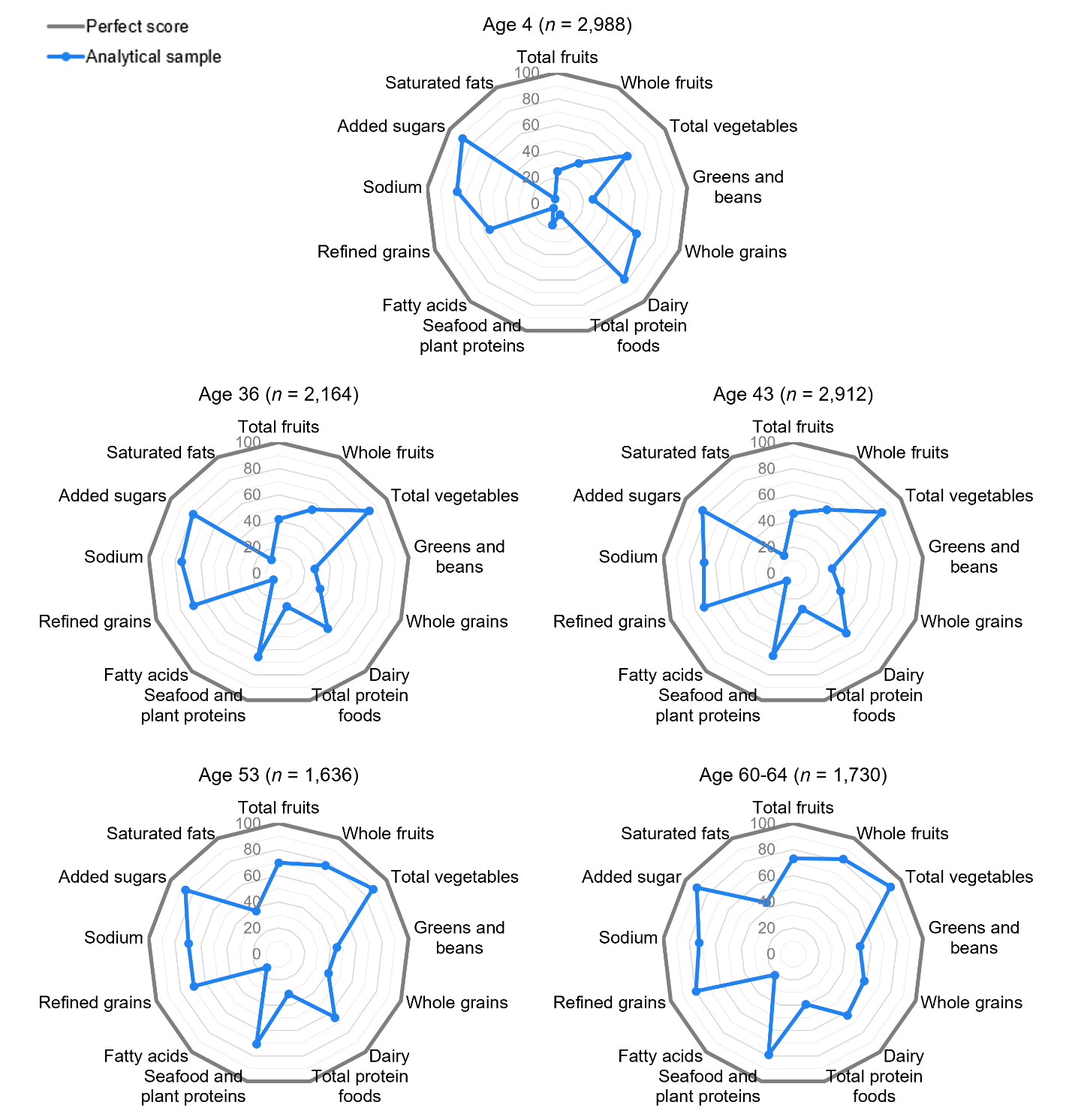


Figure S5. Mean Healthy Eating Index (HEI)-2020 component scores in the full analytical cohort at each data collection period. Higher scores for moderation items (refined grains, sodium, added sugars, saturated fats) indicate lower intake.


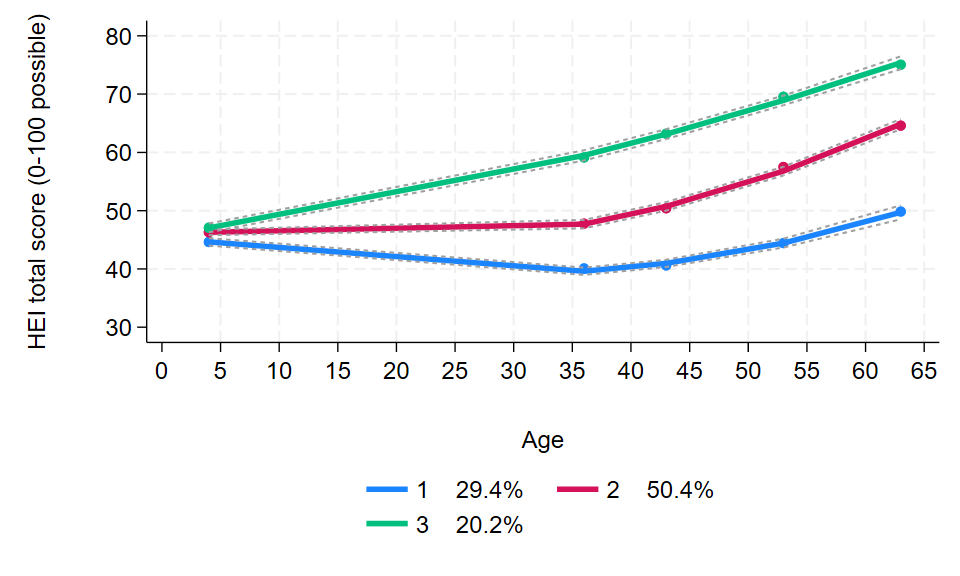

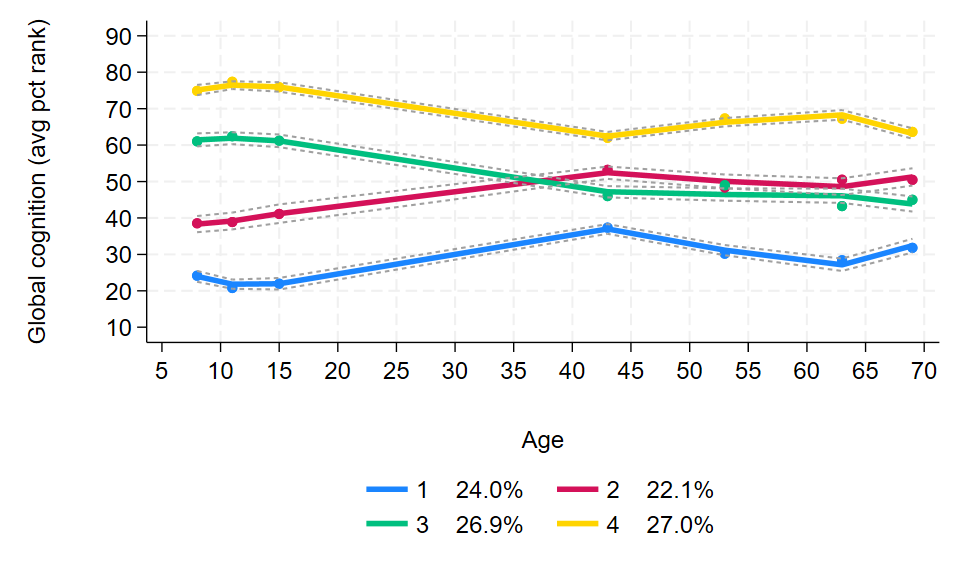


Figure S6. Joint trajectory model combining the unadjusted diet (top) and cognitive (bottom) trajectory models (n = 3,059). Lines = estimated trajectories with 95% CIs, dots = observed group means, legends present expected group membership percentages based on the joint model. avg. pct., average percentile; HEI, Healthy Eating Index
